# National and regional under-5 mortality rate by economic status for low-income and middle-income countries: a systematic assessment

**DOI:** 10.1016/S2214-109X(18)30059-7

**Published:** 2018-04-10

**Authors:** Fengqing Chao, Danzhen You, Jon Pedersen, Lucia Hug, Leontine Alkema

**Affiliations:** a Institute of Policy Studies, Lee Kuan Yew School of Public Policy, National University of Singapore, Singapore; b Division of Data, Research, and Policy, United Nations Children's Fund, New York, NY, USA; c Fafo, Oslo, Norway; d Department of Biostatistics and Epidemiology, School of Public Health and Health Sciences, University of Massachusetts, Amherst, MA, USA

## Abstract

**Background:**

The progress to achieve the fourth Millennium Development Goal in reducing mortality rate in children younger than 5 years since 1990 has been remarkable. However, work remains to be done in the Sustainable Development Goal era. Estimates of under-5 mortality rates at the national level can hide disparities within countries. We assessed disparities in under-5 mortality rates by household economic status in low-income and middle-income countries (LMICs).

**Method:**

We estimated country-year-specific under-5 mortality rates by wealth quintile on the basis of household wealth indices for 137 LMICs from 1990 to 2016, using a Bayesian statistical model. We estimated the association between quintile-specific and national-level under-5 mortality rates. We assessed the levels and trends of absolute and relative disparity in under-5 mortality rate between the poorest and richest quintiles, and among all quintiles.

**Findings:**

In 2016, for all LMICs (excluding China), the aggregated under-5 mortality rate was 64·6 (90% uncertainty interval [UI] 61·1–70·1) deaths per 1000 livebirths in the poorest households (first quintile), 31·3 (29·5–34·2) deaths per 1000 livebirths in the richest households (fifth quintile), and in between those outcomes for the middle quintiles. Between 1990 and 2016, the largest absolute decline in under-5 mortality rate occurred in the two poorest quintiles: 77·6 (90% UI 71·2–82·6) deaths per 1000 livebirths in the poorest quintile and 77·9 (72·0–82·2) deaths per 1000 livebirths in the second poorest quintile. The difference in under-5 mortality rate between the poorest and richest quintiles decreased significantly by 38·8 (90% UI 32·9–43·8) deaths per 1000 livebirths between 1990 and 2016. The poorest to richest under-5 mortality rate ratio, however, remained similar (2·03 [90% UI 1·94–2·11] in 1990, 1·99 [1·91–2·08] in 2000, and 2·06 [1·92–2·20] in 2016). During 1990–2016, around half of the total under-5 deaths occurred in the poorest two quintiles (48·5% in 1990 and 2000, 49·5% in 2016) and less than a third were in the richest two quintiles (30·4% in 1990, 30·5% in 2000, 29·9% in 2016). For all regions, differences in the under-5 mortality rate between the first and fifth quintiles decreased significantly, ranging from 20·6 (90% UI 15·9–25·1) deaths per 1000 livebirths in eastern Europe and central Asia to 59·5 (48·5–70·4) deaths per 1000 livebirths in south Asia. In 2016, the ratios of under-5 mortality rate in the first quintile to under-5 mortality rate in the fifth quintile were significantly above 2·00 in two regions, with 2·49 (90% UI 2·15–2·87) in east Asia and Pacific (excluding China) and 2·41 (2·05–2·80) in south Asia. Eastern and southern Africa had the smallest ratio in 2016 at 1·62 (90% UI 1·48–1·76). Our model suggested that the expected ratio of under-5 mortality rate in the first quintile to under-5 mortality rate in the fifth quintile increases as national-level under-5 mortality rate decreases.

**Interpretation:**

For all LMICs (excluding China) combined, the absolute disparities in under-5 mortality rate between the poorest and richest households have narrowed significantly since 1990, whereas the relative differences have remained stable. To further narrow the rich-and-poor gap in under-5 mortality rate on the relative scale, targeted interventions that focus on the poorest populations are needed.

**Funding:**

National University of Singapore, UN Children's Fund, United States Agency for International Development, and the Bill & Melinda Gates Foundation.

## Introduction

Since 1990, the world has made substantial progress in reducing child mortality. However, continued efforts are needed to ensure further progress and reduce the disparity in child survival across populations. Globally, under-5 mortality rate (the probability of a child dying before age 5 years) reduced by more than 50% in 1990–2016, with significant acceleration in reduction since 2000.^1^ Despite the encouraging advancement in the reduction of child mortality, progress has been uneven across and within countries.^2^ The fourth Millennium Development Goal (MDG),^3^ which was to reduce the under-5 mortality rate by two-thirds between 1990 and 2015, was not achieved in most countries.^4^ Too many children still face very low odds of surviving their first 5 years of life. To continue past efforts to reduce child mortality and complete the unfinished MDG agenda to improve child survival, the Sustainable Development Goals (SDGs) call for an end to preventable deaths of newborns and children by 2030, with all countries aiming to reduce neonatal mortality rates to at least 12 deaths per 1000 livebirths and under-5 mortality rates to at least 25 deaths per 1000 livebirths. Moreover, the SDGs call for disaggregation of reliable data by multiple dimensions including income.^5^ It is important to better understand who and where the most disadvantaged and vulnerable children are at the beginning of the SDG era. The trends in such disparities across countries over time can assist in the understanding of how the benefits of development reach different segments of the population.

**Research in context**

**Evidence before this study**

Data for mortality in children aged under 5 years by household economic status are available from surveys and censuses. Previous studies provided disparity analyses of under-5 mortality rates by household economic status for a subset of low-income and middle-income countries (LMICs) for selected periods between 1990 and 2016. Previous analyses on levels and trends in under-5 mortality rates by economic status mainly relied on direct reporting of the survey and census results.

**Added value of this study**

We estimated under-5 mortality rate by household economic status for all LMICs (excluding China) from 1990 to 2016. To our knowledge, this work covers the widest range of countries among all disparity-related studies of under-5 mortality rate by household economic groups. By contrast with most other studies in which the number of household members in each quintile is the same, we constructed wealth quintiles with equal numbers of births to increase the sample size of births in the richest quintile. This study is the first study to model the association between the ratio of the poorest to richest under-5 mortality rates and the national-level under-5 mortality rate, and to estimate the under-5 mortality rates for all wealth quintile groups. Based on the estimated association, increases in relative disparity are projected to coincide with mortality rate reductions in high-mortality countries. Our study showed that for all LMICs (excluding China) combined, the absolute difference in under-5 mortality rates between the poorest and richest households decreased significantly by 38·8 (90% uncertainty interval [UI] 32·9–43·8) deaths per 1000 livebirths between 1990 and 2016. However, on the relative scale, no significant changes occurred during this period. In 2016, a child born in the poorest quintile faced twice (2·06 [90% UI 1·92–2·20]) the risk of dying before age 5 years compared with a child born in the richest quintile.

**Implications of all the available evidence**

Although the poorest subpopulations in LMICs (excluding China) have been making substantial progress in reducing under-5 mortality rate, even more so than their richest counterparts in terms of absolute reductions, they are not catching up on a relative scale and remain at a disadvantage in most LMICs. Information on disparities in child survival at the country level should form the basis of targeted interventions to reduce the high mortality burden in the poorest subpopulations.

Monitoring of under-5 mortality rate by household economic status is challenging. Countries with good vital registration do not combine mortality data with registry-based economic data, and countries that rely on surveys for mortality estimates usually do not combine mortality surveys with in-depth socioeconomic surveys, such as household income and expenditure surveys. Estimates of under-5 mortality rates by household economic strata, or disparities in under-5 mortality rates between rich and poor households, have been published previously for either one country^6^, ^7^, ^8^, ^9^, ^10^, ^11^ or multiple countries.^12^, ^13^, ^14^, ^15^, ^16^, ^17^, ^18^, ^19^, ^20^, ^21^, ^22^ Before this study, the Health Equity Monitor by WHO provided the most comprehensive information on under-5 mortality rate by household wealth quintile for country-years with available data from Demographic and Health Surveys and Multiple Indicator Cluster Surveys, including 71 countries.^18^ However, no studies provided time trends covering 1990–2016 or included all low-income and middle-income countries (LMICs).

In this study, we estimated levels and trends in under-5 mortality rate by wealth quintile (a measure of household economic status) for 137 LMICs from 1990 to 2016. In our model, the association between ratios among quintile-specific and national-level under-5 mortality rate was assessed by use of all available survey data and was modelled with a flexible splines regression model. We used the national-level estimates of under-5 mortality rate to construct quintile-specific mortality rate for all country-years, including those where such data are unavailable. We identified regions and countries with the largest and smallest absolute and relative disparities in under-5 mortality rate.

## Methods

### Data

**Figure 1:**
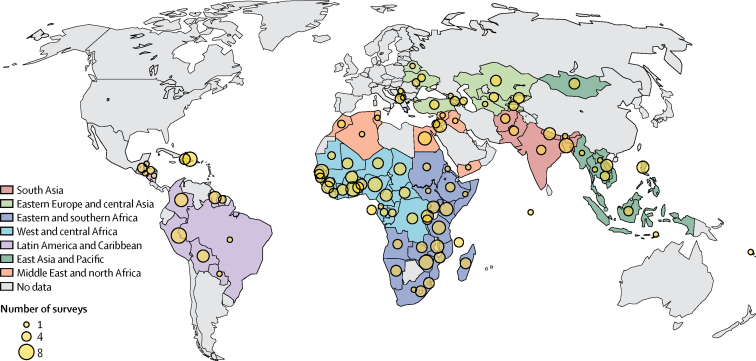
Data availability Countries are coloured by regions. Circle size is proportional to the number of datapoints available for each country. This map does not reflect a position by the UN Inter-agency Group for Child Mortality Estimation agencies or those of the institutions to which the authors are affiliated on the legal status of any country or territory. The dotted line represents approximately the Line of Control in Jammu and Kashmir agreed upon by India and Pakistan. The final status of Jammu and Kashmir has not yet been agreed upon by the parties. The final boundary between Sudan and South Sudan has not yet been determined. The final status of the Abyei area has not yet been determined. The borders are not up to the UN standard

The quintile-specific under-5 mortality rate refers to the probability of children aged under 5 years, born in households of a specific wealth quintile, to die before reaching 5 years. The data used in our systematic assessment are observed under-5 mortality rates by wealth quintile from Demographic and Health Surveys and Multiple Indicator Cluster Surveys done between 1990 and 2016 in 99 LMICs (figure 1, appendix p 13). In August, 2017, the database contained information from 319 surveys, with one to nine available surveys per country (appendix pp 41–52).

Demographic and Health Surveys and Multiple Indicator Cluster Surveys use a wealth index (ie, the asset index) composed of a set of variables asked in household questionnaires that describe household assets and utility services.^23^ The wealth index is used as a proxy for household welfare. The variables constituting the wealth index vary across surveys, reflecting different conditions in different countries as well as technological advances. The wealth index for a survey is constructed by use of a principal component analysis of the wealth-related variables available for that survey; the index is given by the object scores for the first principal component. Typically, the quintiles are constructed so that each quintile contains 20% of the population of individuals. Under this approach, the number of births in the poorest households tends to be higher than in the wealthier quintiles because fertility is usually higher among women in poor households. In our study, division into quintiles was based on the product of the sampling weight and number of births to include equal numbers of births in each quintile.

These two survey programs also collect retrospective information on child mortality through use of birth histories. For surveys that included full birth histories, which collect detailed information on each child including date of birth and age at death, we calculated quintile-specific under-5 mortality rate in the 5 years before the survey, as opposed to a longer retrospective period, to reduce the effect of household wealth changing over time and potential recall bias. For surveys that collected summary birth histories, which only gather information on the number of children ever born and the number of children who died or still survive, an indirect method based on time since first birth was used to estimate under-5 mortality rate for each quintile.^24^ Mortality is calculated on the basis of births and deaths that occurred to mothers who had their first birth 5–9 years before the time of the survey.

### Statistical analysis

We developed a statistical model to estimate levels and trends in under-5 mortality rate by wealth quintile over time. The model took, as an input, the ratio of quintile-specific under-5 mortality rate to the national-level under-5 mortality rate from empirical data, and the estimates of national-level under-5 mortality rate (for all quintiles combined) produced by the UN Inter-agency Group for Child Mortality Estimation (IGME) in 2017.^1^ The model produced disaggregated estimates of under-5 mortality rate by the five wealth quintiles. Details on the model specification, implementation, and validation are presented in the appendix (pp 3–9).

In our model, under-5 mortality rate for the first, second, fourth, and fifth quintiles were modelled relative to the under-5 mortality rate for the third quintile and referred to as third quintile-disparity ratios (with the third quintile serving as the reference point). This approach was used to exchange information within countries over time and across countries on the expected third quintile-disparity ratios, and to incorporate the constraint that the sum of quintile-specific under-5 deaths is equal to the total number of under-5 deaths. National-level under-5 mortality rate (excluding crisis-related deaths) was used to predict the expected third quintile-disparity ratios on the basis of an expected (and empirically observed) association between the ratios and national-level under-5 mortality rate, by use of a flexible (penalised B-splines) regression model. The splines were used to capture the potentially non-linear association between the expected third quintile-disparity ratios and the national-level under-5 mortality rate (appendix pp 4–5). The final country-specific third quintile-disparity ratios were modelled as the product of the expected ratios (based on the national-level under-5 mortality rate in the country-year) and a country-year-specific multiplier. The multiplier represents the deviation of the actual country-specific ratio away from its expected level, as indicated by country-specific data. The country-year-specific levels of the multiplier were assumed to fluctuate around 1 (appendix p 3). The multiplier was modelled on the log-scale by a time series model of a first order autoregressive process (ie, AR[1]) structure, with quintile-specific autoregressive parameters and global distortion variance. We simultaneously estimated the expected third quintile-disparity ratios and final country-specific ratios. We used the observed ratio of the quintile-specific under-5 mortality rate to the national-level under-5 mortality rate as data input for model fitting to reduce the effect of level biases in under-5 mortality rate data.^25^ The data quality model incorporated sampling variance that considers survey design.

We used a Markov Chain Monte Carlo algorithm to generate samples from the posterior distribution of the parameters.^26^ This approach produced a set of trajectories of third quintile-disparity ratios or, equivalently, ratios of quintile-specific to national-level under-5 mortality rate for each country. Estimates of the final third quintile-disparity ratios were combined with estimates of national-level under-5 mortality rate to obtain country-year, quintile-specific, under-5 mortality rate, accounting for the uncertainty in the national-level under-5 mortality rate.^1^ Estimates for countries without data followed from the model and its parameter estimates and were based on the expected third quintile-disparity ratios (determined by the national-level under-5 mortality rate for that country), the uncertainty in country-specific deviations based on simulations of the country-year-specific multiplier (that captured the variability unexplained by the expected third quintile-disparity ratios), and the uncertainty in national-level under-5 mortality rate. The quintile-specific under-5 mortality rate and corresponding deaths were adjusted to account for crisis-related under-5 deaths.^1^ Aggregated estimates of under-5 mortality rate by quintile were derived through application of the proportions of quintile-specific under-5 deaths within a region to the aggregated UN IGME under-5 mortality rate in a region.^1^ We constructed aggregated results for all LMICs (excluding China) using the World Bank income group classification (appendix p 13).^27^ We computed 90% uncertainty intervals (UIs) for all indicators of interest using the fifth and 95th percentiles of the posterior distributions (90% UIs are the standard choice in UN IGME reporting as opposed to the standard 95% intervals given the inherent uncertainty in child mortality related outcomes).

Model performance was assessed through use of an out-of-sample validation (appendix pp 9–10). Validation results suggested that our model was reasonably well calibrated, with generally conservative UIs (ie, wider than expected). The Markov Chain Monte Carlo sampling algorithm was implemented by use of JAGS 4.0.1 Open Source software,^28^ and the analysis was done in R version 3.2.2. Software programs and data are available from the authors.

### Equity analyses

We assessed the household economic disparities in under-5 mortality rate on the absolute and relative scales at the national, regional, and aggregated levels of 137 LMICs. Because absolute and relative measures can lead to different conclusions about the size of and changes in disparities, assessment of absolute and relative measures is important to present a complete picture in disparity.^29^ We calculated two absolute indicators of inequality: the difference between under-5 mortality rate in the poorest (first) and richest (fifth) quintiles, and the slope index of inequality, which captures inequity across all five quintile groups. The slope index is the slope of regression of quintile-specific under-5 mortality rate on its cumulative proportion of livebirths up to the midpoint of each quintile from the poorest to the richest.^13^, ^30^ This index represents the change in quintile-specific under-5 mortality rate (deaths per 1000 livebirths) when the economic status of birth increases from the poorest to the richest.^31^ We also calculated two relative inequality indicators: the ratio of the under-5 mortality rate in the poorest quintile to the richest quintile, and the concentration index. The concentration index captures the inequality across all quintiles and is calculated as twice the area between the mortality concentration curve (the cumulative proportion of under-5 deaths against the cumulative proportion of livebirths, beginning with the poorest quintile) and the diagonal.^13^ The concentration index is expressed in a scale ranging from −100 to 100; a value of 0 represents perfect equality, whereas a value equal to 100 or −100 indicates that only the richest or the poorest households bear the burden of under-5 mortality rate.

### Role of the funding source

The sponsors of the study had no role in the study design, data analysis, data interpretation, or writing of the report. The corresponding author had full access to all data in the study and had final responsibility for the decision to submit for publication.

## Results

**Figure 2:**
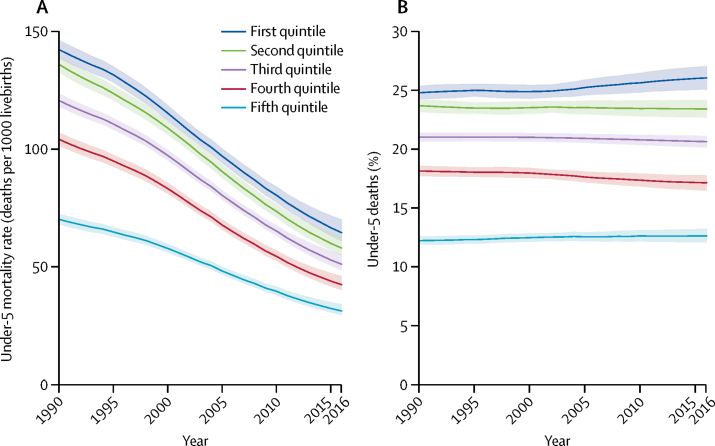
Quintile-specific under-5 mortality rate from 1990 to 2016, for all low-income and middle-income countries (excluding China) combined (A) Under-5 mortality rate and (B) percentage of under-5 deaths by year. Curves are point estimates. Shaded areas are 90% uncertainty intervals. The first quintile is the 20% poorest quintile and the fifth quintile is the 20% richest.

For all 137 LMICs (excluding China) combined, in 2016 the under-5 mortality rate was 64·6 (90% UI 61·1 to 70·1) deaths per 1000 livebirths in the poorest quintile, 31·3 (29·5 to 34·2) in the richest quintile, and in between those outcomes in the middle quintiles (table 1, figure 2). The under-5 mortality rate decreased significantly in all quintiles between 1990 and 2016, with greater point estimates of average yearly absolute and percentage declines observed in 2000–16 than in 1990–2000. The largest absolute declines in under-5 mortality rate between 1990 and 2016 occurred in the two poorest quintiles with 77·6 (90% UI 71·2 to 82·6) deaths per 1000 livebirths in the first quintile and 77·9 (72·0 to 82·2) in the second quintile. The corresponding percentage declines between 1990 and 2016 were 54·6% (50·6 to 57·3) in the first quintile and 57·3% (53·6 to 59·7) in the second quintile (table 1), which are similar to the percentage declines in other quintiles. Because of the greater absolute decline in under-5 mortality rate in the poorest quintile than in the richest quintile, the difference between these quintiles decreased significantly from 72·0 (90% UI 67·7 to 76·5) deaths per 1000 livebirths in 1990 to 57·4 (53·9 to 61·1) in 2000, and 33·2 (29·9 to 37·6) in 2016. Similarly, the absolute disparity in under-5 mortality rate among all five quintiles, measured by the slope index of inequality, narrowed significantly over time and shifted closer to zero from −87·9 (90% UI −92·8 to −83·1) deaths per 1000 livebirths in 1990, to −70·3 (–74·2 to −66·3) in 2000, and −41·0 (–45·9 to −37·4) in 2016. The relative disparity between the poorest and richest under-5 mortality rates, however, remained similar; the ratio of under-5 mortality rate in the first quintile to fifth quintile was 2·03 (90% UI 1·94 to 2·11) in 1990, 1·99 (1·91 to 2·08) in 2000, and 2·06 (1·92 to 2·20) in 2016. The relative disparity across all quintiles, measured by the concentration index, also had minor fluctuations only between 1990 and 2016 (table 1).

**Table 1:** Estimates and 90% uncertainty intervals for quintile-specific under-5 mortality rate in 1990, 2000, and 2016, for all low-income and middle-income countries (excluding China) combined

	**Under-5 mortality rate (deaths per 1000 livebirths)**			**Absolute decline in under-5 mortality rate (deaths per 1000 livebirths)**			**Percentage decline in under-5 mortality rate (%)**			**Quintile-specific to total under-5 deaths (%)**		
	1990	2000	2016	Average decline per year		Total decline (1990–2016)	Average decline per year		Total decline (1990–2016)	1990	2000	2016
				1990–2000	2000–2016		1990–2000	2000–16				
First quintile (poorest)	142·2 (138·6 to 146·2)	115·3 (112·2 to 118·5)	64·6 (61·1 to 70·1)	2·7 (2·3 to 3·1)	3·2 (2·8 to 3·4)	77·6 (71·2 to 82·6)	2·1% (1·8 to 2·4)	3·6% (3·1 to 3·9)	54·6% (50·6 to 57·3)	24·8% (24·3 to 25·4)	24·9% (24·3 to 25·5)	26·1% (25·1 to 27·1)
Second quintile	135·9 (132·6 to 139·4)	108·9 (106·2 to 112·0)	58·0 (54·9 to 63·1)	2·7 (2·3 to 3·1)	3·2 (2·8 to 3·4)	77·9 (72·0 to 82·2)	2·2% (1·9 to 2·5)	3·9% (3·4 to 4·2)	57·3% (53·6 to 59·7)	23·7% (23·2 to 24·2)	23·6% (23·1 to 24·1)	23·4% (22·7 to 24·2)
Third quintile	120·6 (118·0 to 123·5)	97·3 (95·2 to 99·6)	51·1 (48·6 to 55·3)	2·3 (2·1 to 2·6)	2·9 (2·6 to 3·1)	69·4 (64·6 to 73·0)	2·1% (1·9 to 2·3)	3·9% (3·5 to 4·3)	57·6% (54·1 to 59·8)	21·0% (20·7 to 21·4)	21·0% (20·7 to 21·4)	20·7% (20·2 to 21·2)
Fourth quintile	104·1 (101·4 to 107·0)	83·2 (81·1 to 85·7)	42·5 (40·2 to 46·1)	2·1 (1·8 to 2·4)	2·5 (2·3 to 2·7)	61·6 (57·2 to 65·1)	2·2% (1·9 to 2·5)	4·1% (3·6 to 4·5)	59·2% (55·6 to 61·6)	18·2% (17·7 to 18·6)	18·0% (17·6 to 18·4)	17·2% (16·5 to 17·8)
Fifth quintile (richest)	70·2 (68·0 to 72·5)	57·8 (56·1 to 59·7)	31·3 (29·5 to 34·2)	1·2 (1·0 to 1·4)	1·7 (1·5 to 1·8)	38·9 (35·4 to 41·4)	1·9% (1·6 to 2·2)	3·8% (3·2 to 4·1)	55·4% (51·2 to 58·0)	12·2% (11·9 to 12·6)	12·5% (12·2 to 12·9)	12·7% (12·1 to 13·3)
Ratio of first to fifth quintile mortality rate	2·03 (1·94 to 2·11)	1·99 (1·91 to 2·08)	2·06 (1·92 to 2·20)	..	..	..	..	..	..	..	..	..
Difference in first and fifth quintile mortality rate	72·0 (67·7 to 76·5)	57·4 (53·9 to 61·1)	33·2 (29·9 to 37·6)	..	..	..	..	..	..	..	..	..
Concentration index (× 100)	−12·3 (−12·9 to −11·6)	−12·2 (−12·8 to −11·5)	−13·3 (−14·3 to −12·2)	..	..	..	..	..	..	..	..	..
Slope index of inequality (deaths per 1000 livebirths)	−87·9 (−92·8 to −83·1)	−70·3 (−74·2 to −66·3)	−41·0 (−45·9 to −37·4)	..	..	..	..	..	..	..	..	..

Although the absolute burden of under-5 deaths decreased for all LMICs (excluding China) combined, the distribution of under-5 deaths across quintiles remained stable since 1990 (table 1, figure 2). During 1990–2016, around half of the total under-5 deaths were of children born in the poorest two quintiles (48·5% [90% UI 48·0–49·1] in 1990, 48·5% [47·9–49·0] in 2000, and 49·5% [48·6–50·4] in 2016) and only less than a third were from the richest two quintiles (30·4% [29·9–30·9] in 1990, 30·5% [30·0–31·0] in 2000, 29·9% [29·0–30·6] in 2016). In 2016 alone, among the total of 5·41 (90% UI 5·17–5·81) million under-5 deaths in LMICs (excluding China), an estimated 1·41 (1·33–1·53) million children died in the poorest quintile compared with 0·68 (0·65–0·75) million in the richest quintile (appendix p 26).

**Figure 3:**
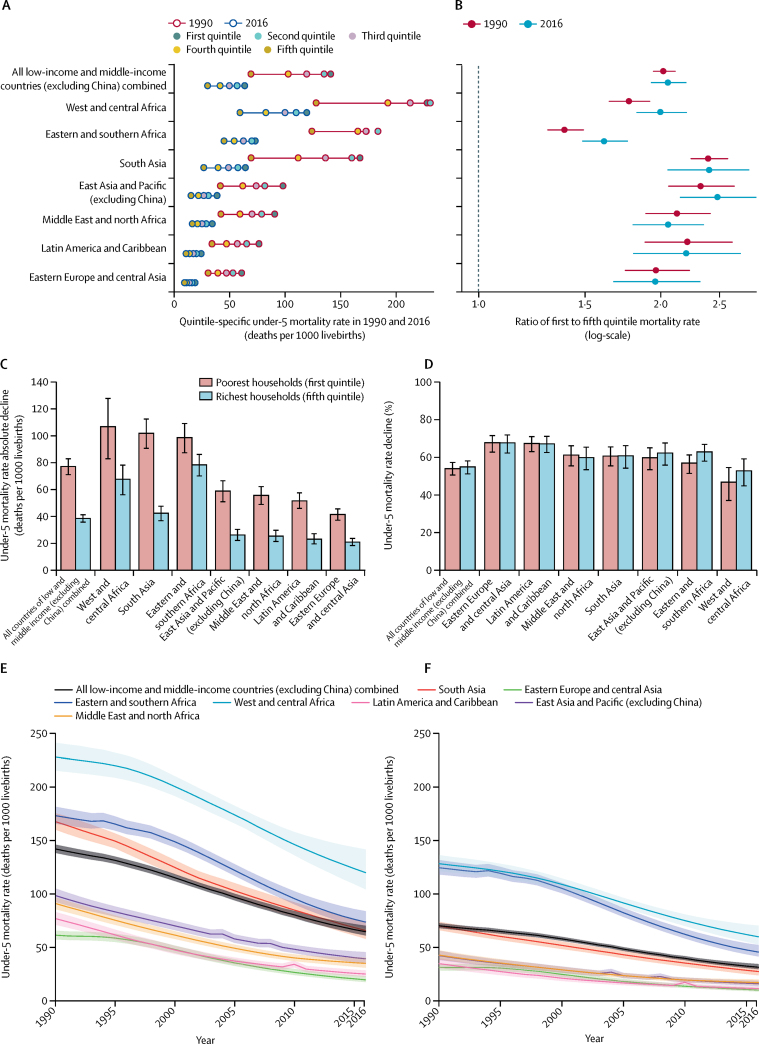
Under-5 mortality rate in 1990 and 2016, for all low-income and middle-income countries (excluding China) combined and by region (A) Point estimates for under-5 mortality rate in each quintile in 1990 and 2016. Coloured dots show the point estimates for under-5 mortality rate in each quintile. The first quintile is the 20% poorest quintile and the fifth quintile is the 20% richest. The distance between the first and fifth quintiles represents the difference in first and fifth quintile mortality rate. Regions are in descending order of point estimates for under-5 mortality rate in the first quintile in 2016. (B) Ratio of first to fifth quintile mortality rate. Dots are point estimates. Error bars denote 90% uncertainty intervals. (C) Absolute decline and (D) percentage decline in under-5 mortality rate in the first (poorest) and fifth (richest) quintiles in 1990–2016. Error bars represent 90% uncertainty intervals. Regions are in descending order of point estimates in the first quintile. (E) Poorest (first) quintile under-5 mortality rate and (F) richest (fifth) quintile under-5 mortality rate. Curves are point estimates. Shaded areas are 90% uncertainty intervals.

Under-5 mortality rate in the first and fifth quintiles in the 137 LMICs varied across regions over time (table 2, figure 3). In 2016, at the regional level, under-5 mortality rate in the first quintile ranged from 19·4 (90% UI 17·4 to 22·9) deaths per 1000 livebirths in eastern Europe and central Asia to 119·9 (104·3 to 141·6) deaths per 1000 livebirths in west and central Africa, and mortality in the fifth quintile ranged from 9·9 (8·6 to 12·0) deaths per 1000 livebirths in eastern Europe and central Asia to 59·8 (52·6 to 70·3) deaths per 1000 livebirths in west and central Africa. In 2016, the ratios of under-5 mortality rate in the first quintile to fifth quintile were found to be significantly above 2·00 in east Asia and Pacific (excluding China) at 2·49 (90% UI 2·15 to 2·87) and in south Asia at 2·41 (2·05 to 2·80). Eastern and southern Africa had the smallest ratio in 2016 of 1·62 (90% UI 1·48 to 1·76) and its concentration index was the closest to zero at −9·4 (–10·8 to −8·0) per 100 (table 3). Compared with all regions studied, west and central Africa and south Asia had the largest absolute differences in under-5 mortality rate between the first and fifth quintiles in 2016 (60·1 [90% UI 49·2 to 75·1] deaths per 1000 livebirths and 38·4 [30·9 to 46·2] deaths per 1000 livebirths; table 2). The region with the smallest absolute disparity in 2016 was eastern Europe and central Asia; the difference between the first quintile and fifth quintile was 9·6 (90% UI 7·3 to 12·5) deaths per 1000 livebirths (table 2), and the slope index of inequality was −12·0 (–15·2 to −9·6) deaths per 1000 livebirths (table 3).

**Table 2:** Estimates and 90% uncertainty intervals for under-5 mortality rate in the first and fifth quintiles, differences between under-5 mortality rate in the first and fifth quintiles, and ratios of under-5 mortality rate in the first quintile to fifth quintile in 1990, 2000, and 2016, for regions

	**Under-5 mortality rate in first quintile (deaths per 1000 livebirths)**			**Under-5 mortality rate in fifth quintile (deaths per 1000 livebirths)**			**Difference in first and fifth quintile mortality rate (deaths per 1000 livebirths)**			**Ratio of first to fifth quintile mortality rate**		
	1990	2000	2016	1990	2000	2016	1990	2000	2016	1990	2000	2016
South Asia	167·8 (160·4–175·6)	124·8 (118·1–131·6)	65·7 (58·0–73·9)	69·8 (66·1–73·8)	51·8 (48·4–55·3)	27·2 (23·8–31·7)	98·0 (89·7–106·4)	73·1 (65·4–80·5)	38·4 (30·9–46·2)	2·40 (2·24–2·58)	2·41 (2·21–2·62)	2·41 (2·05–2·80)
Eastern Europe and central Asia	61·3 (57·4–65·6)	48·0 (44·8–51·6)	19·4 (17·4–22·9)	31·1 (28·3–34·4)	24·6 (22·2–27·2)	9·9 (8·6–12·0)	30·2 (25·0–35·6)	23·4 (19·3–27·7)	9·6 (7·3–12·5)	1·97 (1·75–2·23)	1·95 (1·73–2·21)	1·97 (1·67–2·32)
Eastern and southern Africa	173·4 (165·9–181·7)	149·0 (143·8–155·1)	73·6 (67·5–83·7)	124·5 (118·3–131·7)	105·2 (100·8–110·3)	45·5 (41·4–52·0)	48·9 (38·9–59·0)	43·8 (37·4–50·4)	28·1 (23·0–34·7)	1·39 (1·30–1·49)	1·42 (1·35–1·49)	1·62 (1·48–1·76)
West and central Africa	228·2 (215·6–241·5)	200·7 (191·9–210·4)	119·9 (104·3–141·6)	128·3 (121·3–136·3)	108·9 (103·7–114·5)	59·8 (52·6–70·3)	99·9 (85·5–114·2)	91·8 (82·7–101·4)	60·1 (49·2–75·1)	1·78 (1·64–1·92)	1·84 (1·74–1·95)	2·00 (1·82–2·21)
Latin America and Caribbean	76·9 (71·2–83·0)	47·5 (43·8–51·2)	24·9 (22·3–28·2)	34·6 (30·4–39·6)	21·0 (18·4–23·9)	11·3 (9·7–13·3)	42·3 (34·2–50·3)	26·5 (21·3–31·5)	13·6 (10·2–17·4)	2·22 (1·88–2·62)	2·26 (1·91–2·67)	2·21 (1·80–2·71)
East Asia and Pacific (excluding China)	98·5 (92·6–105·0)	70·1 (66·2–74·6)	39·2 (34·6–45·4)	42·2 (38·2–46·6)	28·8 (26·2–31·9)	15·7 (13·6–18·6)	56·3 (48·4–64·5)	41·3 (36·2–46·7)	23·5 (19·2–28·4)	2·34 (2·06–2·64)	2·43 (2·17–2·73)	2·49 (2·15–2·87)
Middle East and north Africa	91·1 (85·9–96·8)	61·5 (57·9–65·0)	34·9 (30·9–40·4)	42·7 (38·7–47·0)	28·9 (26·3–31·6)	16·9 (14·8–19·9)	48·4 (41·0– 55·9)	32·6 (27·9–37·1)	18·0 (14·5–22·3)	2·13 (1·89–2·41)	2·13 (1·90–2·37)	2·06 (1·80–2·36)

The first quintile is the 20% poorest quintile and the fifth quintile is the 20% richest quintile.

**Table 3:** Estimates and 90% uncertainty intervals for concentration index and slope inequality index in 1990, 2000, and 2016, for regions

	**Concentration index (×100)**			**Slope inequality index (deaths per 1000 livebirths)**		
	1990	2000	2016	1990	2000	2016
South Asia	−15·1 (−16·2 to −14·0)	−15·6 (−16·9 to −14·2)	−15·7 (−18·1 to −13·2)	−122·1 (−131·5 to −112·8)	−91·3 (−99·6 to −82·8)	−47·3 (−56·0 to −39·1)
Eastern Europe and central Asia	−12·7 (−14·5 to −10·8)	−12·9 (−14·8 to −10·9)	−13·3 (−16·1 to −10·6)	−37·0 (−42·6 to −31·5)	−29·2 (−33·8 to −24·8)	−12·0 (−15·2 to −9·6)
Eastern and southern Africa	−5·6 (−6·7 to −4·6)	−6·0 (−6·8 to −5·1)	−9·4 (−10·8 to −8·0)	−57·9 (−69·3 to −46·9)	−51·5 (−58·6 to −44·4)	−36·0 (−43·5 to −30·4)
West and central Africa	−9·6 (−10·8 to −8·3)	−10·3 (−11·2 to −9·3)	−12·5 (−14·0 to −10·9)	−119·0 (−134·8 to −102·5)	−110·9 (−121·8 to −100·4)	−73·7 (−91·8 to −61·3)
Latin America and Caribbean	−14·5 (−16·9 to −12·1)	−15·1 (−17·5 to −12·6)	−15·1 (−18·3 to −11·9)	−51·3 (−59·7 to −42·7)	−32·3 (−37·5 to −26·8)	−16·7 (−20·8 to −13·1)
East Asia and Pacific (excluding China)	−14·7 (−16·6 to −12·9)	−15·7 (−17·4 to −14·0)	−16·4 (−18·6 to −14·1)	−66·2 (−74·9 to −57·9)	−49·0 (−54·7 to −43·7)	−27·9 (−33·5 to −23·4)
Middle East and north Africa	−13·5 (−15·4 to −11·7)	−13·7 (−15·4 to −12·0)	−13·6 (−15·8 to −11·4)	−58·2 (−66·1 to −50·2)	−39·1 (−43·9 to −34·1)	−21·9 (−26·8 to −18·0)

From 1990 to 2016 across all regions, under-5 mortality rate in the first and fifth quintiles decreased significantly. For both these mortality rates, based on point estimates, greater average, yearly, absolute declines were seen after 2000 than during 1990–2000 in eastern Europe and central Asia, eastern and southern Africa, and west and central Africa (table 2). Because of substantial absolute declines in under-5 mortality rate in the first and fifth quintiles, the rates in 2016 were less than half of those in 1990 for all regions except for west and central Africa with a 47·5% (90% UI 37·2 to 54·6) decline in under-5 mortality rate in the first quintile. Absolute declines in under-5 mortality rate were greater in the first quintile than in the fifth quintile in all regions during 1990–2016. Consequently, differences between under-5 mortality rate in the first and fifth quintiles decreased significantly for all regions, with the decreases ranging from −20·6 (90% UI −25·1 to −15·9) deaths per 1000 livebirths in eastern Europe and central Asia to −59·5 (–70·4 to −48·5) deaths per 1000 livebirths in south Asia (table 4). For all regions, in 2016, under-5 mortality rate was the highest in the first quintile and the lowest in the fifth quintile (figure 3). Similarly, in 1990, under-5 mortality rate in the fifth quintile was the lowest quintile-specific under-5 mortality rate for all regions and under-5 mortality rate in the first quintile was the highest, except for eastern and southern Africa, and west and central Africa, where the under-5 mortality rate in the second quintile was lower than in the first. On relative scale, the changes in the ratios of under-5 mortality rate in the first quintile to fifth quintile were significantly different from zero in two sub-Saharan African regions only: the ratios increased between 1990 and 2016 in eastern and southern Africa, and west and central Africa.

**Table 4:** Estimates and 90% uncertainty intervals for absolute decline and percentage decline in under-5 mortality rate in the first and fifth quintiles, and the change in ratios in 1990–2016, 1990–2000, and 2000–16, for regions

	**Absolute decline in first quintile under-5 mortality rate (deaths per 1000 livebirths)**			**Absolute decline in fifth quintile under-5 mortality rate (deaths per 1000 livebirths)**			**Percentage decline in first quintile under-5 mortality rate (%)**			**Percentage decline in fifth quintile under-5 mortality rate (%)**			Change in difference*(deaths per 1000 livebirths), 1990–2016	**Change in ratio**†**(1990–2016)**
	Average decline per year		Total decline (1990–2016)	Average decline per year		Total decline (1990–2016)	Average decline per year		Total decline (1990–2016)	Average decline per year		Total decline (1990–2016)		
	1990–2000	2000–16		1990–2000	2000–16		1990–2000	2000–16		1990–2000	2000–16			
South Asia	4·3 (3·4 to 5·2)	3·7 (3·1 to 4·3)	102·1 (90·8 to 112·6)	1·8 (1·4 to 2·2)	1·5 (1·2 to 1·8)	42·6 (36·9 to 47·7)	2·9% (2·3 to 3·5)	3·9% (3·2 to 4·7)	60·9% (55·5 to 65·6)	3·0% (2·2 to 3·6)	3·9% (3·0 to 4·8)	61·0% (54·3 to 66·3)	−59·5 (−70·4 to −48·5)	0·01 (−0·38 to 0·41)
Eastern Europe and central Asia	1·3 (1·0 to 1·6)	1·8 (1·5 to 2·0)	41·9 (37·4 to 45·8)	0·7 (0·5 to 0·8)	0·9 (0·8 to 1·0)	21·3 (18·5 to 23·8)	2·4% (1·8 to 3·0)	5·5% (4·6 to 6·1)	68·3% (62·8 to 71·6)	2·3% (1·7 to 2·9)	5·5% (4·5 to 6·2)	68·2% (62·4 to 71·9)	−20·6 (−25·1 to −15·9)	0·00 (−0·26 to 0·28)
Eastern and southern Africa	2·4 (1·8 to 3·1)	4·7 (4·1 to 5·2)	99·9 (87·5 to 109·3)	1·9 (1·4 to 2·5)	3·7 (3·3 to 4·1)	79·1 (70·2 to 86·3)	1·5% (1·1 to 1·9)	4·3% (3·5 to 4·8)	57·6% (51·5 to 61·4)	1·7% (1·2 to 2·1)	5·1% (4·3 to 5·6)	63·5% (58·0 to 67·0)	−20·8 (−31·1 to −9·6)	0·23 (0·07 to 0·38)
West and central Africa	2·7 (1·7 to 3·9)	5·1 (3·6 to 6·1)	108·3 (83·0 to 127·9)	1·9 (1·3 to 2·6)	3·1 (2·4 to 3·6)	68·5 (56·3 to 78·3)	1·3% (0·8 to 1·8)	3·2% (2·1 to 4·0)	47·5% (37·2 to 54·6)	1·6% (1·1 to 2·1)	3·7% (2·7 to 4·4)	53·4% (44·9 to 59·2)	−39·8 (−57·3 to −19·1)	0·23 (0·00 to 0·47)
Latin America and Caribbean	2·9 (2·5 to 3·4)	1·4 (1·2 to 1·6)	52·0 (46·1 to 57·7)	1·4 (1·1 to 1·7)	0·6 (0·5 to 0·7)	23·4 (19·7 to 27·3)	4·7% (4·1 to 5·3)	3·9% (3·2 to 4·5)	67·6% (63·1 to 71·1)	4·9% (4·2 to 5·6)	3·5% (2·8 to 4·1)	67·5% (62·6 to 71·2)	−28·6 (−35·7 to −21·2)	−0·01 (−0·38 to 0·40)
East Asia and Pacific (excluding China)	2·8 (2·3 to 3·4)	1·9 (1·5 to 2·3)	59·3 (51·0 to 66·7)	1·3 (1·1 to 1·6)	0·8 (0·6 to 1·0)	26·4 (22·3 to 30·5)	3·3% (2·8 to 3·9)	3·6% (2·7 to 4·3)	60·2% (53·5 to 65·2)	3·7% (3·1 to 4·4)	3·6% (2·7 to 4·4)	62·7% (55·9 to 67·7)	−32·9 (−40·8 to −24·6)	0·15 (−0·19 to 0·51)
Middle East and north Africa	3·0 (2·6 to 3·4)	1·7 (1·3 to 1·9)	56·2 (49·1 to 62·3)	1·4 (1·1 to 1·7)	0·7 (0·6 to 0·9)	25·8 (21·5 to 29·9)	3·9% (3·4 to 4·4)	3·5% (2·6 to 4·2)	61·7% (55·5 to 66·2)	3·8% (3·2 to 4·5)	3·3% (2·4 to 4·0)	60·4% (53·5 to 65·5)	−30·4 (−37·5 to −22·9)	−0·07 (−0·37 to 0·23)

The first quintile is the 20% poorest quintile and the fifth quintile is the 20% richest quintile.

* Difference between under-5 mortality rate in first and fifth quintile.

† Ratio of mortality rate in first to fifth quintile.

**Figure 4:**
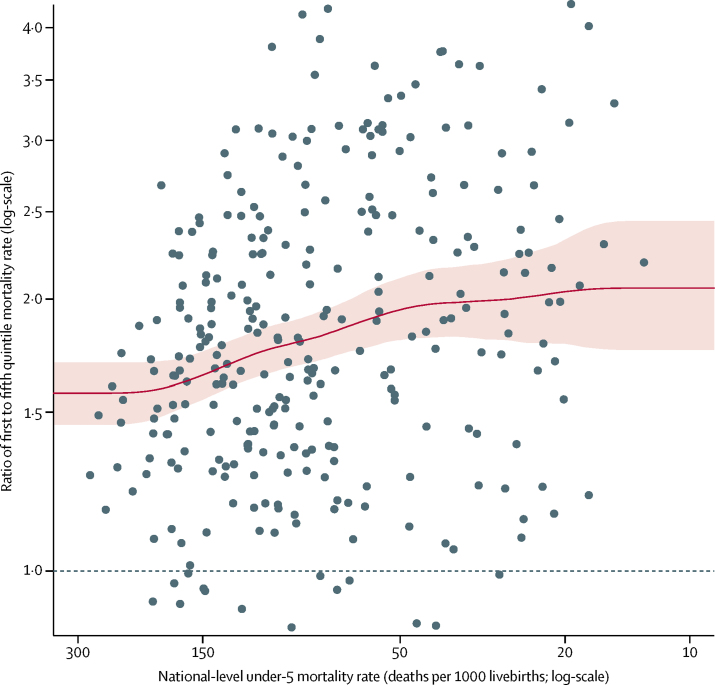
Overview of the average association between the ratio of under-5 mortality rate in the first to fifth quintiles and national-level under-5 mortality rate Observed ratios of first to fifth quintile mortality rate are plotted against decreasing national-level under-5 mortality rate (grey dots) and the model results of the average association between ratios of first to fifth quintile mortality rate and national-level under-5 mortality rate are in red. Curve is point estimates. Shaded area is 90% uncertainty intervals.

Our model suggested an inverse association between the ratio of under-5 mortality rate in the poorest quintile to richest quintile and national-level under-5 mortality rate (figure 4). The expected ratio of under-5 mortality rate in the first quintile to fifth quintile (derived from the expected third quintile-disparity ratios) is around 1·58 (90% UI 1·47–1·70) for high national-level under-5 mortality rate (>200 deaths per 1000 livebirths). The ratio increases to its maximum at 2·04 (90% UI 1·79–2·33) as national-level under-5 mortality rate decreases to around 20 deaths per 1000 livebirths (figure 4).

The results for country-year, quintile-specific, under-5 mortality rate and corresponding absolute and percentage declines for all quintile groups for each of the 99 LMICs with empirical data are in the appendix (pp 14–25). Generally, under-5 mortality rate was the highest in the poorest quintile and the lowest in the richest quintile, but exceptions exist. Specifically, the under-5 mortality rate in the first quintile was greater than 90% of the average of under-5 mortality rate in the first and second quintiles in 1990 for all 99 countries with data except for Chad and Niger. The under-5 mortality rate in the fifth quintile was less than 110% of the average of under-5 mortality rate in the fourth and fifth quintiles for all countries in 1990 and 2016. The country disparity ranks of slope inequality index and concentration index in 2016 roughly agree with the ranks by use of the difference between and ratio of the first and fifth quintiles (appendix pp 55–56), suggesting that disparities between the poorest and the richest households are informative of the disparities across all quintiles.

**Figure 5:**
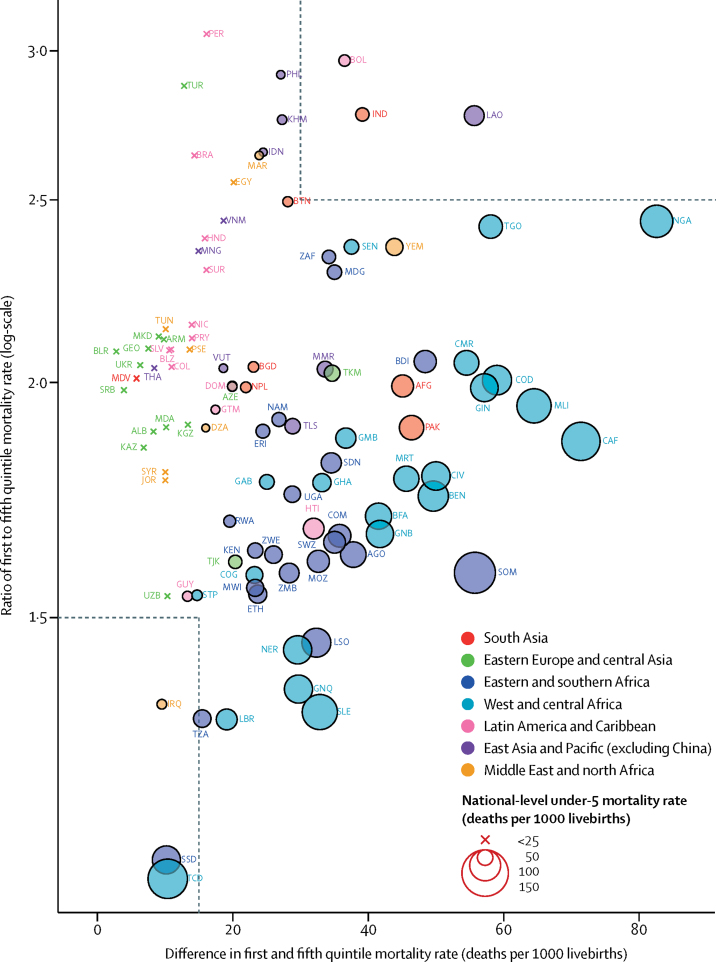
Ratio of first to fifth quintile mortality rate against difference in first and fifth quintile mortality rate in 99 low-income and middle-income countries with empirical data by national-level under-5 mortality rate in 2016 The size of the circle is proportional to the national-level of under-5 mortality rate (UN Inter-agency Group for Child Mortality Estimation 2017 estimates) in 2016.^1^ Circles are colour-coded according to the region each country belongs to. Country codes are International Organization for Standardization country codes. The box in dashed lines in the bottom left corner contains countries with a difference of less than 15 deaths per 1000 livebirths and a ratio of less than 1·5, and the box in the top right corner contains countries with a difference of more than 30 deaths per 1000 livebirths and a ratio of more than 2·5. AFG=Afghanistan. AGO=Angola. ALB=Albania. ARM=Armenia. AZE=Azerbaijan. BDI=Burundi. BEN=Benin. BFA=Burkina Faso. BGD=Bangladesh. BLR=Belarus. BLZ=Belize. BOL=Bolivia. BRA=Brazil. BTN=Bhutan. CAF=Central African Republic. CIV=Côte d'Ivoire. CMR=Cameron. COD=Democratic Republic of the Congo. COG=Congo. COL=Colombia. COM=Comoros. DOM=Dominican Republic. DZA=Algeria. EGY=Egypt. Eritrea=ERI. ETH=Ethiopia. GAB=Gabon. GEO=Georgia. GHA=Ghana. GIN=Guinea. GMB=Gambia. GNB=Guinea-Bissau. GNQ=Equitorial Guinea. GTM=Guatemala. GUY=Guyana. HND=Honduras. HTI=Haiti. IDN=Indonesia. IND=India. IRQ=Iraq. JOR=Jordan. KAZ=Kazakhstan. KEN=Kenya. KGZ=Kyrgyzstan. KHM=Cambodia. LAO=Laos. LBR=Liberia. LSO=Lesotho. MAR=Morocco. MDA=Moldova. MDG=Madagascar. MDV=Maldives. MKD=Macedonia. MLI=Mali. MMR=Myanmar. MNG=Mongolia. MOZ=Mozambique. MRT=Mauritania. MWI=Malawi. NAM=Namibia. NER=Niger. NGA=Nigeria. NIC=Nicaragua. NPL=Nepal. PAK=Pakistan. PER=Peru. PHL=Philippines. PRY=Paraguay. PSE=Palestine. RWA=Rwanda. SDN=Sudan. SEN=Senegal. SLE=Sierra Leone. SLV=El Salvador. SOM=Somalia. SRB=Serbia. SSD=South Sudan. STP=São Tomé and Príncipe. SUR=Suriname. SWZ=Swaziland. SYR=Syria. TCD=Chad. TGO=Togo. THA=Thailand. TJK=Tajikistan. TKM=Turkmenistan. TLS=Timor-Leste. TUN=Tunisia. TUR=Turkey. TZA=Tanzania. UGA=Uganda. UKR=Ukraine. UZB=Uzbekistan. VNM=Vietnam. VUT=Vanuatu. YEM=Yemen. ZAF=South Africa. ZMB=Zambia. ZWE=Zimbabwe.

The absolute and relative disparities in under-5 mortality rate between the poorest and richest quintiles varied substantially among the 99 LMICs with empirical data. In 2016, the difference between under-5 mortality rate in the first and fifth quintiles and the ratio of under-5 mortality rate in the first quintile to fifth quintile varied substantially across countries (figure 5). The differences between the under-5 mortality rate in the first and fifth quintiles ranged from 2·8 (90% UI 1·2–4·2) deaths per 1000 livebirths in Belarus to 82·6 (56·0–116·4) deaths per 1000 livebirths in Nigeria (appendix pp 38–40). The ratios of under-5 mortality rate in the first quintile to fifth quintile ranged from 1·09 (90% UI 0·91–1·32) in Chad to 3·06 (2·32–3·99) in Peru. In 2016, nine countries (Chad, Equatorial Guinea, Iraq, Lesotho, Liberia, Niger, Sierra Leone, South Sudan, and Zimbabwe) had ratios less than 1·5. Among these countries, Chad, Iraq, and South Sudan also had small absolute differences between under-5 mortality rates in the first and fifth quintiles (<15 deaths per 1000 livebirths). 11 countries (Bolivia, Brazil, Cambodia, Egypt, India, Indonesia, Laos, Morocco, Peru, Philippines, and Turkey) had ratios above 2·5 in 2016. Of these 11 countries, all except Cambodia and Laos had ratios above 2·5 in 1990 as well (appendix pp 38–40). Among the 11 countries with the highest ratios in 2016, Bolivia, India, and Laos also had the largest absolute disparities, with under-5 mortality rate differing by more than 30 deaths per 1000 livebirths between the first and fifth quintiles (figure 5).

## Discussion

Between the poorest and richest households from 1990 and 2016, the absolute gap in under-5 mortality rate has narrowed significantly and the difference in aggregated under-5 mortality rate halved for all LMICs (excluding China). The absolute declines in under-5 mortality rate in the poorest households in all regions were more than a third higher than those in the richest households. The relative difference between the poorest and richest under-5 mortality rates, however, remained similar between 1990 and 2016, with children in the poorest quintile being twice as likely to die before their 5th birthday compared with those in the richest quintile. Similarly, the disparity in under-5 mortality rate across all quintiles decreased significantly on the absolute scale but remained approximately constant on the relative scale during 1990 and 2016.

We provided estimates and UIs for quintile-specific under-5 mortality rate in 137 LMICs based on a statistical model. Our model results confirmed the empirical patterns from previous studies^12^, ^29^ that at high national-level under-5 mortality rate, the expected ratio of poor to rich under-5 mortality rate tends to be low. As the under-5 mortality rate at the national level decreases, the expected ratio tends to increase. The association confirms the inverse equity hypothesis^32^ that small disparities are expected at high mortality because most of the population, including the richest households, have inadequate access to basic health care and services. The initial decrease in the national-level under-5 mortality rate is likely to be driven by a decrease in under-5 mortality rate among the richest households, who selectively benefit from improved access to resources.^33^ Eventually, the poorer subpopulations catch up and when they do, experience faster reductions than the wealthiest subpopulations.

At the regional level, west and central Africa continued to have the highest quintile-specific under-5 mortality rate and one of the lowest ratios of under-5 mortality rate in the first quintile to under-5 mortality rate in the fifth quintile during 1990–2016. However, increasing relative disparities have been observed in the region since 1990, as indicated by a significant increase in the ratio of under-5 mortality rate in the poorest quintile to richest quintile. As the aggregated under-5 mortality rate for all quintiles combined in this region decreased from 198·7 (90% UI 192·7–205·2) deaths per 1000 livebirths in 1990 to 94·7 (83·4–110·3) deaths per 1000 livebirths in 2016,^1^ this ratio increased significantly. Because under-5 mortality rate is still high in many countries in this region, our model findings on the association between national-level under-5 mortality rate and the ratio of under-5 mortality rate in the poorest quintile to richest quintile suggest that relative disparities will possibly continue to increase after 2016, as national-level under-5 mortality rates further decrease. Policy interventions with an equity focus, which reach the most disadvantaged and vulnerable children, might help to change these trends. Efforts are needed to reduce high mortality across quintiles as well as address the increasing relative disparities in west and central Africa.

In south Asia, the large disparities in absolute and relative scales were mainly driven by results from India because its population size is the largest among all countries in the region. India's national-level under-5 mortality rate decreased from 125·8 (121·8–130·2) deaths per 1000 livebirths to 47·4 (38·8–47·3) deaths per 1000 livebirths between 1990 and 2016.^1^ In our study, India was identified as a high disparity country on absolute and relative scales. A further breakdown by smaller age groups can help to better understand the persisting high disparity in under-5 mortality rate in India. A previous study^34^ showed that in India during 1992–2006, relative disparities in mortality between the first and third years of life increased, whereas the inequality of mortality in the first year of life decreased.

Several major improvements and advantages in the data processing and modelling approach were used in this study. We calculated under-5 mortality rate by wealth quintile with an equal number of births in each quintile. This procedure has the benefit of providing a stable estimate of under-5 mortality rate for the richest quintile, since more births fall inside this quintile than when the standard method is used. The approach differs from the conventional way of deriving quintiles using data from Demographic and Health Surveys^23^, ^35^ and Multiple Indicator Cluster Surveys, in which the number of household members are the same in each quintile. The statistical model incorporates the association between national-level under-5 mortality rate and expected third quintile-disparity ratios. The model did reasonably well in validation exercises in which data were left out at random and at the end of the observation period (appendix pp 9–10). The results suggested that the model-based estimates were unbiased and UIs were conservative (containing the left-out observations more often than expected), hence suggesting that the approach worked well to construct estimates for country-years with missing information.

One of the main limitations of our study is due to the nature of the data used: we used household assets at the time of the survey as a proxy for household economic status. The household characteristics recorded in surveys only reflect the condition at the time of the interview, whereas the mortality data recorded a period before the survey was done. Additionally, although the set of assets and amenities were tailored in each survey to represent conditions in each country at a specific time, variation within each country might in some cases not be covered adequately. The principal component approach used to construct the wealth indices is also not guaranteed to accurately assign low scores to a country's poorest households. This limitation might explain our finding that mortality rate in the poorest quintile is lower than mortality rate in richer quintiles for a subset of country-years, hence reflecting problems in the index rather than reflecting lower mortality rate among the country's poorest populations. Finally, the fact that the wealth index is country specific implies that absolute country-period specific differences in economic status between the poorest and wealthiest quintiles vary.^36^ This limitation is not restricted to the analysis of disparities based on wealth indices—an income-based or consumption-based relative index would face similar problems because of different consumption patterns and prices within and between countries as well as over time. If interest lies in the estimation of cross-country differences in mortality rate associated with absolute differences in wealth, measures such as a proposed predicted absolute income measure based on households' asset rank, national consumption, and inequality levels^36^ can be used. However, any analysis based on absolute differences would not provide a standardised assessment of relative within-country disparities as done in this study.

The second main limitation in our study is data availability: we did not have data for 38 of 137 countries, and data at lower under-5 mortality rates and for more recent years are scarce. The absence of data for 38 countries results in model-driven estimates for those countries. The disparity pattern in these countries might differ from what the model suggested. For this reason, we did not present the country-specific estimates of under-5 mortality rate by wealth quintile for the 38 countries without any empirical data. We presented aggregated results based on all 137 countries, as opposed to results based on the 99 counties with data, to communicate our best estimates and related uncertainty on all LMICs (excluding China). The aggregated results are mainly driven by the 99 countries with available data as they accounted for 97% of all under-5 deaths in the 137 LMICs during 1990–2016. A comparison of the aggregated results based on the 99 countries with empirical data and the results based on the 137 LMICs is given in the appendix (pp 57–65). This comparison shows that the overall and regional ratios of quintile-specific to national-level under-5 mortality rate based on the 137 and 99 countries are approximately the same across quintiles over time. The aggregated quintile-specific under-5 mortality rates based on the 137 countries are slightly lower than those based on the 99 countries with empirical data, since countries without data tend to be countries with lower national-level under-5 mortality rates than those countries with data.

Because data are scarce on low levels of national under-5 mortality rate (<20 deaths per 1000 livebirths), estimates for the country-years corresponding to those levels were more uncertain and largely based on model extrapolation. Data for countries without information on disparities at low mortality are needed to assess the country-specific situations. Finally, most of the countries with data only have a small number of datapoints. Data are also limited in the most recent period; this study only contains 41 datapoints from 38 countries with reference year from 2010 onward. Extrapolations using past trends were used to derive trends in the most recent years. Efforts are needed to collect reliable, disaggregated, and timely data to better understand trends in mortality disparities.

Despite data limitations, validation exercises (appendix pp 9–10) suggest that our model-based estimates provide valuable information past the most recent datapoint. Point estimates are expected to be unbiased (we expect that the median difference between future observations and current estimates is equal to zero) and UIs are generally expected to be wide enough to convey the uncertainty of the estimates. In those countries where the model projections differ most from the truth (which will become clear with future data collection), we expect that future observations are likely to be less than the UIs. In those countries, observations will indicate less disparity than suggested by the model projections. This validation result suggests that use of the model-based projections presented in this study will not result in the undesirable situation whereby an underestimation of disparities results in lack of action to try to improve disparities.

In our study, we did not incorporate quintile-specific adjustments to reduce the bias associated with retrospective data in countries with high prevalence of HIV. Instead, we assumed that the observed ratios of quintile-specific to national-level under-5 mortality rates provide unbiased information of the true ratios. This assumption might result in the underestimation of the relative burden of HIV/AIDS-related deaths in children in the poorest quintiles. Additionally, we were not able to consider potential variation of reporting errors across quintiles because of the scarcity of information on the quintile-specific occurrence of such errors.

Our study provides a systematic assessment of under-5 mortality rate by wealth quintile for all LMICs (excluding China) and highlighted that the relative gap in child survival between the poorest and richest populations has remained constant during 1990 and 2016. Policy makers should not only acknowledge the progress made in child survival for the poorest subnational population across LMICs (excluding China), but also address the continued existence of within-country disparities and call for greater action to truly close the gap. Identification of patterns of inequity in under-5 mortality rate in countries is crucial for programming and planning.
