# Supplementary material for: National and regional under-5 mortality rate by economic status for low-income and middle-income countries: a systematic assessment
Source: Lancet Glob Health. 2018 Apr 10;6(5):e535–47. doi: 10.1016/S2214-109X(18)30059-7 (PMC5905403; doi:10.1016/S2214-109X(18)30059-7)
Supplement: Supplementary appendix [file mmc1.pdf]

THE LANCET
Global Health

Supplementary appendix

This appendix formed part of the original submission and has been peer reviewed. We post it as supplied by the authors.

Supplement to: Chao F, You D, Pedersen J, Hug L, Alkema L. National and regional under-5 mortality rate by economic status for low-income and middle-income countries: a systematic assessment. *Lancet Glob Health* 2018; **6:** e535–47.

Web appendix
National and regional under-5 mortality rate by economic status for low-income and middle-income countries: a systematic assessment

Fengqing Chao[*] Danzhen You[†] Jon Pedersen[‡] Lucia Hug[†] Leontine Alkema[§]

Contents

1 Abbreviations **1**

2 Data **1**

3 Method **3**
3.1 Wealth quintile-specific under-5 mortality rate model 3
3.2 Data model 5
3.3 Model summary 5
3.4 Computation of final results 6
3.4.1 Constructing posterior samples of outcomes that include crisis-related deaths and uncertainty in national-level under-5 mortality rate 7
3.4.2 Constructing uncertainty intervals and point estimates 7
3.4.3 Imputing results for countries without data 8
3.4.4 Computing aggregated results 8
3.4.5 Rounding 8
3.5 Model validation 9

4 Validation results **9**
4.1 Leaving out data based on survey year 9
4.2 Leaving out data randomly 9

5 Supplementary Tables **12**

6 Supplementary Figures **53**

[*]Institute of Policy Studies, Lee Kuan Yew School of Public Policy, National University of Singapore, Singapore 259599, Singapore. Email: chao.fengqing@nus.edu.sg
[†]Division of Data, Research, and Policy, United Nations Children's Fund, New York, NY, USA
[‡]Fafo, Oslo, Norway
[§]Department of Biostatistics and Epidemiology, School of Public Health and Health Sciences, University of Massachusetts, Amherst, MA, USA

List of Tables

1 Distribution of observations by source type for each wealth quintile 2
2 Data availability by region and reference period 2
3 Notation summary 6
4 Validation results for left-out observations when leaving out data from 2012 and later survey years. 10
5 Validation results for estimates when leaving out data from 2012 and later survey years. 10
6 Validation results for left-out observations when randomly leaving out 20% of all data. 10
7 Data availability for all the low-income and middle-income countries (excluding China) by region 13
8 Levels and trend in wealth quintile-specific under-5 mortality rate, by wealth quintile, for the 99 countries with empirical data 14
9 Number of wealth quintile-specific under-5 deaths, by wealth quintile, for all the low-income and middle-income countries (excluding China) combined, and by region and the 99 countries with empirical data 26
10 Estimates and uncertainty intervals for inequality indexs, for the 99 countries with empirical data . 38
11 Overview of data series by country 41

List of Figures

1 Sampling error distribution for full birth history and summary birth history data, by wealth quintile 2
2 3rd quintile-disparity ratios against national-level under-5 mortality rate – data trend 4
3 B-splines used in the regression model for the expected 3rd quintile-disparity ratios 4
4 3rd quintile-disparity ratios against national-level under-5 mortality rate – model results 54
5 Country ranks for inequality indexes in 2016, for the 99 countries with empirical data 55
6 Slope inequality index and concentration index in 2016, for the 99 countries with empirical data . 56
7 Comparison between aggregated results based on the 137 low-income and middle-income countries, and the aggregated results based on the 99 countries with empirical data. 57
8 Aggregated under-5 mortality rate and percentage of under-5 deaths by wealth quintile, by region. 66
9 Under-5 mortality rate by wealth quintile, for the 99 countries with empirical data. 74
10 Ratio of under-5 mortality rate in wealth quintile 1 (poorest) to wealth quintile 5 (richest), for the 99 countries with empirical data. 100
11 All wealth quintile-specific results, for the 99 countries with empirical data. 106

1 Abbreviations

DHS	Demographic and Health Survey
FBH	Full Birth History
LMICs	Low-income and middle-income countries
LOESS	Local Polynomial Regression
MCMC	Markov chain Monte Carlo
MICS	Multiple Indicator Cluster Survey
SBH	Summary Birth History
TSFB	Time since first birth
U5MR	Under-5 mortality rate
UN IGME	United Nations Inter-agency Group for Child Mortality Estimation
VR	Vital Registration

2 Data

Wealth quintiles Wealth quintiles as constructed in our study refer to five equal-sized birth groups with different levels of economic status (from the poorest to the richest) according to the wealth index assigned to each household. The wealth index is computed based on selected questions asked in the Demographic and Health Surveys (DHSs) and Multiple Indicator Cluster Surveys (MICSs). The questions ask about living conditions and household assets, and assign a score based on different answers. The wealth index is calculated based on the weighted sum of those scores in order to give an indirect or approximate measure for living and wealth standards for each household (1). The division into wealth quintiles in our study uses the product of the sampling weight and the number of births to include equal numbers of births in each wealth quintile.

Under-5 mortality rates by wealth quintile are derived from DHSs and MICSs. Under-5 mortality rates in these survey programs are collected in two forms: the Full Birth History (FBH), whereby women are asked for the date of birth of each of their children, whether the child is still alive, and if not the age at death; and the Summary Birth History (SBH), whereby women are asked only about the number of their children ever born and the number that have died (or equivalently the number still alive). In general, the sampling error of the wealth quintile-specific data points from FBH is smaller than the sampling error from SBH, and hence more informative and assigned more weight in the estimation process. Figure 1 shows the distribution of the the sampling errors for the ratio of wealth quintile-specific under-5 mortality rate to the national-level under-5 mortality rate by wealth quintile group.

FBH data, collected by all DHSs and increasingly so by MICSs, allow the calculation of child mortality indicators for specific time periods in the past. We calculated wealth quintile-specific under-5 mortality rate in the five-year period before the survey. The reference year of the data from FBH represents the mid-point in a five-year interval.

For SBHs, the time since first birth was used as an indicator of exposure time in the model to estimate mortality indicators. For wealth quintile-specific under-5 mortality rates from SBHs the time period of five to nine years since the first birth of mothers has been used, and is called "time since first birth (TSFB)" method. This method of constructing the under-5 mortality rate has been used by the United Nations Children's Fund since the 2014 round of estimation to replace the method which uses the age of the woman as an indicator of exposure time and exposure time period of the children to estimate mortality indicators. The main advantages of the new method (TSFB) over the previous method (using age of women) has been discussed in the Appendix of (2).

For wealth quintile-specific under-5 mortality rates, data are available for 99 low-income and middle-income countries (LMICs) with DHS and/or MICS surveys. Table 7 shows the data availability for all the LMICs (excluding China) by region and the World Bank income grouping. As of August 2017, the database contains 319 data series from DHS and MICS for LMICs. For each survey, we used one observation disaggregated by wealth quintile. In total, there are 1595 observations from LMICs, and the range of observed reference years (the mid-point of a five-year interval for FBH data) is 1987–2012. There are 41 surveys from 38 countries with reference year from 2010 onward. The percentage of the total number of under-5 deaths that were covered by the 38 counties with data since 2010 increased steadily from 32% in 1990 to 41% in 2016.

Table 1 summarizes the observations by source type for each wealth quintile group. Table 2 illustrates the percentage of countries with data for different reference periods among the 137 LMICs. Table 11 provides a full list of data series for the 99 countries.

Data source type	Number of data series
DHS Direct	224
MICS Direct	29
MICS Indirect	66
**total**	**319**

Table 1: **Distribution of observations by source type for each wealth quintile.** Observations are grouped by source type. "Direct" refers to observations obtained from full birth histories. "Indirect" refers to observations obtained from summary information and demographic methods. DHS: Demographic and Health Surveys; MICS: Multiple Indicator Cluster Surveys.

	1990–1994	1995–1999	2000–2004	2005–2009	2010–2016
137 low-income and middle-income countries	19.0	40.9	48.2	41.6	27.7
South Asia	25.0	37.5	50.0	62.5	37.5
Eastern Europe and Central Asia	14.3	33.3	57.1	19.0	9.5
Eastern and Southern Africa	25.0	62.5	58.3	62.5	45.8
West and Central Africa	25.0	75.0	79.2	66.7	45.8
Latin America and Caribbean	26.9	30.8	26.9	23.1	23.1
East Asia and Pacific (excluding China)	4.5	18.2	27.3	27.3	13.6
Middle East and North Africa	8.3	8.3	33.3	41.7	16.7

Table 2: **Data availability by region and reference period (in %).** Percentages of countries with data among the 137 LMICs or within each region by reference period. Note: the reference year for FBH data refers to the mid-point of a five-year interval.

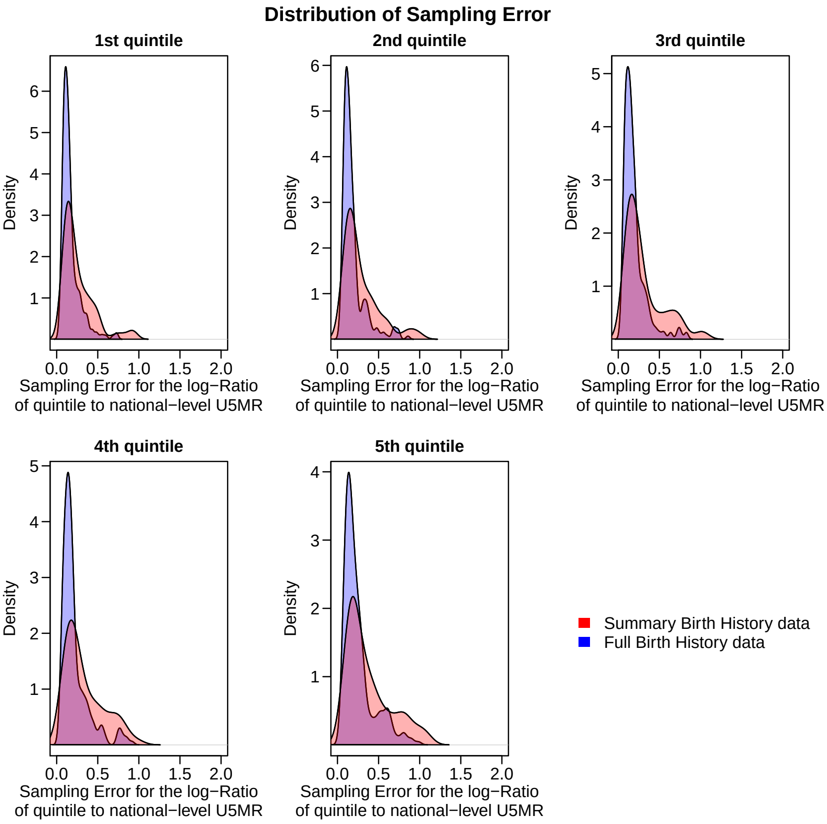

Figure 1: **Sampling error distribution for full birth history and summary birth history data, by wealth quintile.**

3 Method

Throughout the appendix, we use w as an index to denote the wealth quintile group $w = 1, \ldots, 5$. In the appendix, Q refers to the under-5 mortality rate (U5MR).

3.1 Wealth quintile-specific under-5 mortality rate model

Our goal is to estimate the wealth quintile-specific U5MR $Q_{w,c,t}$ for wealth quintile w, country c and year t. The wealth quintile-specific U5MRs are assumed to relate to the national-level U5MR as follows:

$$\begin{aligned}
Q(total)_{c,t} &= D(total)_{c,t}/B(total)_{c,t}, \\
Q_{w,c,t} &= D_{w,c,t}/(B(total)_{c,t}/5), \\
\frac{D(total)_{c,t}}{B(total)_{c,t}} &= \frac{\sum_{w=1}^{5} D_{w,c,t}}{(B(total)_{c,t}/5)\cdot 5}, \\
Q(total)_{c,t} &= \sum_{w=1}^{5} Q_{w,c,t}/5.
\end{aligned}$$

$Q(total)_{c,t}$ is the national-level U5MR, $D(total)_{c,t}$ is the total number of under-5 deaths, $B(total)_{c,t}$ is the total number of livebirths, and $D_{w,c,t}$ is the number of under-5 deaths from the w-th wealth quintile group. All notations are referring to country c in year t.

In order to incorporate the constraint that the wealth quintile-specific U5MRs sum up to five times the national-level U5MR, we estimated the 3rd quintile-disparity ratios $S_{w,c,t} = Q_{w,c,t}/Q_{3,c,t}$ for $w = 1, 2, 4, 5$. After estimating those ratios, the wealth quintile-specific U5MRs are recovered as functions of $S_{w,c,t}$'s:

$$\begin{aligned}
Q_{3,c,t} &= 5 \cdot Q(total)_{c,t}/(S_{1,c,t} + S_{2,c,t} + S_{4,c,t} + S_{5,c,t} + 1), \\
Q_{w,c,t} &= S_{w,c,t} \cdot Q_{3,c,t}, \text{ for } w = 1, 2, 4, 5.
\end{aligned}$$

We used the 3rd wealth quintile group (i.e. $w = 3$) as the reference group in the ratios because it is the group where we expected the proportion of deaths to be closest to 20%. National-level U5MR (excluding crisis-related deaths, i.e. crisis-free deaths) was used to predict the expected 3rd quintile-disparity ratios based on an expected (and empirically observed) relation between the ratios and national-level U5MR, using a flexible (penalized B-splines) regression model. Figure 2 shows the relationship between $S_{w,c,t}$ and the crisis-free national-level U5MR $Q(total)_{c,t}$ for $w = 1, 2, 4, 5$ in the four plots respectively. The national-level U5MR inputs $Q(total)_{c,t}$ used are the point estimates (excluding crisis-related under-5 deaths, and including HIV-related under-5 deaths) from the UN Inter-agency Group for Child Mortality Estimation (UN IGME) 2017 results (3). The LOESS (Local Polynomial Regression) curve in the 1st and 2nd plots increase as the national-level U5MR decreases, implying that as the national-level U5MR is decreasing, survival among children in the 3rd wealth quintile is improving more quickly as compared to survival among the poorest two groups. Similarly, the decreasing trend of the LOESS curve in the 4th plot indicate that as the national-level U5MR is declining over time, the decrease of the U5MR in the 4th richest wealth quintile is faster than that in the 3rd wealth quintile. For the richest wealth quintile, the LOESS curve suggests a survival advantage for children in the richest wealth quintile at any value of national-level U5MR, and the relative difference between the U5MR in the 5th and 3rd wealth quintile increases as the U5MR decreases until a national-level U5MR of about 50 deaths per 1000 livebirths, followed by a decrease of the relative difference.

The relation between national-level U5MR $Q(total)_{c,t}$ and $S_{w,c,t}$ is incorporated into the model for $S_{w,c,t}$:

$$S_{w,c,t} = U_{w,c,t} \cdot P_{w,c,t}, \text{ for } w = 1, 2, 4, 5,$$

where $U_{w,c,t}$ is the expected 3rd quintile-disparity ratio (as illustrated by the green LOESS curves in Figure 2) and $P_{w,c,t}$ is a quintile-country-year-specific multiplier. The specification of $U_{w,c,t}$ is explained in more detail below.

We used an AR(1) process to model the $P_{w,c,t}$'s on the log-scale:

$$\begin{aligned}
\log(P_{w,c,t}) &\sim N(0, (1-\rho_w^2)/\sigma_\epsilon^2), \text{ for } t = 1990, \\
\log(P_{w,c,t}) &\sim N(\rho_w \cdot \log(P_{w,c,t-1}), \sigma_\epsilon^2), \text{ for } t = 1991, \ldots, 2016.
\end{aligned}$$

ρ_w is wealth quintile-specific autoregressive parameter constrained to be between 0 and 1, allowing for a wealth quintile-specific rate of convergence back to zero on the log-scale. σ_ϵ^2 is distortion variance. ρ_w for $w = 1, 2, 4, 5$ and σ_ϵ are assigned with vague priors.

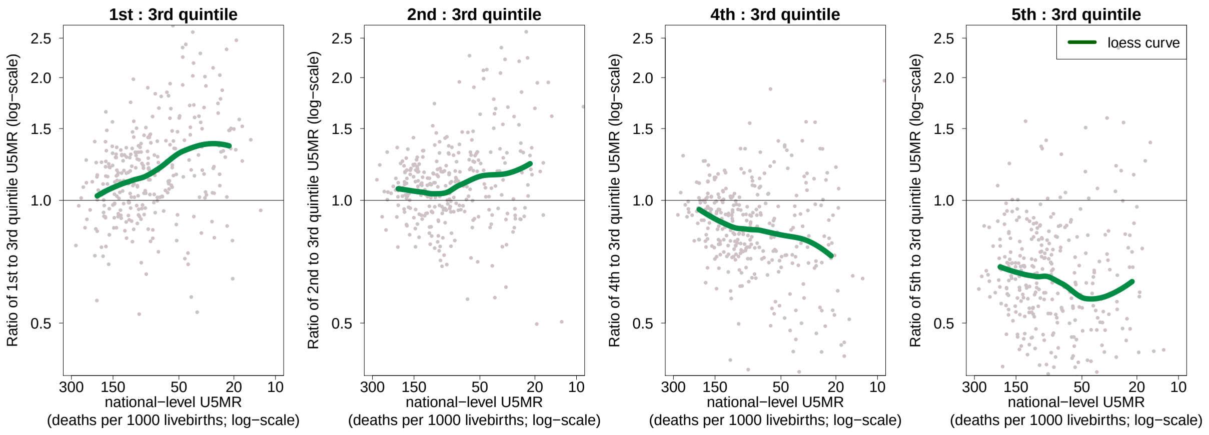

Figure 2: **3rd quintile-disparity ratios against national-level U5MR – data trend.** The grey dots are observed 3rd quintile-disparity ratios $S_{w,c,t}$ (i.e. $= Q_{w,c,t}/Q_{3,c,t}$) for $w = 1, 2, 4, 5$ respectively for the four plots. The green curves are LOESS curves between the 5th and 95th percentiles of the national-level U5MR.

Specification of the expected 3rd quintile-disparity ratio $U_{w,c,t}$ We used flexible penalized B-spline regression models (4; 5) to estimate the relation between national-level U5MR and the expected 3rd quintile-disparity ratios (based on data from the 95 LMICs), denoted by function $f_w(\cdot)$, for $w = 1, 2, 4, 5$. The function $f_w(q)$ for some national-level U5MR value q was specified as follows:

$$f_w(q) = \sum_{k=1}^{K} B_k(q)\alpha_{w,k}, \text{ for } w = 1, 2, 4, 5,$$

where $B_k(q)$ refers to the k-th B-spline evaluated at q and $\alpha_{w,k}$ to the k-th spline coefficient for group w. We set:

$$\log(U_{w,c,t}) = f_w(\widetilde{Q}(total)_{c,t}), \text{ for } w = 1, 2, 4, 5,$$

where $\widetilde{Q}(total)_{c,t}$ is the $Q(total)_{c,t}$ rounded to three decimal places (to reduce the number of splines evaluations).

The B-splines used in the regression models are illustrated in Figure 3. We used symmetric third-order polynomials, equally spaced on the log-transformed national-level U5MR scale (knots are set to be 0.3 apart). The resulting splines add up to unity at any level of national-level U5MR. To avoid extreme extrapolations, splines are combined for national-level U5MR less than 20 deaths per 1000 livebirths, and for national-level U5MR greater than the 95-th percentile of $Q(total)_{c,t}$.

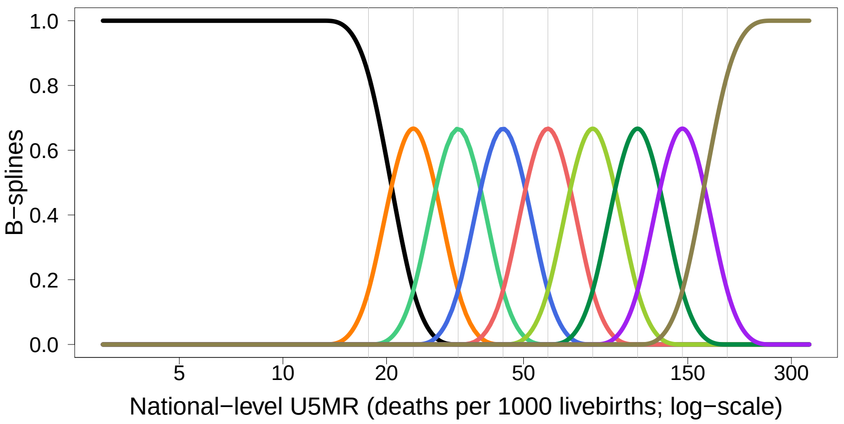

Figure 3: **B-splines used in the regression model for the expected 3rd quintile-disparity ratios.** B-splines plotted against the log-transformed national-level U5MR. The grey vertical lines indicate knots.

When fitting the splines model to observations, the first-order differences in adjacent splines coefficients were penalized to guarantee smoothness of the global relation between national-level mortality and expected ratios. The remainder of this subsection discusses the implementation details.

The splines regression model is specified as follows:

$$\begin{aligned} f_w(\tilde{\boldsymbol{q}}) &= \widetilde{\boldsymbol{B}}\boldsymbol{\alpha}_w, \\ \tilde{\boldsymbol{q}} &= (q_1, \ldots, q_j, \ldots, q_J)'. \end{aligned}$$

$\tilde{\boldsymbol{q}}$ represents the vector of unique values $\tilde{Q}(total)_{c,t}$ (rounded to three digits). Here, $J = 334$ since there are 334 unique values for $\tilde{Q}(total)_{c,t}$. $\widetilde{\boldsymbol{B}} = \boldsymbol{B}(\tilde{\boldsymbol{q}})$ the matrix of splines evaluated at each entry of $\tilde{\boldsymbol{q}}$, and $\boldsymbol{\alpha}_w$ the vector of splines coefficients with length equal to number of knots K. The splines equation can be written as follows (5; 6; 7):

$$\begin{aligned} \widetilde{\boldsymbol{B}}\boldsymbol{\alpha}_w &= \beta_w + \boldsymbol{Z} \times \boldsymbol{\delta}_w, \\ \boldsymbol{Z} &= \widetilde{\boldsymbol{B}}\boldsymbol{D}'(\boldsymbol{D}\boldsymbol{D}')^{-1}, \\ D_{i,j} &= \begin{cases} -1 & \text{if} \quad i = j, \\ 1 & \text{if} \quad i = j-1, \\ 0 & \text{o.w.} \end{cases} \end{aligned} \tag{1}$$

where the difference matrix $\boldsymbol{D}$ has dimension $H \times K$, with $H = K - 1$. The first part in Eq.(1), β_w describes the average constant level in the expected relative difference, and the second part $\boldsymbol{Z} \times \boldsymbol{\delta}_w$ describes the fluctuations around the linear trend. The dimension of matrix $\boldsymbol{Z}$ is $J \times H$. $\boldsymbol{\delta}_w = (\delta_{w,1}, \ldots, \delta_{w,H})'$. The first-order differences are penalized by imposing:

$$\delta_{w,h} \sim N(0, \sigma^2_{\delta_w}), \text{ for } w = 1, 2, 4, 5, \text{ and } h = 1, \ldots, H,$$

where variance $\sigma^2_{\delta_w}$ determines the extent of smoothing. Vague prior distributions are used for the splines model parameters.

3.2 Data model

Instead of using the observed wealth quintile-specific U5MR in the data model, we used the observed ratio of wealth quintile-specific U5MR to the national-level U5MR. By using the ratio, the survey-level bias on national-level is cancelled out (8). The survey-level biases on national-level are the differences between national-level U5MR as measured in the survey and the UN IGME estimate of the U5MR for the corresponding country-year. Let $r_{w,i} = q_{w,i}/q(total)_i$, the i-th observed ratio of the w-th wealth quintile-specific U5MR to the national-level U5MR, which is from country $c[i]$, in year $t[i]$. The data model is:

$$\log(r_{w,i}) \sim N(\log(R_{w,c[i],t[i]}), \gamma^2_{w,i}),$$

where $R_{w,c,t} = Q_{w,c,t}/Q(total)_{c,t}$. $\gamma^2_{w,i}$ is the sampling variance for the i-th observation. This variance term is computed from the micro data (following the methods described in (9)) and used as an input to the model.

3.3 Model summary

Notations Table 3 summarizes the notation and indexes used in Section 3.1 and Section 3.2.

Symbol	Description
t	Indicator for year, $t = 1990, \ldots, 2016$.
c	Indicator for country, $c = 1, \ldots, 99$.
w	Indicator for wealth quintile groups, $w = 1, \ldots, 5$. $w = 1$ refers to the 1st (i.e. the poorest) wealth quintile group, and $w = 5$ refers to the 5th (i.e. the richest) wealth quintile group.
i	Indicator for the i-th observation within a certain wealth quintile group.
$r_{w,i}$	The i-th observed ratio of the w-th wealth quintile-specific U5MR to the national-level U5MR.
$\gamma_{w,i}$	The i-th sampling error for $r_{w,i}$.
$q_{w,i}$	The i-th observed wealth quintile-specific U5MR from the w-th wealth quintile group.
$q(total)_i$	The i-th national-level U5MR from DHS and MICS surveys.
$Q(total)_{c,t}$	The crisis-free national-level U5MR for country c year t, is the point estimates (excluding crisis-related deaths) from the UN Inter-agency Group for Child Mortality Estimation (UN IGME) 2017 results (3).
$\widetilde{Q}(total)_{c,t}$	The $Q(total)_{c,t}$ rounded to three decimal places.
$Q_{w,c,t}$	The crisis-free wealth quintile-specific U5MR (excluding crisis-related deaths) for wealth quintile w, with $w = 1, \ldots, 5$, country c in year t.

Continued on next page

Table 3 – continued from previous page

Symbol	Description
$S_{w,c,t}$	The true 3rd quintile-disparity ratio (i.e. $= Q_{w,c,t}/Q_{3,c,t}$) of the U5MR from the w-th wealth quintile to the 3rd wealth quintile for $w = 1, 2, 4, 5$ respectively, for country c in year t.
$U_{w,c,t}$	The expected 3rd quintile-disparity ratio of the U5MR from the w-th wealth quintile to the 3rd wealth quintile for $w = 1, 2, 4, 5$ respectively, for country c in year t.
$P_{w,c,t}$	The relative difference between $S_{w,c,t}$ and $U_{w,c,t}$ for $w = 1, 2, 4, 5$, for country c in year t.
$R_{w,c,t}$	The ratio of the w-th crisis-free wealth quintile-specific U5MR to the crisis-free national-level U5MR for country c in year t, i.e. $= Q_{w,c,t}/Q(total)_{c,t}$.
ρ_w	Autoregressive parameter for AR(1) time series model for $\log(P_{w,c,t})$, for $w = 1, 2, 4, 5$.
σ^2_ϵ	Variance of distortion terms in AR(1) time series model for $\log(P_{w,c,t})$.

Table 3: **Notation summary.**

Wealth quintile-specific U5MR model

$$
\begin{aligned}
\log(r_{w,i}) &\sim N(\log(R_{w,c[i],t[i]}), \gamma^2_{w,i}), \text{ for } w = 1, \ldots, 5, \\
R_{w,c,t} &= Q_{w,c,t}/Q(total)_{c,t}, \text{ for } w = 1, \ldots, 5, \\
Q_{w,c,t} &= S_{w,c,t} \cdot Q_{3,c,t}, \text{ for } w = 1, 2, 4, 5, \\
Q_{3,c,t} &= 5 \cdot Q(total)_{c,t}/(S_{1,c,t} + S_{2,c,t} + S_{4,c,t} + S_{5,c,t} + 1), \\
S_{w,c,t} &= U_{w,c,t} \cdot P_{w,c,t}, \text{ for } w = 1, 2, 4, 5, \\
\log(U_{w,c,t}) &= f_w(\widetilde{Q}(total)_{c,t}), \text{ for } w = 1, 2, 4, 5, \\
f_w(\tilde{\boldsymbol{q}}) &= \beta_w + \boldsymbol{Z} \times \boldsymbol{\delta}_w, \text{ for } w = 1, 2, 4, 5, \\
\delta_{(w,h)} &\sim N(0, \sigma^2_{\delta_w}), \text{ for } w = 1, 2, 4, 5, \text{ and } h = 1, \ldots, H, \\
\log(P_{w,c,t}) &\sim N(0, (1 - \rho^2_w)/\sigma^2_\epsilon), \text{ for } w = 1, 2, 4, 5, \text{ for } t = 1990, \\
\log(P_{w,c,t}) &\sim N(\rho_w \cdot \log(P_{w,c,t-1}), \sigma^2_\epsilon), \text{ for } w = 1, 2, 4, 5, \text{ for } t = 1991, \ldots, 2016.
\end{aligned}
$$

Prior distributions Vague priors are assigned to hyper-parameters:

$$
\begin{aligned}
\beta_w &\sim U(-5, 5), \text{ for } w = 1, 2, 4, 5, \\
\rho_w &\sim U(0, 1), \text{ for } w = 1, 2, 4, 5, \\
\sigma_{\delta_w} &\sim U(0, 0.5), \text{ for } w = 1, 2, 4, 5, \\
\sigma_\epsilon &\sim U(0, 0.5).
\end{aligned}
$$

Computing We obtained posterior samples of all the model parameters and hyper parameters using a Markov chain Monte Carlo (MCMC) algorithm, implemented in the open source softwares `R 3.2.2` (10) and `JAGS 4.0.1` (Just another Gibbs Sampler) (11), using `R`-packages `coda` (12), `rjags` (13), and `R2jags` (14). Results were obtained from 12 chains with a total number of 742,000 iterations in each chain, while the first 512,000 iterations were discarded as burn-in, and thinning for every 50 iterations, the final posterior sample size for each parameter is 5,520. Convergence of the MCMC algorithm and the sufficiency of the number of samples obtained were checked through visual inspection of trace plots and convergence diagnostics of Gelman and Rubin (15).

All the other `R`-packages used in this project are: `classInt` (16), `doMC` (17), `foreign` (18), `foreach` (19), `ggplot2` (20), `MCMCpack` (21), `RColorBrewer` (22), `scales` (23), `soiltexture` (24), `truncnorm` (25), and `xtable` (26).

3.4 Computation of final results

Throughout the project, we presented our results in the format of "point estimate [lower bound; upper bound]". E.g. the wealth quintile-specific U5MR and under-5 deaths are presented as $Q^{P.E.}_{w,c,t}[Q^L_{w,c,t}; Q^U_{w,c,t}]$ and $D^{P.E.}_{w,c,t}[D^L_{w,c,t}; D^U_{w,c,t}]$ respectively. The rest of this section will explain how we derived each of the components in the result.

3.4.1 Constructing posterior samples of outcomes that include crisis-related deaths and uncertainty in national-level under-5 mortality rate

To include crisis-related deaths in the crisis-free quintile-specific estimates obtained from the model, we followed the procedures used by the UN IGME for adjusting national-level U5MR (2; 3), based on the assumption that the crisis events have the same effect across wealth quintiles. In total, 18 different crises from nine countries were accounted for in the estimation of wealth quintile-specific U5MR. The g-th posterior sample for wealth quintile-specific U5MR including crisis-related deaths, denoted as $Q(full)^{(g)}_{w,c,t}$, is defined as follows:

$$Q(full)^{(g)}_{w,c,t} = \begin{cases} Q(\text{crisis-free})^{(g)}_{w,c,t} + Q(\text{crisis})_{c,t}, & \text{if affected by crisis} \\ Q(\text{crisis-free})^{(g)}_{w,c,t} & \text{o.w.} \end{cases}$$

where

$$Q(\text{crisis-free})^{(g)}_{w,c,t} = R^{(g)}_{w,c,t} \cdot Q(total)^{(g)}_{c,t}.$$

$Q(\text{crisis-free})^{(g)}_{w,c,t}$ is the g-th posterior sample of crisis-free quintile-specific U5MR that includes uncertainty in national-level U5MR. $R^{(g)}_{w,c,t}$ is the g-th posterior sample of the ratio of the crisis-free wealth quintile-specific U5MR to the crisis-free national-level U5MR obtained from the model. $Q(total)^{(g)}_{c,t}$ is the g-th trajectory of crisis-free national-level U5MR obtained from the UN IGME 2017 results (3), and is not part of this modelling process.

$Q(\text{crisis})_{c,t}$ is the country-year-specific national-level U5MR due to crisis. No uncertainty of crisis-related U5MR is included in the final adjusted wealth quintile-specific U5MR results.

Posterior samples for the ratio of wealth quintile-specific U5MR (with crisis-related deaths) to the national-level U5MR (with crisis-related deaths), inclusive of uncertainty in the national-level under-5 death (including crisis-related deaths), were given by:

$$R(full)^{(g)}_{w,c,t} = \frac{Q(full)^{(g)}_{w,c,t}}{Q(full)^{(g)}_{c,t}},$$

where $Q(full)^{(g)}_{c,t}$ is the g-th trajectory of national-level U5MR (including crisis-related deaths). It is from the UN IGME 2017 results (3) and is not part of this modelling process.

Posterior samples for the number of under-5 deaths per wealth quintile (with crisis-related deaths) $D(full)^{(g)}_{w,c,t}$, inclusive of uncertainty in the national-level under-5 death (including crisis-related deaths), were obtained as follows:

$$D(full)^{(g)}_{w,c,t} = R(full)^{(g)}_{w,c,t} \cdot \frac{D(full)^{(g)}_{c,t}}{5}, \text{ for } w = 1, \ldots, 5,$$

where $D(full)^{(g)}_{c,t}$ is the g-th trajectory of number of national-level under-5 deaths (with crisis-related deaths). It is from the UN IGME 2017 results (3) and is not part of this modelling process.

3.4.2 Constructing uncertainty intervals and point estimates

The 90% uncertainty intervals for $R(full)_{w,c,t}$, $Q(full)_{w,c,t}$, and $D(full)_{w,c,t}$, denoted as $[*^L; *^U]$, are the 5th and 95th percentiles of the corresponding posterior samples:

$$*^L = \text{percentile}_{5\%} \left\{ *^{(1)}, \ldots, *^{(G)} \right\},$$
$$*^U = \text{percentile}_{95\%} \left\{ *^{(1)}, \ldots, *^{(G)} \right\}.$$

where $*$ can be substituted with $R(full)_{w,c,t}$, $Q(full)_{w,c,t}$, and $D(full)_{w,c,t}$.

We constructed the point estimates of $R(full)_{w,c,t}$ (denoted as $R(full)^{P.E.}_{w,c,t}$) by re-scaling the medians of $R(full)_{w,c,t}$ (denoted as $R(full)^{M}_{w,c,t}$), such that $R(full)^{P.E.}_{w,c,t}$ across wealth quintiles sum up to 5 for $\forall c, \forall t$:

$$R(full)^{M}_{w,c,t} = \text{median} \left\{ R(full)^{(1)}_{w,c,t}, \ldots, R(full)^{(G)}_{w,c,t} \right\}, \text{ for } w = 1, \ldots, 5,$$

where $R(full)^{(g)}_{w,c,t}$ is the g-th posterior sample of $R(full)_{w,c,t}$ as explained in above Section 3.4.1. Then we re-scaled each $R(full)^{M}_{w,c,t}$ to $R(full)^{P.E.}_{w,c,t}$ for $w = 1, \ldots, 5$:

$$R(full)^{P.E.}_{w,c,t} = 5 \cdot \frac{R(full)^{M}_{w,c,t}}{\sum_{w=1}^{5} R(full)^{M}_{w,c,t}}, \text{ for } \forall c, \forall t.$$

The point estimates $R(full)^{P.E.}_{w,c,t}$ are combined with the point estimates of national-level U5MR $Q(full)^{P.E.}_{c,t}$ and number of national-level under-5 deaths $D(full)^{P.E.}_{c,t}$ (both include crisis-related deaths and were obtained from the UN IGME 2017 results (3), not part of the modelling process) to derive the point estimates of quintile-specific U5MR $Q(full)^{P.E.}_{w,c,t}$ and point estimates of under-5 deaths $D(full)^{P.E.}_{w,c,t}$:

$$\begin{aligned} Q(full)^{P.E.}_{w,c,t} &= R(full)^{P.E.}_{w,c,t} \cdot Q(full)^{P.E.}_{c,t}, \\ D(full)^{P.E.}_{w,c,t} &= R(full)^{P.E.}_{w,c,t} \cdot \frac{D(full)^{P.E.}_{c,t}}{5}. \end{aligned}$$

3.4.3 Imputing results for countries without data

38 out of the 137 LMICs do not have wealth quintile-specific data. For these countries, the results were imputed as follows. Firstly, we imputed $\log(P_{w,c,t})$ for any country without data based on the posterior samples of parameters related to the multiplier. Let $P^{(g)}_{w,c,t}$ be the g-th imputed sample for country c without data in time t, and $\sigma^{(g)}_{\epsilon}$, $\rho^{(g)}$ be the g-th posterior sample from the model:

$$\begin{aligned} \log(P^{(g)}_{w,c,t}) &\sim N\left(0, \frac{(\sigma^{(g)}_{\epsilon})^2}{1-(\rho^{(g)})^2}\right), \text{ for } t = 1990, \\ \log(P^{(g)}_{w,c,t}) &= \rho^{(g)}_{w} \cdot \log(P^{(g)}_{w,c,t-1}) + \epsilon^{(g)}_{c,t}, \text{ for } w = 1,2,4,5, \text{ for } t = 1991, \ldots, 2016, \\ \text{where} & \\ \epsilon^{(g)}_{c,t} &\sim N(0, (\sigma^{(g)}_{\epsilon})^2), \text{ for } t = 1991, \ldots, 2016. \end{aligned}$$

Secondly, for a given country-year, the crisis-free national-level U5MR (rounded to three digits) was used to identify the posterior samples of $\log(U_{w,c,t})$

$$\log(U^{(1,\ldots,G)}_{w,c,t}) = f_w(\widetilde{Q}(total)_{c,t}), \text{ for } w = 1,2,4,5.$$

Hence, we computed the imputed $S^{(g)}_{w,c,t} = P^{(g)}_{w,c,t} \cdot U^{(g)}_{w,c,t}$. We then followed the same steps as described in Section 3.4.1 and Section 3.4.2 to calculate the uncertainty intervals and point estimates of wealth quintile-specific U5MR and under-5 deaths.

3.4.4 Computing aggregated results

The 137 countries were LMICs based on the World Bank country income classification* in 2016. We did not include China. The 137 LMICs are listed in Table 7 by region and data availability. The wealth quintile-specific under-5 deaths from region r for the w-th wealth quintile group for year t, denoted by $DR_{w,r,t}$, was computed as:

$$DR_{w,r,t} = \sum_{c \in \{region[c]=r\}} D(full)_{w,c,t}, \text{ for } w = 1, \ldots, 5.$$

The wealth quintile-specific U5MR from region r for the w-th wealth quintile group for year t, denoted by $QR_{w,r,t}$, was computed as:

$$\begin{aligned} QR_{w,r,t} &= QR(full)_{r,t} \cdot \frac{DR_{w,r,t} \cdot 5}{DR(full)_{r,t}}, \text{ for } w = 1, \ldots, 5, \\ \text{where} & \\ DR(full)_{r,t} &= \sum_{c \in \{region[c]=r\}} D(full)_{c,t}. \end{aligned}$$

$QR(full)_{r,t}$ is the aggregated U5MR from region r in year t from the UN IGME 2017 results (3).

3.4.5 Rounding

We kept three significant figures for all reported estimates and uncertainty intervals, including those for the number of wealth quintile-specific under-5 deaths.

*The World Bank country classification based on income can be downloaded at: https://datahelpdesk.worldbank.org/knowledgebase/articles/906519-world-bank-country-and-lending-groups

3.5 Model validation

Model performance was assessed through out-of-sample validation. In the first exercise, we left out all observations that were obtained after a certain survey year leaving out around 20% observations (27). Based on the current database, all data that were collected in the year 2012 and onward were left out. We fitted the model to the training data set, and obtained point estimates and uncertainty intervals that would have been constructed based on the available data set in the year 2011. We also assessed the model performance using the traditional approach of leaving out data at random, i.e. leaving out 20% of the data randomly, and repeated this exercise 30 times.

We calculated median errors and median absolute errors for the left-out observations, where errors are defined as $e_{a,i} = r_{w,i} - \widehat{r}_{w,i}$, with $\widehat{r}_{w,i}$ the posterior median of the predictive distribution based on training data set for the left-out observation $r_{w,i}$. Coverage is given by $1/n \cdot \sum 1[r_{w,i} \geq l_{w,i}] \cdot 1[r_{w,i} \leq u_{w,i}]$, where n refers to the number of left-out observations, and $l_{w,i}$ and $u_{w,i}$ correspond to the lower and upper bounds of the respective prediction interval for the left-out observation $r_{w,i}$. The validation measures were calculated for 100,000 sets of left-out observations, where each set consisted of only one randomly selected left-out observation from each country. The reported validation results were based on the mean of the outcomes from the 100,000 sets of left-out observations for the validation exercise based on leaving out recent data, and the mean of the 100,000 times 30 training-set specific outcomes for the exercise with randomly left-out data.

For the validation based on leaving out recent data only, point estimates based on the full data set were compared to point estimates and uncertainty intervals obtained from the training data set. For this calculation, errors are defined as $e_{w,c,t} = R_{w,c,t} - R^{(train)}_{w,c,t}$, where $R_{w,c,t}$ is the posterior median for country c in year t for the w-th wealth quintile based on the full data set, and $R^{(train)}_{w,c,t}$ is the posterior median for the same country-year and wealth quintile based on the training data set. Coverage was computed in a similar manner as for the left-out observations, based on the lower and upper bounds of the 95% uncertainty interval of $R^{(train)}_{w,c,t}$ from the training data set.

4 Validation results

4.1 Leaving out data based on survey year

We left out all observations collected since the year 2012: 310 observations were left out, corresponding to 19.4% of all observations. Table 4 summarizes the results related to the left-out observations for the validation exercise based on 90% and 80% prediction intervals (PIs). Median errors were very close to zero for left-out observations in all the wealth quintile groups. Coverage of 90% PIs were higher than expected: 98.2%, 98.2%, 98.2%, 94.6%, 94.6% for the 5 wealth quintiles respectively. Coverage of 80% PIs were higher than expected at 94.6%, 96.4%, 95.5%, 83.9%, and 87.5%. Hence, PIs are conservative, meaning that they contain the truth more often than expected, with only one exception: observations of q_1/q_5 fall below their respective uncertainty interval slightly more often than expected (7.1% of the left out observations fall below their respective 90% PI, as compared to the expected 5%, and 14.3% fall below their respective 80% PI, as compared to the expected 10%). This implies that the lower bound of the uncertainty intervals based on model-based extrapolations after the most recent data point may be too high for the ratio q_1/q_5.

Table 5 shows the results for the comparison between estimates obtained from the full data set, and estimates based on the training set. Median errors were close to zero. The proportion of updated estimates that fell outside the uncertainty intervals constructed based on the training set was well below 5%, as desired.

Given that the median errors are close to zero in both Table 4 and Table 5, the point forecasts for quintile-specific U5MR and the ratio q_1/q_5 are unbiased.

4.2 Leaving out data randomly

Table 6 shows the results of the validation exercises whereby data were left out at random. Median errors are close to zero for all the wealth quintile groups. The proportions of left-out data falling outside the 90% and 80% PIs are lower than expected for all the wealth quintile groups. This means that the PIs of the model are more conservative than expected. No systematic biases are observed for PIs.

	r_1	r_2	r_3	r_4	r_5	q_1/q_5
Median of Error	-0.01	-0.03	-0.01	0.00	0.01	-0.00
Median of absolute Error	0.13	0.09	0.09	0.11	0.10	0.44
Left-out observations fall below 90% PI (%)	1.8	1.8	1.8	0.0	1.8	7.1
Left-out observations fall above 90% PI (%)	0.0	0.0	0.0	5.4	3.6	3.6
**Expected proportions (%)**	**5**	**5**	**5**	**5**	**5**	**5**
Left-out observations fall below 80% PI (%)	5.4	1.8	3.6	5.4	7.1	14.3
Left-out observations fall above 80% PI (%)	0.0	1.8	0.9	10.7	5.4	7.1
**Expected proportions (%)**	**10**	**10**	**10**	**10**	**10**	**10**

Table 4: **Validation results for left-out observations when leaving out data from 2012 and later survey years.** Errors are defined as the difference between a left-out observation and the posterior median of its predictive distribution obtained from the training set.

	R_1			R_2			R_3			R_4			R_5			Q_1/Q_5		
**Year**	**2005**	**2010**	**2013**	**2005**	**2010**	**2013**	**2005**	**2010**	**2013**	**2005**	**2010**	**2013**	**2005**	**2010**	**2013**	**2005**	**2010**	**2013**
Median of Error	0.00	0.00	0.00	-0.00	0.00	0.00	-0.00	-0.01	-0.01	0.00	0.00	0.00	-0.00	-0.00	-0.00	0.00	0.00	0.01
Median of absolute Error	0.02	0.03	0.03	0.01	0.01	0.01	0.00	0.01	0.01	0.01	0.02	0.02	0.01	0.01	0.01	0.05	0.06	0.07
Below 90% CI of validation run (%)	0.0	1.0	1.0	0.0	0.0	0.0	0.0	0.0	0.0	0.0	0.0	0.0	1.0	1.0	1.0	0.0	0.0	0.0
Above 90% CI of validation run (%)	0.0	0.0	0.0	0.0	0.0	0.0	0.0	0.0	0.0	0.0	1.0	0.0	0.0	0.0	0.0	1.0	0.0	0.0
**Expected proportions (%)**	**≤5**	**≤5**	**≤5**	**≤5**	**≤5**	**≤5**	**≤5**	**≤5**	**≤5**	**≤5**	**≤5**	**≤5**	**≤5**	**≤5**	**≤5**	**≤5**	**≤5**	**≤5**

10

Table 5: **Validation results for estimates when leaving out data from 2012 and later survey years.** Errors are defined as the differences between estimates based on the full dataset and the training set. The proportions refer to the proportions (%) of countries in which the median ratio estimates based on the full data set fall below or above their corresponding 90% uncertainty intervals based on the training dataset. The results are broken down by wealth quintile groups and year.

	r_1	r_2	r_3	r_4	r_5	q_1/q_5
Median of Error	0.00	-0.01	-0.01	0.00	-0.01	0.05
Median of absolute Error	0.11	0.09	0.08	0.09	0.10	0.34
Left-out observations fall below 90% PI (%)	3.3	1.3	1.4	2.6	2.3	4.0
Left-out observations fall above 90% PI (%)	1.7	1.0	2.5	1.9	3.4	4.0
**Expected proportions (%)**	**5**	**5**	**5**	**5**	**5**	**5**
Left-out observations fall below 80% PI (%)	5.9	5.1	3.8	8.2	6.5	10.1
Left-out observations fall above 80% PI (%)	4.0	3.7	5.5	4.8	5.6	8.2
**Expected proportions (%)**	**10**	**10**	**10**	**10**	**10**	**10**

Table 6: **Validation results for left-out observations when randomly leaving out 20% of all data.** Errors are defined as the difference between a left-out observation and the posterior median of its predictive distribution.

References

[1] Shea Oscar Rutstein KJ. DHS Comparative Reports No 6: The DHS Wealth Index. ORC Macro; 2004.

[2] You D, Hug L, Ejdemyr S, Idele P, Hogan D, Mathers C, et al. Global, regional, and national levels and trends in under-5 mortality between 1990 and 2015, with scenario-based projections to 2030: a systematic analysis by the UN Inter-agency Group for Child Mortality Estimation. The Lancet. 2015;386(10010):2275–2286.

[3] UNICEF. UN Inter-agency group for Child Mortality Estimation. Levels & Trends in Child Mortality. Report 2017; 2017. Available from: http://www.childmortality.org/files_v21/download/IGME%20report%202017%20child%20mortality%20final.pdf.

[4] Eilers PHC, Marx BD. Flexible Smoothing with B-splines and Penalties. Statistical Science. 1996;11(2):89–121.

[5] Eilers PHC, Marx BD. Splines, knots, and penalties. Wiley Interdisciplinary Reviews: Computational Statistics. 2010 Nov;2(6):637–653. Available from: http://doi.wiley.com/10.1002/wics.125.

[6] Currie ID, Durban M. Flexible smoothing with P-splines: a unified approach. Statistical Modelling. 2002 Dec;2(4):333–349. Available from: http://smj.sagepub.com/content/2/4/333.

[7] Eilers PHC. Discussion of: Verbyla, A. P., B. R. Cullis, M. G. Kenward, and S. J. Welham. "The Analysis of Designed Experiments and Longitudinal Data Using Smoothing Splines.". Journal of the Royal Statistical Society. 1999;Series C(48):300–311.

[8] Alkema L, New JR, et al. Global estimation of child mortality using a Bayesian B-spline bias-reduction model. The Annals of Applied Statistics. 2014;8(4):2122–2149.

[9] Pedersen J, Liu J. Child mortality estimation: appropriate time periods for child mortality estimates from full birth histories. PLoS medicine. 2012;9(8):e1001289.

[10] R Core Team. R: A Language and Environment for Statistical Computing. Vienna, Austria; 2015. Available from: https://www.R-project.org/.

[11] Plummer M. JAGS: A Program for Analysis of Bayesian Graphical Models Using Gibbs Sampling. In: Proceedings of the 3rd International Workshop on Distributed Statistical Computing (DSC 2003), March 20-22, Vienna, Austria. ISSN 1609-395X. http://mcmc-jags.sourceforge.net/; 2003. .

[12] Plummer M, Best N, Cowles K, Vines K. CODA: Convergence Diagnosis and Output Analysis for MCMC. R News. 2006;6(1):7–11. Available from: http://CRAN.R-project.org/doc/Rnews/.

[13] Plummer M. rjags: Bayesian graphical models using MCMC; 2011. R package version 3-5. Available from: http://CRAN.R-project.org/package=rjags.

[14] Su YS, Yajima M. R2jags: A Package for Running jags from R; 2011. R package version 0.02-17. Available from: http://CRAN.R-project.org/package=R2jags.

[15] Gelman A, Rubin D. Inference from iterative simulation using multiple sequences. Statistical Science. 1992;7:457–511.

[16] Bivand R. classInt: Choose Univariate Class Intervals; 2015. R package version 0.1-23. Available from: https://CRAN.R-project.org/package=classInt.

[17] Analytics R, Weston S. doMC: Foreach Parallel Adaptor for 'parallel'; 2015. R package version 1.3.4. Available from: http://CRAN.R-project.org/package=doMC.

[18] R Core Team. foreign: Read Data Stored by Minitab, S, SAS, SPSS, Stata, Systat, Weka, dBase, ...; 2016. R package version 0.8-67. Available from: http://CRAN.R-project.org/package=foreign.

[19] Analytics R, Weston S. foreach: Provides Foreach Looping Construct for R; 2015. R package version 1.4.3. Available from: http://CRAN.R-project.org/package=foreach.

[20] Wickham H. ggplot2: Elegant Graphics for Data Analysis. Springer-Verlag New York; 2009. Available from: http://ggplot2.org.

[21] Martin AD, Quinn KM, Park JH. MCMCpack: Markov Chain Monte Carlo in R. Journal of Statistical Software. 2011;42(9):22. Available from: http://www.jstatsoft.org/v42/i09/.

[22] Neuwirth E. RColorBrewer: ColorBrewer Palettes; 2014. R package version 1.1-2. Available from: https://CRAN.R-project.org/package=RColorBrewer.

[23] Wickham H. scales: Scale Functions for Visualization; 2016. R package version 0.4.0. Available from: https://CRAN.R-project.org/package=scales.

[24] Moeys J. soiltexture: Functions for Soil Texture Plot, Classification and Transformation; 2016. R package version 1.4.1. Available from: https://CRAN.R-project.org/package=soiltexture.

[25] Trautmann H, Steuer D, Mersmann O, Bornkamp B. truncnorm: Truncated normal distribution; 2014. R package version 1.0-7. Available from: http://CRAN.R-project.org/package=truncnorm.

[26] Dahl DB. xtable: Export Tables to LaTeX or HTML; 2016. R package version 1.8-2. Available from: http://CRAN.R-project.org/package=xtable.

[27] Alkema L, Wong MB, Seah PR. Monitoring progress towards Millennium Development Goal 4: a call for improved validation of under-5 mortality rate estimates. Statistics, Politics and Policy. 2012 Jun;3(2). Available from: http://www.degruyter.com/view/j/spp.2012.3.issue-2/2151-7509.1043/2151-7509.1043.xml.

5 Supplementary Tables

- Table 7: Data availability for all the LMICs (excluding China) by region;
- Table 8: Levels of and trends in wealth quintile-specific under-5 mortality rate, by wealth quintile, for the 99 countries with empirical data;
- Table 9: Number of wealth quintile-specific under-5 deaths, by wealth quintile, for all the LMICs (excluding China) combined, by region and the 99 countries with empirical data;
- Table 10: Estimates and uncertainty intervals for inequality indexs, for the 99 countries with empirical data;
- Table 11: Overview of data series by country.

Table 7: **Data availability for all the low-income and middle-income countries (excluding China) by region.** The 137 low-income and middle-income countries are used to generate aggregated results. For counties with data, model estimates are used. For counties without data, we imputed results. Countries are categorized by region. The red numbers in brackets represent the numbers of countries in each group.

**Region**	**[99] Country with data (use model estimates)**		**[38] Country without data (use model-based estimates)**	
	**[30] Low income**	**[69] Middle income**	**[1] Low income**	**[37] Middle income**
South Asia	[2] Afghanistan; Nepal	[5] Bangladesh; Bhutan; India; Maldives; Pakistan	[0]	[1] Sri Lanka
Eastern Europe and Central Asia	[0]	[15] Albania; Armenia; Azerbaijan; Belarus; Georgia; Kazakhstan; Kyrgyzstan; Republic of Moldova; The former Yugoslav Republic of Macedonia; Serbia; Tajikistan; Turkmenistan; Turkey; Ukraine; Uzbekistan	[0]	[6] Bulgaria; Bosnia and Herzegovina; Croatia; Montenegro; Romania; Russian Federation
Eastern and Southern Africa	[13] Burundi; Comoros; Eritrea; Ethiopia; Madagascar; Mozambique; Malawi; Rwanda; Somalia; South Sudan; United Republic of Tanzania; Uganda; Zimbabwe	[8] Angola; Kenya; Lesotho; Namibia; Sudan; Swaziland; South Africa; Zambia	[0]	[3] Botswana; Djibouti; Mauritius
West and Central Africa	[14] Benin; Burkina Faso; Central African Republic; Democratic Republic of the Congo; Guinea; Gambia; Guinea-Bissau; Liberia; Mali; Niger; Senegal; Sierra Leone; Chad; Togo	[9] Cote d'Ivoire; Cameroon; Congo; Gabon; Ghana; Equatorial Guinea; Mauritania; Nigeria; Sao Tome and Principe	[0]	[1] Cabo Verde
Latin America and Caribbean	[1] Haiti	[13] Belize; Bolivia (Plurinational State of); Brazil; Colombia; Dominican Republic; Guatemala; Guyana; Honduras; Nicaragua; Peru; Paraguay; El Salvador; Suriname	[0]	[12] Argentina; Costa Rica; Cuba; Dominica; Ecuador; Grenada; Jamaica; Saint Lucia; Mexico; Panama; Saint Vincent and the Grenadines; Venezuela (Bolivarian Republic of)
East Asia and Pacific (excluding China)	[0]	[10] Indonesia; Cambodia; Lao People's Democratic Republic; Myanmar; Mongolia; Philippines; Thailand; Timor-Leste; Viet Nam; Vanuatu	[1] Democratic People's Republic of Korea	[11] Fiji; Micronesia (Federated States of); Kiribati; Marshall Islands; Malaysia; Nauru; Papua New Guinea; Solomon Islands; Tonga; Tuvalu; Samoa
Middle East and North Africa	[0]	[9] Algeria; Egypt; Iraq; Jordan; Morocco; State of Palestine; Syrian Arab Republic; Tunisia; Yemen	[0]	[3] Iran (Islamic Republic of); Lebanon; Libya

Table 8: **Levels and trend in wealth quintile-specific under-5 mortality rate, by wealth quintile, for the 99 countries with empirical data.** Estimates and 90% uncertainty intervals for (i) wealth quintile-specific U5MR in 1990, 2000, and 2016, (ii) the absolute and percentage declines in the wealth quintile-specific U5MR from 1990 to 2016; (ii) ratio of wealth quintile-specific U5MR to the national-level U5MR per 1,000 in 1990, 2000, and 2016, and the change in the ratio from 1990 to 2016, by wealth quintile, for the 99 countries with empirical data. Numbers in brackets are 90% uncertainty intervals. Countries are ordered alphabetically.

		wealth quintile-specific U5MR (deaths per 1000 livebirths)					Ratio of wealth quintile-specific to national-level U5MR			
	Wealth Quintile	1990	2000	2016	Absolute decline 1990–2016	Percentage decline 1990–2016	1990	2000	2016	Change 1990–2016
Afghanistan	1st	201.9 (171.2 – 237.2)	156.0 (136.2 – 178.2)	90.5 (71.2 – 112.3)	111.3 (75.4 – 150.4)§	55.1% (42.0 – 66.0)§	1.14 (0.99 – 1.30)	1.20 (1.07 – 1.34)‡	1.29 (1.16 – 1.42)‡	0.15 (-0.03 – 0.32)
	2nd	200.7 (176.1 – 228.2)	146.3 (129.9 – 164.0)	80.8 (64.0 – 99.5)	119.9 (89.6 – 152.0)§	59.7% (48.9 – 69.0)§	1.13 (1.02 – 1.24)‡	1.13 (1.03 – 1.22)‡	1.15 (1.05 – 1.25)‡	0.02 (-0.12 – 0.15)
	3rd	188.6 (168.8 – 210.1)	137.6 (124.8 – 151.5)	73.1 (58.5 – 89.0)	115.5 (90.9 – 140.7)§	61.3% (51.6 – 69.7)§	1.06 (1.00 – 1.13)	1.06 (1.00 – 1.12)‡	1.04 (0.98 – 1.10)	-0.03 (-0.10 – 0.05)
	4th	178.0 (152.9 – 205.7)	123.4 (108.5 – 140.0)	62.1 (48.7 – 76.8)	115.9 (87.3 – 145.8)§	65.1% (55.1 – 73.4)§	1.00 (0.89 – 1.12)	0.95 (0.86 – 1.05)	0.88 (0.79 – 0.97)‡	-0.12 (-0.25 – 0.01)
	5th	117.3 (96.5 – 142.0)	85.2 (72.6 – 99.5)	45.5 (35.3 – 57.6)	71.9 (49.5 – 97.0)§	61.2% (48.8 – 71.1)§	0.66 (0.56 – 0.78)‡	0.66 (0.57 – 0.75)‡	0.65 (0.56 – 0.74)‡	-0.02 (-0.14 – 0.10)
Albania	1st	49.5 (39.5 – 60.1)	32.6 (25.6 – 40.6)	17.7 (9.3 – 33.2)	31.8 (14.1 – 43.9)§	64.3% (31.1 – 81.2)§	1.24 (1.03 – 1.45)‡	1.25 (1.04 – 1.47)‡	1.31 (1.08 – 1.56)‡	0.07 (-0.15 – 0.30)
	2nd	47.5 (39.9 – 55.5)	30.6 (25.2 – 37.1)	15.8 (8.3 – 29.4)	31.7 (16.2 – 42.1)§	66.8% (36.7 – 82.4)§	1.19 (1.05 – 1.33)‡	1.18 (1.04 – 1.31)‡	1.17 (1.01 – 1.33)‡	-0.02 (-0.19 – 0.15)
	3rd	40.8 (34.9 – 46.9)	26.5 (22.1 – 31.5)	13.7 (7.4 – 25.2)	27.2 (14.3 – 35.3)§	66.5% (36.8 – 82.0)§	1.02 (0.93 – 1.11)	1.02 (0.93 – 1.10)	1.01 (0.91 – 1.11)	-0.01 (-0.10 – 0.08)
	4th	34.5 (28.5 – 41.2)	22.2 (17.9 – 27.5)	11.0 (5.8 – 20.8)	23.5 (12.3 – 31.5)§	68.1% (38.5 – 83.4)§	0.86 (0.75 – 0.98)‡	0.85 (0.74 – 0.98)‡	0.82 (0.67 – 0.97)‡	-0.05 (-0.21 – 0.11)
	5th	27.8 (20.5 – 36.9)	18.1 (13.3 – 24.6)	9.4 (4.8 – 18.4)	18.4 (8.4 – 27.0)§	66.2% (34.2 – 82.5)§	0.69 (0.53 – 0.90)‡	0.70 (0.53 – 0.91)‡	0.70 (0.52 – 0.92)‡	0.00 (-0.13 – 0.14)
Algeria	1st	63.7 (53.7 – 74.5)	52.3 (45.0 – 59.9)	33.9 (28.7 – 39.8)	29.8 (20.1 – 40.0)§	46.7% (35.6 – 55.7)§	1.30 (1.11 – 1.50)‡	1.32 (1.14 – 1.51)‡	1.35 (1.16 – 1.54)‡	0.05 (-0.16 – 0.25)
	2nd	56.2 (49.0 – 63.6)	45.2 (40.2 – 50.3)	28.3 (24.5 – 32.4)	27.9 (20.5 – 35.3)§	49.7% (40.3 – 57.2)§	1.15 (1.02 – 1.28)‡	1.14 (1.02 – 1.26)‡	1.12 (1.00 – 1.25)	-0.02 (-0.17 – 0.13)
	3rd	48.9 (43.6 – 53.9)	39.2 (35.8 – 42.5)	24.7 (22.0 – 27.6)	24.2 (19.0 – 29.0)§	49.4% (42.4 – 55.5)§	1.00 (0.92 – 1.08)	0.99 (0.91 – 1.07)	0.98 (0.90 – 1.06)	-0.02 (-0.09 – 0.06)
	4th	41.6 (35.4 – 48.0)	33.7 (29.4 – 38.4)	21.1 (18.0 – 24.7)	20.5 (14.2 – 26.8)§	49.3% (38.0 – 57.8)§	0.85 (0.74 – 0.97)‡	0.85 (0.74 – 0.97)‡	0.84 (0.73 – 0.96)‡	-0.01 (-0.15 – 0.12)
	5th	34.6 (26.9 – 44.0)	28.0 (22.4 – 34.8)	17.9 (14.0 – 22.6)	16.6 (10.3 – 23.9)§	48.1% (35.7 – 58.0)§	0.71 (0.56 – 0.89)‡	0.71 (0.57 – 0.88)‡	0.71 (0.56 – 0.89)‡	0.01 (-0.13 – 0.14)
Angola	1st	232.3 (195.8 – 275.0)	217.5 (182.6 – 257.2)	98.7 (48.5 – 178.6)	133.6 (50.3 – 197.0)§	57.5% (22.7 – 79.2)§	1.05 (0.92 – 1.19)	1.05 (0.93 – 1.18)	1.20 (1.04 – 1.37)‡	0.15 (-0.02 – 0.32)
	2nd	248.7 (215.0 – 287.1)	232.6 (199.0 – 270.5)	94.3 (46.6 – 169.5)	154.4 (74.5 – 214.3)§	62.1% (31.0 – 81.3)§	1.12 (1.02 – 1.22)‡	1.12 (1.03 – 1.22)‡	1.14 (1.03 – 1.26)‡	0.02 (-0.11 – 0.16)
	3rd	234.0 (204.8 – 266.2)	219.1 (190.0 – 252.7)	85.9 (42.5 – 153.4)	148.1 (76.9 – 199.9)§	63.3% (34.0 – 81.8)§	1.06 (0.99 – 1.12)	1.06 (0.99 – 1.12)	1.04 (0.97 – 1.11)	-0.02 (-0.09 – 0.06)
	4th	221.0 (188.8 – 257.4)	205.9 (174.7 – 241.7)	72.6 (35.7 – 131.4)	148.4 (84.5 – 197.3)§	67.1% (39.7 – 83.8)§	1.00 (0.89 – 1.10)	0.99 (0.90 – 1.09)	0.88 (0.78 – 0.98)‡	-0.12 (-0.25 – 0.01)
	5th	171.0 (140.4 – 207.7)	160.0 (131.4 – 193.7)	60.9 (29.4 – 112.6)	110.1 (55.2 – 155.5)§	64.4% (34.0 – 82.9)§	0.77 (0.66 – 0.90)‡	0.77 (0.67 – 0.89)‡	0.74 (0.61 – 0.88)‡	-0.03 (-0.17 – 0.10)
Armenia	1st	65.5 (53.9 – 77.8)	40.7 (33.3 – 48.3)	18.7 (13.4 – 26.2)	46.8 (34.6 – 59.4)§	71.4% (59.5 – 79.8)§	1.32 (1.11 – 1.53)‡	1.35 (1.15 – 1.56)‡	1.40 (1.15 – 1.66)‡	0.08 (-0.15 – 0.32)
	2nd	58.1 (49.8 – 67.1)	34.9 (29.9 – 40.3)	15.4 (11.4 – 21.1)	42.7 (33.3 – 52.1)§	73.5% (62.9 – 80.7)§	1.17 (1.04 – 1.30)‡	1.16 (1.03 – 1.29)‡	1.15 (1.00 – 1.31)	-0.02 (-0.19 – 0.15)
	3rd	50.2 (44.0 – 56.5)	30.0 (26.1 – 34.1)	13.3 (10.0 – 17.9)	36.9 (29.6 – 43.8)§	73.5% (63.7 – 80.4)§	1.01 (0.92 – 1.09)	1.00 (0.91 – 1.08)	0.99 (0.89 – 1.09)	-0.02 (-0.11 – 0.08)
	4th	42.2 (35.5 – 49.3)	25.3 (21.1 – 29.8)	10.7 (7.6 – 14.8)	31.5 (24.1 – 39.0)§	74.7% (63.6 – 82.3)§	0.85 (0.74 – 0.97)‡	0.84 (0.72 – 0.96)‡	0.80 (0.65 – 0.95)‡	-0.05 (-0.21 – 0.11)
	5th	32.5 (24.0 – 43.1)	19.7 (14.5 – 25.9)	8.9 (5.9 – 13.2)	23.6 (16.0 – 32.7)§	72.7% (60.6 – 81.0)§	0.65 (0.49 – 0.85)‡	0.65 (0.49 – 0.85)‡	0.66 (0.49 – 0.87)‡	0.01 (-0.13 – 0.15)
Azerbaijan	1st	113.0 (94.5 – 133.3)	90.1 (74.9 – 107.2)	40.0 (23.5 – 69.4)	72.9 (40.0 – 97.3)§	64.6% (37.9 – 79.3)§	1.20 (1.03 – 1.37)‡	1.21 (1.05 – 1.39)‡	1.30 (1.10 – 1.50)‡	0.10 (-0.09 – 0.29)
	2nd	107.8 (93.1 – 123.5)	86.1 (73.5 – 99.9)	36.3 (21.4 – 61.4)	71.5 (42.3 – 92.5)§	66.3% (41.6 – 80.2)§	1.14 (1.02 – 1.26)‡	1.16 (1.04 – 1.28)‡	1.17 (1.04 – 1.31)‡	0.03 (-0.12 – 0.18)
	3rd	100.7 (88.9 – 113.4)	78.0 (67.8 – 89.2)	31.4 (18.9 – 53.4)	69.3 (44.2 – 86.3)§	68.8% (45.7 – 81.4)§	1.07 (0.99 – 1.14)	1.05 (0.97 – 1.13)	1.02 (0.93 – 1.10)	-0.05 (-0.13 – 0.03)
	4th	86.7 (74.0 – 100.5)	67.7 (57.2 – 79.2)	26.7 (15.8 – 46.4)	60.0 (37.8 – 77.2)§	69.2% (45.9 – 81.9)§	0.92 (0.81 – 1.03)	0.91 (0.81 – 1.02)	0.86 (0.75 – 0.99)‡	-0.05 (-0.19 – 0.09)
	5th	64.3 (49.6 – 82.0)	49.5 (37.9 – 63.7)	20.1 (11.3 – 36.2)	44.2 (25.4 – 61.4)§	68.7% (44.9 – 81.8)§	0.68 (0.53 – 0.85)‡	0.67 (0.52 – 0.84)‡	0.65 (0.50 – 0.83)‡	-0.03 (-0.16 – 0.09)
Bangladesh	1st	172.9 (159.9 – 186.7)	111.4 (102.4 – 120.9)	45.3 (38.7 – 52.5)	127.7 (113.3 – 142.2)§	73.8% (69.3 – 77.9)§	1.20 (1.12 – 1.29)‡	1.27 (1.18 – 1.37)‡	1.32 (1.18 – 1.47)‡	0.12 (-0.04 – 0.28)
	2nd	166.0 (154.5 – 177.9)	99.2 (91.3 – 106.6)	40.0 (34.8 – 45.7)	126.0 (113.6 – 138.9)§	75.9% (72.1 – 79.3)§	1.15 (1.08 – 1.23)‡	1.13 (1.05 – 1.21)‡	1.17 (1.06 – 1.28)‡	0.01 (-0.11 – 0.14)
	3rd	150.4 (141.9 – 158.8)	90.9 (85.5 – 96.3)	34.4 (30.5 – 38.6)	115.9 (106.5 – 124.9)§	77.1% (74.0 – 79.9)§	1.05 (1.00 – 1.09)	1.04 (0.99 – 1.09)	1.01 (0.94 – 1.07)	-0.04 (-0.11 – 0.04)
	4th	133.6 (122.7 – 144.6)	77.8 (71.3 – 84.6)	29.2 (25.0 – 33.8)	104.4 (92.8 – 115.9)§	78.2% (74.2 – 81.6)§	0.93 (0.86 – 1.00)	0.89 (0.82 – 0.96)‡	0.85 (0.76 – 0.95)‡	-0.08 (-0.19 – 0.04)
	5th	96.1 (87.2 – 105.6)	57.8 (52.2 – 63.5)	22.2 (18.6 – 26.3)	73.9 (64.4 – 83.6)§	76.9% (72.2 – 80.9)§	0.67 (0.61 – 0.73)‡	0.66 (0.60 – 0.72)‡	0.65 (0.56 – 0.75)‡	-0.02 (-0.12 – 0.09)
Belarus	1st	20.9 (17.1 – 25.0)	17.7 (14.4 – 21.1)	5.4 (4.3 – 6.4)	15.5 (12.4 – 19.0)§	74.3% (70.3 – 78.1)§	1.37 (1.13 – 1.64)‡	1.38 (1.13 – 1.65)‡	1.38 (1.13 – 1.65)‡	0.00 (-0.19 – 0.19)
	2nd	17.5 (15.1 – 20.2)	14.7 (12.6 – 16.9)	4.5 (3.8 – 5.2)	13.0 (10.9 – 15.3)§	74.4% (70.7 – 77.8)§	1.15 (0.99 – 1.32)	1.15 (0.99 – 1.32)	1.15 (1.00 – 1.32)	0.00 (-0.14 – 0.14)
	3rd	15.3 (13.7 – 16.9)	12.9 (11.4 – 14.2)	3.9 (3.4 – 4.3)	11.4 (10.0 – 12.7)§	74.4% (72.1 – 76.8)§	1.00 (0.90 – 1.11)	1.00 (0.90 – 1.11)	1.00 (0.90 – 1.11)	0.00 (-0.07 – 0.07)
	4th	12.2 (10.0 – 14.6)	10.3 (8.4 – 12.3)	3.1 (2.5 – 3.8)	9.1 (7.2 – 11.1)§	74.4% (70.4 – 78.3)§	0.81 (0.66 – 0.96)‡	0.80 (0.66 – 0.96)‡	0.80 (0.65 – 0.96)‡	0.00 (-0.12 – 0.11)
	5th	10.1 (7.4 – 13.5)	8.5 (6.2 – 11.3)	2.6 (1.9 – 3.5)	7.5 (5.4 – 10.3)§	74.3% (69.2 – 79.0)§	0.66 (0.49 – 0.88)‡	0.66 (0.49 – 0.88)‡	0.66 (0.48 – 0.89)‡	0.00 (-0.12 – 0.12)

Continued on next page

‡: Ratio is significantly different from one.
§: Change/decline is significantly different from zero.
Absolute decline: wealth quintile-specific U5MR (1990) - wealth quintile-specific U5MR (2016).
Percentage decline: absolute decline over wealth quintile-specific U5MR (1990) ×100.
Change in ratio: ratio (2016) - ratio (1990).

Table 8 – continued from previous page

	Wealth Quintile	wealth quintile-specific U5MR (deaths per 1000 livebirths) 1990	2000	2016	Absolute decline 1990–2016	Percentage decline 1990–2016	Ratio of wealth quintile-specific to national-level U5MR 1990	2000	2016	Change 1990–2016
Belize	1st	51.2 (41.2 – 62.9)	32.4 (26.7 – 38.7)	20.5 (16.3 – 25.1)	30.8 (21.4 – 41.6)§	60.1% (49.4 – 68.4)§	1.31 (1.09 – 1.55)‡	1.34 (1.12 – 1.59)‡	1.37 (1.13 – 1.65)‡	0.06 (-0.17 – 0.31)
	2nd	45.7 (38.4 – 54.0)	27.8 (24.1 – 31.9)	17.2 (14.4 – 20.2)	28.5 (21.2 – 36.5)§	62.4% (53.4 – 69.6)§	1.17 (1.04 – 1.31)‡	1.15 (1.02 – 1.29)‡	1.15 (1.00 – 1.32)	-0.02 (-0.19 – 0.15)
	3rd	39.5 (33.8 – 45.5)	24.3 (21.7 – 27.0)	15.0 (12.9 – 17.1)	24.5 (19.0 – 30.5)§	62.1% (54.7 – 68.3)§	1.01 (0.92 – 1.09)	1.01 (0.91 – 1.10)	1.00 (0.90 – 1.10)	-0.01 (-0.10 – 0.08)
	4th	33.0 (27.2 – 39.5)	20.1 (16.9 – 23.6)	12.0 (9.5 – 14.6)	21.0 (15.2 – 27.6)§	63.5% (53.8 – 72.1)§	0.85 (0.73 – 0.97)‡	0.83 (0.71 – 0.96)‡	0.81 (0.66 – 0.96)‡	-0.04 (-0.21 – 0.11)
	5th	25.6 (18.4 – 34.8)	15.9 (11.6 – 21.2)	9.8 (7.1 – 13.3)	15.8 (10.0 – 23.0)§	61.6% (50.4 – 70.3)§	0.66 (0.48 – 0.87)‡	0.66 (0.49 – 0.87)‡	0.66 (0.48 – 0.88)‡	0.00 (-0.13 – 0.15)
Benin	1st	198.8 (177.1 – 222.5)	164.5 (147.9 – 183.1)	116.6 (86.6 – 161.5)	82.2 (34.6 – 118.1)§	41.3% (18.3 – 56.9)§	1.11 (1.01 – 1.22)‡	1.14 (1.06 – 1.22)‡	1.19 (1.07 – 1.34)‡	0.08 (-0.08 – 0.24)
	2nd	199.9 (180.5 – 220.9)	162.0 (146.6 – 179.2)	112.3 (84.5 – 152.3)	87.6 (44.0 – 121.4)§	43.8% (22.8 – 58.3)§	1.12 (1.03 – 1.21)‡	1.12 (1.05 – 1.20)‡	1.15 (1.04 – 1.26)‡	0.03 (-0.10 – 0.17)
	3rd	193.2 (177.6 – 210.5)	158.2 (145.0 – 173.1)	105.3 (79.8 – 142.8)	87.9 (49.6 – 117.2)§	45.5% (26.0 – 58.7)§	1.08 (1.03 – 1.14)‡	1.10 (1.04 – 1.15)‡	1.08 (1.02 – 1.14)‡	0.00 (-0.08 – 0.07)
	4th	172.4 (153.6 – 192.3)	136.4 (122.8 – 151.6)	86.8 (64.8 – 120.4)	85.6 (49.1 – 114.7)§	49.6% (29.7 – 63.0)§	0.97 (0.88 – 1.06)	0.94 (0.88 – 1.02)	0.89 (0.79 – 0.99)‡	-0.08 (-0.20 – 0.05)
	5th	127.7 (111.3 – 146.9)	100.9 (88.7 – 114.4)	67.0 (48.9 – 93.8)	60.7 (32.4 – 85.2)§	47.5% (26.4 – 62.4)§	0.72 (0.63 – 0.81)‡	0.70 (0.63 – 0.77)‡	0.69 (0.58 – 0.80)‡	-0.03 (-0.15 – 0.09)
Bhutan	1st	164.6 (135.2 – 200.3)	106.5 (89.0 – 126.7)	46.9 (32.0 – 66.5)	117.7 (84.0 – 154.9)§	71.5% (58.3 – 81.0)§	1.29 (1.09 – 1.50)‡	1.38 (1.18 – 1.59)‡	1.45 (1.23 – 1.68)‡	0.16 (-0.05 – 0.38)
	2nd	143.8 (122.1 – 170.5)	88.2 (75.8 – 101.5)	37.4 (25.8 – 52.1)	106.5 (79.8 – 135.2)§	74.0% (62.4 – 82.6)§	1.13 (1.00 – 1.25)‡	1.14 (1.01 – 1.26)‡	1.15 (1.01 – 1.29)‡	0.03 (-0.13 – 0.18)
	3rd	134.1 (116.6 – 155.7)	79.6 (70.2 – 90.0)	32.2 (22.4 – 44.5)	101.9 (80.1 – 125.9)§	76.0% (65.8 – 83.8)§	1.05 (0.97 – 1.13)	1.03 (0.95 – 1.10)	0.99 (0.91 – 1.08)	-0.06 (-0.14 – 0.03)
	4th	117.1 (98.2 – 140.7)	66.7 (56.2 – 78.1)	26.7 (18.1 – 37.6)	90.4 (69.1 – 114.8)§	77.2% (66.9 – 85.1)§	0.92 (0.80 – 1.05)	0.86 (0.75 – 0.97)‡	0.82 (0.71 – 0.94)‡	-0.09 (-0.23 – 0.05)
	5th	78.3 (58.1 – 104.8)	46.1 (34.4 – 60.5)	18.8 (11.9 – 28.7)	59.5 (40.2 – 84.1)§	76.0% (64.3 – 84.5)§	0.61 (0.46 – 0.79)‡	0.60 (0.45 – 0.76)‡	0.58 (0.44 – 0.76)‡	-0.03 (-0.15 – 0.09)
Bolivia (Plurinational State of)	1st	170.8 (154.5 – 188.0)	113.8 (102.8 – 125.5)	55.1 (37.4 – 78.1)	115.7 (88.9 – 138.9)§	67.7% (53.9 – 78.1)§	1.38 (1.27 – 1.49)‡	1.43 (1.32 – 1.53)‡	1.49 (1.31 – 1.68)‡	0.11 (-0.08 – 0.30)
	2nd	143.0 (130.4 – 157.1)	95.8 (86.4 – 105.8)	43.8 (30.0 – 61.7)	99.1 (78.1 – 117.8)§	69.3% (56.6 – 79.1)§	1.15 (1.07 – 1.24)‡	1.20 (1.12 – 1.29)‡	1.19 (1.07 – 1.31)‡	0.03 (-0.11 – 0.17)
	3rd	130.5 (120.5 – 140.9)	82.1 (75.2 – 89.5)	36.9 (25.6 – 51.9)	93.6 (76.8 – 108.5)§	71.7% (60.3 – 80.4)§	1.05 (1.00 – 1.11)	1.03 (0.97 – 1.09)	1.00 (0.93 – 1.07)	-0.05 (-0.14 – 0.03)
	4th	111.1 (99.8 – 122.9)	67.6 (60.1 – 75.4)	30.1 (20.5 – 42.9)	81.0 (64.3 – 95.5)§	72.9% (61.0 – 81.5)§	0.90 (0.82 – 0.98)‡	0.85 (0.77 – 0.93)‡	0.82 (0.72 – 0.92)‡	-0.08 (-0.20 – 0.04)
	5th	63.6 (55.4 – 72.5)	39.6 (34.5 – 45.3)	18.6 (12.4 – 27.1)	45.1 (34.0 – 55.2)§	70.8% (56.9 – 80.5)§	0.51 (0.45 – 0.58)‡	0.50 (0.44 – 0.56)‡	0.50 (0.42 – 0.60)‡	-0.01 (-0.10 – 0.09)
Brazil	1st	93.9 (80.7 – 108.1)	53.9 (46.0 – 62.8)	23.1 (17.7 – 29.6)	70.9 (57.6 – 84.6)§	75.5% (68.5 – 81.2)§	1.46 (1.28 – 1.66)‡	1.51 (1.30 – 1.72)‡	1.53 (1.27 – 1.80)‡	0.06 (-0.18 – 0.32)
	2nd	73.9 (65.1 – 83.2)	41.3 (36.0 – 46.5)	17.2 (13.5 – 21.6)	56.7 (47.3 – 66.2)§	76.8% (70.3 – 82.0)§	1.15 (1.03 – 1.27)‡	1.15 (1.02 – 1.28)‡	1.14 (0.98 – 1.30)	-0.01 (-0.18 – 0.15)
	3rd	64.2 (57.6 – 70.8)	35.1 (31.3 – 38.9)	14.8 (11.8 – 18.3)	49.4 (42.5 – 56.1)§	76.9% (71.4 – 81.7)§	1.00 (0.92 – 1.08)	0.98 (0.90 – 1.06)	0.98 (0.88 – 1.08)	-0.02 (-0.11 – 0.07)
	4th	52.6 (45.0 – 60.6)	28.7 (24.4 – 33.3)	11.7 (8.8 – 15.1)	40.9 (33.1 – 49.0)§	77.7% (70.7 – 83.4)§	0.82 (0.71 – 0.93)‡	0.80 (0.69 – 0.92)‡	0.78 (0.63 – 0.92)‡	-0.04 (-0.20 – 0.11)
	5th	36.4 (27.8 – 46.9)	20.1 (15.2 – 25.9)	8.7 (6.1 – 12.1)	27.6 (20.1 – 36.8)§	76.0% (68.0 – 82.1)§	0.57 (0.44 – 0.72)‡	0.56 (0.43 – 0.72)‡	0.58 (0.43 – 0.76)‡	0.01 (-0.10 – 0.14)
Burkina Faso	1st	208.4 (187.5 – 229.8)	189.5 (171.3 – 209.0)	101.0 (75.5 – 133.6)	107.4 (70.0 – 138.2)§	51.5% (35.0 – 63.7)§	1.05 (0.97 – 1.13)	1.05 (0.97 – 1.13)	1.19 (1.06 – 1.34)‡	0.14 (0.00 – 0.30)
	2nd	220.6 (200.7 – 243.1)	206.4 (187.6 – 226.6)	97.9 (74.8 – 127.9)	122.8 (88.4 – 153.0)§	55.6% (41.8 – 66.3)§	1.11 (1.03 – 1.19)‡	1.14 (1.07 – 1.22)‡	1.16 (1.06 – 1.27)‡	0.05 (-0.08 – 0.18)
	3rd	213.9 (197.2 – 231.6)	193.0 (178.1 – 209.3)	88.9 (68.4 – 114.7)	125.0 (96.3 – 150.7)§	58.4% (46.3 – 68.1)§	1.08 (1.03 – 1.13)‡	1.07 (1.02 – 1.12)‡	1.05 (0.99 – 1.11)	-0.03 (-0.10 – 0.04)
	4th	203.6 (184.1 – 224.8)	182.4 (165.2 – 201.6)	75.8 (57.3 – 99.6)	127.7 (98.1 – 154.3)§	62.8% (50.3 – 72.0)§	1.03 (0.95 – 1.10)	1.01 (0.94 – 1.08)	0.90 (0.80 – 1.00)‡	-0.13 (-0.25 – -0.01)§
	5th	146.5 (131.0 – 163.5)	131.6 (117.4 – 146.9)	59.5 (44.4 – 79.4)	87.1 (63.5 – 108.6)§	59.4% (45.6 – 70.0)§	0.74 (0.67 – 0.81)‡	0.73 (0.67 – 0.80)‡	0.70 (0.61 – 0.81)‡	-0.04 (-0.14 – 0.08)
Burundi	1st	204.8 (172.8 – 241.5)	186.1 (159.2 – 218.0)	94.5 (71.1 – 123.1)	110.4 (71.6 – 149.1)§	53.9% (38.4 – 65.8)§	1.20 (1.05 – 1.38)‡	1.23 (1.09 – 1.39)‡	1.32 (1.14 – 1.51)‡	0.11 (-0.08 – 0.30)
	2nd	189.5 (164.3 – 215.8)	167.7 (145.9 – 191.1)	82.0 (62.8 – 106.6)	107.5 (74.5 – 138.7)§	56.7% (42.0 – 67.5)§	1.11 (1.00 – 1.22)‡	1.11 (1.01 – 1.21)‡	1.14 (1.03 – 1.27)‡	0.03 (-0.11 – 0.17)
	3rd	179.2 (158.7 – 200.3)	159.2 (141.1 – 178.8)	74.0 (57.5 – 94.7)	105.1 (77.0 – 130.6)§	58.7% (46.0 – 68.3)§	1.05 (0.98 – 1.12)	1.05 (0.98 – 1.13)	1.03 (0.96 – 1.11)	-0.02 (-0.09 – 0.05)
	4th	164.2 (140.6 – 189.0)	142.4 (123.1 – 163.7)	62.0 (47.4 – 81.5)	102.2 (73.6 – 130.2)§	62.2% (49.0 – 72.0)§	0.97 (0.86 – 1.08)	0.94 (0.84 – 1.04)	0.86 (0.76 – 0.97)‡	-0.10 (-0.23 – 0.03)
	5th	112.8 (90.8 – 138.6)	99.5 (81.6 – 121.4)	46.1 (33.7 – 62.7)	66.8 (44.3 – 91.3)§	59.2% (44.1 – 70.0)§	0.66 (0.54 – 0.80)‡	0.66 (0.55 – 0.78)‡	0.64 (0.52 – 0.79)‡	-0.02 (-0.14 – 0.10)
Cambodia	1st	147.3 (129.2 – 166.8)	138.0 (122.7 – 154.5)	42.8 (26.2 – 69.7)	104.5 (73.0 – 129.6)§	70.9% (51.9 – 82.4)§	1.27 (1.14 – 1.41)‡	1.29 (1.19 – 1.40)‡	1.40 (1.23 – 1.57)‡	0.13 (-0.07 – 0.33)
	2nd	134.7 (119.5 – 150.6)	123.9 (110.5 – 138.2)	36.5 (22.5 – 58.8)	98.2 (71.1 – 119.0)§	72.9% (55.5 – 83.4)§	1.16 (1.06 – 1.26)‡	1.16 (1.07 – 1.24)‡	1.19 (1.07 – 1.32)‡	0.03 (-0.12 – 0.19)
	3rd	127.0 (115.5 – 139.1)	117.4 (106.5 – 129.3)	31.8 (19.6 – 51.0)	95.2 (73.4 – 111.7)§	74.9% (59.6 – 84.6)§	1.09 (1.03 – 1.16)‡	1.10 (1.04 – 1.16)‡	1.04 (0.96 – 1.12)	-0.05 (-0.14 – 0.03)
	4th	110.5 (97.3 – 124.7)	100.5 (88.9 – 113.4)	26.3 (15.9 – 42.8)	84.1 (63.2 – 100.8)§	76.2% (60.8 – 85.6)§	0.95 (0.86 – 1.05)	0.94 (0.86 – 1.02)	0.86 (0.75 – 0.98)‡	-0.09 (-0.23 – 0.05)
	5th	61.0 (50.4 – 72.9)	55.3 (46.3 – 65.2)	15.5 (9.2 – 26.3)	45.4 (32.0 – 57.5)§	74.5% (57.2 – 84.7)§	0.53 (0.44 – 0.62)‡	0.52 (0.44 – 0.60)‡	0.51 (0.41 – 0.62)‡	-0.02 (-0.12 – 0.09)
Cameroon	1st	185.6 (165.0 – 207.6)	205.8 (183.8 – 230.4)	106.5 (79.8 – 142.2)	79.1 (40.9 – 111.6)§	42.6% (23.4 – 57.4)§	1.30 (1.18 – 1.42)‡	1.24 (1.15 – 1.34)‡	1.34 (1.17 – 1.50)‡	0.04 (-0.14 – 0.22)
	2nd	162.0 (145.9 – 179.9)	186.9 (167.8 – 207.4)	90.4 (68.6 – 119.1)	71.6 (40.5 – 97.9)§	44.2% (26.0 – 57.6)§	1.13 (1.05 – 1.22)‡	1.13 (1.05 – 1.21)‡	1.13 (1.03 – 1.24)‡	0.00 (-0.13 – 0.13)
	3rd	146.7 (133.6 – 160.5)	170.2 (154.4 – 187.1)	81.2 (62.5 – 105.6)	65.6 (40.3 – 86.9)§	44.7% (28.1 – 57.5)§	1.03 (0.97 – 1.08)	1.03 (0.97 – 1.08)	1.02 (0.95 – 1.08)	-0.01 (-0.08 – 0.07)
	4th	128.0 (113.6 – 143.3)	156.0 (139.4 – 174.8)	68.4 (51.8 – 90.6)	59.6 (34.5 – 80.9)§	46.6% (28.0 – 59.9)§	0.89 (0.81 – 0.98)‡	0.94 (0.87 – 1.02)	0.86 (0.76 – 0.96)‡	-0.04 (-0.15 – 0.09)
	5th	93.2 (80.9 – 107.1)	109.7 (96.5 – 125.0)	52.0 (38.3 – 71.0)	41.2 (20.6 – 58.2)§	44.2% (23.2 – 59.0)§	0.65 (0.58 – 0.73)‡	0.66 (0.60 – 0.73)‡	0.65 (0.55 – 0.77)‡	0.00 (-0.11 – 0.12)

Continued on next page

‡: Ratio is significantly different from one.
§: Change/decline is significantly different from zero.
Absolute decline: wealth quintile-specific U5MR (1990) - wealth quintile-specific U5MR (2016).
Percentage decline: absolute decline over wealth quintile-specific U5MR (1990) ×100.
Change in ratio: ratio (2016) - ratio (1990).

Table 8 – continued from previous page

	Wealth Quintile	wealth quintile-specific U5MR (deaths per 1000 livebirths) 1990	2000	2016	Absolute decline 1990–2016	Percentage decline 1990–2016	Ratio of wealth quintile-specific to national-level U5MR 1990	2000	2016	Change 1990–2016
Central African Republic	1st	199.3 (174.5 – 227.5)	204.4 (177.5 – 234.2)	154.3 (97.6 – 242.2)	44.9 (-45.5 – 107.5)	22.5% (-24.2 – 51.8)	1.15 (1.05 – 1.25)‡	1.19 (1.09 – 1.29)‡	1.25 (1.10 – 1.40)‡	0.10 (-0.06 – 0.27)
	2nd	202.0 (178.5 – 229.6)	198.3 (173.2 – 226.0)	139.3 (89.0 – 218.4)	62.7 (-18.9 – 120.4)	31.1% (-9.9 – 56.8)	1.16 (1.08 – 1.24)‡	1.15 (1.07 – 1.24)‡	1.13 (1.02 – 1.23)‡	-0.04 (-0.16 – 0.09)
	3rd	186.4 (165.6 – 209.7)	183.2 (161.5 – 206.6)	130.4 (83.8 – 203.0)	55.9 (-19.3 – 108.1)	30.0% (-11.0 – 55.7)	1.07 (1.01 – 1.13)‡	1.06 (1.01 – 1.12)‡	1.06 (0.99 – 1.12)	-0.02 (-0.09 – 0.05)
	4th	163.1 (142.5 – 186.7)	159.1 (137.4 – 183.1)	111.0 (70.4 – 175.1)	52.0 (-15.1 – 98.7)	31.9% (-9.5 – 57.4)	0.94 (0.86 – 1.02)	0.92 (0.84 – 1.00)	0.90 (0.80 – 1.00)	-0.04 (-0.15 – 0.08)
	5th	118.3 (101.1 – 137.7)	116.5 (99.4 – 135.9)	82.9 (52.1 – 132.4)	35.4 (-16.5 – 71.2)	29.9% (-14.7 – 56.8)	0.68 (0.61 – 0.76)‡	0.68 (0.60 – 0.76)‡	0.67 (0.57 – 0.79)‡	-0.01 (-0.12 – 0.10)
Chad	1st	178.1 (156.7 – 201.0)	158.7 (142.2 – 176.7)	126.2 (101.3 – 155.5)	51.9 (17.7 – 84.0)§	29.1% (10.6 – 44.0)§	0.84 (0.76 – 0.93)‡	0.86 (0.79 – 0.93)‡	0.99 (0.88 – 1.12)	0.15 (0.02 – 0.29)§
	2nd	241.6 (217.6 – 266.3)	206.1 (187.1 – 227.6)	141.3 (114.8 – 169.5)	100.3 (65.4 – 136.0)§	41.5% (28.4 – 53.3)§	1.15 (1.06 – 1.23)‡	1.11 (1.04 – 1.19)‡	1.11 (1.02 – 1.20)‡	-0.04 (-0.16 – 0.09)
	3rd	225.2 (205.7 – 246.1)	197.2 (180.8 – 215.8)	133.6 (110.1 – 159.1)	91.6 (61.4 – 120.4)§	40.7% (28.4 – 51.3)§	1.07 (1.01 – 1.12)‡	1.07 (1.01 – 1.12)‡	1.05 (0.99 – 1.11)	-0.02 (-0.09 – 0.05)
	4th	216.0 (192.7 – 241.0)	191.9 (172.8 – 213.2)	119.6 (96.5 – 144.8)	96.4 (64.5 – 128.5)§	44.6% (31.8 – 56.0)§	1.02 (0.94 – 1.11)	1.04 (0.96 – 1.12)	0.94 (0.85 – 1.04)	-0.09 (-0.21 – 0.04)
	5th	193.1 (171.0 – 217.7)	171.5 (153.9 – 191.2)	115.8 (92.3 – 141.0)	77.3 (45.6 – 108.9)§	40.0% (25.1 – 52.8)§	0.92 (0.83 – 1.00)	0.93 (0.85 – 1.00)	0.91 (0.81 – 1.02)	-0.01 (-0.14 – 0.12)
Colombia	1st	47.3 (41.3 – 53.6)	34.4 (30.3 – 38.9)	21.5 (16.1 – 28.4)	25.7 (17.1 – 33.4)§	54.5% (38.9 – 66.3)§	1.35 (1.20 – 1.49)‡	1.38 (1.24 – 1.52)‡	1.41 (1.22 – 1.60)‡	0.06 (-0.15 – 0.28)
	2nd	40.1 (35.5 – 45.0)	28.4 (25.1 – 31.9)	17.6 (13.2 – 22.9)	22.5 (16.0 – 28.5)§	56.2% (42.3 – 67.1)§	1.14 (1.03 – 1.25)‡	1.14 (1.03 – 1.24)‡	1.15 (1.02 – 1.28)‡	0.01 (-0.15 – 0.17)
	3rd	35.0 (31.6 – 38.6)	24.8 (22.3 – 27.5)	15.1 (11.6 – 19.4)	19.9 (14.8 – 24.4)§	56.9% (44.2 – 66.9)§	1.00 (0.93 – 1.07)	0.99 (0.92 – 1.06)	0.99 (0.90 – 1.07)	-0.01 (-0.10 – 0.08)
	4th	28.9 (25.2 – 33.0)	19.9 (17.2 – 22.8)	11.8 (8.7 – 15.7)	17.1 (12.1 – 21.9)§	59.3% (45.0 – 70.2)§	0.82 (0.73 – 0.92)‡	0.80 (0.70 – 0.90)‡	0.77 (0.64 – 0.90)‡	-0.05 (-0.20 – 0.09)
	5th	24.3 (20.2 – 29.1)	17.5 (14.6 – 20.7)	10.6 (7.6 – 14.4)	13.7 (8.9 – 18.6)§	56.5% (40.6 – 68.2)§	0.69 (0.58 – 0.82)‡	0.70 (0.59 – 0.82)‡	0.69 (0.57 – 0.83)‡	0.00 (-0.13 – 0.13)
Comoros	1st	148.8 (123.5 – 177.0)	124.8 (95.9 – 153.3)	90.1 (46.9 – 181.5)	58.7 (-34.8 – 107.6)	39.5% (-24.2 – 68.4)	1.18 (1.03 – 1.35)‡	1.21 (1.05 – 1.38)‡	1.23 (1.05 – 1.41)‡	0.05 (-0.13 – 0.23)
	2nd	141.4 (120.2 – 162.6)	115.2 (90.0 – 138.5)	82.9 (43.5 – 163.0)	58.5 (-25.9 – 102.0)	41.4% (-19.8 – 69.3)	1.12 (1.01 – 1.23)‡	1.12 (1.01 – 1.23)‡	1.13 (1.01 – 1.25)‡	0.01 (-0.13 – 0.15)
	3rd	133.3 (115.6 – 150.9)	108.4 (85.5 – 128.4)	75.6 (40.2 – 148.4)	57.7 (-19.3 – 96.0)	43.3% (-15.1 – 69.9)	1.06 (0.99 – 1.13)	1.05 (0.98 – 1.12)	1.03 (0.95 – 1.11)	-0.03 (-0.10 – 0.04)
	4th	113.8 (95.8 – 132.7)	89.9 (69.8 – 109.2)	63.6 (33.3 – 125.4)	50.2 (-15.8 – 84.8)	44.1% (-14.6 – 70.9)	0.90 (0.80 – 1.01)	0.87 (0.77 – 0.98)‡	0.87 (0.76 – 0.98)‡	-0.04 (-0.16 – 0.09)
	5th	91.8 (73.4 – 112.6)	76.2 (57.1 – 97.2)	54.3 (27.8 – 110.1)	37.4 (-20.3 – 68.1)	40.8% (-22.9 – 69.5)	0.73 (0.60 – 0.87)‡	0.74 (0.61 – 0.89)‡	0.74 (0.60 – 0.90)‡	0.01 (-0.11 – 0.14)
Congo	1st	102.5 (84.8 – 122.5)	127.0 (109.3 – 147.1)	63.2 (43.6 – 87.8)	39.4 (10.1 – 65.5)§	38.4% (10.7 – 58.5)§	1.13 (0.99 – 1.28)	1.08 (0.97 – 1.20)	1.17 (1.02 – 1.32)‡	0.04 (-0.13 – 0.22)
	2nd	104.6 (88.4 – 122.4)	134.5 (117.2 – 153.3)	64.0 (44.6 – 88.6)	40.6 (12.1 – 66.7)§	38.8% (12.5 – 58.3)§	1.15 (1.04 – 1.27)‡	1.15 (1.05 – 1.25)‡	1.18 (1.07 – 1.30)‡	0.03 (-0.11 – 0.17)
	3rd	96.7 (83.5 – 111.6)	126.3 (111.8 – 142.4)	56.1 (39.7 – 77.2)	40.7 (16.0 – 62.2)§	42.0% (17.7 – 59.8)§	1.07 (0.99 – 1.13)	1.08 (1.01 – 1.14)‡	1.04 (0.96 – 1.11)	-0.03 (-0.11 – 0.05)
	4th	82.0 (68.9 – 96.7)	109.0 (94.1 – 125.9)	47.3 (32.8 – 65.7)	34.7 (12.1 – 55.1)§	42.3% (16.3 – 61.4)§	0.90 (0.80 – 1.01)	0.93 (0.84 – 1.02)	0.87 (0.78 – 0.98)‡	-0.03 (-0.16 – 0.10)
	5th	68.1 (53.8 – 85.5)	89.7 (74.0 – 108.4)	40.0 (27.5 – 57.2)	28.2 (8.1 – 47.3)§	41.3% (13.4 – 60.9)§	0.75 (0.62 – 0.90)‡	0.76 (0.65 – 0.90)‡	0.74 (0.61 – 0.89)‡	-0.01 (-0.15 – 0.12)
Cote d'Ivoire	1st	181.9 (162.3 – 203.3)	173.4 (152.5 – 196.0)	113.8 (83.0 – 153.9)	68.1 (26.5 – 103.1)§	37.5% (14.9 – 54.8)§	1.21 (1.10 – 1.32)‡	1.19 (1.08 – 1.31)‡	1.24 (1.09 – 1.39)‡	0.03 (-0.13 – 0.20)
	2nd	172.3 (155.2 – 190.8)	167.3 (148.9 – 187.1)	104.4 (76.3 – 141.2)	67.9 (29.3 – 100.3)§	39.4% (17.5 – 56.0)§	1.14 (1.06 – 1.23)‡	1.15 (1.06 – 1.24)‡	1.14 (1.03 – 1.25)‡	0.00 (-0.14 – 0.12)
	3rd	155.7 (142.0 – 169.6)	151.0 (136.1 – 166.1)	95.2 (70.5 – 126.7)	60.5 (27.6 – 87.3)§	38.9% (18.2 – 54.8)§	1.03 (0.97 – 1.09)	1.04 (0.97 – 1.09)	1.04 (0.97 – 1.10)	0.01 (-0.07 – 0.07)
	4th	143.6 (128.5 – 159.6)	138.0 (121.8 – 155.3)	81.8 (59.8 – 110.8)	61.7 (30.7 – 87.8)§	43.0% (21.8 – 58.8)§	0.95 (0.87 – 1.03)	0.95 (0.86 – 1.03)	0.89 (0.80 – 0.99)‡	-0.06 (-0.18 – 0.06)
	5th	101.0 (88.0 – 115.0)	98.9 (85.9 – 114.4)	63.8 (45.8 – 88.4)	37.2 (11.3 – 57.9)§	36.8% (11.7 – 54.7)§	0.67 (0.60 – 0.75)‡	0.68 (0.60 – 0.76)‡	0.69 (0.59 – 0.82)‡	0.03 (-0.08 – 0.15)
Democratic Republic of the Congo	1st	215.3 (185.1 – 249.4)	190.4 (167.1 – 217.0)	117.7 (82.2 – 164.7)	97.6 (44.8 – 144.8)§	45.3% (22.1 – 62.6)§	1.17 (1.05 – 1.31)‡	1.19 (1.09 – 1.30)‡	1.25 (1.10 – 1.40)‡	0.08 (-0.10 – 0.25)
	2nd	209.9 (183.3 – 239.0)	182.7 (161.2 – 206.0)	108.7 (76.0 – 150.9)	101.2 (53.4 – 142.5)§	48.2% (26.8 – 64.1)§	1.14 (1.04 – 1.24)‡	1.14 (1.06 – 1.23)‡	1.15 (1.04 – 1.26)‡	0.01 (-0.13 – 0.15)
	3rd	196.3 (174.6 – 220.0)	170.8 (152.7 – 191.1)	100.5 (70.7 – 139.0)	95.8 (54.1 – 132.0)§	48.8% (28.3 – 64.1)§	1.07 (1.01 – 1.13)‡	1.07 (1.01 – 1.13)‡	1.07 (1.00 – 1.13)	0.00 (-0.08 – 0.07)
	4th	185.5 (161.2 – 212.8)	158.6 (138.9 – 181.0)	85.9 (60.2 – 120.7)	99.5 (58.8 – 135.8)§	53.7% (33.9 – 68.0)§	1.01 (0.91 – 1.11)	0.99 (0.91 – 1.08)	0.91 (0.82 – 1.02)	-0.10 (-0.23 – 0.03)
	5th	111.0 (92.4 – 133.2)	96.4 (81.9 – 113.0)	58.7 (40.0 – 84.2)	52.3 (22.5 – 78.5)§	47.1% (22.5 – 64.2)§	0.60 (0.52 – 0.70)‡	0.60 (0.53 – 0.68)‡	0.62 (0.53 – 0.73)‡	0.02 (-0.09 – 0.13)
Dominican Republic	1st	77.8 (68.1 – 88.3)	54.7 (48.1 – 61.7)	40.0 (29.4 – 54.0)	37.7 (21.7 – 52.1)§	48.5% (29.5 – 62.6)§	1.30 (1.15 – 1.45)‡	1.33 (1.21 – 1.47)‡	1.30 (1.14 – 1.47)‡	0.01 (-0.18 – 0.20)
	2nd	70.0 (62.4 – 78.2)	47.4 (42.0 – 53.2)	35.8 (26.8 – 47.9)	34.2 (20.7 – 45.9)§	48.9% (30.9 – 62.4)§	1.17 (1.06 – 1.28)‡	1.16 (1.05 – 1.26)‡	1.17 (1.05 – 1.29)‡	0.00 (-0.15 – 0.15)
	3rd	61.5 (56.2 – 67.2)	41.6 (37.4 – 45.8)	31.1 (23.4 – 41.4)	30.4 (19.8 – 39.6)§	49.5% (32.9 – 62.2)§	1.03 (0.96 – 1.10)	1.01 (0.94 – 1.08)	1.01 (0.94 – 1.09)	-0.01 (-0.09 – 0.06)
	4th	52.2 (45.7 – 59.5)	35.2 (30.6 – 40.1)	26.5 (19.5 – 36.0)	25.7 (14.7 – 35.3)§	49.3% (30.2 – 63.0)§	0.87 (0.77 – 0.98)‡	0.86 (0.77 – 0.96)‡	0.86 (0.76 – 0.98)‡	-0.01 (-0.14 – 0.12)
	5th	38.0 (31.0 – 46.4)	26.2 (21.4 – 31.4)	20.1 (14.3 – 27.8)	17.9 (9.1 – 26.4)§	47.0% (26.3 – 62.0)§	0.63 (0.52 – 0.76)‡	0.64 (0.53 – 0.76)‡	0.65 (0.53 – 0.79)‡	0.02 (-0.10 – 0.14)
Egypt	1st	123.1 (112.1 – 134.7)	68.7 (62.4 – 75.3)	33.1 (25.3 – 43.5)	90.1 (75.3 – 103.6)§	73.1% (64.2 – 79.7)§	1.43 (1.32 – 1.55)‡	1.47 (1.36 – 1.57)‡	1.45 (1.28 – 1.62)‡	0.02 (-0.18 – 0.21)
	2nd	98.4 (89.6 – 107.5)	52.9 (47.8 – 58.2)	26.0 (19.8 – 34.3)	72.4 (60.4 – 83.5)§	73.6% (64.6 – 80.1)§	1.15 (1.06 – 1.23)‡	1.13 (1.04 – 1.21)‡	1.14 (1.03 – 1.26)‡	-0.01 (-0.15 – 0.14)
	3rd	88.5 (82.0 – 95.0)	46.9 (43.1 – 51.0)	22.8 (17.7 – 29.7)	65.7 (56.6 – 73.6)§	74.2% (66.1 – 80.0)§	1.03 (0.97 – 1.09)	1.00 (0.94 – 1.06)	1.00 (0.92 – 1.07)	-0.03 (-0.11 – 0.05)
	4th	73.7 (66.0 – 81.9)	41.0 (36.7 – 45.6)	19.2 (14.5 – 25.6)	54.4 (45.1 – 63.3)§	73.9% (65.0 – 80.5)§	0.86 (0.78 – 0.94)‡	0.87 (0.80 – 0.96)‡	0.84 (0.73 – 0.96)‡	-0.01 (-0.14 – 0.12)
	5th	45.8 (39.3 – 53.3)	25.0 (21.8 – 28.7)	12.9 (9.6 – 17.6)	32.9 (25.4 – 40.4)§	71.8% (61.2 – 79.3)§	0.53 (0.46 – 0.61)‡	0.53 (0.47 – 0.60)‡	0.57 (0.48 – 0.67)‡	0.03 (-0.07 – 0.14)

Continued on next page

‡: Ratio is significantly different from one.
§: Change/decline is significantly different from zero.
Absolute decline: wealth quintile-specific U5MR (1990) - wealth quintile-specific U5MR (2016).
Percentage decline: absolute decline over wealth quintile-specific U5MR (1990) $\times 100$.
Change in ratio: ratio (2016) - ratio (1990).

Table 8 – continued from previous page

	Wealth Quintile	wealth quintile-specific U5MR (deaths per 1000 livebirths) 1990	2000	2016	Absolute decline 1990–2016	Percentage decline 1990–2016	Ratio of wealth quintile-specific to national-level U5MR 1990	2000	2016	Change 1990–2016
El Salvador	1st	76.5 (62.7 – 91.7)	43.5 (35.4 – 52.5)	21.0 (13.8 – 31.3)	55.6 (40.1 – 71.1)§	72.6% (58.5 – 82.0)§	1.28 (1.07 – 1.50)‡	1.34 (1.12 – 1.56)‡	1.40 (1.16 – 1.65)‡	0.11 (-0.11 – 0.35)
	2nd	69.2 (59.8 – 79.7)	37.7 (32.1 – 43.9)	17.2 (11.6 – 25.5)	52.0 (40.0 – 63.4)§	75.2% (62.8 – 83.5)§	1.16 (1.03 – 1.30)‡	1.16 (1.03 – 1.30)‡	1.15 (0.99 – 1.31)	-0.02 (-0.18 – 0.15)
	3rd	60.8 (53.9 – 68.6)	32.5 (28.2 – 37.1)	14.9 (10.1 – 21.9)	45.9 (36.3 – 54.6)§	75.4% (63.6 – 83.5)§	1.02 (0.94 – 1.10)	1.00 (0.91 – 1.09)	1.00 (0.89 – 1.10)	-0.02 (-0.12 – 0.07)
	4th	51.1 (43.2 – 59.9)	27.1 (22.5 – 32.3)	11.9 (7.8 – 17.8)	39.3 (29.7 – 48.4)§	76.8% (64.7 – 85.1)§	0.86 (0.74 – 0.98)‡	0.84 (0.72 – 0.96)‡	0.79 (0.65 – 0.94)‡	-0.07 (-0.23 – 0.09)
	5th	40.3 (29.5 – 53.4)	21.8 (15.8 – 29.1)	10.1 (6.2 – 16.1)	30.2 (20.1 – 41.6)§	75.0% (61.1 – 83.8)§	0.68 (0.50 – 0.89)‡	0.67 (0.50 – 0.87)‡	0.67 (0.50 – 0.88)‡	0.00 (-0.14 – 0.14)
Equatorial Guinea	1st	206.6 (169.2 – 252.3)	169.5 (142.9 – 202.9)	109.0 (70.5 – 162.7)	97.6 (33.5 – 157.0)§	47.3% (18.3 – 67.1)§	1.08 (0.94 – 1.24)	1.12 (0.98 – 1.26)	1.20 (1.03 – 1.38)‡	0.12 (-0.06 – 0.29)
	2nd	199.7 (166.2 – 237.8)	158.3 (136.1 – 185.2)	97.7 (64.1 – 143.8)	102.0 (45.8 – 152.0)§	51.1% (25.0 – 69.3)§	1.05 (0.94 – 1.16)	1.04 (0.94 – 1.15)	1.07 (0.95 – 1.19)	0.03 (-0.11 – 0.16)
	3rd	190.9 (161.3 – 225.2)	151.3 (132.6 – 174.1)	90.8 (60.2 – 132.4)	100.1 (49.1 – 146.7)§	52.4% (28.1 – 69.7)§	1.00 (0.93 – 1.07)	1.00 (0.92 – 1.07)	1.00 (0.92 – 1.07)	0.00 (-0.07 – 0.07)
	4th	183.4 (150.5 – 221.2)	141.4 (120.7 – 166.9)	77.7 (50.7 – 114.0)	105.6 (58.6 – 150.7)§	57.6% (35.2 – 73.1)§	0.96 (0.85 – 1.07)	0.93 (0.82 – 1.04)	0.86 (0.75 – 0.97)‡	-0.11 (-0.23 – 0.03)
	5th	172.9 (137.3 – 216.9)	137.5 (111.8 – 169.2)	79.3 (50.0 – 122.2)	93.6 (44.8 – 141.1)§	54.1% (28.8 – 71.6)§	0.91 (0.76 – 1.08)	0.91 (0.76 – 1.07)	0.87 (0.70 – 1.07)	-0.03 (-0.19 – 0.12)
Eritrea	1st	150.8 (125.5 – 178.7)	93.7 (79.7 – 109.8)	52.1 (32.1 – 82.9)	98.7 (62.1 – 130.0)§	65.5% (44.0 – 78.8)§	1.00 (0.86 – 1.15)	1.05 (0.93 – 1.19)	1.17 (0.99 – 1.36)	0.17 (0.00 – 0.35)
	2nd	179.7 (156.9 – 204.4)	107.3 (93.8 – 121.9)	54.9 (34.9 – 86.4)	124.9 (86.6 – 155.2)§	69.5% (50.8 – 80.9)§	1.19 (1.08 – 1.31)‡	1.21 (1.10 – 1.31)‡	1.23 (1.10 – 1.37)‡	0.04 (-0.11 – 0.19)
	3rd	168.9 (150.9 – 188.8)	99.8 (88.9 – 112.0)	47.6 (30.2 – 75.5)	121.3 (89.9 – 146.6)§	71.8% (55.2 – 82.4)§	1.12 (1.05 – 1.19)‡	1.12 (1.05 – 1.19)‡	1.07 (0.99 – 1.15)	-0.05 (-0.13 – 0.03)
	4th	157.2 (135.8 – 181.1)	86.5 (74.9 – 99.9)	40.3 (25.1 – 64.1)	116.9 (86.0 – 143.8)§	74.3% (58.7 – 84.1)§	1.04 (0.93 – 1.16)	0.97 (0.88 – 1.08)	0.91 (0.79 – 1.03)	-0.14 (-0.28 – 0.01)
	5th	97.9 (76.8 – 123.2)	57.1 (45.2 – 71.6)	27.6 (16.7 – 45.6)	70.2 (46.2 – 94.2)§	71.8% (53.6 – 82.7)§	0.65 (0.52 – 0.80)‡	0.64 (0.52 – 0.79)‡	0.62 (0.49 – 0.78)‡	-0.03 (-0.15 – 0.09)
Ethiopia	1st	190.5 (164.6 – 218.7)	140.3 (125.1 – 156.6)	67.2 (52.0 – 87.4)	123.2 (92.2 – 153.6)§	64.7% (53.1 – 73.3)§	0.94 (0.83 – 1.05)	0.98 (0.90 – 1.06)	1.15 (1.01 – 1.30)‡	0.21 (0.04 – 0.39)§
	2nd	224.0 (198.9 – 249.7)	155.7 (139.6 – 172.1)	67.8 (52.9 – 86.4)	156.2 (127.0 – 184.4)§	69.7% (60.6 – 76.7)§	1.10 (1.00 – 1.20)‡	1.08 (1.00 – 1.16)‡	1.16 (1.05 – 1.28)‡	0.06 (-0.08 – 0.20)
	3rd	219.6 (199.9 – 240.6)	155.4 (141.4 – 169.6)	60.6 (47.7 – 76.4)	159.0 (134.6 – 182.2)§	72.4% (64.8 – 78.4)§	1.08 (1.02 – 1.14)‡	1.08 (1.02 – 1.14)‡	1.04 (0.97 – 1.11)	-0.04 (-0.12 – 0.03)
	4th	220.7 (194.7 – 248.1)	153.2 (138.1 – 169.9)	52.8 (40.6 – 68.0)	167.9 (139.0 – 196.4)§	76.1% (68.5 – 81.8)§	1.09 (0.98 – 1.19)	1.07 (0.99 – 1.15)	0.90 (0.80 – 1.01)	-0.18 (-0.32 – -0.04)§
	5th	161.3 (138.9 – 186.7)	113.9 (100.5 – 128.0)	43.6 (33.1 – 56.9)	117.7 (92.7 – 143.7)§	73.0% (63.9 – 79.8)§	0.79 (0.70 – 0.90)‡	0.79 (0.72 – 0.87)‡	0.75 (0.64 – 0.87)‡	-0.05 (-0.18 – 0.09)
Gabon	1st	104.3 (85.5 – 126.4)	95.1 (79.1 – 116.1)	57.5 (37.8 – 84.7)	46.8 (15.5 – 75.9)§	44.9% (16.3 – 64.7)§	1.13 (0.98 – 1.28)	1.12 (0.99 – 1.26)	1.21 (1.05 – 1.38)‡	0.08 (-0.09 – 0.27)
	2nd	109.1 (92.0 – 130.2)	100.6 (85.0 – 121.1)	56.7 (38.4 – 82.5)	52.4 (22.2 – 80.2)§	48.0% (22.1 – 66.3)§	1.18 (1.07 – 1.30)‡	1.19 (1.08 – 1.30)‡	1.20 (1.08 – 1.33)‡	0.02 (-0.13 – 0.17)
	3rd	99.1 (84.9 – 116.5)	90.5 (77.8 – 107.7)	49.1 (33.2 – 71.6)	50.0 (24.5 – 73.8)§	50.5% (26.6 – 67.5)§	1.07 (1.00 – 1.14)	1.07 (1.00 – 1.14)	1.04 (0.96 – 1.11)	-0.04 (-0.12 – 0.04)
	4th	85.0 (70.6 – 102.9)	77.8 (65.1 – 95.0)	41.2 (27.6 – 60.6)	43.8 (20.4 – 65.5)§	51.5% (26.3 – 68.8)§	0.92 (0.82 – 1.04)	0.92 (0.82 – 1.03)	0.87 (0.76 – 0.98)‡	-0.05 (-0.19 – 0.08)
	5th	64.5 (49.3 – 83.4)	59.1 (46.0 – 76.6)	32.5 (20.9 – 49.1)	32.0 (12.8 – 51.1)§	49.7% (22.4 – 68.0)§	0.70 (0.56 – 0.86)‡	0.70 (0.57 – 0.85)‡	0.68 (0.55 – 0.85)‡	-0.01 (-0.14 – 0.12)
Gambia	1st	183.8 (153.5 – 218.9)	136.5 (114.1 – 161.4)	78.9 (48.3 – 126.8)	104.9 (51.4 – 148.8)§	57.1% (29.5 – 74.2)§	1.10 (0.95 – 1.25)	1.17 (1.03 – 1.31)‡	1.21 (1.05 – 1.38)‡	0.11 (-0.07 – 0.29)
	2nd	191.2 (164.5 – 221.0)	133.1 (113.6 – 154.8)	77.0 (47.3 – 122.2)	114.1 (63.1 – 156.1)§	59.7% (35.3 – 75.4)§	1.14 (1.03 – 1.25)‡	1.14 (1.03 – 1.24)‡	1.18 (1.06 – 1.30)‡	0.04 (-0.11 – 0.19)
	3rd	180.5 (158.1 – 205.4)	126.6 (109.9 – 145.8)	68.9 (42.8 – 108.7)	111.6 (68.2 – 146.2)§	61.8% (39.1 – 76.4)§	1.08 (1.01 – 1.15)‡	1.08 (1.01 – 1.15)‡	1.06 (0.98 – 1.12)	-0.02 (-0.10 – 0.05)
	4th	168.8 (144.0 – 198.5)	110.5 (93.5 – 130.1)	59.4 (36.7 – 95.0)	109.4 (66.9 – 144.9)§	64.8% (42.8 – 78.6)§	1.01 (0.90 – 1.13)	0.94 (0.85 – 1.05)	0.91 (0.80 – 1.03)	-0.10 (-0.23 – 0.04)
	5th	113.7 (91.7 – 139.8)	78.8 (64.0 – 96.5)	42.2 (25.5 – 68.7)	71.5 (41.4 – 99.6)§	62.9% (39.7 – 77.6)§	0.68 (0.57 – 0.81)‡	0.67 (0.57 – 0.79)‡	0.65 (0.53 – 0.78)‡	-0.03 (-0.15 – 0.09)
Georgia	1st	60.3 (48.9 – 72.8)	45.6 (37.0 – 55.7)	14.5 (11.5 – 18.2)	45.8 (35.0 – 57.6)§	76.0% (69.2 – 81.0)§	1.28 (1.07 – 1.50)‡	1.29 (1.08 – 1.50)‡	1.36 (1.11 – 1.62)‡	0.08 (-0.15 – 0.31)
	2nd	56.6 (48.3 – 65.9)	42.9 (36.6 – 50.5)	12.6 (10.4 – 15.2)	44.0 (35.5 – 53.1)§	77.7% (72.0 – 82.2)§	1.20 (1.07 – 1.34)‡	1.21 (1.08 – 1.35)‡	1.18 (1.02 – 1.34)‡	-0.02 (-0.20 – 0.15)
	3rd	48.4 (42.1 – 55.3)	36.1 (31.3 – 41.8)	10.8 (9.2 – 12.7)	37.6 (31.3 – 44.3)§	77.6% (72.7 – 81.4)§	1.03 (0.94 – 1.11)	1.02 (0.93 – 1.10)	1.01 (0.91 – 1.11)	-0.01 (-0.11 – 0.08)
	4th	40.3 (33.5 – 47.7)	30.0 (24.9 – 36.0)	8.6 (6.8 – 10.8)	31.7 (24.9 – 38.9)§	78.6% (72.2 – 83.5)§	0.85 (0.74 – 0.98)‡	0.85 (0.73 – 0.97)‡	0.81 (0.66 – 0.96)‡	-0.05 (-0.21 – 0.11)
	5th	30.4 (22.1 – 40.7)	22.8 (16.6 – 30.8)	7.0 (5.0 – 9.5)	23.4 (16.2 – 32.2)§	77.1% (70.0 – 82.4)§	0.64 (0.48 – 0.85)‡	0.64 (0.48 – 0.85)‡	0.65 (0.47 – 0.86)‡	0.01 (-0.13 – 0.15)
Ghana	1st	155.8 (140.5 – 172.0)	125.9 (113.7 – 138.8)	76.3 (56.8 – 101.1)	79.6 (51.9 – 103.8)§	51.1% (34.6 – 63.9)§	1.23 (1.12 – 1.33)‡	1.26 (1.16 – 1.36)‡	1.30 (1.15 – 1.45)‡	0.07 (-0.09 – 0.24)
	2nd	141.2 (128.2 – 155.1)	107.2 (97.0 – 117.6)	65.0 (48.5 – 86.0)	76.2 (52.6 – 97.3)§	53.9% (38.6 – 65.8)§	1.11 (1.03 – 1.20)‡	1.07 (0.99 – 1.15)	1.11 (1.00 – 1.22)	-0.01 (-0.14 – 0.12)
	3rd	131.7 (121.7 – 142.2)	103.6 (95.2 – 112.5)	58.9 (44.3 – 77.3)	72.8 (52.8 – 89.5)§	55.3% (41.1 – 66.3)§	1.04 (0.98 – 1.10)	1.04 (0.98 – 1.09)	1.00 (0.93 – 1.07)	-0.04 (-0.11 – 0.03)
	4th	116.8 (105.1 – 129.4)	90.2 (80.8 – 100.4)	50.7 (37.7 – 67.5)	66.1 (46.6 – 83.4)§	56.6% (41.7 – 68.0)§	0.92 (0.84 – 1.01)	0.90 (0.82 – 0.98)‡	0.86 (0.77 – 0.96)‡	-0.06 (-0.18 – 0.06)
	5th	89.0 (77.2 – 102.0)	73.7 (64.6 – 84.4)	43.1 (31.5 – 58.5)	45.9 (28.4 – 61.6)§	51.6% (33.8 – 64.8)§	0.70 (0.62 – 0.79)‡	0.74 (0.66 – 0.83)‡	0.73 (0.62 – 0.85)‡	0.03 (-0.09 – 0.16)
Guatemala	1st	93.2 (81.6 – 105.2)	62.3 (54.1 – 71.2)	36.1 (28.4 – 45.5)	57.1 (43.4 – 69.7)§	61.3% (50.3 – 69.7)§	1.14 (1.01 – 1.27)‡	1.20 (1.06 – 1.34)‡	1.27 (1.09 – 1.44)‡	0.13 (-0.05 – 0.31)
	2nd	95.8 (85.7 – 105.9)	62.7 (55.3 – 70.6)	34.5 (27.9 – 43.1)	61.3 (48.9 – 72.8)§	64.0% (54.3 – 71.3)§	1.17 (1.07 – 1.27)‡	1.21 (1.10 – 1.32)‡	1.21 (1.09 – 1.34)‡	0.04 (-0.10 – 0.19)
	3rd	87.8 (80.0 – 95.7)	54.2 (48.6 – 60.2)	29.2 (23.7 – 36.0)	58.6 (49.2 – 67.4)§	66.8% (58.6 – 73.1)§	1.07 (1.00 – 1.14)‡	1.04 (0.97 – 1.12)	1.02 (0.95 – 1.10)	-0.05 (-0.13 – 0.03)
	4th	75.1 (66.1 – 84.9)	45.5 (39.3 – 52.2)	24.1 (19.0 – 30.5)	51.0 (40.8 – 61.1)§	67.9% (58.9 – 75.0)§	0.92 (0.82 – 1.02)	0.88 (0.78 – 0.98)‡	0.85 (0.74 – 0.96)‡	-0.07 (-0.20 – 0.05)
	5th	57.2 (46.5 – 69.3)	34.8 (28.0 – 42.6)	18.7 (13.9 – 24.8)	38.5 (28.5 – 49.2)§	67.4% (57.1 – 75.1)§	0.70 (0.57 – 0.84)‡	0.67 (0.55 – 0.81)‡	0.65 (0.53 – 0.80)‡	-0.04 (-0.17 – 0.08)

Continued on next page

‡: Ratio is significantly different from one.
§: Change/decline is significantly different from zero.
Absolute decline: wealth quintile-specific U5MR (1990) - wealth quintile-specific U5MR (2016).
Percentage decline: absolute decline over wealth quintile-specific U5MR (1990) $\times 100$.
Change in ratio: ratio (2016) - ratio (1990).

Table 8 – continued from previous page

	Wealth Quintile	wealth quintile-specific U5MR (deaths per 1000 livebirths)					Ratio of wealth quintile-specific to national-level U5MR			
		1990	2000	2016	Absolute decline 1990–2016	Percentage decline 1990–2016	1990	2000	2016	Change 1990–2016
	1st	272.9 (240.1 – 308.6)	196.8 (176.8 – 217.6)	115.0 (90.6 – 146.8)	157.9 (114.3 – 199.0)§	57.8% (45.1 – 67.5)§	1.16 (1.04 – 1.29)‡	1.19 (1.10 – 1.28)‡	1.29 (1.15 – 1.44)‡	0.13 (-0.05 – 0.31)
	2nd	264.7 (237.5 – 295.6)	185.9 (168.3 – 204.8)	100.0 (79.6 – 127.2)	164.7 (128.5 – 201.0)§	62.2% (51.4 – 70.5)§	1.13 (1.03 – 1.23)‡	1.12 (1.04 – 1.20)‡	1.12 (1.03 – 1.23)‡	0.00 (-0.14 – 0.13)
Guinea	3rd	245.9 (225.4 – 268.0)	172.7 (157.8 – 187.9)	92.2 (73.9 – 115.8)	153.7 (125.0 – 180.7)§	62.5% (52.7 – 70.2)§	1.05 (0.99 – 1.11)	1.04 (0.99 – 1.09)	1.04 (0.97 – 1.10)	-0.01 (-0.09 – 0.06)
	4th	227.9 (201.6 – 255.0)	161.5 (145.4 – 178.8)	79.9 (62.8 – 101.5)	148.0 (115.1 – 179.1)§	64.9% (54.4 – 72.8)§	0.97 (0.87 – 1.07)	0.97 (0.90 – 1.05)	0.90 (0.80 – 1.00)‡	-0.07 (-0.20 – 0.06)
	5th	162.6 (140.4 – 188.9)	112.1 (99.1 – 127.2)	57.9 (44.7 – 75.6)	104.7 (78.9 – 132.3)§	64.4% (53.0 – 73.0)§	0.69 (0.61 – 0.79)‡	0.68 (0.61 – 0.75)‡	0.65 (0.56 – 0.75)‡	-0.04 (-0.16 – 0.07)
	1st	241.0 (204.1 – 282.0)	192.6 (166.3 – 221.2)	104.8 (70.4 – 147.4)	136.2 (82.1 – 187.6)§	56.5% (37.1 – 71.2)§	1.10 (0.97 – 1.23)	1.11 (1.00 – 1.22)	1.19 (1.03 – 1.35)‡	0.09 (-0.09 – 0.26)
	2nd	254.3 (220.3 – 292.2)	204.6 (179.9 – 232.4)	101.7 (68.6 – 143.3)	152.6 (99.6 – 202.6)§	60.0% (42.3 – 73.3)§	1.16 (1.06 – 1.27)‡	1.17 (1.09 – 1.26)‡	1.15 (1.05 – 1.26)‡	-0.01 (-0.14 – 0.13)
Guinea-Bissau	3rd	232.0 (204.5 – 261.7)	184.1 (163.4 – 207.0)	92.5 (63.2 – 129.5)	139.5 (94.5 – 181.0)§	60.1% (43.0 – 73.1)§	1.06 (0.99 – 1.12)	1.06 (1.00 – 1.12)	1.05 (0.98 – 1.12)	-0.01 (-0.08 – 0.07)
	4th	213.6 (182.5 – 246.9)	164.7 (142.9 – 189.0)	78.5 (53.1 – 111.2)	135.1 (91.3 – 176.6)§	63.3% (46.8 – 75.3)§	0.97 (0.87 – 1.08)	0.95 (0.86 – 1.03)	0.89 (0.79 – 0.99)‡	-0.08 (-0.21 – 0.05)
	5th	155.1 (129.1 – 184.8)	124.9 (107.1 – 145.5)	63.1 (42.4 – 91.2)	92.1 (57.6 – 126.4)§	59.4% (40.4 – 73.0)§	0.71 (0.61 – 0.81)‡	0.72 (0.64 – 0.81)‡	0.72 (0.61 – 0.83)‡	0.01 (-0.11 – 0.13)
	1st	68.4 (56.1 – 81.8)	53.0 (43.8 – 63.0)	37.9 (24.6 – 58.4)	30.4 (9.2 – 47.6)§	44.5% (14.2 – 64.3)§	1.14 (0.96 – 1.32)	1.14 (0.97 – 1.32)	1.17 (0.98 – 1.36)	0.03 (-0.15 – 0.22)
	2nd	71.0 (61.0 – 81.6)	55.3 (47.4 – 63.6)	38.5 (25.7 – 57.6)	32.5 (11.2 – 48.6)§	45.8% (16.7 – 64.2)§	1.18 (1.05 – 1.31)‡	1.19 (1.07 – 1.32)‡	1.19 (1.05 – 1.32)‡	0.01 (-0.14 – 0.16)
Guyana	3rd	62.4 (55.0 – 70.6)	47.9 (41.9 – 54.2)	33.1 (22.4 – 49.4)	29.3 (11.7 – 42.4)§	47.0% (19.8 – 64.7)§	1.04 (0.96 – 1.12)	1.03 (0.95 – 1.11)	1.02 (0.94 – 1.11)	-0.02 (-0.09 – 0.06)
	4th	52.8 (44.7 – 62.3)	40.3 (34.0 – 47.4)	27.8 (18.5 – 42.2)	25.0 (9.3 – 37.7)§	47.3% (18.9 – 65.6)§	0.88 (0.77 – 0.99)‡	0.87 (0.76 – 0.98)‡	0.86 (0.74 – 0.98)‡	-0.02 (-0.15 – 0.12)
	5th	45.8 (35.5 – 59.3)	35.5 (27.3 – 46.0)	24.6 (15.6 – 39.4)	21.2 (6.7 – 34.3)§	46.2% (15.7 – 65.3)§	0.76 (0.60 – 0.97)‡	0.76 (0.60 – 0.97)‡	0.76 (0.59 – 0.97)‡	0.00 (-0.14 – 0.14)
	1st	158.0 (140.2 – 177.7)	120.4 (106.5 – 135.7)	79.4 (59.8 – 107.4)	78.6 (48.6 – 104.5)§	49.7% (32.1 – 62.6)§	1.09 (0.99 – 1.20)	1.15 (1.05 – 1.26)‡	1.19 (1.04 – 1.33)‡	0.09 (-0.07 – 0.26)
	2nd	164.4 (147.7 – 182.4)	119.4 (106.6 – 132.9)	77.8 (59.0 – 103.7)	86.6 (58.2 – 110.4)§	52.7% (36.9 – 64.3)§	1.13 (1.04 – 1.23)‡	1.14 (1.05 – 1.24)‡	1.16 (1.05 – 1.28)‡	0.03 (-0.11 – 0.16)
Haiti	3rd	154.5 (141.5 – 168.3)	111.1 (100.8 – 122.1)	69.6 (53.3 – 91.6)	84.8 (61.3 – 104.3)§	54.9% (40.7 – 65.5)§	1.07 (1.01 – 1.13)‡	1.06 (1.00 – 1.12)‡	1.04 (0.97 – 1.10)	-0.03 (-0.10 – 0.04)
	4th	140.5 (124.8 – 157.5)	95.9 (84.4 – 108.2)	60.6 (45.6 – 81.8)	79.9 (55.3 – 101.0)§	56.9% (41.2 – 67.8)§	0.97 (0.87 – 1.07)	0.92 (0.83 – 1.01)	0.90 (0.80 – 1.02)	-0.06 (-0.19 – 0.07)
	5th	107.0 (91.5 – 124.5)	75.8 (64.6 – 88.0)	47.5 (35.3 – 65.0)	59.6 (39.3 – 78.4)§	55.6% (39.2 – 67.2)§	0.74 (0.64 – 0.85)‡	0.73 (0.63 – 0.83)‡	0.71 (0.60 – 0.83)‡	-0.03 (-0.15 – 0.09)
	1st	79.9 (68.2 – 92.5)	53.5 (46.6 – 61.3)	27.3 (19.6 – 37.5)	52.6 (38.4 – 66.2)§	65.8% (52.5 – 75.5)§	1.37 (1.20 – 1.56)‡	1.43 (1.28 – 1.60)‡	1.46 (1.26 – 1.67)‡	0.09 (-0.14 – 0.32)
	2nd	67.0 (58.9 – 76.3)	42.8 (37.7 – 48.3)	21.1 (15.2 – 28.7)	45.9 (35.5 – 56.0)§	68.5% (56.7 – 77.3)§	1.15 (1.03 – 1.27)‡	1.15 (1.04 – 1.26)‡	1.13 (1.00 – 1.27)	-0.02 (-0.19 – 0.14)
Honduras	3rd	59.0 (52.9 – 65.4)	37.2 (33.3 – 41.4)	18.6 (13.6 – 25.1)	40.4 (32.6 – 47.9)§	68.5% (57.5 – 76.8)§	1.02 (0.94 – 1.09)	1.00 (0.92 – 1.07)	0.99 (0.90 – 1.08)	-0.02 (-0.11 – 0.07)
	4th	49.1 (42.1 – 56.7)	30.6 (26.3 – 35.3)	15.0 (10.7 – 20.7)	34.1 (25.7 – 42.4)§	69.5% (57.2 – 78.5)§	0.85 (0.74 – 0.96)‡	0.82 (0.72 – 0.92)‡	0.80 (0.68 – 0.93)‡	-0.04 (-0.19 – 0.11)
	5th	35.5 (27.8 – 44.4)	22.3 (17.8 – 27.7)	11.5 (7.8 – 16.5)	24.0 (16.6 – 32.2)§	67.7% (54.0 – 77.6)§	0.61 (0.49 – 0.75)‡	0.60 (0.49 – 0.73)‡	0.61 (0.49 – 0.76)‡	0.00 (-0.12 – 0.13)
	1st	169.7 (160.0 – 179.5)	125.8 (117.0 – 134.5)	61.2 (52.2 – 70.7)	108.5 (95.0 – 121.9)§	63.9% (57.7 – 69.6)§	1.35 (1.29 – 1.41)‡	1.37 (1.29 – 1.46)‡	1.42 (1.26 – 1.59)‡	0.07 (-0.10 – 0.25)
	2nd	160.3 (150.8 – 170.1)	114.8 (106.7 – 123.0)	52.8 (45.7 – 60.4)	107.5 (95.1 – 119.5)§	67.0% (61.7 – 71.9)§	1.27 (1.21 – 1.34)‡	1.25 (1.18 – 1.33)‡	1.23 (1.11 – 1.35)‡	-0.04 (-0.18 – 0.09)
India	3rd	132.8 (125.7 – 140.3)	96.6 (90.7 – 102.7)	44.0 (38.7 – 49.2)	88.8 (80.4 – 97.4)§	66.9% (62.5 – 71.0)§	1.05 (1.01 – 1.10)‡	1.06 (1.00 – 1.11)‡	1.02 (0.95 – 1.09)	-0.03 (-0.11 – 0.04)
	4th	105.1 (98.4 – 112.2)	74.0 (68.4 – 80.2)	35.0 (29.7 – 40.7)	70.1 (61.3 – 78.9)§	66.7% (60.6 – 72.1)§	0.83 (0.79 – 0.88)‡	0.81 (0.75 – 0.87)‡	0.81 (0.71 – 0.92)‡	-0.02 (-0.13 – 0.09)
	5th	61.6 (57.2 – 66.3)	46.3 (42.3 – 50.7)	22.0 (18.2 – 26.9)	39.6 (33.4 – 45.6)§	64.2% (56.2 – 70.9)§	0.49 (0.46 – 0.52)‡	0.51 (0.47 – 0.55)‡	0.51 (0.44 – 0.61)‡	0.02 (-0.06 – 0.12)
	1st	119.2 (106.5 – 133.6)	77.1 (69.7 – 85.5)	39.3 (30.3 – 50.9)	79.9 (64.2 – 96.0)§	67.0% (57.0 – 74.8)§	1.41 (1.28 – 1.57)‡	1.47 (1.35 – 1.61)‡	1.49 (1.32 – 1.67)‡	0.08 (-0.12 – 0.27)
	2nd	94.4 (84.8 – 103.9)	59.7 (53.6 – 65.7)	29.8 (23.0 – 38.2)	64.6 (52.8 – 75.5)§	68.4% (59.0 – 75.8)§	1.12 (1.02 – 1.22)‡	1.14 (1.04 – 1.24)‡	1.13 (1.01 – 1.25)‡	0.01 (-0.13 – 0.16)
Indonesia	3rd	87.2 (80.6 – 94.1)	52.7 (48.3 – 57.1)	26.2 (20.5 – 33.4)	61.0 (51.6 – 69.3)§	69.9% (61.6 – 76.5)§	1.03 (0.97 – 1.10)	1.01 (0.94 – 1.07)	0.99 (0.92 – 1.07)	-0.04 (-0.12 – 0.04)
	4th	73.4 (64.8 – 82.3)	43.6 (38.6 – 49.0)	21.8 (16.6 – 28.3)	51.6 (41.5 – 61.4)§	70.3% (61.0 – 77.6)§	0.87 (0.78 – 0.97)‡	0.83 (0.75 – 0.92)‡	0.82 (0.72 – 0.94)‡	-0.05 (-0.18 – 0.08)
	5th	47.2 (39.3 – 56.5)	28.3 (23.8 – 33.5)	14.8 (11.0 – 19.9)	32.4 (24.5 – 41.3)§	68.6% (57.8 – 76.8)§	0.56 (0.47 – 0.67)‡	0.54 (0.46 – 0.64)‡	0.56 (0.46 – 0.67)‡	0.00 (-0.11 – 0.11)
	1st	63.0 (52.5 – 74.1)	52.2 (44.4 – 61.1)	36.8 (26.5 – 51.0)	26.3 (10.1 – 40.2)§	41.7% (17.6 – 58.2)§	1.16 (1.00 – 1.34)	1.16 (1.01 – 1.32)‡	1.18 (1.03 – 1.34)‡	0.02 (-0.17 – 0.20)
	2nd	61.3 (53.1 – 70.0)	50.6 (43.9 – 57.7)	34.8 (25.4 – 47.9)	26.5 (12.2 – 38.4)§	43.2% (21.0 – 59.0)§	1.13 (1.01 – 1.25)‡	1.13 (1.01 – 1.24)‡	1.12 (1.00 – 1.24)‡	-0.02 (-0.16 – 0.12)
Iraq	3rd	54.0 (47.7 – 60.4)	44.5 (39.3 – 49.8)	30.6 (22.3 – 41.7)	23.4 (11.6 – 32.6)§	43.3% (22.2 – 58.2)§	1.00 (0.92 – 1.07)	0.99 (0.91 – 1.06)	0.98 (0.91 – 1.05)	-0.01 (-0.09 – 0.06)
	4th	46.3 (39.5 – 54.1)	38.5 (32.8 – 44.7)	26.5 (19.0 – 36.7)	19.8 (8.2 – 29.5)§	42.7% (19.1 – 58.9)§	0.85 (0.75 – 0.97)‡	0.86 (0.75 – 0.96)‡	0.85 (0.75 – 0.97)‡	0.00 (-0.13 – 0.13)
	5th	46.3 (37.0 – 57.4)	39.2 (31.8 – 47.7)	27.2 (19.1 – 38.4)	19.1 (6.7 – 30.5)§	41.2% (15.8 – 58.2)§	0.85 (0.70 – 1.04)	0.87 (0.72 – 1.04)	0.87 (0.72 – 1.04)	0.02 (-0.14 – 0.18)
	1st	45.2 (39.4 – 51.6)	34.8 (30.0 – 40.1)	23.0 (15.7 – 32.8)	22.2 (11.4 – 31.2)§	49.1% (26.5 – 65.4)§	1.24 (1.10 – 1.39)‡	1.25 (1.10 – 1.40)‡	1.31 (1.11 – 1.51)‡	0.07 (-0.13 – 0.28)
	2nd	41.3 (36.6 – 46.3)	31.5 (27.5 – 35.7)	20.0 (14.1 – 28.2)	21.3 (12.1 – 28.9)§	51.7% (30.9 – 66.3)§	1.13 (1.02 – 1.24)‡	1.13 (1.01 – 1.24)‡	1.14 (1.00 – 1.28)	0.00 (-0.15 – 0.16)
Jordan	3rd	36.7 (33.3 – 40.6)	28.0 (25.0 – 31.1)	17.6 (12.5 – 24.5)	19.2 (11.8 – 25.3)§	52.2% (33.1 – 66.3)§	1.01 (0.94 – 1.08)	1.00 (0.93 – 1.08)	1.00 (0.91 – 1.08)	-0.01 (-0.09 – 0.07)
	4th	32.3 (28.3 – 36.8)	24.4 (21.0 – 28.2)	14.5 (9.9 – 20.8)	17.8 (10.6 – 24.0)§	55.0% (34.7 – 69.7)§	0.89 (0.79 – 0.99)‡	0.88 (0.77 – 0.99)‡	0.83 (0.69 – 0.96)‡	-0.06 (-0.22 – 0.09)
	5th	26.9 (22.5 – 32.1)	20.8 (17.2 – 25.0)	13.0 (8.8 – 18.8)	14.0 (7.7 – 19.8)§	51.9% (30.3 – 67.2)§	0.74 (0.62 – 0.87)‡	0.75 (0.63 – 0.88)‡	0.74 (0.59 – 0.90)‡	0.00 (-0.14 – 0.14)

Continued on next page

‡: Ratio is significantly different from one.
§: Change/decline is significantly different from zero.
Absolute decline: wealth quintile-specific U5MR (1990) - wealth quintile-specific U5MR (2016).
Percentage decline: absolute decline over wealth quintile-specific U5MR (1990) $\times 100$.
Change in ratio: ratio (2016) - ratio (1990).

Table 8 – continued from previous page

		wealth quintile-specific U5MR (deaths per 1000 livebirths)					Ratio of wealth quintile-specific to national-level U5MR			
	Wealth Quintile	1990	2000	2016	Absolute decline 1990–2016	Percentage decline 1990–2016	1990	2000	2016	Change 1990–2016
Kazakhstan	1st	63.6 (53.4 – 74.6)	52.9 (44.9 – 61.7)	14.9 (12.3 – 17.8)	48.7 (39.1 – 59.3)§	76.5% (71.5 – 80.8)§	1.22 (1.05 – 1.39)‡	1.23 (1.07 – 1.40)‡	1.31 (1.09 – 1.55)‡	0.09 (-0.12 – 0.32)
	2nd	61.9 (53.5 – 70.8)	51.2 (44.7 – 58.1)	13.3 (11.4 – 15.2)	48.7 (40.5 – 57.5)§	78.6% (74.5 – 82.2)§	1.19 (1.06 – 1.31)‡	1.19 (1.08 – 1.31)‡	1.16 (1.01 – 1.32)‡	-0.02 (-0.19 – 0.15)
	3rd	53.6 (47.6 – 60.2)	44.0 (39.2 – 49.1)	11.5 (10.2 – 12.8)	42.1 (36.2 – 48.5)§	78.5% (75.5 – 81.3)§	1.03 (0.95 – 1.10)	1.02 (0.95 – 1.10)	1.01 (0.91 – 1.11)	-0.02 (-0.11 – 0.07)
	4th	45.1 (38.4 – 52.2)	36.5 (31.2 – 42.2)	9.2 (7.5 – 10.9)	35.9 (29.5 – 42.9)§	79.6% (74.8 – 83.7)§	0.86 (0.76 – 0.97)‡	0.85 (0.74 – 0.96)‡	0.81 (0.66 – 0.95)‡	-0.06 (-0.22 – 0.10)
	5th	36.8 (28.8 – 46.6)	30.4 (24.1 – 37.8)	8.1 (6.2 – 10.3)	28.7 (21.7 – 37.6)§	78.0% (72.6 – 82.5)§	0.70 (0.56 – 0.87)‡	0.71 (0.57 – 0.87)‡	0.71 (0.55 – 0.90)‡	0.00 (-0.14 – 0.15)
Kenya	1st	125.4 (112.6 – 139.3)	125.5 (112.9 – 139.7)	60.4 (48.3 – 75.3)	65.0 (46.5 – 82.5)§	51.8% (39.0 – 61.9)§	1.28 (1.17 – 1.40)‡	1.25 (1.15 – 1.35)‡	1.23 (1.10 – 1.37)‡	-0.05 (-0.22 – 0.11)
	2nd	112.1 (101.2 – 123.2)	112.1 (100.7 – 124.3)	56.7 (45.7 – 70.0)	55.3 (39.2 – 70.2)§	49.4% (36.6 – 59.6)§	1.14 (1.06 – 1.23)‡	1.11 (1.03 – 1.20)‡	1.15 (1.05 – 1.26)‡	0.01 (-0.12 – 0.14)
	3rd	99.4 (91.3 – 107.5)	102.6 (93.7 – 111.8)	48.8 (40.0 – 59.7)	50.6 (37.6 – 62.0)§	50.9% (39.3 – 60.2)§	1.01 (0.96 – 1.07)	1.02 (0.96 – 1.07)	0.99 (0.93 – 1.06)	-0.02 (-0.09 – 0.05)
	4th	81.5 (72.2 – 90.8)	86.4 (77.1 – 96.3)	42.9 (34.3 – 53.7)	38.5 (24.7 – 50.5)§	47.3% (32.5 – 58.6)§	0.83 (0.75 – 0.91)‡	0.86 (0.78 – 0.93)‡	0.87 (0.78 – 0.97)‡	0.04 (-0.07 – 0.17)
	5th	72.1 (63.2 – 81.9)	77.4 (68.5 – 87.4)	37.1 (29.2 – 47.6)	35.0 (22.7 – 46.4)§	48.6% (33.3 – 60.0)§	0.74 (0.66 – 0.82)‡	0.77 (0.69 – 0.85)‡	0.75 (0.65 – 0.87)‡	0.02 (-0.10 – 0.14)
Kyrgyzstan	1st	82.0 (68.0 – 97.0)	63.3 (52.6 – 73.7)	28.2 (23.8 – 33.1)	53.7 (40.3 – 68.2)§	65.6% (57.7 – 71.7)§	1.25 (1.08 – 1.43)‡	1.28 (1.11 – 1.46)‡	1.34 (1.14 – 1.55)‡	0.08 (-0.12 – 0.30)
	2nd	75.1 (64.2 – 87.2)	57.6 (49.4 – 65.6)	24.2 (21.1 – 27.5)	50.9 (40.0 – 62.9)§	67.8% (61.2 – 73.1)§	1.15 (1.04 – 1.27)‡	1.17 (1.05 – 1.29)‡	1.15 (1.01 – 1.29)‡	0.00 (-0.16 – 0.16)
	3rd	66.9 (58.1 – 76.3)	49.8 (43.3 – 55.6)	21.1 (19.0 – 23.2)	45.8 (37.2 – 55.1)§	68.5% (63.2 – 72.8)§	1.02 (0.95 – 1.10)	1.01 (0.93 – 1.08)	1.00 (0.91 – 1.08)	-0.02 (-0.11 – 0.06)
	4th	56.3 (47.2 – 66.7)	41.4 (34.8 – 48.0)	17.1 (14.4 – 20.0)	39.2 (30.1 – 49.6)§	69.6% (62.4 – 75.6)§	0.86 (0.75 – 0.98)‡	0.84 (0.73 – 0.95)‡	0.81 (0.69 – 0.94)‡	-0.05 (-0.19 – 0.09)
	5th	46.8 (36.3 – 59.3)	34.9 (27.4 – 43.6)	14.9 (11.7 – 18.7)	31.9 (23.1 – 42.9)§	68.2% (60.2 – 74.6)§	0.72 (0.57 – 0.88)‡	0.71 (0.57 – 0.87)‡	0.70 (0.56 – 0.88)‡	-0.01 (-0.15 – 0.13)
Lao People’s Democratic Republic	1st	196.1 (162.2 – 235.0)	151.2 (125.6 – 181.3)	87.1 (60.2 – 123.6)	109.1 (63.9 – 150.0)§	55.6% (36.1 – 69.3)§	1.21 (1.03 – 1.41)‡	1.30 (1.12 – 1.49)‡	1.36 (1.17 – 1.56)‡	0.15 (-0.05 – 0.34)
	2nd	189.2 (163.1 – 217.5)	136.9 (117.7 – 158.9)	76.9 (54.0 – 107.5)	112.3 (74.7 – 147.7)§	59.4% (42.4 – 71.7)§	1.17 (1.05 – 1.29)‡	1.18 (1.05 – 1.30)‡	1.20 (1.08 – 1.33)‡	0.03 (-0.12 – 0.18)
	3rd	176.2 (155.6 – 198.7)	126.9 (111.6 – 144.4)	67.6 (48.1 – 94.1)	108.6 (77.8 – 136.9)§	61.6% (46.2 – 73.0)§	1.09 (1.01 – 1.17)‡	1.09 (1.01 – 1.16)‡	1.06 (0.98 – 1.13)	-0.03 (-0.11 – 0.05)
	4th	161.9 (137.3 – 188.8)	108.3 (91.6 – 127.6)	56.5 (39.5 – 80.1)	105.4 (74.3 – 135.9)§	65.1% (49.8 – 76.1)§	1.00 (0.88 – 1.13)	0.93 (0.82 – 1.05)	0.88 (0.78 – 1.00)‡	-0.12 (-0.25 – 0.02)
	5th	84.5 (63.7 – 109.0)	59.1 (45.4 – 76.1)	31.4 (20.5 – 46.9)	53.1 (33.2 – 75.0)§	62.8% (45.5 – 74.9)§	0.52 (0.40 – 0.67)‡	0.51 (0.40 – 0.63)‡	0.49 (0.38 – 0.62)‡	-0.03 (-0.13 – 0.06)
Lesotho	1st	102.6 (87.0 – 120.0)	119.5 (103.7 – 136.3)	103.4 (77.2 – 138.1)	-0.8 (-36.3 – 28.3)	-0.8% (-37.6 – 25.8)	1.12 (0.99 – 1.27)	1.08 (0.96 – 1.21)	1.11 (0.97 – 1.26)	-0.02 (-0.18 – 0.14)
	2nd	103.0 (89.9 – 117.2)	123.3 (108.8 – 138.5)	105.4 (79.3 – 138.3)	-2.4 (-37.2 – 25.7)	-2.3% (-38.0 – 23.5)	1.13 (1.02 – 1.24)‡	1.12 (1.02 – 1.22)‡	1.13 (1.02 – 1.23)‡	0.00 (-0.14 – 0.13)
	3rd	96.6 (85.7 – 107.7)	117.5 (105.6 – 129.7)	98.8 (74.9 – 128.3)	-2.2 (-33.0 – 22.8)	-2.3% (-35.4 – 22.6)	1.06 (0.99 – 1.13)	1.06 (1.00 – 1.13)	1.06 (0.99 – 1.13)	0.00 (-0.07 – 0.07)
	4th	83.9 (71.6 – 97.2)	105.2 (92.1 – 120.0)	88.7 (66.7 – 117.6)	-4.9 (-34.9 – 19.4)	-5.8% (-44.0 – 21.9)	0.92 (0.82 – 1.03)	0.95 (0.86 – 1.06)	0.95 (0.85 – 1.06)	0.03 (-0.10 – 0.16)
	5th	70.4 (57.9 – 84.7)	86.1 (73.0 – 101.6)	71.1 (52.0 – 96.4)	-0.7 (-26.3 – 20.1)	-1.0% (-39.8 – 26.2)	0.77 (0.65 – 0.90)‡	0.78 (0.68 – 0.90)‡	0.76 (0.64 – 0.90)‡	-0.01 (-0.14 – 0.12)
Liberia	1st	260.1 (219.0 – 307.0)	186.9 (159.8 – 215.1)	78.0 (56.4 – 109.8)	182.1 (134.7 – 231.5)§	70.0% (57.2 – 78.7)§	1.01 (0.87 – 1.16)	1.02 (0.89 – 1.15)	1.16 (1.01 – 1.31)‡	0.15 (-0.03 – 0.32)
	2nd	276.4 (240.5 – 313.9)	192.9 (170.3 – 216.6)	73.9 (54.1 – 102.9)	202.5 (160.1 – 243.4)§	73.3% (62.5 – 80.6)§	1.07 (0.96 – 1.18)	1.05 (0.95 – 1.14)	1.10 (0.99 – 1.21)	0.02 (-0.11 – 0.16)
	3rd	264.6 (236.9 – 294.2)	188.5 (170.0 – 207.4)	67.6 (49.9 – 93.5)	197.0 (162.6 – 229.1)§	74.4% (64.6 – 81.3)§	1.03 (0.96 – 1.10)	1.02 (0.96 – 1.09)	1.00 (0.93 – 1.07)	-0.02 (-0.10 – 0.05)
	4th	256.8 (221.0 – 295.8)	182.9 (160.2 – 207.8)	58.6 (43.0 – 82.0)	198.2 (158.5 – 237.7)§	77.2% (67.7 – 83.6)§	1.00 (0.88 – 1.11)	0.99 (0.89 – 1.10)	0.87 (0.77 – 0.97)‡	-0.13 (-0.26 – 0.01)
	5th	231.6 (192.8 – 276.9)	168.3 (144.5 – 195.3)	58.9 (42.0 – 83.3)	172.7 (131.2 – 215.9)§	74.6% (63.9 – 81.9)§	0.90 (0.76 – 1.06)	0.92 (0.80 – 1.04)	0.87 (0.75 – 1.02)	-0.02 (-0.18 – 0.13)
Madagascar	1st	187.0 (164.9 – 210.5)	135.2 (119.6 – 152.7)	62.2 (42.2 – 90.8)	124.8 (90.1 – 154.1)§	66.7% (50.6 – 77.6)§	1.17 (1.06 – 1.29)‡	1.26 (1.15 – 1.37)‡	1.34 (1.17 – 1.52)‡	0.17 (-0.02 – 0.36)
	2nd	185.7 (166.4 – 206.3)	125.5 (111.6 – 140.1)	55.3 (37.7 – 79.8)	130.3 (99.8 – 156.3)§	70.2% (56.7 – 79.7)§	1.16 (1.07 – 1.26)‡	1.17 (1.08 – 1.26)‡	1.19 (1.07 – 1.32)‡	0.03 (-0.12 – 0.17)
	3rd	170.0 (155.4 – 185.4)	114.5 (103.3 – 126.2)	47.5 (32.6 – 68.5)	122.5 (97.5 – 143.1)§	72.0% (59.4 – 80.9)§	1.07 (1.01 – 1.13)‡	1.06 (1.00 – 1.12)‡	1.02 (0.95 – 1.10)	-0.04 (-0.12 – 0.04)
	4th	158.3 (141.0 – 177.3)	98.4 (87.0 – 111.1)	39.8 (26.9 – 57.8)	118.5 (93.8 – 140.4)§	74.9% (62.8 – 83.1)§	0.99 (0.90 – 1.09)	0.91 (0.83 – 1.00)	0.86 (0.75 – 0.96)‡	-0.14 (-0.26 – 0.00)§
	5th	96.5 (81.8 – 113.5)	64.3 (54.4 – 75.7)	27.2 (17.8 – 40.7)	69.4 (51.5 – 86.8)§	71.8% (57.9 – 81.5)§	0.61 (0.52 – 0.70)‡	0.60 (0.52 – 0.69)‡	0.59 (0.48 – 0.71)‡	-0.02 (-0.13 – 0.09)
Malawi	1st	242.4 (218.5 – 267.4)	185.3 (170.1 – 201.5)	65.2 (49.2 – 85.5)	177.1 (146.7 – 205.9)§	73.1% (64.4 – 79.9)§	1.04 (0.96 – 1.13)	1.06 (0.99 – 1.13)	1.18 (1.06 – 1.32)‡	0.14 (-0.01 – 0.30)
	2nd	254.8 (231.5 – 278.4)	193.9 (178.0 – 211.0)	64.1 (49.0 – 83.7)	190.7 (160.8 – 218.3)§	74.8% (66.8 – 81.0)§	1.10 (1.02 – 1.18)‡	1.11 (1.05 – 1.18)‡	1.16 (1.06 – 1.27)‡	0.07 (-0.06 – 0.20)
	3rd	248.0 (230.2 – 266.4)	184.6 (171.7 – 198.4)	56.3 (43.4 – 72.4)	191.7 (168.3 – 213.9)§	77.3% (70.7 – 82.6)§	1.07 (1.02 – 1.12)‡	1.06 (1.01 – 1.10)‡	1.02 (0.96 – 1.08)	-0.05 (-0.12 – 0.03)
	4th	241.7 (218.7 – 265.1)	177.4 (162.9 – 193.2)	47.9 (36.0 – 62.9)	193.7 (167.3 – 219.2)§	80.2% (73.7 – 85.2)§	1.04 (0.96 – 1.12)	1.02 (0.95 – 1.08)	0.87 (0.78 – 0.97)‡	-0.17 (-0.29 – -0.05)§
	5th	175.2 (156.3 – 195.8)	132.2 (120.1 – 145.5)	41.9 (31.4 – 55.5)	133.3 (110.8 – 155.6)§	76.1% (67.8 – 82.4)§	0.75 (0.68 – 0.83)‡	0.76 (0.70 – 0.81)‡	0.76 (0.67 – 0.86)‡	0.01 (-0.11 – 0.12)
Maldives	1st	113.6 (92.9 – 136.9)	55.8 (45.5 – 67.2)	11.5 (8.4 – 15.3)	102.1 (81.6 – 124.6)§	89.9% (86.1 – 92.6)§	1.21 (1.01 – 1.43)‡	1.28 (1.07 – 1.50)‡	1.35 (1.11 – 1.61)‡	0.14 (-0.08 – 0.38)
	2nd	107.3 (92.2 – 124.2)	51.3 (43.7 – 59.5)	9.9 (7.4 – 12.9)	97.4 (82.1 – 114.0)§	90.8% (87.6 – 93.2)§	1.14 (1.01 – 1.27)‡	1.18 (1.04 – 1.32)‡	1.16 (1.00 – 1.33)	0.02 (-0.15 – 0.20)
	3rd	99.1 (87.2 – 111.4)	44.1 (38.4 – 50.1)	8.5 (6.6 – 11.0)	90.5 (78.2 – 103.0)§	91.4% (88.6 – 93.4)§	1.06 (0.97 – 1.13)	1.01 (0.93 – 1.10)	1.00 (0.90 – 1.10)	-0.05 (-0.14 – 0.05)
	4th	84.3 (71.2 – 98.6)	37.1 (31.1 – 43.8)	6.9 (5.0 – 9.2)	77.4 (64.3 – 91.9)§	91.9% (88.8 – 94.2)§	0.90 (0.78 – 1.02)	0.85 (0.74 – 0.97)‡	0.81 (0.66 – 0.96)‡	-0.09 (-0.25 – 0.07)
	5th	65.2 (48.3 – 87.4)	29.1 (21.6 – 39.0)	5.7 (3.9 – 8.2)	59.5 (43.3 – 80.6)§	91.2% (87.8 – 93.7)§	0.69 (0.52 – 0.92)‡	0.67 (0.50 – 0.88)‡	0.67 (0.49 – 0.90)‡	-0.02 (-0.16 – 0.12)

Continued on next page

‡: Ratio is significantly different from one.
§: Change/decline is significantly different from zero.
Absolute decline: wealth quintile-specific U5MR (1990) - wealth quintile-specific U5MR (2016).
Percentage decline: absolute decline over wealth quintile-specific U5MR (1990) ×100.
Change in ratio: ratio (2016) - ratio (1990).

Table 8 – continued from previous page

	Wealth Quintile	wealth quintile-specific U5MR (deaths per 1000 livebirths) 1990	2000	2016	Absolute decline 1990–2016	Percentage decline 1990–2016	Ratio of wealth quintile-specific to national-level U5MR 1990	2000	2016	Change 1990–2016
	1st	278.8 (252.0 – 308.2)	237.3 (214.4 – 261.8)	132.8 (80.8 – 212.2)	146.0 (63.9 – 202.8)§	52.4% (23.7 – 71.1)§	1.10 (1.02 – 1.18)‡	1.08 (1.01 – 1.14)‡	1.20 (1.07 – 1.34)‡	0.10 (-0.05 – 0.26)
	2nd	286.2 (261.1 – 313.6)	253.3 (230.4 – 278.5)	127.9 (78.6 – 203.5)	158.3 (82.0 – 212.6)§	55.3% (29.1 – 72.6)§	1.13 (1.05 – 1.20)‡	1.15 (1.09 – 1.22)‡	1.16 (1.06 – 1.26)‡	0.03 (-0.09 – 0.16)
Mali	3rd	272.8 (251.9 – 295.5)	238.1 (218.4 – 259.8)	119.7 (73.6 – 189.3)	153.0 (84.1 – 202.4)§	56.1% (30.8 – 72.9)§	1.07 (1.03 – 1.12)‡	1.08 (1.04 – 1.13)‡	1.08 (1.02 – 1.14)‡	0.01 (-0.06 – 0.08)
	4th	260.9 (237.1 – 286.5)	230.1 (208.0 – 253.8)	104.3 (64.0 – 166.3)	156.7 (92.7 – 201.7)§	60.0% (36.2 – 75.4)§	1.03 (0.96 – 1.10)	1.05 (0.98 – 1.11)	0.94 (0.85 – 1.05)	-0.09 (-0.20 – 0.04)
	5th	170.8 (152.1 – 191.3)	140.2 (125.6 – 156.3)	68.3 (41.5 – 109.8)	102.4 (59.6 – 135.4)§	60.0% (35.8 – 75.8)§	0.67 (0.61 – 0.74)‡	0.64 (0.59 – 0.69)‡	0.62 (0.53 – 0.71)‡	-0.05 (-0.16 – 0.05)
	1st	146.0 (122.5 – 171.8)	144.1 (121.2 – 169.6)	104.3 (56.9 – 190.9)	41.8 (-45.5 – 94.0)	28.6% (-32.4 – 61.3)	1.25 (1.09 – 1.43)‡	1.27 (1.13 – 1.43)‡	1.28 (1.12 – 1.45)‡	0.03 (-0.16 – 0.21)
	2nd	128.9 (110.4 – 148.6)	124.6 (107.0 – 144.5)	91.7 (50.2 – 168.0)	37.2 (-39.3 – 81.3)	28.8% (-31.9 – 60.9)	1.11 (0.99 – 1.22)	1.10 (1.00 – 1.21)	1.13 (1.02 – 1.24)‡	0.02 (-0.12 – 0.16)
Mauritania	3rd	120.4 (105.9 – 135.9)	116.5 (101.6 – 133.2)	82.9 (45.6 – 149.9)	37.5 (-30.3 – 76.6)	31.2% (-25.5 – 62.0)	1.03 (0.96 – 1.10)	1.03 (0.96 – 1.10)	1.02 (0.95 – 1.09)	-0.02 (-0.09 – 0.06)
	4th	103.1 (87.3 – 120.0)	98.7 (83.6 – 116.2)	69.4 (37.9 – 126.9)	33.7 (-25.2 – 68.8)	32.7% (-25.5 – 63.5)	0.89 (0.78 – 0.99)‡	0.87 (0.78 – 0.97)‡	0.85 (0.75 – 0.96)‡	-0.03 (-0.16 – 0.09)
	5th	84.0 (66.9 – 103.5)	81.6 (66.1 – 100.1)	58.6 (31.6 – 109.6)	25.4 (-25.0 – 56.2)	30.2% (-32.2 – 62.6)	0.72 (0.59 – 0.87)‡	0.72 (0.61 – 0.85)‡	0.72 (0.60 – 0.86)‡	0.00 (-0.13 – 0.13)
	1st	138.2 (118.2 – 159.9)	84.9 (72.9 – 97.9)	26.0 (17.1 – 39.0)	112.2 (89.0 – 134.7)§	81.2% (71.4 – 87.7)§	1.27 (1.12 – 1.43)‡	1.34 (1.19 – 1.49)‡	1.45 (1.25 – 1.67)‡	0.18 (-0.03 – 0.41)
	2nd	124.7 (109.0 – 141.5)	75.1 (65.4 – 85.6)	20.8 (13.6 – 31.0)	103.9 (85.5 – 121.8)§	83.3% (74.7 – 89.0)§	1.15 (1.04 – 1.26)‡	1.18 (1.08 – 1.29)‡	1.16 (1.02 – 1.31)‡	0.01 (-0.15 – 0.18)
Mongolia	3rd	113.6 (101.2 – 126.4)	64.6 (56.9 – 72.5)	17.7 (11.8 – 26.1)	95.8 (81.2 – 109.4)§	84.4% (76.7 – 89.7)§	1.05 (0.97 – 1.12)	1.02 (0.95 – 1.09)	0.99 (0.90 – 1.08)	-0.06 (-0.15 – 0.03)
	4th	94.1 (80.9 – 108.8)	52.5 (44.9 – 60.9)	13.9 (8.9 – 20.8)	80.2 (65.6 – 95.1)§	85.3% (77.7 – 90.6)§	0.87 (0.77 – 0.97)‡	0.83 (0.73 – 0.93)‡	0.77 (0.65 – 0.90)‡	-0.09 (-0.24 – 0.05)
	5th	71.9 (58.1 – 87.9)	40.0 (32.2 – 48.8)	11.1 (6.9 – 17.1)	60.9 (46.7 – 75.8)§	84.6% (76.2 – 90.2)§	0.66 (0.54 – 0.80)‡	0.63 (0.52 – 0.75)‡	0.62 (0.49 – 0.77)‡	-0.04 (-0.17 – 0.09)
	1st	106.1 (92.8 – 120.4)	69.4 (60.0 – 79.5)	38.5 (27.0 – 53.7)	67.6 (48.6 – 84.8)§	63.7% (48.7 – 74.7)§	1.33 (1.18 – 1.49)‡	1.39 (1.25 – 1.54)‡	1.42 (1.22 – 1.62)‡	0.09 (-0.11 – 0.29)
	2nd	95.3 (84.8 – 106.6)	61.5 (53.9 – 69.7)	32.3 (23.2 – 44.7)	62.9 (47.3 – 77.3)§	66.1% (52.4 – 75.9)§	1.20 (1.09 – 1.31)‡	1.23 (1.13 – 1.35)‡	1.19 (1.06 – 1.33)‡	0.00 (-0.15 – 0.14)
Morocco	3rd	84.1 (76.2 – 92.2)	51.0 (45.3 – 57.2)	27.5 (19.9 – 37.8)	56.5 (43.7 – 67.5)§	67.2% (54.4 – 76.5)§	1.06 (0.98 – 1.12)	1.02 (0.95 – 1.09)	1.02 (0.93 – 1.10)	-0.04 (-0.12 – 0.04)
	4th	70.4 (61.5 – 79.9)	41.3 (35.4 – 47.9)	22.6 (16.0 – 31.8)	47.8 (35.4 – 59.1)§	67.9% (54.2 – 77.6)§	0.88 (0.79 – 0.99)‡	0.83 (0.73 – 0.93)‡	0.83 (0.72 – 0.96)‡	-0.05 (-0.18 – 0.09)
	5th	42.2 (34.6 – 50.8)	25.9 (20.8 – 31.6)	14.6 (9.9 – 21.2)	27.6 (19.0 – 36.1)§	65.4% (49.7 – 76.2)§	0.53 (0.44 – 0.63)‡	0.52 (0.43 – 0.62)‡	0.54 (0.43 – 0.67)‡	0.01 (-0.09 – 0.12)
	1st	267.9 (234.4 – 306.3)	190.5 (171.8 – 211.1)	86.4 (62.7 – 119.7)	181.5 (137.1 – 223.6)§	67.8% (54.7 – 76.9)§	1.08 (0.97 – 1.20)	1.08 (1.01 – 1.16)‡	1.21 (1.08 – 1.36)‡	0.13 (-0.04 – 0.30)
	2nd	277.1 (246.8 – 310.2)	192.4 (173.4 – 212.3)	79.7 (58.3 – 109.2)	197.4 (157.4 – 235.7)§	71.2% (60.2 – 79.1)§	1.12 (1.03 – 1.21)‡	1.10 (1.03 – 1.17)‡	1.12 (1.01 – 1.22)‡	0.00 (-0.14 – 0.13)
Mozambique	3rd	259.3 (235.1 – 284.4)	184.3 (168.3 – 201.1)	73.0 (54.0 – 98.9)	186.2 (152.4 – 215.8)§	71.8% (61.5 – 79.3)§	1.05 (0.99 – 1.10)	1.05 (1.00 – 1.10)	1.02 (0.96 – 1.09)	-0.02 (-0.10 – 0.05)
	4th	249.5 (219.3 – 282.7)	180.3 (163.2 – 199.3)	63.6 (46.1 – 87.9)	185.9 (149.0 – 220.6)§	74.5% (64.1 – 81.6)§	1.01 (0.91 – 1.11)	1.03 (0.96 – 1.10)	0.89 (0.80 – 0.99)‡	-0.12 (-0.25 – 0.02)
	5th	184.7 (158.4 – 214.4)	130.5 (116.2 – 146.3)	53.8 (38.4 – 75.1)	131.0 (98.9 – 162.0)§	70.9% (58.3 – 79.3)§	0.75 (0.65 – 0.84)‡	0.74 (0.68 – 0.81)‡	0.75 (0.66 – 0.86)‡	0.01 (-0.12 – 0.14)
	1st	139.4 (114.3 – 166.4)	111.4 (93.1 – 132.4)	66.2 (48.5 – 86.9)	73.2 (45.1 – 101.4)§	52.5% (36.1 – 65.1)§	1.21 (1.01 – 1.41)‡	1.24 (1.05 – 1.44)‡	1.30 (1.11 – 1.51)‡	0.10 (-0.09 – 0.28)
	2nd	132.0 (113.9 – 151.0)	103.9 (90.3 – 118.9)	60.4 (45.6 – 78.2)	71.6 (47.5 – 95.2)§	54.2% (39.0 – 66.0)§	1.14 (1.01 – 1.27)‡	1.15 (1.03 – 1.28)‡	1.19 (1.06 – 1.32)‡	0.05 (-0.10 – 0.19)
Myanmar	3rd	122.7 (108.7 – 137.3)	95.1 (84.7 – 106.1)	52.0 (39.4 – 66.3)	70.7 (51.9 – 89.1)§	57.7% (45.2 – 68.3)§	1.06 (0.98 – 1.14)	1.06 (0.97 – 1.14)	1.02 (0.94 – 1.10)	-0.04 (-0.12 – 0.04)
	4th	105.4 (89.3 – 123.2)	79.7 (67.8 – 92.4)	42.8 (31.8 – 55.8)	62.6 (43.8 – 82.3)§	59.4% (45.6 – 70.4)§	0.91 (0.79 – 1.03)	0.89 (0.77 – 1.00)‡	0.84 (0.73 – 0.96)‡	-0.07 (-0.20 – 0.06)
	5th	77.9 (57.4 – 103.3)	59.9 (44.9 – 79.1)	32.6 (22.2 – 46.4)	45.3 (27.5 – 66.0)§	58.2% (42.5 – 69.9)§	0.67 (0.50 – 0.88)‡	0.67 (0.51 – 0.87)‡	0.64 (0.48 – 0.84)‡	-0.03 (-0.16 – 0.09)
	1st	81.7 (70.7 – 93.6)	85.7 (74.4 – 98.3)	56.2 (37.8 – 83.8)	25.5 (-3.4 – 46.2)	31.2% (-4.4 – 53.8)	1.15 (1.02 – 1.28)‡	1.15 (1.03 – 1.28)‡	1.24 (1.08 – 1.42)‡	0.10 (-0.07 – 0.28)
	2nd	84.5 (74.6 – 95.1)	88.7 (78.0 – 100.1)	53.9 (36.7 – 79.9)	30.6 (3.0 – 50.4)§	36.2% (3.9 – 56.9)§	1.19 (1.08 – 1.29)‡	1.19 (1.08 – 1.29)‡	1.19 (1.07 – 1.32)‡	0.01 (-0.13 – 0.15)
Namibia	3rd	74.8 (66.6 – 82.8)	78.5 (70.4 – 87.1)	46.3 (31.7 – 67.9)	28.4 (5.8 – 44.6)§	38.1% (7.6 – 57.8)§	1.05 (0.98 – 1.11)	1.05 (0.99 – 1.12)	1.02 (0.95 – 1.10)	-0.02 (-0.10 – 0.05)
	4th	66.2 (57.5 – 76.2)	70.0 (60.9 – 80.3)	40.1 (27.1 – 60.0)	26.1 (5.3 – 41.8)§	39.4% (8.2 – 59.6)§	0.93 (0.83 – 1.04)	0.94 (0.84 – 1.05)	0.89 (0.78 – 1.01)	-0.04 (-0.17 – 0.09)
	5th	49.3 (40.9 – 58.9)	50.2 (41.5 – 60.1)	29.4 (19.3 – 45.0)	19.9 (3.7 – 32.3)§	40.3% (8.0 – 61.0)§	0.69 (0.59 – 0.81)‡	0.67 (0.57 – 0.78)‡	0.65 (0.53 – 0.78)‡	-0.04 (-0.16 – 0.08)
	1st	153.9 (136.4 – 172.8)	96.2 (85.9 – 106.9)	44.1 (35.4 – 55.1)	109.8 (89.5 – 129.4)§	71.3% (63.4 – 77.5)§	1.09 (0.99 – 1.20)	1.18 (1.07 – 1.28)‡	1.28 (1.12 – 1.45)‡	0.19 (0.01 – 0.37)§
	2nd	163.0 (147.1 – 179.7)	95.1 (85.9 – 104.8)	41.1 (33.3 – 50.7)	121.8 (103.9 – 139.7)§	74.8% (68.3 – 79.9)§	1.16 (1.07 – 1.25)‡	1.16 (1.08 – 1.25)‡	1.19 (1.07 – 1.31)‡	0.04 (-0.11 – 0.18)
Nepal	3rd	154.9 (142.2 – 168.2)	88.7 (81.3 – 96.4)	35.6 (29.2 – 43.4)	119.3 (105.0 – 133.6)§	77.0% (71.6 – 81.4)§	1.10 (1.04 – 1.16)‡	1.09 (1.03 – 1.15)‡	1.03 (0.96 – 1.10)	-0.07 (-0.15 – 0.01)
	4th	137.8 (123.1 – 154.3)	74.1 (66.2 – 82.3)	29.5 (23.7 – 36.9)	108.3 (92.5 – 125.2)§	78.6% (72.9 – 83.1)§	0.98 (0.89 – 1.07)	0.91 (0.83 – 0.99)‡	0.86 (0.75 – 0.96)‡	-0.12 (-0.26 – 0.01)
	5th	95.0 (81.4 – 109.3)	54.4 (47.2 – 62.5)	22.2 (17.3 – 28.7)	72.8 (59.2 – 86.8)§	76.6% (69.5 – 82.0)§	0.67 (0.59 – 0.77)‡	0.67 (0.59 – 0.75)‡	0.64 (0.54 – 0.76)‡	-0.03 (-0.14 – 0.09)
	1st	81.4 (69.8 – 94.2)	51.0 (43.2 – 59.7)	26.1 (16.1 – 43.1)	55.3 (36.6 – 70.4)§	67.9% (47.1 – 80.4)§	1.21 (1.06 – 1.37)‡	1.26 (1.10 – 1.43)‡	1.33 (1.12 – 1.55)‡	0.12 (-0.08 – 0.33)
	2nd	81.9 (72.5 – 92.3)	49.4 (43.0 – 56.3)	23.5 (14.6 – 37.9)	58.4 (41.7 – 72.2)§	71.3% (53.4 – 82.2)§	1.21 (1.10 – 1.33)‡	1.22 (1.10 – 1.35)‡	1.19 (1.05 – 1.33)‡	-0.02 (-0.18 – 0.14)
Nicaragua	3rd	71.8 (64.7 – 79.4)	42.0 (37.4 – 47.0)	20.3 (12.7 – 32.5)	51.5 (37.8 – 62.0)§	71.8% (54.2 – 82.3)§	1.06 (0.99 – 1.14)	1.04 (0.96 – 1.12)	1.03 (0.94 – 1.12)	-0.04 (-0.12 – 0.05)
	4th	60.7 (52.7 – 69.7)	35.1 (30.1 – 40.9)	16.5 (10.3 – 26.8)	44.3 (31.6 – 54.9)§	72.9% (54.9 – 83.4)§	0.90 (0.80 – 1.01)	0.87 (0.76 – 0.98)‡	0.84 (0.71 – 0.97)‡	-0.06 (-0.21 – 0.08)
	5th	41.6 (33.1 – 51.9)	24.5 (19.3 – 30.8)	12.2 (7.1 – 20.4)	29.5 (19.4 – 39.0)§	70.8% (51.7 – 82.4)§	0.62 (0.50 – 0.76)‡	0.61 (0.48 – 0.75)‡	0.62 (0.48 – 0.78)‡	0.00 (-0.12 – 0.13)

Continued on next page

‡: Ratio is significantly different from one.
§: Change/decline is significantly different from zero.
Absolute decline: wealth quintile-specific U5MR (1990) - wealth quintile-specific U5MR (2016).
Percentage decline: absolute decline over wealth quintile-specific U5MR (1990) $\times 100$.
Change in ratio: ratio (2016) - ratio (1990).

Table 8 – continued from previous page

	Wealth Quintile	wealth quintile-specific U5MR (deaths per 1000 livebirths) 1990	2000	2016	Absolute decline 1990–2016	Percentage decline 1990–2016	Ratio of wealth quintile-specific to national-level U5MR 1990	2000	2016	Change 1990–2016
Niger	1st	305.4 (272.0 – 341.9)	206.1 (185.5 – 228.5)	96.5 (67.8 – 138.1)	208.9 (156.1 – 254.1)§	68.4% (54.2 – 78.2)§	0.93 (0.84 – 1.02)	0.91 (0.84 – 0.98)‡	1.06 (0.93 – 1.20)	0.13 (-0.02 – 0.28)
	2nd	395.2 (357.3 – 435.4)	266.4 (240.6 – 293.3)	106.9 (74.9 – 152.8)	288.3 (232.7 – 340.7)§	73.0% (61.2 – 81.2)§	1.20 (1.11 – 1.29)‡	1.18 (1.10 – 1.25)‡	1.17 (1.06 – 1.28)‡	-0.03 (-0.17 – 0.10)
	3rd	360.4 (332.0 – 390.3)	247.4 (226.2 – 269.6)	98.7 (69.9 – 138.9)	261.7 (214.6 – 302.2)§	72.6% (61.4 – 80.7)§	1.10 (1.04 – 1.15)‡	1.09 (1.04 – 1.14)‡	1.08 (1.02 – 1.14)‡	-0.01 (-0.09 – 0.06)
	4th	349.5 (313.6 – 386.9)	246.7 (222.4 – 272.7)	87.6 (61.2 – 124.5)	261.9 (210.6 – 307.8)§	74.9% (63.7 – 82.8)§	1.06 (0.97 – 1.15)	1.09 (1.01 – 1.17)‡	0.96 (0.86 – 1.07)	-0.10 (-0.24 – 0.03)
	5th	234.0 (205.3 – 264.1)	166.0 (147.7 – 186.5)	66.9 (46.2 – 96.9)	167.1 (126.5 – 203.4)§	71.4% (57.9 – 80.4)§	0.71 (0.64 – 0.79)‡	0.73 (0.67 – 0.80)‡	0.73 (0.63 – 0.85)‡	0.02 (-0.10 – 0.15)
Nigeria	1st	253.5 (225.3 – 284.2)	226.3 (206.1 – 247.1)	140.1 (101.4 – 189.5)	113.4 (58.4 – 160.2)§	44.7% (24.0 – 60.1)§	1.19 (1.08 – 1.31)‡	1.21 (1.13 – 1.29)‡	1.34 (1.21 – 1.48)‡	0.15 (-0.02 – 0.32)
	2nd	254.7 (230.1 – 281.4)	233.2 (213.5 – 254.3)	125.0 (92.0 – 169.4)	129.7 (80.3 – 170.0)§	50.9% (32.8 – 64.1)§	1.20 (1.10 – 1.29)‡	1.24 (1.17 – 1.32)‡	1.20 (1.10 – 1.31)‡	0.00 (-0.13 – 0.14)
	3rd	231.2 (211.9 – 251.0)	202.7 (186.9 – 218.7)	111.8 (82.5 – 149.6)	119.4 (78.9 – 152.4)§	51.7% (34.8 – 64.2)§	1.09 (1.03 – 1.14)‡	1.08 (1.03 – 1.13)‡	1.07 (1.01 – 1.13)‡	-0.01 (-0.09 – 0.06)
	4th	200.6 (177.7 – 224.9)	172.2 (155.7 – 189.7)	87.1 (63.2 – 118.1)	113.6 (76.1 – 145.7)§	56.6% (40.3 – 68.5)§	0.94 (0.85 – 1.04)	0.92 (0.85 – 0.99)‡	0.83 (0.75 – 0.93)‡	-0.11 (-0.23 – 0.01)
	5th	124.5 (109.4 – 142.2)	102.6 (91.6 – 114.2)	57.5 (41.5 – 79.2)	67.0 (42.3 – 89.5)§	53.8% (35.8 – 67.1)§	0.58 (0.52 – 0.65)‡	0.55 (0.50 – 0.60)‡	0.55 (0.49 – 0.63)‡	-0.03 (-0.13 – 0.06)
Pakistan	1st	160.0 (142.3 – 178.1)	135.7 (121.2 – 151.0)	98.4 (74.4 – 130.4)	61.6 (26.9 – 90.8)§	38.5% (17.5 – 54.1)§	1.15 (1.03 – 1.28)‡	1.20 (1.08 – 1.33)‡	1.25 (1.10 – 1.41)‡	0.10 (-0.07 – 0.27)
	2nd	161.0 (145.9 – 176.6)	131.0 (118.8 – 143.5)	92.1 (70.1 – 120.7)	68.9 (37.3 – 94.9)§	42.8% (23.9 – 56.9)§	1.16 (1.06 – 1.26)‡	1.16 (1.07 – 1.26)‡	1.17 (1.06 – 1.28)‡	0.01 (-0.13 – 0.14)
	3rd	148.5 (138.2 – 158.7)	120.6 (111.9 – 129.6)	82.7 (63.6 – 108.1)	65.8 (39.0 – 87.2)§	44.3% (26.8 – 57.2)§	1.07 (1.01 – 1.13)‡	1.07 (1.01 – 1.13)‡	1.05 (0.98 – 1.12)	-0.02 (-0.09 – 0.05)
	4th	132.2 (118.5 – 146.4)	100.7 (90.1 – 111.5)	68.8 (51.8 – 91.6)	63.4 (37.4 – 85.0)§	48.0% (29.5 – 61.2)§	0.95 (0.86 – 1.05)	0.89 (0.81 – 0.98)‡	0.87 (0.78 – 0.98)‡	-0.08 (-0.20 – 0.05)
	5th	92.2 (79.1 – 106.6)	76.0 (65.6 – 87.5)	52.0 (38.0 – 70.3)	40.3 (20.2 – 58.4)§	43.7% (23.2 – 59.1)§	0.66 (0.57 – 0.77)‡	0.67 (0.59 – 0.77)‡	0.66 (0.56 – 0.77)‡	-0.01 (-0.12 – 0.11)
Paraguay	1st	58.7 (49.1 – 69.7)	43.2 (34.8 – 52.8)	26.6 (15.8 – 44.1)	32.1 (13.5 – 46.4)§	54.7% (24.5 – 73.1)§	1.26 (1.09 – 1.44)‡	1.28 (1.09 – 1.49)‡	1.33 (1.12 – 1.57)‡	0.08 (-0.12 – 0.29)
	2nd	55.4 (47.6 – 64.0)	39.9 (33.0 – 47.7)	23.4 (14.1 – 38.0)	32.0 (16.3 – 43.8)§	57.8% (30.6 – 74.6)§	1.19 (1.06 – 1.32)‡	1.19 (1.05 – 1.32)‡	1.18 (1.03 – 1.32)‡	-0.01 (-0.18 – 0.14)
	3rd	48.6 (42.5 – 55.1)	34.6 (29.5 – 40.7)	20.4 (12.4 – 33.0)	28.2 (15.0 – 38.1)§	58.1% (31.8 – 74.6)§	1.04 (0.96 – 1.12)	1.03 (0.95 – 1.11)	1.02 (0.93 – 1.12)	-0.02 (-0.11 – 0.06)
	4th	41.1 (34.8 – 48.3)	29.3 (24.0 – 35.6)	16.6 (9.9 – 27.2)	24.5 (12.8 – 33.8)§	59.6% (33.2 – 76.0)§	0.88 (0.77 – 1.00)‡	0.87 (0.75 – 1.00)‡	0.84 (0.70 – 0.98)‡	-0.05 (-0.20 – 0.10)
	5th	29.2 (21.8 – 38.1)	21.1 (15.4 – 28.2)	12.6 (7.2 – 21.7)	16.6 (7.3 – 25.0)§	56.9% (27.5 – 74.3)§	0.63 (0.47 – 0.80)‡	0.63 (0.47 – 0.81)‡	0.63 (0.47 – 0.83)‡	0.01 (-0.11 – 0.14)
Peru	1st	118.7 (108.1 – 129.3)	59.1 (53.3 – 65.1)	23.9 (18.0 – 32.1)	94.8 (81.5 – 106.6)§	79.8% (72.5 – 84.9)§	1.48 (1.37 – 1.58)‡	1.53 (1.42 – 1.65)‡	1.56 (1.38 – 1.76)‡	0.09 (-0.13 – 0.31)
	2nd	100.4 (91.4 – 109.6)	48.2 (43.5 – 53.3)	18.2 (13.8 – 24.5)	82.2 (71.4 – 92.5)§	81.9% (75.2 – 86.5)§	1.25 (1.16 – 1.34)‡	1.25 (1.16 – 1.35)‡	1.19 (1.05 – 1.33)‡	-0.06 (-0.22 – 0.10)
	3rd	82.0 (75.4 – 88.8)	38.0 (34.3 – 41.6)	15.1 (11.5 – 20.1)	66.8 (59.1 – 74.2)§	81.5% (75.3 – 85.9)§	1.02 (0.95 – 1.08)	0.99 (0.92 – 1.05)	0.99 (0.90 – 1.08)	-0.03 (-0.12 – 0.06)
	4th	61.9 (55.4 – 68.7)	28.5 (25.1 – 32.3)	11.4 (8.3 – 15.6)	50.5 (43.1 – 57.7)§	81.6% (74.5 – 86.8)§	0.77 (0.70 – 0.85)‡	0.74 (0.67 – 0.82)‡	0.75 (0.62 – 0.87)‡	-0.02 (-0.16 – 0.11)
	5th	39.1 (33.4 – 45.1)	18.7 (15.9 – 21.9)	7.8 (5.6 – 11.0)	31.2 (25.4 – 37.2)§	80.0% (71.5 – 85.6)§	0.49 (0.42 – 0.55)‡	0.49 (0.42 – 0.56)‡	0.51 (0.41 – 0.63)‡	0.02 (-0.07 – 0.14)
Philippines	1st	87.5 (78.5 – 97.1)	60.7 (53.9 – 68.1)	41.2 (29.6 – 57.1)	46.3 (29.1 – 60.6)§	52.9% (34.5 – 66.3)§	1.51 (1.38 – 1.64)‡	1.53 (1.40 – 1.66)‡	1.52 (1.34 – 1.70)‡	0.01 (-0.18 – 0.21)
	2nd	68.6 (61.6 – 75.9)	47.4 (42.4 – 52.9)	31.5 (22.6 – 43.2)	37.1 (23.9 – 48.6)§	54.1% (36.2 – 67.5)§	1.18 (1.09 – 1.28)‡	1.19 (1.10 – 1.29)‡	1.16 (1.05 – 1.28)‡	-0.02 (-0.16 – 0.12)
	3rd	58.5 (53.2 – 64.1)	39.2 (35.4 – 43.3)	26.8 (19.5 – 36.7)	31.7 (20.8 – 40.5)§	54.1% (36.7 – 67.0)§	1.01 (0.94 – 1.07)	0.99 (0.92 – 1.05)	0.99 (0.91 – 1.06)	-0.02 (-0.09 – 0.06)
	4th	45.3 (39.6 – 51.2)	31.1 (27.1 – 35.3)	21.8 (15.6 – 30.3)	23.5 (13.8 – 31.8)§	51.9% (32.3 – 65.9)§	0.78 (0.69 – 0.86)‡	0.78 (0.70 – 0.87)‡	0.80 (0.70 – 0.91)‡	0.02 (-0.09 – 0.15)
	5th	30.6 (25.8 – 36.0)	20.1 (16.8 – 24.1)	14.2 (9.8 – 20.3)	16.4 (9.7 – 22.6)§	53.7% (33.6 – 67.8)§	0.53 (0.45 – 0.61)‡	0.51 (0.43 – 0.59)‡	0.52 (0.43 – 0.63)‡	0.00 (-0.10 – 0.09)
Republic of Moldova	1st	42.9 (33.9 – 53.3)	40.3 (31.3 – 51.3)	21.5 (14.3 – 33.0)	21.4 (8.0 – 33.0)§	49.8% (20.7 – 67.1)§	1.30 (1.08 – 1.52)‡	1.30 (1.08 – 1.52)‡	1.35 (1.11 – 1.61)‡	0.05 (-0.17 – 0.29)
	2nd	38.1 (31.2 – 46.1)	35.9 (28.9 – 44.6)	18.2 (12.5 – 27.2)	19.9 (8.6 – 29.6)§	52.2% (24.8 – 68.4)§	1.16 (1.02 – 1.30)‡	1.15 (1.02 – 1.30)‡	1.15 (0.99 – 1.31)	-0.01 (-0.18 – 0.16)
	3rd	33.0 (27.5 – 39.0)	31.1 (25.3 – 37.8)	15.8 (11.0 – 23.5)	17.1 (7.7 – 24.7)§	52.0% (25.9 – 67.5)§	1.00 (0.91 – 1.08)	1.00 (0.91 – 1.08)	1.00 (0.89 – 1.09)	0.00 (-0.09 – 0.08)
	4th	27.6 (22.2 – 33.6)	25.9 (20.5 – 32.4)	12.6 (8.5 – 19.4)	15.0 (6.7 – 22.0)§	54.3% (26.8 – 70.2)§	0.84 (0.72 – 0.95)‡	0.83 (0.72 – 0.95)‡	0.79 (0.65 – 0.94)‡	-0.04 (-0.20 – 0.11)
	5th	23.5 (17.0 – 32.0)	22.3 (16.0 – 31.1)	11.4 (7.2 – 18.4)	12.1 (4.3 – 19.7)§	51.6% (21.2 – 68.4)§	0.71 (0.53 – 0.93)‡	0.72 (0.55 – 0.94)‡	0.71 (0.54 – 0.95)‡	0.00 (-0.13 – 0.15)
Rwanda	1st	149.4 (133.0 – 167.2)	199.8 (177.5 – 224.6)	47.9 (30.1 – 75.4)	101.4 (69.5 – 125.8)§	67.9% (48.5 – 79.9)§	0.99 (0.89 – 1.09)	1.03 (0.94 – 1.12)	1.25 (1.10 – 1.40)‡	0.25 (0.09 – 0.43)§
	2nd	167.8 (151.6 – 185.0)	206.9 (185.0 – 230.6)	43.4 (27.3 – 68.1)	124.4 (96.2 – 146.8)§	74.1% (59.4 – 83.6)§	1.11 (1.03 – 1.20)‡	1.06 (0.98 – 1.14)	1.13 (1.02 – 1.25)‡	0.01 (-0.12 – 0.15)
	3rd	157.0 (144.5 – 170.7)	202.7 (184.4 – 223.1)	38.4 (24.4 – 60.1)	118.5 (94.4 – 137.2)§	75.5% (61.5 – 84.4)§	1.04 (0.99 – 1.10)	1.04 (0.99 – 1.10)	1.00 (0.93 – 1.07)	-0.04 (-0.12 – 0.03)
	4th	157.7 (141.5 – 174.6)	212.2 (190.3 – 236.7)	34.3 (21.6 – 54.1)	123.4 (98.6 – 144.2)§	78.2% (65.3 – 86.3)§	1.05 (0.96 – 1.14)	1.09 (1.01 – 1.18)‡	0.89 (0.79 – 1.00)‡	-0.16 (-0.29 – -0.03)§
	5th	121.2 (107.3 – 136.6)	151.9 (133.6 – 171.7)	28.4 (17.7 – 45.2)	92.8 (72.0 – 111.4)§	76.6% (62.3 – 85.4)§	0.80 (0.72 – 0.89)‡	0.78 (0.71 – 0.86)‡	0.74 (0.63 – 0.85)‡	-0.07 (-0.20 – 0.06)
Sao Tome and Principe	1st	119.1 (96.0 – 144.9)	96.0 (77.4 – 116.9)	41.9 (26.9 – 63.0)	77.2 (48.2 – 104.3)§	64.8% (45.1 – 77.5)§	1.14 (0.96 – 1.32)	1.15 (0.98 – 1.34)	1.24 (1.04 – 1.44)‡	0.10 (-0.09 – 0.30)
	2nd	114.3 (96.1 – 134.7)	91.4 (76.3 – 108.2)	38.4 (25.0 – 57.9)	75.9 (49.5 – 99.5)§	66.4% (48.1 – 78.5)§	1.09 (0.97 – 1.21)	1.10 (0.98 – 1.22)	1.14 (1.01 – 1.27)‡	0.05 (-0.10 – 0.20)
	3rd	107.9 (92.6 – 124.9)	85.0 (72.8 – 99.3)	33.4 (22.0 – 50.0)	74.5 (52.7 – 94.5)§	69.0% (52.7 – 80.0)§	1.03 (0.95 – 1.11)	1.02 (0.94 – 1.10)	0.99 (0.91 – 1.07)	-0.04 (-0.12 – 0.04)
	4th	91.6 (75.7 – 109.5)	72.0 (59.3 – 86.5)	28.1 (18.3 – 42.5)	63.6 (43.0 – 82.5)§	69.4% (51.8 – 80.5)§	0.87 (0.76 – 0.99)‡	0.87 (0.76 – 0.98)‡	0.83 (0.72 – 0.95)‡	-0.04 (-0.17 – 0.09)
	5th	91.0 (70.6 – 115.9)	71.6 (55.7 – 91.2)	27.2 (17.1 – 42.8)	63.9 (42.0 – 87.4)§	70.1% (53.0 – 81.1)§	0.87 (0.70 – 1.07)	0.86 (0.70 – 1.05)	0.80 (0.64 – 1.00)	-0.06 (-0.22 – 0.08)

Continued on next page

‡: Ratio is significantly different from one.
§: Change/decline is significantly different from zero.
Absolute decline: wealth quintile-specific U5MR (1990) - wealth quintile-specific U5MR (2016).
Percentage decline: absolute decline over wealth quintile-specific U5MR (1990) $\times 100$.
Change in ratio: ratio (2016) - ratio (1990).

Table 8 – continued from previous page

	Wealth Quintile	wealth quintile-specific U5MR (deaths per 1000 livebirths) 1990	2000	2016	Absolute decline 1990–2016	Percentage decline 1990–2016	Ratio of wealth quintile-specific to national-level U5MR 1990	2000	2016	Change 1990–2016
Senegal	1st	179.1 (160.7 – 198.3)	173.4 (157.8 – 190.6)	65.1 (50.4 – 84.7)	114.0 (88.3 – 138.0)§	63.6% (52.2 – 72.2)§	1.28 (1.16 – 1.39)‡	1.29 (1.20 – 1.39)‡	1.38 (1.24 – 1.52)‡	0.11 (-0.07 – 0.28)
	2nd	162.4 (147.4 – 178.2)	154.9 (141.2 – 169.4)	55.6 (43.2 – 71.9)	106.8 (85.2 – 126.7)§	65.8% (54.8 – 73.6)§	1.16 (1.06 – 1.25)‡	1.15 (1.08 – 1.23)‡	1.18 (1.08 – 1.29)‡	0.02 (-0.11 – 0.16)
	3rd	148.9 (138.1 – 160.2)	142.1 (131.1 – 154.1)	47.9 (37.6 – 61.5)	101.0 (84.4 – 115.8)§	67.8% (58.5 – 74.9)§	1.06 (1.00 – 1.12)‡	1.06 (1.00 – 1.12)‡	1.02 (0.95 – 1.08)	-0.04 (-0.12 – 0.03)
	4th	125.5 (111.5 – 140.3)	118.1 (105.9 – 131.5)	39.4 (30.3 – 51.4)	86.1 (67.7 – 103.3)§	68.6% (58.1 – 76.3)§	0.89 (0.80 – 0.99)‡	0.88 (0.80 – 0.96)‡	0.84 (0.74 – 0.93)‡	-0.06 (-0.18 – 0.07)
	5th	86.2 (74.2 – 99.7)	82.5 (71.9 – 94.0)	27.6 (20.8 – 36.7)	58.6 (45.0 – 72.2)§	68.0% (56.5 – 76.1)§	0.61 (0.53 – 0.70)‡	0.61 (0.54 – 0.69)‡	0.59 (0.50 – 0.68)‡	-0.03 (-0.13 – 0.08)
Serbia	1st	37.3 (31.1 – 44.2)	17.3 (14.2 – 20.9)	7.9 (6.3 – 10.0)	29.4 (23.7 – 35.5)§	78.8% (73.6 – 82.6)§	1.32 (1.10 – 1.56)‡	1.37 (1.12 – 1.64)‡	1.37 (1.11 – 1.64)‡	0.04 (-0.18 – 0.28)
	2nd	32.6 (28.5 – 36.6)	14.6 (12.5 – 16.6)	6.7 (5.5 – 8.0)	25.9 (22.0 – 29.6)§	79.5% (75.2 – 83.0)§	1.15 (1.01 – 1.29)‡	1.15 (0.99 – 1.31)	1.15 (0.99 – 1.31)	-0.01 (-0.17 – 0.16)
	3rd	28.2 (25.6 – 30.7)	12.6 (11.3 – 13.9)	5.8 (4.9 – 6.8)	22.4 (20.0 – 24.6)§	79.5% (76.1 – 82.2)§	1.00 (0.91 – 1.09)	1.00 (0.89 – 1.10)	1.00 (0.89 – 1.10)	0.00 (-0.09 – 0.08)
	4th	23.6 (20.1 – 27.2)	10.1 (8.2 – 12.0)	4.6 (3.7 – 5.8)	18.9 (15.6 – 22.4)§	80.3% (75.4 – 84.4)§	0.84 (0.71 – 0.96)‡	0.80 (0.65 – 0.95)‡	0.80 (0.65 – 0.95)‡	-0.04 (-0.20 – 0.11)
	5th	19.4 (14.5 – 25.8)	8.8 (6.5 – 11.7)	4.0 (2.9 – 5.5)	15.4 (11.2 – 20.7)§	79.4% (73.9 – 83.4)§	0.69 (0.51 – 0.91)‡	0.69 (0.51 – 0.93)‡	0.69 (0.51 – 0.92)‡	0.00 (-0.13 – 0.14)
Sierra Leone	1st	280.2 (241.9 – 320.9)	255.0 (227.8 – 284.7)	130.5 (99.8 – 165.9)	149.7 (102.9 – 197.3)§	53.4% (39.5 – 65.1)§	1.07 (0.96 – 1.19)	1.09 (1.01 – 1.18)‡	1.15 (1.03 – 1.28)‡	0.08 (-0.09 – 0.24)
	2nd	275.0 (240.4 – 312.0)	243.1 (217.5 – 270.0)	120.2 (92.6 – 150.5)	154.8 (111.6 – 197.8)§	56.3% (43.9 – 66.8)§	1.05 (0.95 – 1.15)	1.04 (0.96 – 1.11)	1.06 (0.96 – 1.15)	0.01 (-0.12 – 0.14)
	3rd	268.1 (240.0 – 297.3)	238.7 (216.4 – 262.8)	115.8 (90.4 – 144.6)	152.2 (116.7 – 188.1)§	56.8% (45.5 – 66.3)§	1.02 (0.96 – 1.08)	1.02 (0.97 – 1.07)	1.02 (0.96 – 1.08)	0.00 (-0.07 – 0.07)
	4th	274.5 (240.7 – 313.2)	242.1 (216.4 – 271.3)	103.3 (78.9 – 130.9)	171.2 (130.1 – 214.1)§	62.4% (51.4 – 71.7)§	1.05 (0.95 – 1.16)	1.04 (0.96 – 1.12)	0.91 (0.82 – 1.01)	-0.14 (-0.28 – -0.01)§
	5th	212.7 (179.8 – 247.8)	190.5 (167.5 – 215.8)	97.7 (74.2 – 125.2)	115.0 (75.6 – 152.9)§	54.1% (39.4 – 65.6)§	0.81 (0.70 – 0.92)‡	0.81 (0.74 – 0.89)‡	0.86 (0.76 – 0.97)‡	0.05 (-0.08 – 0.19)
Somalia	1st	195.6 (150.1 – 252.5)	188.6 (138.8 – 253.9)	151.1 (81.3 – 282.0)	44.5 (-69.5 – 112.3)	22.7% (-35.1 – 55.0)	1.08 (0.90 – 1.27)	1.08 (0.91 – 1.26)	1.14 (0.96 – 1.33)	0.06 (-0.11 – 0.23)
	2nd	205.3 (165.3 – 257.4)	198.2 (151.8 – 263.5)	150.4 (82.4 – 279.4)	54.9 (-56.7 – 119.2)	26.7% (-26.7 – 57.4)	1.14 (1.01 – 1.26)‡	1.14 (1.02 – 1.26)‡	1.14 (1.01 – 1.26)‡	0.00 (-0.14 – 0.14)
	3rd	191.7 (156.8 – 238.0)	184.7 (143.9 – 242.0)	140.4 (78.0 – 259.8)	51.3 (-50.3 – 110.2)	26.8% (-26.2 – 56.8)	1.06 (0.98 – 1.14)	1.06 (0.98 – 1.14)	1.06 (0.98 – 1.14)	0.00 (-0.07 – 0.07)
	4th	180.4 (144.0 – 228.3)	173.4 (132.4 – 231.5)	125.3 (68.8 – 234.5)	55.2 (-40.4 – 109.8)	30.6% (-21.8 – 59.3)	1.00 (0.88 – 1.12)	0.99 (0.88 – 1.12)	0.95 (0.83 – 1.07)	-0.05 (-0.19 – 0.08)
	5th	131.5 (93.4 – 182.0)	127.0 (87.3 – 184.6)	95.4 (50.6 – 183.1)	36.1 (-38.3 – 83.3)	27.5% (-29.8 – 57.9)	0.73 (0.55 – 0.95)‡	0.73 (0.55 – 0.95)‡	0.72 (0.54 – 0.94)‡	-0.01 (-0.13 – 0.13)
South Africa	1st	78.7 (65.1 – 94.7)	89.9 (75.4 – 106.1)	59.9 (47.5 – 73.3)	18.8 (2.8 – 36.1)§	23.9% (3.9 – 40.9)§	1.37 (1.19 – 1.56)‡	1.35 (1.17 – 1.54)‡	1.38 (1.18 – 1.60)‡	0.01 (-0.19 – 0.21)
	2nd	67.3 (57.0 – 78.6)	77.7 (66.9 – 89.0)	50.8 (41.8 – 61.1)	16.4 (3.4 – 30.0)§	24.4% (5.7 – 40.2)§	1.17 (1.05 – 1.29)‡	1.16 (1.04 – 1.28)‡	1.17 (1.04 – 1.30)‡	0.00 (-0.14 – 0.15)
	3rd	58.4 (50.6 – 67.4)	68.5 (60.0 – 77.1)	43.7 (36.5 – 51.6)	14.7 (4.6 – 25.3)§	25.2% (8.6 – 39.5)§	1.02 (0.94 – 1.09)	1.03 (0.95 – 1.10)	1.01 (0.93 – 1.09)	-0.01 (-0.08 – 0.07)
	4th	48.9 (41.0 – 58.2)	57.7 (48.5 – 67.4)	36.5 (29.5 – 44.5)	12.5 (2.5 – 22.7)§	25.5% (5.7 – 41.4)§	0.85 (0.74 – 0.97)‡	0.87 (0.76 – 0.98)‡	0.84 (0.73 – 0.96)‡	-0.01 (-0.14 – 0.12)
	5th	33.7 (25.2 – 44.5)	39.6 (29.7 – 51.5)	25.7 (18.4 – 34.7)	8.0 (0.3 – 16.3)§	23.9% (0.9 – 41.8)§	0.59 (0.45 – 0.75)‡	0.59 (0.45 – 0.76)‡	0.59 (0.45 – 0.77)‡	0.01 (-0.10 – 0.12)
South Sudan	1st	251.6 (194.2 – 317.6)	180.6 (143.6 – 224.6)	98.6 (59.5 – 160.3)	153.0 (71.5 – 226.4)§	60.8% (32.8 – 77.1)§	0.98 (0.82 – 1.16)	0.98 (0.82 – 1.15)	1.09 (0.92 – 1.27)	0.11 (-0.06 – 0.27)
	2nd	276.3 (221.3 – 334.1)	197.7 (162.6 – 238.6)	98.1 (59.3 – 157.4)	178.1 (98.2 – 249.9)§	64.5% (39.9 – 79.2)§	1.08 (0.95 – 1.20)	1.07 (0.95 – 1.19)	1.08 (0.96 – 1.20)	0.00 (-0.13 – 0.14)
	3rd	259.4 (210.6 – 308.2)	185.6 (154.7 – 220.5)	90.7 (54.9 – 145.6)	168.6 (96.0 – 231.2)§	65.0% (41.1 – 79.4)§	1.01 (0.93 – 1.09)	1.01 (0.93 – 1.08)	1.00 (0.92 – 1.08)	-0.01 (-0.08 – 0.06)
	4th	247.0 (196.2 – 302.1)	175.4 (142.9 – 213.3)	77.5 (46.5 – 124.6)	169.4 (99.3 – 232.7)§	68.6% (46.1 – 81.6)§	0.96 (0.84 – 1.09)	0.95 (0.83 – 1.07)	0.85 (0.75 – 0.97)‡	-0.11 (-0.24 – 0.03)
	5th	247.8 (186.4 – 322.6)	182.7 (142.6 – 235.2)	88.5 (52.5 – 145.0)	159.3 (81.7 – 237.6)§	64.3% (38.8 – 79.7)§	0.97 (0.78 – 1.19)	0.99 (0.82 – 1.20)	0.98 (0.80 – 1.18)	0.01 (-0.16 – 0.18)
State of Palestine	1st	57.6 (47.3 – 68.7)	39.1 (32.2 – 46.8)	26.2 (18.0 – 38.3)	31.4 (17.2 – 43.8)§	54.5% (32.7 – 68.8)§	1.29 (1.08 – 1.51)‡	1.31 (1.10 – 1.53)‡	1.35 (1.12 – 1.59)‡	0.06 (-0.16 – 0.28)
	2nd	52.7 (45.4 – 60.2)	35.0 (30.2 – 40.2)	22.6 (16.0 – 32.4)	30.1 (18.3 – 40.1)§	57.2% (37.3 – 70.4)§	1.18 (1.05 – 1.32)‡	1.17 (1.04 – 1.31)‡	1.16 (1.02 – 1.31)‡	-0.02 (-0.18 – 0.14)
	3rd	45.6 (40.4 – 50.9)	30.4 (26.8 – 34.1)	19.7 (13.9 – 27.8)	25.9 (16.5 – 33.7)§	56.9% (38.0 – 69.9)§	1.02 (0.94 – 1.10)	1.02 (0.93 – 1.10)	1.01 (0.92 – 1.10)	-0.01 (-0.10 – 0.08)
	4th	38.3 (32.4 – 44.8)	25.6 (21.6 – 30.0)	16.0 (11.1 – 23.5)	22.3 (13.3 – 30.1)§	58.2% (37.5 – 71.7)§	0.86 (0.74 – 0.98)‡	0.86 (0.74 – 0.98)‡	0.82 (0.69 – 0.96)‡	-0.03 (-0.18 – 0.12)
	5th	28.9 (21.5 – 37.7)	19.4 (14.5 – 25.1)	12.6 (8.1 – 19.3)	16.3 (8.7 – 23.9)§	56.4% (34.9 – 70.9)§	0.65 (0.49 – 0.84)‡	0.65 (0.50 – 0.83)‡	0.65 (0.49 – 0.84)‡	0.00 (-0.12 – 0.13)
Sudan	1st	145.2 (122.4 – 170.2)	118.3 (100.5 – 137.6)	77.0 (59.8 – 98.3)	68.2 (40.4 – 96.0)§	47.0% (30.6 – 59.8)§	1.11 (0.95 – 1.27)	1.13 (0.98 – 1.28)	1.18 (1.04 – 1.33)‡	0.08 (-0.10 – 0.25)
	2nd	153.4 (134.6 – 173.5)	123.5 (109.0 – 139.5)	78.5 (61.9 – 98.9)	74.9 (48.7 – 99.9)§	48.8% (34.2 – 60.5)§	1.17 (1.05 – 1.29)‡	1.18 (1.07 – 1.29)‡	1.21 (1.10 – 1.32)‡	0.04 (-0.11 – 0.19)
	3rd	142.2 (128.3 – 156.6)	113.5 (102.1 – 125.9)	68.8 (55.0 – 85.1)	73.4 (52.9 – 92.2)§	51.6% (39.5 – 61.8)§	1.08 (1.01 – 1.15)‡	1.08 (1.01 – 1.15)‡	1.06 (0.99 – 1.12)	-0.03 (-0.10 – 0.05)
	4th	126.7 (109.6 – 145.9)	97.1 (84.2 – 111.7)	58.6 (46.0 – 74.3)	68.1 (46.4 – 89.4)§	53.7% (40.0 – 64.5)§	0.96 (0.85 – 1.08)	0.93 (0.83 – 1.04)	0.90 (0.81 – 1.00)	-0.06 (-0.19 – 0.07)
	5th	89.4 (71.8 – 109.6)	71.1 (58.4 – 85.6)	42.5 (32.1 – 55.2)	46.9 (27.9 – 66.8)§	52.5% (36.4 – 64.6)§	0.68 (0.56 – 0.83)‡	0.68 (0.57 – 0.81)‡	0.65 (0.55 – 0.77)‡	-0.03 (-0.16 – 0.09)
Suriname	1st	64.1 (49.2 – 82.1)	47.2 (34.2 – 64.5)	28.6 (13.6 – 59.4)	35.5 (3.9 – 57.3)§	55.4% (6.8 – 79.1)§	1.38 (1.18 – 1.61)‡	1.41 (1.20 – 1.63)‡	1.43 (1.20 – 1.68)‡	0.05 (-0.18 – 0.27)
	2nd	53.8 (42.1 – 66.8)	38.4 (28.3 – 52.1)	22.8 (11.0 – 47.3)	31.0 (5.3 – 47.8)§	57.6% (10.4 – 79.6)§	1.16 (1.03 – 1.29)‡	1.15 (1.01 – 1.28)‡	1.14 (1.00 – 1.29)	-0.02 (-0.18 – 0.14)
	3rd	46.7 (37.2 – 57.6)	33.4 (25.0 – 44.8)	19.9 (9.6 – 41.2)	26.8 (5.1 – 40.9)§	57.3% (11.4 – 79.6)§	1.01 (0.92 – 1.09)	1.00 (0.91 – 1.08)	1.00 (0.90 – 1.09)	-0.01 (-0.10 – 0.08)
	4th	39.0 (30.5 – 49.4)	28.1 (20.6 – 38.1)	16.2 (7.7 – 33.8)	22.8 (4.8 – 35.7)§	58.4% (12.9 – 80.7)§	0.84 (0.73 – 0.96)‡	0.84 (0.72 – 0.96)‡	0.81 (0.68 – 0.95)‡	-0.03 (-0.18 – 0.11)
	5th	28.4 (19.9 – 40.1)	20.4 (13.6 – 30.5)	12.5 (5.6 – 26.8)	16.0 (2.2 – 27.3)§	56.2% (8.7 – 79.6)§	0.61 (0.46 – 0.81)‡	0.61 (0.45 – 0.81)‡	0.62 (0.46 – 0.82)‡	0.01 (-0.11 – 0.13)

Continued on next page

‡: Ratio is significantly different from one.
§: Change/decline is significantly different from zero.
Absolute decline: wealth quintile-specific U5MR (1990) - wealth quintile-specific U5MR (2016).
Percentage decline: absolute decline over wealth quintile-specific U5MR (1990) ×100.
Change in ratio: ratio (2016) - ratio (1990).

Table 8 – continued from previous page

	Wealth Quintile	wealth quintile-specific U5MR (deaths per 1000 livebirths) 1990	2000	2016	Absolute decline 1990–2016	Percentage decline 1990–2016	Ratio of wealth quintile-specific to national-level U5MR 1990	2000	2016	Change 1990–2016
Swaziland	1st	83.4 (69.5 – 99.4)	141.4 (121.1 – 163.4)	89.3 (59.7 – 130.7)	-6.0 (-47.5 – 25.2)	-7.2% (-58.9 – 28.5)	1.27 (1.11 – 1.44)‡	1.20 (1.07 – 1.34)‡	1.27 (1.12 – 1.43)‡	0.00 (-0.18 – 0.18)
	2nd	72.3 (61.2 – 84.7)	122.8 (106.8 – 139.4)	75.9 (51.2 – 109.9)	-3.6 (-38.8 – 22.0)	-5.0% (-56.0 – 29.3)	1.10 (0.99 – 1.21)	1.05 (0.95 – 1.14)	1.08 (0.97 – 1.19)	-0.02 (-0.15 – 0.11)
	3rd	65.4 (56.6 – 75.2)	119.1 (105.4 – 134.3)	69.9 (47.2 – 101.7)	-4.5 (-36.3 – 18.4)	-6.9% (-57.2 – 27.2)	0.99 (0.92 – 1.06)	1.01 (0.95 – 1.08)	0.99 (0.92 – 1.06)	0.00 (-0.07 – 0.07)
	4th	57.5 (48.1 – 68.1)	109.9 (95.3 – 127.4)	62.5 (42.1 – 92.2)	-5.0 (-35.4 – 16.5)	-8.7% (-63.3 – 27.3)	0.87 (0.77 – 0.98)‡	0.94 (0.84 – 1.04)	0.89 (0.79 – 1.00)	0.01 (-0.11 – 0.14)
	5th	50.4 (40.6 – 61.7)	94.2 (79.1 – 111.5)	54.3 (35.9 – 80.0)	-3.9 (-29.6 – 15.4)	-7.7% (-63.0 – 29.2)	0.77 (0.64 – 0.90)‡	0.80 (0.69 – 0.92)‡	0.77 (0.65 – 0.90)‡	0.01 (-0.13 – 0.14)
Syrian Arab Republic	1st	46.6 (38.2 – 55.6)	30.1 (24.8 – 35.6)	22.7 (16.5 – 33.0)	24.0 (11.2 – 33.8)§	51.4% (26.5 – 65.0)§	1.26 (1.05 – 1.47)‡	1.28 (1.07 – 1.48)‡	1.30 (1.07 – 1.53)‡	0.04 (-0.09 – 0.38)
	2nd	43.6 (37.6 – 50.0)	27.5 (23.6 – 31.4)	20.1 (15.0 – 28.9)	23.5 (12.7 – 31.5)§	53.9% (31.6 – 66.2)§	1.18 (1.04 – 1.31)‡	1.16 (1.03 – 1.30)‡	1.15 (1.00 – 1.31)	-0.03 (-0.11 – 0.24)
	3rd	37.7 (33.3 – 42.1)	23.9 (21.1 – 26.7)	17.6 (13.2 – 25.2)	20.0 (11.1 – 26.0)§	53.2% (31.3 – 65.0)§	1.02 (0.93 – 1.09)	1.01 (0.92 – 1.10)	1.01 (0.90 – 1.10)	-0.01 (-0.02 – 0.17)
	4th	31.8 (26.8 – 37.0)	19.9 (16.8 – 23.3)	14.4 (10.4 – 21.1)	17.3 (9.2 – 23.5)§	54.5% (31.8 – 67.6)§	0.86 (0.74 – 0.98)‡	0.84 (0.73 – 0.97)‡	0.83 (0.68 – 0.97)‡	-0.03 (-0.13 – 0.20)
	5th	25.8 (19.3 – 34.0)	16.6 (12.5 – 21.9)	12.6 (8.5 – 19.6)	13.2 (5.9 – 20.0)§	51.0% (25.5 – 65.3)§	0.70 (0.53 – 0.91)‡	0.70 (0.54 – 0.91)‡	0.72 (0.54 – 0.95)‡	0.03 (-0.06 – 0.24)
Tajikistan	1st	124.2 (103.9 – 147.0)	107.8 (89.3 – 130.4)	54.0 (32.0 – 92.1)	70.3 (29.8 – 99.2)§	56.6% (25.0 – 74.2)§	1.16 (1.02 – 1.31)‡	1.16 (1.03 – 1.30)‡	1.25 (1.08 – 1.43)‡	0.09 (-0.09 – 0.28)
	2nd	118.4 (101.7 – 136.9)	103.7 (87.1 – 123.6)	49.8 (29.6 – 85.1)	68.6 (31.5 – 93.7)§	58.0% (27.8 – 74.8)§	1.11 (1.00 – 1.22)‡	1.12 (1.01 – 1.22)‡	1.15 (1.03 – 1.28)‡	0.05 (-0.09 – 0.19)
	3rd	111.2 (97.2 – 126.5)	96.1 (81.9 – 113.0)	42.9 (25.9 – 73.2)	68.3 (37.4 – 89.4)§	61.4% (34.4 – 76.6)§	1.04 (0.97 – 1.11)	1.04 (0.97 – 1.10)	1.00 (0.92 – 1.07)	-0.04 (-0.12 – 0.03)
	4th	93.7 (79.3 – 109.9)	79.2 (65.9 – 95.2)	35.2 (21.0 – 61.2)	58.5 (31.0 – 78.5)§	62.4% (34.7 – 77.6)§	0.87 (0.78 – 0.98)‡	0.85 (0.76 – 0.95)‡	0.82 (0.71 – 0.93)‡	-0.06 (-0.18 – 0.07)
	5th	88.0 (71.1 – 107.9)	76.8 (61.7 – 95.1)	33.6 (19.5 – 59.1)	54.4 (26.6 – 75.9)§	61.8% (32.2 – 77.5)§	0.82 (0.68 – 0.98)‡	0.83 (0.70 – 0.97)‡	0.78 (0.64 – 0.94)‡	-0.04 (-0.18 – 0.10)
Thailand	1st	48.8 (40.4 – 57.7)	30.4 (24.2 – 37.0)	16.5 (9.7 – 28.1)	32.3 (19.3 – 42.5)§	66.2% (41.9 – 80.1)§	1.29 (1.08 – 1.51)‡	1.32 (1.09 – 1.54)‡	1.35 (1.11 – 1.63)‡	0.06 (-0.16 – 0.31)
	2nd	44.8 (38.9 – 50.9)	27.2 (22.7 – 32.0)	14.2 (8.5 – 24.0)	30.5 (19.8 – 38.5)§	68.2% (46.3 – 80.9)§	1.18 (1.05 – 1.33)‡	1.18 (1.04 – 1.32)‡	1.17 (1.01 – 1.33)‡	-0.02 (-0.19 – 0.16)
	3rd	38.4 (34.2 – 42.4)	23.3 (19.7 – 26.8)	12.3 (7.4 – 20.5)	26.1 (17.3 – 32.1)§	67.9% (46.1 – 80.6)§	1.01 (0.93 – 1.10)	1.01 (0.91 – 1.10)	1.01 (0.90 – 1.11)	-0.01 (-0.10 – 0.08)
	4th	32.2 (27.3 – 37.4)	19.2 (15.7 – 23.2)	9.9 (5.8 – 17.0)	22.3 (14.3 – 28.6)§	69.4% (47.4 – 82.2)§	0.85 (0.74 – 0.98)‡	0.83 (0.71 – 0.96)‡	0.81 (0.66 – 0.96)‡	-0.04 (-0.21 – 0.11)
	5th	24.9 (18.3 – 33.3)	15.3 (11.0 – 20.6)	8.1 (4.5 – 14.4)	16.8 (9.4 – 24.2)§	67.5% (43.9 – 80.9)§	0.66 (0.49 – 0.87)‡	0.66 (0.49 – 0.88)‡	0.66 (0.48 – 0.88)‡	0.01 (-0.13 – 0.14)
The former Yugoslav Republic of Macedonia	1st	50.1 (41.7 – 58.5)	22.7 (18.8 – 27.1)	17.2 (11.9 – 28.1)	32.9 (19.9 – 41.9)§	65.7% (43.3 – 76.1)§	1.35 (1.13 – 1.58)‡	1.42 (1.18 – 1.68)‡	1.41 (1.16 – 1.67)‡	0.06 (-0.17 – 0.30)
	2nd	42.9 (37.7 – 48.1)	18.2 (15.7 – 21.0)	13.9 (9.9 – 22.6)	29.0 (18.9 – 35.1)§	67.5% (46.3 – 76.9)§	1.16 (1.02 – 1.30)‡	1.14 (0.98 – 1.30)	1.14 (0.98 – 1.31)	-0.02 (-0.19 – 0.16)
	3rd	37.0 (33.6 – 40.4)	15.8 (14.1 – 17.6)	12.1 (8.7 – 19.5)	24.9 (16.5 – 29.4)§	67.3% (46.5 – 76.3)§	1.00 (0.91 – 1.08)	0.99 (0.89 – 1.09)	0.99 (0.89 – 1.10)	-0.01 (-0.10 – 0.08)
	4th	30.8 (26.4 – 35.2)	12.6 (10.3 – 15.0)	9.7 (6.7 – 15.9)	21.1 (13.8 – 26.2)§	68.6% (47.4 – 78.5)§	0.83 (0.71 – 0.95)‡	0.79 (0.64 – 0.93)‡	0.79 (0.64 – 0.94)‡	-0.04 (-0.20 – 0.11)
	5th	24.6 (18.3 – 32.4)	10.7 (7.8 – 14.2)	8.1 (5.2 – 13.9)	16.5 (9.3 – 23.2)§	67.0% (44.3 – 77.4)§	0.66 (0.49 – 0.87)‡	0.67 (0.49 – 0.88)‡	0.67 (0.49 – 0.88)‡	0.00 (-0.13 – 0.14)
Timor-Leste	1st	186.4 (152.7 – 223.4)	124.3 (103.5 – 147.4)	61.0 (39.4 – 94.6)	125.4 (82.1 – 165.4)§	67.3% (49.0 – 78.9)§	1.06 (0.90 – 1.24)	1.14 (0.97 – 1.31)	1.23 (1.04 – 1.42)‡	0.16 (-0.02 – 0.34)
	2nd	203.2 (175.2 – 232.6)	126.8 (110.4 – 145.1)	59.8 (39.1 – 91.2)	143.3 (103.7 – 177.9)§	70.5% (54.5 – 80.8)§	1.16 (1.04 – 1.28)‡	1.17 (1.05 – 1.29)‡	1.20 (1.08 – 1.33)‡	0.04 (-0.10 – 0.19)
	3rd	189.6 (167.4 – 213.3)	117.9 (105.0 – 132.2)	51.9 (34.3 – 78.0)	137.7 (104.2 – 166.6)§	72.6% (58.2 – 82.0)§	1.08 (1.00 – 1.16)‡	1.08 (1.01 – 1.16)‡	1.04 (0.96 – 1.12)	-0.04 (-0.12 – 0.04)
	4th	177.5 (150.9 – 206.6)	101.2 (86.8 – 117.7)	43.6 (28.3 – 66.6)	133.9 (100.7 – 165.2)§	75.4% (61.7 – 84.1)§	1.01 (0.90 – 1.14)	0.93 (0.82 – 1.05)	0.88 (0.76 – 0.99)‡	-0.14 (-0.28 – 0.00)
	5th	118.8 (91.7 – 151.7)	73.3 (57.3 – 93.6)	32.2 (20.3 – 51.3)	86.6 (58.1 – 116.7)§	72.9% (57.2 – 82.8)§	0.68 (0.53 – 0.85)‡	0.67 (0.53 – 0.84)‡	0.65 (0.51 – 0.81)‡	-0.03 (-0.16 – 0.09)
Togo	1st	170.1 (149.6 – 192.8)	149.7 (132.9 – 167.6)	99.0 (77.1 – 125.7)	71.0 (38.9 – 100.5)§	41.8% (24.3 – 55.1)§	1.17 (1.06 – 1.30)‡	1.25 (1.15 – 1.36)‡	1.31 (1.17 – 1.46)‡	0.13 (-0.03 – 0.31)
	2nd	170.4 (152.2 – 189.8)	141.5 (126.5 – 157.1)	91.2 (71.9 – 114.7)	79.2 (51.8 – 105.1)§	46.5% (32.1 – 58.2)§	1.18 (1.08 – 1.28)‡	1.19 (1.10 – 1.27)‡	1.20 (1.10 – 1.31)‡	0.03 (-0.11 – 0.16)
	3rd	158.7 (144.3 – 174.0)	131.1 (118.9 – 143.5)	81.4 (64.5 – 101.3)	77.3 (55.3 – 97.6)§	48.7% (35.8 – 59.3)§	1.10 (1.03 – 1.16)‡	1.10 (1.04 – 1.16)‡	1.07 (1.01 – 1.14)‡	-0.02 (-0.10 – 0.05)
	4th	139.6 (123.2 – 157.0)	106.9 (94.8 – 120.0)	66.0 (51.3 – 83.9)	73.6 (51.1 – 94.7)§	52.7% (38.8 – 63.6)§	0.96 (0.87 – 1.06)	0.90 (0.82 – 0.98)‡	0.87 (0.77 – 0.97)‡	-0.09 (-0.22 – 0.03)
	5th	85.2 (72.1 – 99.7)	67.4 (57.7 – 78.4)	40.9 (31.0 – 53.1)	44.3 (28.5 – 59.9)§	52.0% (36.6 – 64.0)§	0.59 (0.51 – 0.68)‡	0.56 (0.50 – 0.64)‡	0.54 (0.46 – 0.64)‡	-0.05 (-0.15 – 0.05)
Tunisia	1st	73.6 (58.9 – 90.2)	42.5 (33.2 – 52.7)	19.0 (12.9 – 27.5)	54.6 (38.5 – 71.6)§	74.2% (61.2 – 82.8)§	1.30 (1.08 – 1.52)‡	1.34 (1.12 – 1.58)‡	1.40 (1.15 – 1.66)‡	0.10 (-0.13 – 0.34)
	2nd	66.2 (55.3 – 77.9)	36.7 (30.0 – 44.0)	15.6 (10.8 – 22.3)	50.6 (38.1 – 63.4)§	76.5% (65.2 – 84.2)§	1.17 (1.03 – 1.30)‡	1.16 (1.02 – 1.30)‡	1.15 (0.99 – 1.31)	-0.02 (-0.19 – 0.15)
	3rd	58.1 (49.6 – 67.5)	31.9 (26.5 – 37.8)	13.6 (9.6 – 19.1)	44.5 (34.2 – 54.8)§	76.6% (65.9 – 84.1)§	1.02 (0.94 – 1.11)	1.01 (0.92 – 1.09)	1.00 (0.90 – 1.10)	-0.02 (-0.11 – 0.07)
	4th	48.9 (40.1 – 59.0)	26.7 (21.5 – 32.9)	10.9 (7.4 – 15.7)	38.0 (28.1 – 48.5)§	77.7% (66.5 – 85.6)§	0.86 (0.75 – 0.98)‡	0.84 (0.72 – 0.97)‡	0.80 (0.65 – 0.95)‡	-0.06 (-0.22 – 0.10)
	5th	37.2 (26.8 – 51.0)	20.7 (14.8 – 28.5)	8.9 (5.7 – 13.6)	28.3 (18.6 – 40.4)§	76.1% (63.9 – 84.3)§	0.65 (0.49 – 0.87)‡	0.65 (0.48 – 0.87)‡	0.65 (0.48 – 0.88)‡	0.00 (-0.13 – 0.13)
Turkey	1st	109.4 (96.7 – 123.8)	59.8 (52.5 – 68.0)	19.6 (16.6 – 22.9)	89.8 (77.4 – 103.6)§	82.0% (78.6 – 85.0)§	1.47 (1.32 – 1.64)‡	1.53 (1.36 – 1.70)‡	1.55 (1.31 – 1.80)‡	0.07 (-0.17 – 0.32)
	2nd	84.0 (74.6 – 93.9)	45.6 (40.5 – 51.2)	14.5 (12.6 – 16.5)	69.5 (60.2 – 79.4)§	82.7% (79.7 – 85.4)§	1.13 (1.03 – 1.24)‡	1.16 (1.05 – 1.27)‡	1.15 (1.00 – 1.30)	0.01 (-0.15 – 0.18)
	3rd	75.3 (68.4 – 82.5)	38.6 (34.9 – 42.6)	12.6 (11.2 – 13.8)	62.7 (56.1 – 69.9)§	83.3% (81.3 – 85.3)§	1.01 (0.94 – 1.08)	0.98 (0.91 – 1.06)	0.99 (0.89 – 1.08)	-0.03 (-0.13 – 0.07)
	4th	62.9 (55.1 – 71.6)	31.6 (27.4 – 36.4)	9.9 (8.1 – 11.8)	53.0 (45.2 – 61.5)§	84.2% (80.8 – 87.3)§	0.85 (0.75 – 0.95)‡	0.81 (0.71 – 0.91)‡	0.78 (0.64 – 0.92)‡	-0.07 (-0.22 – 0.09)
	5th	39.4 (31.3 – 49.0)	20.4 (16.1 – 25.4)	6.8 (5.3 – 8.8)	32.5 (25.3 – 41.1)§	82.6% (78.3 – 86.0)§	0.53 (0.43 – 0.65)‡	0.52 (0.41 – 0.64)‡	0.54 (0.41 – 0.69)‡	0.01 (-0.10 – 0.13)

Continued on next page

‡: Ratio is significantly different from one.
§: Change/decline is significantly different from zero.
Absolute decline: wealth quintile-specific U5MR (1990) - wealth quintile-specific U5MR (2016).
Percentage decline: absolute decline over wealth quintile-specific U5MR (1990) ×100.
Change in ratio: ratio (2016) - ratio (1990).

Table 8 – continued from previous page

	Wealth Quintile	wealth quintile-specific U5MR (deaths per 1000 livebirths) 1990	2000	2016	Absolute decline 1990–2016	Percentage decline 1990–2016	Ratio of wealth quintile-specific to national-level U5MR 1990	2000	2016	Change 1990–2016
Turkmenistan	1st	109.4 (87.2 – 135.9)	106.3 (81.8 – 138.3)	68.6 (28.7 – 147.5)	40.8 (-37.4 – 84.5)	37.3% (-35.2 – 73.3)	1.27 (1.07 – 1.49)‡	1.28 (1.08 – 1.51)‡	1.34 (1.14 – 1.57)‡	0.08 (-0.11 – 0.27)
	2nd	97.5 (80.3 – 116.7)	94.0 (74.2 – 118.6)	58.7 (24.7 – 125.1)	38.8 (-27.6 – 75.4)	39.8% (-28.6 – 74.2)	1.13 (1.01 – 1.25)‡	1.13 (1.00 – 1.26)‡	1.15 (1.02 – 1.28)‡	0.02 (-0.13 – 0.16)
	3rd	89.6 (75.6 – 105.5)	86.1 (69.0 – 107.4)	51.5 (21.8 – 108.3)	38.1 (-19.5 – 69.7)	42.6% (-22.3 – 75.3)	1.04 (0.95 – 1.12)	1.04 (0.95 – 1.11)	1.01 (0.92 – 1.09)	-0.03 (-0.11 – 0.04)
	4th	75.6 (61.6 – 92.0)	72.3 (56.6 – 91.6)	42.4 (17.8 – 89.9)	33.3 (-14.5 – 60.8)	44.0% (-19.6 – 76.6)	0.88 (0.76 – 1.00)‡	0.87 (0.75 – 0.99)‡	0.83 (0.72 – 0.94)‡	-0.05 (-0.18 – 0.08)
	5th	59.3 (42.7 – 79.4)	56.7 (40.2 – 78.9)	33.9 (13.8 – 74.0)	25.4 (-12.5 – 50.5)	42.9% (-22.9 – 75.8)	0.69 (0.52 – 0.89)‡	0.68 (0.52 – 0.89)‡	0.66 (0.50 – 0.86)‡	-0.02 (-0.15 – 0.11)
Uganda	1st	196.1 (175.2 – 218.3)	188.2 (169.9 – 208.0)	67.4 (54.6 – 82.4)	128.6 (104.7 – 153.1)§	65.6% (57.2 – 72.6)§	1.12 (1.02 – 1.23)‡	1.11 (1.02 – 1.20)‡	1.27 (1.12 – 1.43)‡	0.15 (-0.02 – 0.32)
	2nd	186.6 (168.9 – 205.5)	180.1 (163.7 – 197.0)	60.0 (49.3 – 72.2)	126.6 (105.9 – 147.7)§	67.8% (60.4 – 74.0)§	1.07 (0.98 – 1.15)	1.06 (0.98 – 1.14)	1.13 (1.02 – 1.24)‡	0.07 (-0.07 – 0.20)
	3rd	178.0 (164.1 – 193.3)	171.7 (158.1 – 185.6)	52.1 (43.2 – 61.9)	125.8 (110.0 – 142.1)§	70.7% (64.9 – 75.9)§	1.02 (0.96 – 1.07)	1.01 (0.95 – 1.06)	0.98 (0.92 – 1.05)	-0.03 (-0.11 – 0.04)
	4th	181.1 (162.8 – 201.9)	178.2 (161.2 – 195.8)	46.8 (37.9 – 57.0)	134.4 (113.9 – 156.0)§	74.2% (68.0 – 79.5)§	1.03 (0.95 – 1.13)	1.05 (0.97 – 1.13)	0.88 (0.78 – 0.99)‡	-0.15 (-0.28 – -0.02)§
	5th	133.3 (117.5 – 150.8)	132.4 (118.4 – 147.8)	38.7 (30.7 – 47.9)	94.6 (77.2 – 112.7)§	71.0% (63.2 – 77.3)§	0.76 (0.68 – 0.85)‡	0.78 (0.71 – 0.85)‡	0.73 (0.62 – 0.84)‡	-0.03 (-0.16 – 0.10)
Ukraine	1st	26.2 (21.1 – 32.6)	24.8 (20.0 – 30.2)	12.4 (10.1 – 15.0)	13.8 (9.6 – 19.1)§	52.6% (43.0 – 61.2)§	1.35 (1.11 – 1.60)‡	1.35 (1.12 – 1.60)‡	1.36 (1.12 – 1.64)‡	0.01 (-0.18 – 0.21)
	2nd	22.4 (18.8 – 26.9)	21.1 (17.9 – 24.7)	10.5 (9.0 – 12.1)	11.8 (8.7 – 16.0)§	52.9% (44.5 – 61.1)§	1.15 (1.00 – 1.31)‡	1.15 (1.00 – 1.31)‡	1.16 (1.00 – 1.32)	0.00 (-0.14 – 0.14)
	3rd	19.5 (16.9 – 22.9)	18.4 (16.1 – 21.1)	9.1 (8.1 – 10.2)	10.4 (8.0 – 13.5)§	53.2% (46.4 – 60.1)§	1.01 (0.91 – 1.11)	1.01 (0.91 – 1.10)	1.00 (0.90 – 1.11)	0.00 (-0.07 – 0.07)
	4th	15.9 (12.9 – 19.5)	15.0 (12.1 – 18.2)	7.4 (5.9 – 8.8)	8.5 (6.0 – 11.8)§	53.7% (44.3 – 62.5)§	0.82 (0.68 – 0.96)‡	0.82 (0.68 – 0.96)‡	0.81 (0.66 – 0.96)‡	-0.01 (-0.13 – 0.11)
	5th	13.0 (9.4 – 17.8)	12.3 (9.0 – 16.6)	6.1 (4.4 – 8.1)	7.0 (4.4 – 10.5)§	53.4% (42.4 – 63.1)§	0.67 (0.49 – 0.89)‡	0.67 (0.50 – 0.89)‡	0.67 (0.49 – 0.89)‡	0.00 (-0.13 – 0.12)
United Republic of Tanzania	1st	171.9 (152.4 – 193.2)	133.3 (120.4 – 147.3)	63.0 (49.8 – 80.9)	108.9 (84.1 – 132.8)§	63.3% (52.4 – 71.5)§	0.96 (0.86 – 1.06)	1.01 (0.93 – 1.10)	1.11 (0.99 – 1.25)	0.15 (0.00 – 0.30)
	2nd	207.6 (188.5 – 228.4)	148.1 (135.1 – 162.1)	64.2 (50.9 – 81.2)	143.4 (118.3 – 167.1)§	69.1% (60.2 – 75.7)§	1.16 (1.07 – 1.25)‡	1.12 (1.05 – 1.20)‡	1.13 (1.03 – 1.24)‡	-0.03 (-0.17 – 0.10)
	3rd	190.0 (175.5 – 204.7)	140.5 (129.6 – 152.0)	57.9 (46.8 – 72.5)	132.1 (112.5 – 149.5)§	69.5% (61.4 – 75.5)§	1.06 (1.01 – 1.12)‡	1.07 (1.01 – 1.12)‡	1.02 (0.96 – 1.09)	-0.04 (-0.12 – 0.03)
	4th	181.8 (163.9 – 201.5)	127.6 (115.4 – 140.8)	50.8 (40.0 – 64.7)	131.1 (108.9 – 152.8)§	72.1% (63.8 – 78.1)§	1.02 (0.93 – 1.11)	0.97 (0.90 – 1.05)	0.90 (0.80 – 0.99)‡	-0.12 (-0.25 – 0.00)
	5th	142.1 (124.5 – 161.3)	109.4 (97.6 – 122.2)	47.5 (37.3 – 61.2)	94.5 (72.9 – 115.3)§	66.5% (56.2 – 74.3)§	0.80 (0.71 – 0.89)‡	0.83 (0.75 – 0.91)‡	0.84 (0.74 – 0.96)‡	0.04 (-0.08 – 0.18)
Uzbekistan	1st	80.5 (66.4 – 96.7)	70.8 (57.9 – 86.1)	29.5 (23.0 – 37.4)	51.0 (39.0 – 64.3)§	63.4% (55.1 – 70.1)§	1.11 (0.96 – 1.27)	1.12 (0.98 – 1.27)	1.22 (1.04 – 1.43)‡	0.11 (-0.07 – 0.31)
	2nd	83.7 (70.1 – 98.1)	73.3 (60.8 – 88.0)	27.8 (22.0 – 34.2)	55.9 (45.1 – 68.1)§	66.8% (60.4 – 72.6)§	1.16 (1.05 – 1.27)‡	1.16 (1.05 – 1.27)‡	1.15 (1.02 – 1.29)‡	-0.01 (-0.16 – 0.14)
	3rd	74.4 (63.8 – 86.3)	64.3 (54.0 – 76.4)	24.1 (19.5 – 29.2)	50.3 (42.0 – 59.6)§	67.7% (62.5 – 72.3)§	1.03 (0.95 – 1.10)	1.02 (0.95 – 1.10)	1.00 (0.91 – 1.08)	-0.03 (-0.11 – 0.05)
	4th	63.5 (52.9 – 75.8)	55.0 (45.0 – 66.9)	20.0 (15.6 – 25.1)	43.5 (34.7 – 53.9)§	68.5% (61.6 – 74.5)§	0.88 (0.78 – 0.99)‡	0.87 (0.77 – 0.98)‡	0.83 (0.71 – 0.95)‡	-0.05 (-0.18 – 0.08)
	5th	59.4 (47.3 – 73.9)	51.5 (40.7 – 65.2)	19.2 (14.2 – 25.2)	40.3 (30.6 – 51.7)§	67.8% (60.0 – 74.4)§	0.82 (0.69 – 0.98)‡	0.82 (0.69 – 0.97)‡	0.79 (0.64 – 0.98)‡	-0.03 (-0.18 – 0.13)
Vanuatu	1st	46.8 (36.6 – 58.8)	38.0 (30.0 – 47.5)	36.6 (23.5 – 57.5)	10.2 (-11.2 – 26.3)	21.8% (-26.1 – 50.6)	1.32 (1.10 – 1.54)‡	1.32 (1.11 – 1.55)‡	1.33 (1.10 – 1.57)‡	0.01 (-0.18 – 0.21)
	2nd	41.6 (34.0 – 50.9)	33.4 (27.2 – 40.4)	32.1 (20.9 – 49.4)	9.5 (-8.9 – 23.3)	22.8% (-23.1 – 51.1)	1.17 (1.04 – 1.31)‡	1.16 (1.03 – 1.30)‡	1.16 (1.02 – 1.30)‡	-0.01 (-0.16 – 0.13)
	3rd	36.0 (29.8 – 43.3)	29.0 (24.1 – 34.5)	27.9 (18.3 – 42.5)	8.1 (-7.6 – 19.7)	22.4% (-22.0 – 50.4)	1.01 (0.93 – 1.10)	1.01 (0.92 – 1.10)	1.01 (0.92 – 1.09)	0.00 (-0.07 – 0.07)
	4th	30.2 (24.2 – 37.5)	24.4 (19.5 – 30.0)	23.4 (15.1 – 36.5)	6.8 (-6.8 – 17.2)	22.5% (-25.1 – 51.5)	0.85 (0.74 – 0.98)‡	0.85 (0.73 – 0.98)‡	0.85 (0.73 – 0.98)‡	0.00 (-0.13 – 0.13)
	5th	23.0 (16.1 – 31.9)	18.6 (13.1 – 25.5)	18.0 (10.8 – 29.6)	5.0 (-5.8 – 13.9)	21.7% (-29.1 – 51.6)	0.65 (0.47 – 0.85)‡	0.65 (0.48 – 0.86)‡	0.65 (0.48 – 0.86)‡	0.00 (-0.11 – 0.12)
Viet Nam	1st	72.4 (61.7 – 84.4)	43.9 (35.3 – 53.6)	31.6 (25.3 – 39.8)	40.8 (29.0 – 52.7)§	56.4% (44.6 – 65.4)§	1.42 (1.24 – 1.61)‡	1.46 (1.28 – 1.66)‡	1.46 (1.25 – 1.69)‡	0.04 (-0.18 – 0.26)
	2nd	58.9 (51.1 – 67.1)	34.3 (27.9 – 41.1)	24.6 (20.2 – 30.5)	34.3 (25.4 – 42.9)§	58.3% (47.4 – 66.5)§	1.16 (1.03 – 1.28)‡	1.14 (1.02 – 1.27)‡	1.14 (1.00 – 1.27)‡	-0.02 (-0.17 – 0.14)
	3rd	51.3 (45.8 – 57.0)	29.7 (24.3 – 35.3)	21.4 (18.0 – 26.0)	29.8 (23.1 – 36.0)§	58.2% (48.6 – 65.3)§	1.00 (0.93 – 1.08)	0.99 (0.91 – 1.07)	0.99 (0.90 – 1.08)	-0.01 (-0.10 – 0.07)
	4th	42.0 (35.9 – 48.5)	24.4 (19.5 – 29.8)	17.4 (14.0 – 21.8)	24.6 (17.6 – 31.4)§	58.5% (46.8 – 67.5)§	0.82 (0.72 – 0.93)‡	0.81 (0.71 – 0.92)‡	0.81 (0.68 – 0.93)‡	-0.02 (-0.16 – 0.12)
	5th	30.4 (23.5 – 38.6)	17.7 (13.2 – 23.3)	13.0 (9.7 – 17.6)	17.5 (11.3 – 24.2)§	57.4% (43.6 – 67.1)§	0.60 (0.47 – 0.74)‡	0.59 (0.47 – 0.74)‡	0.60 (0.46 – 0.77)‡	0.00 (-0.11 – 0.13)
Yemen	1st	160.3 (135.9 – 187.4)	127.1 (108.2 – 147.8)	76.1 (52.9 – 107.6)	84.1 (49.3 – 116.0)§	52.5% (32.7 – 66.9)§	1.27 (1.09 – 1.48)‡	1.34 (1.17 – 1.52)‡	1.38 (1.18 – 1.59)‡	0.10 (-0.09 – 0.30)
	2nd	142.2 (124.6 – 160.5)	107.4 (93.8 – 122.2)	64.4 (45.1 – 90.0)	77.8 (48.1 – 103.4)§	54.7% (35.8 – 68.4)§	1.13 (1.01 – 1.25)‡	1.13 (1.01 – 1.24)‡	1.17 (1.04 – 1.29)‡	0.03 (-0.11 – 0.18)
	3rd	133.1 (120.5 – 146.0)	99.9 (89.2 – 110.9)	56.4 (40.1 – 78.3)	76.7 (52.0 – 96.4)§	57.6% (40.5 – 70.0)§	1.06 (0.98 – 1.13)	1.05 (0.97 – 1.12)	1.02 (0.94 – 1.10)	-0.04 (-0.12 – 0.04)
	4th	116.7 (101.0 – 133.8)	84.3 (72.9 – 97.0)	47.3 (33.1 – 66.9)	69.4 (46.1 – 90.1)§	59.5% (42.1 – 71.9)§	0.93 (0.82 – 1.05)	0.89 (0.78 – 0.99)‡	0.85 (0.75 – 0.97)‡	-0.07 (-0.20 – 0.06)
	5th	76.7 (59.3 – 97.7)	56.8 (44.1 – 72.1)	32.3 (21.0 – 48.9)	44.4 (26.1 – 63.0)§	57.9% (39.1 – 71.4)§	0.61 (0.47 – 0.77)‡	0.60 (0.47 – 0.75)‡	0.58 (0.45 – 0.74)‡	-0.03 (-0.14 – 0.09)
Zambia	1st	196.4 (176.6 – 218.2)	172.1 (155.2 – 191.4)	76.8 (55.0 – 104.9)	119.6 (86.1 – 149.2)§	60.9% (46.2 – 72.2)§	1.08 (0.99 – 1.17)	1.07 (0.99 – 1.15)	1.21 (1.08 – 1.35)‡	0.13 (-0.02 – 0.29)
	2nd	199.3 (180.8 – 219.2)	174.3 (158.4 – 191.9)	71.5 (51.7 – 97.3)	127.9 (97.8 – 154.9)§	64.2% (51.1 – 74.3)§	1.09 (1.01 – 1.17)‡	1.09 (1.02 – 1.16)‡	1.13 (1.03 – 1.23)‡	0.03 (-0.09 – 0.16)
	3rd	188.2 (173.7 – 203.4)	168.2 (154.7 – 182.9)	64.5 (46.8 – 87.2)	123.7 (98.4 – 145.7)§	65.7% (53.7 – 75.2)§	1.03 (0.98 – 1.08)	1.05 (1.00 – 1.10)	1.02 (0.96 – 1.08)	-0.02 (-0.09 – 0.06)
	4th	187.5 (169.0 – 207.6)	161.9 (145.9 – 180.2)	55.8 (40.0 – 76.3)	131.7 (104.6 – 156.1)§	70.3% (58.8 – 78.8)§	1.03 (0.95 – 1.12)	1.01 (0.93 – 1.09)	0.88 (0.79 – 0.98)‡	-0.15 (-0.27 – -0.03)§
	5th	139.6 (123.6 – 157.1)	126.5 (112.7 – 141.7)	48.5 (34.5 – 66.8)	91.1 (68.2 – 112.3)§	65.3% (51.7 – 75.5)§	0.77 (0.69 – 0.85)‡	0.79 (0.72 – 0.86)‡	0.76 (0.66 – 0.87)‡	0.00 (-0.12 – 0.12)

Continued on next page

‡: Ratio is significantly different from one.
§: Change/decline is significantly different from zero.
Absolute decline: wealth quintile-specific U5MR (1990) - wealth quintile-specific U5MR (2016).
Percentage decline: absolute decline over wealth quintile-specific U5MR (1990) ×100.
Change in ratio: ratio (2016) - ratio (1990).

Table 8 – continued from previous page

	Wealth Quintile	wealth quintile-specific U5MR (deaths per 1000 livebirths) 1990	2000	2016	Absolute decline 1990–2016	Percentage decline 1990–2016	Ratio of wealth quintile-specific to national-level U5MR 1990	2000	2016	Change 1990–2016
Zimbabwe	1st	82.4 (71.3 – 94.1)	106.8 (93.9 – 120.8)	67.9 (51.0 – 88.9)	14.5 (-8.1 – 33.4)	17.6% (-10.2 – 38.6)	1.10 (0.98 – 1.22)	1.10 (1.00 – 1.21)‡	1.20 (1.07 – 1.34)‡	0.11 (-0.04 – 0.27)
	2nd	83.3 (73.5 – 94.0)	105.6 (93.7 – 117.9)	64.4 (48.7 – 83.0)	19.0 (-1.0 – 37.2)	22.7% (-1.2 – 42.4)	1.11 (1.01 – 1.20)‡	1.09 (1.00 – 1.18)‡	1.14 (1.04 – 1.25)‡	0.03 (-0.10 – 0.16)
	3rd	78.7 (70.7 – 87.1)	102.6 (92.5 – 113.4)	57.9 (44.4 – 74.1)	20.9 (3.8 – 35.7)§	26.5% (4.9 – 43.7)§	1.05 (0.98 – 1.11)	1.06 (1.00 – 1.12)	1.03 (0.96 – 1.09)	-0.02 (-0.09 – 0.05)
	4th	71.7 (62.9 – 81.4)	91.6 (81.1 – 103.5)	49.9 (37.6 – 65.0)	21.8 (5.2 – 36.4)§	30.4% (7.7 – 48.1)§	0.95 (0.86 – 1.05)	0.95 (0.87 – 1.03)	0.88 (0.79 – 0.98)‡	-0.07 (-0.19 – 0.05)
	5th	59.8 (50.9 – 69.7)	77.4 (66.8 – 89.2)	41.9 (31.0 – 55.0)	17.9 (3.5 – 31.5)§	29.9% (6.3 – 48.7)§	0.80 (0.70 – 0.91)‡	0.80 (0.71 – 0.89)‡	0.74 (0.64 – 0.85)‡	-0.05 (-0.18 – 0.07)

‡: Ratio is significantly different from one.
§: Change/decline is significantly different from zero.
Absolute decline: wealth quintile-specific U5MR (1990) - wealth quintile-specific U5MR (2016).
Percentage decline: absolute decline over wealth quintile-specific U5MR (1990) $\times 100$.
Change in ratio: ratio (2016) - ratio (1990).

Table 9: **Number of wealth quintile-specific under-5 deaths, by wealth quintile, for all the low-income and middle-income countries (excluding China) combined, and by region and the 99 countries with empirical data.** Estimates and 90% uncertainty intervals for (i) number of wealth quintile-specific under-5 deaths in 1990, 2000, and 2016; (ii) the absolute and percentage declines in wealth quintile-specific under-5 deaths from 1990 to 2016; (iii) the percentage of wealth quintile-specific under-5 deaths among all under-5 deaths in 1990, 2000, and 2016; by wealth quintile, for the 99 countries with empirical data. Numbers in brackets are 90% uncertainty intervals. The share of total death by wealth quintile may not add up to 100% due to rounding. Countries are ordered alphabetically.

	Wealth Quintile	number of wealth quintile-specific under-5 deaths 1990	2000	2016	Absolute decline 1990–2016	Percentage decline 1990–2016	Share of total death (in percentage) 1990	2000	2016
All low-income and middle-income countries (excluding	1st	2,740,000 (2,670,000 – 2,810,000)	2,280,000 (2,220,000 – 2,350,000)	1,410,000 (1,330,000 – 1,530,000)	1,330,000 (1,190,000 – 1,430,000)	48.6%	24.8	24.9	26.1
	2nd	2,620,000 (2,550,000 – 2,680,000)	2,160,000 (2,100,000 – 2,220,000)	1,270,000 (1,200,000 – 1,380,000)	1,350,000 (1,230,000 – 1,440,000)	51.6%	23.7	23.6	23.4
	3rd	2,320,000 (2,270,000 – 2,380,000)	1,930,000 (1,890,000 – 1,970,000)	1,120,000 (1,060,000 – 1,210,000)	1,200,000 (1,100,000 – 1,280,000)	51.7%	21.0	21.0	20.7
	4th	2,000,000 (1,950,000 – 2,060,000)	1,650,000 (1,610,000 – 1,700,000)	928,000 (878,000 – 1,010,000)	1,080,000 (983,000 – 1,150,000)	53.9%	18.2	18.0	17.2
	5th	1,350,000 (1,310,000 – 1,400,000)	1,150,000 (1,110,000 – 1,180,000)	684,000 (645,000 – 746,000)	667,000 (595,000 – 717,000)	49.4%	12.2	12.5	12.7
South Asia	1st	1,230,000 (1,170,000 – 1,280,000)	939,000 (888,000 – 989,000)	468,000 (413,000 – 526,000)	759,000 (679,000 – 835,000)	61.9%	25.9	26.6	27.3
	2nd	1,170,000 (1,120,000 – 1,230,000)	862,000 (817,000 – 910,000)	413,000 (368,000 – 463,000)	760,000 (686,000 – 830,000)	64.8%	24.8	24.5	24.1
	3rd	1,000,000 (960,000 – 1,040,000)	744,000 (711,000 – 780,000)	353,000 (317,000 – 390,000)	647,000 (593,000 – 699,000)	64.7%	21.1	21.1	20.6
	4th	820,000 (781,000 – 861,000)	588,000 (554,000 – 625,000)	286,000 (252,000 – 324,000)	534,000 (480,000 – 586,000)	65.1%	17.3	16.7	16.7
	5th	511,000 (484,000 – 540,000)	389,000 (364,000 – 416,000)	194,000 (169,000 – 226,000)	317,000 (276,000 – 354,000)	62.1%	10.8	11.0	11.3
Eastern Europe and Central Asia	1st	86,900 (81,500 – 93,000)	50,700 (47,400 – 54,600)	23,700 (21,200 – 27,800)	63,200 (57,300 – 68,700)	72.7%	26.3	26.4	27.0
	2nd	75,800 (72,000 – 80,200)	44,400 (41,800 – 47,400)	20,200 (18,100 – 23,600)	55,600 (51,000 – 59,600)	73.3%	22.9	23.1	23.0
	3rd	67,400 (64,400 – 70,800)	38,700 (36,700 – 41,000)	17,500 (15,800 – 20,300)	49,900 (46,300 – 53,000)	74.0%	20.4	20.2	20.0
	4th	56,600 (53,300 – 60,400)	32,200 (30,100 – 34,600)	14,300 (12,500 – 16,900)	42,300 (38,600 – 45,800)	74.8%	17.1	16.8	16.2
	5th	44,100 (40,100 – 48,800)	26,000 (23,500 – 28,800)	12,100 (10,500 – 14,500)	32,100 (28,200 – 35,800)	72.8%	13.3	13.5	13.7
Eastern and Southern Africa	1st	391,000 (374,000 – 410,000)	410,000 (396,000 – 427,000)	264,000 (243,000 – 300,000)	127,000 (88,000 – 153,000)	32.5%	21.1	21.6	24.0
	2nd	415,000 (399,000 – 432,000)	419,000 (405,000 – 435,000)	253,000 (233,000 – 286,000)	162,000 (127,000 – 186,000)	39.1%	22.4	22.1	22.9
	3rd	390,000 (378,000 – 405,000)	399,000 (386,000 – 413,000)	226,000 (209,000 – 255,000)	164,000 (134,000 – 185,000)	42.0%	21.1	21.1	20.5
	4th	374,000 (358,000 – 391,000)	377,000 (363,000 – 392,000)	196,000 (180,000 – 223,000)	178,000 (148,000 – 199,000)	47.6%	20.2	19.9	17.8
	5th	281,000 (267,000 – 297,000)	289,000 (277,000 – 303,000)	163,000 (149,000 – 187,000)	117,000 (91,100 – 136,000)	41.7%	15.2	15.3	14.8
West and Central Africa	1st	469,000 (443,000 – 497,000)	524,000 (501,000 – 549,000)	445,000 (387,000 – 525,000)	24,400 (-61,500 – 86,200)	5.2%	23.0	23.2	25.3
	2nd	475,000 (452,000 – 500,000)	533,000 (509,000 – 557,000)	409,000 (358,000 – 483,000)	65,700 (-11,600 – 121,000)	13.8%	23.3	23.6	23.3
	3rd	438,000 (419,000 – 457,000)	482,000 (464,000 – 502,000)	372,000 (326,000 – 436,000)	65,600 (660 – 113,000)	15.0%	21.4	21.4	21.2
	4th	396,000 (375,000 – 419,000)	433,000 (413,000 – 453,000)	308,000 (270,000 – 362,000)	88,200 (30,400 – 131,000)	22.2%	19.4	19.2	17.6
	5th	264,000 (249,000 – 280,000)	284,000 (271,000 – 299,000)	222,000 (195,000 – 260,000)	42,000 (1,770 – 71,600)	15.9%	12.9	12.6	12.6
Latin America and Caribbean	1st	175,000 (162,000 – 189,000)	107,000 (98,500 – 115,000)	51,600 (46,300 – 58,600)	124,000 (111,000 – 137,000)	70.7%	27.2	27.8	28.0
	2nd	150,000 (141,000 – 159,000)	89,200 (83,700 – 95,000)	42,700 (38,800 – 47,800)	107,000 (97,800 – 116,000)	71.3%	23.3	23.3	23.2
	3rd	131,000 (124,000 – 137,000)	76,700 (72,700 – 81,000)	36,900 (33,900 – 40,600)	94,000 (87,300 – 100,000)	71.8%	20.3	20.0	20.0
	4th	109,000 (101,000 – 117,000)	63,300 (58,500 – 68,100)	29,900 (26,300 – 34,100)	79,000 (70,700 – 87,000)	72.6%	16.9	16.5	16.2
	5th	79,000 (69,400 – 90,300)	47,200 (41,500 – 53,700)	23,300 (20,100 – 27,600)	55,600 (47,200 – 64,800)	70.4%	12.3	12.3	12.7
East Asia and Pacific (excluding China)	1st	248,000 (233,000 – 264,000)	169,000 (159,000 – 179,000)	96,800 (85,600 – 112,000)	151,000 (130,000 – 170,000)	60.9%	27.4	28.0	28.8
	2nd	207,000 (196,000 – 218,000)	139,000 (132,000 – 147,000)	77,400 (68,800 – 89,500)	129,000 (114,000 – 142,000)	62.3%	22.8	23.1	23.1
	3rd	187,000 (179,000 – 196,000)	123,000 (117,000 – 129,000)	67,300 (60,000 – 77,400)	120,000 (108,000 – 130,000)	64.0%	20.7	20.4	20.0
	4th	157,000 (147,000 – 167,000)	102,000 (95,400 – 108,000)	55,400 (48,600 – 64,700)	102,000 (88,900 – 113,000)	65.0%	17.3	16.9	16.5
	5th	106,000 (96,200 – 117,000)	69,400 (62,900 – 76,600)	38,900 (33,600 – 46,100)	67,400 (56,800 – 77,400)	63.4%	11.7	11.5	11.6
Middle East and North Africa	1st	140,000 (132,000 – 148,000)	84,700 (79,800 – 89,600)	61,200 (54,300 – 70,900)	78,300 (66,600 – 88,100)	56.1%	26.5	27.0	27.2
	2nd	122,000 (116,000 – 128,000)	72,100 (68,500 – 76,000)	51,600 (46,000 – 59,300)	69,900 (60,300 – 77,800)	57.5%	23.1	22.9	22.9
	3rd	108,000 (104,000 – 113,000)	63,500 (60,700 – 66,500)	45,100 (40,400 – 51,600)	63,200 (55,700 – 69,000)	58.4%	20.6	20.2	20.0
	4th	91,700 (86,500 – 97,200)	54,200 (50,900 – 57,600)	37,900 (33,400 – 44,100)	53,800 (46,000 – 60,100)	58.7%	17.4	17.2	16.8
	5th	65,400 (59,300 – 72,100)	39,800 (36,300 – 43,500)	29,700 (26,000 – 34,900)	35,700 (28,800 – 42,000)	54.6%	12.4	12.7	13.2

Continued on next page

Absolute decline: wealth quintile-specific under-5 deaths (1990) - wealth quintile-specific under-5 deaths (2016).
Percentage decline: absolute decline over wealth quintile-specific under-5 deaths (1990) ×100.
Share of total death (in percentage): wealth quintile-specific under-5 deaths over the total under-5 deaths × 100.

Table 9 – continued from previous page

	Wealth Quintile	number of wealth quintile-specific under-5 deaths 1990	2000	2016	Absolute decline 1990–2016	Percentage decline 1990–2016	Share of total death (in percentage) 1990	2000	2016
	1st	24,600 (20,900 – 28,900)	29,300 (25,600 – 33,500)	20,500 (16,000 – 25,300)	4,030 (-1,720 – 9,880)	16.4%	22.8	24.1	25.7
	2nd	24,400 (21,400 – 27,700)	27,500 (24,500 – 30,700)	18,300 (14,400 – 22,500)	6,100 (1,200 – 11,100)	25.0%	22.6	22.6	23.0
Afghanistan	3rd	23,000 (20,600 – 25,500)	25,900 (23,500 – 28,300)	16,600 (13,200 – 20,100)	6,380 (2,250 – 10,500)	27.8%	21.3	21.2	20.8
	4th	21,700 (18,700 – 25,000)	23,200 (20,400 – 26,300)	14,100 (11,100 – 17,400)	7,580 (3,370 – 11,800)	35.0%	20.1	19.0	17.6
	5th	14,300 (11,800 – 17,200)	16,000 (13,700 – 18,700)	10,300 (7,920 – 13,100)	3,970 (712 – 7,510)	27.8%	13.2	13.1	12.9
	1st	783 (628 – 948)	346 (272 – 430)	122 (64 – 228)	660 (480 – 826)	84.3%	24.7	25.1	26.2
	2nd	751 (634 – 880)	324 (266 – 394)	109 (58 – 200)	642 (493 – 777)	85.5%	23.7	23.5	23.3
Albania	3rd	646 (555 – 741)	281 (233 – 335)	95 (50 – 174)	552 (433 – 651)	85.4%	20.4	20.4	20.3
	4th	546 (450 – 649)	236 (190 – 290)	76 (40 – 140)	469 (355 – 575)	86.0%	17.2	17.1	16.3
	5th	439 (324 – 580)	192 (140 – 262)	65 (33 – 128)	374 (255 – 501)	85.1%	13.9	13.9	13.9
	1st	10,600 (8,970 – 12,400)	6,470 (5,580 – 7,410)	6,380 (5,380 – 7,480)	4,260 (2,600 – 5,990)	40.1%	26.0	26.3	26.9
	2nd	9,390 (8,160 – 10,600)	5,590 (4,980 – 6,220)	5,320 (4,620 – 6,070)	4,070 (2,780 – 5,350)	43.3%	23.0	22.8	22.5
Algeria	3rd	8,160 (7,310 – 9,010)	4,860 (4,440 – 5,260)	4,650 (4,140 – 5,190)	3,510 (2,660 – 4,370)	43.0%	19.9	19.8	19.6
	4th	6,950 (5,950 – 8,040)	4,180 (3,640 – 4,750)	3,970 (3,370 – 4,650)	2,980 (1,920 – 4,080)	42.9%	17.0	17.0	16.8
	5th	5,770 (4,490 – 7,320)	3,470 (2,780 – 4,310)	3,370 (2,640 – 4,250)	2,400 (1,350 – 3,610)	41.6%	14.1	14.1	14.2
	1st	28,500 (24,200 – 33,700)	33,600 (28,300 – 39,800)	23,000 (11,300 – 41,400)	5,600 (-12,400 – 17,900)	19.6%	21.0	21.0	23.9
	2nd	30,600 (26,400 – 35,200)	35,900 (30,900 – 41,700)	21,900 (10,900 – 39,400)	8,630 (-8,910 – 20,500)	28.2%	22.5	22.5	22.9
Angola	3rd	28,800 (25,200 – 32,700)	33,800 (29,400 – 38,900)	20,000 (9,980 – 35,100)	8,770 (-6,740 – 19,400)	30.5%	21.1	21.2	20.8
	4th	27,200 (23,200 – 31,500)	31,800 (27,000 – 37,100)	16,900 (8,380 – 30,100)	10,300 (-3,450 – 19,600)	37.9%	20.0	19.9	17.6
	5th	21,000 (17,300 – 25,600)	24,700 (20,400 – 29,900)	14,200 (6,970 – 25,600)	6,840 (-4,590 – 14,900)	32.6%	15.4	15.5	14.8
	1st	1,000 (824 – 1,190)	329 (271 – 392)	149 (107 – 208)	851 (676 – 1,040)	85.1%	26.3	27.0	27.9
	2nd	888 (759 – 1,020)	283 (242 – 327)	123 (91 – 168)	765 (635 – 902)	86.2%	23.4	23.2	23.0
Armenia	3rd	767 (675 – 864)	243 (212 – 275)	106 (79 – 143)	661 (566 – 762)	86.2%	20.2	19.9	19.9
	4th	645 (543 – 757)	204 (171 – 241)	85 (61 – 118)	559 (458 – 670)	86.7%	17.0	16.8	16.0
	5th	496 (368 – 658)	159 (117 – 211)	71 (47 – 105)	425 (307 – 572)	85.7%	13.1	13.1	13.2
	1st	4,610 (3,850 – 5,440)	2,600 (2,170 – 3,100)	1,390 (811 – 2,390)	3,220 (2,020 – 4,170)	69.8%	23.9	24.3	25.9
	2nd	4,400 (3,800 – 5,050)	2,490 (2,120 – 2,880)	1,260 (749 – 2,130)	3,140 (2,120 – 3,920)	71.4%	22.8	23.2	23.5
Azerbaijan	3rd	4,110 (3,630 – 4,620)	2,250 (1,960 – 2,570)	1,090 (652 – 1,840)	3,020 (2,130 – 3,670)	73.5%	21.3	21.0	20.3
	4th	3,540 (3,030 – 4,110)	1,950 (1,650 – 2,300)	928 (547 – 1,580)	2,610 (1,780 – 3,280)	73.7%	18.4	18.2	17.3
	5th	2,620 (2,020 – 3,340)	1,430 (1,090 – 1,830)	698 (396 – 1,240)	1,930 (1,210 – 2,610)	73.5%	13.6	13.3	13.0
	1st	128,000 (118,000 – 138,000)	80,500 (74,100 – 87,000)	28,000 (24,100 – 32,400)	100,000 (89,500 – 110,000)	78.1%	24.1	25.5	26.5
	2nd	123,000 (115,000 – 132,000)	71,700 (66,000 – 77,200)	24,700 (21,500 – 28,300)	98,200 (89,100 – 108,000)	79.9%	23.1	22.7	23.4
Bangladesh	3rd	111,000 (105,000 – 118,000)	65,700 (61,800 – 69,600)	21,300 (18,900 – 23,900)	90,000 (83,400 – 96,600)	80.8%	20.9	20.8	20.1
	4th	98,900 (91,000 – 107,000)	56,200 (51,500 – 61,300)	18,000 (15,500 – 20,900)	80,900 (72,500 – 89,500)	81.8%	18.6	17.8	17.1
	5th	71,200 (64,600 – 78,000)	41,800 (37,800 – 45,900)	13,700 (11,500 – 16,300)	57,400 (50,700 – 64,300)	80.6%	13.4	13.2	13.0
	1st	623 (510 – 743)	321 (263 – 383)	122 (99 – 147)	500 (404 – 606)	80.3%	27.5	27.6	27.5
	2nd	523 (450 – 599)	268 (230 – 306)	102 (87 – 118)	421 (355 – 489)	80.5%	23.1	23.0	23.0
Belarus	3rd	456 (407 – 503)	234 (208 – 257)	89 (78 – 99)	366 (325 – 407)	80.3%	20.1	20.1	20.1
	4th	365 (298 – 434)	187 (152 – 223)	71 (57 – 85)	294 (235 – 356)	80.5%	16.1	16.1	16.1
	5th	301 (220 – 401)	154 (113 – 204)	59 (43 – 79)	242 (175 – 326)	80.5%	13.3	13.2	13.3
	1st	68 (54 – 83)	47 (39 – 56)	34 (27 – 41)	34 (21 – 48)	50.4%	26.3	26.9	27.5
	2nd	60 (50 – 71)	40 (35 – 46)	28 (24 – 33)	32 (22 – 43)	53.1%	23.4	23.1	23.1
Belize	3rd	52 (45 – 60)	35 (31 – 39)	25 (21 – 28)	27 (20 – 35)	51.9%	20.2	20.1	20.1
	4th	44 (36 – 53)	29 (25 – 34)	20 (16 – 24)	24 (16 – 33)	55.2%	16.9	16.7	16.2
	5th	34 (24 – 46)	23 (17 – 31)	16 (12 – 22)	18 (11 – 27)	53.4%	13.1	13.2	13.2

Continued on next page

Absolute decline: wealth quintile-specific under-5 deaths (1990) - wealth quintile-specific under-5 deaths (2016).
Percentage decline: absolute decline over wealth quintile-specific under-5 deaths (1990) $\times$100.
Share of total death (in percentage): wealth quintile-specific under-5 deaths over the total under-5 deaths $\times$ 100.

Table 9 – continued from previous page

	Wealth Quintile	number of wealth quintile-specific under-5 deaths 1990	2000	2016	Absolute decline 1990–2016	Percentage decline 1990–2016	Share of total death (in percentage) 1990	2000	2016
Benin	1st	8,700 (7,740 – 9,730)	9,210 (8,280 – 10,200)	9,110 (6,750 – 12,600)	-413 (-3,930 – 2,150)	-4.8%	22.3	22.8	23.9
	2nd	8,740 (7,900 – 9,680)	9,070 (8,180 – 10,000)	8,770 (6,560 – 12,000)	-26 (-3,190 – 2,360)	-0.3%	22.4	22.4	23.0
	3rd	8,450 (7,760 – 9,190)	8,850 (8,100 – 9,680)	8,230 (6,220 – 11,200)	222 (-2,650 – 2,300)	2.6%	21.7	21.9	21.6
	4th	7,540 (6,750 – 8,400)	7,640 (6,880 – 8,480)	6,780 (5,080 – 9,320)	759 (-1,770 – 2,640)	10.1%	19.3	18.9	17.8
	5th	5,590 (4,870 – 6,420)	5,640 (4,990 – 6,380)	5,230 (3,820 – 7,350)	353 (-1,740 – 1,950)	6.3%	14.3	14.0	13.7
Bhutan	1st	663 (541 – 807)	347 (289 – 413)	136 (92 – 192)	528 (401 – 672)	79.6%	25.8	27.5	29.0
	2nd	580 (491 – 687)	287 (247 – 332)	108 (75 – 151)	471 (373 – 586)	81.3%	22.5	22.8	23.1
	3rd	540 (470 – 627)	260 (229 – 293)	93 (65 – 129)	447 (367 – 538)	82.7%	21.0	20.6	19.9
	4th	472 (397 – 567)	217 (184 – 254)	77 (53 – 109)	395 (313 – 491)	83.7%	18.4	17.2	16.5
	5th	316 (234 – 422)	150 (111 – 196)	54 (35 – 83)	261 (185 – 360)	82.7%	12.3	11.9	11.6
Bolivia (Plurinational State of)	1st	8,130 (7,370 – 8,910)	5,790 (5,230 – 6,380)	2,770 (1,900 – 3,920)	5,360 (4,010 – 6,500)	65.9%	27.6	28.5	29.9
	2nd	6,800 (6,190 – 7,470)	4,880 (4,410 – 5,370)	2,210 (1,530 – 3,130)	4,600 (3,540 – 5,520)	67.6%	23.1	24.0	23.8
	3rd	6,210 (5,730 – 6,690)	4,180 (3,820 – 4,550)	1,860 (1,290 – 2,610)	4,360 (3,510 – 5,070)	70.2%	21.1	20.6	20.0
	4th	5,290 (4,750 – 5,870)	3,440 (3,060 – 3,840)	1,520 (1,020 – 2,160)	3,770 (2,980 – 4,500)	71.3%	17.9	16.9	16.3
	5th	3,030 (2,630 – 3,460)	2,020 (1,760 – 2,310)	936 (629 – 1,380)	2,090 (1,550 – 2,590)	69.0%	10.3	9.9	10.1
Brazil	1st	70,100 (60,100 – 80,800)	38,300 (32,800 – 44,500)	13,600 (10,400 – 17,500)	56,500 (46,700 – 66,700)	80.6%	29.3	30.1	30.5
	2nd	55,200 (48,500 – 62,000)	29,300 (25,600 – 33,100)	10,100 (7,980 – 12,700)	45,000 (38,200 – 52,000)	81.6%	23.0	23.0	22.7
	3rd	47,900 (43,100 – 52,800)	24,900 (22,200 – 27,600)	8,750 (7,020 – 10,800)	39,200 (34,100 – 44,100)	81.8%	20.0	19.6	19.6
	4th	39,300 (33,500 – 45,300)	20,400 (17,400 – 23,600)	6,920 (5,250 – 8,940)	32,300 (26,600 – 38,400)	82.3%	16.4	16.0	15.5
	5th	27,100 (20,700 – 34,900)	14,300 (10,800 – 18,400)	5,150 (3,590 – 7,180)	22,000 (16,300 – 28,800)	81.1%	11.3	11.2	11.6
Burkina Faso	1st	16,600 (14,900 – 18,300)	19,400 (17,500 – 21,300)	14,200 (10,800 – 18,700)	2,360 (-2,210 – 5,990)	14.2%	21.0	21.0	23.9
	2nd	17,600 (16,000 – 19,300)	21,100 (19,200 – 23,200)	13,800 (10,500 – 18,100)	3,770 (-592 – 7,340)	21.5%	22.2	22.9	23.1
	3rd	17,000 (15,700 – 18,500)	19,700 (18,200 – 21,400)	12,500 (9,610 – 16,200)	4,500 (826 – 7,540)	26.4%	21.5	21.4	21.0
	4th	16,200 (14,700 – 17,900)	18,600 (16,800 – 20,600)	10,700 (8,120 – 14,000)	5,520 (2,020 – 8,460)	34.0%	20.5	20.2	17.9
	5th	11,700 (10,400 – 13,000)	13,400 (12,000 – 15,000)	8,380 (6,200 – 11,200)	3,290 (358 – 5,720)	28.2%	14.8	14.6	14.1
Burundi	1st	10,700 (9,070 – 12,600)	9,980 (8,560 – 11,700)	8,130 (6,080 – 10,700)	2,590 (-149 – 5,070)	24.2%	24.1	24.7	26.3
	2nd	9,920 (8,580 – 11,300)	8,990 (7,880 – 10,300)	7,060 (5,420 – 9,200)	2,860 (431 – 4,880)	28.8%	22.3	22.2	22.9
	3rd	9,380 (8,290 – 10,500)	8,540 (7,600 – 9,590)	6,370 (4,960 – 8,160)	3,000 (978 – 4,700)	32.0%	21.1	21.1	20.7
	4th	8,600 (7,360 – 9,950)	7,640 (6,610 – 8,760)	5,340 (4,070 – 6,950)	3,260 (1,300 – 5,000)	37.9%	19.3	18.9	17.3
	5th	5,910 (4,760 – 7,250)	5,340 (4,400 – 6,500)	3,960 (2,890 – 5,380)	1,940 (419 – 3,330)	32.8%	13.3	13.2	12.8
Cambodia	1st	11,200 (9,830 – 12,700)	9,380 (8,340 – 10,500)	3,130 (1,920 – 5,090)	8,070 (5,740 – 9,920)	72.1%	25.4	25.8	28.0
	2nd	10,200 (9,120 – 11,400)	8,420 (7,500 – 9,360)	2,670 (1,640 – 4,300)	7,580 (5,610 – 9,090)	74.0%	23.2	23.2	23.9
	3rd	9,660 (8,800 – 10,600)	7,980 (7,220 – 8,780)	2,330 (1,440 – 3,760)	7,330 (5,720 – 8,560)	75.9%	21.9	21.9	20.8
	4th	8,400 (7,380 – 9,500)	6,830 (6,030 – 7,710)	1,930 (1,180 – 3,120)	6,470 (4,900 – 7,760)	77.0%	19.0	18.8	17.2
	5th	4,640 (3,840 – 5,540)	3,760 (3,170 – 4,430)	1,140 (684 – 1,910)	3,500 (2,490 – 4,420)	75.5%	10.5	10.3	10.1
Cameroon	1st	18,700 (16,600 – 20,900)	24,900 (22,300 – 27,900)	17,700 (13,300 – 23,500)	949 (-4,930 – 5,530)	5.1%	25.9	24.8	26.7
	2nd	16,300 (14,600 – 18,100)	22,700 (20,300 – 25,100)	15,000 (11,400 – 19,600)	1,260 (-3,410 – 5,100)	7.7%	22.6	22.6	22.7
	3rd	14,800 (13,400 – 16,200)	20,600 (18,700 – 22,700)	13,500 (10,400 – 17,400)	1,260 (-2,740 – 4,480)	8.5%	20.5	20.5	20.4
	4th	12,900 (11,500 – 14,500)	18,900 (16,900 – 21,100)	11,400 (8,560 – 15,000)	1,500 (-2,280 – 4,540)	11.7%	17.9	18.8	17.2
	5th	9,370 (8,140 – 10,700)	13,300 (11,700 – 15,100)	8,650 (6,360 – 11,700)	722 (-2,450 – 3,140)	7.7%	13.0	13.2	13.1
Central African Republic	1st	4,680 (4,090 – 5,360)	5,900 (5,130 – 6,750)	5,060 (3,240 – 7,970)	-377 (-3,380 – 1,590)	-8.0%	22.9	23.7	25.0
	2nd	4,750 (4,190 – 5,380)	5,730 (5,000 – 6,520)	4,570 (2,940 – 7,140)	181 (-2,460 – 1,930)	3.8%	23.2	23.0	22.5
	3rd	4,380 (3,890 – 4,910)	5,290 (4,680 – 5,960)	4,280 (2,760 – 6,680)	103 (-2,320 – 1,710)	2.4%	21.4	21.3	21.1
	4th	3,830 (3,350 – 4,380)	4,590 (4,000 – 5,280)	3,640 (2,300 – 5,700)	193 (-1,910 – 1,630)	5.0%	18.8	18.5	18.0
	5th	2,780 (2,380 – 3,250)	3,360 (2,860 – 3,930)	2,720 (1,680 – 4,340)	61 (-1,600 – 1,150)	2.2%	13.6	13.5	13.4

Continued on next page

Absolute decline: wealth quintile-specific under-5 deaths (1990) - wealth quintile-specific under-5 deaths (2016).
Percentage decline: absolute decline over wealth quintile-specific under-5 deaths (1990) $\times$100.
Share of total death (in percentage): wealth quintile-specific under-5 deaths over the total under-5 deaths $\times$ 100.

Table 9 – continued from previous page

	Wealth Quintile	number of wealth quintile-specific under-5 deaths 1990	2000	2016	Absolute decline 1990–2016	Percentage decline 1990–2016	Share of total death (in percentage) 1990	2000	2016
Chad	1st	10,100 (8,960 – 11,400)	12,700 (11,400 – 14,200)	15,200 (12,300 – 18,600)	-5,090 (-8,590 – -2,000)	-50.3%	16.9	17.2	19.8
	2nd	13,700 (12,400 – 15,200)	16,500 (14,900 – 18,200)	17,000 (13,800 – 20,500)	-3,300 (-6,900 – 146)	-24.0%	22.9	22.3	22.2
	3rd	12,800 (11,700 – 14,000)	15,800 (14,500 – 17,200)	16,100 (13,300 – 19,200)	-3,310 (-6,380 – -352)	-25.9%	21.4	21.3	21.0
	4th	12,300 (11,000 – 13,700)	15,400 (13,900 – 17,000)	14,400 (11,600 – 17,500)	-2,140 (-5,350 – 819)	-17.4%	20.5	20.7	18.8
	5th	11,000 (9,720 – 12,300)	13,700 (12,300 – 15,300)	14,000 (11,200 – 17,000)	-2,980 (-6,210 – 113)	-27.1%	18.3	18.5	18.2
Colombia	1st	8,530 (7,470 – 9,640)	5,840 (5,140 – 6,600)	3,200 (2,410 – 4,210)	5,330 (3,940 – 6,610)	62.5%	26.9	27.5	28.1
	2nd	7,240 (6,410 – 8,110)	4,820 (4,280 – 5,410)	2,610 (1,960 – 3,440)	4,620 (3,510 – 5,660)	63.9%	22.8	22.7	22.9
	3rd	6,320 (5,710 – 6,980)	4,200 (3,780 – 4,670)	2,250 (1,700 – 2,920)	4,070 (3,260 – 4,860)	64.4%	20.0	19.8	19.7
	4th	5,210 (4,540 – 5,960)	3,380 (2,920 – 3,890)	1,750 (1,280 – 2,340)	3,460 (2,620 – 4,300)	66.4%	16.5	16.0	15.4
	5th	4,380 (3,630 – 5,230)	2,970 (2,480 – 3,520)	1,570 (1,130 – 2,140)	2,800 (2,020 – 3,630)	64.0%	13.8	14.0	13.8
Comoros	1st	506 (420 – 601)	493 (382 – 606)	462 (243 – 908)	44 (-415 – 276)	8.7%	23.7	24.3	24.6
	2nd	481 (410 – 554)	455 (360 – 548)	425 (224 – 838)	56 (-371 – 266)	11.6%	22.5	22.4	22.6
	3rd	453 (395 – 513)	428 (341 – 509)	388 (206 – 758)	65 (-312 – 253)	14.3%	21.2	21.1	20.6
	4th	387 (327 – 449)	355 (276 – 432)	326 (170 – 647)	61 (-265 – 223)	15.8%	18.1	17.5	17.3
	5th	312 (250 – 387)	301 (226 – 387)	279 (144 – 562)	33 (-253 – 175)	10.6%	14.6	14.8	14.8
Congo	1st	1,850 (1,540 – 2,210)	3,060 (2,640 – 3,550)	2,210 (1,530 – 3,070)	-357 (-1,260 – 395)	-19.3%	22.6	21.7	23.4
	2nd	1,890 (1,610 – 2,220)	3,240 (2,820 – 3,710)	2,240 (1,570 – 3,070)	-349 (-1,210 – 383)	-18.5%	23.0	22.9	23.7
	3rd	1,750 (1,510 – 2,010)	3,050 (2,700 – 3,440)	1,960 (1,390 – 2,680)	-214 (-963 – 412)	-12.2%	21.3	21.5	20.7
	4th	1,480 (1,250 – 1,760)	2,630 (2,280 – 3,030)	1,660 (1,150 – 2,320)	-173 (-856 – 376)	-11.7%	18.1	18.6	17.5
	5th	1,230 (977 – 1,550)	2,160 (1,770 – 2,610)	1,400 (943 – 2,000)	-167 (-754 – 328)	-13.6%	15.0	15.3	14.8
Cote d'Ivoire	1st	18,500 (16,500 – 20,700)	22,700 (19,900 – 25,700)	19,300 (14,000 – 25,900)	-757 (-7,430 – 4,780)	-4.1%	24.1	23.8	24.8
	2nd	17,500 (15,800 – 19,400)	21,900 (19,500 – 24,500)	17,700 (12,900 – 23,700)	-150 (-6,150 – 4,750)	-0.9%	22.8	23.0	22.7
	3rd	15,800 (14,500 – 17,300)	19,700 (17,800 – 21,800)	16,100 (11,900 – 21,300)	-276 (-5,480 – 3,930)	-1.7%	20.6	20.7	20.7
	4th	14,600 (13,100 – 16,300)	18,000 (16,000 – 20,300)	13,900 (10,200 – 18,700)	750 (-4,150 – 4,730)	5.1%	19.0	18.9	17.8
	5th	10,300 (8,970 – 11,700)	12,900 (11,200 – 14,900)	10,800 (7,720 – 14,900)	-526 (-4,530 – 2,570)	-5.1%	13.4	13.6	13.9
Democratic Republic of the Congo	1st	65,600 (56,400 – 75,900)	79,000 (69,200 – 90,100)	75,900 (53,300 – 106,000)	-10,300 (-41,300 – 13,600)	-15.7%	23.5	23.8	25.0
	2nd	64,000 (56,100 – 72,600)	75,800 (67,300 – 85,800)	70,100 (49,500 – 97,500)	-6,130 (-34,400 – 15,500)	-9.6%	22.9	22.9	23.0
	3rd	59,800 (53,600 – 66,800)	70,900 (63,500 – 79,300)	64,900 (46,500 – 89,700)	-5,010 (-30,000 – 14,200)	-8.4%	21.4	21.4	21.3
	4th	56,500 (49,000 – 64,700)	65,800 (57,900 – 74,900)	55,400 (39,300 – 77,400)	1,090 (-21,500 – 18,500)	1.9%	20.2	19.9	18.2
	5th	33,800 (28,200 – 40,600)	40,000 (34,200 – 46,700)	37,900 (26,200 – 54,200)	-4,020 (-20,300 – 8,130)	-11.9%	12.1	12.1	12.4
Dominican Republic	1st	3,290 (2,880 – 3,740)	2,340 (2,060 – 2,640)	1,720 (1,280 – 2,350)	1,570 (873 – 2,180)	47.7%	26.0	26.7	26.1
	2nd	2,970 (2,650 – 3,320)	2,030 (1,800 – 2,270)	1,540 (1,150 – 2,070)	1,430 (831 – 1,930)	48.2%	23.4	23.1	23.3
	3rd	2,610 (2,380 – 2,860)	1,780 (1,600 – 1,960)	1,340 (1,010 – 1,780)	1,270 (801 – 1,670)	48.7%	20.5	20.3	20.3
	4th	2,210 (1,940 – 2,520)	1,500 (1,310 – 1,710)	1,140 (842 – 1,550)	1,070 (596 – 1,480)	48.4%	17.4	17.1	17.2
	5th	1,610 (1,310 – 1,960)	1,120 (919 – 1,350)	864 (615 – 1,220)	744 (354 – 1,100)	46.3%	12.7	12.8	13.1
Egypt	1st	47,200 (42,900 – 51,700)	24,000 (21,800 – 26,400)	16,700 (12,600 – 21,700)	30,500 (24,200 – 36,400)	64.7%	28.7	29.3	29.0
	2nd	37,700 (34,300 – 41,100)	18,500 (16,800 – 20,300)	13,100 (10,000 – 17,100)	24,600 (19,600 – 29,100)	65.3%	22.9	22.6	22.8
	3rd	33,900 (31,500 – 36,400)	16,400 (15,100 – 17,800)	11,500 (8,920 – 14,800)	22,400 (18,500 – 25,800)	66.1%	20.6	20.0	20.0
	4th	28,200 (25,300 – 31,400)	14,300 (12,800 – 15,900)	9,680 (7,330 – 12,900)	18,500 (14,300 – 22,400)	65.6%	17.1	17.5	16.9
	5th	17,600 (15,000 – 20,400)	8,740 (7,590 – 10,000)	6,520 (4,840 – 8,750)	11,000 (7,870 – 14,100)	62.6%	10.7	10.7	11.4
El Salvador	1st	2,500 (2,050 – 3,000)	1,290 (1,050 – 1,560)	494 (326 – 742)	2,000 (1,530 – 2,490)	80.1%	25.7	26.7	27.9
	2nd	2,260 (1,960 – 2,610)	1,120 (955 – 1,310)	405 (273 – 601)	1,850 (1,500 – 2,210)	81.9%	23.2	23.2	22.9
	3rd	1,980 (1,760 – 2,230)	966 (841 – 1,100)	352 (239 – 517)	1,630 (1,360 – 1,890)	82.1%	20.4	20.0	19.9
	4th	1,670 (1,410 – 1,960)	808 (668 – 957)	279 (183 – 419)	1,390 (1,110 – 1,680)	83.3%	17.2	16.7	15.8
	5th	1,310 (970 – 1,740)	647 (473 – 864)	237 (146 – 377)	1,080 (753 – 1,460)	82.2%	13.5	13.4	13.4

Continued on next page

Absolute decline: wealth quintile-specific under-5 deaths (1990) - wealth quintile-specific under-5 deaths (2016).
Percentage decline: absolute decline over wealth quintile-specific under-5 deaths (1990) $\times$100.
Share of total death (in percentage): wealth quintile-specific under-5 deaths over the total under-5 deaths $\times$ 100.

Table 9 – continued from previous page

	Wealth Quintile	number of wealth quintile-specific under-5 deaths 1990	2000	2016	Absolute decline 1990–2016	Percentage decline 1990–2016	Share of total death (in percentage) 1990	2000	2016
Equatorial Guinea	1st	704 (572 – 862)	809 (681 – 962)	875 (579 – 1,310)	-171 (-613 – 156)	-24.3%	21.7	22.4	24.0
	2nd	680 (563 – 811)	755 (651 – 882)	784 (518 – 1,160)	-104 (-485 – 191)	-15.3%	20.9	20.9	21.5
	3rd	650 (551 – 764)	722 (634 – 832)	729 (485 – 1,060)	-79 (-431 – 192)	-12.1%	20.0	20.0	20.0
	4th	624 (514 – 750)	674 (572 – 798)	624 (411 – 931)	0 (-320 – 244)	0.0%	19.2	18.6	17.1
	5th	589 (466 – 738)	656 (537 – 806)	637 (403 – 976)	-48 (-380 – 201)	-8.2%	18.1	18.1	17.4
Eritrea	1st	3,760 (3,150 – 4,460)	2,190 (1,860 – 2,570)	1,660 (1,030 – 2,670)	2,110 (998 – 2,970)	56.1%	20.0	21.1	23.4
	2nd	4,490 (3,910 – 5,110)	2,510 (2,190 – 2,860)	1,740 (1,100 – 2,780)	2,740 (1,610 – 3,600)	61.1%	23.8	24.1	24.7
	3rd	4,220 (3,760 – 4,710)	2,340 (2,070 – 2,620)	1,510 (961 – 2,400)	2,700 (1,740 – 3,410)	64.0%	22.4	22.5	21.4
	4th	3,920 (3,380 – 4,530)	2,020 (1,740 – 2,330)	1,280 (800 – 2,050)	2,640 (1,740 – 3,380)	67.3%	20.8	19.5	18.1
	5th	2,440 (1,910 – 3,080)	1,340 (1,050 – 1,670)	879 (526 – 1,480)	1,560 (907 – 2,160)	63.9%	13.0	12.8	12.4
Ethiopia	1st	82,800 (71,900 – 94,900)	78,900 (70,500 – 88,100)	43,000 (33,000 – 55,200)	39,700 (23,900 – 54,400)	48.0%	18.7	19.5	23.0
	2nd	97,300 (86,600 – 109,000)	87,600 (78,800 – 96,800)	43,400 (33,800 – 55,400)	54,000 (38,600 – 68,400)	55.5%	22.1	21.7	23.2
	3rd	95,400 (86,800 – 104,000)	87,400 (79,800 – 95,500)	38,800 (30,500 – 48,900)	56,600 (44,300 – 67,900)	59.3%	21.6	21.6	20.8
	4th	95,900 (84,500 – 108,000)	86,200 (77,500 – 95,600)	33,800 (25,900 – 43,200)	62,100 (48,000 – 75,300)	64.8%	21.7	21.3	18.1
	5th	70,100 (60,200 – 81,300)	64,100 (56,700 – 71,900)	27,900 (21,200 – 36,200)	42,200 (30,200 – 54,300)	60.2%	15.9	15.9	14.9
Gabon	1st	712 (586 – 864)	764 (635 – 934)	656 (439 – 960)	57 (-263 – 318)	8.0%	22.6	22.5	24.3
	2nd	745 (626 – 885)	808 (681 – 970)	647 (438 – 944)	98 (-209 – 356)	13.2%	23.6	23.8	23.9
	3rd	677 (576 – 795)	727 (620 – 864)	560 (380 – 815)	117 (-144 – 337)	17.3%	21.5	21.4	20.7
	4th	581 (484 – 704)	625 (523 – 759)	470 (316 – 690)	110 (-119 – 309)	18.9%	18.4	18.4	17.4
	5th	441 (338 – 567)	474 (369 – 607)	370 (234 – 563)	70 (-118 – 229)	15.9%	14.0	14.0	13.7
Gambia	1st	1,480 (1,230 – 1,760)	1,460 (1,220 – 1,710)	1,230 (755 – 1,940)	244 (-485 – 776)	16.5%	21.9	23.3	24.2
	2nd	1,540 (1,320 – 1,770)	1,420 (1,210 – 1,640)	1,200 (739 – 1,890)	333 (-358 – 835)	21.7%	22.8	22.7	23.6
	3rd	1,450 (1,270 – 1,640)	1,350 (1,170 – 1,550)	1,080 (674 – 1,680)	374 (-241 – 816)	25.8%	21.5	21.6	21.1
	4th	1,360 (1,160 – 1,590)	1,180 (996 – 1,380)	928 (571 – 1,470)	429 (-133 – 836)	31.6%	20.1	18.9	18.2
	5th	914 (742 – 1,120)	840 (683 – 1,020)	660 (401 – 1,070)	254 (-149 – 555)	27.8%	13.6	13.5	12.9
Georgia	1st	1,110 (904 – 1,350)	530 (430 – 644)	158 (126 – 197)	955 (756 – 1,180)	85.8%	25.6	25.7	27.1
	2nd	1,040 (890 – 1,210)	498 (422 – 585)	137 (114 – 166)	907 (752 – 1,070)	86.8%	24.0	24.2	23.5
	3rd	894 (775 – 1,020)	420 (362 – 486)	118 (100 – 140)	776 (658 – 898)	86.8%	20.5	20.4	20.2
	4th	744 (616 – 886)	348 (290 – 418)	94 (75 – 117)	650 (523 – 788)	87.4%	17.1	16.9	16.1
	5th	561 (412 – 751)	264 (194 – 358)	76 (54 – 104)	485 (348 – 659)	86.4%	12.9	12.8	13.0
Ghana	1st	17,100 (15,500 – 18,900)	16,400 (14,800 – 18,100)	13,100 (9,750 – 17,500)	3,990 (-575 – 7,750)	23.3%	24.6	25.1	25.9
	2nd	15,500 (14,100 – 17,000)	14,000 (12,700 – 15,400)	11,200 (8,360 – 14,600)	4,320 (743 – 7,500)	27.8%	22.3	21.4	22.1
	3rd	14,500 (13,400 – 15,700)	13,500 (12,500 – 14,700)	10,100 (7,670 – 13,300)	4,330 (1,160 – 6,950)	29.9%	20.8	20.7	20.0
	4th	12,800 (11,500 – 14,300)	11,800 (10,600 – 13,100)	8,740 (6,490 – 11,700)	4,100 (1,110 – 6,670)	31.9%	18.4	18.0	17.3
	5th	9,790 (8,510 – 11,200)	9,620 (8,440 – 11,000)	7,430 (5,410 – 10,100)	2,360 (-350 – 4,610)	24.1%	14.0	14.7	14.7
Guatemala	1st	6,560 (5,760 – 7,410)	5,070 (4,380 – 5,800)	3,000 (2,350 – 3,800)	3,560 (2,540 – 4,520)	54.3%	22.8	24.0	25.3
	2nd	6,740 (6,050 – 7,480)	5,100 (4,510 – 5,730)	2,870 (2,300 – 3,590)	3,880 (2,920 – 4,720)	57.5%	23.4	24.1	24.2
	3rd	6,180 (5,650 – 6,730)	4,410 (3,960 – 4,880)	2,420 (1,980 – 3,010)	3,760 (3,050 – 4,400)	60.8%	21.5	20.9	20.5
	4th	5,290 (4,650 – 5,960)	3,710 (3,200 – 4,240)	2,000 (1,590 – 2,530)	3,290 (2,510 – 4,000)	62.2%	18.4	17.5	16.9
	5th	4,030 (3,270 – 4,890)	2,830 (2,270 – 3,470)	1,550 (1,160 – 2,070)	2,480 (1,780 – 3,220)	61.6%	14.0	13.4	13.1
Guinea	1st	14,600 (12,900 – 16,500)	14,300 (12,900 – 15,800)	10,000 (7,840 – 12,700)	4,620 (1,430 – 7,380)	31.5%	23.2	23.7	25.9
	2nd	14,200 (12,800 – 15,800)	13,500 (12,200 – 14,800)	8,720 (6,900 – 11,000)	5,480 (2,830 – 7,780)	38.6%	22.5	22.4	22.5
	3rd	13,200 (12,100 – 14,400)	12,600 (11,500 – 13,700)	8,040 (6,430 – 10,100)	5,160 (2,980 – 7,050)	39.1%	20.9	20.8	20.7
	4th	12,200 (10,800 – 13,700)	11,700 (10,600 – 13,000)	6,970 (5,480 – 8,880)	5,260 (3,020 – 7,300)	43.0%	19.4	19.5	18.0
	5th	8,730 (7,540 – 10,100)	8,150 (7,190 – 9,230)	5,050 (3,900 – 6,520)	3,680 (1,940 – 5,320)	42.2%	13.9	13.5	13.0

Continued on next page

Absolute decline: wealth quintile-specific under-5 deaths (1990) - wealth quintile-specific under-5 deaths (2016).
Percentage decline: absolute decline over wealth quintile-specific under-5 deaths (1990) $\times$100.
Share of total death (in percentage): wealth quintile-specific under-5 deaths over the total under-5 deaths $\times$ 100.

Table 9 – continued from previous page

	Wealth Quintile	number of wealth quintile-specific under-5 deaths 1990	2000	2016	Absolute decline 1990–2016	Percentage decline 1990–2016	Share of total death (in percentage) 1990	2000	2016
Guinea-Bissau	1st	2,190 (1,860 – 2,550)	1,930 (1,670 – 2,210)	1,360 (930 – 1,920)	829 (218 – 1,370)	37.9%	22.0	22.1	23.8
	2nd	2,310 (2,010 – 2,650)	2,050 (1,800 – 2,330)	1,320 (903 – 1,870)	990 (392 – 1,510)	42.9%	23.2	23.5	23.1
	3rd	2,110 (1,850 – 2,370)	1,840 (1,640 – 2,070)	1,200 (829 – 1,680)	907 (393 – 1,340)	43.1%	21.2	21.1	21.0
	4th	1,940 (1,660 – 2,240)	1,650 (1,440 – 1,890)	1,020 (697 – 1,430)	921 (436 – 1,340)	47.5%	19.5	18.9	17.8
	5th	1,410 (1,180 – 1,680)	1,250 (1,070 – 1,460)	818 (552 – 1,180)	591 (204 – 940)	42.0%	14.2	14.3	14.3
Guyana	1st	292 (241 – 350)	209 (171 – 248)	120 (79 – 184)	172 (97 – 236)	58.9%	22.8	22.9	23.4
	2nd	303 (261 – 349)	218 (187 – 251)	122 (82 – 184)	181 (109 – 242)	59.8%	23.6	23.8	23.7
	3rd	266 (235 – 301)	188 (166 – 214)	105 (71 – 157)	162 (103 – 209)	60.8%	20.8	20.6	20.4
	4th	225 (191 – 265)	159 (134 – 186)	88 (59 – 135)	137 (81 – 184)	60.8%	17.6	17.4	17.2
	5th	196 (150 – 253)	140 (108 – 180)	78 (49 – 124)	117 (65 – 169)	59.8%	15.3	15.3	15.2
Haiti	1st	8,240 (7,320 – 9,300)	6,340 (5,620 – 7,140)	4,150 (3,130 – 5,550)	4,100 (2,560 – 5,410)	49.8%	21.8	23.0	23.7
	2nd	8,570 (7,710 – 9,500)	6,280 (5,590 – 7,000)	4,060 (3,100 – 5,400)	4,510 (3,040 – 5,780)	52.6%	22.7	22.8	23.2
	3rd	8,050 (7,380 – 8,780)	5,850 (5,290 – 6,420)	3,640 (2,800 – 4,760)	4,420 (3,170 – 5,430)	54.9%	21.3	21.3	20.8
	4th	7,330 (6,480 – 8,240)	5,050 (4,460 – 5,670)	3,160 (2,410 – 4,230)	4,160 (2,900 – 5,280)	56.8%	19.4	18.3	18.1
	5th	5,580 (4,790 – 6,490)	3,990 (3,410 – 4,630)	2,480 (1,820 – 3,360)	3,100 (2,070 – 4,090)	55.5%	14.8	14.5	14.2
Honduras	1st	2,990 (2,540 – 3,460)	2,260 (1,960 – 2,580)	1,080 (777 – 1,480)	1,910 (1,360 – 2,430)	63.9%	27.5	28.7	29.2
	2nd	2,510 (2,200 – 2,840)	1,810 (1,590 – 2,040)	835 (608 – 1,130)	1,670 (1,260 – 2,050)	66.6%	23.1	23.0	22.6
	3rd	2,210 (1,970 – 2,440)	1,570 (1,400 – 1,740)	734 (537 – 992)	1,470 (1,160 – 1,740)	66.6%	20.3	20.0	19.9
	4th	1,840 (1,570 – 2,120)	1,290 (1,110 – 1,480)	593 (423 – 822)	1,250 (922 – 1,550)	68.0%	16.9	16.4	16.0
	5th	1,330 (1,050 – 1,650)	942 (752 – 1,160)	452 (307 – 645)	874 (597 – 1,170)	65.9%	12.2	12.0	12.2
India	1st	915,000 (863,000 – 969,000)	693,000 (645,000 – 742,000)	308,000 (262,000 – 356,000)	608,000 (538,000 – 678,000)	66.4%	27.0	27.5	28.5
	2nd	865,000 (813,000 – 917,000)	633,000 (589,000 – 678,000)	266,000 (229,000 – 303,000)	599,000 (537,000 – 662,000)	69.3%	25.5	25.1	24.6
	3rd	716,000 (679,000 – 756,000)	532,000 (500,000 – 565,000)	221,000 (195,000 – 247,000)	495,000 (453,000 – 541,000)	69.1%	21.1	21.1	20.5
	4th	567,000 (530,000 – 605,000)	408,000 (376,000 – 443,000)	176,000 (149,000 – 204,000)	391,000 (344,000 – 437,000)	69.0%	16.7	16.2	16.3
	5th	332,000 (309,000 – 358,000)	255,000 (233,000 – 280,000)	111,000 (91,300 – 135,000)	222,000 (189,000 – 252,000)	66.8%	9.8	10.1	10.3
Indonesia	1st	112,000 (99,700 – 125,000)	69,900 (63,000 – 77,400)	39,000 (30,200 – 50,300)	72,500 (57,200 – 87,600)	65.0%	28.3	29.5	29.8
	2nd	88,400 (79,700 – 97,200)	54,100 (48,600 – 59,600)	29,600 (22,900 – 38,000)	58,800 (47,000 – 68,800)	66.5%	22.4	22.8	22.6
	3rd	81,700 (75,300 – 88,200)	47,800 (43,800 – 51,800)	26,000 (20,400 – 33,200)	55,600 (46,800 – 63,400)	68.1%	20.7	20.2	19.9
	4th	68,700 (60,700 – 76,900)	39,500 (34,900 – 44,400)	21,600 (16,500 – 28,200)	47,100 (37,600 – 56,500)	68.6%	17.4	16.7	16.5
	5th	44,200 (36,800 – 52,900)	25,700 (21,700 – 30,400)	14,700 (10,800 – 19,700)	29,500 (21,900 – 37,700)	66.7%	11.2	10.8	11.2
Iraq	1st	8,140 (6,800 – 9,600)	8,540 (7,290 – 9,980)	8,880 (6,440 – 12,400)	-736 (-4,260 – 1,880)	-9.0%	23.3	23.2	23.6
	2nd	7,920 (6,870 – 9,070)	8,280 (7,180 – 9,480)	8,410 (6,180 – 11,500)	-485 (-3,600 – 1,890)	-6.1%	22.6	22.5	22.3
	3rd	6,970 (6,180 – 7,800)	7,270 (6,440 – 8,150)	7,400 (5,460 – 10,100)	-422 (-3,110 – 1,540)	-6.1%	19.9	19.8	19.6
	4th	5,990 (5,090 – 6,960)	6,300 (5,360 – 7,310)	6,410 (4,680 – 8,930)	-423 (-2,960 – 1,430)	-7.1%	17.1	17.1	17.0
	5th	5,980 (4,780 – 7,410)	6,400 (5,210 – 7,800)	6,580 (4,650 – 9,350)	-594 (-3,200 – 1,400)	-9.9%	17.1	17.4	17.5
Jordan	1st	1,110 (969 – 1,270)	1,090 (942 – 1,260)	1,110 (768 – 1,570)	-2 (-471 – 359)	-0.2%	24.8	24.9	26.1
	2nd	1,020 (897 – 1,140)	992 (865 – 1,120)	968 (680 – 1,360)	49 (-356 – 354)	4.8%	22.6	22.6	22.7
	3rd	904 (818 – 995)	880 (786 – 978)	850 (601 – 1,180)	54 (-287 – 310)	6.0%	20.1	20.1	20.0
	4th	795 (696 – 905)	768 (662 – 888)	704 (475 – 1,010)	91 (-214 – 338)	11.5%	17.7	17.5	16.5
	5th	662 (552 – 792)	654 (541 – 786)	627 (419 – 922)	35 (-240 – 250)	5.3%	14.8	14.9	14.7
Kazakhstan	1st	4,960 (4,160 – 5,820)	2,550 (2,140 – 2,970)	1,140 (939 – 1,360)	3,820 (3,070 – 4,630)	77.1%	24.4	24.6	26.2
	2nd	4,820 (4,160 – 5,520)	2,460 (2,150 – 2,810)	1,010 (867 – 1,160)	3,810 (3,170 – 4,500)	79.0%	23.7	23.8	23.3
	3rd	4,180 (3,680 – 4,710)	2,120 (1,880 – 2,370)	880 (784 – 977)	3,300 (2,830 – 3,810)	79.0%	20.6	20.5	20.2
	4th	3,510 (2,990 – 4,070)	1,760 (1,500 – 2,030)	703 (570 – 833)	2,810 (2,290 – 3,360)	80.0%	17.3	17.0	16.1
	5th	2,870 (2,230 – 3,630)	1,460 (1,150 – 1,830)	617 (474 – 786)	2,250 (1,680 – 2,930)	78.5%	14.1	14.1	14.2

Continued on next page

Absolute decline: wealth quintile-specific under-5 deaths (1990) - wealth quintile-specific under-5 deaths (2016).
Percentage decline: absolute decline over wealth quintile-specific under-5 deaths (1990) $\times$100.
Share of total death (in percentage): wealth quintile-specific under-5 deaths over the total under-5 deaths $\times$ 100.

Table 9 – continued from previous page

	Wealth Quintile	number of wealth quintile-specific under-5 deaths 1990	2000	2016	Absolute decline 1990–2016	Percentage decline 1990–2016	Share of total death (in percentage) 1990	2000	2016
Kenya	1st	24,300 (21,900 – 27,000)	30,200 (27,100 – 33,500)	18,100 (14,400 – 22,700)	6,260 (1,450 – 10,600)	25.7%	25.6	24.9	24.6
	2nd	21,700 (19,700 – 23,900)	26,900 (24,300 – 29,800)	17,000 (13,700 – 21,100)	4,770 (443 – 8,650)	22.0%	22.8	22.2	23.1
	3rd	19,300 (17,700 – 20,900)	24,600 (22,600 – 26,900)	14,600 (11,900 – 18,000)	4,680 (1,050 – 7,540)	24.3%	20.3	20.4	19.9
	4th	15,800 (14,000 – 17,600)	20,700 (18,600 – 23,100)	12,800 (10,200 – 16,000)	2,970 (-615 – 6,020)	18.8%	16.6	17.1	17.4
	5th	14,000 (12,300 – 15,800)	18,600 (16,400 – 21,000)	11,100 (8,700 – 14,100)	2,900 (-391 – 5,620)	20.7%	14.7	15.4	15.1
Kyrgyzstan	1st	2,220 (1,840 – 2,640)	1,360 (1,130 – 1,580)	851 (717 – 995)	1,370 (1,010 – 1,760)	61.6%	25.1	25.6	26.8
	2nd	2,040 (1,750 – 2,370)	1,240 (1,060 – 1,400)	730 (638 – 828)	1,310 (1,020 – 1,630)	64.3%	23.0	23.3	22.9
	3rd	1,820 (1,580 – 2,070)	1,070 (932 – 1,190)	635 (572 – 698)	1,180 (952 – 1,430)	65.0%	20.4	20.2	20.0
	4th	1,530 (1,280 – 1,820)	887 (741 – 1,030)	516 (436 – 602)	1,010 (762 – 1,290)	66.1%	17.2	16.7	16.2
	5th	1,270 (988 – 1,610)	749 (589 – 936)	448 (352 – 564)	822 (579 – 1,120)	64.7%	14.3	14.1	14.1
Lao People's Democratic Republic	1st	6,860 (5,700 – 8,200)	5,170 (4,280 – 6,170)	2,810 (1,950 – 3,980)	4,050 (2,580 – 5,520)	59.0%	24.3	26.0	27.3
	2nd	6,620 (5,700 – 7,620)	4,680 (4,020 – 5,440)	2,480 (1,760 – 3,470)	4,140 (2,900 – 5,280)	62.6%	23.4	23.5	24.1
	3rd	6,160 (5,460 – 6,930)	4,340 (3,810 – 4,940)	2,180 (1,550 – 3,040)	3,980 (2,950 – 4,920)	64.6%	21.8	21.8	21.2
	4th	5,660 (4,830 – 6,560)	3,710 (3,150 – 4,370)	1,820 (1,270 – 2,580)	3,840 (2,800 – 4,860)	67.8%	20.0	18.6	17.7
	5th	2,960 (2,250 – 3,830)	2,020 (1,560 – 2,610)	1,010 (662 – 1,530)	1,940 (1,250 – 2,710)	65.6%	10.5	10.2	9.8
Lesotho	1st	1,160 (982 – 1,340)	1,410 (1,220 – 1,620)	1,250 (937 – 1,650)	-95 (-509 – 240)	-8.2%	22.5	21.7	22.1
	2nd	1,160 (1,010 – 1,320)	1,450 (1,280 – 1,630)	1,280 (969 – 1,670)	-115 (-529 – 220)	-9.9%	22.6	22.4	22.6
	3rd	1,090 (965 – 1,220)	1,380 (1,250 – 1,530)	1,200 (916 – 1,550)	-107 (-471 – 182)	-9.8%	21.2	21.3	21.1
	4th	945 (811 – 1,090)	1,240 (1,090 – 1,410)	1,070 (808 – 1,420)	-129 (-486 – 156)	-13.7%	18.4	19.1	19.0
	5th	793 (655 – 957)	1,020 (861 – 1,190)	860 (628 – 1,160)	-67 (-371 – 179)	-8.5%	15.4	15.6	15.2
Liberia	1st	4,970 (4,220 – 5,860)	4,120 (3,530 – 4,750)	2,420 (1,780 – 3,400)	2,550 (1,450 – 3,550)	51.3%	20.2	20.3	23.1
	2nd	5,280 (4,610 – 6,020)	4,250 (3,770 – 4,790)	2,300 (1,700 – 3,180)	2,990 (1,960 – 3,850)	56.6%	21.4	21.0	21.9
	3rd	5,060 (4,530 – 5,630)	4,160 (3,760 – 4,590)	2,100 (1,570 – 2,910)	2,960 (2,090 – 3,670)	58.5%	20.5	20.5	20.1
	4th	4,910 (4,210 – 5,650)	4,030 (3,530 – 4,580)	1,820 (1,330 – 2,540)	3,090 (2,170 – 3,930)	63.0%	19.9	19.9	17.4
	5th	4,430 (3,670 – 5,300)	3,710 (3,180 – 4,330)	1,830 (1,330 – 2,600)	2,600 (1,630 – 3,530)	58.7%	18.0	18.3	17.5
Madagascar	1st	18,300 (16,100 – 20,600)	17,000 (15,100 – 19,200)	10,000 (6,790 – 14,400)	8,280 (3,460 – 12,100)	45.3%	23.4	25.1	26.8
	2nd	18,200 (16,300 – 20,200)	15,800 (14,000 – 17,700)	8,920 (6,130 – 12,800)	9,250 (5,050 – 12,600)	50.9%	23.3	23.3	23.9
	3rd	16,600 (15,200 – 18,100)	14,400 (13,000 – 15,800)	7,660 (5,300 – 10,900)	8,980 (5,580 – 11,600)	54.0%	21.3	21.3	20.5
	4th	15,500 (13,800 – 17,300)	12,400 (10,900 – 13,900)	6,410 (4,370 – 9,160)	9,080 (5,870 – 11,700)	58.6%	19.8	18.3	17.1
	5th	9,440 (7,990 – 11,100)	8,090 (6,830 – 9,500)	4,380 (2,890 – 6,520)	5,070 (2,840 – 7,100)	53.7%	12.1	12.0	11.7
Malawi	1st	20,500 (18,500 – 22,600)	17,900 (16,400 – 19,500)	8,450 (6,400 – 11,100)	12,100 (8,740 – 15,000)	59.0%	20.9	21.2	23.7
	2nd	21,600 (19,600 – 23,600)	18,800 (17,300 – 20,300)	8,300 (6,320 – 10,900)	13,300 (10,100 – 16,100)	61.6%	21.9	22.2	23.3
	3rd	21,000 (19,500 – 22,500)	17,900 (16,700 – 19,200)	7,290 (5,610 – 9,430)	13,700 (11,200 – 15,900)	65.2%	21.3	21.1	20.4
	4th	20,500 (18,600 – 22,400)	17,200 (15,700 – 18,700)	6,210 (4,690 – 8,150)	14,300 (11,600 – 16,700)	69.9%	20.8	20.3	17.4
	5th	14,800 (13,300 – 16,600)	12,800 (11,600 – 14,000)	5,430 (4,110 – 7,200)	9,410 (7,130 – 11,400)	63.4%	15.1	15.1	15.2
Maldives	1st	207 (170 – 250)	73 (60 – 88)	18 (13 – 24)	189 (152 – 230)	91.3%	24.2	25.7	27.1
	2nd	195 (168 – 225)	67 (57 – 78)	15 (12 – 20)	180 (153 – 210)	92.1%	22.8	23.6	23.2
	3rd	180 (159 – 202)	58 (51 – 66)	13 (10 – 17)	167 (145 – 189)	92.6%	21.1	20.3	20.1
	4th	154 (130 – 181)	49 (41 – 58)	11 (8 – 14)	143 (119 – 170)	93.1%	18.0	17.1	16.1
	5th	119 (88 – 159)	38 (28 – 51)	9 (6 – 13)	110 (80 – 148)	92.6%	13.9	13.4	13.5
Mali	1st	22,100 (20,100 – 24,400)	24,000 (21,700 – 26,400)	19,800 (12,100 – 31,900)	2,360 (-9,660 – 10,100)	10.7%	22.0	21.6	24.0
	2nd	22,700 (20,700 – 24,900)	25,600 (23,300 – 28,200)	19,100 (11,700 – 30,600)	3,670 (-7,690 – 11,100)	16.1%	22.5	23.0	23.1
	3rd	21,700 (20,000 – 23,400)	24,100 (22,100 – 26,300)	17,800 (11,100 – 28,600)	3,820 (-6,850 – 10,600)	17.6%	21.5	21.7	21.7
	4th	20,700 (18,800 – 22,800)	23,300 (21,100 – 25,700)	15,500 (9,640 – 25,200)	5,190 (-4,440 – 11,400)	25.0%	20.6	20.9	18.9
	5th	13,600 (12,100 – 15,200)	14,200 (12,700 – 15,800)	10,200 (6,170 – 16,500)	3,380 (-2,960 – 7,600)	24.9%	13.5	12.8	12.4

Continued on next page

Absolute decline: wealth quintile-specific under-5 deaths (1990) - wealth quintile-specific under-5 deaths (2016).
Percentage decline: absolute decline over wealth quintile-specific under-5 deaths (1990) $\times$100.
Share of total death (in percentage): wealth quintile-specific under-5 deaths over the total under-5 deaths $\times$ 100.

Table 9 – continued from previous page

	Wealth Quintile	number of wealth quintile-specific under-5 deaths 1990	2000	2016	Absolute decline 1990–2016	Percentage decline 1990–2016	Share of total death (in percentage) 1990	2000	2016
Mauritania	1st	2,330 (1,960 – 2,760)	2,880 (2,440 – 3,400)	2,970 (1,630 – 5,420)	-643 (-3,100 – 731)	-27.6%	25.1	25.5	25.6
	2nd	2,060 (1,770 – 2,360)	2,490 (2,140 – 2,900)	2,620 (1,440 – 4,750)	-559 (-2,680 – 644)	-27.2%	22.1	22.0	22.5
	3rd	1,920 (1,690 – 2,170)	2,330 (2,030 – 2,660)	2,360 (1,310 – 4,260)	-442 (-2,340 – 617)	-23.0%	20.7	20.6	20.4
	4th	1,650 (1,400 – 1,910)	1,980 (1,680 – 2,310)	1,980 (1,090 – 3,570)	-334 (-1,950 – 581)	-20.3%	17.7	17.5	17.1
	5th	1,340 (1,070 – 1,640)	1,630 (1,320 – 2,000)	1,670 (909 – 3,110)	-332 (-1,730 – 456)	-24.8%	14.4	14.4	14.4
Mongolia	1st	1,940 (1,660 – 2,250)	803 (691 – 928)	377 (248 – 569)	1,570 (1,240 – 1,880)	80.7%	25.5	26.8	29.1
	2nd	1,760 (1,540 – 1,990)	711 (619 – 809)	302 (199 – 448)	1,450 (1,200 – 1,700)	82.6%	23.0	23.7	23.3
	3rd	1,600 (1,430 – 1,780)	611 (537 – 687)	257 (170 – 378)	1,340 (1,140 – 1,540)	83.8%	20.9	20.4	19.8
	4th	1,320 (1,140 – 1,530)	497 (422 – 577)	201 (130 – 303)	1,120 (926 – 1,320)	84.6%	17.3	16.6	15.5
	5th	1,010 (814 – 1,240)	378 (304 – 462)	161 (102 – 249)	852 (651 – 1,070)	84.2%	13.3	12.6	12.4
Morocco	1st	15,500 (13,500 – 17,700)	8,890 (7,700 – 10,200)	5,410 (3,800 – 7,550)	10,100 (7,390 – 12,600)	65.2%	26.7	27.9	28.4
	2nd	13,900 (12,400 – 15,500)	7,870 (6,920 – 8,930)	4,550 (3,240 – 6,290)	9,360 (7,120 – 11,300)	67.3%	23.9	24.7	23.9
	3rd	12,300 (11,100 – 13,500)	6,530 (5,820 – 7,300)	3,880 (2,800 – 5,310)	8,400 (6,630 – 9,930)	68.4%	21.1	20.5	20.3
	4th	10,300 (8,960 – 11,700)	5,290 (4,540 – 6,140)	3,180 (2,260 – 4,440)	7,100 (5,320 – 8,660)	69.1%	17.7	16.6	16.7
	5th	6,160 (5,050 – 7,440)	3,320 (2,670 – 4,070)	2,050 (1,400 – 3,000)	4,110 (2,900 – 5,350)	66.7%	10.6	10.4	10.8
Mozambique	1st	32,100 (28,000 – 36,700)	29,500 (26,600 – 32,700)	18,900 (13,500 – 25,900)	13,300 (5,440 – 19,700)	41.4%	21.6	21.7	24.2
	2nd	33,200 (29,800 – 37,200)	29,800 (27,000 – 32,900)	17,400 (12,700 – 23,800)	15,800 (8,870 – 21,800)	47.5%	22.4	21.9	22.4
	3rd	31,100 (28,200 – 34,100)	28,500 (26,100 – 31,200)	16,000 (11,700 – 21,700)	15,100 (9,020 – 20,100)	48.5%	20.9	21.0	20.5
	4th	29,900 (26,400 – 33,800)	27,900 (25,200 – 30,800)	13,900 (10,100 – 19,100)	16,000 (9,960 – 21,100)	53.5%	20.1	20.5	17.8
	5th	22,200 (19,000 – 25,600)	20,200 (18,000 – 22,600)	11,800 (8,400 – 16,300)	10,400 (5,150 – 15,000)	46.9%	14.9	14.9	15.1
Myanmar	1st	31,000 (25,500 – 37,100)	24,700 (20,500 – 29,300)	12,400 (9,100 – 16,400)	18,500 (12,600 – 24,800)	59.7%	24.1	24.8	26.1
	2nd	29,300 (25,300 – 33,500)	23,000 (19,900 – 26,300)	11,400 (8,560 – 14,700)	18,000 (13,100 – 22,800)	61.4%	22.9	23.1	23.8
	3rd	27,300 (24,200 – 30,600)	21,000 (18,800 – 23,500)	9,770 (7,470 – 12,400)	17,500 (13,700 – 21,400)	64.2%	21.2	21.1	20.5
	4th	23,400 (19,800 – 27,300)	17,600 (15,000 – 20,400)	8,050 (6,010 – 10,500)	15,400 (11,400 – 19,600)	65.7%	18.3	17.7	16.9
	5th	17,300 (12,700 – 22,900)	13,300 (9,950 – 17,500)	6,130 (4,190 – 8,700)	11,200 (7,270 – 15,800)	64.7%	13.5	13.3	12.8
Namibia	1st	844 (727 – 969)	1,010 (877 – 1,160)	798 (540 – 1,180)	46 (-341 – 319)	5.5%	22.9	23.0	24.9
	2nd	872 (770 – 985)	1,050 (925 – 1,180)	765 (522 – 1,130)	107 (-256 – 370)	12.3%	23.7	23.8	23.9
	3rd	772 (692 – 855)	927 (835 – 1,030)	657 (449 – 961)	114 (-197 – 334)	14.8%	21.0	21.1	20.5
	4th	683 (594 – 787)	827 (722 – 950)	569 (385 – 846)	114 (-168 – 323)	16.7%	18.6	18.8	17.8
	5th	508 (422 – 608)	592 (492 – 704)	417 (275 – 634)	91 (-122 – 250)	17.9%	13.8	13.4	13.0
Nepal	1st	21,600 (19,200 – 24,300)	14,600 (13,000 – 16,300)	5,040 (4,050 – 6,280)	16,600 (14,000 – 19,300)	76.7%	21.8	23.5	25.6
	2nd	22,900 (20,700 – 25,300)	14,400 (13,000 – 15,900)	4,700 (3,830 – 5,800)	18,200 (15,800 – 20,700)	79.4%	23.1	23.3	23.8
	3rd	21,800 (20,000 – 23,700)	13,400 (12,300 – 14,600)	4,070 (3,360 – 4,960)	17,700 (15,800 – 19,700)	81.3%	22.0	21.7	20.6
	4th	19,400 (17,300 – 21,700)	11,200 (10,100 – 12,500)	3,370 (2,690 – 4,230)	16,000 (13,800 – 18,300)	82.6%	19.6	18.1	17.1
	5th	13,400 (11,500 – 15,400)	8,250 (7,170 – 9,470)	2,540 (1,970 – 3,240)	10,800 (8,950 – 12,800)	80.9%	13.5	13.3	12.9
Nicaragua	1st	2,420 (2,080 – 2,790)	1,380 (1,170 – 1,620)	630 (387 – 1,010)	1,790 (1,300 – 2,210)	74.1%	24.1	25.3	26.5
	2nd	2,430 (2,150 – 2,740)	1,340 (1,170 – 1,530)	567 (350 – 914)	1,860 (1,440 – 2,230)	76.5%	24.3	24.5	23.9
	3rd	2,130 (1,920 – 2,360)	1,140 (1,010 – 1,280)	489 (307 – 786)	1,640 (1,300 – 1,920)	77.0%	21.3	20.8	20.6
	4th	1,800 (1,560 – 2,070)	953 (816 – 1,110)	398 (245 – 646)	1,400 (1,070 – 1,710)	77.7%	18.0	17.4	16.7
	5th	1,240 (978 – 1,540)	663 (521 – 835)	294 (174 – 495)	941 (659 – 1,220)	76.2%	12.3	12.1	12.4
Niger	1st	25,500 (22,700 – 28,600)	23,300 (21,000 – 25,800)	18,200 (12,700 – 26,200)	7,330 (-983 – 13,400)	28.7%	18.6	18.2	21.1
	2nd	33,000 (29,800 – 36,400)	30,100 (27,200 – 33,200)	20,100 (14,300 – 28,600)	12,900 (3,730 – 19,600)	39.1%	24.0	23.5	23.4
	3rd	30,100 (27,600 – 32,600)	28,000 (25,600 – 30,500)	18,600 (13,300 – 26,400)	11,500 (3,630 – 17,300)	38.2%	21.9	21.8	21.6
	4th	29,200 (26,100 – 32,400)	27,900 (25,200 – 30,800)	16,500 (11,600 – 23,600)	12,700 (5,180 – 18,400)	43.5%	21.3	21.8	19.2
	5th	19,500 (17,100 – 22,100)	18,800 (16,700 – 21,000)	12,600 (8,800 – 18,200)	6,940 (1,040 – 11,400)	35.5%	14.2	14.7	14.7

Continued on next page

Absolute decline: wealth quintile-specific under-5 deaths (1990) - wealth quintile-specific under-5 deaths (2016).
Percentage decline: absolute decline over wealth quintile-specific under-5 deaths (1990) ×100.
Share of total death (in percentage): wealth quintile-specific under-5 deaths over the total under-5 deaths × 100.

Table 9 – continued from previous page

	Wealth Quintile	number of wealth quintile-specific under-5 deaths 1990	2000	2016	Absolute decline 1990–2016	Percentage decline 1990–2016	Share of total death (in percentage) 1990	2000	2016
Nigeria	1st	205,000 (182,000 – 230,000)	228,000 (208,000 – 249,000)	197,000 (144,000 – 267,000)	8,290 (-64,500 – 64,000)	4.0%	23.8	24.2	26.9
	2nd	206,000 (186,000 – 228,000)	235,000 (215,000 – 255,000)	176,000 (129,000 – 238,000)	30,500 (-33,200 – 80,900)	14.8%	23.9	24.9	24.0
	3rd	187,000 (171,000 – 203,000)	204,000 (188,000 – 220,000)	157,000 (116,000 – 210,000)	30,100 (-23,300 – 72,500)	16.1%	21.7	21.6	21.4
	4th	162,000 (144,000 – 182,000)	173,000 (156,000 – 190,000)	122,000 (89,000 – 167,000)	40,100 (-6,310 – 75,900)	24.7%	18.8	18.4	16.7
	5th	101,000 (88,600 – 115,000)	103,000 (92,400 – 115,000)	80,900 (58,900 – 111,000)	19,900 (-12,800 – 44,800)	19.7%	11.7	11.0	11.0
Pakistan	1st	135,000 (120,000 – 150,000)	119,000 (106,000 – 133,000)	106,000 (79,300 – 141,000)	28,800 (-7,360 – 57,700)	21.4%	23.1	24.1	25.0
	2nd	135,000 (123,000 – 149,000)	115,000 (104,000 – 126,000)	99,100 (75,300 – 130,000)	36,400 (3,520 – 63,200)	26.9%	23.2	23.2	23.4
	3rd	125,000 (116,000 – 134,000)	106,000 (98,300 – 113,000)	88,900 (68,200 – 116,000)	36,000 (7,930 – 58,100)	28.8%	21.4	21.4	21.0
	4th	111,000 (99,700 – 123,000)	88,300 (79,100 – 98,000)	74,000 (55,600 – 98,400)	37,300 (11,300 – 58,500)	33.5%	19.1	17.9	17.5
	5th	77,600 (66,900 – 89,900)	66,600 (57,500 – 76,900)	55,900 (41,000 – 75,700)	21,700 (1,470 – 39,100)	28.0%	13.3	13.5	13.2
Paraguay	1st	1,630 (1,360 – 1,930)	1,240 (1,000 – 1,510)	745 (444 – 1,220)	889 (387 – 1,290)	54.4%	25.2	25.7	26.7
	2nd	1,540 (1,330 – 1,780)	1,150 (954 – 1,370)	656 (395 – 1,060)	887 (453 – 1,220)	57.5%	23.8	23.7	23.5
	3rd	1,350 (1,190 – 1,540)	996 (843 – 1,170)	571 (346 – 924)	782 (418 – 1,060)	57.8%	20.8	20.6	20.5
	4th	1,140 (970 – 1,340)	842 (684 – 1,020)	466 (280 – 760)	679 (369 – 939)	59.3%	17.7	17.4	16.7
	5th	813 (603 – 1,070)	606 (441 – 812)	353 (198 – 606)	461 (205 – 692)	56.7%	12.5	12.5	12.6
Peru	1st	15,500 (14,100 – 16,900)	7,390 (6,640 – 8,150)	2,940 (2,210 – 3,960)	12,500 (10,900 – 14,100)	80.9%	29.5	30.7	31.3
	2nd	13,100 (11,900 – 14,300)	6,030 (5,440 – 6,680)	2,230 (1,690 – 2,990)	10,800 (9,500 – 12,100)	82.7%	25.0	25.0	23.8
	3rd	10,700 (9,820 – 11,500)	4,750 (4,310 – 5,200)	1,860 (1,410 – 2,470)	8,810 (7,810 – 9,760)	82.6%	20.4	19.7	19.8
	4th	8,050 (7,200 – 8,940)	3,570 (3,140 – 4,020)	1,400 (1,020 – 1,910)	6,650 (5,710 – 7,610)	82.6%	15.4	14.8	14.9
	5th	5,080 (4,360 – 5,860)	2,340 (1,990 – 2,740)	959 (696 – 1,340)	4,120 (3,350 – 4,890)	81.0%	9.7	9.7	10.2
Philippines	1st	35,000 (31,400 – 38,900)	27,600 (24,600 – 30,900)	19,600 (14,100 – 27,200)	15,400 (7,410 – 21,700)	44.0%	30.1	30.6	30.4
	2nd	27,400 (24,600 – 30,400)	21,500 (19,200 – 24,100)	15,000 (10,800 – 20,500)	12,400 (6,390 – 17,200)	45.2%	23.6	23.9	23.3
	3rd	23,400 (21,300 – 25,600)	17,800 (16,100 – 19,700)	12,800 (9,290 – 17,400)	10,600 (5,750 – 14,500)	45.3%	20.1	19.8	19.8
	4th	18,100 (15,900 – 20,400)	14,100 (12,300 – 16,000)	10,400 (7,400 – 14,500)	7,750 (3,340 – 11,300)	42.8%	15.6	15.6	16.1
	5th	12,200 (10,300 – 14,500)	9,150 (7,640 – 10,900)	6,730 (4,670 – 9,650)	5,490 (2,510 – 8,160)	44.9%	10.5	10.1	10.4
Republic of Moldova	1st	715 (560 – 887)	402 (312 – 512)	183 (123 – 277)	532 (364 – 704)	74.4%	26.0	25.9	27.0
	2nd	636 (521 – 768)	358 (288 – 443)	155 (106 – 232)	481 (344 – 618)	75.7%	23.1	23.1	22.9
	3rd	550 (459 – 652)	310 (251 – 376)	134 (93 – 199)	415 (306 – 523)	75.5%	20.0	20.0	19.9
	4th	459 (373 – 561)	258 (204 – 324)	107 (71 – 162)	352 (250 – 455)	76.6%	16.7	16.6	15.9
	5th	391 (282 – 531)	222 (160 – 308)	96 (61 – 157)	295 (189 – 419)	75.4%	14.2	14.3	14.3
Rwanda	1st	9,520 (8,440 – 10,700)	11,700 (10,500 – 13,200)	3,530 (2,220 – 5,610)	5,990 (3,680 – 7,690)	62.9%	19.8	20.5	24.9
	2nd	10,700 (9,680 – 11,800)	12,200 (10,900 – 13,600)	3,190 (2,030 – 5,000)	7,490 (5,430 – 9,070)	70.1%	22.3	21.3	22.5
	3rd	10,000 (9,200 – 10,900)	11,900 (10,800 – 13,100)	2,830 (1,800 – 4,420)	7,170 (5,430 – 8,480)	71.7%	20.8	20.8	20.0
	4th	10,000 (9,030 – 11,200)	12,500 (11,200 – 13,900)	2,530 (1,600 – 4,000)	7,520 (5,780 – 8,940)	74.9%	20.9	21.8	17.8
	5th	7,720 (6,840 – 8,730)	8,930 (7,860 – 10,100)	2,090 (1,310 – 3,350)	5,630 (4,150 – 6,880)	72.9%	16.1	15.6	14.8
Sao Tome and Principe	1st	106 (86 – 129)	103 (83 – 126)	56 (36 – 85)	50 (17 – 78)	47.2%	22.7	23.1	24.8
	2nd	102 (85 – 119)	98 (82 – 117)	51 (34 – 77)	50 (21 – 75)	49.2%	21.8	22.0	22.7
	3rd	96 (82 – 111)	92 (78 – 107)	45 (29 – 67)	51 (26 – 72)	53.1%	20.6	20.4	19.8
	4th	81 (67 – 97)	78 (64 – 93)	38 (24 – 56)	44 (21 – 64)	54.0%	17.5	17.3	16.6
	5th	81 (63 – 103)	77 (60 – 98)	36 (23 – 57)	45 (21 – 67)	55.6%	17.4	17.2	16.1
Senegal	1st	11,200 (10,100 – 12,400)	13,000 (11,800 – 14,300)	6,990 (5,440 – 9,020)	4,230 (1,930 – 6,160)	37.7%	25.5	25.8	27.6
	2nd	10,200 (9,260 – 11,200)	11,600 (10,600 – 12,700)	5,970 (4,630 – 7,690)	4,210 (2,270 – 5,850)	41.4%	23.1	23.1	23.6
	3rd	9,330 (8,640 – 10,000)	10,700 (9,810 – 11,500)	5,140 (4,040 – 6,550)	4,190 (2,670 – 5,450)	44.9%	21.2	21.2	20.3
	4th	7,870 (6,980 – 8,780)	8,850 (7,900 – 9,870)	4,230 (3,280 – 5,480)	3,640 (2,150 – 4,930)	46.3%	17.9	17.6	16.7
	5th	5,400 (4,660 – 6,260)	6,180 (5,420 – 7,060)	2,960 (2,240 – 3,920)	2,440 (1,290 – 3,470)	45.2%	12.3	12.3	11.7

Continued on next page

Absolute decline: wealth quintile-specific under-5 deaths (1990) - wealth quintile-specific under-5 deaths (2016).
Percentage decline: absolute decline over wealth quintile-specific under-5 deaths (1990) $\times$100.
Share of total death (in percentage): wealth quintile-specific under-5 deaths over the total under-5 deaths $\times$ 100.

Table 9 – continued from previous page

	Wealth Quintile	number of wealth quintile-specific under-5 deaths 1990	2000	2016	Absolute decline 1990–2016	Percentage decline 1990–2016	Share of total death (in percentage) 1990	2000	2016
Serbia	1st	1,080 (894 – 1,280)	414 (339 – 498)	148 (118 – 186)	928 (757 – 1,110)	86.2%	26.5	27.3	27.3
	2nd	939 (826 – 1,060)	348 (298 – 396)	125 (104 – 149)	815 (702 – 925)	86.8%	23.1	22.9	23.0
	3rd	813 (737 – 887)	302 (269 – 333)	108 (93 – 127)	705 (634 – 773)	86.7%	20.0	19.9	19.9
	4th	679 (581 – 785)	242 (196 – 287)	87 (69 – 108)	593 (498 – 693)	87.3%	16.7	16.0	16.0
	5th	560 (418 – 743)	210 (154 – 281)	75 (54 – 104)	485 (356 – 649)	86.6%	13.8	13.9	13.8
Sierra Leone	1st	10,600 (9,210 – 12,200)	10,500 (9,380 – 11,700)	6,650 (5,050 – 8,470)	3,970 (1,850 – 6,060)	37.4%	21.4	21.8	23.0
	2nd	10,400 (9,130 – 11,800)	9,980 (8,940 – 11,100)	6,130 (4,700 – 7,730)	4,300 (2,420 – 6,120)	41.3%	21.0	20.8	21.2
	3rd	10,200 (9,100 – 11,200)	9,800 (8,880 – 10,800)	5,900 (4,560 – 7,400)	4,260 (2,610 – 5,830)	41.9%	20.5	20.4	20.4
	4th	10,400 (9,080 – 11,800)	9,940 (8,870 – 11,100)	5,260 (4,000 – 6,720)	5,140 (3,330 – 6,960)	49.4%	20.9	20.7	18.2
	5th	8,060 (6,820 – 9,370)	7,820 (6,880 – 8,870)	4,980 (3,760 – 6,410)	3,080 (1,320 – 4,690)	38.2%	16.2	16.3	17.2
Somalia	1st	13,200 (10,100 – 17,100)	15,900 (11,800 – 21,600)	18,000 (9,780 – 33,500)	-4,840 (-19,000 – 2,690)	-36.7%	21.6	21.6	22.8
	2nd	13,900 (11,100 – 17,400)	16,700 (12,900 – 21,900)	18,000 (9,900 – 32,900)	-4,100 (-17,600 – 3,270)	-29.6%	22.7	22.7	22.7
	3rd	12,900 (10,600 – 16,000)	15,600 (12,100 – 20,300)	16,700 (9,260 – 30,600)	-3,820 (-16,500 – 2,920)	-29.5%	21.2	21.2	21.2
	4th	12,200 (9,680 – 15,400)	14,600 (11,100 – 19,400)	14,900 (8,200 – 27,600)	-2,770 (-14,200 – 3,460)	-22.8%	19.9	19.9	18.9
	5th	8,870 (6,330 – 12,300)	10,700 (7,420 – 15,500)	11,400 (5,960 – 22,100)	-2,510 (-11,800 – 2,310)	-28.3%	14.5	14.6	14.4
South Africa	1st	17,200 (14,300 – 20,600)	19,800 (16,600 – 23,300)	14,000 (11,200 – 17,200)	3,190 (-374 – 6,980)	18.6%	27.4	27.0	27.7
	2nd	14,700 (12,500 – 17,300)	17,100 (14,700 – 19,500)	11,900 (9,790 – 14,300)	2,810 (-32 – 5,930)	19.1%	23.4	23.3	23.5
	3rd	12,700 (11,000 – 14,700)	15,100 (13,200 – 17,000)	10,200 (8,530 – 12,100)	2,540 (312 – 4,920)	19.9%	20.3	20.5	20.2
	4th	10,700 (8,950 – 12,800)	12,700 (10,700 – 14,800)	8,530 (6,880 – 10,400)	2,160 (-77 – 4,530)	20.2%	17.1	17.3	16.8
	5th	7,370 (5,540 – 9,700)	8,720 (6,540 – 11,300)	6,000 (4,320 – 8,110)	1,360 (-348 – 3,250)	18.5%	11.8	11.9	11.9
South Sudan	1st	13,100 (10,100 – 16,500)	9,850 (7,800 – 12,300)	8,370 (5,030 – 13,600)	4,750 (-1,010 – 9,300)	36.2%	19.6	19.6	21.7
	2nd	14,400 (11,500 – 17,500)	10,800 (8,840 – 13,000)	8,330 (5,040 – 13,300)	6,080 (426 – 10,600)	42.2%	21.5	21.4	21.6
	3rd	13,500 (11,000 – 16,100)	10,100 (8,440 – 12,000)	7,700 (4,710 – 12,200)	5,820 (641 – 9,900)	43.0%	20.2	20.1	20.0
	4th	12,900 (10,200 – 15,700)	9,560 (7,760 – 11,600)	6,580 (3,980 – 10,500)	6,300 (1,480 – 10,100)	48.9%	19.3	19.0	17.1
	5th	12,900 (9,780 – 16,800)	9,960 (7,870 – 12,700)	7,500 (4,500 – 12,300)	5,420 (-1 – 9,940)	42.0%	19.3	19.8	19.5
State of Palestine	1st	1,070 (876 – 1,280)	935 (765 – 1,120)	785 (532 – 1,130)	286 (-68 – 578)	26.7%	25.8	26.2	27.0
	2nd	981 (852 – 1,120)	837 (723 – 961)	677 (468 – 974)	304 (-8 – 550)	31.0%	23.6	23.4	23.3
	3rd	849 (754 – 946)	726 (640 – 814)	590 (415 – 835)	259 (-4 – 455)	30.5%	20.4	20.3	20.3
	4th	712 (604 – 830)	612 (515 – 717)	480 (328 – 697)	233 (-1 – 417)	32.7%	17.2	17.1	16.5
	5th	537 (401 – 705)	463 (348 – 600)	377 (244 – 577)	160 (-25 – 325)	29.8%	12.9	13.0	13.0
Sudan	1st	23,500 (19,800 – 27,500)	24,700 (21,100 – 28,700)	19,700 (15,300 – 25,000)	3,800 (-1,960 – 9,200)	16.2%	22.1	22.6	23.7
	2nd	24,800 (21,800 – 28,000)	25,800 (22,700 – 29,000)	20,100 (15,900 – 25,100)	4,740 (-747 – 9,840)	19.1%	23.3	23.6	24.1
	3rd	23,000 (20,700 – 25,300)	23,700 (21,300 – 26,200)	17,600 (14,100 – 21,700)	5,410 (848 – 9,470)	23.5%	21.6	21.7	21.1
	4th	20,500 (17,700 – 23,400)	20,300 (17,600 – 23,200)	15,000 (11,800 – 19,000)	5,520 (892 – 9,680)	26.9%	19.3	18.6	18.0
	5th	14,500 (11,700 – 17,800)	14,900 (12,200 – 18,000)	10,900 (8,200 – 14,200)	3,600 (-43 – 7,090)	24.9%	13.6	13.6	13.1
Suriname	1st	142 (110 – 181)	104 (75 – 142)	58 (28 – 121)	84 (21 – 130)	59.0%	27.6	28.2	28.6
	2nd	119 (94 – 149)	84 (62 – 113)	46 (22 – 96)	73 (21 – 109)	61.2%	23.2	22.9	22.8
	3rd	104 (83 – 127)	73 (55 – 98)	40 (19 – 84)	63 (19 – 93)	60.8%	20.1	20.0	19.9
	4th	87 (68 – 110)	62 (45 – 84)	33 (15 – 69)	54 (15 – 81)	62.3%	16.8	16.8	16.2
	5th	63 (44 – 89)	45 (30 – 67)	25 (12 – 54)	38 (8 – 62)	60.2%	12.3	12.2	12.5
Swaziland	1st	592 (492 – 706)	976 (834 – 1,130)	684 (460 – 1,010)	-92 (-418 – 146)	-15.5%	25.3	24.1	25.4
	2nd	513 (434 – 598)	848 (736 – 967)	581 (393 – 859)	-68 (-343 – 125)	-13.3%	22.0	20.9	21.6
	3rd	464 (402 – 533)	823 (729 – 928)	536 (365 – 781)	-71 (-317 – 99)	-15.3%	19.9	20.3	19.9
	4th	408 (341 – 483)	759 (658 – 879)	479 (323 – 708)	-70 (-300 – 89)	-17.1%	17.5	18.7	17.8
	5th	358 (288 – 437)	651 (545 – 768)	416 (276 – 621)	-58 (-258 – 85)	-16.2%	15.3	16.0	15.4

Continued on next page

Absolute decline: wealth quintile-specific under-5 deaths (1990) - wealth quintile-specific under-5 deaths (2016).
Percentage decline: absolute decline over wealth quintile-specific under-5 deaths (1990) ×100.
Share of total death (in percentage): wealth quintile-specific under-5 deaths over the total under-5 deaths × 100.

Table 9 – continued from previous page

	Wealth Quintile	number of wealth quintile-specific under-5 deaths 1990	2000	2016	Absolute decline 1990–2016	Percentage decline 1990–2016	Share of total death (in percentage) 1990	2000	2016
Syrian Arab Republic	1st	4,130 (3,390 – 4,950)	3,020 (2,480 – 3,580)	1,930 (1,400 – 2,820)	2,200 (1,120 – 3,060)	53.3%	25.1	25.5	25.9
	2nd	3,870 (3,320 – 4,430)	2,760 (2,380 – 3,170)	1,710 (1,280 – 2,470)	2,150 (1,210 – 2,860)	55.6%	23.5	23.3	23.0
	3rd	3,340 (2,950 – 3,740)	2,400 (2,110 – 2,690)	1,500 (1,150 – 2,130)	1,830 (1,060 – 2,370)	54.8%	20.3	20.2	20.2
	4th	2,820 (2,380 – 3,300)	2,000 (1,680 – 2,350)	1,230 (898 – 1,800)	1,580 (877 – 2,140)	56.1%	17.1	16.9	16.5
	5th	2,290 (1,710 – 3,010)	1,670 (1,260 – 2,200)	1,080 (736 – 1,680)	1,210 (580 – 1,800)	52.9%	13.9	14.1	14.5
Tajikistan	1st	5,170 (4,350 – 6,100)	4,060 (3,350 – 4,900)	2,690 (1,590 – 4,720)	2,480 (417 – 3,780)	48.0%	23.2	23.2	25.0
	2nd	4,930 (4,250 – 5,710)	3,900 (3,280 – 4,620)	2,480 (1,480 – 4,290)	2,450 (564 – 3,620)	49.7%	22.1	22.4	23.1
	3rd	4,630 (4,070 – 5,270)	3,620 (3,090 – 4,240)	2,140 (1,290 – 3,700)	2,490 (880 – 3,500)	53.8%	20.8	20.7	19.9
	4th	3,900 (3,320 – 4,580)	2,980 (2,490 – 3,570)	1,750 (1,040 – 3,050)	2,150 (818 – 3,050)	55.1%	17.5	17.1	16.3
	5th	3,660 (2,960 – 4,490)	2,890 (2,320 – 3,600)	1,670 (979 – 2,970)	1,990 (648 – 2,970)	54.3%	16.4	16.6	15.6
Thailand	1st	10,600 (8,780 – 12,600)	5,630 (4,500 – 6,900)	2,380 (1,390 – 4,090)	8,240 (5,950 – 10,300)	77.6%	25.8	26.3	27.0
	2nd	9,750 (8,500 – 11,100)	5,040 (4,180 – 5,920)	2,060 (1,220 – 3,450)	7,700 (5,880 – 9,210)	79.0%	23.7	23.6	23.3
	3rd	8,360 (7,470 – 9,260)	4,320 (3,670 – 4,980)	1,780 (1,070 – 2,960)	6,580 (5,180 – 7,680)	78.7%	20.3	20.2	20.2
	4th	7,020 (5,950 – 8,180)	3,560 (2,910 – 4,310)	1,420 (834 – 2,400)	5,590 (4,230 – 6,830)	79.7%	17.0	16.7	16.2
	5th	5,430 (3,980 – 7,250)	2,840 (2,040 – 3,830)	1,170 (649 – 2,090)	4,260 (2,800 – 5,860)	78.5%	13.2	13.3	13.3
The former Yugoslav Republic of Macedonia	1st	358 (298 – 418)	122 (101 – 145)	80 (56 – 132)	277 (203 – 336)	77.5%	27.0	28.3	28.1
	2nd	307 (269 – 343)	98 (84 – 112)	65 (46 – 106)	241 (186 – 281)	78.6%	23.1	22.8	22.8
	3rd	264 (240 – 287)	85 (76 – 94)	57 (41 – 91)	208 (166 – 236)	78.7%	20.0	19.8	19.9
	4th	220 (188 – 252)	67 (55 – 80)	45 (31 – 73)	175 (133 – 207)	79.6%	16.6	15.7	15.9
	5th	176 (130 – 231)	57 (42 – 76)	38 (25 – 65)	138 (92 – 186)	78.6%	13.3	13.3	13.3
Timor-Leste	1st	1,160 (953 – 1,400)	972 (808 – 1,150)	535 (343 – 825)	630 (300 – 906)	54.1%	21.3	22.9	24.6
	2nd	1,270 (1,100 – 1,460)	992 (863 – 1,130)	524 (347 – 805)	745 (435 – 992)	58.7%	23.2	23.3	24.1
	3rd	1,180 (1,050 – 1,330)	922 (822 – 1,030)	455 (298 – 691)	730 (473 – 930)	61.6%	21.7	21.7	20.9
	4th	1,110 (950 – 1,290)	792 (680 – 921)	382 (247 – 591)	727 (476 – 936)	65.5%	20.3	18.6	17.5
	5th	742 (571 – 950)	574 (449 – 729)	282 (177 – 451)	460 (263 – 659)	62.0%	13.6	13.5	12.9
Togo	1st	5,250 (4,620 – 5,940)	5,580 (4,960 – 6,250)	5,020 (3,920 – 6,390)	233 (-1,200 – 1,460)	4.4%	23.5	25.1	26.2
	2nd	5,260 (4,700 – 5,870)	5,270 (4,710 – 5,870)	4,620 (3,630 – 5,820)	642 (-612 – 1,750)	12.2%	23.5	23.7	24.1
	3rd	4,900 (4,470 – 5,370)	4,890 (4,430 – 5,360)	4,130 (3,290 – 5,160)	777 (-255 – 1,660)	15.8%	21.9	22.0	21.5
	4th	4,310 (3,800 – 4,840)	3,980 (3,520 – 4,460)	3,350 (2,610 – 4,260)	965 (-2 – 1,820)	22.4%	19.3	17.9	17.4
	5th	2,630 (2,240 – 3,080)	2,510 (2,160 – 2,920)	2,080 (1,560 – 2,720)	558 (-95 – 1,150)	21.2%	11.8	11.3	10.8
Tunisia	1st	3,190 (2,560 – 3,930)	1,440 (1,140 – 1,800)	792 (535 – 1,130)	2,400 (1,730 – 3,130)	75.3%	25.9	26.8	27.9
	2nd	2,870 (2,400 – 3,400)	1,250 (1,020 – 1,510)	650 (446 – 919)	2,220 (1,680 – 2,790)	77.4%	23.3	23.2	22.9
	3rd	2,520 (2,160 – 2,920)	1,080 (902 – 1,280)	568 (395 – 790)	1,950 (1,520 – 2,380)	77.4%	20.5	20.1	20.0
	4th	2,120 (1,760 – 2,560)	907 (730 – 1,120)	455 (305 – 654)	1,660 (1,250 – 2,120)	78.4%	17.2	16.9	16.0
	5th	1,610 (1,170 – 2,210)	701 (503 – 969)	371 (236 – 569)	1,240 (829 – 1,780)	77.0%	13.1	13.0	13.1
Turkey	1st	30,600 (27,000 – 34,600)	16,400 (14,400 – 18,600)	5,070 (4,300 – 5,910)	25,500 (22,100 – 29,400)	83.3%	29.5	30.5	30.9
	2nd	23,500 (21,000 – 26,200)	12,500 (11,100 – 14,000)	3,750 (3,250 – 4,270)	19,800 (17,300 – 22,500)	84.2%	22.6	23.3	22.9
	3rd	21,100 (19,100 – 23,100)	10,600 (9,560 – 11,700)	3,240 (2,900 – 3,550)	17,800 (16,000 – 19,900)	84.5%	20.3	19.7	19.8
	4th	17,600 (15,400 – 20,000)	8,640 (7,490 – 9,920)	2,560 (2,090 – 3,030)	15,000 (12,900 – 17,400)	85.2%	17.0	16.1	15.6
	5th	11,000 (8,750 – 13,700)	5,570 (4,400 – 6,950)	1,760 (1,360 – 2,280)	9,260 (7,230 – 11,600)	84.0%	10.6	10.4	10.8
Turkmenistan	1st	2,810 (2,260 – 3,500)	2,280 (1,760 – 2,930)	1,950 (830 – 4,150)	856 (-1,330 – 2,070)	30.5%	25.3	25.6	26.9
	2nd	2,510 (2,080 – 2,990)	2,020 (1,600 – 2,540)	1,670 (720 – 3,560)	833 (-1,010 – 1,860)	33.2%	22.6	22.6	23.0
	3rd	2,300 (1,940 – 2,710)	1,850 (1,490 – 2,310)	1,470 (629 – 3,110)	836 (-799 – 1,720)	36.3%	20.8	20.7	20.2
	4th	1,940 (1,580 – 2,360)	1,550 (1,220 – 1,980)	1,210 (511 – 2,600)	736 (-624 – 1,490)	37.9%	17.5	17.4	16.6
	5th	1,520 (1,100 – 2,050)	1,220 (852 – 1,710)	966 (398 – 2,130)	559 (-546 – 1,230)	36.7%	13.8	13.7	13.3

Continued on next page

Absolute decline: wealth quintile-specific under-5 deaths (1990) - wealth quintile-specific under-5 deaths (2016).
Percentage decline: absolute decline over wealth quintile-specific under-5 deaths (1990) ×100.
Share of total death (in percentage): wealth quintile-specific under-5 deaths over the total under-5 deaths × 100.

Table 9 – continued from previous page

	Wealth Quintile	number of wealth quintile-specific under-5 deaths 1990	2000	2016	Absolute decline 1990–2016	Percentage decline 1990–2016	Share of total death (in percentage) 1990	2000	2016
Uganda	1st	32,100 (28,600 – 35,700)	42,200 (38,000 – 46,400)	22,900 (18,500 – 27,900)	9,180 (3,370 – 14,400)	28.6%	22.4	22.1	25.4
	2nd	30,500 (27,600 – 33,700)	40,400 (36,600 – 44,200)	20,400 (16,800 – 24,500)	10,100 (5,290 – 14,800)	33.1%	21.3	21.2	22.6
	3rd	29,100 (26,800 – 31,500)	38,500 (35,400 – 41,600)	17,700 (14,800 – 20,900)	11,400 (7,730 – 14,900)	39.2%	20.3	20.2	19.7
	4th	29,600 (26,600 – 33,000)	39,900 (36,100 – 44,000)	15,900 (13,000 – 19,300)	13,800 (9,510 – 17,900)	46.6%	20.7	21.0	17.6
	5th	21,800 (19,200 – 24,600)	29,700 (26,500 – 33,100)	13,100 (10,400 – 16,300)	8,680 (4,940 – 12,300)	39.8%	15.2	15.6	14.6
Ukraine	1st	3,470 (2,790 – 4,330)	2,050 (1,650 – 2,500)	1,180 (963 – 1,430)	2,290 (1,720 – 3,040)	65.9%	27.0	27.1	27.3
	2nd	2,970 (2,500 – 3,550)	1,740 (1,470 – 2,040)	1,000 (856 – 1,150)	1,960 (1,530 – 2,510)	66.0%	23.1	23.0	23.1
	3rd	2,590 (2,240 – 3,030)	1,520 (1,330 – 1,750)	872 (774 – 972)	1,720 (1,390 – 2,130)	66.4%	20.1	20.1	20.1
	4th	2,110 (1,710 – 2,580)	1,240 (1,010 – 1,490)	702 (567 – 835)	1,400 (1,070 – 1,840)	66.4%	16.4	16.3	16.2
	5th	1,730 (1,260 – 2,360)	1,010 (732 – 1,380)	580 (424 – 772)	1,150 (782 – 1,660)	66.5%	13.4	13.4	13.3
United Republic of Tanzania	1st	37,000 (32,700 – 41,500)	36,500 (33,000 – 40,400)	26,100 (20,500 – 33,200)	10,900 (2,960 – 17,700)	29.5%	19.2	20.2	22.2
	2nd	44,600 (40,600 – 49,100)	40,600 (37,000 – 44,400)	26,600 (21,100 – 33,600)	18,100 (10,400 – 24,900)	40.6%	23.2	22.5	22.7
	3rd	40,900 (37,700 – 44,100)	38,500 (35,700 – 41,600)	23,900 (19,300 – 29,900)	16,900 (10,500 – 22,200)	41.4%	21.3	21.3	20.4
	4th	39,100 (35,100 – 43,400)	35,000 (31,700 – 38,600)	21,000 (16,600 – 26,700)	18,100 (11,400 – 24,000)	46.3%	20.4	19.4	17.9
	5th	30,500 (26,700 – 34,700)	30,000 (26,700 – 33,600)	19,700 (15,400 – 25,200)	10,900 (4,380 – 16,500)	35.7%	15.9	16.6	16.8
Uzbekistan	1st	11,300 (9,270 – 13,600)	8,160 (6,580 – 10,000)	3,890 (3,020 – 4,940)	7,410 (5,690 – 9,260)	65.6%	22.3	22.5	24.5
	2nd	11,700 (9,920 – 13,800)	8,440 (7,000 – 10,200)	3,660 (2,920 – 4,500)	8,080 (6,570 – 9,790)	68.9%	23.1	23.3	23.0
	3rd	10,400 (8,940 – 12,100)	7,410 (6,210 – 8,810)	3,170 (2,570 – 3,860)	7,260 (6,120 – 8,540)	69.6%	20.6	20.4	20.0
	4th	8,910 (7,430 – 10,600)	6,330 (5,180 – 7,740)	2,640 (2,070 – 3,300)	6,270 (5,030 – 7,710)	70.4%	17.6	17.5	16.6
	5th	8,330 (6,660 – 10,300)	5,940 (4,710 – 7,490)	2,520 (1,890 – 3,310)	5,810 (4,430 – 7,460)	69.7%	16.4	16.4	15.9
Vanuatu	1st	49 (38 – 61)	46 (36 – 57)	51 (32 – 79)	-2 (-30 – 19)	-4.1%	26.3	26.5	26.5
	2nd	43 (35 – 53)	40 (33 – 48)	44 (29 – 68)	-1 (-25 – 17)	-2.3%	23.4	23.3	23.3
	3rd	37 (31 – 45)	35 (29 – 42)	39 (25 – 58)	-1 (-22 – 14)	-2.7%	20.3	20.2	20.2
	4th	31 (25 – 39)	29 (23 – 36)	32 (21 – 50)	-1 (-19 – 13)	-3.2%	17.0	17.0	17.0
	5th	24 (17 – 33)	22 (16 – 31)	25 (15 – 41)	-1 (-15 – 10)	-4.2%	12.9	13.0	13.0
Viet Nam	1st	28,100 (23,800 – 32,700)	12,700 (10,200 – 15,500)	9,960 (8,050 – 12,500)	18,100 (13,900 – 22,600)	64.5%	28.4	29.3	29.3
	2nd	22,800 (20,000 – 26,100)	9,940 (8,020 – 12,000)	7,750 (6,360 – 9,610)	15,100 (11,900 – 18,300)	66.1%	23.1	22.9	22.8
	3rd	19,900 (17,700 – 22,200)	8,600 (7,060 – 10,200)	6,740 (5,650 – 8,190)	13,100 (10,800 – 15,400)	65.9%	20.1	19.8	19.8
	4th	16,300 (14,000 – 18,900)	7,070 (5,660 – 8,610)	5,490 (4,400 – 6,950)	10,800 (8,290 – 13,400)	66.3%	16.5	16.3	16.1
	5th	11,800 (9,150 – 15,000)	5,130 (3,800 – 6,770)	4,090 (3,010 – 5,580)	7,710 (5,460 – 10,400)	65.4%	11.9	11.8	12.0
Yemen	1st	19,400 (16,400 – 22,800)	17,600 (15,100 – 20,600)	13,100 (9,110 – 18,700)	6,330 (584 – 11,000)	32.6%	25.5	26.7	27.5
	2nd	17,200 (15,200 – 19,500)	14,900 (13,000 – 16,900)	11,100 (7,780 – 15,700)	6,160 (1,410 – 9,780)	35.7%	22.6	22.6	23.3
	3rd	16,100 (14,600 – 17,700)	13,900 (12,400 – 15,400)	9,720 (6,900 – 13,400)	6,420 (2,550 – 9,430)	39.8%	21.2	21.0	20.4
	4th	14,100 (12,300 – 16,200)	11,700 (10,100 – 13,500)	8,140 (5,610 – 11,500)	6,010 (2,460 – 8,950)	42.5%	18.6	17.7	17.1
	5th	9,300 (7,190 – 11,800)	7,880 (6,120 – 10,000)	5,550 (3,670 – 8,230)	3,740 (1,090 – 6,140)	40.2%	12.2	11.9	11.7
Zambia	1st	13,600 (12,300 – 15,100)	15,500 (14,000 – 17,200)	9,420 (6,730 – 12,900)	4,190 (646 – 7,220)	30.8%	21.6	21.4	24.2
	2nd	13,800 (12,500 – 15,200)	15,700 (14,200 – 17,300)	8,760 (6,270 – 11,900)	5,050 (1,790 – 7,860)	36.6%	21.9	21.7	22.5
	3rd	13,000 (12,000 – 14,100)	15,100 (13,900 – 16,500)	7,910 (5,740 – 10,700)	5,130 (2,290 – 7,520)	39.3%	20.7	20.9	20.3
	4th	13,000 (11,700 – 14,400)	14,500 (13,100 – 16,100)	6,840 (4,900 – 9,300)	6,160 (3,460 – 8,480)	47.4%	20.6	20.2	17.6
	5th	9,680 (8,550 – 10,900)	11,400 (10,100 – 12,800)	5,950 (4,210 – 8,160)	3,730 (1,300 – 5,730)	38.6%	15.3	15.8	15.3
Zimbabwe	1st	6,160 (5,330 – 7,040)	8,520 (7,510 – 9,640)	7,200 (5,360 – 9,380)	-1,040 (-3,320 – 871)	-16.9%	21.9	22.1	24.1
	2nd	6,230 (5,500 – 7,010)	8,430 (7,500 – 9,460)	6,820 (5,160 – 8,820)	-591 (-2,640 – 1,170)	-9.5%	22.2	21.8	22.8
	3rd	5,890 (5,290 – 6,520)	8,190 (7,380 – 9,060)	6,130 (4,710 – 7,890)	-244 (-2,000 – 1,240)	-4.1%	20.9	21.2	20.5
	4th	5,360 (4,700 – 6,100)	7,310 (6,440 – 8,240)	5,290 (4,000 – 6,920)	73 (-1,570 – 1,460)	1.4%	19.1	18.9	17.7
	5th	4,470 (3,810 – 5,200)	6,180 (5,320 – 7,090)	4,440 (3,300 – 5,820)	31 (-1,370 – 1,270)	0.7%	15.9	16.0	14.9

Absolute decline: wealth quintile-specific under-5 deaths (1990) - wealth quintile-specific under-5 deaths (2016).
Percentage decline: absolute decline over wealth quintile-specific under-5 deaths (1990) $\times 100$.
Share of total death (in percentage): wealth quintile-specific under-5 deaths over the total under-5 deaths $\times$ 100.

Table 10: **Estimates and uncertainty intervals for inequality indexs, for the 99 countries with empirical data.** Estimates and 90% uncertainty intervals for (i) difference in 1st and 5th wealth quintile-specific U5MR in 1990 and 2016; (ii) ratio of 1st to 5th wealth quintile-specific U5MR in 1990 and 2016; (iii) concentration index in 1990 and 2016; and (iv) slope inequality index in 1990 and 2016; for the 99 countries with empirical data. Numbers in brackets are 90% uncertainty intervals. §: change of ratio is significantly different from zero. ¶: point estimates of ratio < 1.5 in 2016. ‡: point estimates of ratio > 2.5 in 2016. Countries are ordered alphabetically.

	**Difference*** (deaths per 1000 livebirths)		**Ratio†**		**Concentration index** ($\times$ 100)		**Slope inequality index** (deaths per 1000 livebirths)	
	**1990**	**2016**	**1990**	**2016**	**1990**	**2016**	**1990**	**2016**
Afghanistan	84.5 (45.7 – 124.2)	45.1 (29.9 – 61.6)	1.72 (1.34 – 2.20)	1.99 (1.62 – 2.42)	-8.7 (-12.4 – -4.9)	-12.4 (-15.4 – -9.1)	-95.9 (-138.6 – -53.5)	-54.4 (-72.6 – -37.5)
Albania	21.7 (8.0 – 35.0)	8.3 (2.6 – 18.0)	1.78 (1.23 – 2.57)	1.88 (1.27 – 2.80)	-11.3 (-16.5 – -5.7)	-12.6 (-18.7 – -6.4)	-28.2 (-42.0 – -13.9)	-10.7 (-22.2 – -4.1)
Algeria	29.1 (14.3 – 43.3)	16.0 (8.5 – 23.5)	1.84 (1.34 – 2.53)	1.89 (1.39 – 2.57)	-11.9 (-16.7 – -6.9)	-12.4 (-17.1 – -7.5)	-36.4 (-51.0 – -20.9)	-19.6 (-27.4 – -11.8)
Angola	61.3 (15.2 – 108.2)	37.8 (13.5 – 78.2)	1.36 (1.08 – 1.72)	1.62 (1.24 – 2.12)	-5.4 (-9.0 – -1.9)	-9.4 (-13.5 – -5.1)	-75.2 (-126.5 – -25.9)	-48.6 (-95.0 – -19.6)
Armenia	33.0 (16.0 – 49.3)	9.8 (4.6 – 16.2)	2.02 (1.40 – 2.93)	2.11 (1.42 – 3.19)	-13.2 (-18.3 – -7.6)	-14.5 (-20.7 – -8.2)	-41 (-57.7 – -23.3)	-12.2 (-19.1 – -6.5)
Azerbaijan	48.7 (22.7 – 74.3)	19.9 (8.6 – 38.4)	1.76 (1.29 – 2.40)	1.99 (1.43 – 2.84)	-10.0 (-14.5 – -5.4)	-12.8 (-17.7 – -7.6)	-59.2 (-85.9 – -31.6)	-24.7 (-46.5 – -11.9)
Bangladesh	76.8 (59.9 – 93.6)	23.1 (16.0 – 30.4)	1.80 (1.58 – 2.04)	2.04 (1.66 – 2.51)	-10.3 (-12.4 – -8.3)	-13.3 (-16.6 – -9.9)	-93.0 (-111.9 – -74.6)	-28.4 (-36.3 – -20.9)
Belarus	10.8 (4.8 – 16.5)	2.8 (1.2 – 4.2)	2.07 (1.37 – 3.11)	2.08 (1.36 – 3.14)	-14.1 (-20.3 – -7.7)	-14.2 (-20.5 – -7.6)	-13.4 (-19.3 – -7.3)	-3.5 (-5.0 – -1.8)
Belize	25.6 (11.1 – 39.8)	10.6 (4.7 – 16.5)	2.00 (1.34 – 2.98)	2.08 (1.37 – 3.17)	-13.1 (-18.7 – -7.1)	-14.2 (-20.5 – -7.5)	-32.0 (-46.4 – -16.9)	-13.2 (-19.3 – -7.0)
Benin	71.1 (42.4 – 98.9)	49.6 (27.7 – 78.4)	1.56 (1.30 – 1.85)	1.74 (1.39 – 2.19)	-7.6 (-10.3 – -4.9)	-10.2 (-13.8 – -6.7)	-84.8 (-115.7 – -54.5)	-62.3 (-94.5 – -37.9)
Bhutan	86.3 (47.0 – 126.3)	28.1 (15.7 – 43.8)	2.10 (1.48 – 3.00)	2.49 (1.74 – 3.56)	-12.5 (-17.4 – -7.3)	-16.5 (-21.6 – -11.2)	-99.7 (-140.9 – -58.3)	-33.4 (-50.5 – -19.8)
Bolivia (Plurinational State of)‡	107.1 (89.4 – 125.3)	36.5 (23.3 – 53.6)	2.68 (2.29 – 3.15)	2.96 (2.31 – 3.77)	-15.9 (-18.3 – -13.6)	-18.8 (-22.4 – -14.9)	-123.1 (-143.0 – -103.4)	-43.4 (-62.9 – -28.2)
Brazil‡	57.6 (39.4 – 75.4)	14.3 (8.6 – 20.8)	2.58 (1.88 – 3.57)	2.64 (1.81 – 3.89)	-17.0 (-21.4 – -12.3)	-18.1 (-24.0 – -12.2)	-68.2 (-87 – -49.2)	-17.0 (-24.1 – -11.0)
Burkina Faso	61.8 (37.4 – 85.5)	41.5 (23.4 – 63.7)	1.42 (1.24 – 1.62)	1.70 (1.38 – 2.10)	-5.7 (-7.8 – -3.5)	-9.9 (-13.3 – -6.6)	-70.4 (-97.3 – -43.2)	-52.5 (-77.7 – -32.6)
Burundi	92.0 (51.2 – 132.9)	48.4 (27.5 – 71.9)	1.82 (1.40 – 2.36)	2.05 (1.54 – 2.70)	-9.8 (-13.7 – -5.8)	-13 (-17.2 – -8.6)	-104.7 (-147.8 – -61.5)	-58.4 (-84.1 – -35.6)
Cambodia‡	86.3 (64.3 – 108.4)	27.3 (15.4 – 46.1)	2.42 (1.94 – 3.01)	2.76 (2.11 – 3.57)	-13.6 (-16.7 – -10.4)	-16.9 (-20.6 – -13.0)	-98.4 (-122.3 – -74.5)	-32.4 (-54.1 – -18.8)
Cameroon	92.4 (69.1 – 115.8)	54.5 (33.2 – 80.2)	1.99 (1.68 – 2.35)	2.05 (1.60 – 2.59)	-12.2 (-14.9 – -9.5)	-13.2 (-16.9 – -9.3)	-109.4 (-135 – -84.4)	-65.5 (-93.8 – -42.4)
Central African Republic	81.0 (55.4 – 107.5)	71.4 (38.2 – 121.7)	1.68 (1.43 – 1.98)	1.86 (1.48 – 2.32)	-9.2 (-11.7 – -6.7)	-11.1 (-14.5 – -7.4)	-100.4 (-130.2 – -71.3)	-85.5 (-143.0 – -47.4)
Chad¶	-15.1 (-43.3 – 13.3)	10.4 (-11.3 – 34.9)	0.92 (0.79 – 1.07)	1.09 (0.91 – 1.32)	0.2 (-2.2 – 2.6)	-2.7 (-5.9 – 0.3)	2.3 (-29.4 – 34.3)	-21.2 (-47.8 – 2.3)
Colombia	23.0 (15.1 – 30.7)	11.0 (6.4 – 16.3)	1.95 (1.55 – 2.45)	2.04 (1.54 – 2.69)	-13.0 (-16.6 – -9.2)	-14.5 (-19.1 – -9.8)	-28.6 (-36.8 – -20.2)	-13.9 (-19.9 – -8.9)
Comoros	57.0 (25.5 – 88.4)	35.8 (12.1 – 80.9)	1.62 (1.24 – 2.11)	1.66 (1.23 – 2.20)	-9.0 (-13.0 – -4.9)	-9.9 (-14.5 – -5.2)	-70.9 (-104.2 – -37.2)	-45.4 (-99.5 – -17.9)
Congo	34.4 (12.7 – 56.2)	23.2 (9.5 – 39.8)	1.50 (1.16 – 1.96)	1.58 (1.22 – 2.06)	-8.0 (-11.9 – -4.0)	-9.3 (-13.3 – -5.3)	-45.7 (-68.2 – -22.5)	-31.5 (-50.3 – -16.0)
Cote d'Ivoire	80.9 (58.8 – 104.7)	50.0 (27.9 – 76.8)	1.80 (1.54 – 2.13)	1.78 (1.41 – 2.25)	-10.1 (-12.7 – -7.6)	-10.7 (-14.3 – -6.9)	-95.3 (-120.5 – -70.7)	-61.3 (-91.8 – -36.5)
Democratic Republic of the Congo	104.2 (70.7 – 139.5)	59.0 (35.0 – 89.3)	1.94 (1.57 – 2.39)	2.01 (1.59 – 2.51)	-10.1 (-13.2 – -7.0)	-11.9 (-15.4 – -8.3)	-116.5 (-154.1 – -78.4)	-70.4 (-105.1 – -43.1)
Dominican Republic	39.8 (26.0 – 52.9)	19.9 (11.7 – 30.3)	2.05 (1.59 – 2.63)	1.99 (1.53 – 2.59)	-13.0 (-16.6 – -9.1)	-12.8 (-16.8 – -8.7)	-48.7 (-62.8 – -33.9)	-24.6 (-36.4 – -15.3)
Egypt‡	77.3 (63.5 – 91.3)	20.1 (13.8 – 28.0)	2.69 (2.24 – 3.24)	2.55 (2.01 – 3.22)	-16.7 (-19.4 – -14.0)	-16.5 (-20.1 – -12.6)	-89.7 (-104.7 – -74.9)	-23.5 (-32.2 – -16.5)
El Salvador	36.3 (15.6 – 56.6)	10.9 (4.8 – 18.8)	1.90 (1.31 – 2.78)	2.08 (1.41 – 3.08)	-12.2 (-17.6 – -6.5)	-14.5 (-20.3 – -8.2)	-45.3 (-66.4 – -23.9)	-13.6 (-22.5 – -6.9)
Equatorial Guinea¶	33.8 (-14.8 – 80.4)	29.7 (2.8 – 61.5)	1.20 (0.93 – 1.53)	1.37 (1.03 – 1.85)	-3.5 (-7.6 – 0.8)	-7.0 (-11.9 – -2.0)	-41.9 (-92.6 – 9.7)	-39.7 (-75.2 – -10.7)
Eritrea	52.9 (17.6 – 89.0)	24.4 (10.7 – 44.7)	1.54 (1.15 – 2.10)	1.88 (1.35 – 2.63)	-6.8 (-10.8 – -2.7)	-11.4 (-16.1 – -6.6)	-64.2 (-103.7 – -25.8)	-31.7 (-55.7 – -16.0)
Ethiopia	29.2 (-6.1 – 64.4)	23.7 (10.9 – 38.7)	1.18 (0.97 – 1.44)	1.54 (1.23 – 1.94)	-2.4 (-5.5 – 0.7)	-8.5 (-12.3 – -4.8)	-30.9 (-69.5 – 8.7)	-31.2 (-48.3 – -16.8)
Gabon	39.8 (16.7 – 63.4)	25.0 (11.0 – 42.7)	1.62 (1.22 – 2.16)	1.77 (1.31 – 2.37)	-9.0 (-12.9 – -4.8)	-11.1 (-15.3 – -6.5)	-51.8 (-77.1 – -27.4)	-32.8 (-53.4 – -17.0)
Georgia	29.9 (13.9 – 46.1)	7.5 (3.4 – 11.7)	1.98 (1.36 – 2.93)	2.08 (1.39 – 3.15)	-12.9 (-18.3 – -7.2)	-14.3 (-20.3 – -7.9)	-38.1 (-55.0 – -21.1)	-9.5 (-14.1 – -5.2)
Ghana	66.8 (46.5 – 87.2)	33.2 (18.8 – 50.0)	1.75 (1.47 – 2.09)	1.77 (1.42 – 2.22)	-10.0 (-12.7 – -7.2)	-11.0 (-14.6 – -7.3)	-79.0 (-101.0 – -56.7)	-40.3 (-59.3 – -24.6)
Guatemala	36.0 (18.2 – 52.9)	17.4 (9.8 – 25.9)	1.63 (1.27 – 2.09)	1.93 (1.46 – 2.57)	-9.1 (-12.6 – -5.3)	-12.7 (-16.8 – -8.4)	-46.3 (-64.6 – -26.9)	-22.6 (-31.9 – -14.5)
Guinea	110.3 (69.3 – 151.6)	57.1 (37.4 – 81.6)	1.68 (1.39 – 2.02)	1.99 (1.61 – 2.45)	-8.8 (-11.7 – -5.7)	-12.1 (-15.4 – -8.7)	-128.7 (-174 – -83.6)	-67.2 (-93.5 – -45.7)
Guinea-Bissau	85.8 (45.1 – 127.8)	41.7 (20.4 – 68.6)	1.55 (1.27 – 1.90)	1.66 (1.32 – 2.09)	-7.8 (-10.9 – -4.6)	-9.7 (-13.3 – -6.0)	-106.2 (-153.1 – -61.4)	-53.3 (-84.0 – -29.0)
Guyana	22.6 (3.0 – 40.4)	13.3 (2.3 – 26.5)	1.49 (1.05 – 2.07)	1.54 (1.09 – 2.17)	-8.4 (-13.3 – -3.1)	-9.2 (-14.2 – -3.7)	-31.6 (-50.1 – -11.3)	-18.6 (-33.9 – -6.8)
Haiti	51.0 (25.9 – 75.4)	31.9 (16.5 – 50.5)	1.48 (1.21 – 1.78)	1.67 (1.32 – 2.12)	-6.9 (-9.8 – -3.9)	-9.7 (-13.3 – -6.0)	-62.9 (-89.7 – -35.6)	-40.5 (-61.5 – -23.4)
Honduras	44.4 (28.8 – 60.0)	15.9 (9.6 – 23.9)	2.25 (1.69 – 3.01)	2.38 (1.76 – 3.22)	-14.7 (-18.9 – -10.3)	-16.2 (-20.7 – -11.5)	-53.4 (-69.9 – -37.2)	-18.9 (-27.8 – -12.0)
India‡	108.0 (97.7 – 118.4)	39.1 (29.5 – 49.0)	2.75 (2.51 – 3.01)	2.78 (2.19 – 3.47)	-17.2 (-18.6 – -15.9)	-17.9 (-21.4 – -14.2)	-135.6 (-147.2 – -124.1)	-48.1 (-58.7 – -37.3)
Indonesia‡	72.0 (55.0 – 89.4)	24.5 (16.9 – 33.8)	2.52 (2.01 – 3.18)	2.65 (2.07 – 3.42)	-15.7 (-19 – -12.3)	-17.3 (-21.1 – -13.4)	-82.5 (-100.9 – -64.3)	-28.5 (-38.8 – -20.3)
Iraq¶	16.7 (1.2 – 32.2)	9.5 (1.3 – 18.9)	1.36 (1.02 – 1.82)	1.35 (1.05 – 1.76)	-7.1 (-11.9 – -2.3)	-7.0 (-11.3 – -2.6)	-24.2 (-40.5 – -7.8)	-13.7 (-24.1 – -4.8)
Jordan	18.3 (10.5 – 26.4)	10.0 (4.5 – 17.0)	1.68 (1.34 – 2.12)	1.77 (1.32 – 2.39)	-10.0 (-13.6 – -6.3)	-11.6 (-16.5 – -6.6)	-22.8 (-31.4 – -14.3)	-12.8 (-20.7 – -6.7)

Continued on next page

*: Difference in 1st and 5th wealth quintile-specific U5MR.
†: Ratio of 1st to 5th wealth quintile-specific U5MR.

Table 10 – continued from previous page

	Difference* (deaths per 1000 livebirths)		Ratio†		Concentration index (× 100)		Slope inequality index (deaths per 1000 livebirths)	
	1990	2016	1990	2016	1990	2016	1990	2016
Kazakhstan	26.8 (12.2 – 40.8)	6.8 (3.1 – 10.7)	1.73 (1.28 – 2.33)	1.85 (1.31 – 2.64)	-10.8 (-15.1 – -6.2)	-12.5 (-18.1 – -6.8)	-35.2 (-49.9 – -19.9)	-8.9 (-12.9 – -4.8)
Kenya	53.3 (37.9 – 69.3)	23.3 (12.8 – 35.0)	1.74 (1.48 – 2.05)	1.63 (1.32 – 2.01)	-11.2 (-13.9 – -8.5)	-9.8 (-13.2 – -6.2)	-68.6 (-85.9 – -51.6)	-30.2 (-43.4 – -18.7)
Kyrgyz Republic	35.2 (16.6 – 53.3)	13.4 (6.8 – 19.8)	1.75 (1.30 – 2.34)	1.90 (1.38 – 2.61)	-10.9 (-15.3 – -6.1)	-12.8 (-17.9 – -7.6)	-44.6 (-63.4 – -24.7)	-16.9 (-23.8 – -10.0)
Lao People's Democratic Republic‡	111.6 (69.5 – 156.2)	55.6 (34.3 – 83.9)	2.32 (1.68 – 3.24)	2.77 (2.02 – 3.79)	-12.4 (-16.8 – -8.0)	-16.5 (-20.6 – -12.1)	-125.2 (-172.3 – -79.9)	-65.8 (-97.1 – -41.7)
Lesotho¶	32.3 (12.4 – 52.4)	32.3 (11.1 – 57.9)	1.46 (1.15 – 1.84)	1.45 (1.14 – 1.86)	-7.3 (-11.1 – -3.6)	-7.0 (-10.7 – -3.1)	-41.8 (-64.0 – -20.4)	-40.7 (-68.0 – -17.6)
Liberia¶	28.5 (-33.3 – 92.1)	19.1 (2.8 – 37.9)	1.12 (0.88 – 1.46)	1.32 (1.04 – 1.67)	-2.4 (-6.5 – 1.7)	-6.4 (-10.3 – -2.3)	-38.3 (-105.9 – 27.0)	-26.8 (-47.8 – -9.4)
Madagascar	90.5 (63.4 – 117.5)	35.0 (20.8 – 54.7)	1.94 (1.58 – 2.36)	2.29 (1.76 – 2.97)	-10.4 (-13.3 – -7.5)	-14.8 (-18.6 – -10.9)	-104.1 (-133.6 – -73.8)	-42.8 (-65.3 – -26.7)
Malawi	67.2 (37.2 – 97.3)	23.3 (12.5 – 36.3)	1.38 (1.20 – 1.60)	1.56 (1.28 – 1.89)	-5.1 (-7.3 – -2.8)	-9.1 (-12.2 – -5.9)	-73.7 (-106.6 – -40.3)	-31.4 (-46.5 – -19.1)
Maldives	48.4 (15.9 – 79.4)	5.8 (2.4 – 9.5)	1.74 (1.19 – 2.54)	2.01 (1.34 – 3.03)	-10.2 (-15.6 – -4.3)	-13.7 (-19.8 – -7.2)	-59.9 (-92.5 – -25.2)	-7.3 (-11.3 – -3.7)
Mali	108.1 (78.1 – 137.5)	64.5 (35.1 – 109.1)	1.63 (1.43 – 1.86)	1.94 (1.59 – 2.38)	-7.6 (-9.6 – -5.5)	-11 (-14.1 – -7.9)	-120.7 (-153.4 – -87.5)	-76.3 (-127.6 – -42.2)
Mauritania	62.0 (31.8 – 92.4)	45.6 (20.4 – 90.3)	1.74 (1.33 – 2.29)	1.78 (1.38 – 2.28)	-10.3 (-14.5 – -6.0)	-11.2 (-15.1 – -7.0)	-74.9 (-107.1 – -43.8)	-56.8 (-109.5 – -27.1)
Mongolia	66.3 (40.4 – 91.6)	14.9 (8.4 – 24.4)	1.92 (1.49 – 2.47)	2.35 (1.74 – 3.20)	-12.0 (-15.8 – -8.1)	-16.5 (-21.3 – -11.7)	-81.5 (-108.8 – -54.7)	-18.4 (-29.2 – -10.8)
Morocco‡	63.9 (47.7 – 80.2)	23.9 (14.9 – 35.7)	2.52 (1.99 – 3.19)	2.64 (1.96 – 3.56)	-15.3 (-18.7 – -12.0)	-17 (-21.3 – -12.5)	-76.4 (-94.2 – -59.2)	-28.8 (-42.3 – -18.5)
Mozambique	83.2 (41.0 – 126.9)	32.6 (17.3 – 52.3)	1.45 (1.20 – 1.76)	1.61 (1.31 – 1.96)	-6.3 (-9.3 – -3.2)	-9.1 (-12.5 – -5.8)	-97.0 (-144.3 – -49.9)	-40.7 (-62.9 – -23.8)
Myanmar	61.5 (22.8 – 97.7)	33.6 (16.0 – 52.7)	1.79 (1.23 – 2.61)	2.03 (1.41 – 2.93)	-10.4 (-15.5 – -4.9)	-13.4 (-18.3 – -7.9)	-74.8 (-112 – -35.2)	-42.4 (-63.2 – -23.5)
Namibia	32.5 (18.5 – 46.6)	26.8 (14.2 – 44.8)	1.66 (1.33 – 2.07)	1.91 (1.47 – 2.51)	-9.3 (-12.5 – -5.9)	-11.9 (-16.0 – -7.9)	-41.6 (-56.7 – -26.6)	-33.7 (-54.4 – -19.1)
Nepal	59.0 (36.7 – 81.3)	21.9 (13.7 – 31.0)	1.62 (1.35 – 1.95)	1.99 (1.56 – 2.53)	-8.1 (-10.8 – -5.4)	-12.9 (-16.6 – -9.1)	-71.5 (-96.0 – -47.2)	-27.7 (-38.1 – -18.7)
Nicaragua	39.8 (23.9 – 55.8)	13.9 (6.6 – 25.6)	1.96 (1.48 – 2.59)	2.15 (1.53 – 3.04)	-12.0 (-15.7 – -7.9)	-14.2 (-19.2 – -8.9)	-50.4 (-67.4 – -33.2)	-17.5 (-31 – -8.9)
Niger¶	71.4 (29.1 – 116.2)	29.6 (11.6 – 52.6)	1.31 (1.11 – 1.54)	1.44 (1.16 – 1.79)	-4.6 (-7.1 – -2.2)	-6.9 (-10.1 – -3.5)	-94.3 (-146.9 – -46.1)	-39.2 (-66.7 – -18.4)
Nigeria	129.0 (96.9 – 161.9)	82.6 (56.0 – 116.4)	2.04 (1.72 – 2.39)	2.44 (2.01 – 2.91)	-11.7 (-14.3 – -9.1)	-15.6 (-18.4 – -12.6)	-156.0 (-193.1 – -119.8)	-101.6 (-142.4 – -70.3)
Pakistan	67.8 (43.0 – 91.8)	46.4 (27.8 – 69.8)	1.73 (1.42 – 2.11)	1.89 (1.49 – 2.40)	-9.5 (-12.5 – -6.4)	-11.8 (-15.4 – -8.2)	-82.2 (-108.5 – -55.3)	-58 (-84.3 – -37.5)
Paraguay	29.4 (16.3 – 42.5)	14.0 (6.0 – 25.9)	2.01 (1.44 – 2.85)	2.11 (1.45 – 3.09)	-12.6 (-17.1 – -7.8)	-14 (-19.4 – -8.2)	-36.6 (-50.4 – -22.5)	-17.4 (-31.2 – -8.5)
Peru‡	79.7 (67.5 – 91.8)	16.1 (11.1 – 22.3)	3.04 (2.56 – 3.61)	3.06 (2.32 – 3.99)	-19.7 (-22.1 – -17.2)	-20.4 (-24.6 – -16.0)	-98.9 (-112.4 – -85.7)	-19.5 (-27.1 – -13.7)
Philippines‡	57.0 (46.5 – 67.4)	27.1 (18.0 – 38.9)	2.86 (2.37 – 3.47)	2.91 (2.24 – 3.76)	-18.9 (-21.7 – -16.0)	-18.9 (-22.6 – -14.8)	-68.6 (-79.9 – -57.6)	-32.0 (-45.7 – -21.8)
Republic of Moldova	19.4 (7.0 – 31.3)	10.1 (3.5 – 18.9)	1.83 (1.23 – 2.65)	1.89 (1.25 – 2.78)	-12.0 (-17.5 – -5.7)	-13.0 (-19 – -6.4)	-24.7 (-37.3 – -11.8)	-13 (-22.6 – -5.8)
Rwanda§	28.2 (5.7 – 49.8)	19.5 (9.6 – 34.6)	1.23 (1.04 – 1.45)	1.69 (1.36 – 2.12)	-3.5 (-6.1 – -0.9)	-10.0 (-13.8 – -6.3)	-33.2 (-57.3 – -8.4)	-24.1 (-41.6 – -12.5)
Sao Tome and Principe	28.1 (-4.9 – 59.9)	14.7 (3.5 – 28.6)	1.31 (0.95 – 1.78)	1.54 (1.11 – 2.12)	-6.0 (-10.9 – -0.8)	-9.4 (-14.6 – -4.0)	-39.4 (-72.3 – -5.3)	-19.9 (-35.2 – -7.7)
Senegal	92.9 (69.3 – 116.5)	37.5 (26.4 – 51.6)	2.08 (1.73 – 2.50)	2.36 (1.90 – 2.92)	-12.7 (-15.5 – -9.8)	-15.5 (-18.6 – -12.1)	-111.3 (-136.9 – -85.7)	-45.6 (-61.7 – -32.7)
Serbia	17.9 (7.3 – 28.0)	3.9 (1.5 – 6.4)	1.92 (1.29 – 2.84)	1.98 (1.30 – 3.00)	-12.7 (-18.5 – -6.4)	-13.6 (-19.9 – -6.9)	-22.4 (-32.7 – -11.3)	-4.9 (-7.5 – -2.5)
Sierra Leone¶	67.5 (21.4 – 116.1)	32.8 (10.5 – 56.6)	1.32 (1.09 – 1.61)	1.34 (1.10 – 1.61)	-4.1 (-7.3 – -1.0)	-5.8 (-9.0 – -2.5)	-67.8 (-119.8 – -16.5)	-41.3 (-67.2 – -16.9)
Somalia	64.1 (3.9 – 124.4)	55.7 (9.0 – 127.7)	1.49 (1.02 – 2.18)	1.58 (1.08 – 2.29)	-6.8 (-12 – -1.2)	-8.3 (-13.5 – -2.5)	-76.6 (-141.6 – -13.6)	-68.3 (-150.1 – -17.7)
South Africa	45.0 (27.8 – 62.2)	34.2 (19.9 – 49.2)	2.33 (1.68 – 3.24)	2.33 (1.64 – 3.35)	-15.1 (-19.5 – -10.3)	-15.3 (-20.2 – -10.1)	-54.1 (-72.3 – -36.4)	-41.4 (-57 – -26.2)
South Sudan¶	3.9 (-78.0 – 81.9)	10.2 (-18.6 – 40.8)	1.02 (0.74 – 1.39)	1.12 (0.83 – 1.49)	-1.2 (-6.2 – 4.2)	-3.6 (-8.5 – 1.5)	-18.5 (-100.9 – 66.4)	-20.5 (-54.6 – 9.5)
State of Palestine	28.7 (13.7 – 43.2)	13.6 (6.5 – 22.8)	2.00 (1.38 – 2.89)	2.08 (1.45 – 3.01)	-12.9 (-18.1 – -7.3)	-13.9 (-19.5 – -8.3)	-35.9 (-50.9 – -20.3)	-16.9 (-27.2 – -9.3)
Sudan	55.8 (23.5 – 87.8)	34.5 (19.7 – 51.0)	1.62 (1.22 – 2.16)	1.81 (1.43 – 2.30)	-8.4 (-12.5 – -4.3)	-10.9 (-14.4 – -7.3)	-69.2 (-103.6 – -35.0)	-44.5 (-63.2 – -28.3)
Suriname	35.6 (19.1 – 53.2)	16.1 (6.3 – 35.6)	2.25 (1.55 – 3.28)	2.29 (1.57 – 3.43)	-14.8 (-20.1 – -9.3)	-15.5 (-21.4 – -9.5)	-43 (-61.8 – -25.5)	-19.4 (-42.4 – -8.0)
Swaziland	33.0 (17.1 – 50.3)	35.0 (16.9 – 59.9)	1.65 (1.30 – 2.13)	1.64 (1.31 – 2.10)	-9.8 (-14 – -5.8)	-9.5 (-13.4 – -5.6)	-40.3 (-58.8 – -23.3)	-41.7 (-69.5 – -22.1)
Syria	20.8 (7.9 – 33.0)	10.0 (3.4 – 18.2)	1.81 (1.25 – 2.60)	1.79 (1.22 – 2.63)	-11.5 (-16.8 – -5.9)	-11.7 (-17.7 – -5.4)	-26.7 (-39.3 – -13.6)	-12.9 (-21.9 – -5.7)
Tajikistan	36.2 (10.1 – 62.3)	20.4 (7.3 – 39.9)	1.41 (1.10 – 1.81)	1.61 (1.21 – 2.11)	-7.3 (-11.2 – -3.2)	-10.3 (-14.7 – -5.6)	-48.6 (-76.4 – -21.3)	-27.6 (-51.5 – -12.4)
Thailand	23.8 (10.3 – 36.6)	8.4 (3.1 – 16.5)	1.96 (1.32 – 2.90)	2.03 (1.35 – 3.13)	-12.8 (-18.2 – -6.7)	-13.9 (-20.2 – -7.4)	-30.1 (-43 – -15.9)	-10.6 (-19.9 – -4.8)
The former Yugoslav Republic of Macedonia	25.5 (11.9 – 37.8)	9.0 (4.0 – 16.6)	2.04 (1.38 – 2.99)	2.11 (1.42 – 3.19)	-13.6 (-19 – -7.5)	-14.7 (-20.7 – -8.1)	-31.5 (-44.1 – -17.4)	-11.2 (-19.8 – -5.8)
The Gambia	70.1 (34.0 – 108.6)	36.7 (17.8 – 65.3)	1.62 (1.26 – 2.08)	1.87 (1.44 – 2.43)	-7.8 (-11.5 – -4.0)	-11.2 (-15 – -7.2)	-81.3 (-123.3 – -41.9)	-45.5 (-79.5 – -23.7)
Timor-Leste	67.7 (18.2 – 114.5)	28.8 (13.1 – 50.8)	1.57 (1.13 – 2.17)	1.90 (1.38 – 2.59)	-7.4 (-11.8 – -2.6)	-11.9 (-16.5 – -7.2)	-80.5 (-130.5 – -28.9)	-37.0 (-62.0 – -19.1)
Togo	84.8 (60.5 – 109.1)	58.1 (40.6 – 79.2)	1.99 (1.64 – 2.42)	2.42 (1.95 – 3.02)	-11.1 (-13.8 – -8.2)	-14.9 (-18.2 – -11.8)	-100.2 (-126.9 – -74.0)	-70.6 (-94.4 – -50.7)
Tunisia	36.4 (15.9 – 56.4)	10.1 (4.4 – 16.8)	1.98 (1.34 – 2.91)	2.13 (1.42 – 3.23)	-12.7 (-18.2 – -6.8)	-14.6 (-20.6 – -8.1)	-45.1 (-65.8 – -23.8)	-12.4 (-19.9 – -6.4)
Turkey‡	70.0 (53.0 – 87.0)	12.8 (8.7 – 16.8)	2.78 (2.13 – 3.65)	2.87 (2.03 – 4.07)	-17.4 (-21 – -13.6)	-19.0 (-24.2 – -13.5)	-80.6 (-98.7 – -62.5)	-15.1 (-19.3 – -10.7)
Turkmenistan	50.0 (21.2 – 80.6)	34.7 (11.3 – 79.5)	1.84 (1.29 – 2.72)	2.02 (1.40 – 2.96)	-11.3 (-16.7 – -5.7)	-13.4 (-18.9 – -7.8)	-61.0 (-92.7 – -30.6)	-42.8 (-96.8 – -15.4)

Continued on next page

*: Difference in 1st and 5th wealth quintile-specific U5MR.
†: Ratio of 1st to 5th wealth quintile-specific U5MR.

Table 10 – continued from previous page

	Difference* (deaths per 1000 livebirths)		Ratio†		Concentration index (× 100)		Slope inequality index (deaths per 1000 livebirths)	
	1990	2016	1990	2016	1990	2016	1990	2016
Uganda	62.8 (36.4 – 89.6)	28.8 (16.8 – 42.0)	1.47 (1.25 – 1.73)	1.74 (1.39 – 2.19)	-6.0 (-8.7 – -3.3)	-10.7 (-14.5 – -6.9)	-65.5 (-95.6 – -35.8)	-35.4 (-50.2 – -22.2)
Ukraine	13.2 (5.7 – 20.8)	6.3 (2.8 – 9.8)	2.01 (1.35 – 3.03)	2.04 (1.35 – 3.09)	-13.5 (-19.5 – -7.3)	-13.9 (-20.2 – -7.5)	-16.4 (-24.4 – -8.9)	-7.9 (-11.5 – -4.3)
United Republic of Tanzania¶	29.9 (2.2 – 57.5)	15.5 (4.3 – 27.6)	1.21 (1.01 – 1.44)	1.33 (1.08 – 1.61)	-3.8 (-6.5 – -1.0)	-6.3 (-9.5 – -2.9)	-42.8 (-73.1 – -11.6)	-22.2 (-35.9 – -9.9)
Uzbekistan	21.1 (3.3 – 38.7)	10.3 (2.8 – 18.2)	1.36 (1.05 – 1.74)	1.54 (1.13 – 2.11)	-6.9 (-10.8 – -2.7)	-9.4 (-14.5 – -4.3)	-31.2 (-49.9 – -12.2)	-14.2 (-22.7 – -6.2)
Vanuatu	23.8 (11.0 – 36.7)	18.6 (7.9 – 33.8)	2.04 (1.38 – 3.03)	2.03 (1.37 – 3.06)	-13.3 (-18.8 – -7.3)	-13.3 (-19.1 – -7.4)	-29.5 (-43.3 – -16.0)	-22.9 (-39.8 – -11.3)
Vietnam	42.0 (27.7 – 56.4)	18.6 (11.4 – 26.5)	2.38 (1.76 – 3.25)	2.44 (1.74 – 3.42)	-15.8 (-20.2 – -11.2)	-16.5 (-21.5 – -11.1)	-50.4 (-65.3 – -35.5)	-22.2 (-30.8 – -14.5)
Yemen	83.6 (48.1 – 119.1)	43.9 (25.2 – 67.9)	2.09 (1.51 – 2.91)	2.36 (1.70 – 3.28)	-12.3 (-16.9 – -7.5)	-15.2 (-19.9 – -10.4)	-96.3 (-133.5 – -58.8)	-52.4 (-79.1 – -31.5)
Zambia	56.8 (32.5 – 81.8)	28.3 (15.0 – 45.8)	1.41 (1.22 – 1.64)	1.58 (1.30 – 1.94)	-5.5 (-7.9 – -3.1)	-9.1 (-12.5 – -5.8)	-62.7 (-90.7 – -35.2)	-36.2 (-56.6 – -20.8)
Zimbabwe	22.6 (8.8 – 36.4)	26.0 (14.5 – 40.3)	1.38 (1.14 – 1.67)	1.62 (1.33 – 2.01)	-6.0 (-9.2 – -2.9)	-9.4 (-12.8 – -6.2)	-28.4 (-43.4 – -13.5)	-33.3 (-49.3 – -20.3)

*: Difference in 1st and 5th wealth quintile-specific U5MR.

†: Ratio of 1st to 5th wealth quintile-specific U5MR.

Table 11: **Overview of data series by country.** For each country, the total number of observation and the most recent observation year are shown after the country name. For each country-specific data series, the number of observations and the most recent observation year within that series are shown before each data series name. Countries are ordered alphabetically.

Country	ISO code	# obs.	Most recent ref. year	Series Name (Survey Year)	Data collection method
Afghanistan	AFG	3	2012.5		
		1	2007.5	Demographic and Health Survey (2010)	Full birth histories
		1	2006.6	Multiple Indicator Cluster Survey (2010–2011)	Summary birth histories
		1	2012.5	Demographic and Health Survey (2015)	Full birth histories
Albania	ALB	3	2005.5		
		1	1995.3	Multiple Indicator Cluster Survey (2000)	Summary birth histories
		1	2000.9	Multiple Indicator Cluster Survey (2005)	Summary birth histories
		1	2005.5	Demographic and Health Survey (2008–2009)	Full birth histories
Algeria	DZA	1	2009.5		
		1	2009.5	Multiple Indicator Cluster Survey (2012–2013)	Full birth histories
Angola	AGO	3	2012.5		
		1	1997	Multiple Indicator Cluster Survey (2001)	Summary birth histories
		1	2008.5	Demographic and Health Survey (2011)	Full birth histories
		1	2012.5	Demographic and Health Survey (2015–2016)	Full birth histories
Armenia	ARM	3	2007.5		
		1	1997.5	Demographic and Health Survey (2000)	Full birth histories
		1	2002.5	Demographic and Health Survey (2005)	Full birth histories
		1	2007.5	Demographic and Health Survey (2010)	Full birth histories
Azerbaijan	AZE	2	2003.5		
		1	1995.6	Multiple Indicator Cluster Survey (2000)	Summary birth histories
		1	2003.5	Demographic and Health Survey (2006)	Full birth histories
Bangladesh	BGD	7	2011.5		
		1	1990.5	Demographic and Health Survey (1993–1994)	Full birth histories
		1	1993.5	Demographic and Health Survey (1996–1997)	Full birth histories
		1	1996.5	Demographic and Health Survey (1999–2000)	Full birth histories
		1	2001.5	Demographic and Health Survey (2004)	Full birth histories
		1	2004.5	Demographic and Health Survey (2007)	Full birth histories
		1	2008.5	Demographic and Health Survey (2011)	Full birth histories
		1	2011.5	Demographic and Health Survey (2014)	Full birth histories
Belarus	BLR	1	2000.5		
		1	2000.5	Multiple Indicator Cluster Survey (2005)	Summary birth histories
Belize	BLZ	1	2006.8		
		1	2006.8	Multiple Indicator Cluster Survey (2011)	Summary birth histories

Continued on next page

obs.: the short form of “number of observations”
ref. year: the short form of “reference year”. Reference year of data from full birth history represents the mid-point of a five-year interval.

Table 11 – continued from previous page

Country	ISO code	# obs.	Most recent ref. year	Series Name (Survey Year)	Data collection method
Benin	BEN	4	2008.5		
		1	1993.5	Demographic and Health Survey (1996)	Full birth histories
		1	1998.5	Demographic and Health Survey (2001)	Full birth histories
		1	2003.5	Demographic and Health Survey (2006)	Full birth histories
		1	2008.5	Demographic and Health Survey (2011–2012)	Full birth histories
Bhutan	BTN	1	2005.7		
		1	2005.7	Multiple Indicator Cluster Survey (2010)	Summary birth histories
Bolivia (Plurinational State of)	BOL	5	2005.5		
		1	1990.5	Demographic and Health Survey (1994)	Full birth histories
		1	1995.5	Demographic and Health Survey (1998)	Full birth histories
		1	1996.1	Multiple Indicator Cluster Survey (2000)	Summary birth histories
		1	2000.5	Demographic and Health Survey (2003)	Full birth histories
		1	2005.5	Demographic and Health Survey (2008)	Full birth histories
Brazil	BRA	1	1993.5		
		1	1993.5	Demographic and Health Survey (1996)	Full birth histories
Burkina Faso	BFA	5	2007.5		
		1	1989.5	Demographic and Health Survey (1993)	Full birth histories
		1	1995.5	Demographic and Health Survey (1998–1999)	Full birth histories
		1	2000.5	Demographic and Health Survey (2003)	Full birth histories
		1	2002	Multiple Indicator Cluster Survey (2006)	Summary birth histories
		1	2007.5	Demographic and Health Survey (2010)	Full birth histories
Burundi	BDI	3	2007.5		
		1	1995.7	Multiple Indicator Cluster Survey (2000)	Summary birth histories
		1	2001.5	Multiple Indicator Cluster Survey (2005)	Summary birth histories
		1	2007.5	Demographic and Health Survey (2010)	Full birth histories
Cambodia	KHM	3	2007.5		
		1	1997.5	Demographic and Health Survey (2000)	Full birth histories
		1	2002.5	Demographic and Health Survey (2005)	Full birth histories
		1	2007.5	Demographic and Health Survey (2010)	Full birth histories
Cameroon	CMR	5	2008.5		
		1	1988.5	Demographic and Health Survey (1991)	Full birth histories
		1	1995.5	Demographic and Health Survey (1998)	Full birth histories
		1	1996.2	Multiple Indicator Cluster Survey (2000)	Summary birth histories
		1	2001.5	Demographic and Health Survey (2004)	Full birth histories
		1	2008.5	Demographic and Health Survey (2011)	Full birth histories
Central African Republic	CAF	4	2006.4		

Continued on next page

obs.: the short form of “number of observations”
ref. year: the short form of “reference year”. Reference year of data from full birth history represents the mid-point of a five-year interval.

Table 11 – continued from previous page

Country	ISO code	# obs.	Most recent ref. year	Series Name (Survey Year)	Data collection method
		1	1991.5	Demographic and Health Survey (1994–1995)	Full birth histories
		1	1996.4	Multiple Indicator Cluster Survey (2000)	Summary birth histories
		1	2002.3	Multiple Indicator Cluster Survey (2006)	Summary birth histories
		1	2006.4	Multiple Indicator Cluster Survey (2010)	Summary birth histories
Chad	TCD	5	2011.5		
		1	1993.5	Demographic and Health Survey (1996–1997)	Full birth histories
		1	1996.1	Multiple Indicator Cluster Survey (2000)	Summary birth histories
		1	2001.5	Demographic and Health Survey (2004)	Full birth histories
		1	2005.8	Multiple Indicator Cluster Survey (2010)	Summary birth histories
		1	2011.5	Demographic and Health Survey (2014–2015)	Full birth histories
Colombia	COL	6	2012.5		
		1	1987.5	Demographic and Health Survey (1990)	Full birth histories
		1	1992.5	Demographic and Health Survey (1995)	Full birth histories
		1	1997.5	Demographic and Health Survey (2000)	Full birth histories
		1	2001.5	Demographic and Health Survey (2005)	Full birth histories
		1	2006.5	Demographic and Health Survey (2010)	Full birth histories
		1	2012.5	Demographic and Health Survey (2015)	Full birth histories
Comoros	COM	3	2009.5		
		1	1993.5	Demographic and Health Survey (1996)	Full birth histories
		1	1996.2	Multiple Indicator Cluster Survey (2000)	Summary birth histories
		1	2009.5	Demographic and Health Survey (2012)	Full birth histories
Congo	COG	2	2008.5		
		1	2002.5	Demographic and Health Survey (2005)	Full birth histories
		1	2008.5	Demographic and Health Survey (2011–2012)	Full birth histories
Democratic Republic of the Congo	COD	4	2010.5		
		1	1997.2	Multiple Indicator Cluster Survey (2001)	Summary birth histories
		1	2004.5	Demographic and Health Survey (2007)	Full birth histories
		1	2006	Multiple Indicator Cluster Survey (2010)	Summary birth histories
		1	2010.5	Demographic and Health Survey (2013–2014)	Full birth histories
Cote d'Ivoire	CIV	4	2008.5		
		1	1991.5	Demographic and Health Survey (1994)	Full birth histories
		1	1995.5	Demographic and Health Survey (1998–1999)	Full birth histories
		1	2002.5	Demographic and Health Survey (2005)	Full birth histories
		1	2008.5	Demographic and Health Survey (2011–2012)	Full birth histories
Dominican Republic	DOM	7	2011.5		
		1	1993.5	Demographic and Health Survey (1996)	Full birth histories

Continued on next page

obs.: the short form of "number of observations"
ref. year: the short form of "reference year". Reference year of data from full birth history represents the mid-point of a five-year interval.

Table 11 – continued from previous page

Country	ISO code	# obs.	Most recent ref. year	Series Name (Survey Year)	Data collection method
		1	1996.5	Demographic and Health Survey (1999)	Full birth histories
		1	1996.2	Multiple Indicator Cluster Survey (2000)	Summary birth histories
		1	1999.5	Demographic and Health Survey (2002)	Full birth histories
		1	2004.5	Demographic and Health Survey (2007)	Full birth histories
		1	2010.5	Demographic and Health Survey (2013)	Full birth histories
		1	2011.5	Multiple Indicator Cluster Survey (2014)	Full birth histories
Egypt	EGY	6	2011.5		
		1	1992.5	Demographic and Health Survey (1995)	Full birth histories
		1	1997.5	Demographic and Health Survey (2000)	Full birth histories
		1	2000.5	Demographic and Health Survey (2003)	Full birth histories
		1	2002.5	Demographic and Health Survey (2005)	Full birth histories
		1	2005.5	Demographic and Health Survey (2008)	Full birth histories
		1	2011.5	Demographic and Health Survey (2014)	Full birth histories
El Salvador	SLV	1	2011.5		
		1	2011.5	Multiple Indicator Cluster Survey (2014)	Full birth histories
Equatorial Guinea	GNQ	1	1996.2		
		1	1996.2	Multiple Indicator Cluster Survey (2000)	Summary birth histories
Eritrea	ERI	1	1999.5		
		1	1999.5	Demographic and Health Survey (2002)	Full birth histories
Ethiopia	ETH	3	2007.5		
		1	1997.5	Demographic and Health Survey (2000)	Full birth histories
		1	2002.5	Demographic and Health Survey (2005)	Full birth histories
		1	2007.5	Demographic and Health Survey (2011)	Full birth histories
Gabon	GAB	2	2009.5		
		1	1997.5	Demographic and Health Survey (2000)	Full birth histories
		1	2009.5	Demographic and Health Survey (2012)	Full birth histories
Gambia	GMB	3	2010.5		
		1	1995.5	Multiple Indicator Cluster Survey (2000)	Summary birth histories
		1	2001.9	Multiple Indicator Cluster Survey (2005–2006)	Summary birth histories
		1	2010.5	Demographic and Health Survey (2013)	Full birth histories
Georgia	GEO	1	2000.7		
		1	2000.7	Multiple Indicator Cluster Survey (2005)	Summary birth histories
Ghana	GHA	7	2011.5		
		1	1990.5	Demographic and Health Survey (1993)	Full birth histories
		1	1995.5	Demographic and Health Survey (1998)	Full birth histories
		1	2000.5	Demographic and Health Survey (2003)	Full birth histories

Continued on next page

obs.: the short form of “number of observations”
ref. year: the short form of “reference year”. Reference year of data from full birth history represents the mid-point of a five-year interval.

Table 11 – continued from previous page

Country	ISO code	# obs.	Most recent ref. year	Series Name (Survey Year)	Data collection method
		1	2002	Multiple Indicator Cluster Survey (2006)	Summary birth histories
		1	2005.5	Demographic and Health Survey (2008)	Full birth histories
		1	2008.5	Multiple Indicator Cluster Survey (2011)	Full birth histories
		1	2011.5	Demographic and Health Survey (2014)	Full birth histories
Guatemala	GTM	3	2011.5		
		1	1992.5	Demographic and Health Survey (1995)	Full birth histories
		1	1995.5	Demographic and Health Survey (1998–1999)	Full birth histories
		1	2011.5	Demographic and Health Survey (2014–2015)	Full birth histories
Guinea	GIN	3	2009.5		
		1	1996.5	Demographic and Health Survey (1999)	Full birth histories
		1	2002.5	Demographic and Health Survey (2005)	Full birth histories
		1	2009.5	Demographic and Health Survey (2012)	Full birth histories
Guinea-Bissau	GNB	3	2011.5		
		1	1995.8	Multiple Indicator Cluster Survey (2000)	Summary birth histories
		1	2000.9	Multiple Indicator Cluster Survey (2006)	Summary birth histories
		1	2011.5	Multiple Indicator Cluster Survey (2014)	Full birth histories
Guyana	GUY	5	2011.5		
		1	1996	Multiple Indicator Cluster Survey (2000–2001)	Summary birth histories
		1	2002.5	Demographic and Health Survey (2005)	Full birth histories
		1	2001.6	Multiple Indicator Cluster Survey (2006)	Summary birth histories
		1	2006.5	Demographic and Health Survey (2009)	Full birth histories
		1	2011.5	Multiple Indicator Cluster Survey (2014)	Full birth histories
Haiti	HTI	4	2009.5		
		1	1991.5	Demographic and Health Survey (1994–1995)	Full birth histories
		1	1997.5	Demographic and Health Survey (2000)	Full birth histories
		1	2002.5	Demographic and Health Survey (2005–2006)	Full birth histories
		1	2009.5	Demographic and Health Survey (2012)	Full birth histories
Honduras	HND	2	2008.5		
		1	2002.5	Demographic and Health Survey (2005–2006)	Full birth histories
		1	2008.5	Demographic and Health Survey (2011–2012)	Full birth histories
India	IND	3	2002.5		
		1	1989.5	Demographic and Health Survey (1992–1993)	Full birth histories
		1	1995.5	Demographic and Health Survey (1998–1999)	Full birth histories
		1	2002.5	Demographic and Health Survey (2005–2006)	Full birth histories
Indonesia	IDN	3	2009.5		
		1	1994.5	Demographic and Health Survey (1997)	Full birth histories

Continued on next page

obs.: the short form of “number of observations”

ref. year: the short form of “reference year”. Reference year of data from full birth history represents the mid-point of a five-year interval.

Table 11 – continued from previous page

Country	ISO code	# obs.	Most recent ref. year	Series Name (Survey Year)	Data collection method
		1	1999.5	Demographic and Health Survey (2002–2003)	Full birth histories
		1	2009.5	Demographic and Health Survey (2012)	Full birth histories
Iraq	IRQ	1	2008.5		
		1	2008.5	Multiple Indicator Cluster Survey (2011)	Full birth histories
Jordan	JOR	6	2009.5		
		1	1987.5	Demographic and Health Survey (1990)	Full birth histories
		1	1994.5	Demographic and Health Survey (1997)	Full birth histories
		1	1999.5	Demographic and Health Survey (2002)	Full birth histories
		1	2004.5	Demographic and Health Survey (2007)	Full birth histories
		1	2006.5	Demographic and Health Survey (2009)	Full birth histories
		1	2009.5	Demographic and Health Survey (2012)	Full birth histories
Kazakhstan	KAZ	4	2005.9		
		1	1992.5	Demographic and Health Survey (1995)	Full birth histories
		1	1996.5	Demographic and Health Survey (1999)	Full birth histories
		1	2000.9	Multiple Indicator Cluster Survey (2006)	Summary birth histories
		1	2005.9	Multiple Indicator Cluster Survey (2010–2011)	Summary birth histories
Kenya	KEN	6	2011.5		
		1	1990.5	Demographic and Health Survey (1993)	Full birth histories
		1	1995.5	Demographic and Health Survey (1998)	Full birth histories
		1	1996	Multiple Indicator Cluster Survey (2000)	Summary birth histories
		1	2000.5	Demographic and Health Survey (2003)	Full birth histories
		1	2005.5	Demographic and Health Survey (2008–2009)	Full birth histories
		1	2011.5	Demographic and Health Survey (2014)	Full birth histories
Kyrgyzstan	KGZ	4	2011.5		
		1	1994.5	Demographic and Health Survey (1997)	Full birth histories
		1	2001.2	Multiple Indicator Cluster Survey (2005–2006)	Summary birth histories
		1	2009.5	Demographic and Health Survey (2012)	Full birth histories
		1	2011.5	Multiple Indicator Cluster Survey (2014)	Full birth histories
Lao People's Democratic Republic	LAO	1	2008.5		
		1	2008.5	Multiple Indicator Cluster Survey (2011–2012)	Full birth histories
Lesotho	LSO	4	2011.5		
		1	1995.6	Multiple Indicator Cluster Survey (2000)	Summary birth histories
		1	2001.5	Demographic and Health Survey (2004)	Full birth histories
		1	2006.5	Demographic and Health Survey (2009)	Full birth histories
		1	2011.5	Demographic and Health Survey (2014)	Full birth histories
Liberia	LBR	3	2010.5		

Continued on next page

obs.: the short form of "number of observations"
ref. year: the short form of "reference year". Reference year of data from full birth history represents the mid-point of a five-year interval.

Table 11 – continued from previous page

**Country**	**ISO code**	**# obs.**	**Most recent ref. year**	**Series Name (Survey Year)**	**Data collection method**
		1	2003.5	Demographic and Health Survey (2007)	Full birth histories
		1	2005.5	Demographic and Health Survey (2009)	Full birth histories
		1	2010.5	Demographic and Health Survey (2013)	Full birth histories
The former Yugoslav Republic of Macedonia	MKD	1	2000.5		
		1	2000.5	Multiple Indicator Cluster Survey (2005)	Summary birth histories
Madagascar	MDG	4	2005.5		
		1	1994.5	Demographic and Health Survey (1997)	Full birth histories
		1	1996.2	Multiple Indicator Cluster Survey (2000)	Summary birth histories
		1	2000.5	Demographic and Health Survey (2003–2004)	Full birth histories
		1	2005.5	Demographic and Health Survey (2008–2009)	Full birth histories
Malawi	MWI	6	2012.5		
		1	1989.5	Demographic and Health Survey (1992)	Full birth histories
		1	1997.5	Demographic and Health Survey (2000)	Full birth histories
		1	2001.5	Demographic and Health Survey (2004)	Full birth histories
		1	2003.5	Multiple Indicator Cluster Survey (2006)	Full birth histories
		1	2007.5	Demographic and Health Survey (2010)	Full birth histories
		1	2012.5	Demographic and Health Survey (2015–2016)	Full birth histories
Maldives	MDV	1	2006.5		
		1	2006.5	Demographic and Health Survey (2009)	Full birth histories
Mali	MLI	4	2009.5		
		1	1992.5	Demographic and Health Survey (1995–1996)	Full birth histories
		1	1998.5	Demographic and Health Survey (2001)	Full birth histories
		1	2003.5	Demographic and Health Survey (2006)	Full birth histories
		1	2009.5	Demographic and Health Survey (2012–2013)	Full birth histories
Mauritania	MRT	2	2008.5		
		1	2003.1	Multiple Indicator Cluster Survey (2007)	Summary birth histories
		1	2008.5	Multiple Indicator Cluster Survey (2011)	Full birth histories
Republic of Moldova	MDA	2	2009.5		
		1	2002.5	Demographic and Health Survey (2005)	Full birth histories
		1	2009.5	Multiple Indicator Cluster Survey (2012)	Full birth histories
Mongolia	MNG	4	2010.5		
		1	1996	Multiple Indicator Cluster Survey (2000)	Summary birth histories
		1	2001.2	Multiple Indicator Cluster Survey (2005)	Summary birth histories
		1	2005.8	Multiple Indicator Cluster Survey (2010)	Summary birth histories
		1	2010.5	Multiple Indicator Cluster Survey (2013–2014)	Full birth histories
Morocco	MAR	2	2000.5		

Continued on next page

obs.: the short form of “number of observations”
ref. year: the short form of “reference year”. Reference year of data from full birth history represents the mid-point of a five-year interval.

Table 11 – continued from previous page

Country	ISO code	# obs.	Most recent ref. year	Series Name (Survey Year)	Data collection method
		1	1989.5	Demographic and Health Survey (1992)	Full birth histories
		1	2000.5	Demographic and Health Survey (2003–2004)	Full birth histories
Mozambique	MOZ	4	2008.5		
		1	1994.5	Demographic and Health Survey (1997)	Full birth histories
		1	2000.5	Demographic and Health Survey (2003)	Full birth histories
		1	2005.5	Multiple Indicator Cluster Survey (2008)	Full birth histories
		1	2008.5	Demographic and Health Survey (2011)	Full birth histories
Myanmar	MMR	1	2012.5		
		1	2012.5	Demographic and Health Survey (2015–2016)	Full birth histories
Namibia	NAM	4	2010.5		
		1	1989.5	Demographic and Health Survey (1992)	Full birth histories
		1	1997.5	Demographic and Health Survey (2000)	Full birth histories
		1	2003.5	Demographic and Health Survey (2006–2007)	Full birth histories
		1	2010.5	Demographic and Health Survey (2013)	Full birth histories
Nepal	NPL	5	2011.5		
		1	1993.5	Demographic and Health Survey (1996)	Full birth histories
		1	1998.5	Demographic and Health Survey (2001)	Full birth histories
		1	2003.5	Demographic and Health Survey (2006)	Full birth histories
		1	2008.5	Demographic and Health Survey (2011)	Full birth histories
		1	2011.5	Multiple Indicator Cluster Survey (2014)	Full birth histories
Nicaragua	NIC	1	1994.5		
		1	1994.5	Demographic and Health Survey (1998)	Full birth histories
Niger	NER	4	2009.5		
		1	1995.5	Demographic and Health Survey (1998)	Full birth histories
		1	1996	Multiple Indicator Cluster Survey (2000)	Summary birth histories
		1	2003.5	Demographic and Health Survey (2006)	Full birth histories
		1	2009.5	Demographic and Health Survey (2012)	Full birth histories
Nigeria	NGA	7	2010.5		
		1	1987.5	Demographic and Health Survey (1990)	Full birth histories
		1	2000.5	Demographic and Health Survey (2003)	Full birth histories
		1	2002.5	Multiple Indicator Cluster Survey (2007)	Summary birth histories
		1	2005.5	Demographic and Health Survey (2008)	Full birth histories
		1	2007.5	Demographic and Health Survey (2010)	Full birth histories
		1	2006.6	Multiple Indicator Cluster Survey (2011)	Summary birth histories
		1	2010.5	Demographic and Health Survey (2013)	Full birth histories
Pakistan	PAK	3	2009.5		

Continued on next page

obs.: the short form of “number of observations”
ref. year: the short form of “reference year”. Reference year of data from full birth history represents the mid-point of a five-year interval.

Table 11 – continued from previous page

Country	ISO code	# obs.	Most recent ref. year	Series Name (Survey Year)	Data collection method
		1	1987.5	Demographic and Health Survey (1990–1991)	Full birth histories
		1	2003.5	Demographic and Health Survey (2006–2007)	Full birth histories
		1	2009.5	Demographic and Health Survey (2012–2013)	Full birth histories
Paraguay	PRY	1	1987.5		
		1	1987.5	Demographic and Health Survey (1990)	Full birth histories
Peru	PER	9	2011.5		
		1	1988.5	Demographic and Health Survey (1991–1992)	Full birth histories
		1	1993.5	Demographic and Health Survey (1996)	Full birth histories
		1	1997.5	Demographic and Health Survey (2000)	Full birth histories
		1	2000.5	Demographic and Health Survey (2004–2008)	Full birth histories
		1	2006.5	Demographic and Health Survey (2009)	Full birth histories
		1	2007.5	Demographic and Health Survey (2010)	Full birth histories
		1	2008.5	Demographic and Health Survey (2011)	Full birth histories
		1	2009.5	Demographic and Health Survey (2012)	Full birth histories
		1	2011.5	Demographic and Health Survey (2014)	Full birth histories
Philippines	PHL	5	2010.5		
		1	1990.5	Demographic and Health Survey (1993)	Full birth histories
		1	1995.5	Demographic and Health Survey (1998)	Full birth histories
		1	2000.5	Demographic and Health Survey (2003)	Full birth histories
		1	2005.5	Demographic and Health Survey (2008)	Full birth histories
		1	2010.5	Demographic and Health Survey (2013)	Full birth histories
Rwanda	RWA	5	2011.5		
		1	1989.5	Demographic and Health Survey (1992)	Full birth histories
		1	2002.5	Demographic and Health Survey (2005)	Full birth histories
		1	2004.5	Demographic and Health Survey (2007–2008)	Full birth histories
		1	2007.5	Demographic and Health Survey (2010)	Full birth histories
		1	2011.5	Demographic and Health Survey (2014–2015)	Full birth histories
Sao Tome and Principe	STP	3	2011.5		
		1	1996.1	Multiple Indicator Cluster Survey (2000)	Summary birth histories
		1	2005.5	Demographic and Health Survey (2008–2009)	Full birth histories
		1	2011.5	Multiple Indicator Cluster Survey (2014)	Full birth histories
Senegal	SEN	7	2012.5		
		1	1994.5	Demographic and Health Survey (1997)	Full birth histories
		1	2002.5	Demographic and Health Survey (2005)	Full birth histories
		1	2005.5	Demographic and Health Survey (2008–2009)	Full birth histories
		1	2007.5	Demographic and Health Survey (2010–2011)	Full birth histories

Continued on next page

obs.: the short form of “number of observations”
ref. year: the short form of “reference year”. Reference year of data from full birth history represents the mid-point of a five-year interval.

Table 11 – continued from previous page

Country	ISO code	# obs.	Most recent ref. year	Series Name (Survey Year)	Data collection method
		1	2009.5	Demographic and Health Survey (2012–2013)	Full birth histories
		1	2011.5	Demographic and Health Survey (2014)	Full birth histories
		1	2012.5	Demographic and Health Survey (2015)	Full birth histories
Serbia	SRB	1	2005.7		
		1	2005.7	Multiple Indicator Cluster Survey (2010)	Summary birth histories
Sierra Leone	SLE	5	2010.5		
		1	1995.8	Multiple Indicator Cluster Survey (2000)	Summary birth histories
		1	2000.9	Multiple Indicator Cluster Survey (2005–2006)	Summary birth histories
		1	2005.5	Demographic and Health Survey (2008)	Full birth histories
		1	2006.3	Multiple Indicator Cluster Survey (2010)	Summary birth histories
		1	2010.5	Demographic and Health Survey (2013)	Full birth histories
Somalia	SOM	1	2003.5		
		1	2003.5	Multiple Indicator Cluster Survey (2006)	Full birth histories
South Africa	ZAF	1	1995.5		
		1	1995.5	Demographic and Health Survey (1998)	Full birth histories
South Sudan	SSD	1	2007.5		
		1	2007.5	Multiple Indicator Cluster Survey (2010)	Full birth histories
State of Palestine	PSE	2	2011.5		
		1	2007.5	Multiple Indicator Cluster Survey (2010)	Full birth histories
		1	2011.5	Multiple Indicator Cluster Survey (2014)	Full birth histories
Sudan	SDN	2	2011.5		
		1	2007.5	Multiple Indicator Cluster Survey (2010)	Full birth histories
		1	2011.5	Multiple Indicator Cluster Survey (2014)	Full birth histories
Suriname	SUR	2	2001.5		
		1	1995.2	Multiple Indicator Cluster Survey (1999–2000)	Summary birth histories
		1	2001.5	Multiple Indicator Cluster Survey (2006)	Summary birth histories
Swaziland	SWZ	4	2011.5		
		1	1995.9	Multiple Indicator Cluster Survey (1999–2000)	Summary birth histories
		1	2003.5	Demographic and Health Survey (2006–2007)	Full birth histories
		1	2007.5	Multiple Indicator Cluster Survey (2010)	Full birth histories
		1	2011.5	Multiple Indicator Cluster Survey (2014)	Full birth histories
Syrian Arab Republic	SYR	1	2001.8		
		1	2001.8	Multiple Indicator Cluster Survey (2006)	Summary birth histories
Tajikistan	TJK	3	2009.5		
		1	1996.1	Multiple Indicator Cluster Survey (2000)	Summary birth histories
		1	2001	Multiple Indicator Cluster Survey (2005)	Summary birth histories

Continued on next page

obs.: the short form of “number of observations”

ref. year: the short form of “reference year”. Reference year of data from full birth history represents the mid-point of a five-year interval.

Table 11 – continued from previous page

Country	ISO code	# obs.	Most recent ref. year	Series Name (Survey Year)	Data collection method
		1	2009.5	Demographic and Health Survey (2012)	Full birth histories
United Republic of Tanzania	TZA	6	2012.5		
		1	1993.5	Demographic and Health Survey (1996)	Full birth histories
		1	1996.5	Demographic and Health Survey (1999)	Full birth histories
		1	2001.5	Demographic and Health Survey (2004–2005)	Full birth histories
		1	2004.5	Demographic and Health Survey (2007–2008)	Full birth histories
		1	2006.5	Demographic and Health Survey (2010)	Full birth histories
		1	2012.5	Demographic and Health Survey (2015–2016)	Full birth histories
Thailand	THA	1	2000.9		
		1	2000.9	Multiple Indicator Cluster Survey (2005–2006)	Summary birth histories
Timor-Leste	TLS	1	2006.5		
		1	2006.5	Demographic and Health Survey (2009–2010)	Full birth histories
Togo	TGO	4	2010.5		
		1	1995.5	Demographic and Health Survey (1998)	Full birth histories
		1	2001.8	Multiple Indicator Cluster Survey (2006)	Summary birth histories
		1	2006.1	Multiple Indicator Cluster Survey (2010)	Summary birth histories
		1	2010.5	Demographic and Health Survey (2013–2014)	Full birth histories
Tunisia	TUN	1	2008.5		
		1	2008.5	Multiple Indicator Cluster Survey (2011–2012)	Full birth histories
Turkey	TUR	3	2000.5		
		1	1990.5	Demographic and Health Survey (1993)	Full birth histories
		1	1995.5	Demographic and Health Survey (1998)	Full birth histories
		1	2000.5	Demographic and Health Survey (2003)	Full birth histories
Turkmenistan	TKM	1	2012.5		
		1	2012.5	Multiple Indicator Cluster Survey (2015–2016)	Full birth histories
Uganda	UGA	5	2008.5		
		1	1992.5	Demographic and Health Survey (1995)	Full birth histories
		1	1997.5	Demographic and Health Survey (2000–2001)	Full birth histories
		1	2003.5	Demographic and Health Survey (2006)	Full birth histories
		1	2006.5	Demographic and Health Survey (2009)	Full birth histories
		1	2008.5	Demographic and Health Survey (2011)	Full birth histories
Ukraine	UKR	2	2004.5		
		1	1999.9	Multiple Indicator Cluster Survey (2005)	Summary birth histories
		1	2004.5	Demographic and Health Survey (2007)	Full birth histories
Uzbekistan	UZB	3	2001.4		
		1	1993.5	Demographic and Health Survey (1996)	Full birth histories

Continued on next page

obs.: the short form of "number of observations"
ref. year: the short form of "reference year". Reference year of data from full birth history represents the mid-point of a five-year interval.

Table 11 – continued from previous page

Country	ISO code	# obs.	Most recent ref. year	Series Name (Survey Year)	Data collection method
		1	1995.9	Multiple Indicator Cluster Survey (2000)	Summary birth histories
		1	2001.4	Multiple Indicator Cluster Survey (2006)	Summary birth histories
Vanuatu	VUT	1	2003.4		
		1	2003.4	Multiple Indicator Cluster Survey (2007–2008)	Summary birth histories
Viet Nam	VNM	5	2005.7		
		1	1994.5	Demographic and Health Survey (1997)	Full birth histories
		1	1995.3	Multiple Indicator Cluster Survey (2000)	Summary birth histories
		1	1999.5	Demographic and Health Survey (2002)	Full birth histories
		1	2001.6	Multiple Indicator Cluster Survey (2006)	Summary birth histories
		1	2005.7	Multiple Indicator Cluster Survey (2010–2011)	Summary birth histories
Yemen	YEM	1	2003.5		
		1	2003.5	Multiple Indicator Cluster Survey (2006)	Full birth histories
Zambia	ZMB	4	2010.5		
		1	1993.5	Demographic and Health Survey (1996)	Full birth histories
		1	1998.5	Demographic and Health Survey (2001–2002)	Full birth histories
		1	2004.5	Demographic and Health Survey (2007)	Full birth histories
		1	2010.5	Demographic and Health Survey (2013–2014)	Full birth histories
Zimbabwe	ZWE	7	2012.5		
		1	1991.5	Demographic and Health Survey (1994)	Full birth histories
		1	1996.5	Demographic and Health Survey (1999)	Full birth histories
		1	2002.5	Demographic and Health Survey (2005–2006)	Full birth histories
		1	2006.5	Multiple Indicator Cluster Survey (2009)	Full birth histories
		1	2007.5	Demographic and Health Survey (2010–2011)	Full birth histories
		1	2011.5	Multiple Indicator Cluster Survey (2014)	Full birth histories
		1	2012.5	Demographic and Health Survey (2015)	Full birth histories

6 Supplementary Figures

- Figure 4: 3rd quintile-disparity ratios against national-level U5MR – model results;
- Figure 5: Country ranks for inequality indexes in 2016;
- Figure 6: Slope inequality index and concentration index in 2016, for the 99 countries with empirical data;
- Figure 7: Comparison between aggregated results based on the 137 LMICs, and the aggregated results based on the 99 countries with empirical data;
- Figure 8: Aggregated U5MR and percentage of under-5 deaths by wealth quintile, for the 137 LMICs combined and for regions;
- Figure 9: U5MR by wealth quintile, for the 99 countries with empirical data;
- Figure 10: Ratio of U5MR in wealth quintile 1 (poorest) to wealth quintile 5 (richest), for the 99 countries with empirical data;
- Figure 11: All wealth quintile-specific results, for the 99 countries with empirical data;

Figure 4 shows the model results of the average relative difference $U_{w,c,t}$ given the national-level U5MR for that country-year. From left to right, the four plots show the model results of $U_{w,c,t}$ for $w = 1, 2, 4, 5$ respectively. Comparing to the LOESS curves in green, within the 95% bounds of national-level U5MR, the model estimates and LOESS curves produce mostly agree with each other.

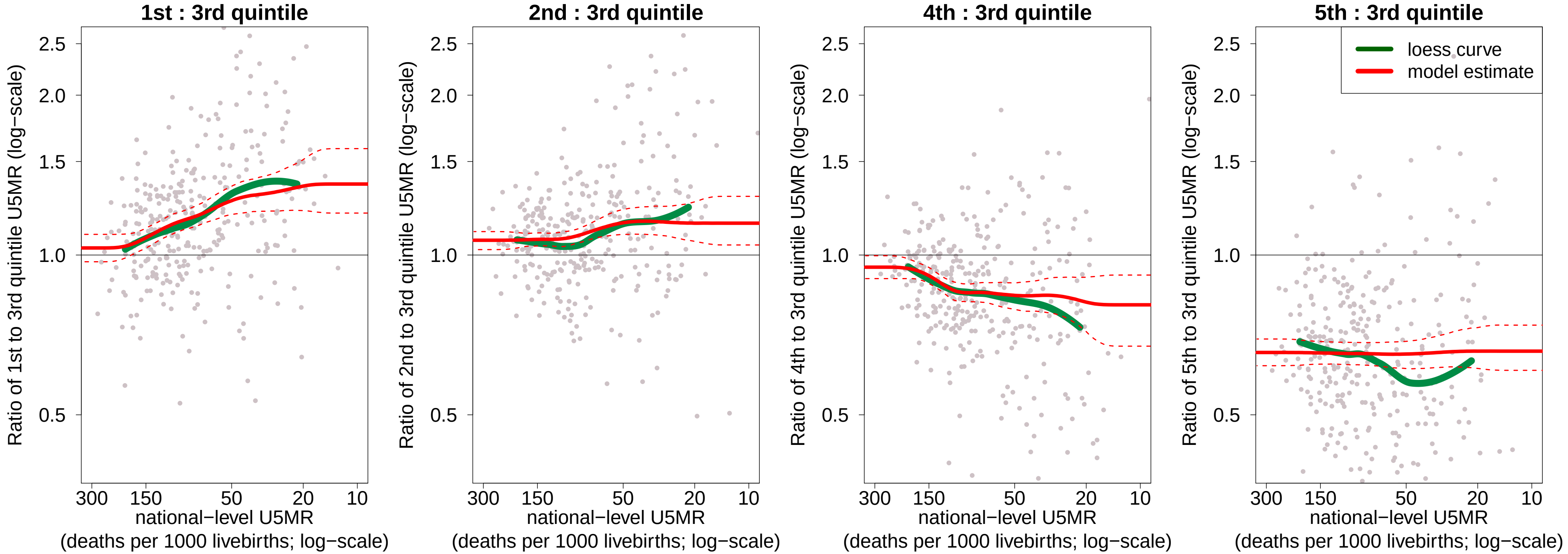

Figure 4: **3rd quintile-disparity ratios against national-level U5MR – model results.** The grey dots are observed 3rd quintile-disparity ratios $S_{w,c,t}$ (i.e. $= Q_{w,c,t}/Q_{3,c,t}$) for $w = 1, 2, 4, 5$ respectively for the four plots. The red solid curves are point estimates for the expected 3rd quintile-disparity ratios $U_{w,c,t}$ (i.e. $= S_{w,c,t}/P_{w,c,t}$) and dashed lines are the corresponding 5th and 95th percentiles of the uncertainty bounds. The green curves are LOESS curves between the 5th and 95th percentiles of the national-level U5MR.

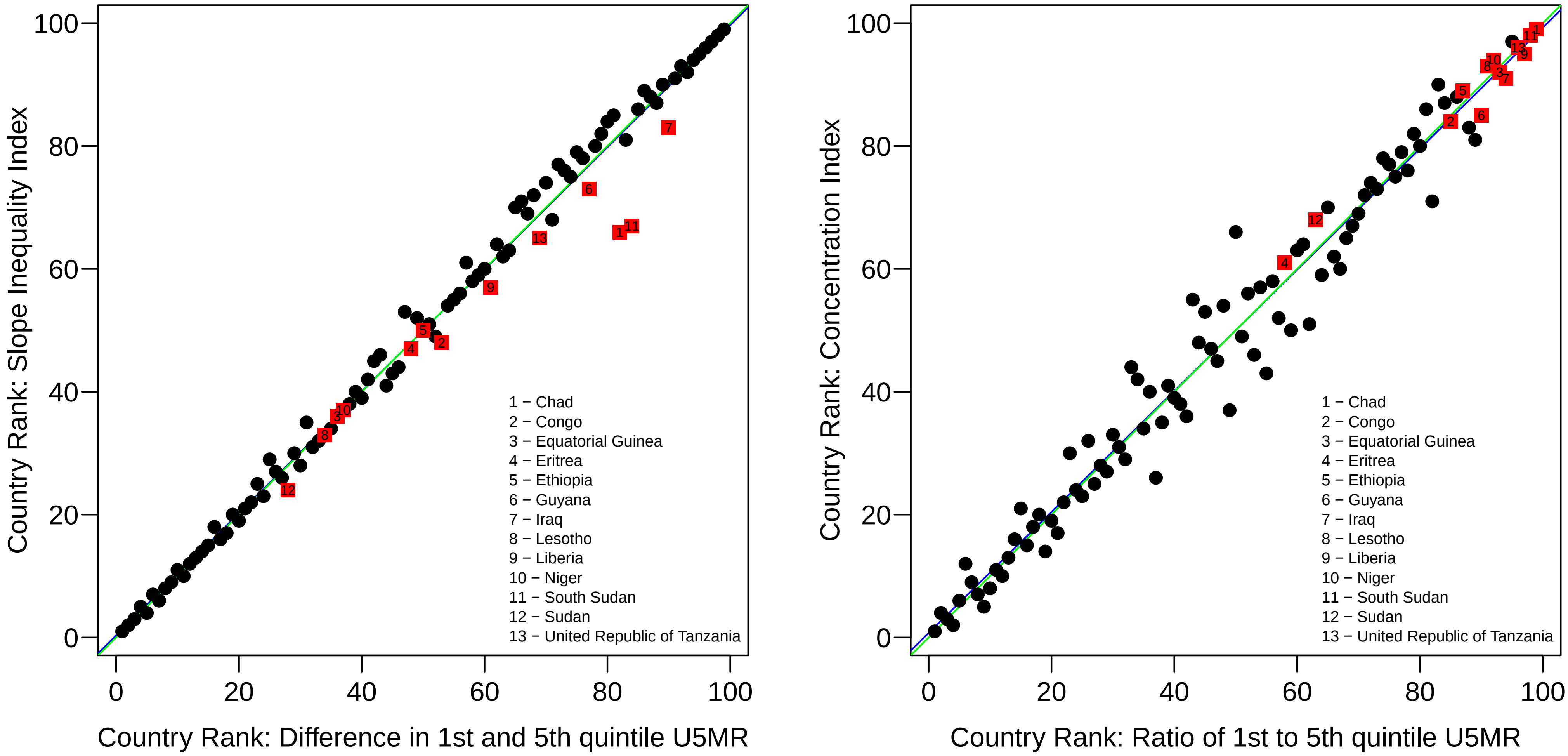

Figure 5: **Country ranks for inequality indexes in 2016, for the 99 countries with empirical data.** The dots are the country disparity ranks based on point estimates of the four inequality indexes in 2016. The smaller the ranks, the greater the disparity is. The green line is the diagonal, indicating equality of two ranks. The blue line is the fitted regression line. The red squared dots refer to countries in which the U5MR in the 1st wealth quintile is not the largest and/or the U5MR in the 5th wealth quintile is not the smallest, among all the wealth quintiles for a country in 2016.

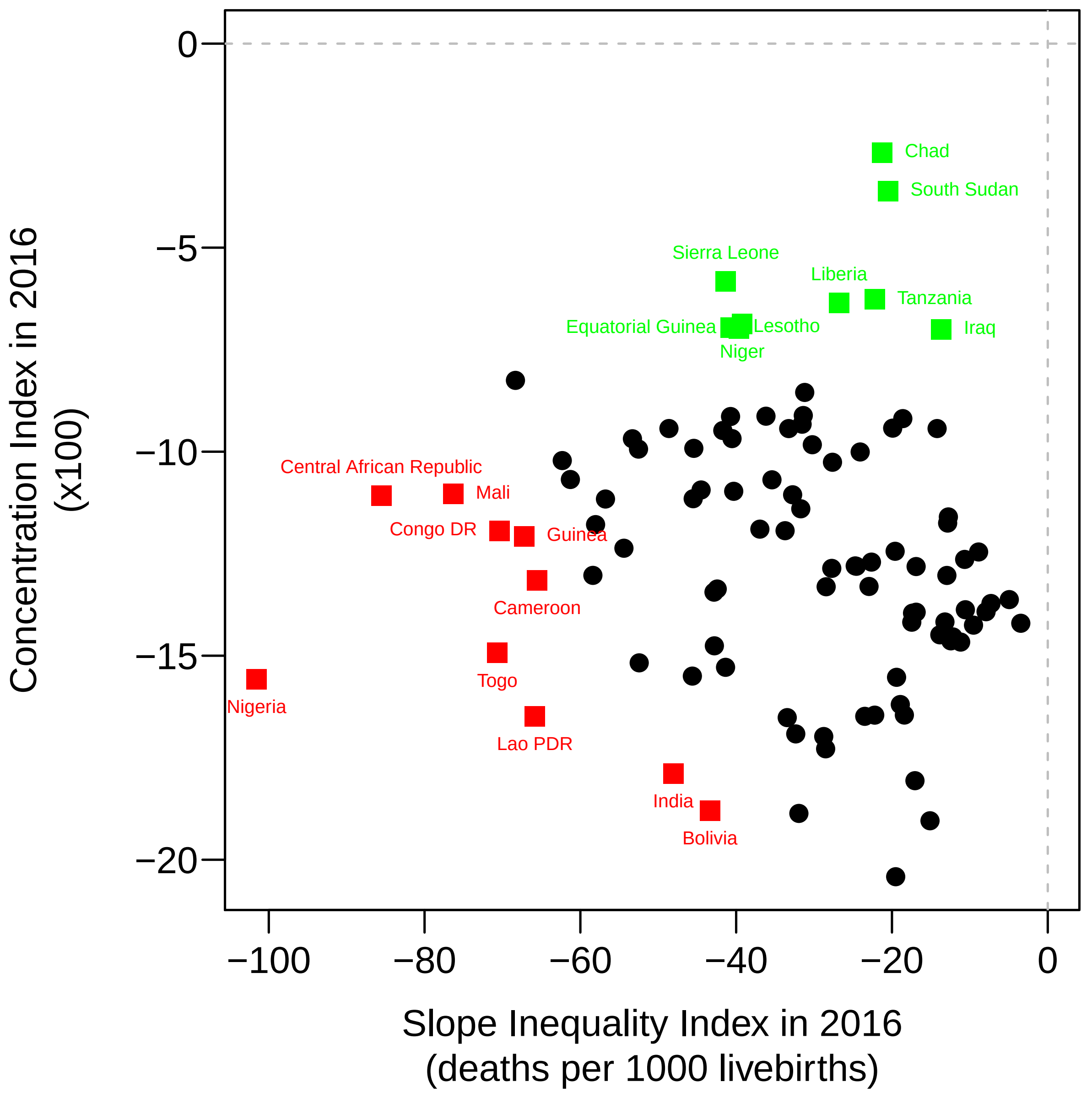

Figure 6: **Slope inequality index and concentration index in 2016, for the 99 countries with empirical data.** The dots are point estimates of indexes in 2016 for the 99 countries with empirical data. The green dots highlight countries with the smallest absolute disparity (based on slope inequality index) and the smallest relative disparity (based on concentration index). The red dots highlighted countries with the largest absolute and relative disparity.

Figure 7: **Comparison between aggregated results based on the 137 low-income and middle-income countries, and the aggregated results based on the 99 countries with empirical data.** Solid curves are point estimates from the model. Shaded areas around the solid curves are the 90% uncertainty intervals. Top: estimated ratio of wealth quintile-specific U5MR to national-level U5MR. Bottom: U5MR by wealth quintile.

All countries combined

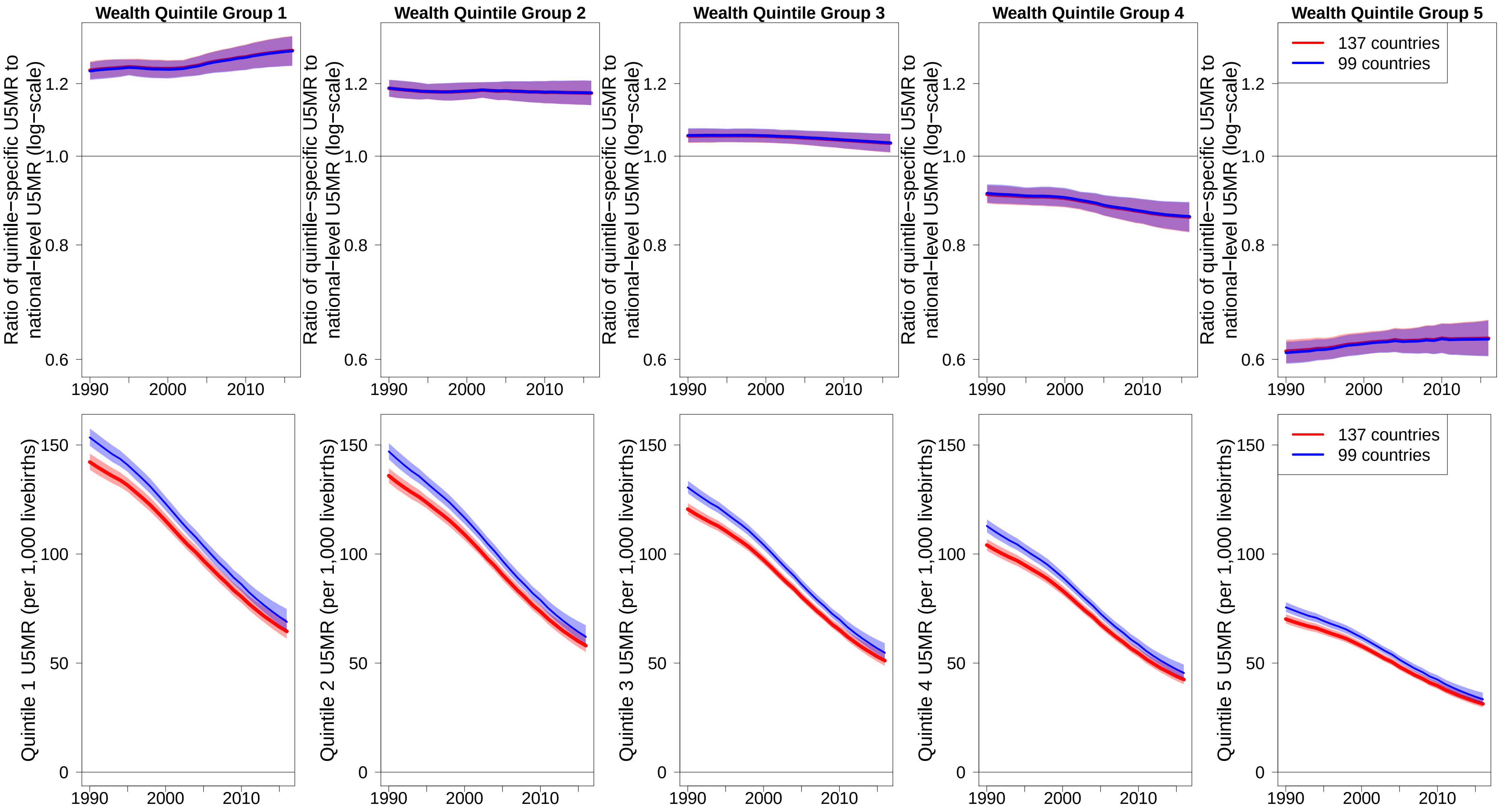

South Asia

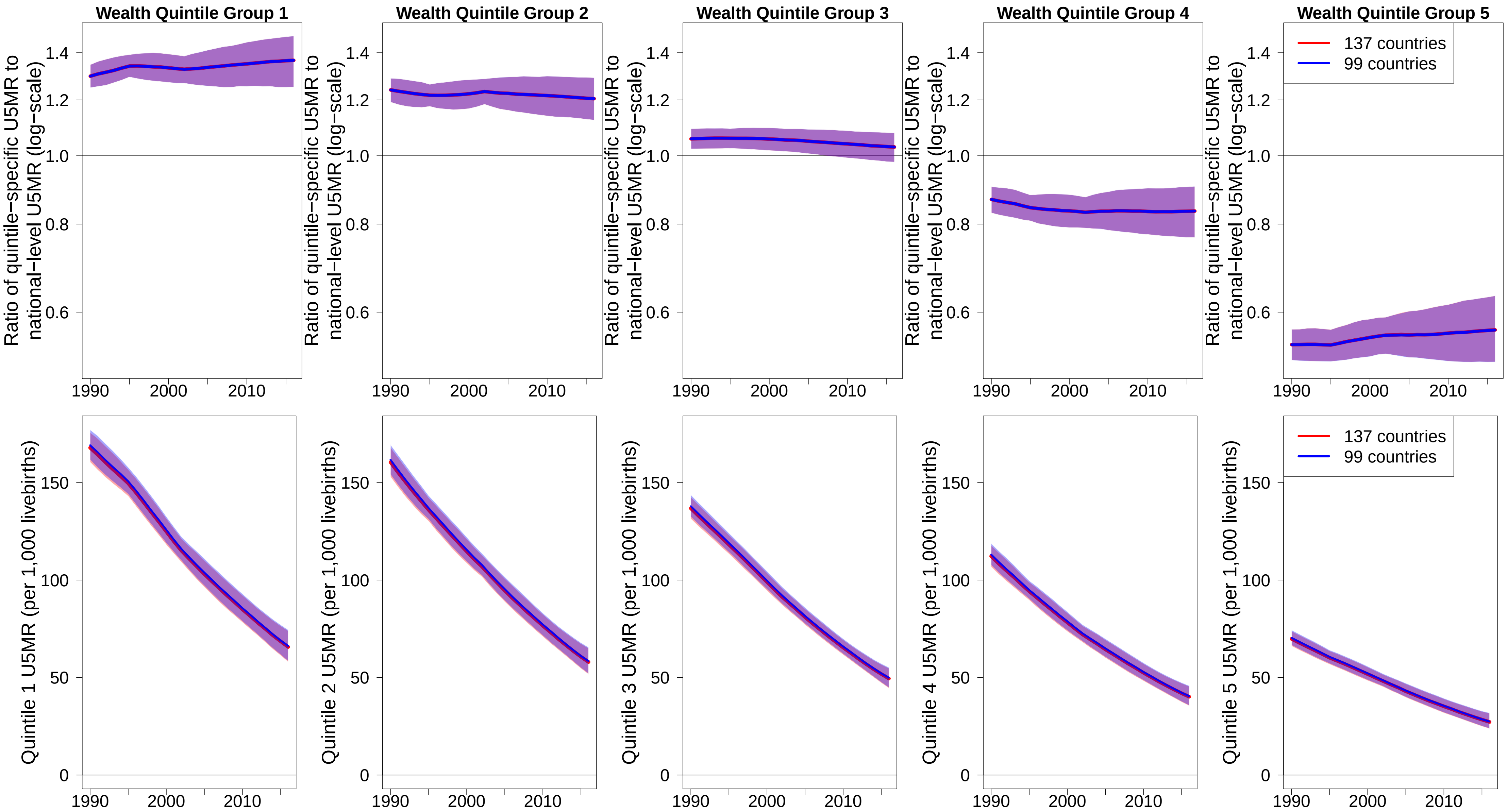

Wealth Quintile Group 1
Wealth Quintile Group 2
Wealth Quintile Group 3
Wealth Quintile Group 4
Wealth Quintile Group 5
Ratio of quintile−specific U5MR to national−level U5MR (log−scale)
137 countries
99 countries
Quintile 1 U5MR (per 1,000 livebirths)
Quintile 2 U5MR (per 1,000 livebirths)
Quintile 3 U5MR (per 1,000 livebirths)
Quintile 4 U5MR (per 1,000 livebirths)
Quintile 5 U5MR (per 1,000 livebirths)

Eastern Europe and Central Asia

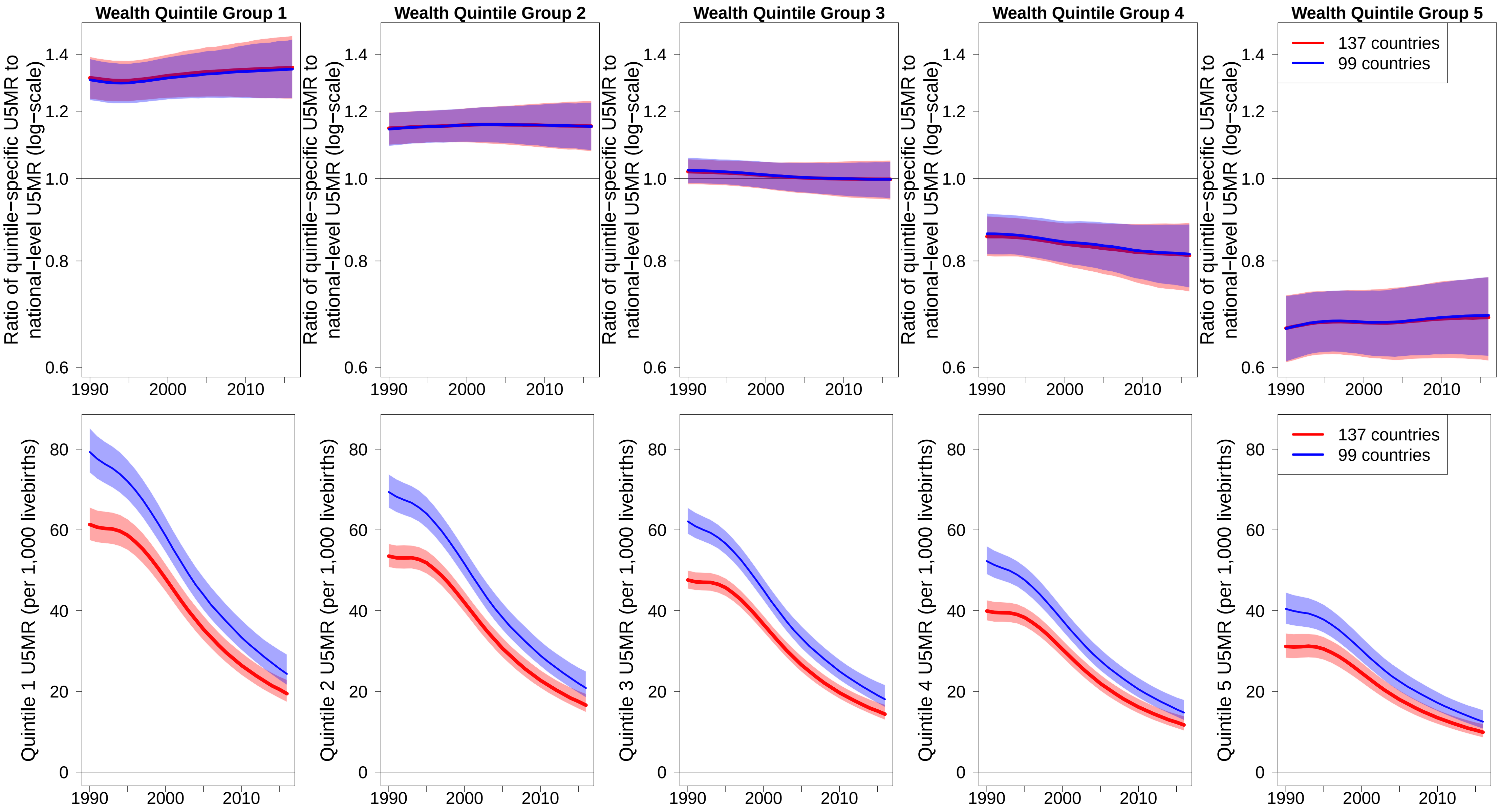

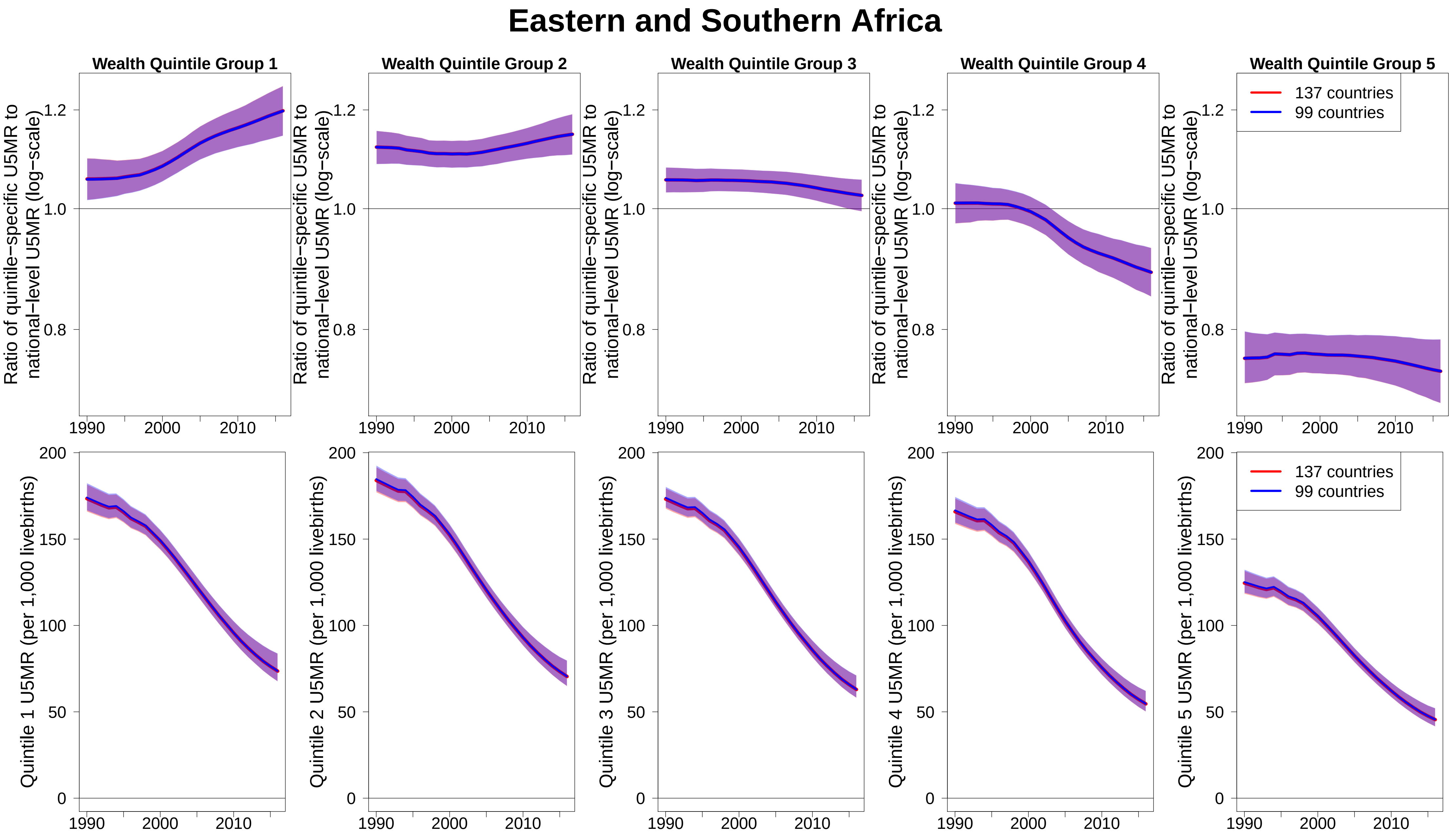
Eastern and Southern Africa
Wealth Quintile Group 1
Wealth Quintile Group 2
Wealth Quintile Group 3
Wealth Quintile Group 4
Wealth Quintile Group 5
Ratio of quintile−specific U5MR to national−level U5MR (log−scale)
137 countries
99 countries
Quintile 1 U5MR (per 1,000 livebirths)
Quintile 2 U5MR (per 1,000 livebirths)
Quintile 3 U5MR (per 1,000 livebirths)
Quintile 4 U5MR (per 1,000 livebirths)
Quintile 5 U5MR (per 1,000 livebirths)
1990
2000
2010

West and Central Africa

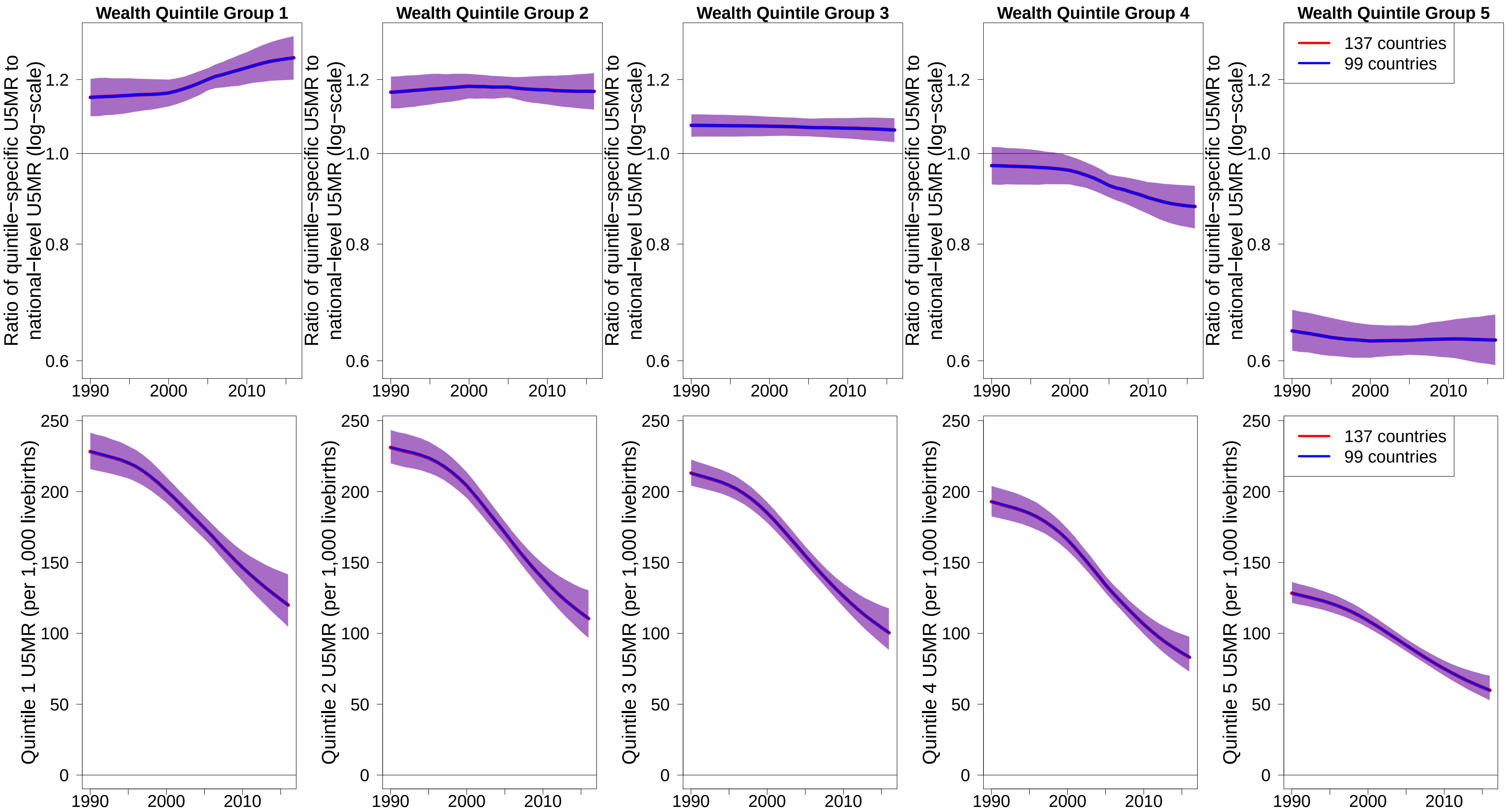

Latin America and Caribbean

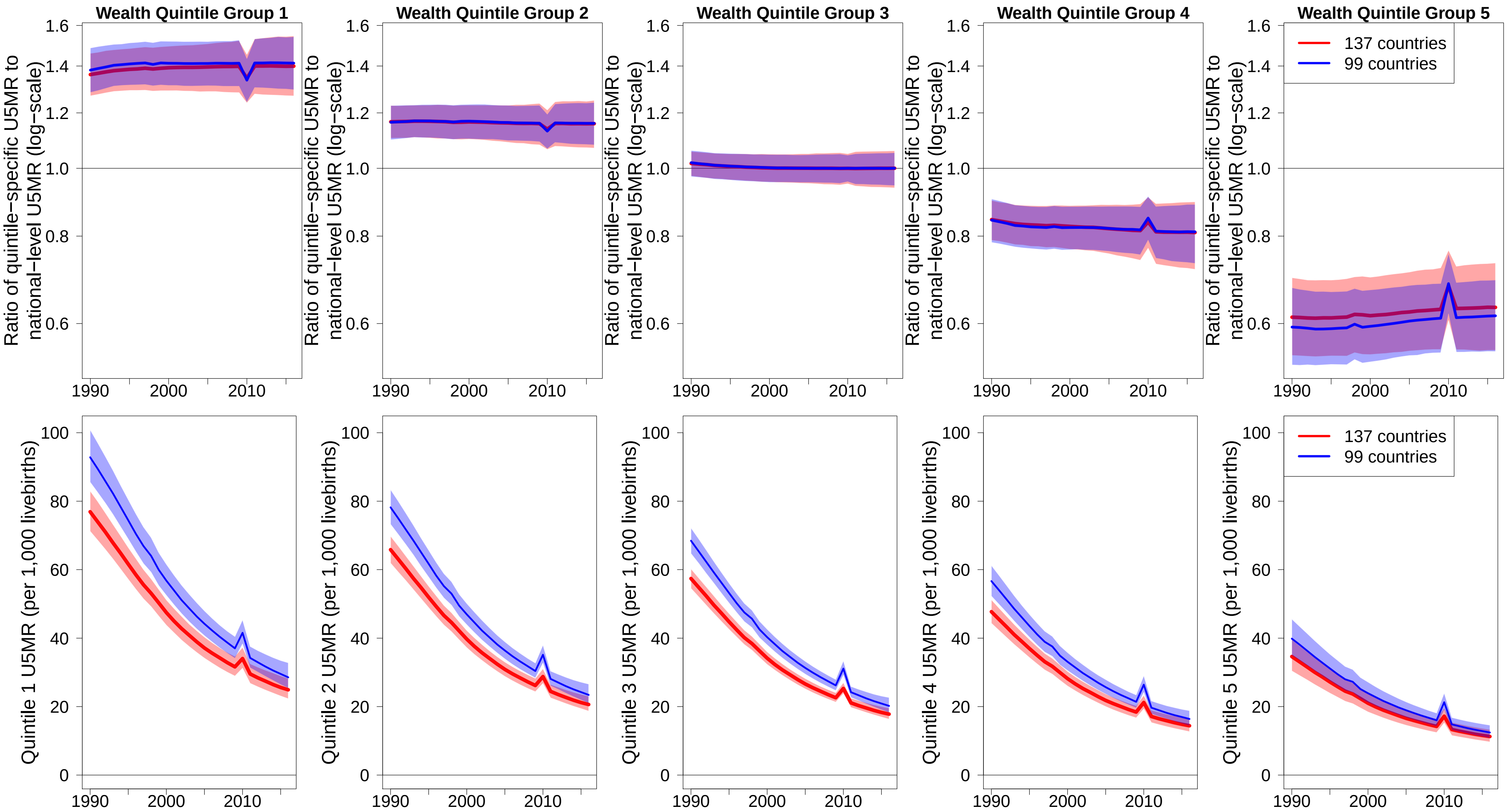

East Asia and Pacific (excluding China)

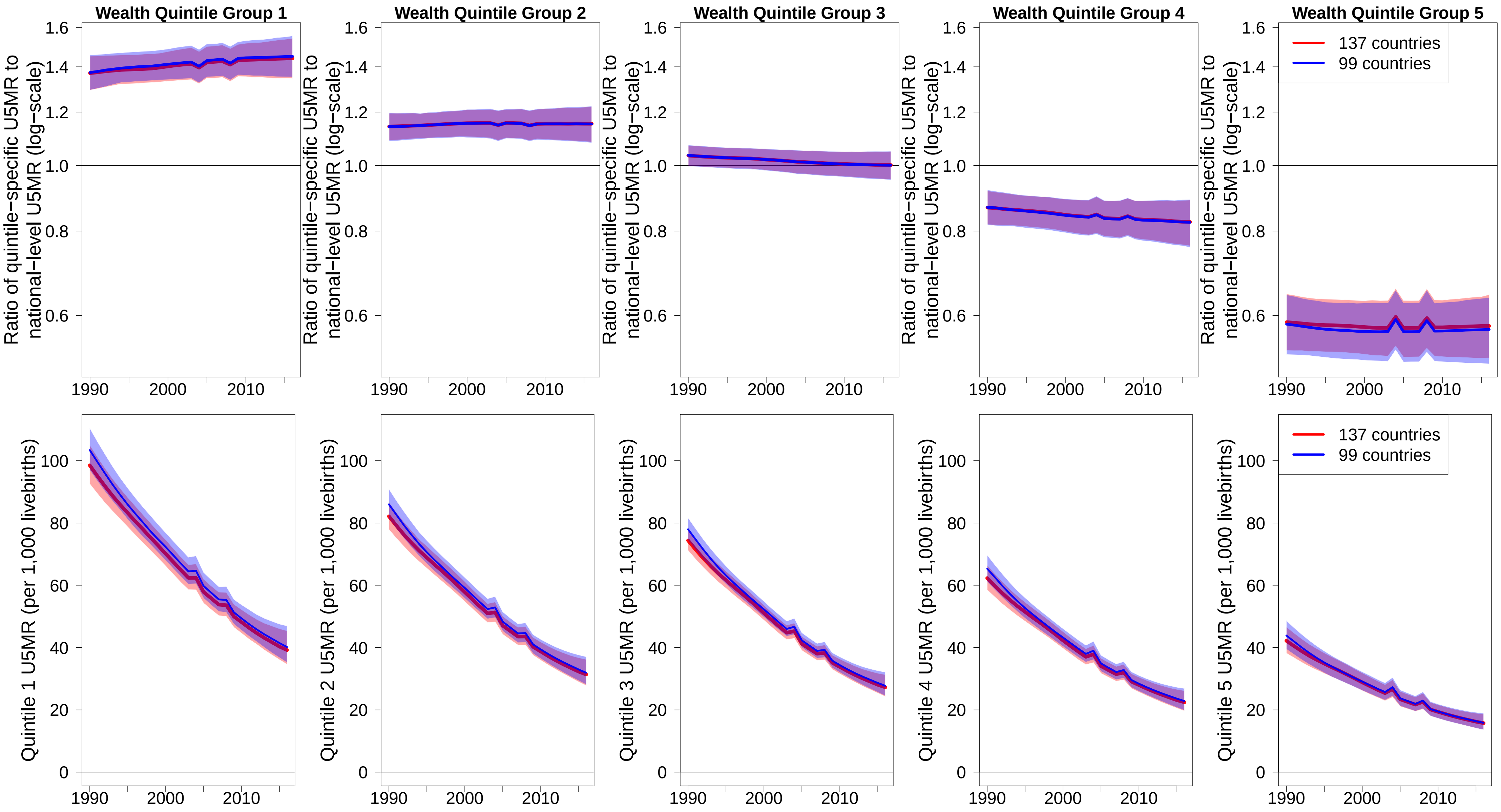

Middle East and North Africa

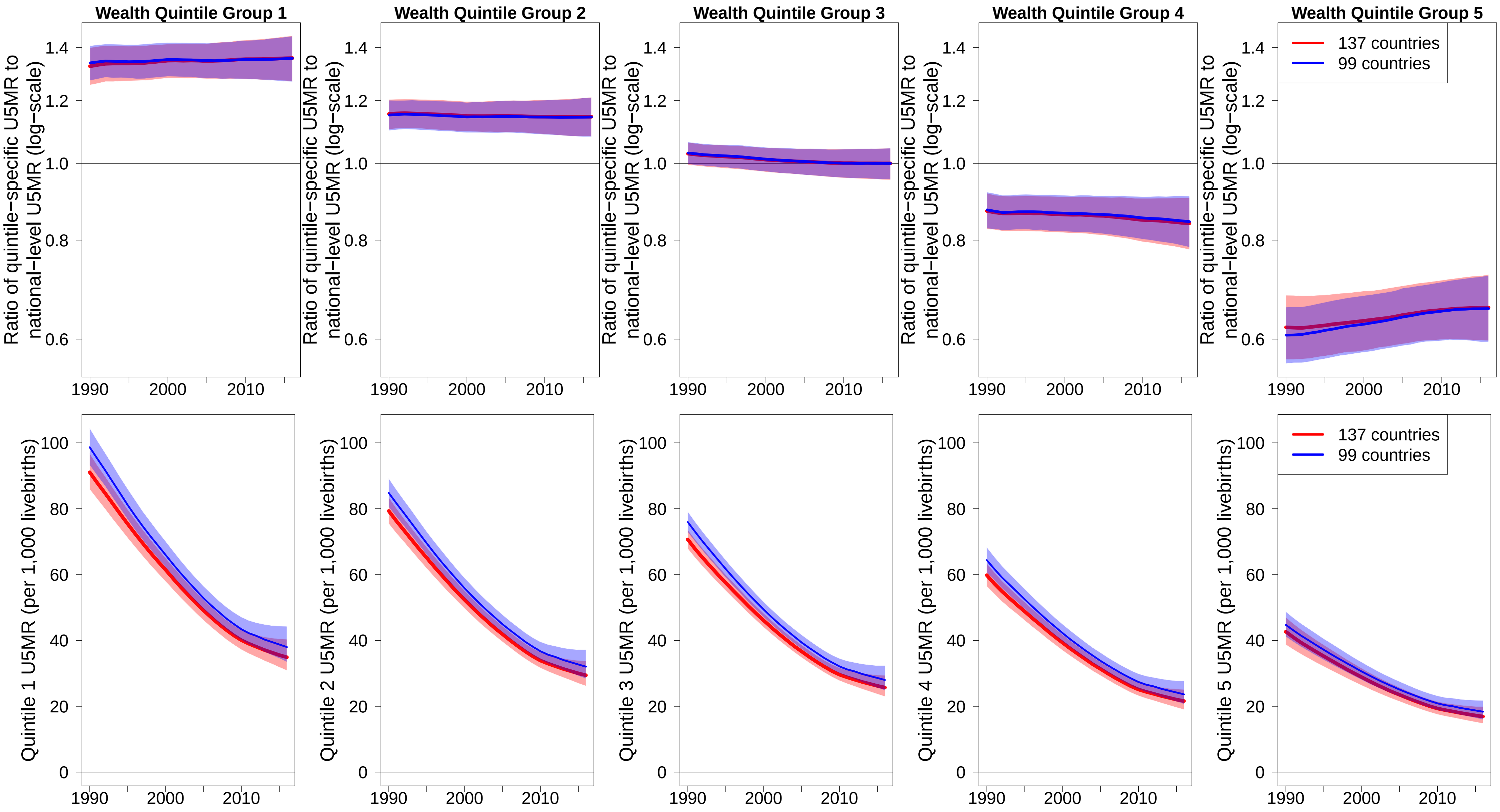

Figure 8: **Aggregated U5MR and percentage of under-5 deaths by wealth quintile, by region.** Solid curves are point estimates from the model. Shaded areas around the solid curves are the 90% uncertainty intervals. Left: U5MR by wealth quintile. Right: percentage of wealth quintile-specific under-5 deaths among national-level under-5 deaths (in %) by wealth quintile. Q1: the 1st wealth quintile, the 20% poorest wealth quintile; Q5: the 5th wealth quintile, the 20% richest wealth quintile.

South Asia

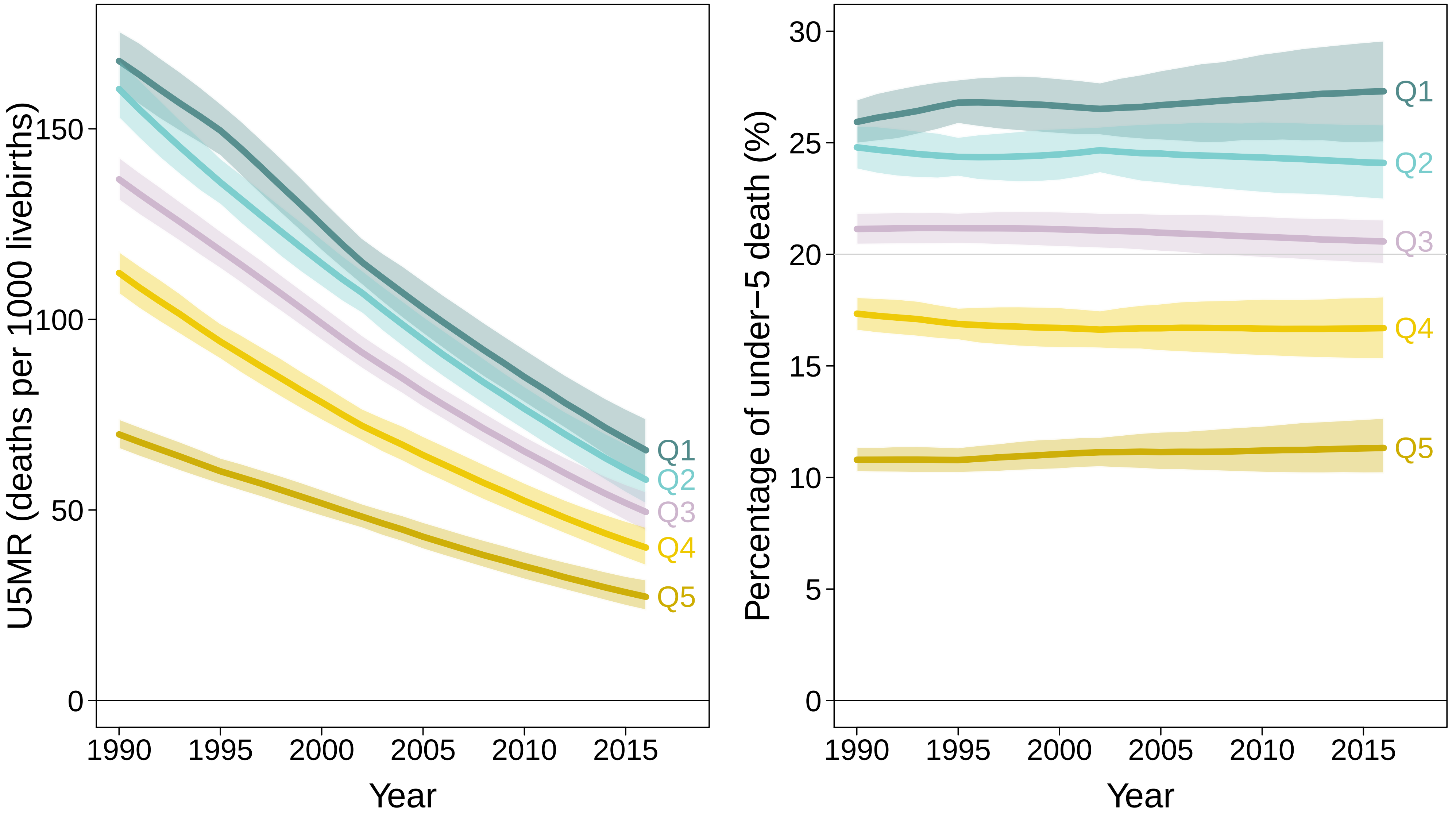

U5MR (deaths per 1000 livebirths)
150
100
50
0
1990
1995
2000
2005
2010
2015
Year
Q1
Q2
Q3
Q4
Q5
Percentage of under−5 death (%)
30
25
20
15
10
5
0
1990
1995
2000
2005
2010
2015
Year
Q1
Q2
Q3
Q4
Q5

Eastern Europe and Central Asia

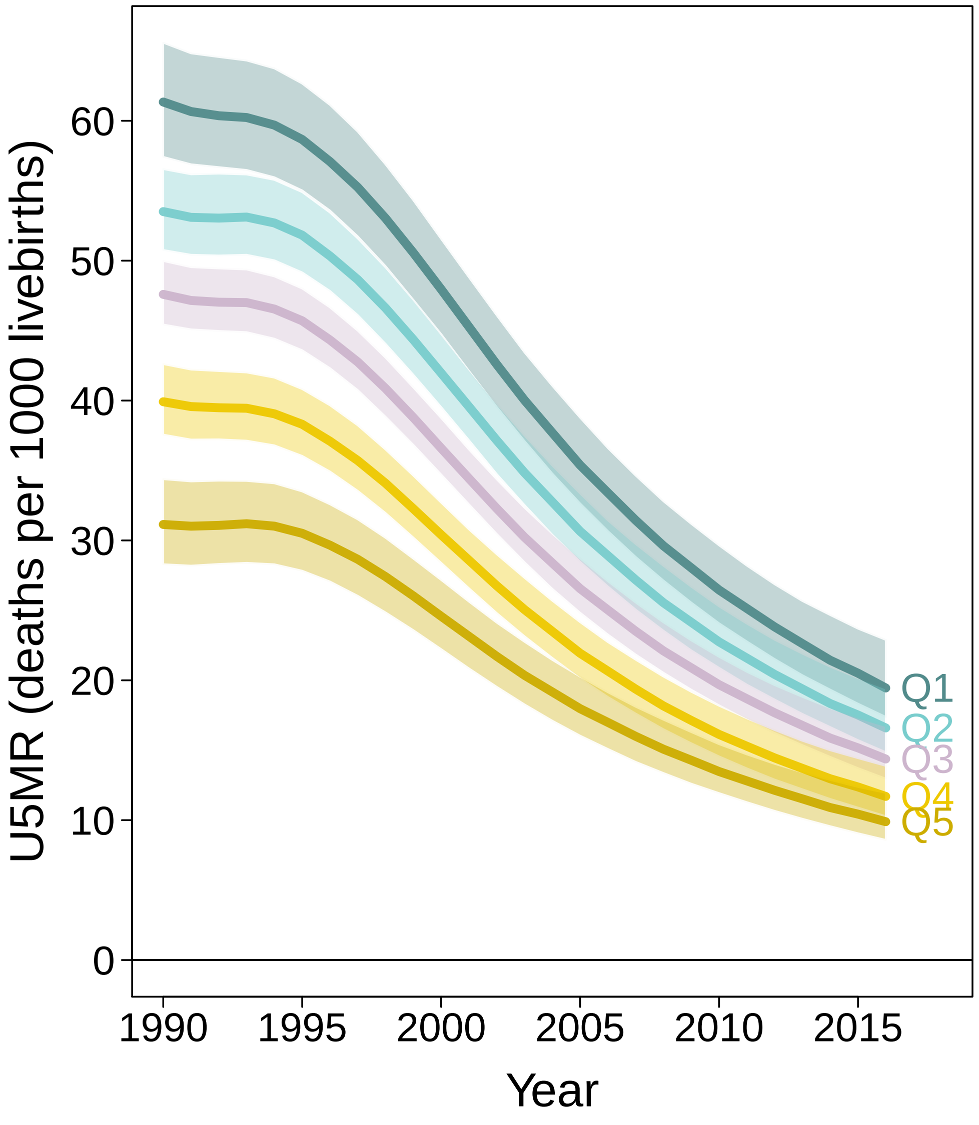

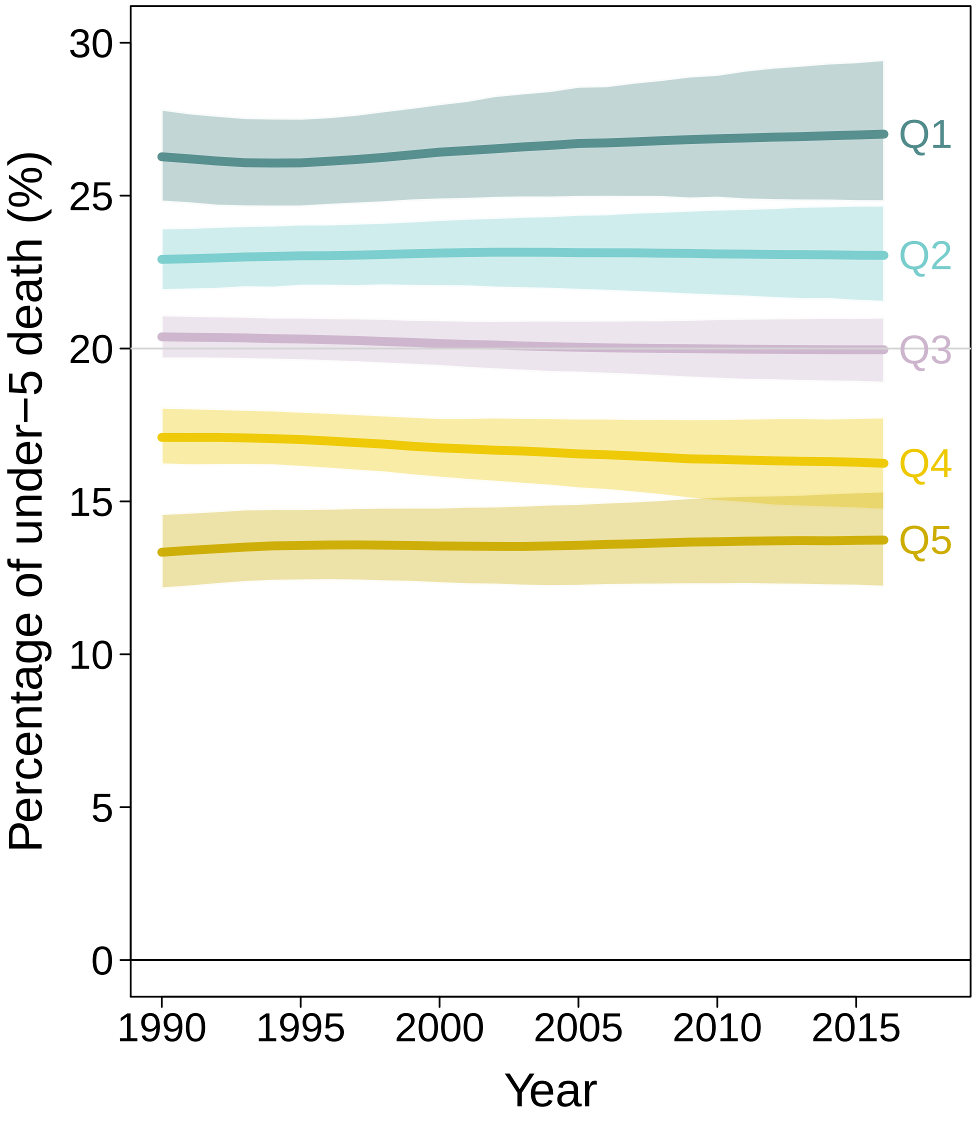

Eastern and Southern Africa

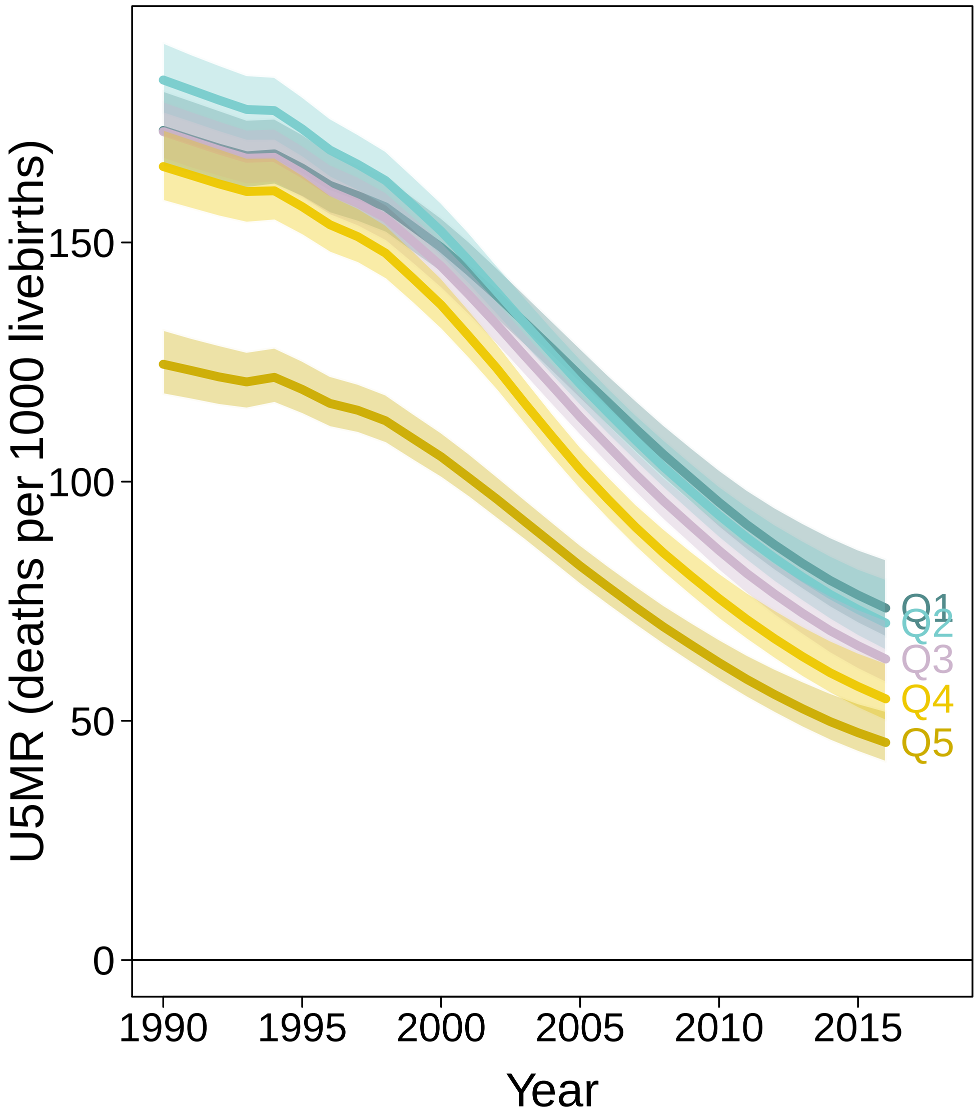

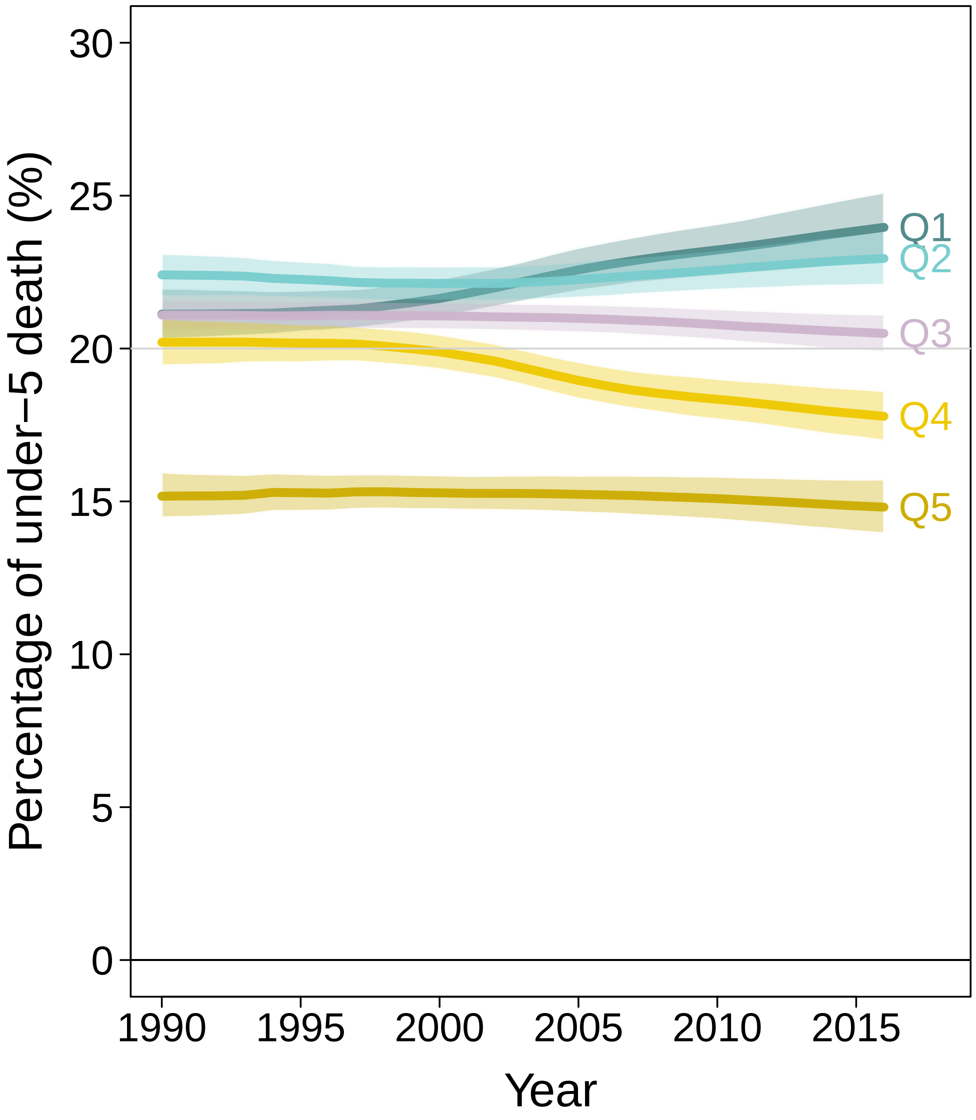

West and Central Africa

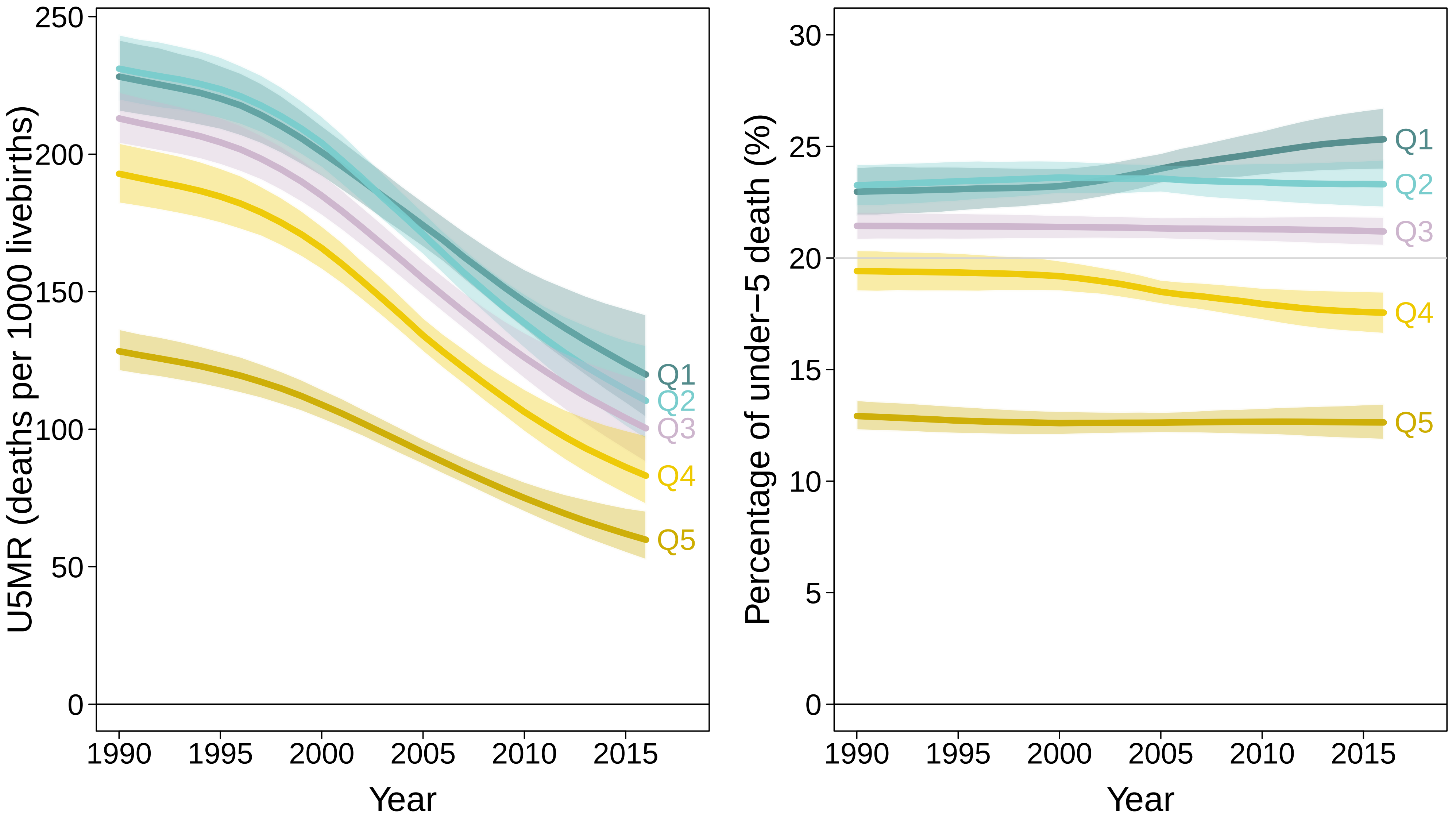

Latin America and Caribbean

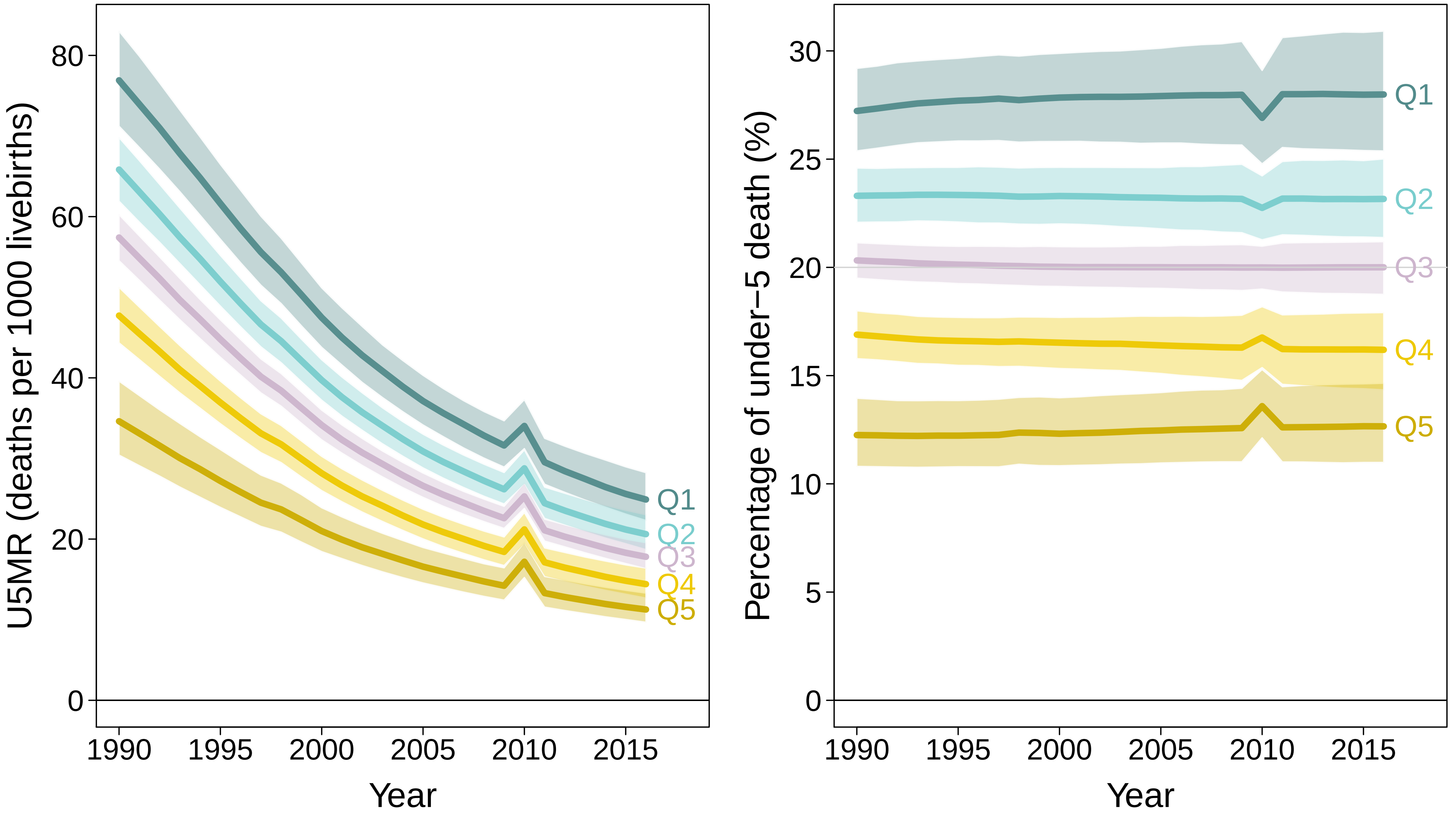

East Asia and Pacific (excluding China)

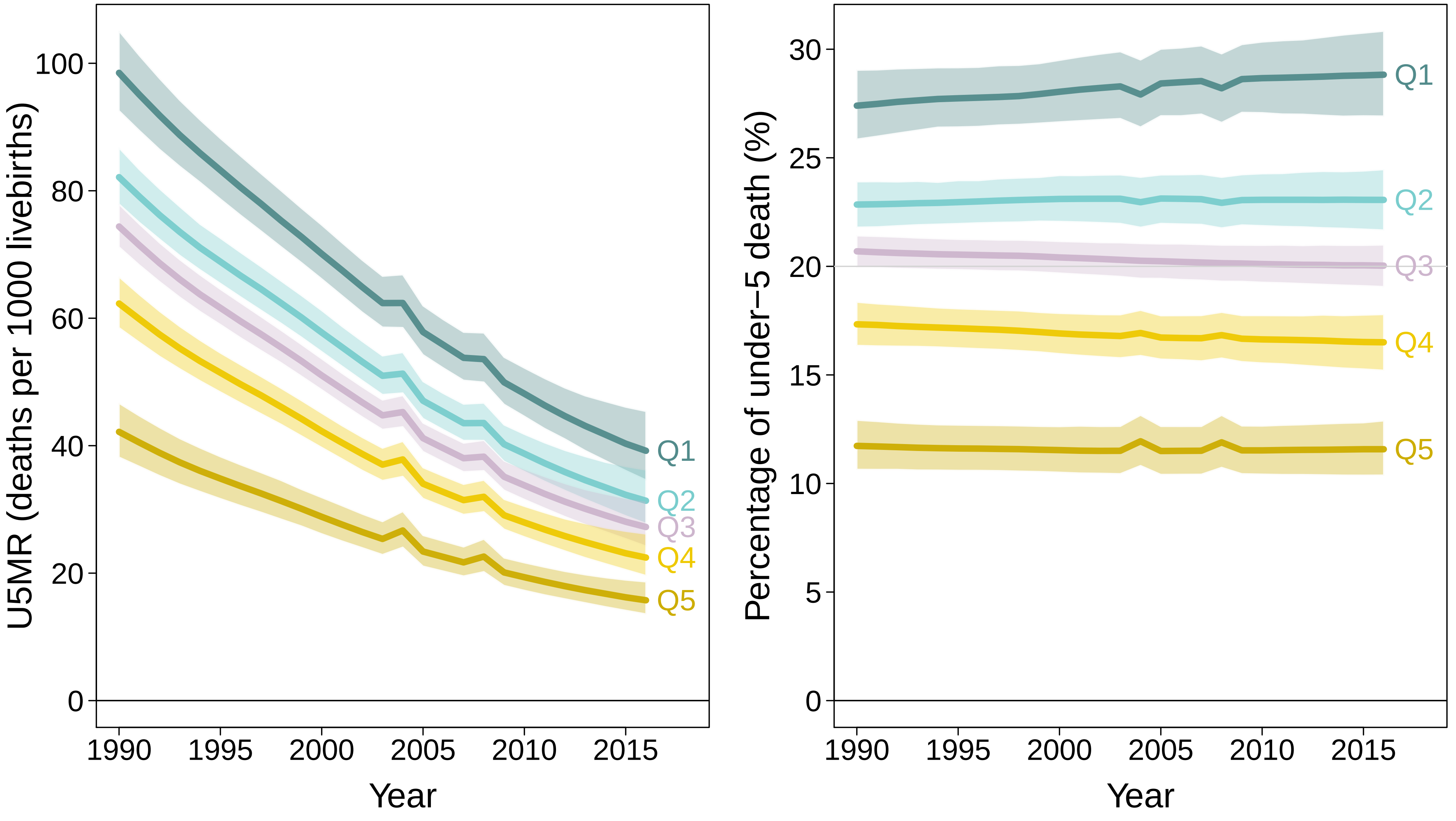

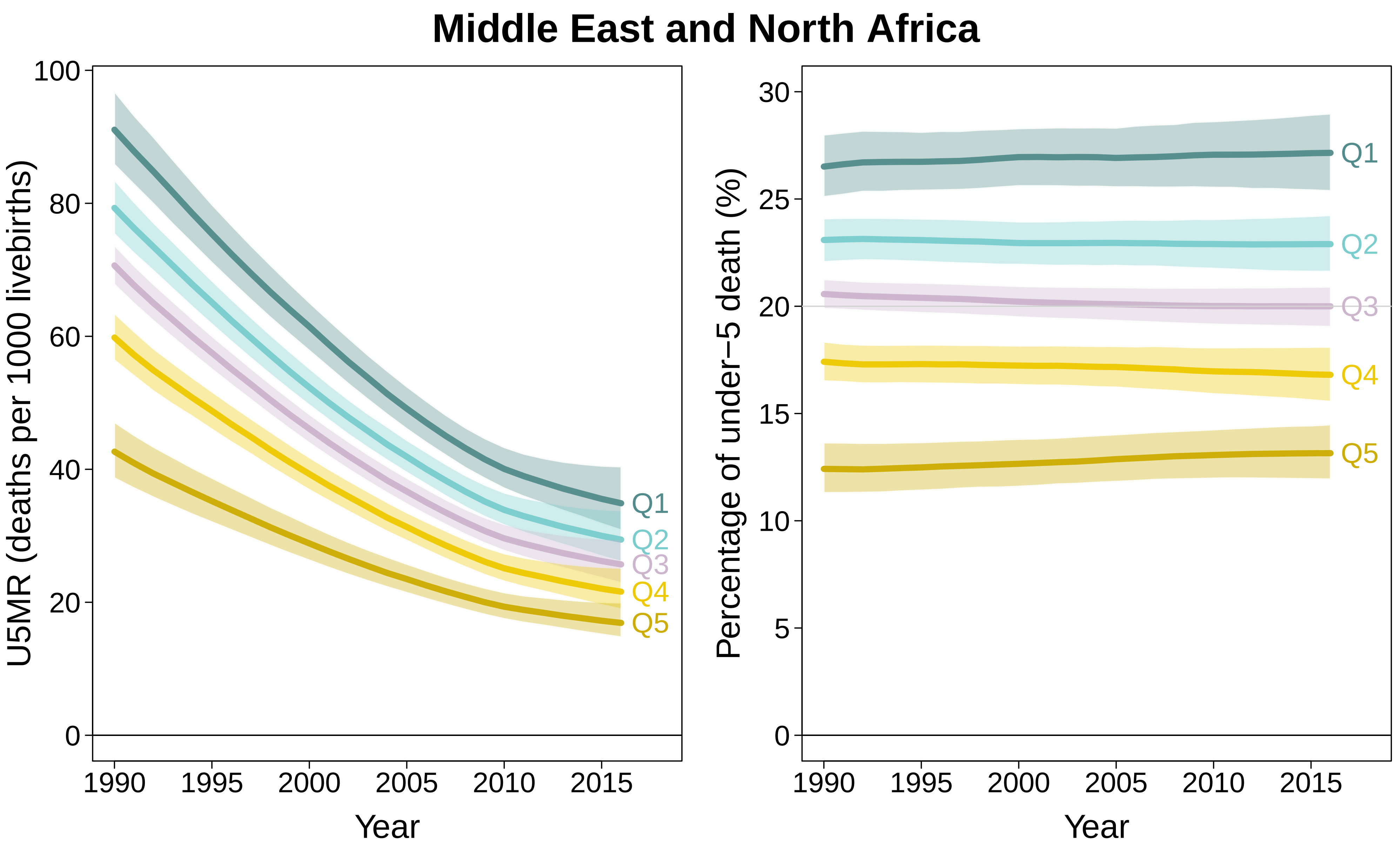
Middle East and North Africa
U5MR (deaths per 1000 livebirths)
100
80
60
40
20
0
1990
1995
2000
2005
2010
2015
Year
Q1
Q2
Q3
Q4
Q5
Percentage of under−5 death (%)
30
25
20
15
10
5
0
1990
1995
2000
2005
2010
2015
Year
Q1
Q2
Q3
Q4
Q5

Figure 9: **U5MR by wealth quintile, for the 99 countries with empirical data.** Solid curves are point estimates from the model. Shaded areas around the solid curves are the 90% uncertainty intervals. Vertical grey lines indicate the most recent reference year of data points for each country. Q1: the 1st wealth quintile, the 20% poorest wealth quintile; Q5: the 5th wealth quintile, the 20% richest wealth quintile.

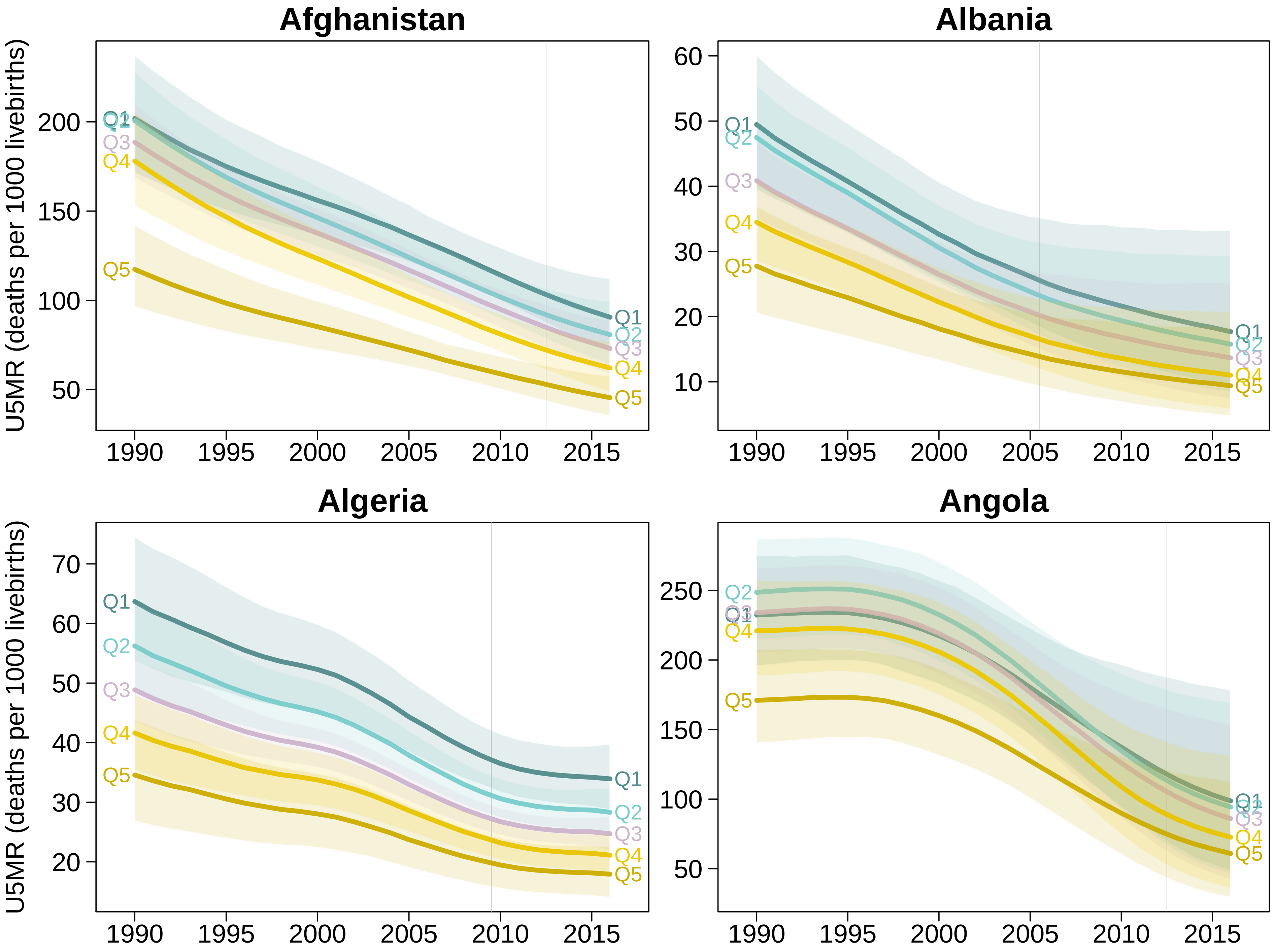
Afghanistan
Albania
Algeria
Angola
U5MR (deaths per 1000 livebirths)
U5MR (deaths per 1000 livebirths)
Q1
Q2
Q3
Q4
Q5
1990
1995
2000
2005
2010
2015

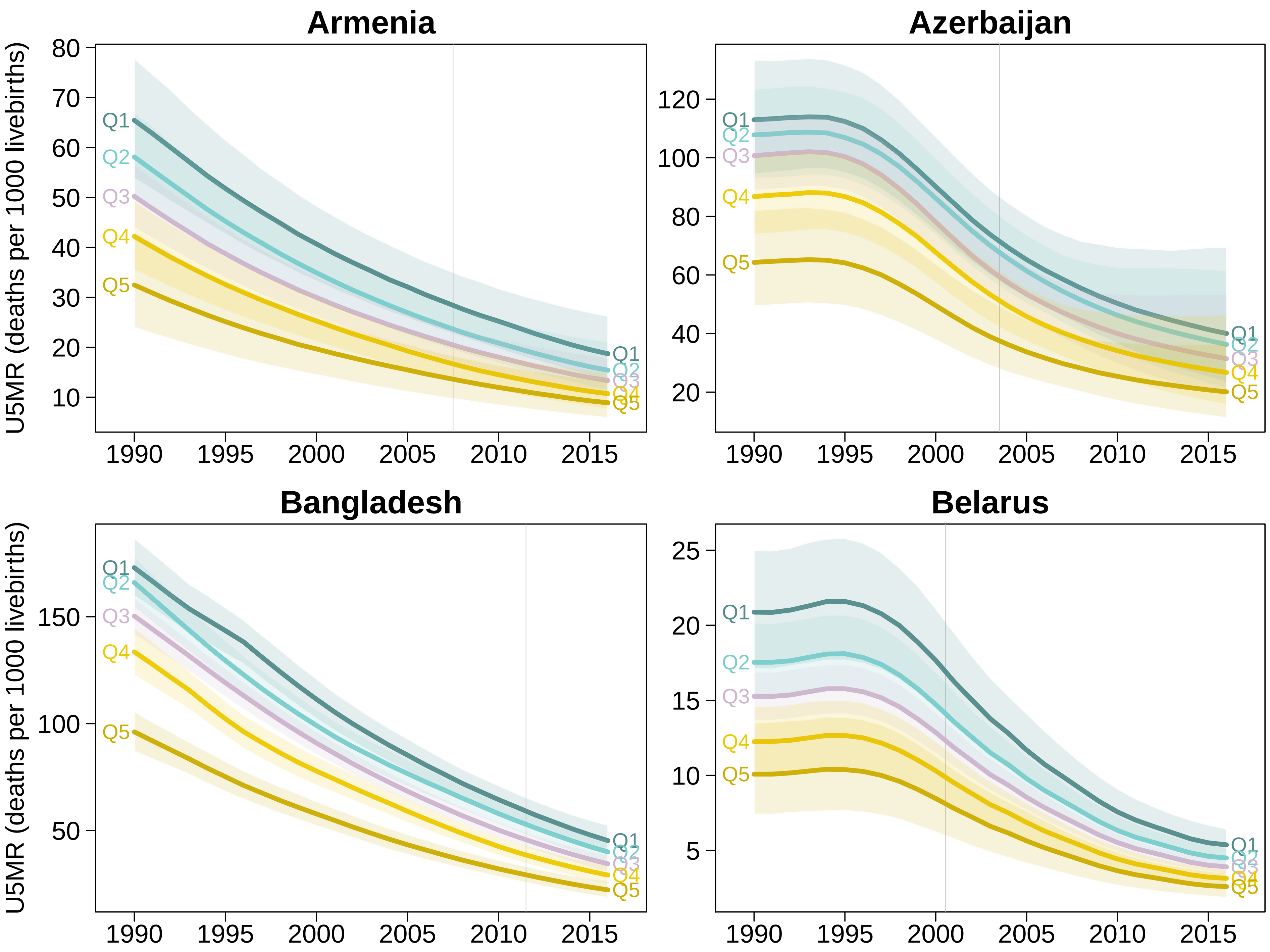
Armenia
Azerbaijan
Bangladesh
Belarus
U5MR (deaths per 1000 livebirths)
U5MR (deaths per 1000 livebirths)
Q1
Q2
Q3
Q4
Q5
1990
1995
2000
2005
2010
2015
80
70
60
50
40
30
20
10
120
100
150
25
5

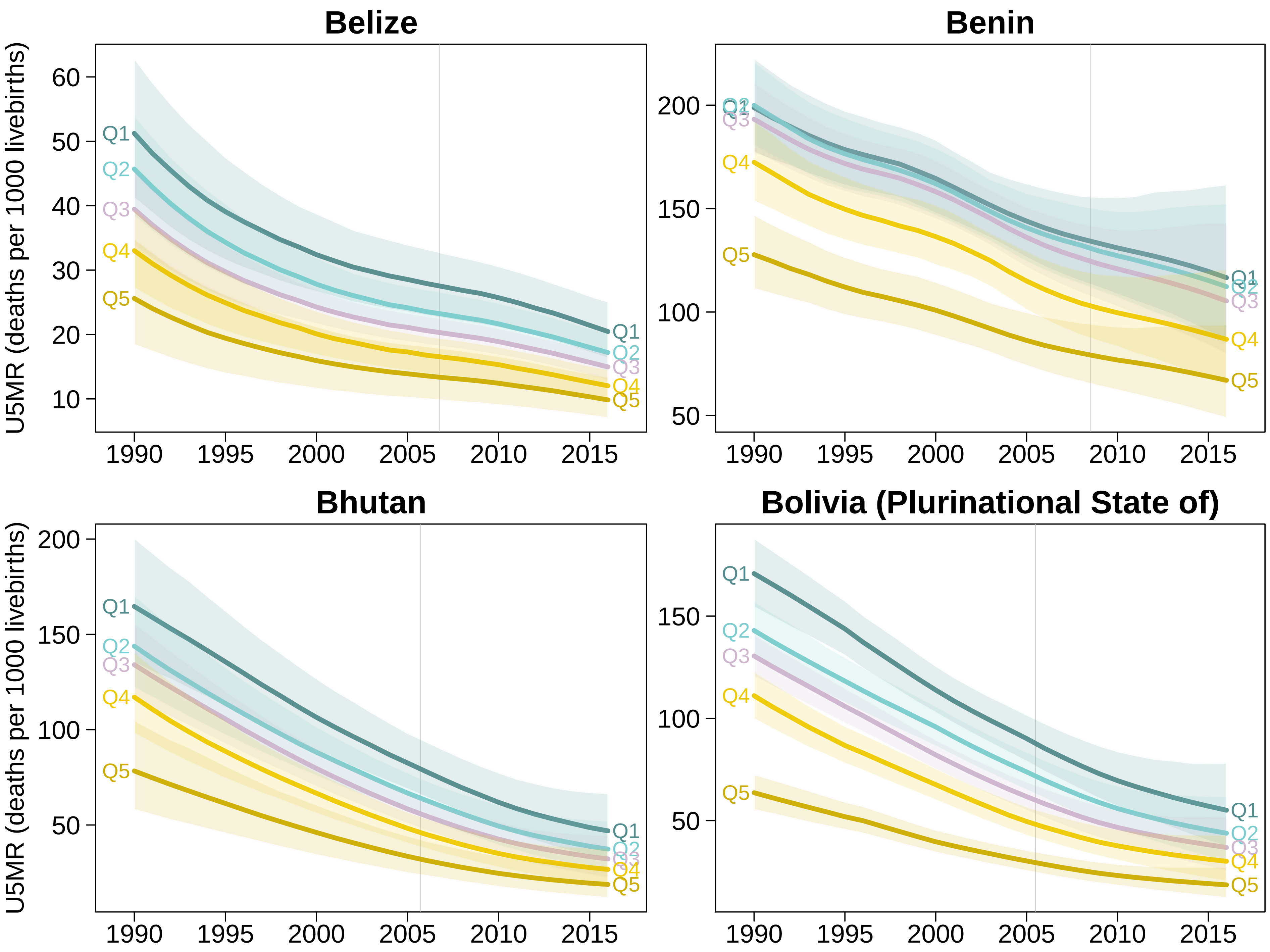
Belize
Benin
Bhutan
Bolivia (Plurinational State of)
U5MR (deaths per 1000 livebirths)
Q1
Q2
Q3
Q4
Q5
1990
1995
2000
2005
2010
2015

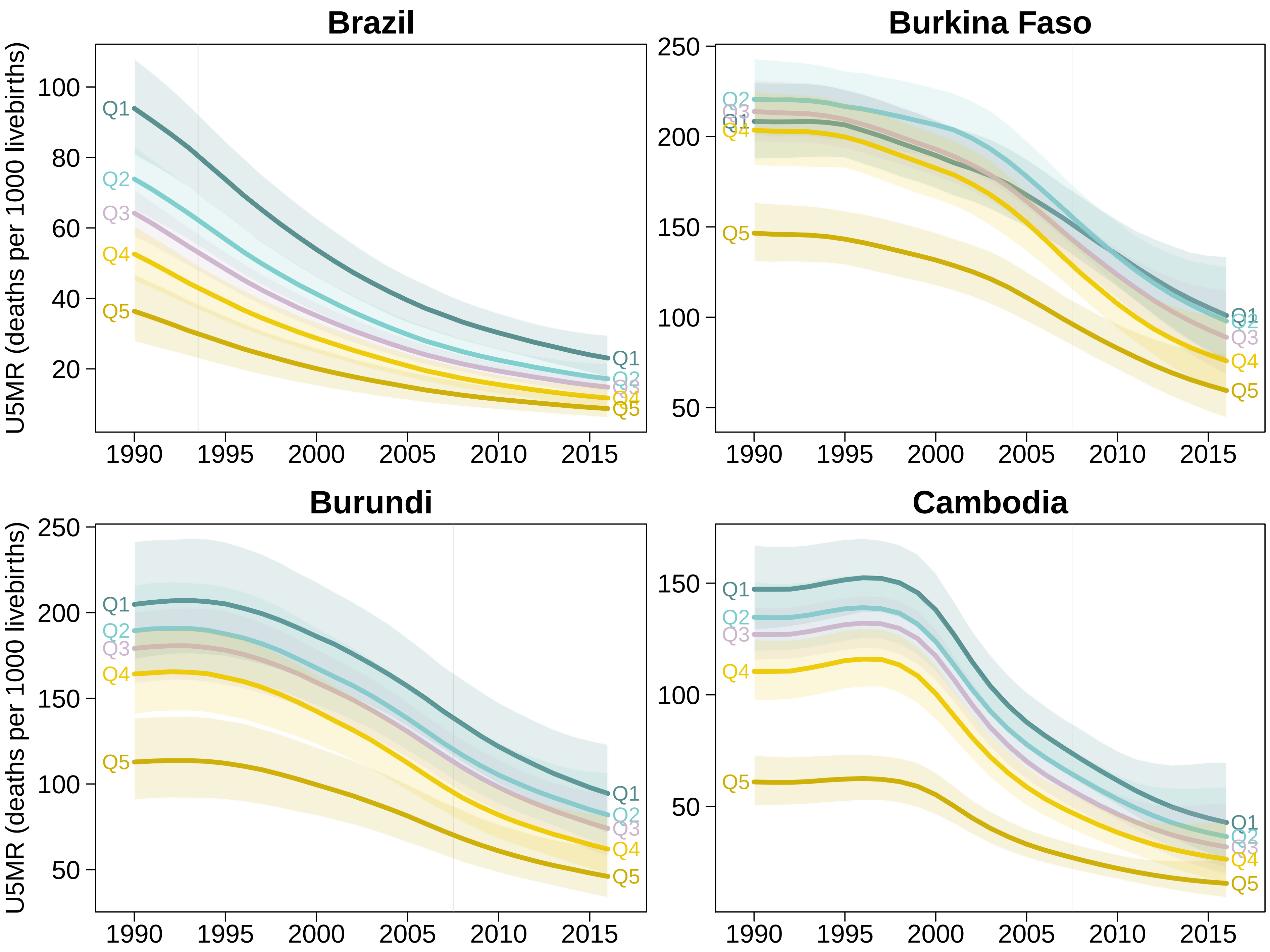

Brazil
Burkina Faso
Burundi
Cambodia
U5MR (deaths per 1000 livebirths)
Q1
Q2
Q3
Q4
Q5
1990
1995
2000
2005
2010
2015

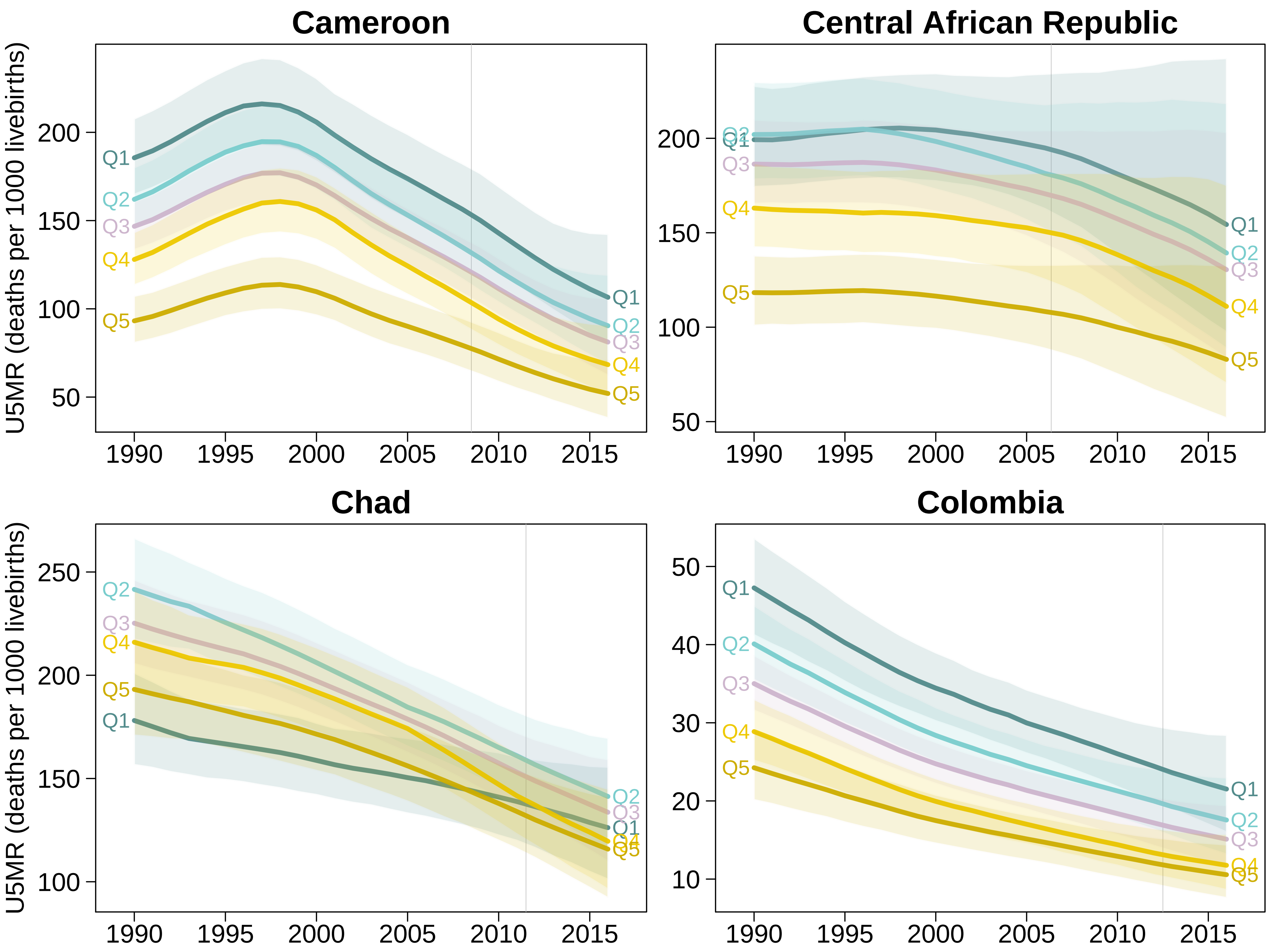
Cameroon
Central African Republic
Chad
Colombia
U5MR (deaths per 1000 livebirths)
Q1
Q2
Q3
Q4
Q5
1990
1995
2000
2005
2010
2015

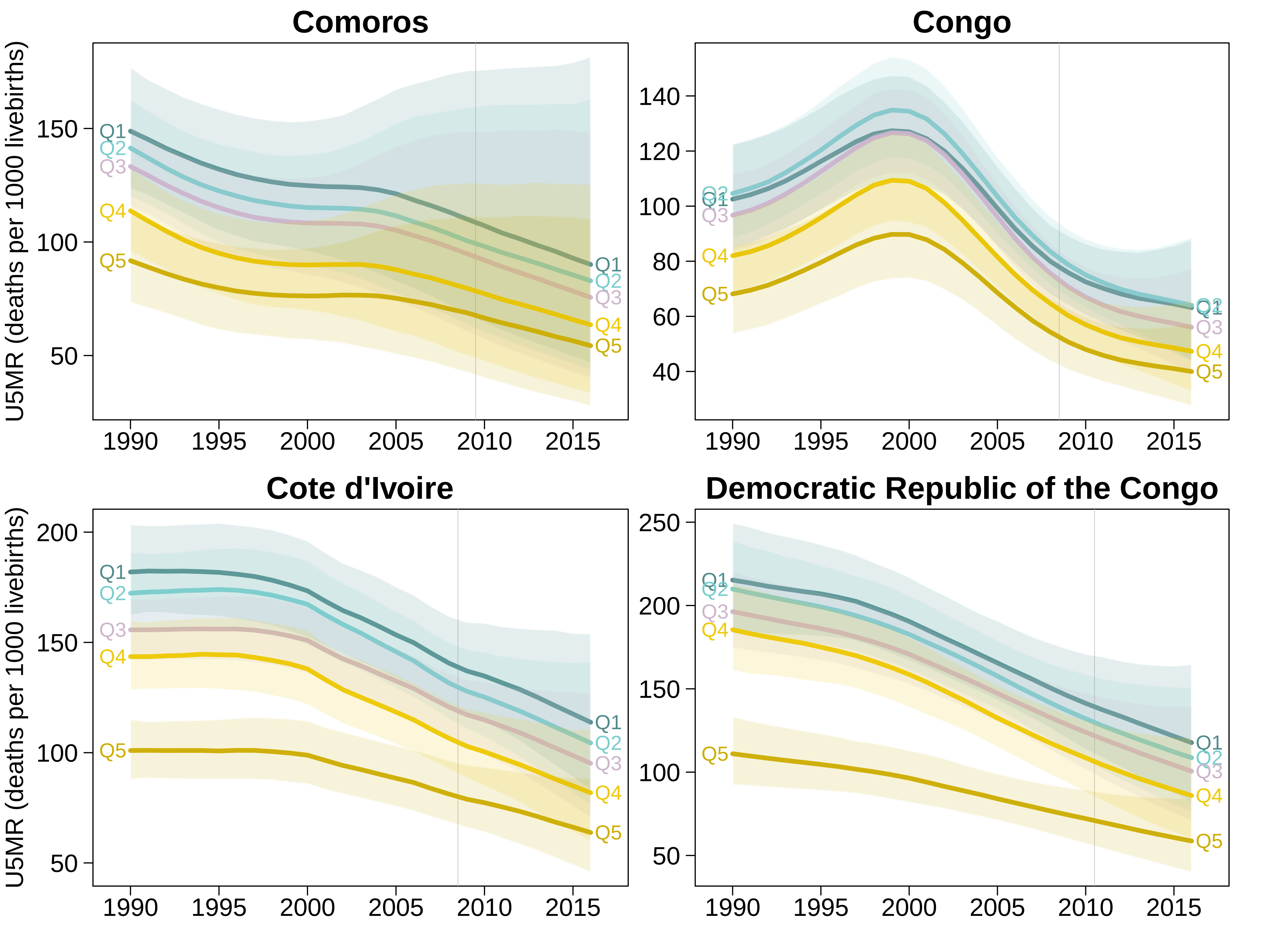
Comoros
Congo
Cote d'Ivoire
Democratic Republic of the Congo
U5MR (deaths per 1000 livebirths)
Q1
Q2
Q3
Q4
Q5
1990
1995
2000
2005
2010
2015

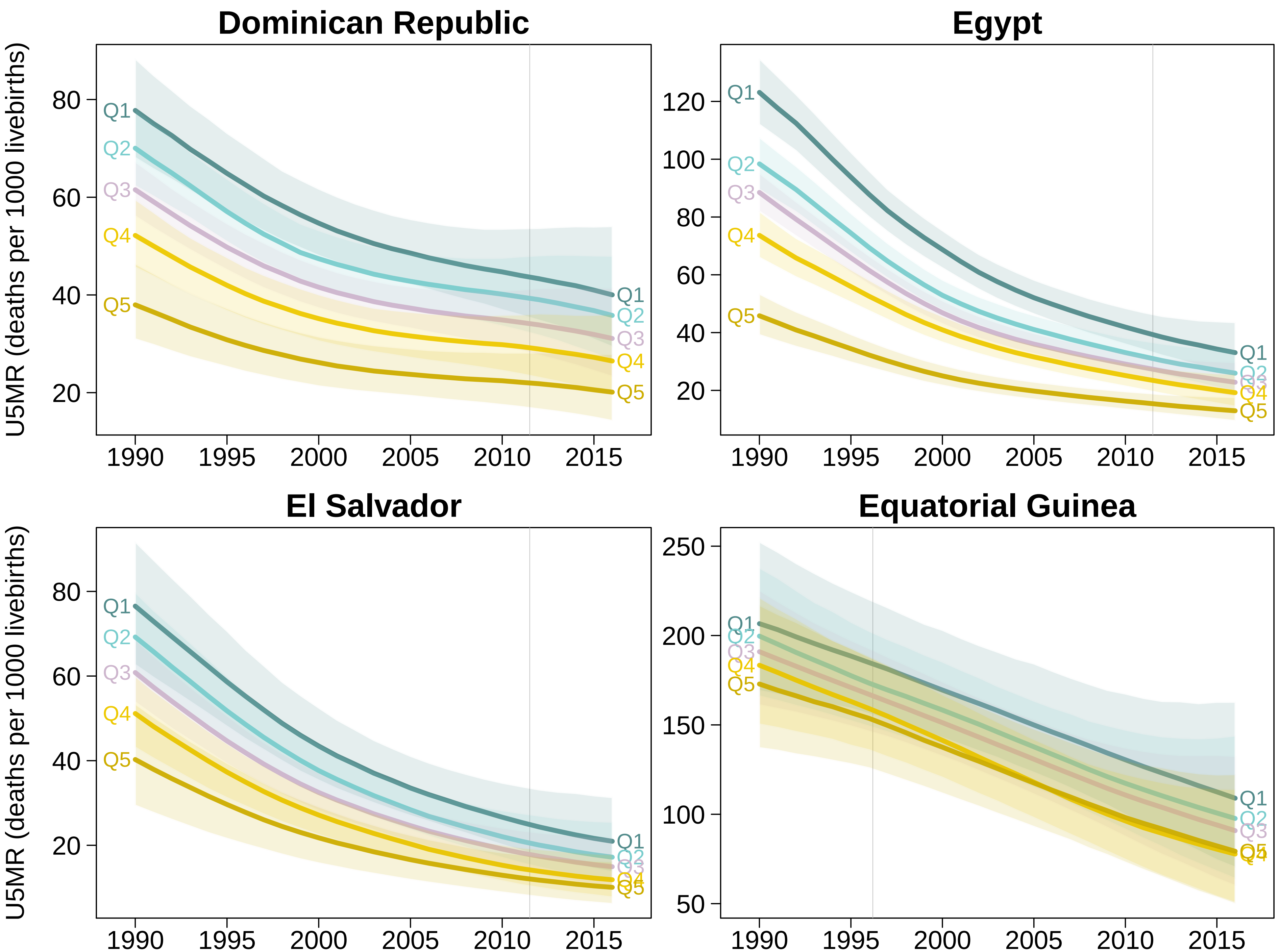
Dominican Republic
Egypt
El Salvador
Equatorial Guinea
U5MR (deaths per 1000 livebirths)
U5MR (deaths per 1000 livebirths)
Q1
Q2
Q3
Q4
Q5
1990
1995
2000
2005
2010
2015

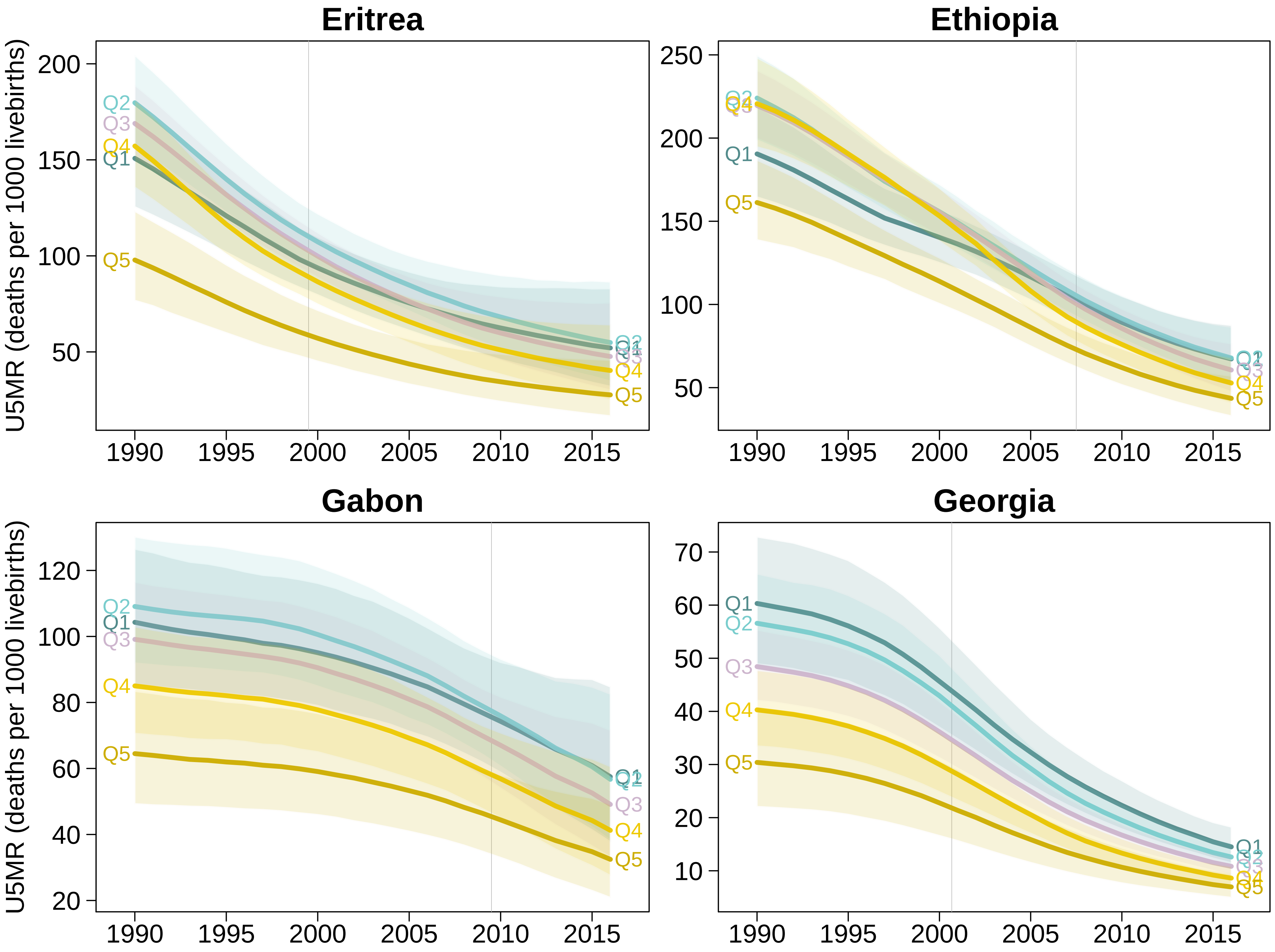

Eritrea
Ethiopia
Gabon
Georgia
U5MR (deaths per 1000 livebirths)
Q1
Q2
Q3
Q4
Q5
1990
1995
2000
2005
2010
2015

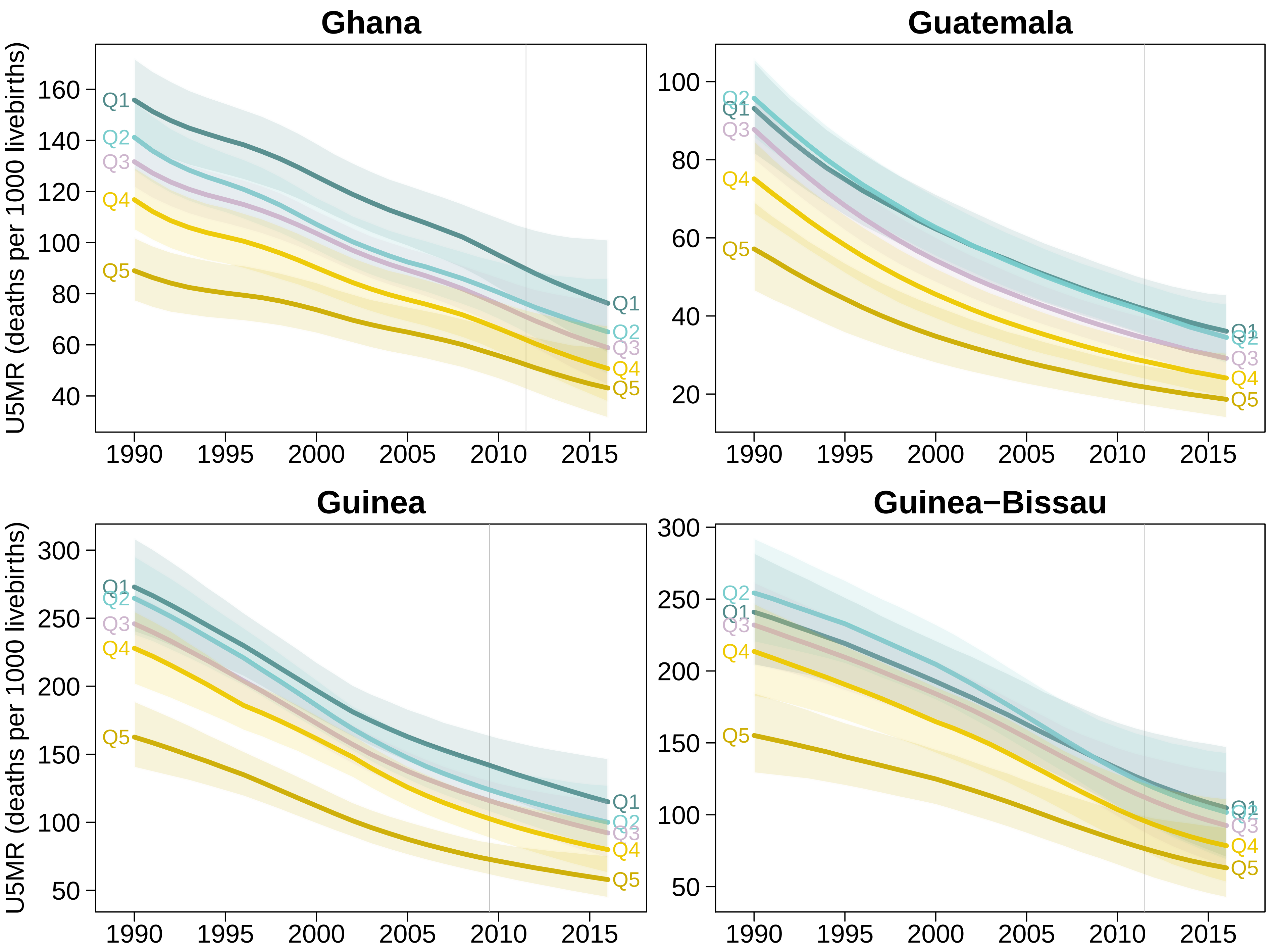

Ghana
Guatemala
Guinea
Guinea−Bissau
U5MR (deaths per 1000 livebirths)
Q1
Q2
Q3
Q4
Q5
1990
1995
2000
2005
2010
2015

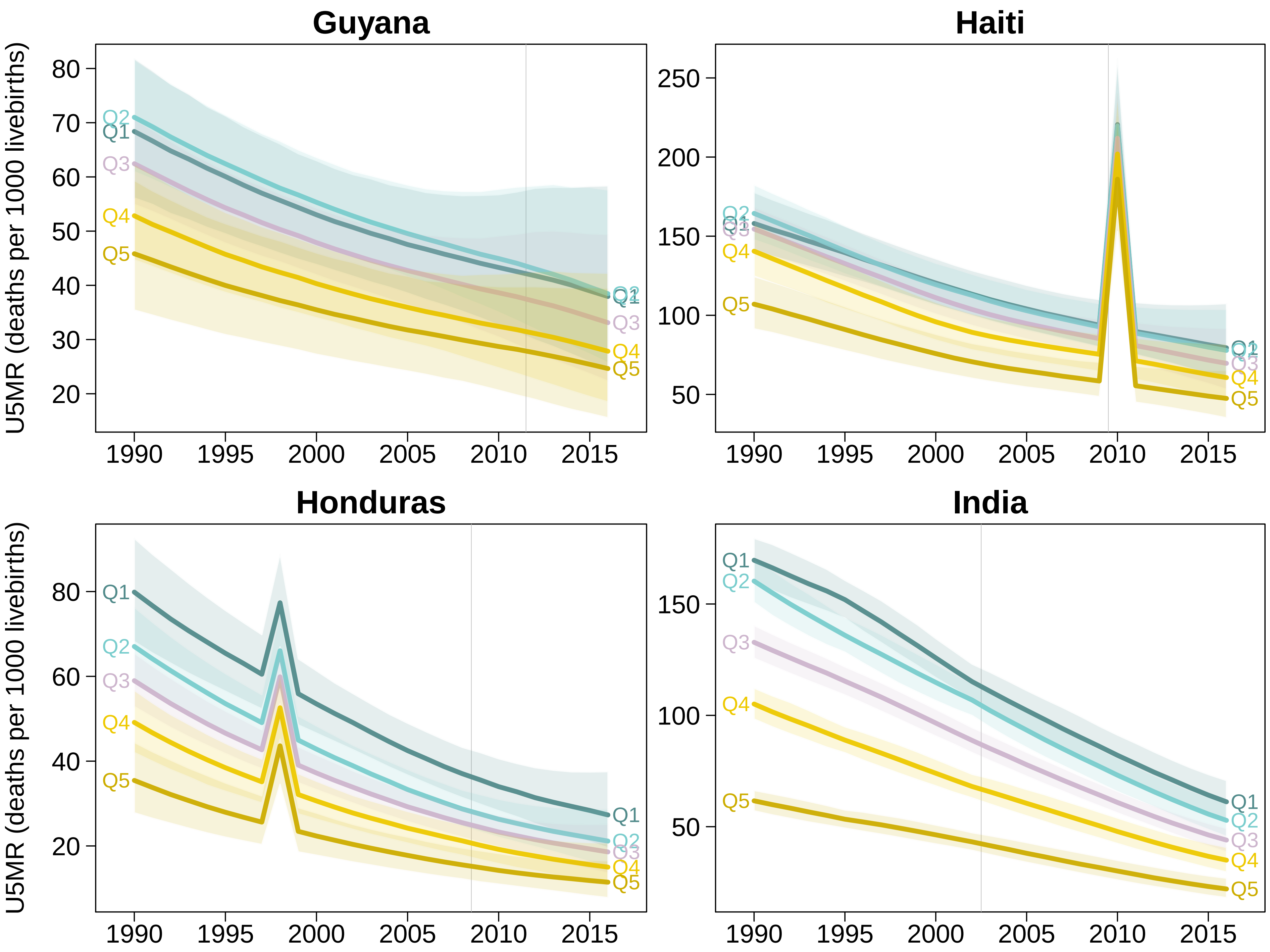
Guyana
Haiti
Honduras
India
U5MR (deaths per 1000 livebirths)
1990
1995
2000
2005
2010
2015
Q1
Q2
Q3
Q4
Q5

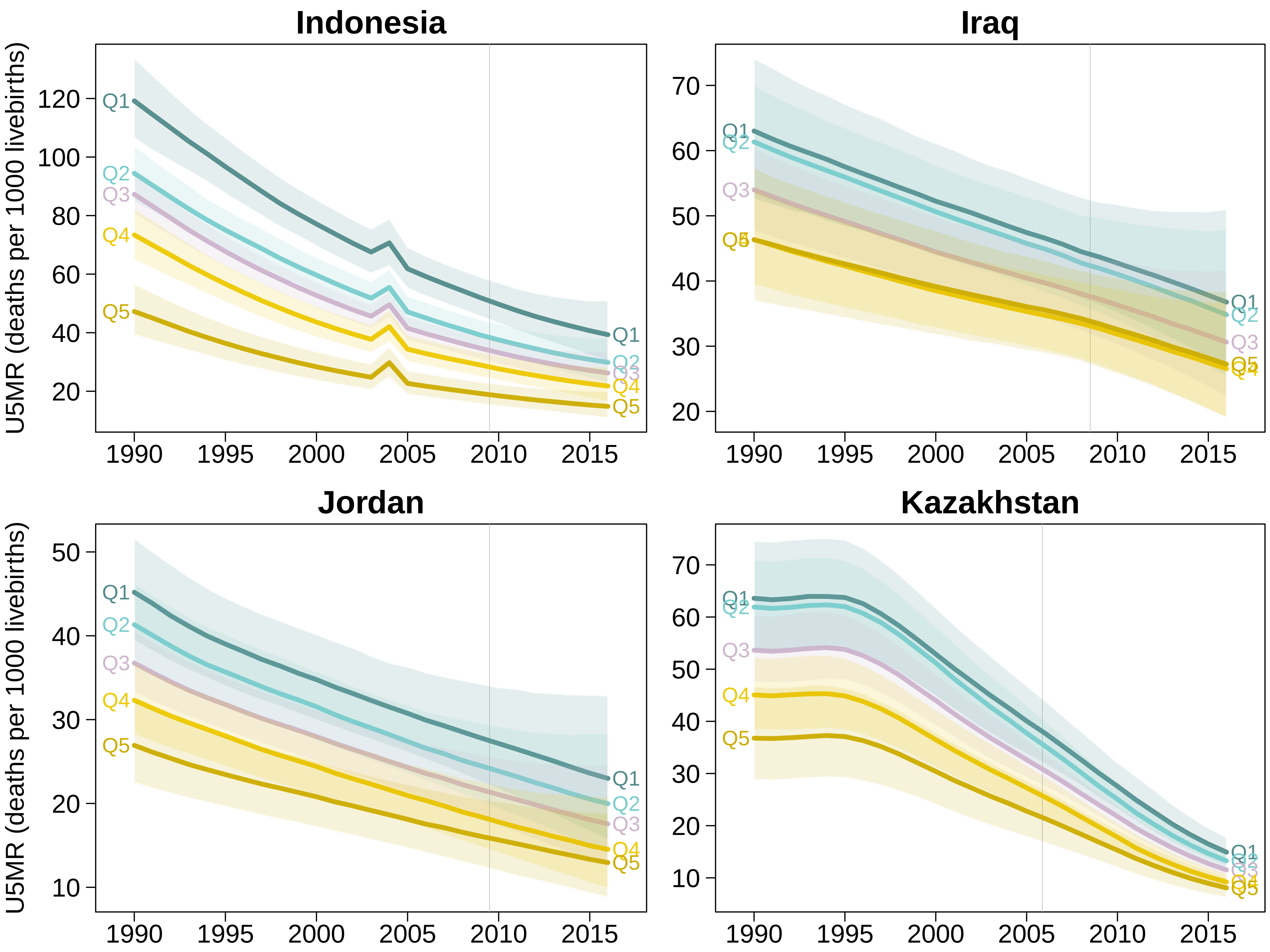

Indonesia
Iraq
Jordan
Kazakhstan
U5MR (deaths per 1000 livebirths)
U5MR (deaths per 1000 livebirths)
Q1
Q2
Q3
Q4
Q5
1990
1995
2000
2005
2010
2015

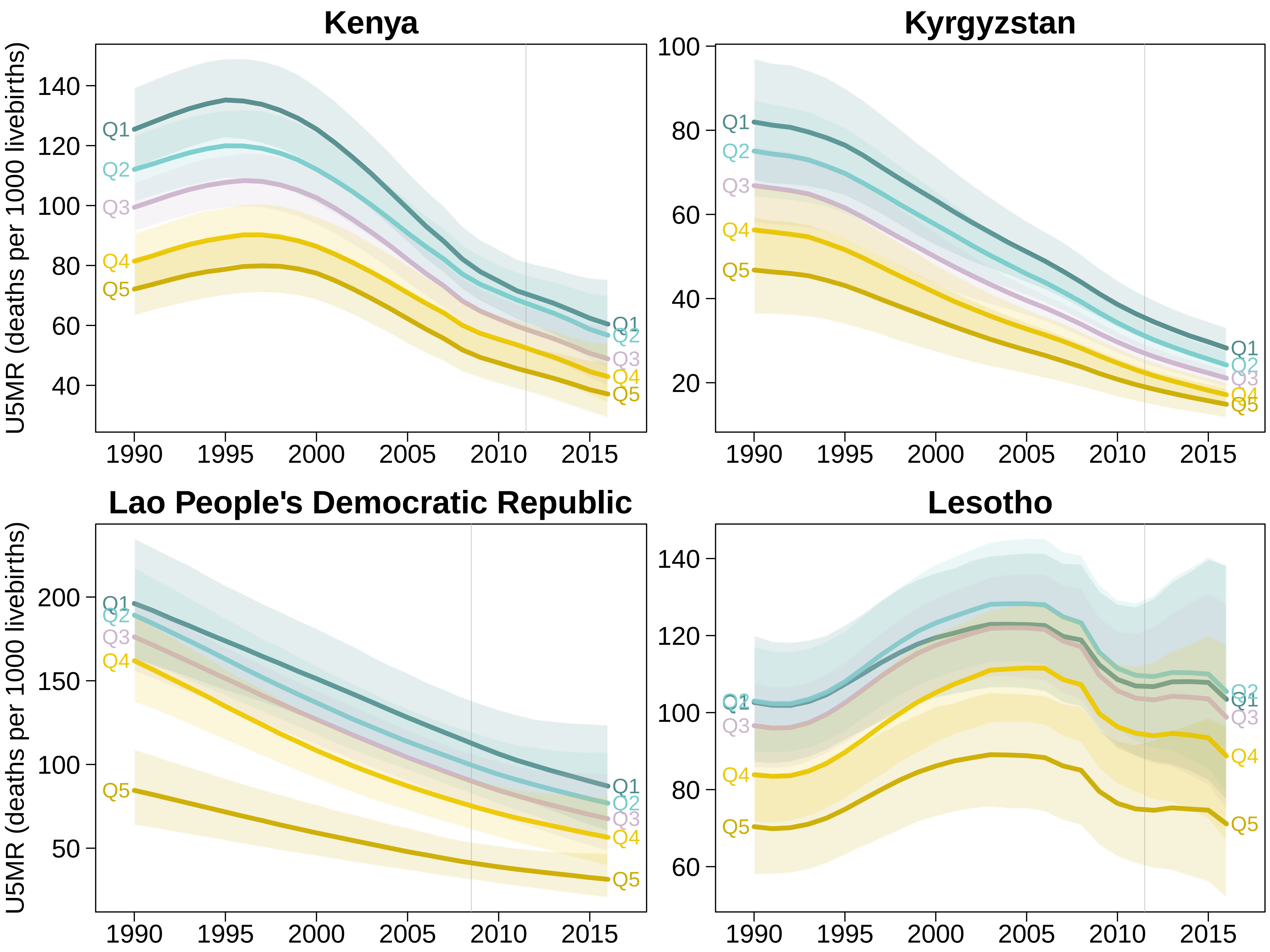
Kenya
Kyrgyzstan
Lao People's Democratic Republic
Lesotho
U5MR (deaths per 1000 livebirths)
U5MR (deaths per 1000 livebirths)
Q1
Q2
Q3
Q4
Q5
1990
1995
2000
2005
2010
2015

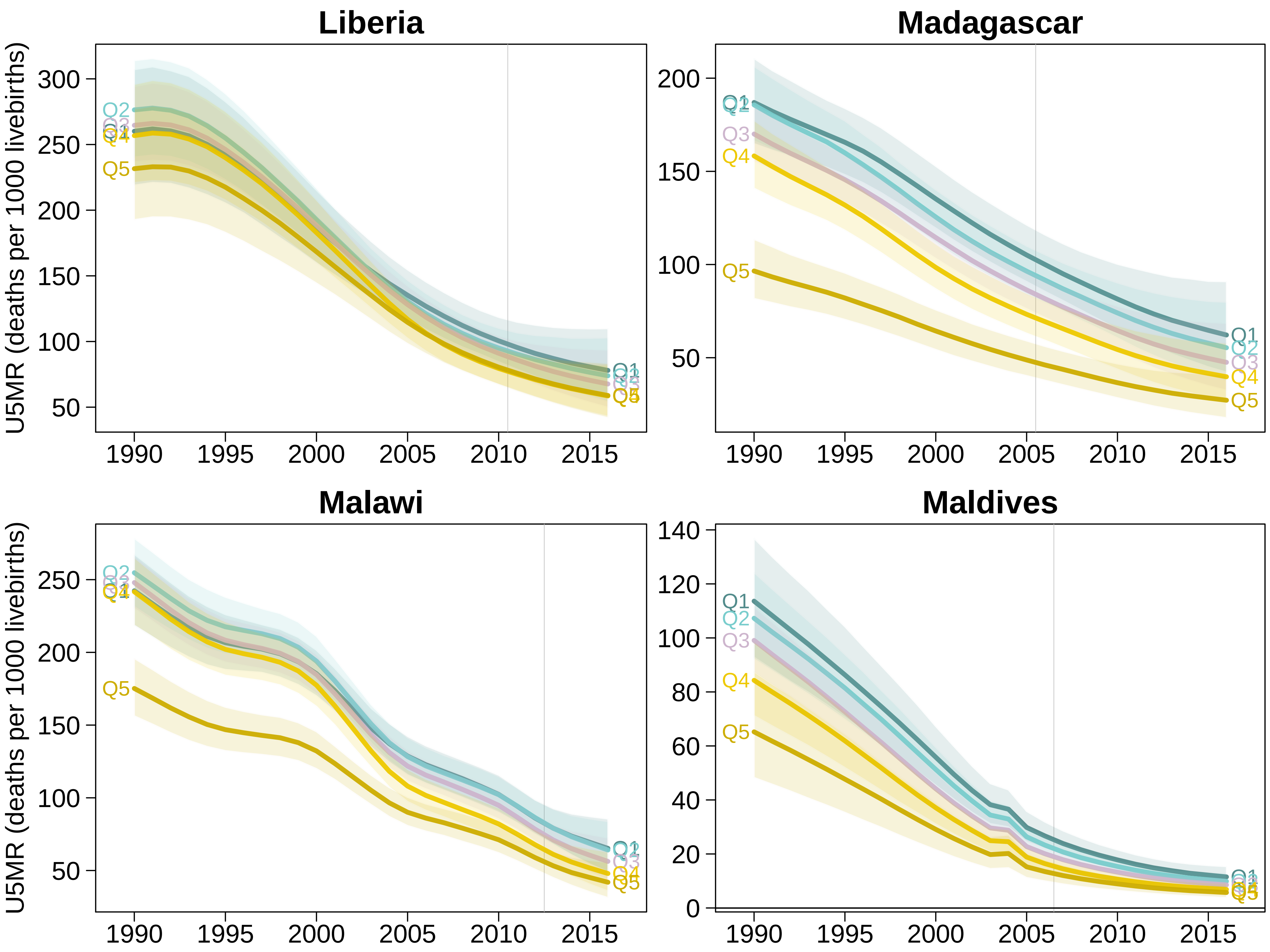
Liberia
Madagascar
Malawi
Maldives
U5MR (deaths per 1000 livebirths)
Q1
Q2
Q3
Q4
Q5
1990
1995
2000
2005
2010
2015

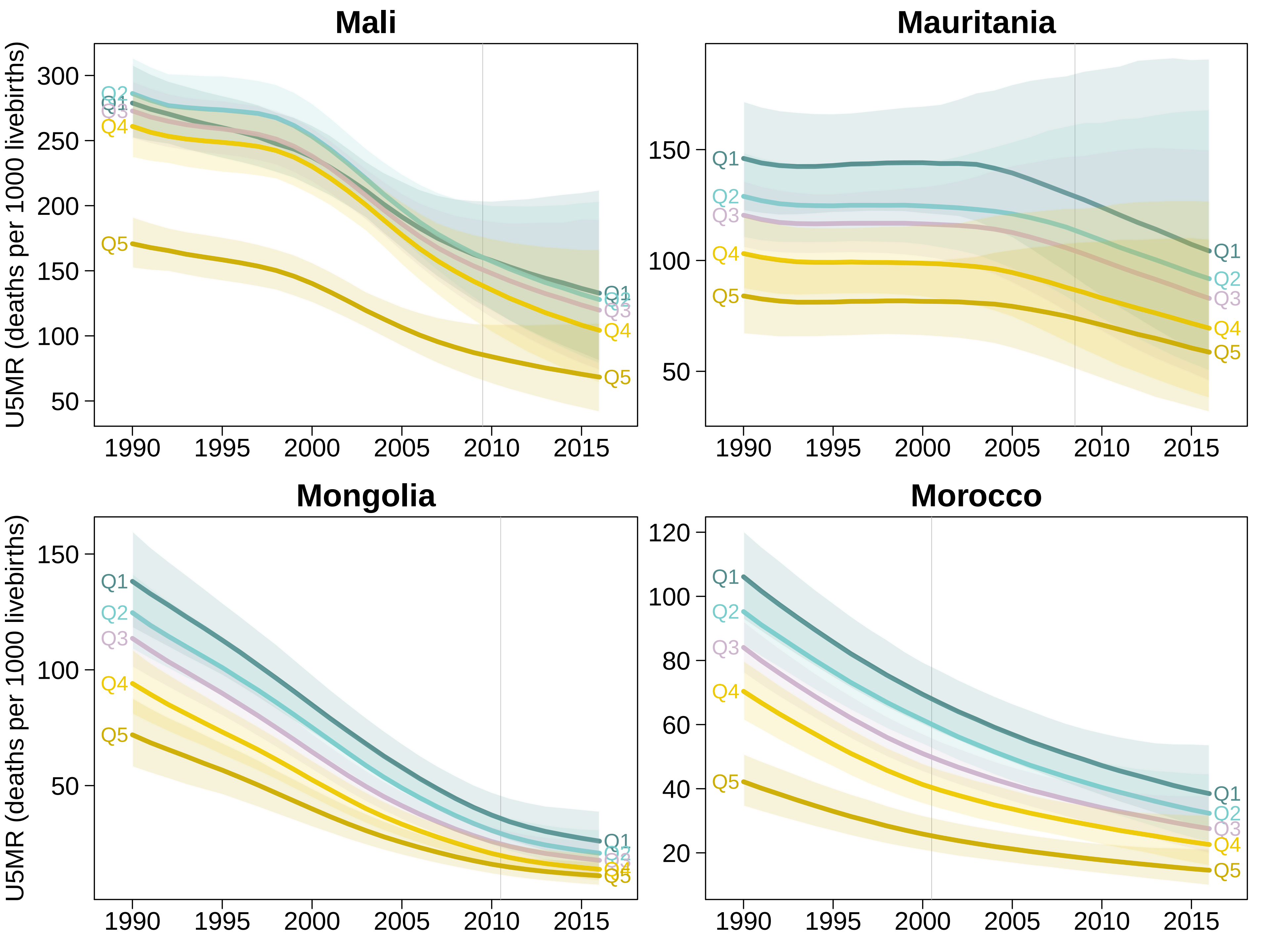
Mali
Mauritania
Mongolia
Morocco
U5MR (deaths per 1000 livebirths)
Q1
Q2
Q3
Q4
Q5
1990
1995
2000
2005
2010
2015

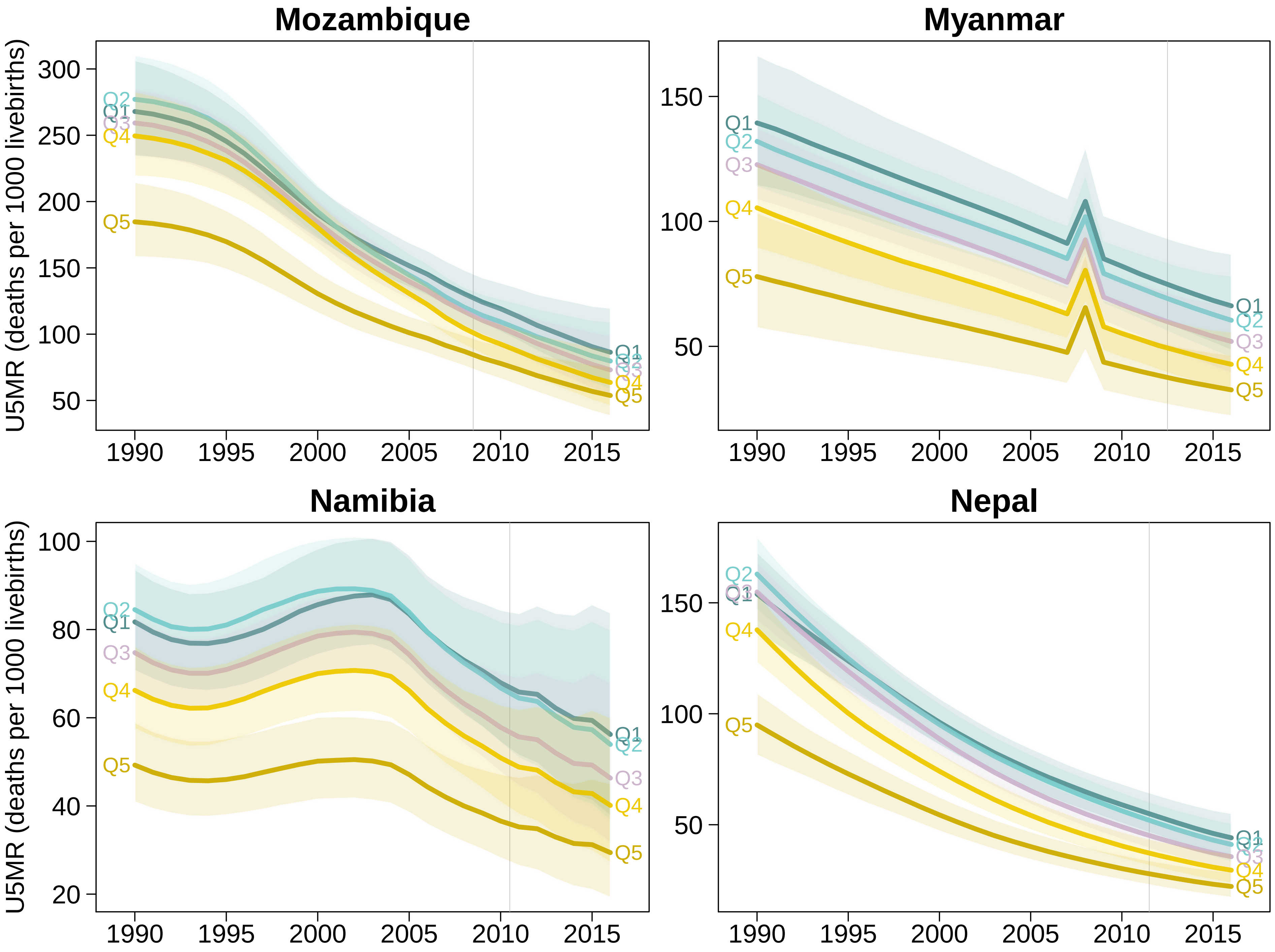

Mozambique
Myanmar
Namibia
Nepal
U5MR (deaths per 1000 livebirths)
U5MR (deaths per 1000 livebirths)
Q1
Q2
Q3
Q4
Q5

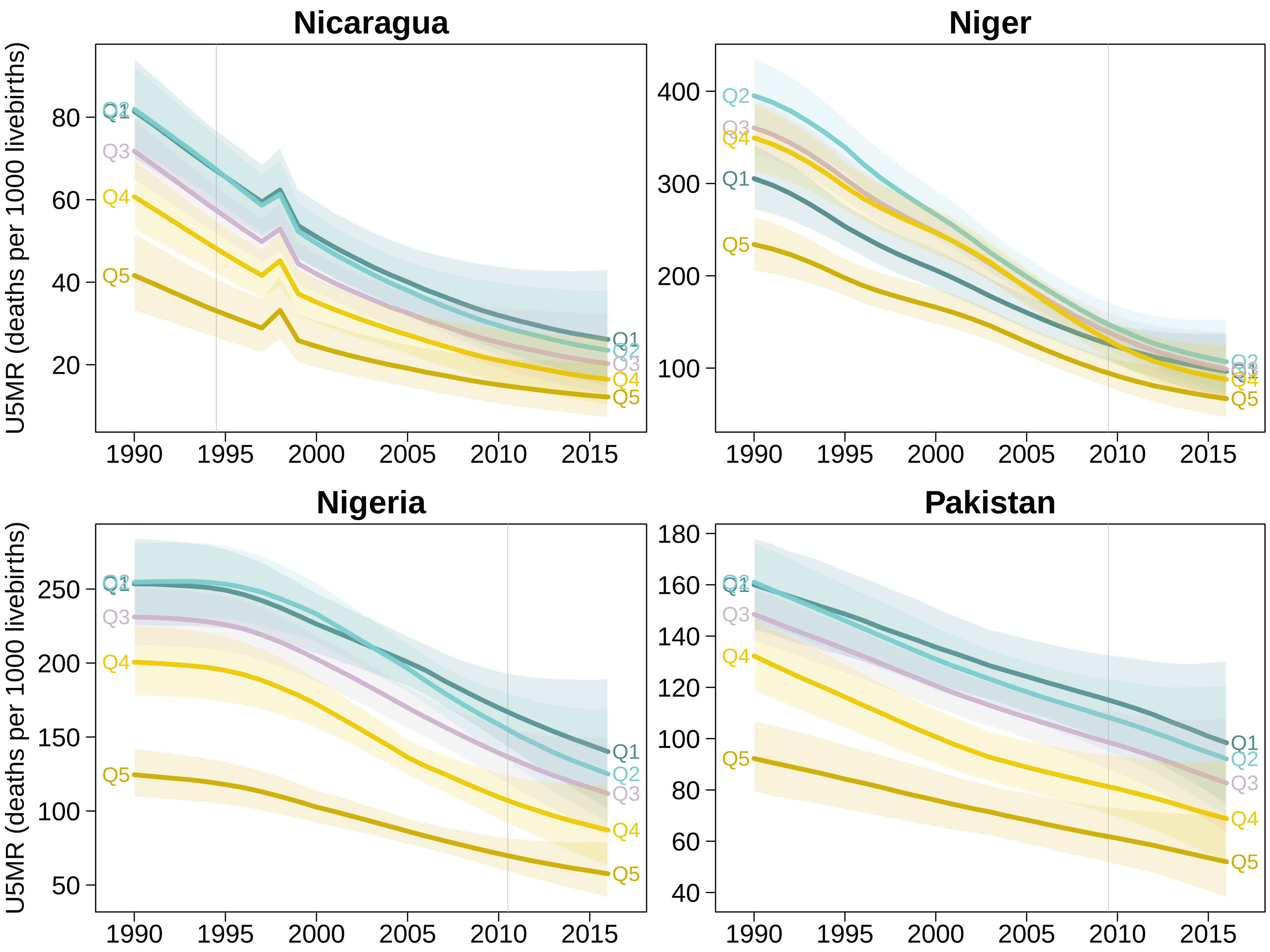
Nicaragua
Niger
Nigeria
Pakistan
U5MR (deaths per 1000 livebirths)
Q1
Q2
Q3
Q4
Q5
1990
1995
2000
2005
2010
2015

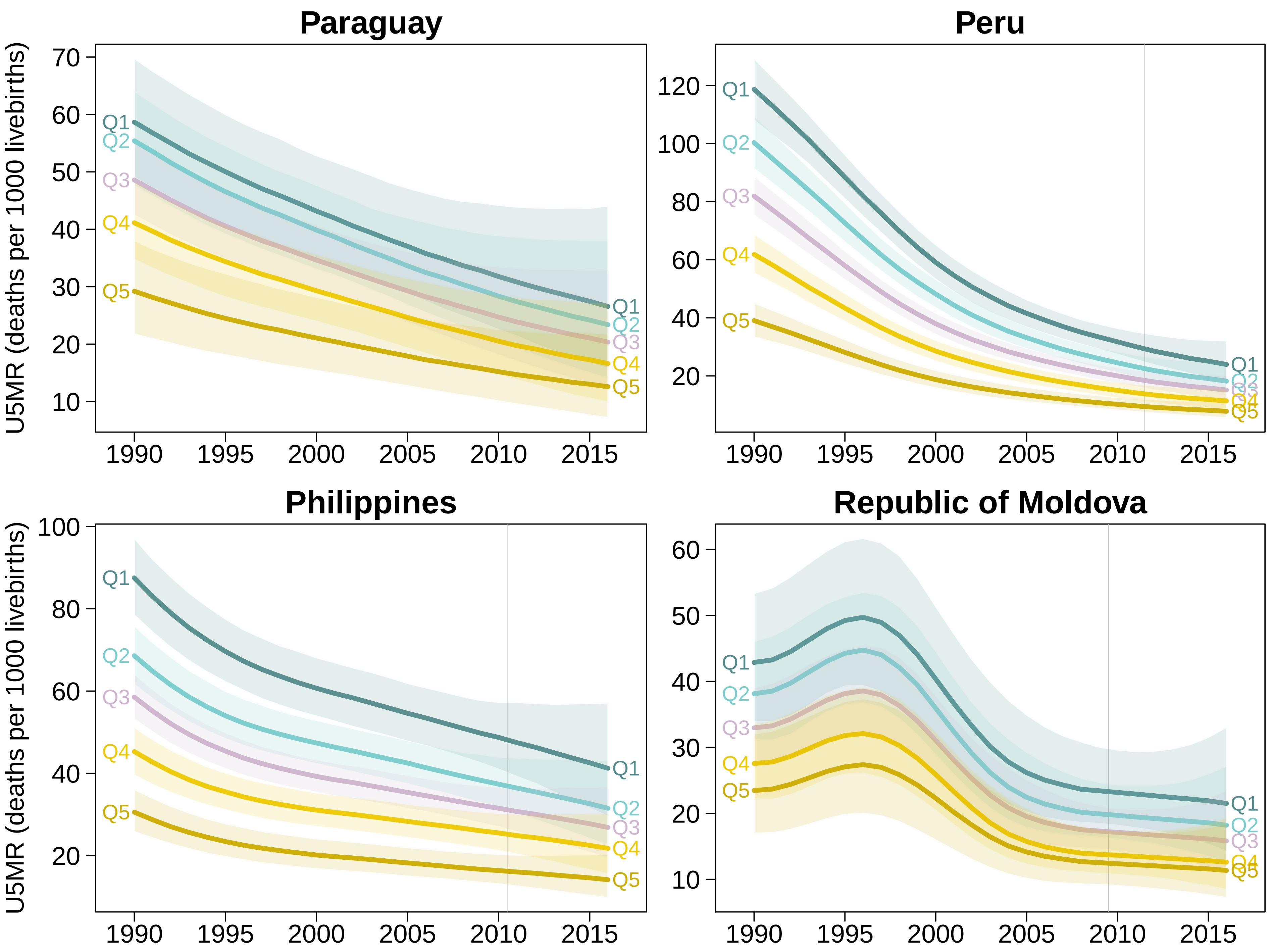
Paraguay
Peru
Philippines
Republic of Moldova
U5MR (deaths per 1000 livebirths)
Q1
Q2
Q3
Q4
Q5
1990
1995
2000
2005
2010
2015

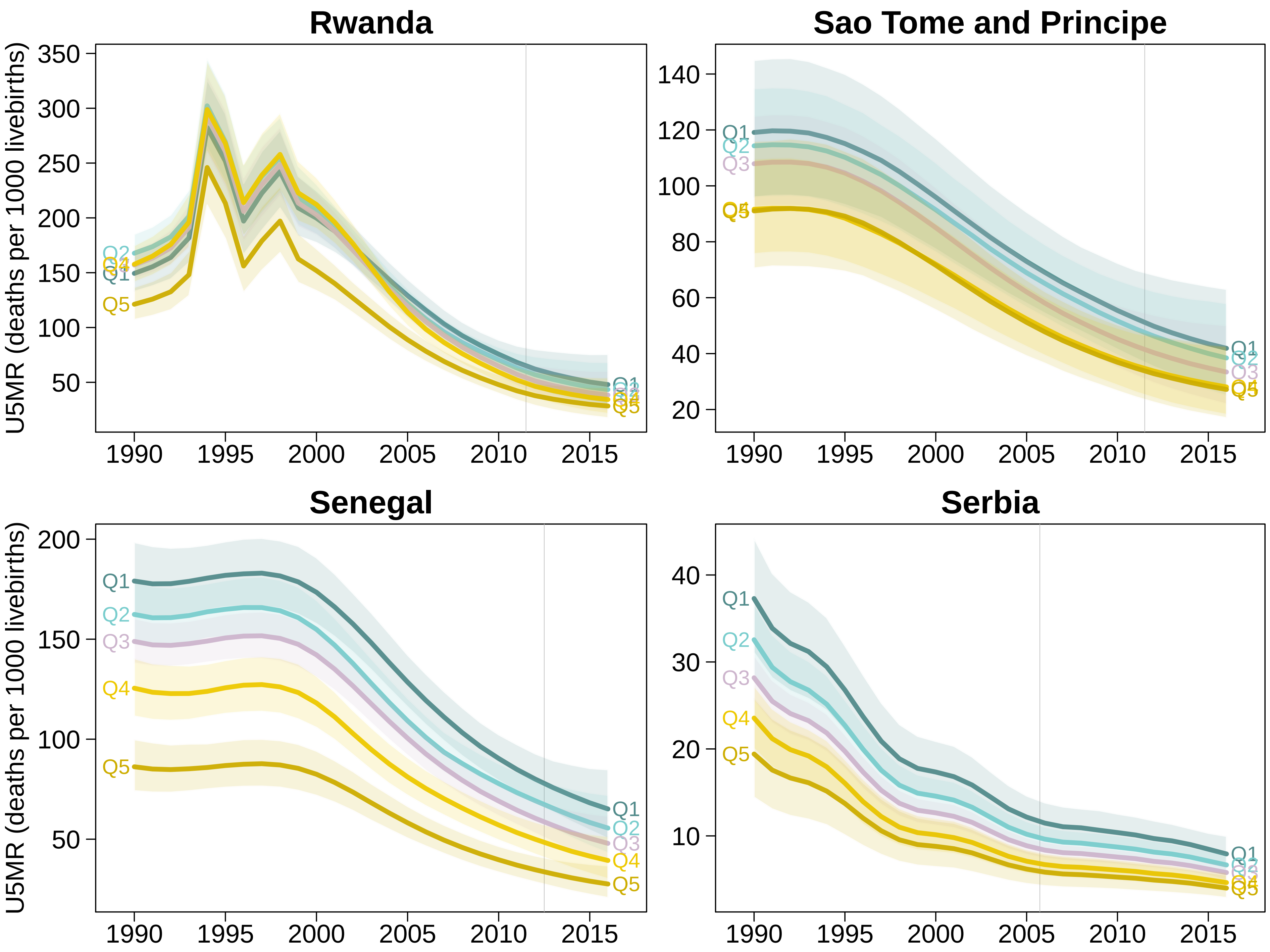

Rwanda
Sao Tome and Principe
Senegal
Serbia
U5MR (deaths per 1000 livebirths)
Q1
Q2
Q3
Q4
Q5
1990
1995
2000
2005
2010
2015

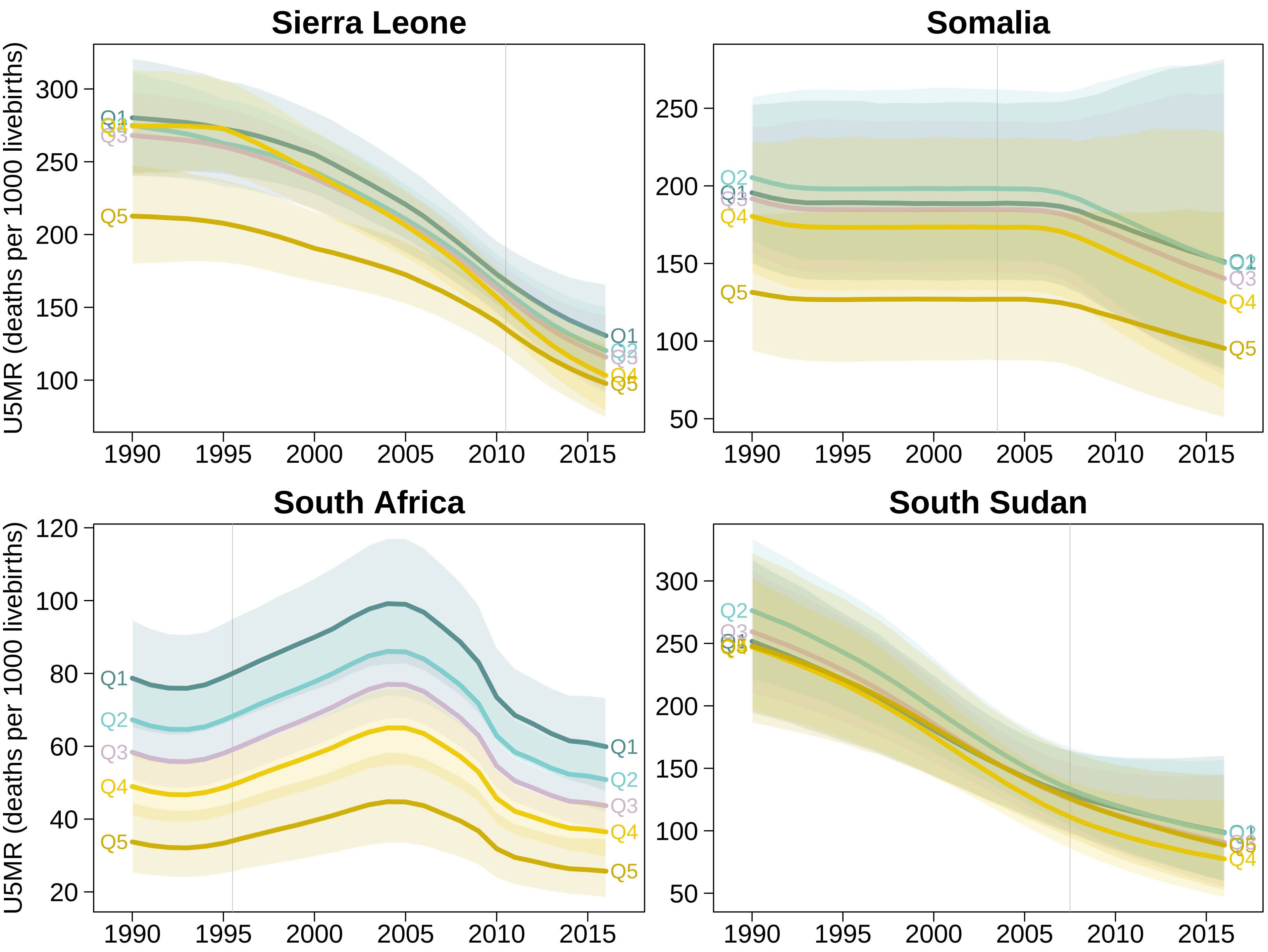
Sierra Leone
Somalia
South Africa
South Sudan
U5MR (deaths per 1000 livebirths)
U5MR (deaths per 1000 livebirths)
300
250
200
150
100
250
200
150
100
50
120
100
80
60
40
20
300
250
200
150
100
50
1990
1995
2000
2005
2010
2015
Q1
Q2
Q3
Q4
Q5

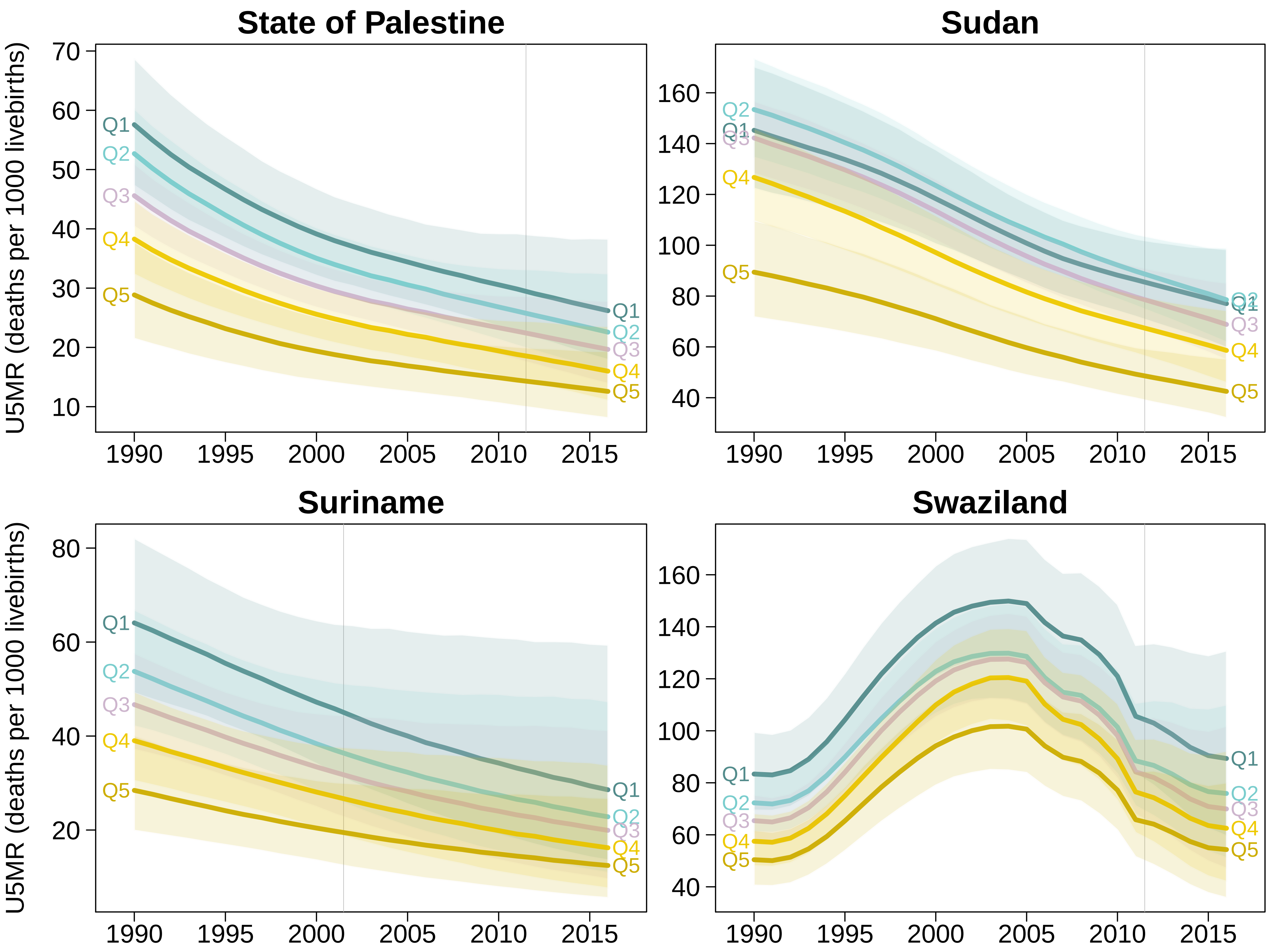
State of Palestine
Sudan
Suriname
Swaziland
U5MR (deaths per 1000 livebirths)
Q1
Q2
Q3
Q4
Q5
1990
1995
2000
2005
2010
2015

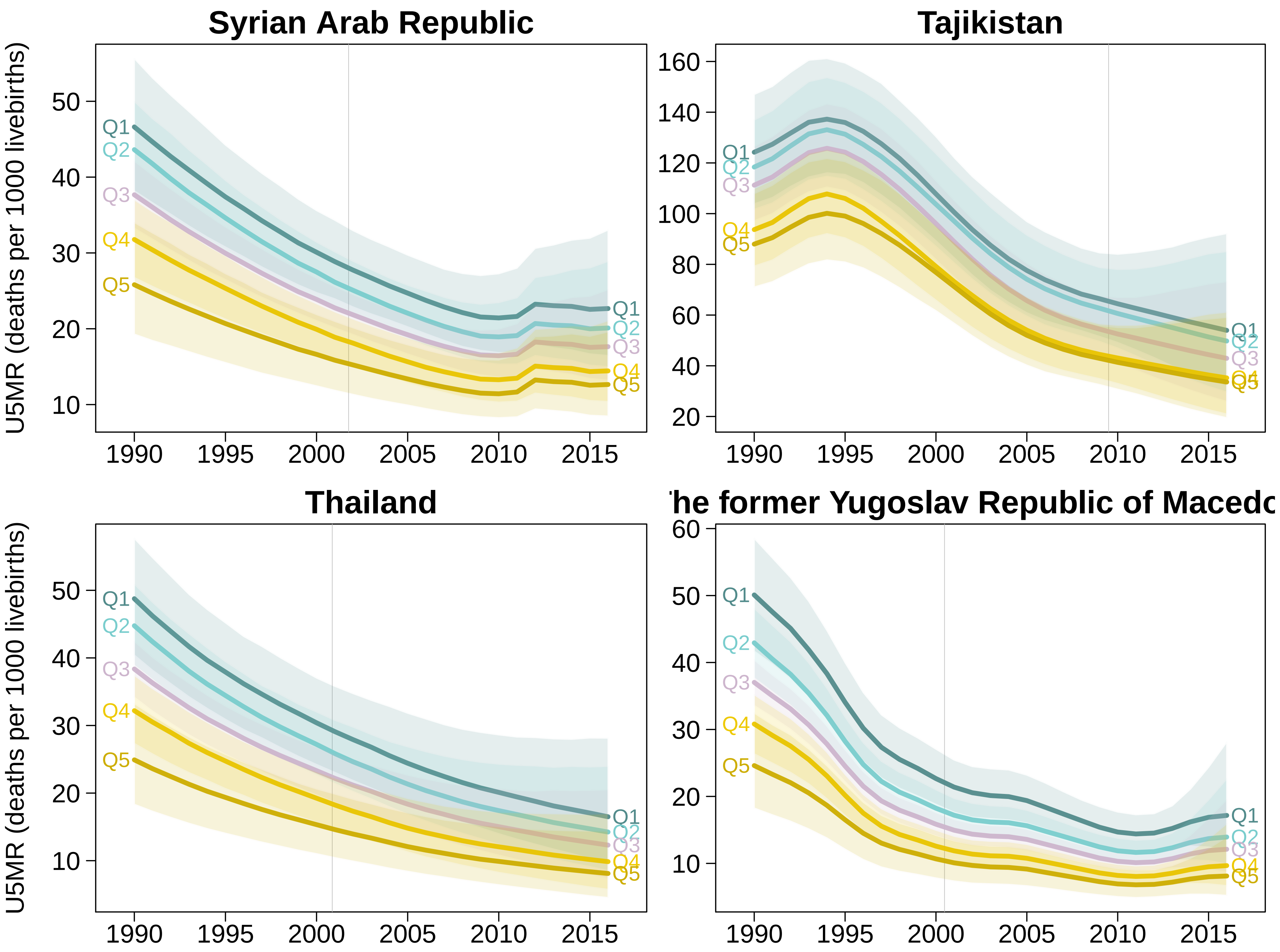
Syrian Arab Republic
Tajikistan
Thailand
he former Yugoslav Republic of Macedo
U5MR (deaths per 1000 livebirths)
Q1
Q2
Q3
Q4
Q5
1990
1995
2000
2005
2010
2015

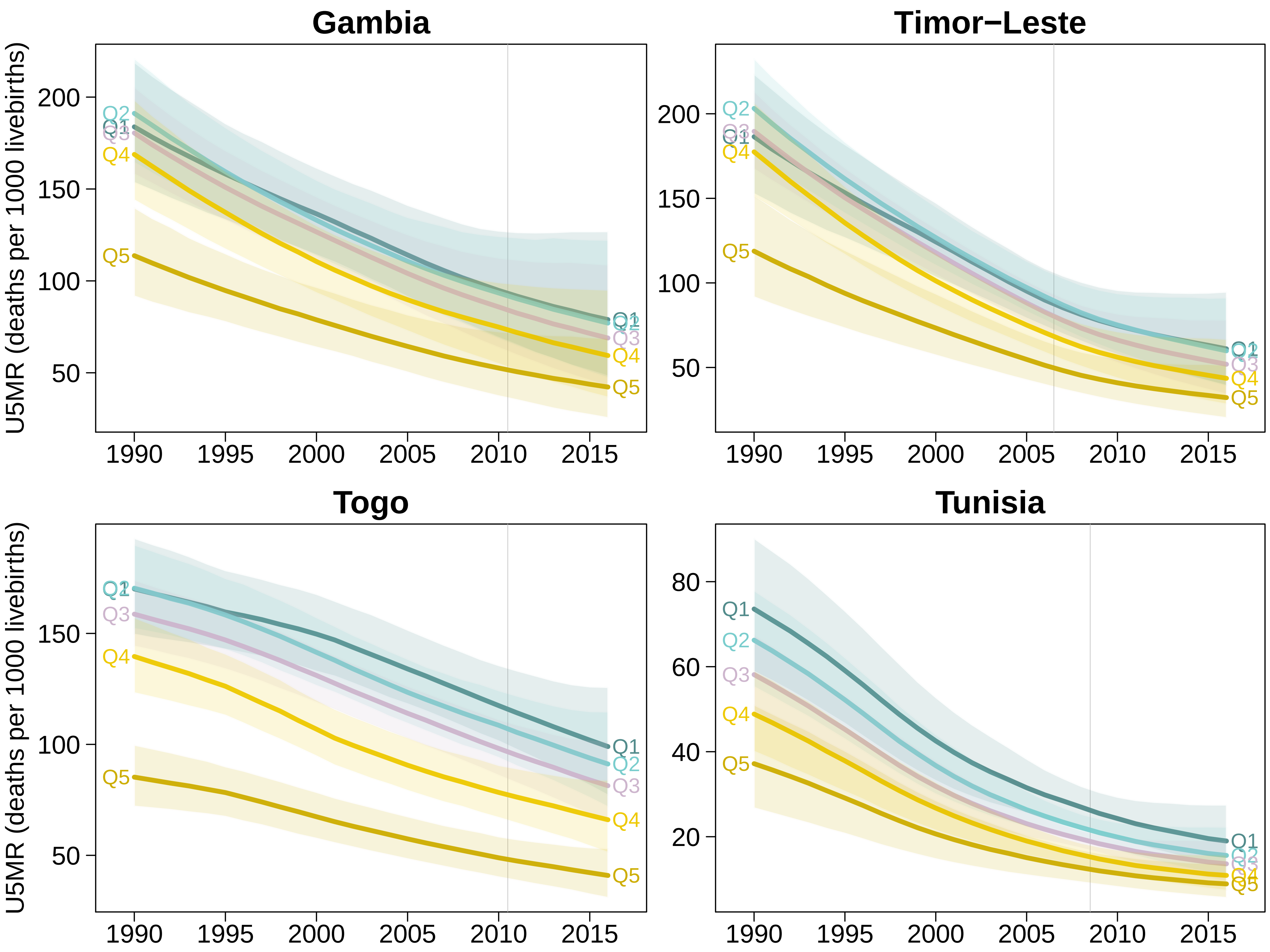
Gambia
Timor−Leste
Togo
Tunisia
U5MR (deaths per 1000 livebirths)
U5MR (deaths per 1000 livebirths)
Q1
Q2
Q3
Q4
Q5

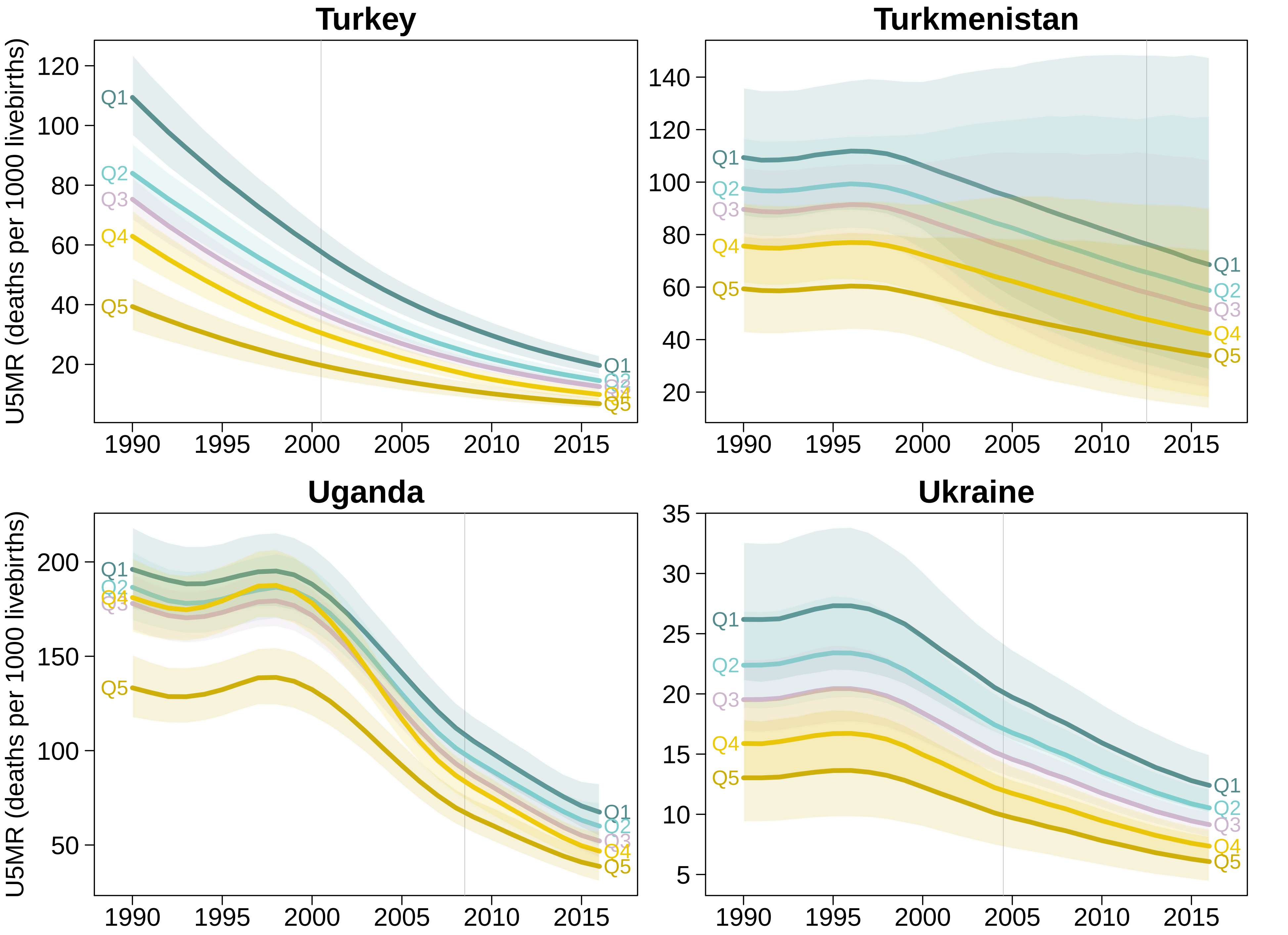
Turkey
Turkmenistan
Uganda
Ukraine
U5MR (deaths per 1000 livebirths)
Q1
Q2
Q3
Q4
Q5
1990
1995
2000
2005
2010
2015

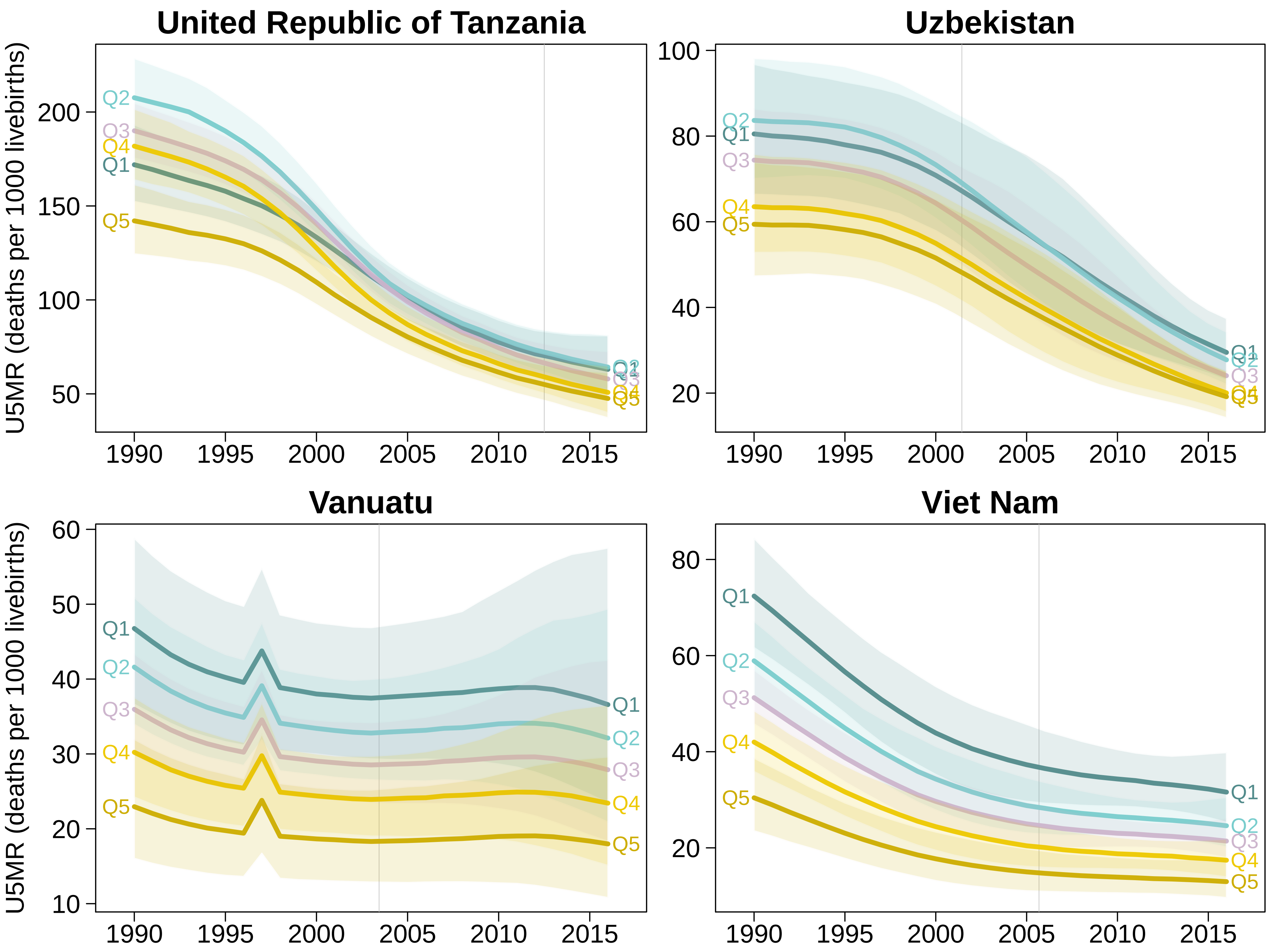

United Republic of Tanzania
Uzbekistan
Vanuatu
Viet Nam
U5MR (deaths per 1000 livebirths)
U5MR (deaths per 1000 livebirths)
Q1
Q2
Q3
Q4
Q5
1990
1995
2000
2005
2010
2015

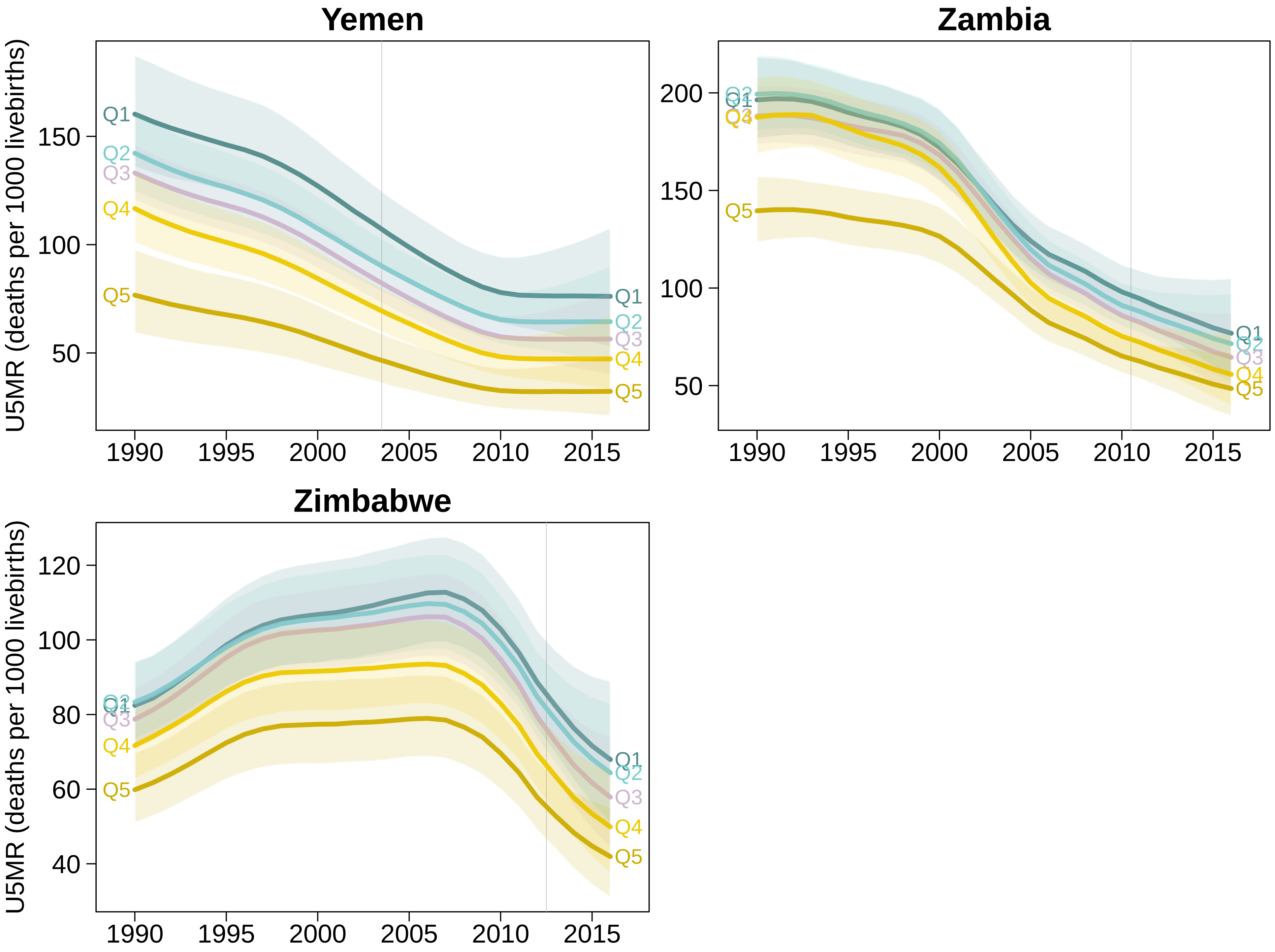
Yemen
U5MR (deaths per 1000 livebirths)
150
100
50
Q1
Q2
Q3
Q4
Q5
1990
1995
2000
2005
2010
2015
Zambia
200
150
100
50
Zimbabwe
120
100
80
60
40

Figure 10: **Ratio of U5MR in wealth quintile 1 (poorest) to wealth quintile 5 (richest), for the 99 countries with empirical data.** Solid curves are point estimates from the model. Shaded areas around the solid curves are the 90% uncertainty intervals. The maximum and minimum point estimates within each country are the values in blue. Vertical grey lines indicate the most recent reference year of data points for each country.

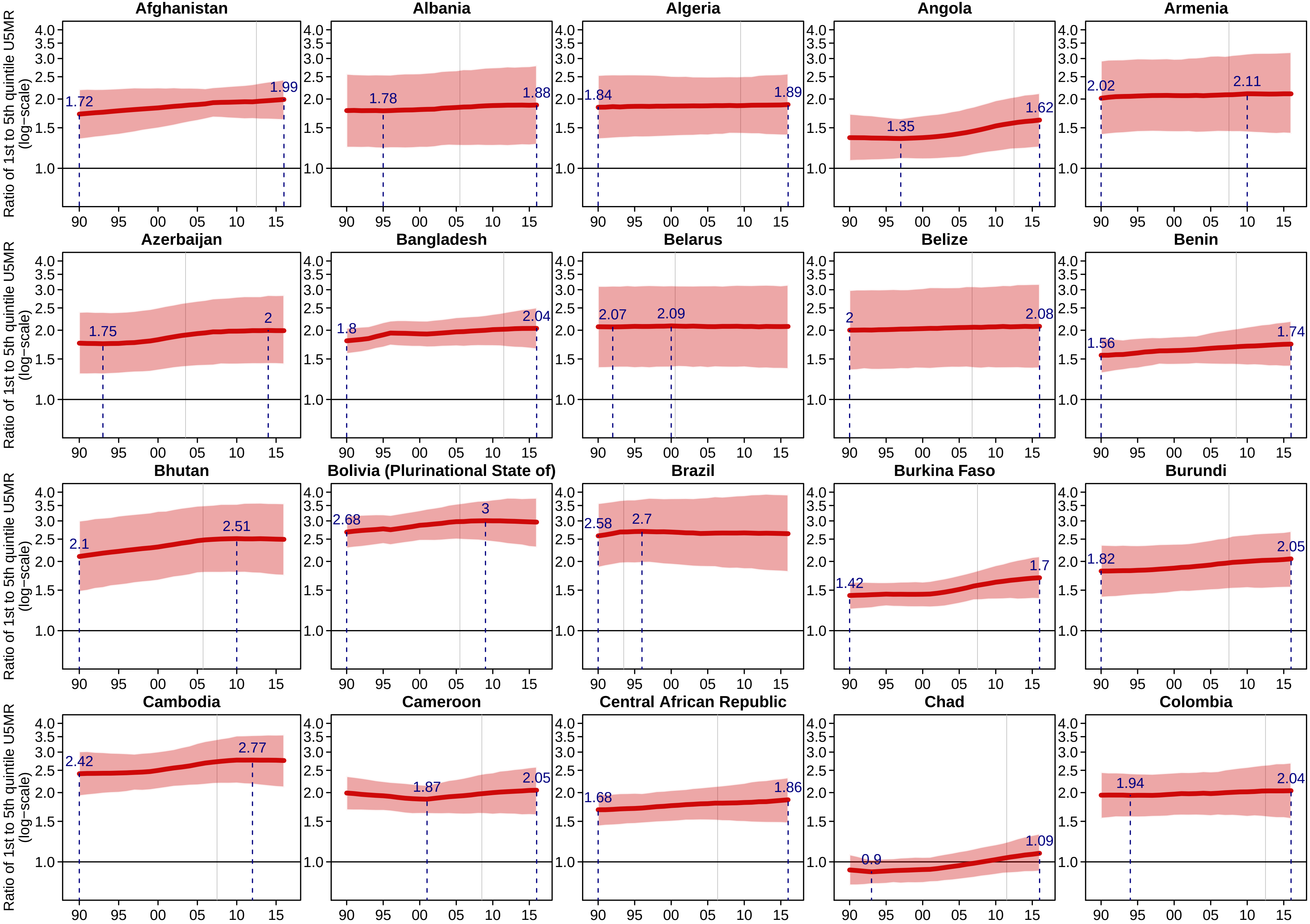
Ratio of 1st to 5th quintile U5MR (log−scale)
Afghanistan
1.72
1.99
Albania
1.78
1.88
Algeria
1.84
1.89
Angola
1.35
1.62
Armenia
2.02
2.11
Azerbaijan
1.75
2
Bangladesh
1.8
2.04
Belarus
2.07
2.09
Belize
2
2.08
Benin
1.56
1.74
Bhutan
2.1
2.51
Bolivia (Plurinational State of)
2.68
3
Brazil
2.58
2.7
Burkina Faso
1.42
1.7
Burundi
1.82
2.05
Cambodia
2.42
2.77
Cameroon
1.87
2.05
Central African Republic
1.68
1.86
Chad
0.9
1.09
Colombia
1.94
2.04
90 95 00 05 10 15
1.0 1.5 2.0 2.5 3.0 3.5 4.0

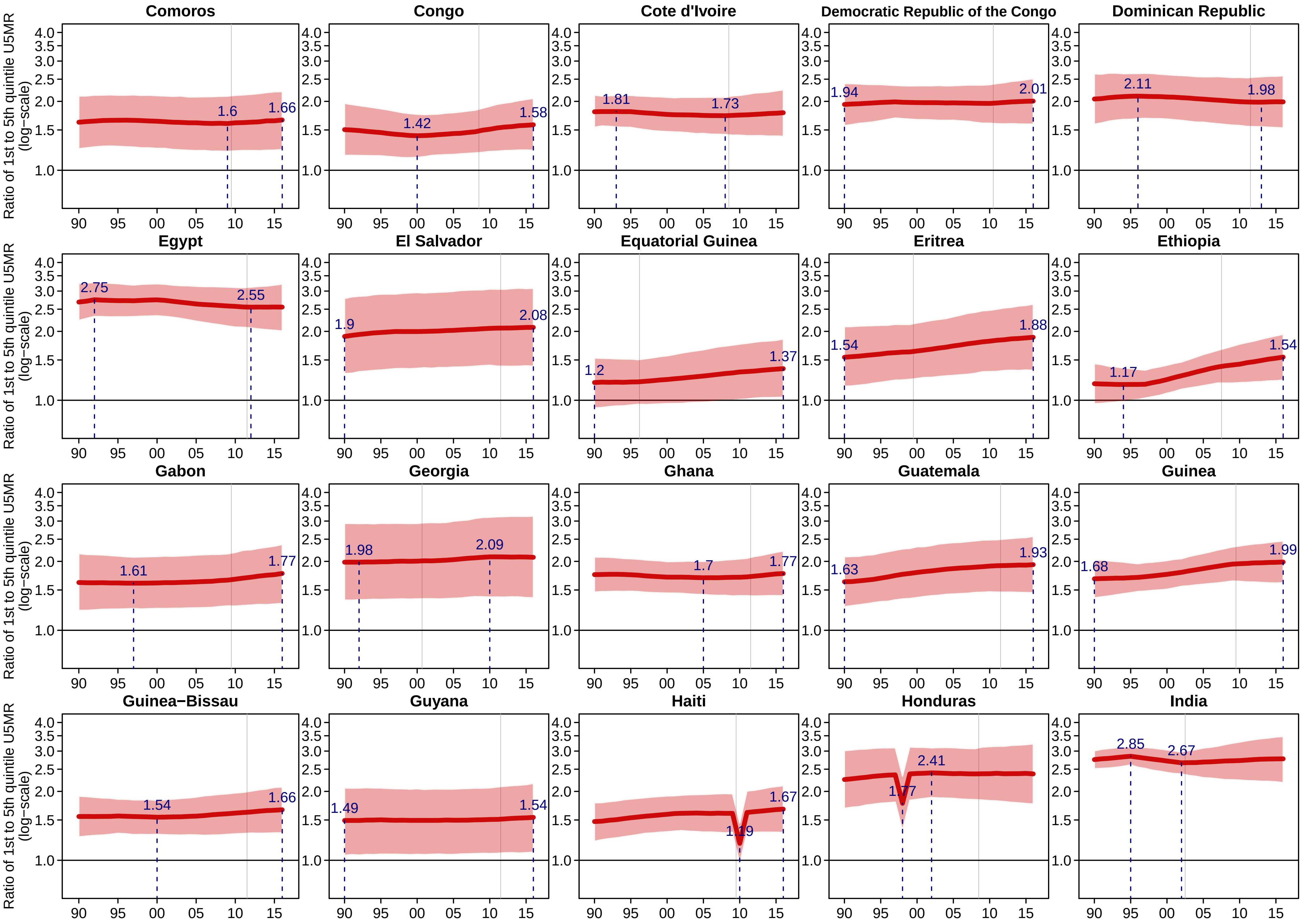

Comoros
Congo
Cote d'Ivoire
Democratic Republic of the Congo
Dominican Republic
Egypt
El Salvador
Equatorial Guinea
Eritrea
Ethiopia
Gabon
Georgia
Ghana
Guatemala
Guinea
Guinea−Bissau
Guyana
Haiti
Honduras
India
Ratio of 1st to 5th quintile U5MR (log−scale)
1.0
1.5
2.0
2.5
3.0
3.5
4.0
90
95
00
05
10
15
1.6
1.66
1.42
1.58
1.81
1.73
1.94
2.01
2.11
1.98
2.75
2.55
1.9
2.08
1.2
1.37
1.54
1.88
1.17
1.54
1.61
1.77
1.98
2.09
1.7
1.77
1.63
1.93
1.68
1.99
1.54
1.66
1.49
1.54
1.19
1.67
1.77
2.41
2.85
2.67

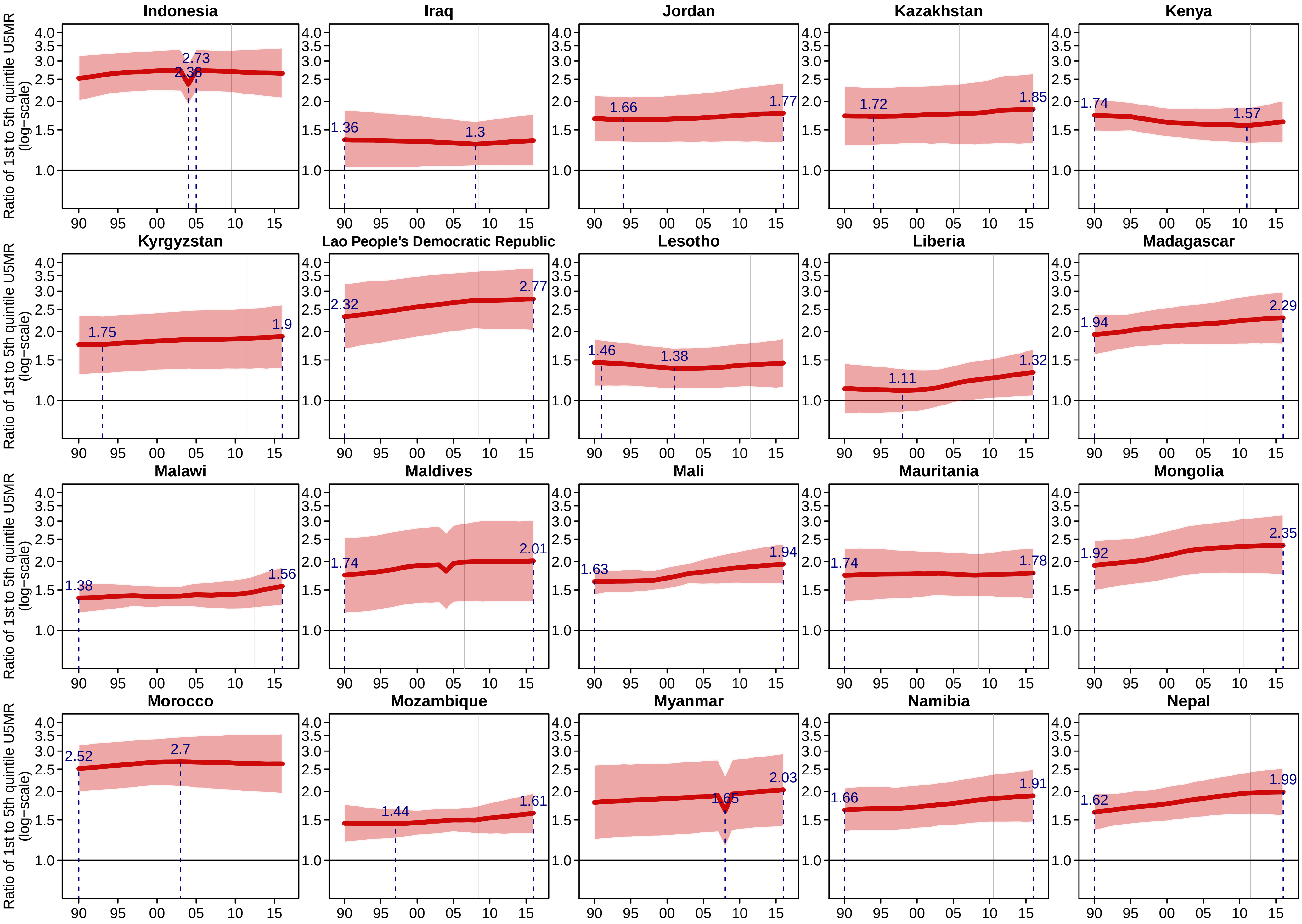
Ratio of 1st to 5th quintile U5MR (log−scale)
Indonesia
2.73
2.38
Iraq
1.36
1.3
Jordan
1.66
1.77
Kazakhstan
1.72
1.85
Kenya
1.74
1.57
Kyrgyzstan
1.75
1.9
Lao People's Democratic Republic
2.32
2.77
Lesotho
1.46
1.38
Liberia
1.11
1.32
Madagascar
1.94
2.29
Malawi
1.38
1.56
Maldives
1.74
2.01
Mali
1.63
1.94
Mauritania
1.74
1.78
Mongolia
1.92
2.35
Morocco
2.52
2.7
Mozambique
1.44
1.61
Myanmar
1.65
2.03
Namibia
1.66
1.91
Nepal
1.62
1.99
4.0
3.5
3.0
2.5
2.0
1.5
1.0
90
95
00
05
10
15

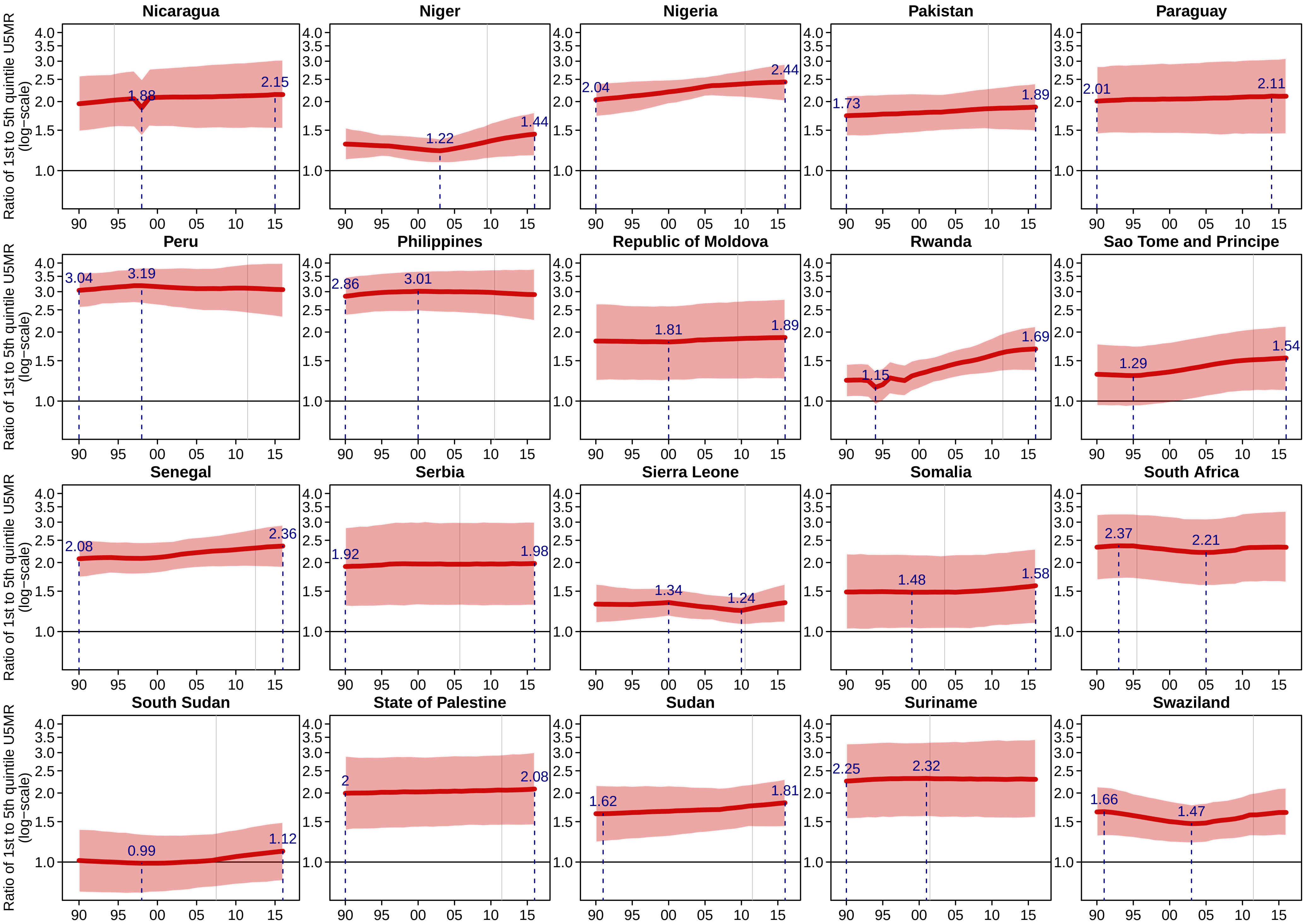
Nicaragua
Niger
Nigeria
Pakistan
Paraguay
Peru
Philippines
Republic of Moldova
Rwanda
Sao Tome and Principe
Senegal
Serbia
Sierra Leone
Somalia
South Africa
South Sudan
State of Palestine
Sudan
Suriname
Swaziland
Ratio of 1st to 5th quintile U5MR (log−scale)
1.88
2.15
1.22
1.44
2.04
2.44
1.73
1.89
2.01
2.11
3.04
3.19
2.86
3.01
1.81
1.89
1.15
1.69
1.29
1.54
2.08
2.36
1.92
1.98
1.34
1.24
1.48
1.58
2.37
2.21
0.99
1.12
2
2.08
1.62
1.81
2.25
2.32
1.66
1.47

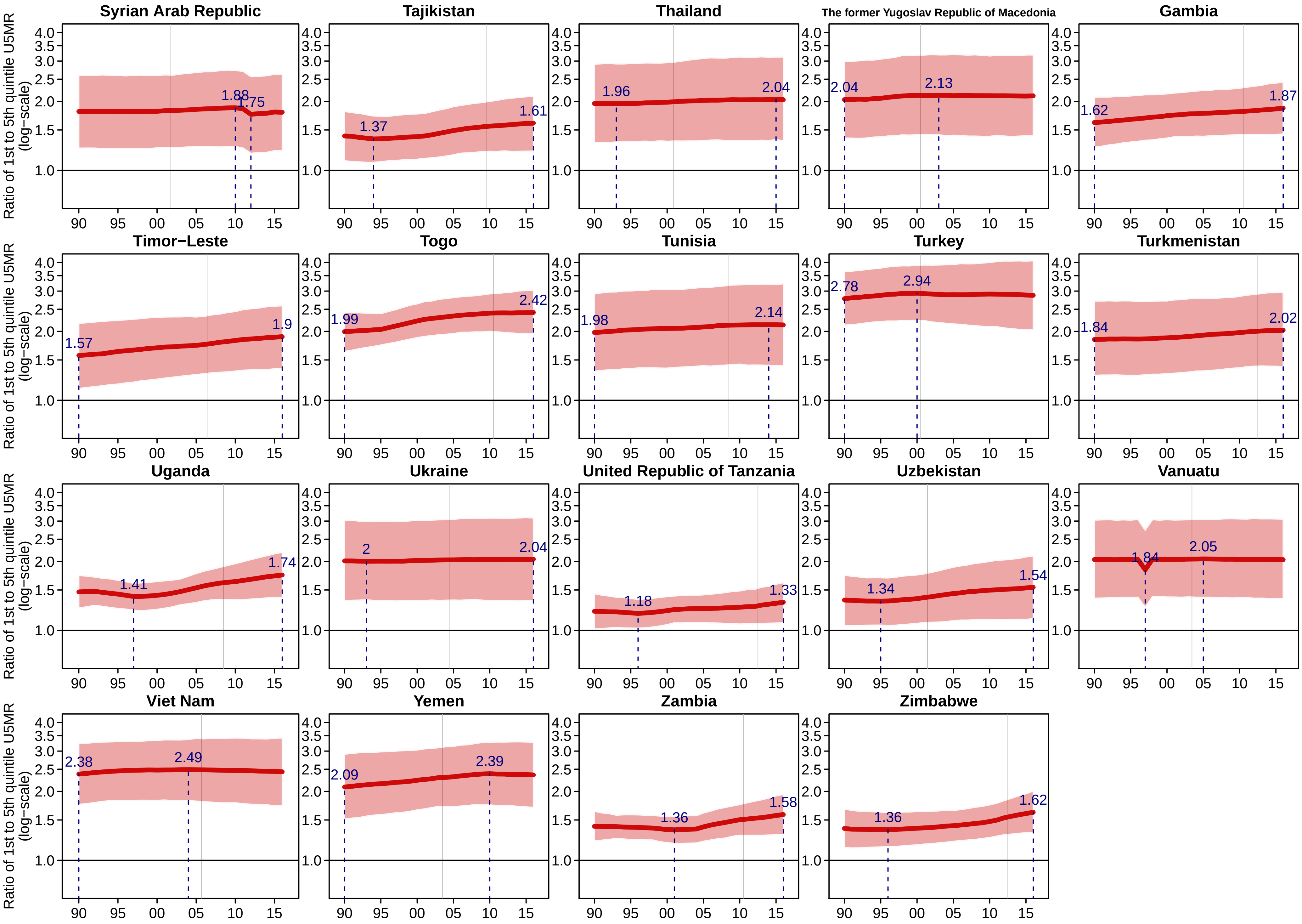

Syrian Arab Republic
Tajikistan
Thailand
The former Yugoslav Republic of Macedonia
Gambia
Timor−Leste
Togo
Tunisia
Turkey
Turkmenistan
Uganda
Ukraine
United Republic of Tanzania
Uzbekistan
Vanuatu
Viet Nam
Yemen
Zambia
Zimbabwe
Ratio of 1st to 5th quintile U5MR (log−scale)

Figure 11: **All wealth quintile-specific results, for the 99 countries with empirical data.** 1st row: (i) wealth quintile-specific U5MR (blue); (ii) the point estimates of national-level U5MR from IGME 2017 results (black). 2nd row: number of wealth quintile-specific under-5 deaths. 3rd row (all on log-scale): (i) estimated ratio of wealth quintile-specific U5MR to national-level U5MR (red); (ii) expected ratio of wealth quintile-specific U5MR to national-level U5MR (green); (iii) colored dots refer to the ratio of wealth quintile-specific U5MR from survey to national-level U5MR from survey (these dots are the input data in our data model); (iv) vertical line segments around the dots are sampling errors and different colors differentiate data series. 4th row: percentage of wealth quintile-specific under-5 deaths among national-level under-5 deaths (in %). Results for wealth quintile groups 1 (the poorest) to 5 (the richest) are displayed in the five columns respectively. Solid curves are point estimates from the model. Shaded areas around the solid curves are the 90% uncertainty intervals. Vertical grey lines indicate the most recent reference year of data points for each country.

Afghanistan

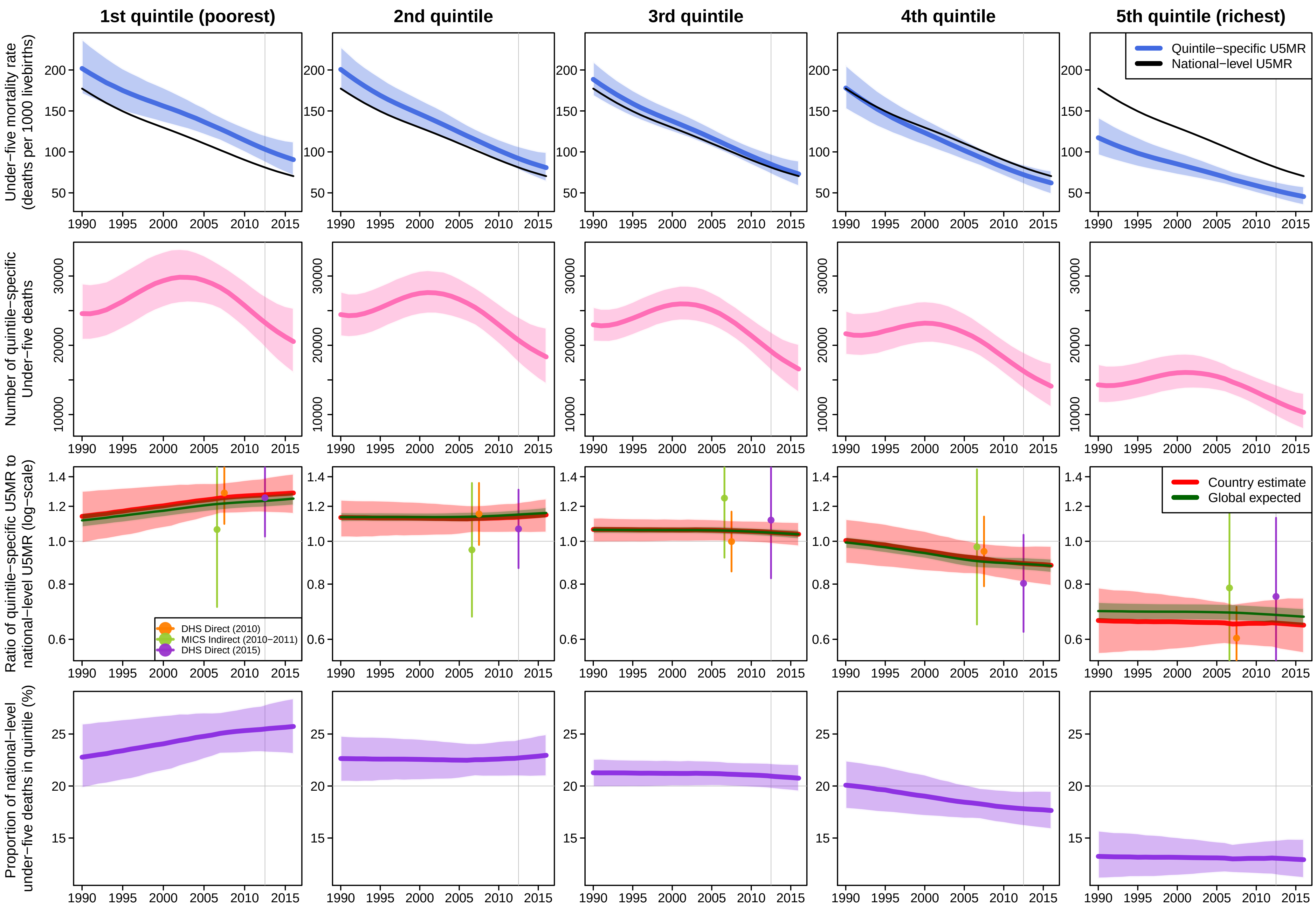

Albania

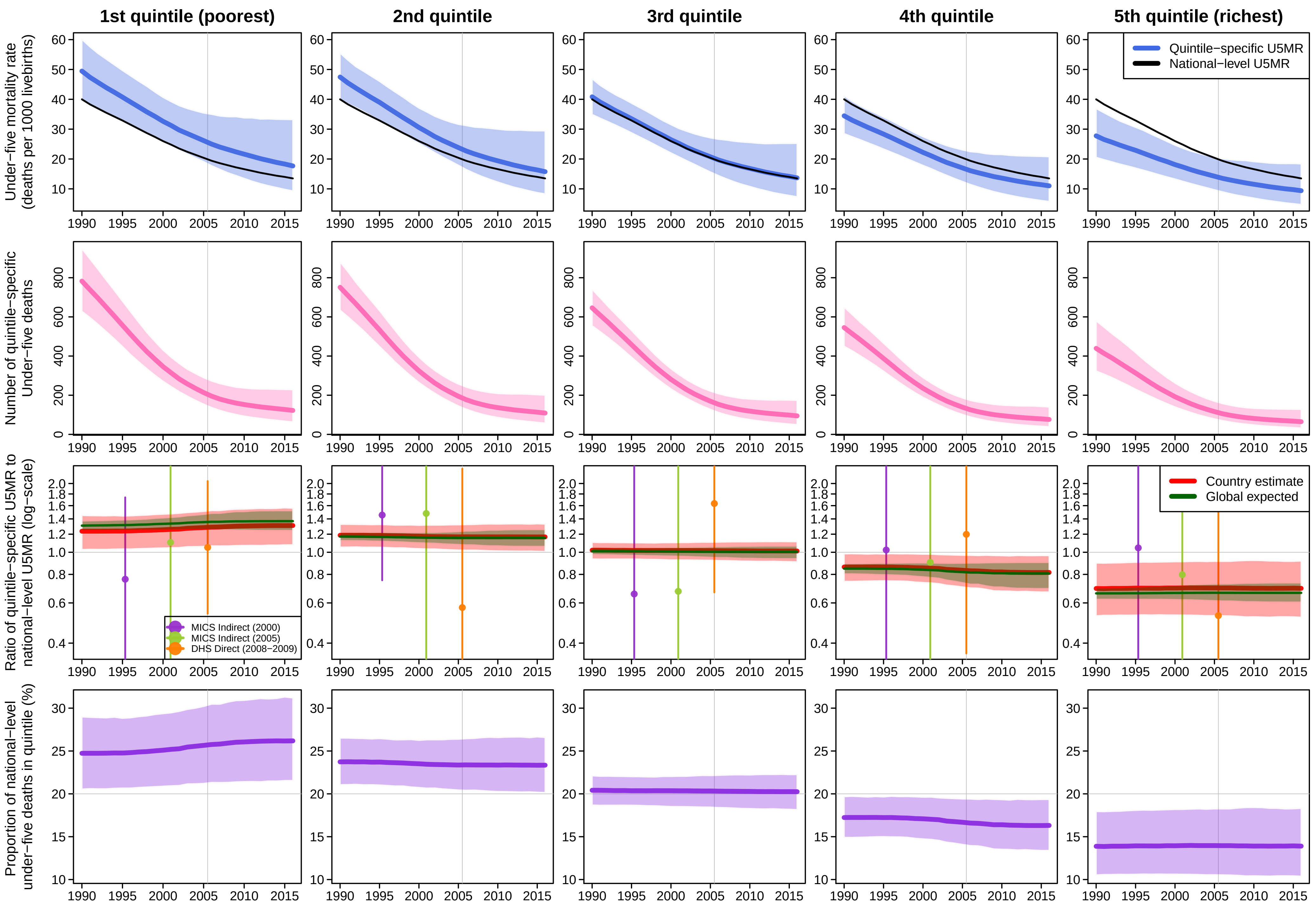

1st quintile (poorest)
2nd quintile
3rd quintile
4th quintile
5th quintile (richest)
Under−five mortality rate (deaths per 1000 livebirths)
Number of quintile−specific Under−five deaths
Ratio of quintile−specific U5MR to national−level U5MR (log−scale)
Proportion of national−level under−five deaths in quintile (%)
Quintile−specific U5MR
National−level U5MR
Country estimate
Global expected
MICS Indirect (2000)
MICS Indirect (2005)
DHS Direct (2008−2009)

Algeria

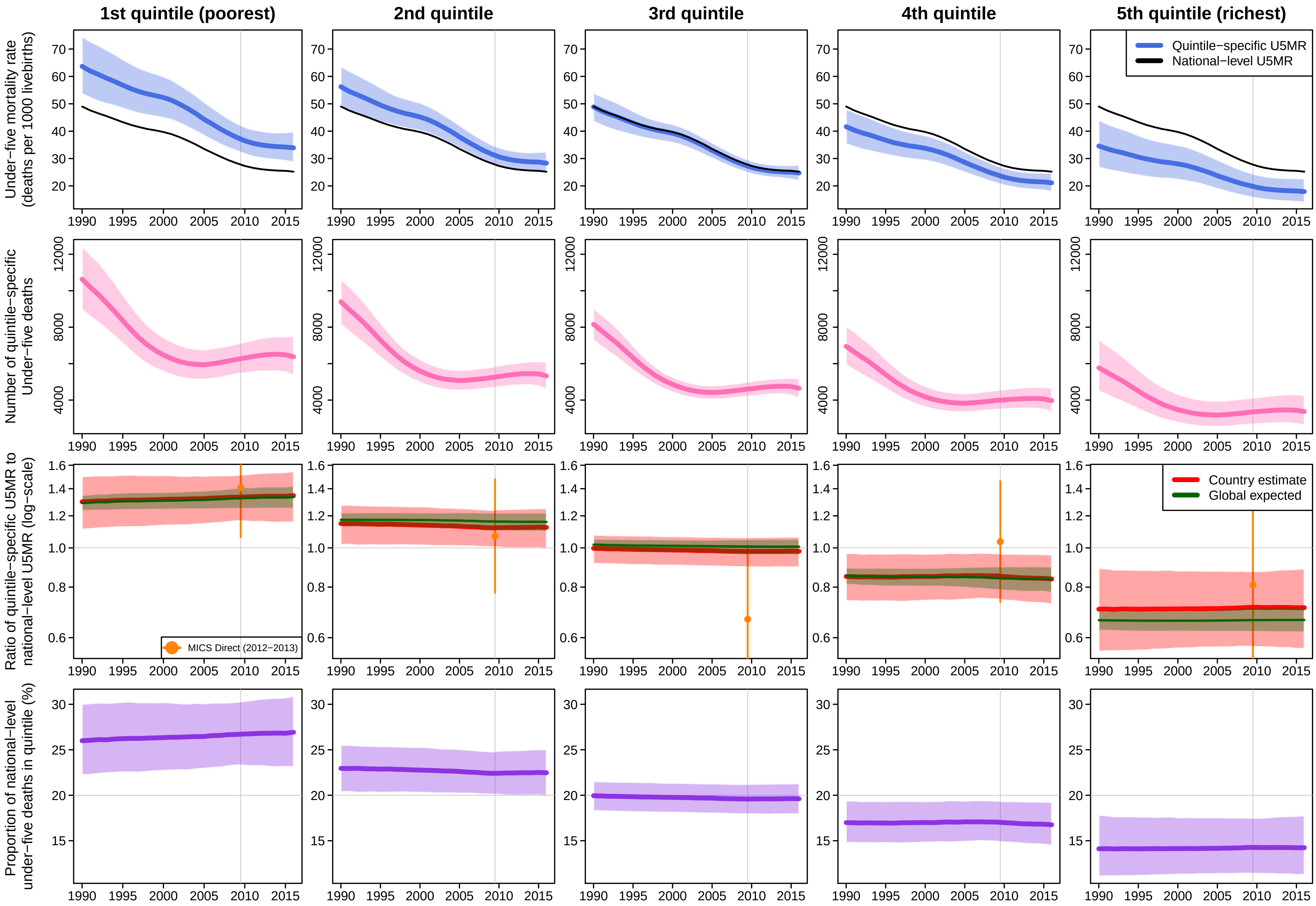
1st quintile (poorest)
2nd quintile
3rd quintile
4th quintile
5th quintile (richest)
Under−five mortality rate (deaths per 1000 livebirths)
Quintile−specific U5MR
National−level U5MR
Number of quintile−specific Under−five deaths
Ratio of quintile−specific U5MR to national−level U5MR (log−scale)
MICS Direct (2012−2013)
Country estimate
Global expected
Proportion of national−level under−five deaths in quintile (%)

Angola

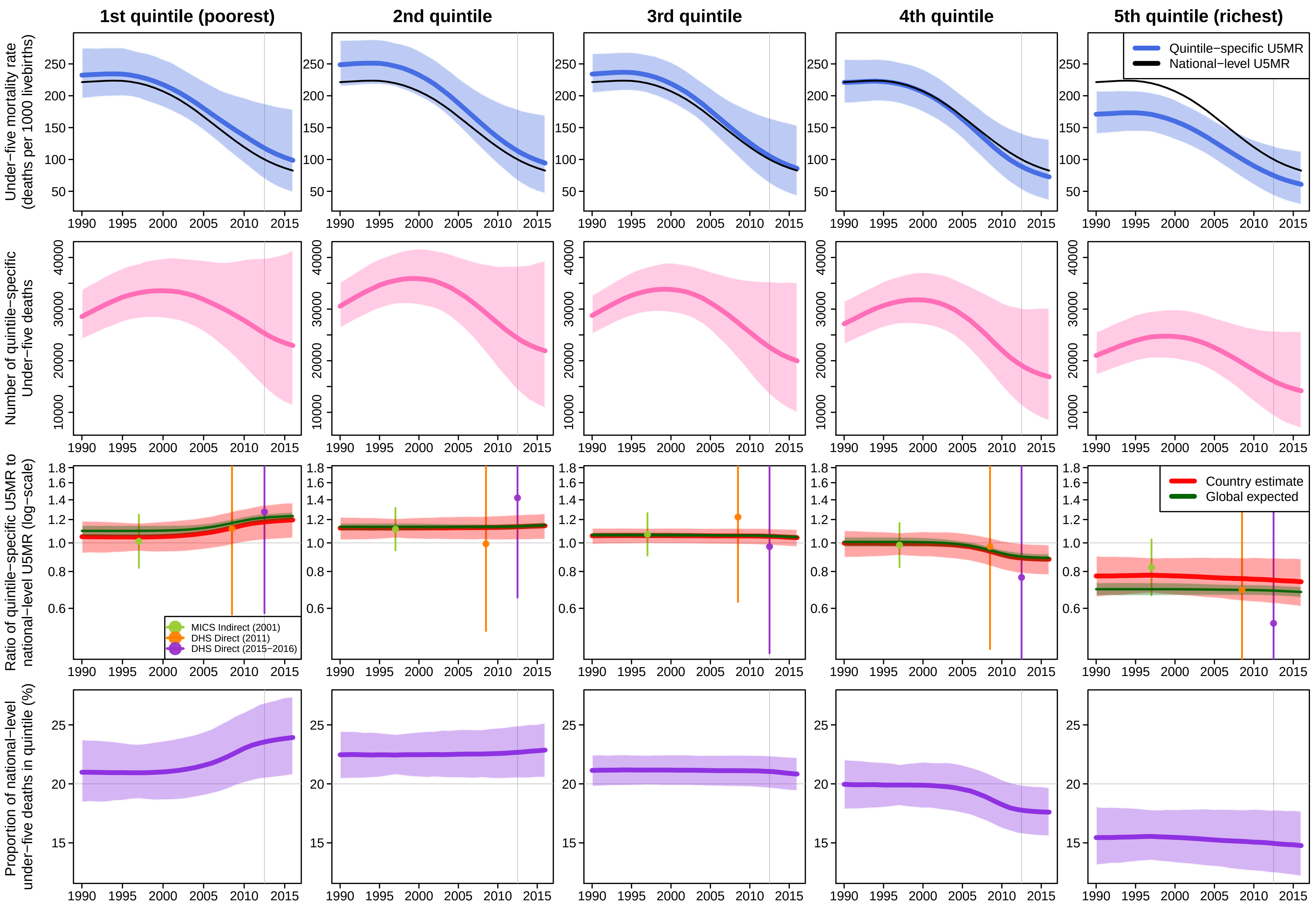
1st quintile (poorest)
2nd quintile
3rd quintile
4th quintile
5th quintile (richest)
Under−five mortality rate (deaths per 1000 livebirths)
Quintile−specific U5MR
National−level U5MR
Number of quintile−specific Under−five deaths
Ratio of quintile−specific U5MR to national−level U5MR (log−scale)
Country estimate
Global expected
MICS Indirect (2001)
DHS Direct (2011)
DHS Direct (2015−2016)
Proportion of national−level under−five deaths in quintile (%)

Armenia

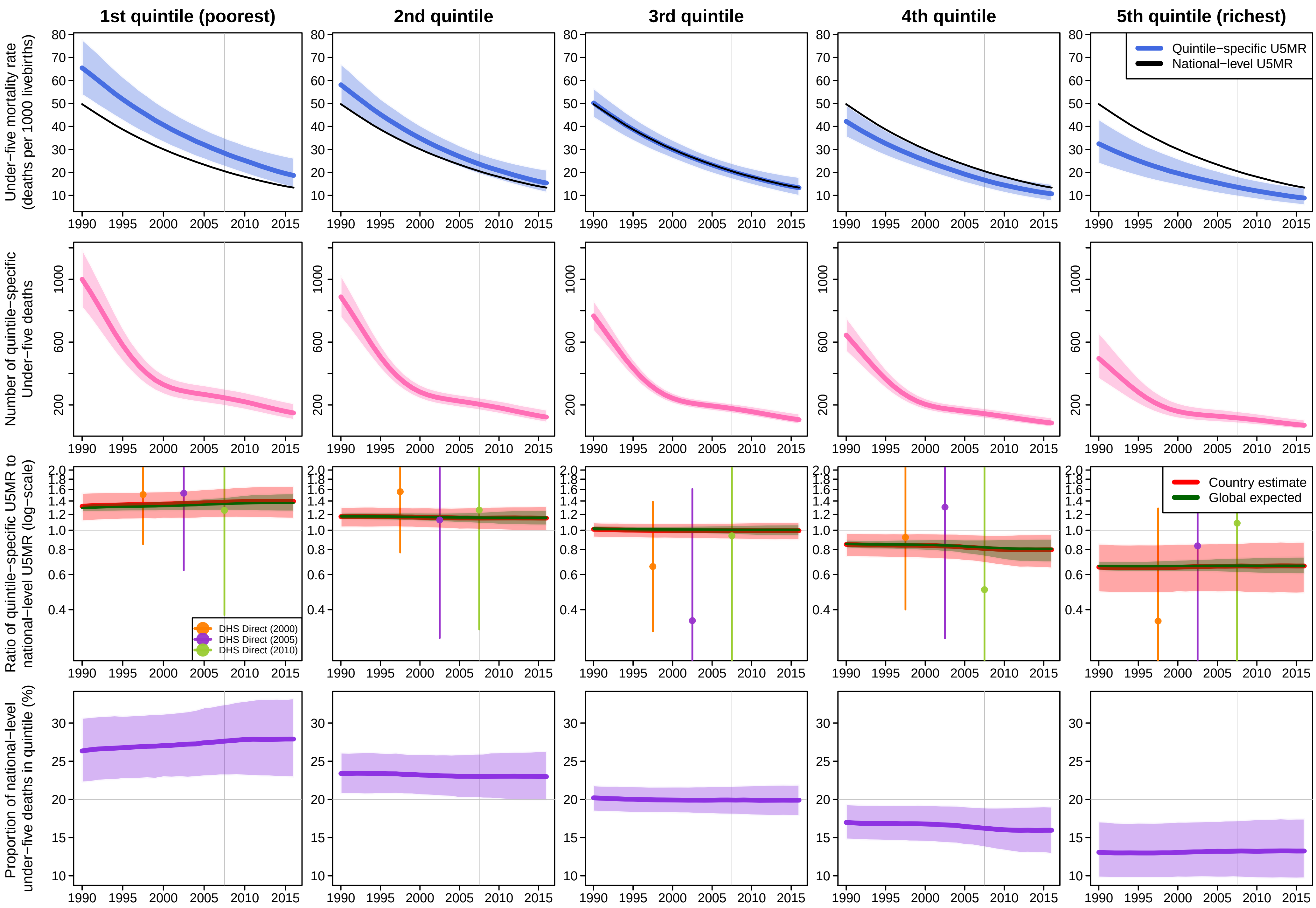

1st quintile (poorest)
2nd quintile
3rd quintile
4th quintile
5th quintile (richest)
Under−five mortality rate (deaths per 1000 livebirths)
Quintile−specific U5MR
National−level U5MR
Number of quintile−specific Under−five deaths
Ratio of quintile−specific U5MR to national−level U5MR (log−scale)
Country estimate
Global expected
DHS Direct (2000)
DHS Direct (2005)
DHS Direct (2010)
Proportion of national−level under−five deaths in quintile (%)

Azerbaijan

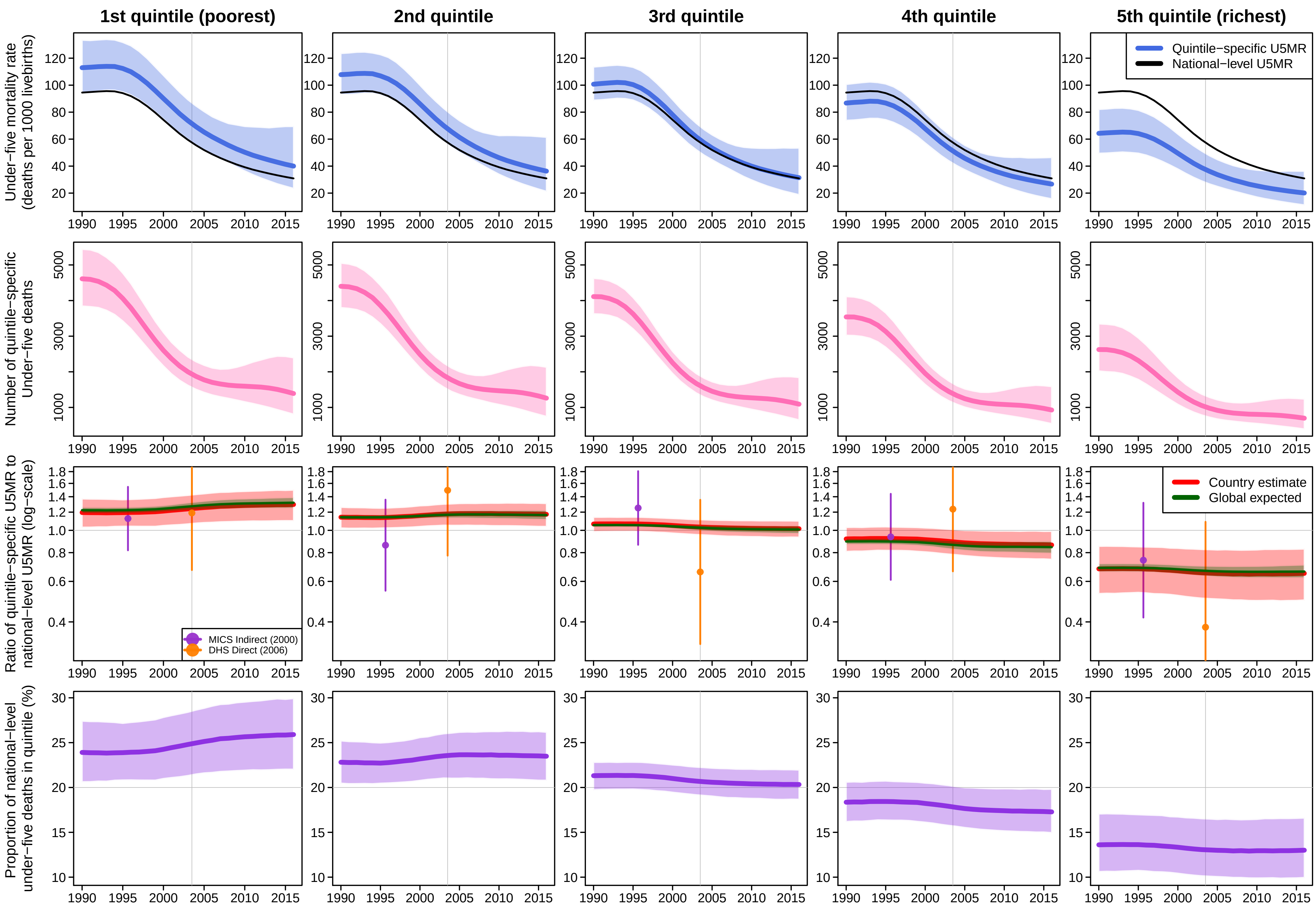

1st quintile (poorest)
2nd quintile
3rd quintile
4th quintile
5th quintile (richest)
Under−five mortality rate (deaths per 1000 livebirths)
Quintile−specific U5MR
National−level U5MR
Number of quintile−specific Under−five deaths
Ratio of quintile−specific U5MR to national−level U5MR (log−scale)
Country estimate
Global expected
MICS Indirect (2000)
DHS Direct (2006)
Proportion of national−level under−five deaths in quintile (%)

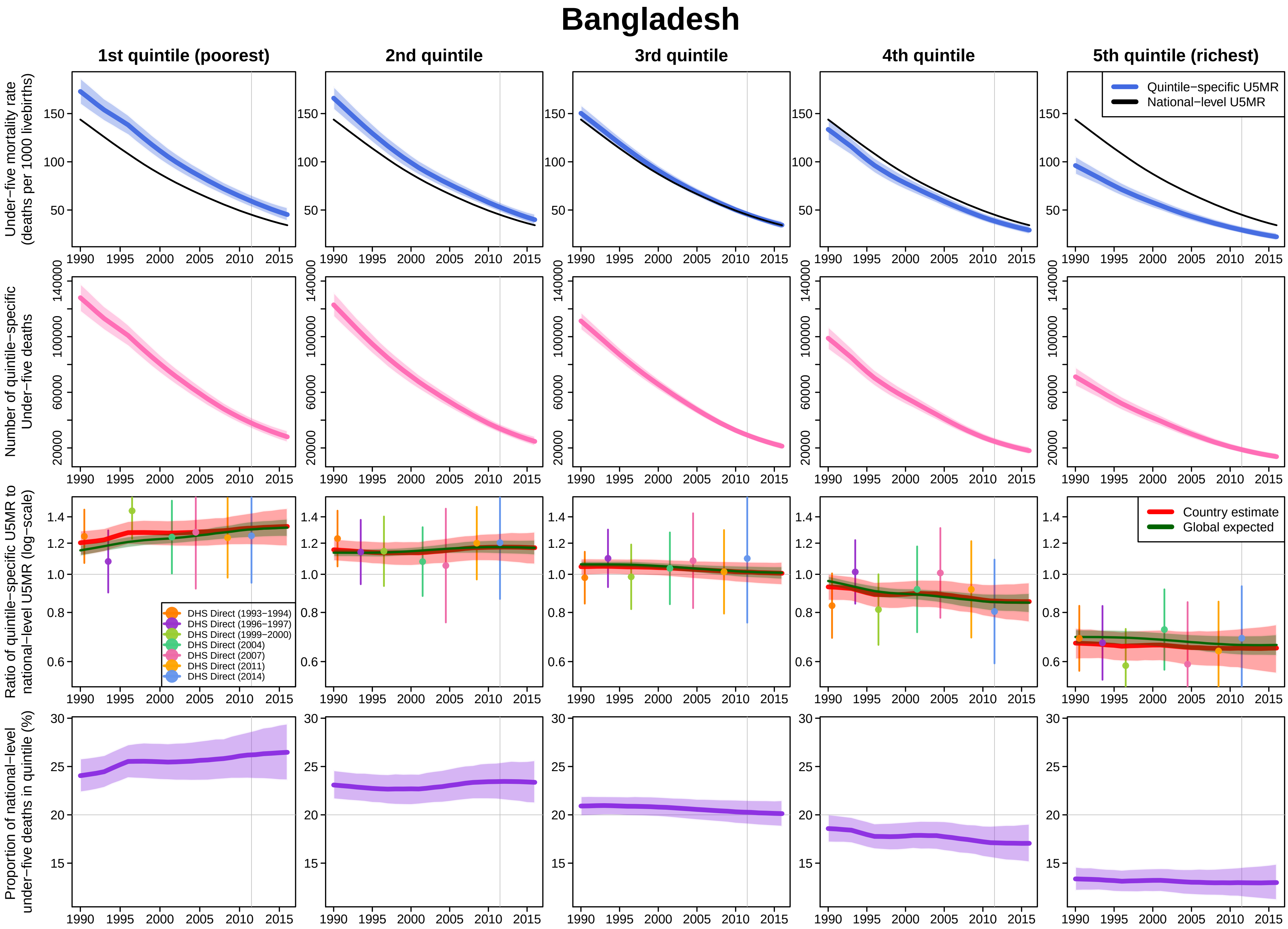
Bangladesh
1st quintile (poorest)
2nd quintile
3rd quintile
4th quintile
5th quintile (richest)
Under−five mortality rate
(deaths per 1000 livebirths)
Quintile−specific U5MR
National−level U5MR
Number of quintile−specific
Under−five deaths
Ratio of quintile−specific U5MR to
national−level U5MR (log−scale)
Country estimate
Global expected
DHS Direct (1993−1994)
DHS Direct (1996−1997)
DHS Direct (1999−2000)
DHS Direct (2004)
DHS Direct (2007)
DHS Direct (2011)
DHS Direct (2014)
Proportion of national−level
under−five deaths in quintile (%)

Belarus

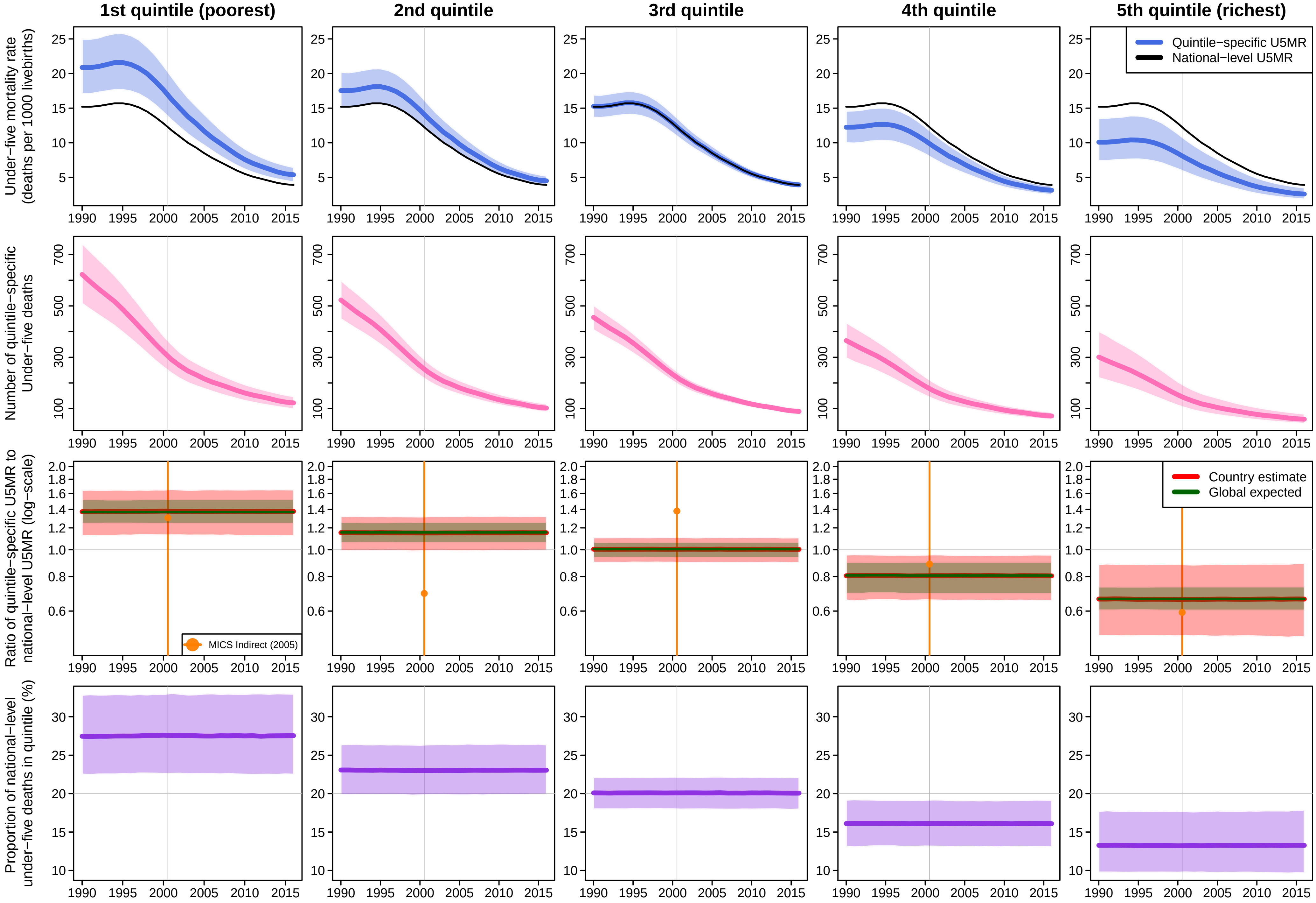
1st quintile (poorest)
2nd quintile
3rd quintile
4th quintile
5th quintile (richest)
Under−five mortality rate (deaths per 1000 livebirths)
Quintile−specific U5MR
National−level U5MR
Number of quintile−specific Under−five deaths
Ratio of quintile−specific U5MR to national−level U5MR (log−scale)
Country estimate
Global expected
MICS Indirect (2005)
Proportion of national−level under−five deaths in quintile (%)

Belize

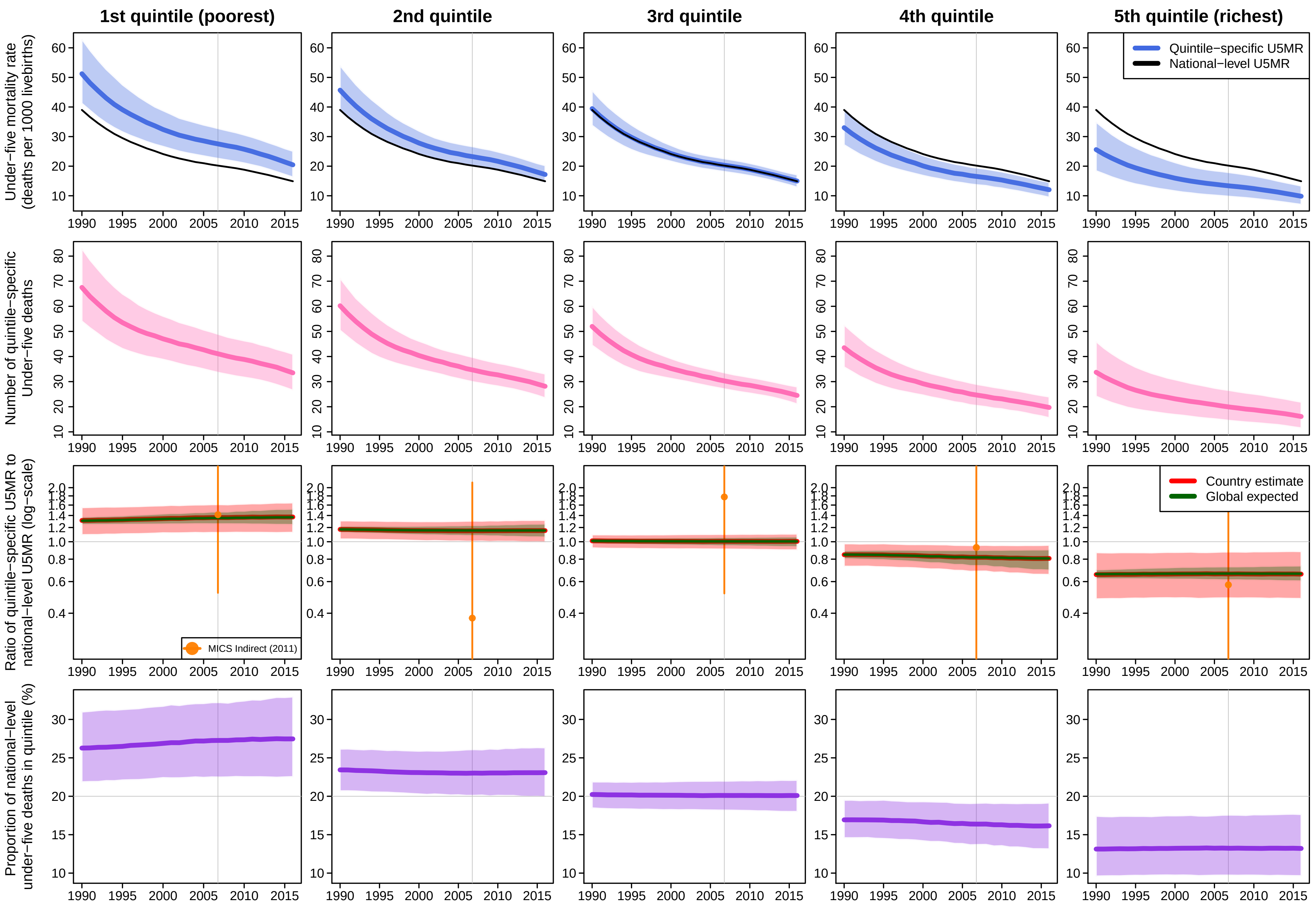
1st quintile (poorest)
2nd quintile
3rd quintile
4th quintile
5th quintile (richest)
Under−five mortality rate (deaths per 1000 livebirths)
Number of quintile−specific Under−five deaths
Ratio of quintile−specific U5MR to national−level U5MR (log−scale)
Proportion of national−level under−five deaths in quintile (%)
Quintile−specific U5MR
National−level U5MR
Country estimate
Global expected
MICS Indirect (2011)
1990
1995
2000
2005
2010
2015

Benin

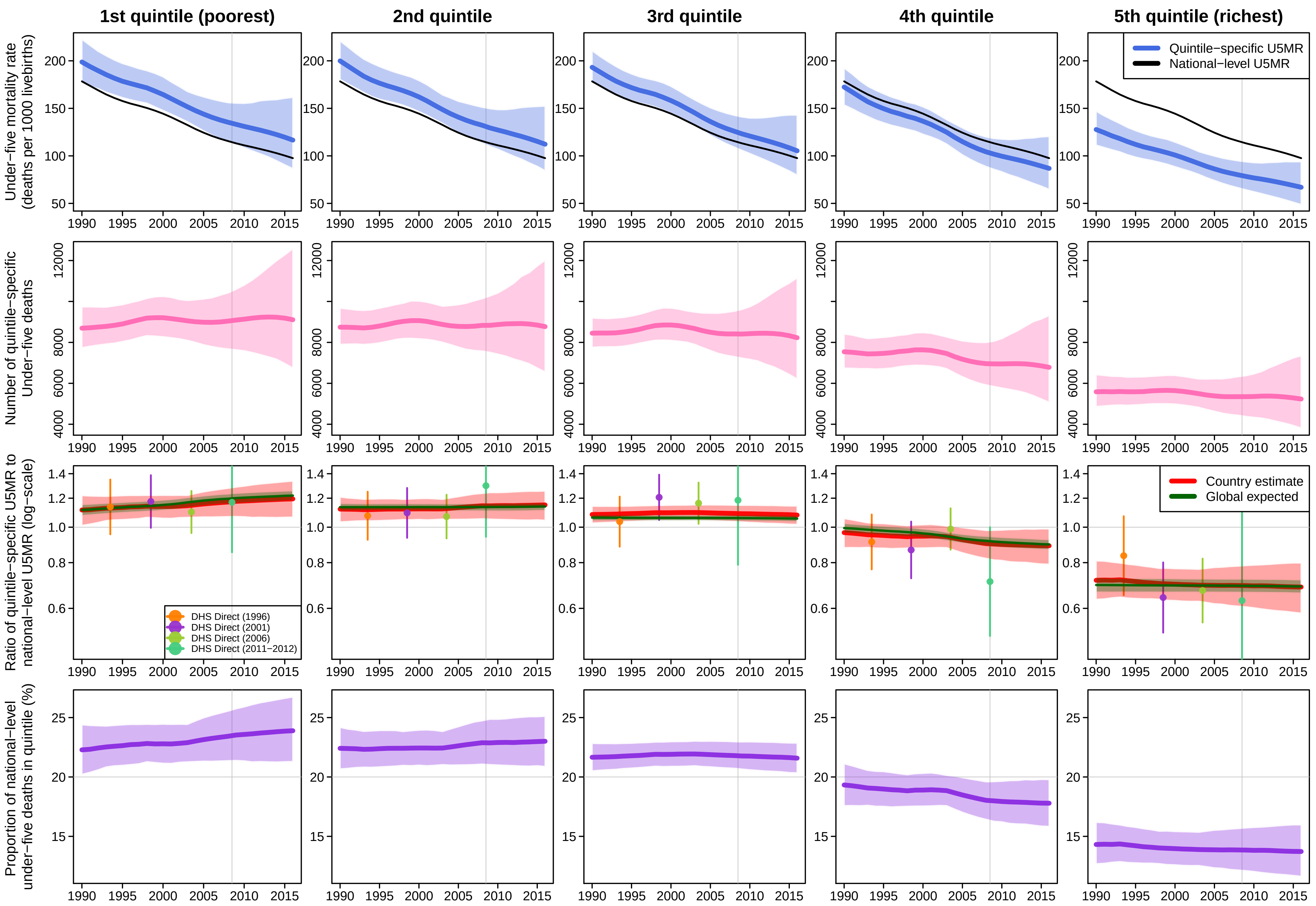

1st quintile (poorest)
2nd quintile
3rd quintile
4th quintile
5th quintile (richest)
Under−five mortality rate (deaths per 1000 livebirths)
Quintile−specific U5MR
National−level U5MR
Number of quintile−specific Under−five deaths
Ratio of quintile−specific U5MR to national−level U5MR (log−scale)
Country estimate
Global expected
DHS Direct (1996)
DHS Direct (2001)
DHS Direct (2006)
DHS Direct (2011−2012)
Proportion of national−level under−five deaths in quintile (%)

Bhutan

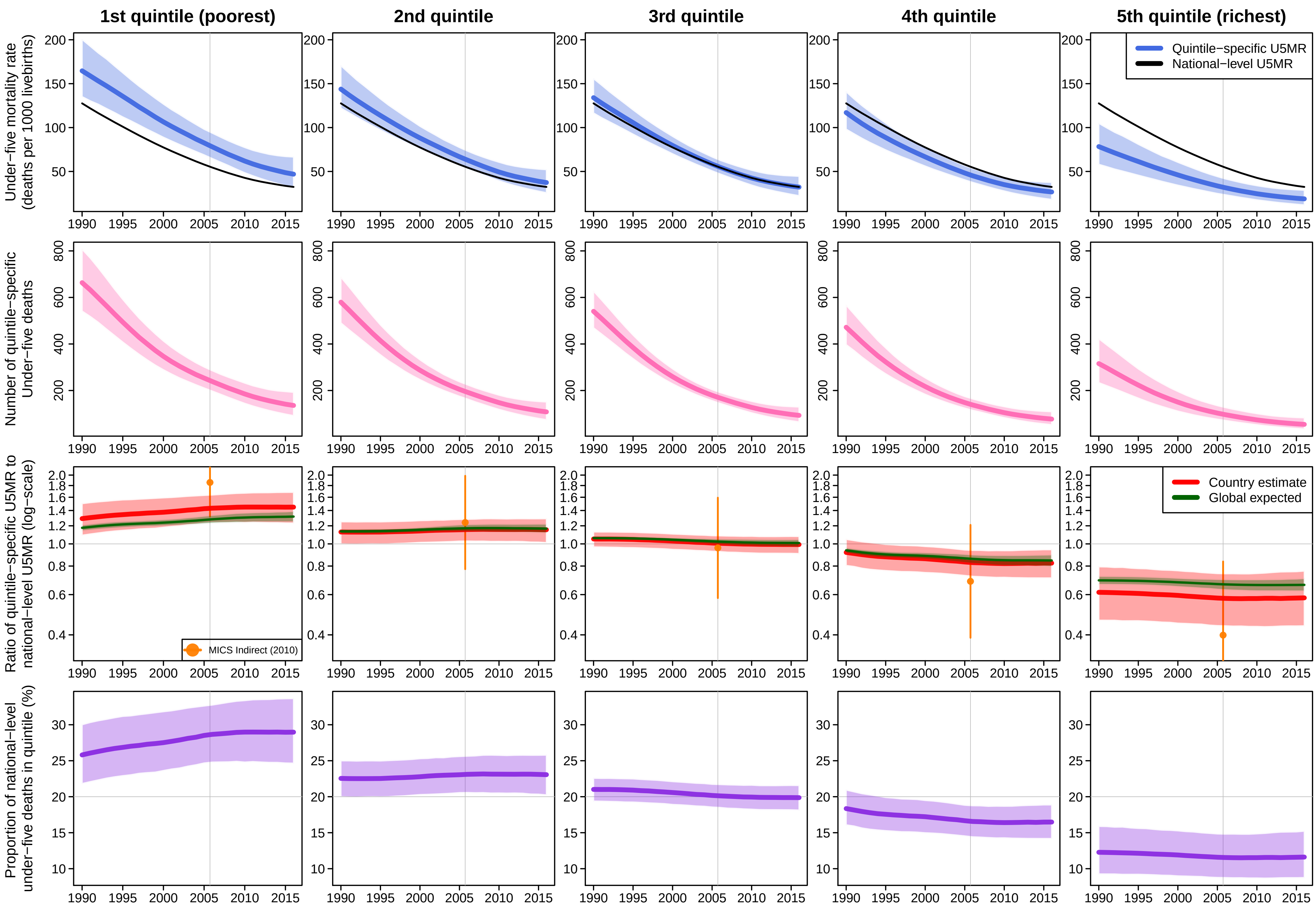

1st quintile (poorest)
2nd quintile
3rd quintile
4th quintile
5th quintile (richest)
Under−five mortality rate (deaths per 1000 livebirths)
Number of quintile−specific Under−five deaths
Ratio of quintile−specific U5MR to national−level U5MR (log−scale)
Proportion of national−level under−five deaths in quintile (%)
Quintile−specific U5MR
National−level U5MR
Country estimate
Global expected
MICS Indirect (2010)

Bolivia (Plurinational State of)

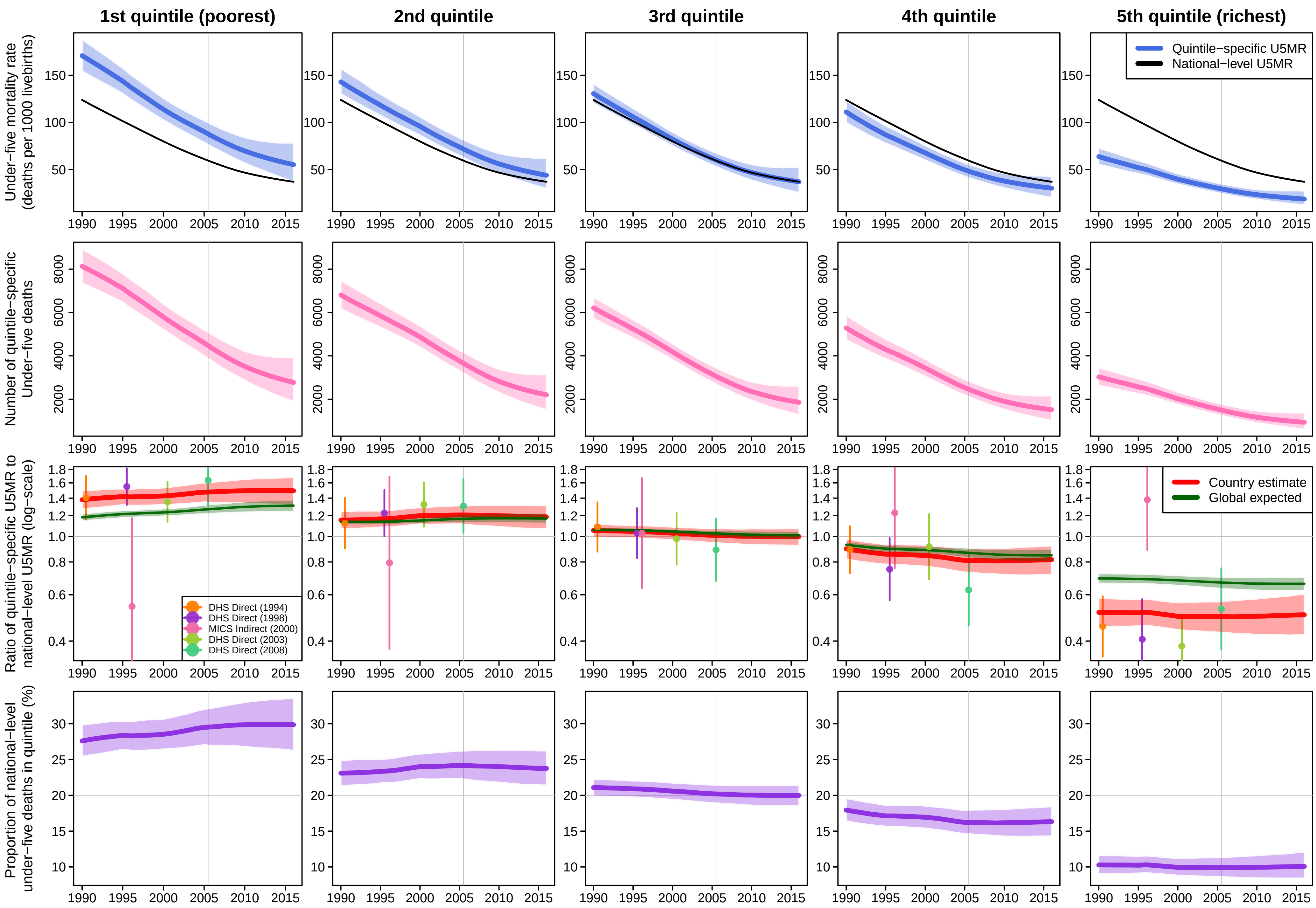

Brazil

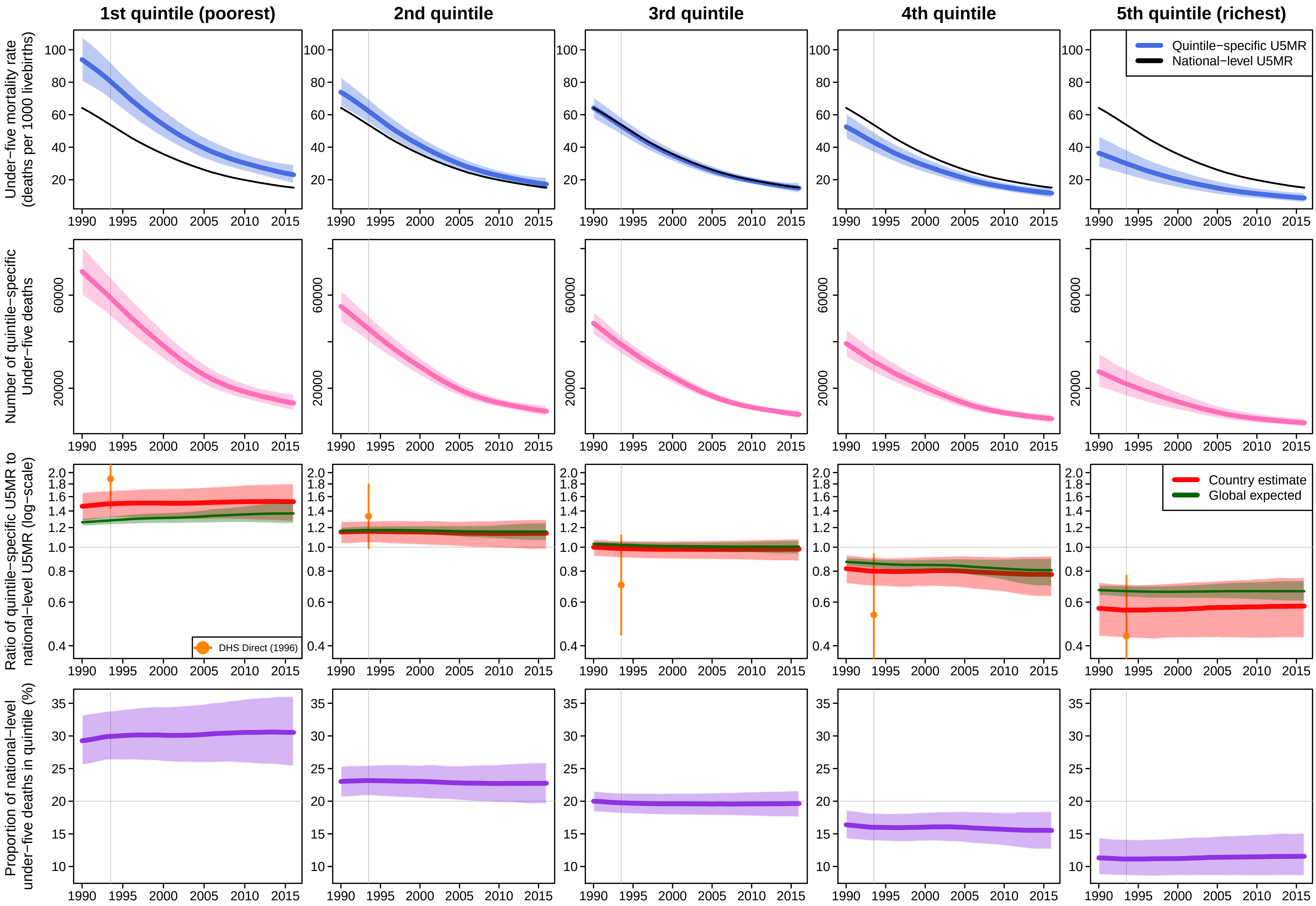
1st quintile (poorest)
2nd quintile
3rd quintile
4th quintile
5th quintile (richest)
Under−five mortality rate (deaths per 1000 livebirths)
Quintile−specific U5MR
National−level U5MR
Number of quintile−specific Under−five deaths
Ratio of quintile−specific U5MR to national−level U5MR (log−scale)
Country estimate
Global expected
DHS Direct (1996)
Proportion of national−level under−five deaths in quintile (%)

Burkina Faso

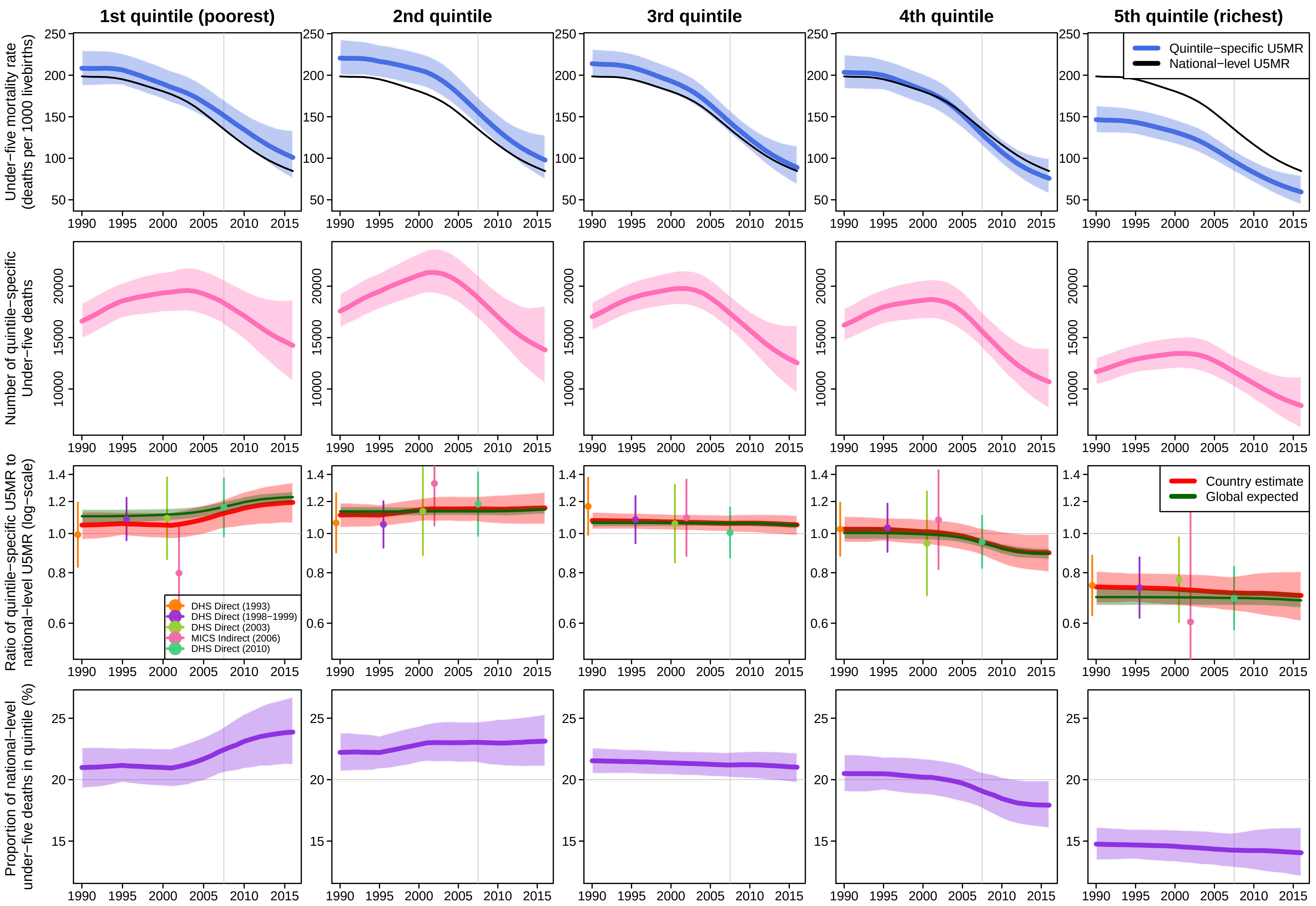

Burundi

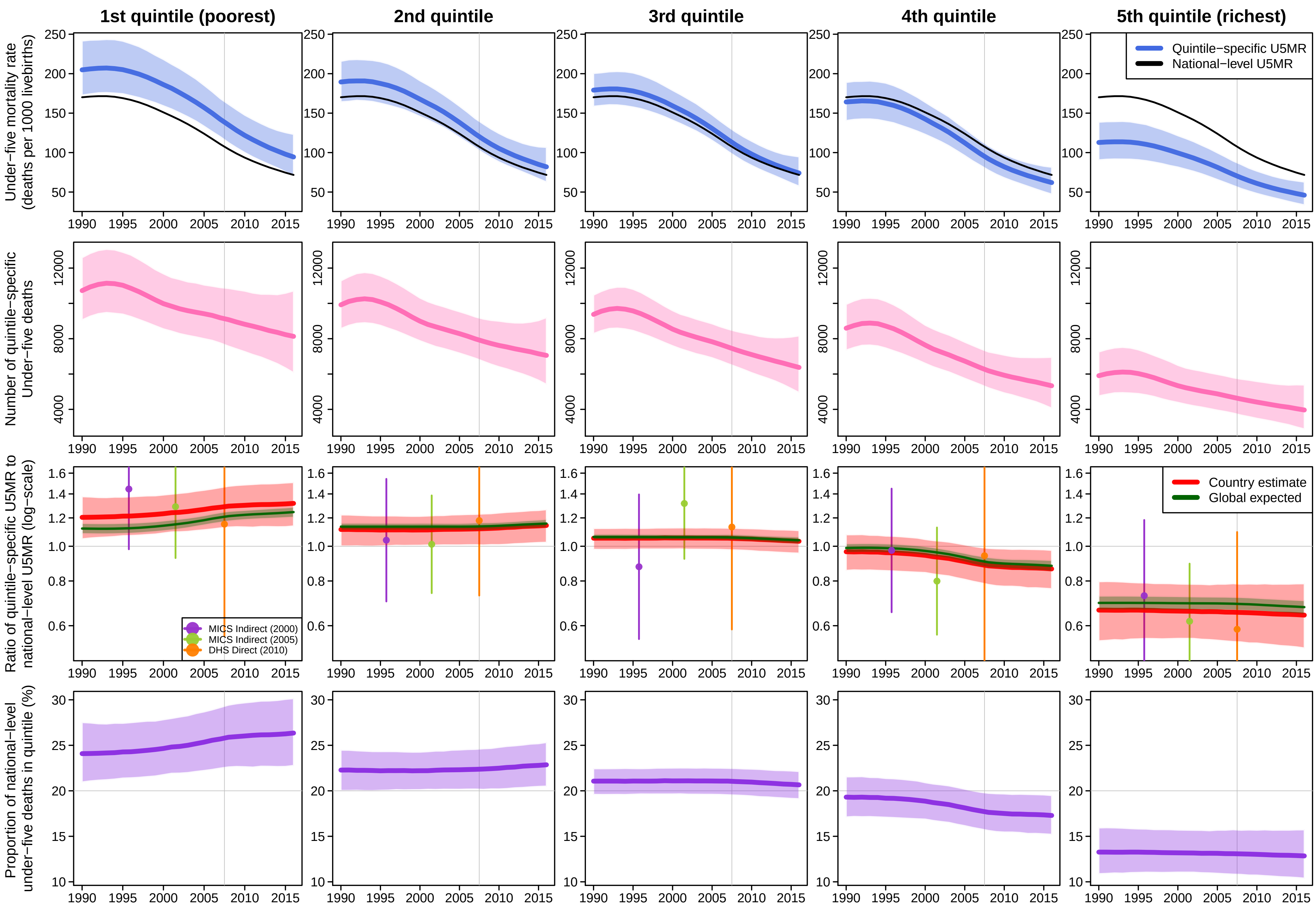
1st quintile (poorest)
2nd quintile
3rd quintile
4th quintile
5th quintile (richest)
Under−five mortality rate (deaths per 1000 livebirths)
Quintile−specific U5MR
National−level U5MR
Number of quintile−specific Under−five deaths
Ratio of quintile−specific U5MR to national−level U5MR (log−scale)
Country estimate
Global expected
MICS Indirect (2000)
MICS Indirect (2005)
DHS Direct (2010)
Proportion of national−level under−five deaths in quintile (%)

Cambodia

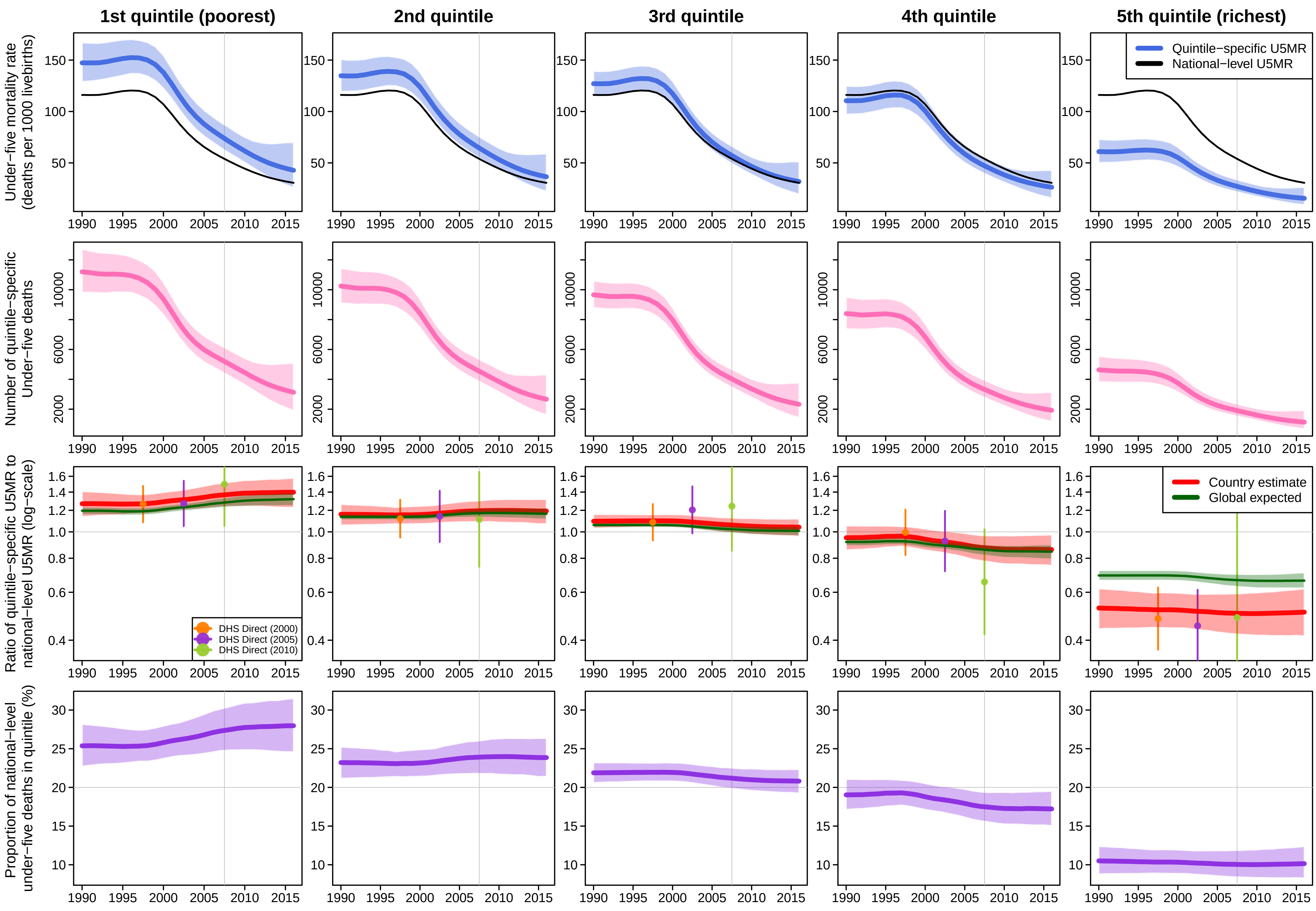

1st quintile (poorest)
2nd quintile
3rd quintile
4th quintile
5th quintile (richest)
Quintile−specific U5MR
National−level U5MR
Under−five mortality rate (deaths per 1000 livebirths)
Number of quintile−specific Under−five deaths
Ratio of quintile−specific U5MR to national−level U5MR (log−scale)
Country estimate
Global expected
DHS Direct (2000)
DHS Direct (2005)
DHS Direct (2010)
Proportion of national−level under−five deaths in quintile (%)

Cameroon

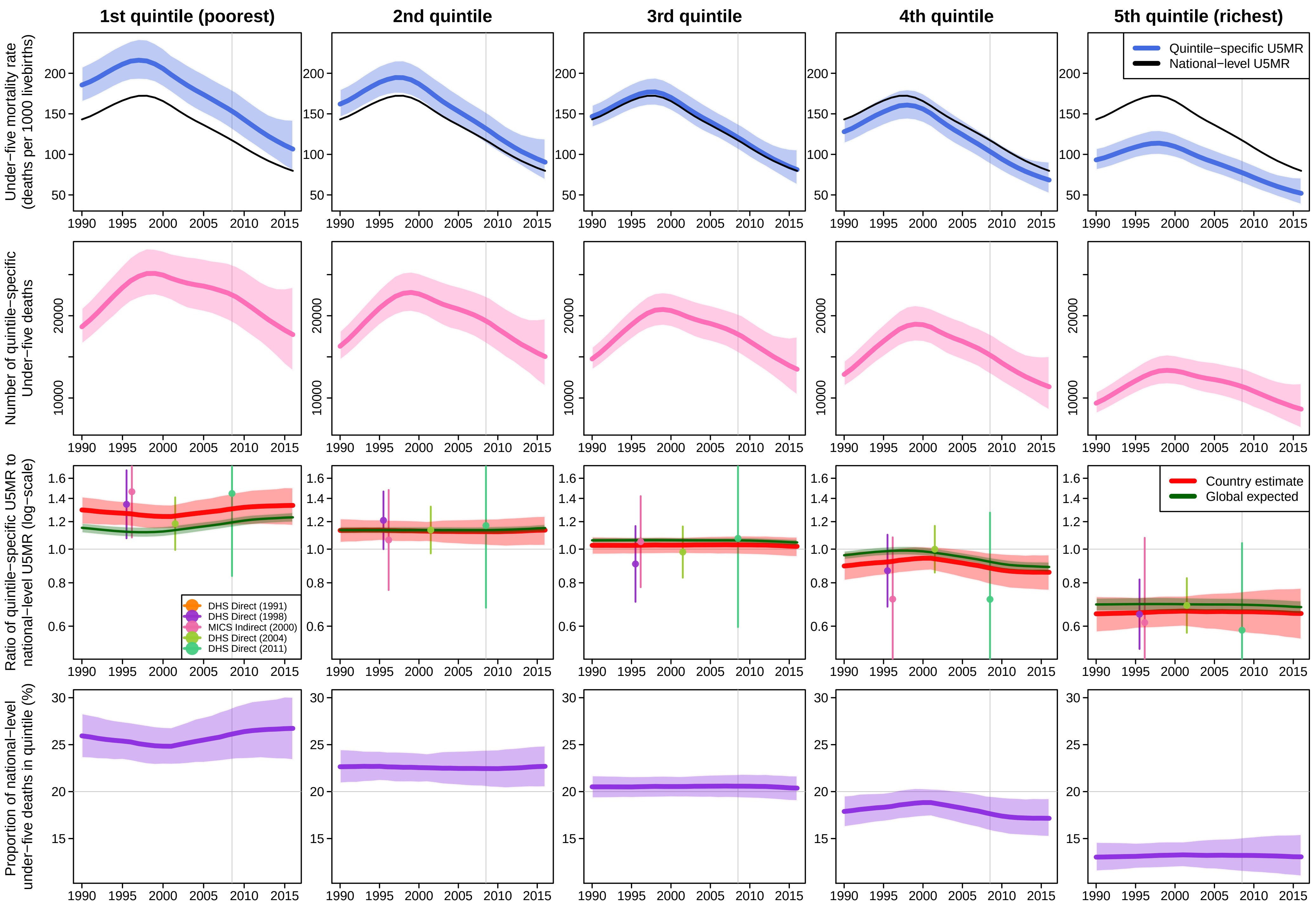

1st quintile (poorest)
2nd quintile
3rd quintile
4th quintile
5th quintile (richest)
Quintile−specific U5MR
National−level U5MR
Under−five mortality rate
(deaths per 1000 livebirths)
Number of quintile−specific
Under−five deaths
Ratio of quintile−specific U5MR to
national−level U5MR (log−scale)
Country estimate
Global expected
DHS Direct (1991)
DHS Direct (1998)
MICS Indirect (2000)
DHS Direct (2004)
DHS Direct (2011)
Proportion of national−level
under−five deaths in quintile (%)

Central African Republic

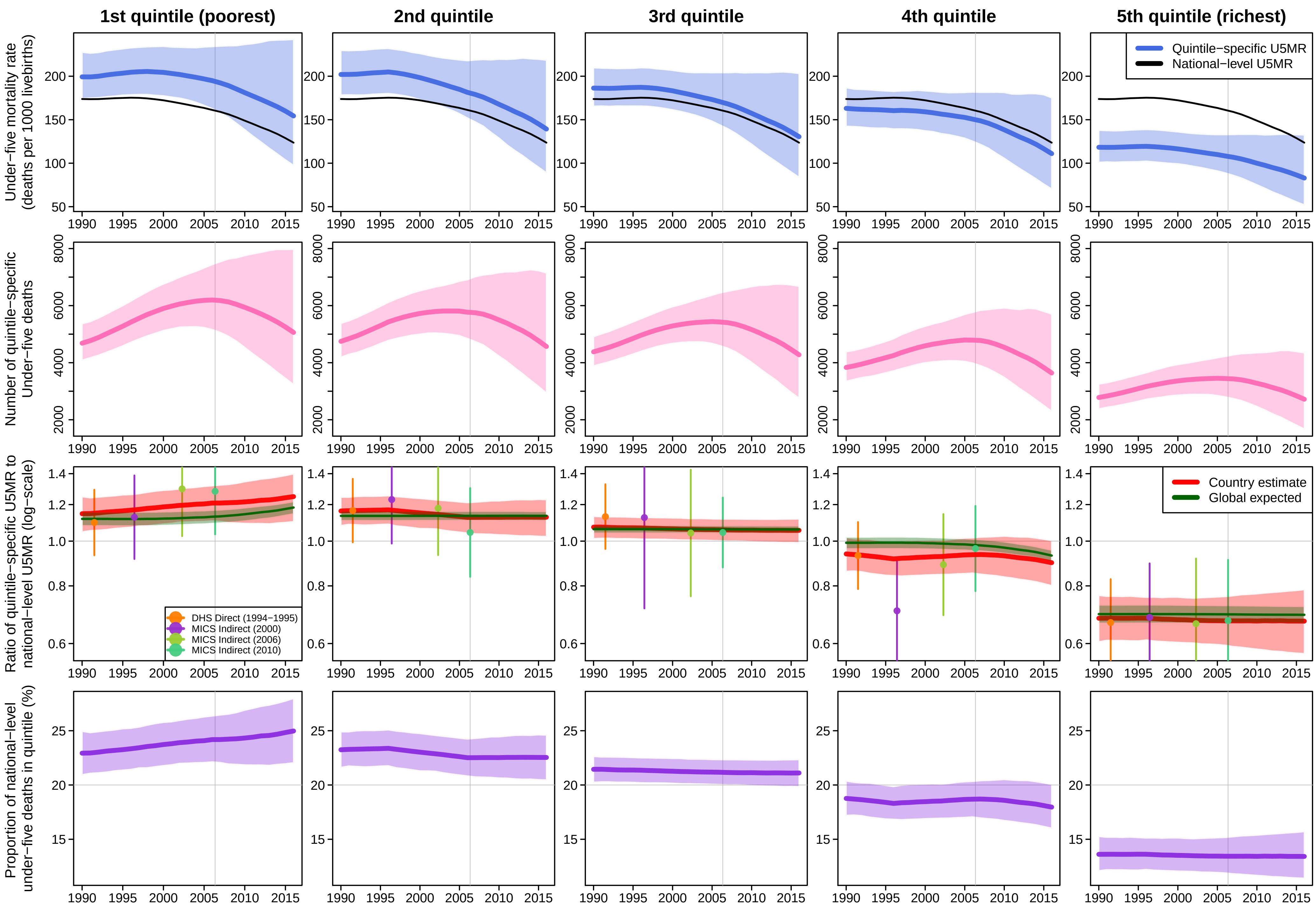

Chad

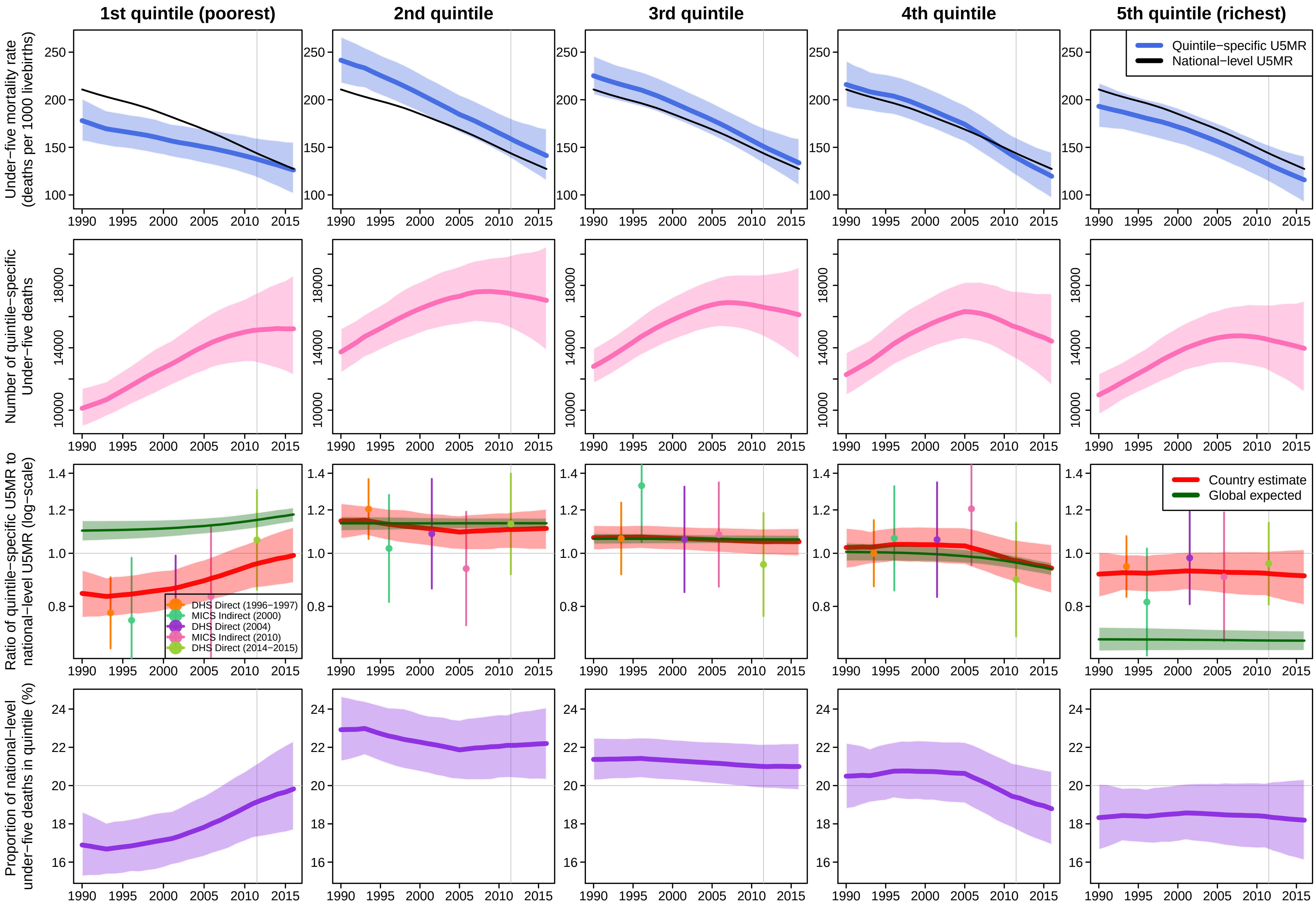
1st quintile (poorest)
2nd quintile
3rd quintile
4th quintile
5th quintile (richest)
Under−five mortality rate (deaths per 1000 livebirths)
Quintile−specific U5MR
National−level U5MR
Number of quintile−specific Under−five deaths
Ratio of quintile−specific U5MR to national−level U5MR (log−scale)
Country estimate
Global expected
DHS Direct (1996−1997)
MICS Indirect (2000)
DHS Direct (2004)
MICS Indirect (2010)
DHS Direct (2014−2015)
Proportion of national−level under−five deaths in quintile (%)

Colombia

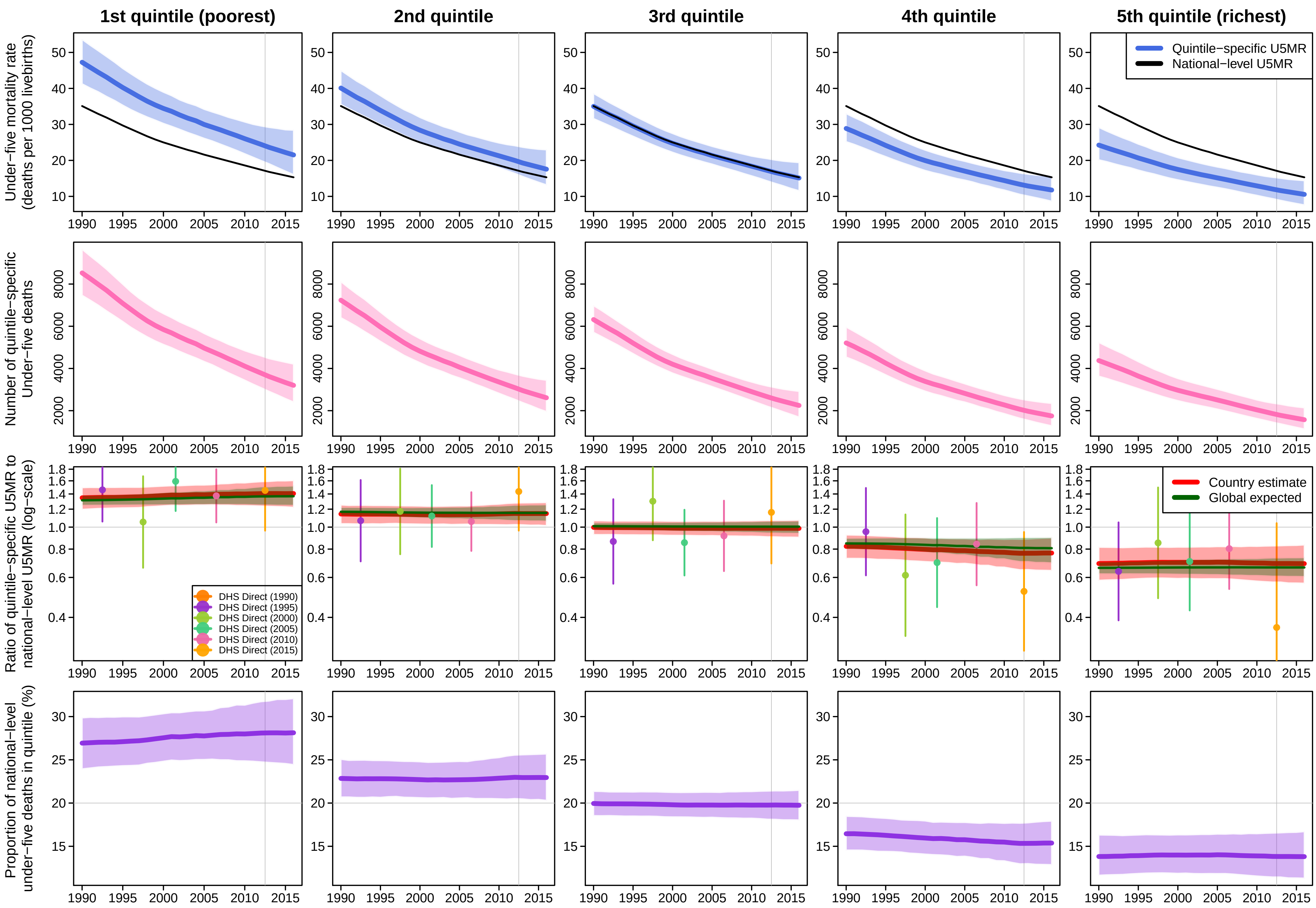

1st quintile (poorest)
2nd quintile
3rd quintile
4th quintile
5th quintile (richest)
Quintile−specific U5MR
National−level U5MR
Under−five mortality rate (deaths per 1000 livebirths)
Number of quintile−specific Under−five deaths
Ratio of quintile−specific U5MR to national−level U5MR (log−scale)
Country estimate
Global expected
DHS Direct (1990)
DHS Direct (1995)
DHS Direct (2000)
DHS Direct (2005)
DHS Direct (2010)
DHS Direct (2015)
Proportion of national−level under−five deaths in quintile (%)

Comoros

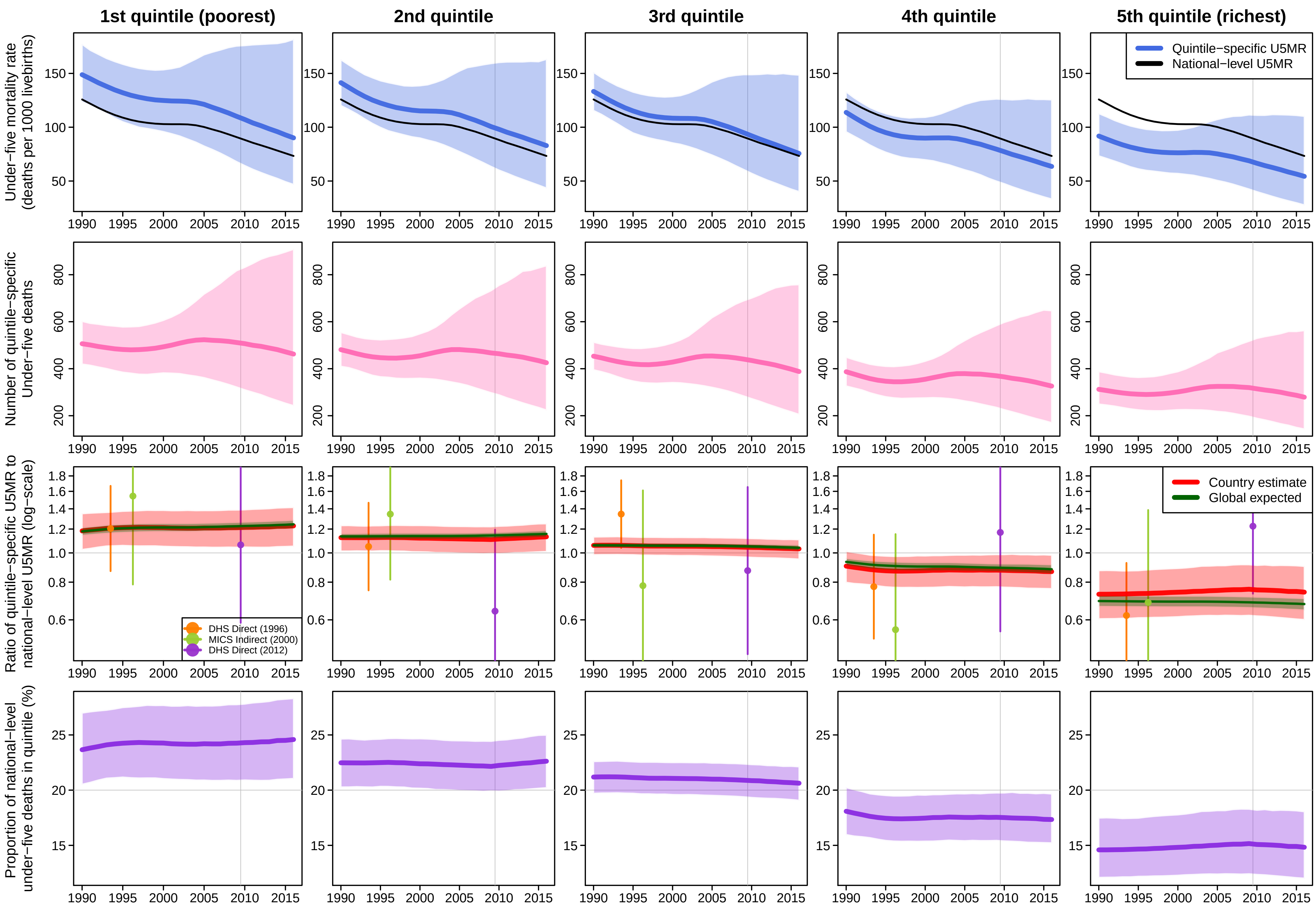

1st quintile (poorest)
2nd quintile
3rd quintile
4th quintile
5th quintile (richest)
Under−five mortality rate (deaths per 1000 livebirths)
Quintile−specific U5MR
National−level U5MR
Number of quintile−specific Under−five deaths
Ratio of quintile−specific U5MR to national−level U5MR (log−scale)
Country estimate
Global expected
DHS Direct (1996)
MICS Indirect (2000)
DHS Direct (2012)
Proportion of national−level under−five deaths in quintile (%)

Congo

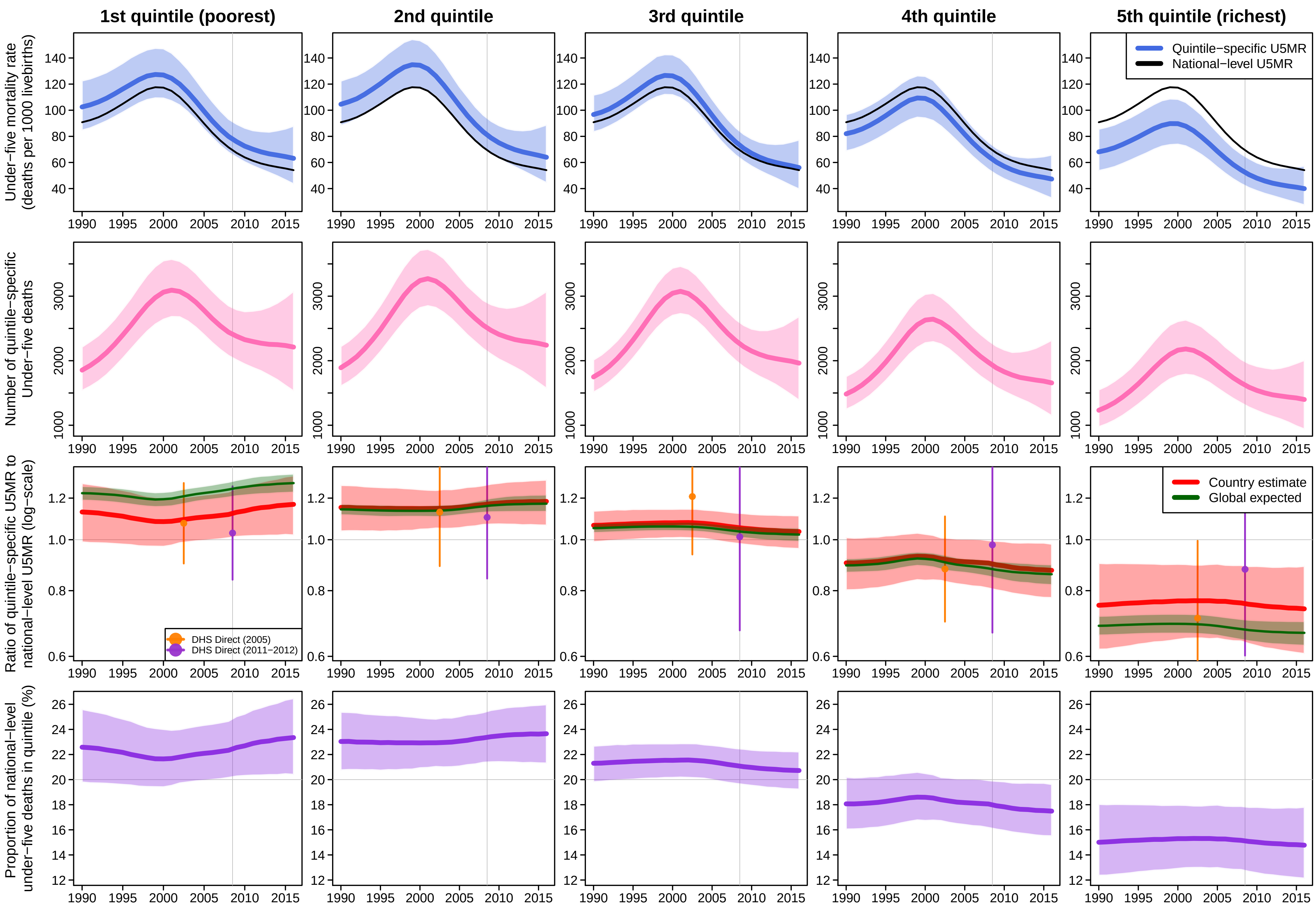
1st quintile (poorest)
2nd quintile
3rd quintile
4th quintile
5th quintile (richest)
Under−five mortality rate (deaths per 1000 livebirths)
Quintile−specific U5MR
National−level U5MR
Number of quintile−specific Under−five deaths
Ratio of quintile−specific U5MR to national−level U5MR (log−scale)
Country estimate
Global expected
DHS Direct (2005)
DHS Direct (2011−2012)
Proportion of national−level under−five deaths in quintile (%)

Cote d'Ivoire

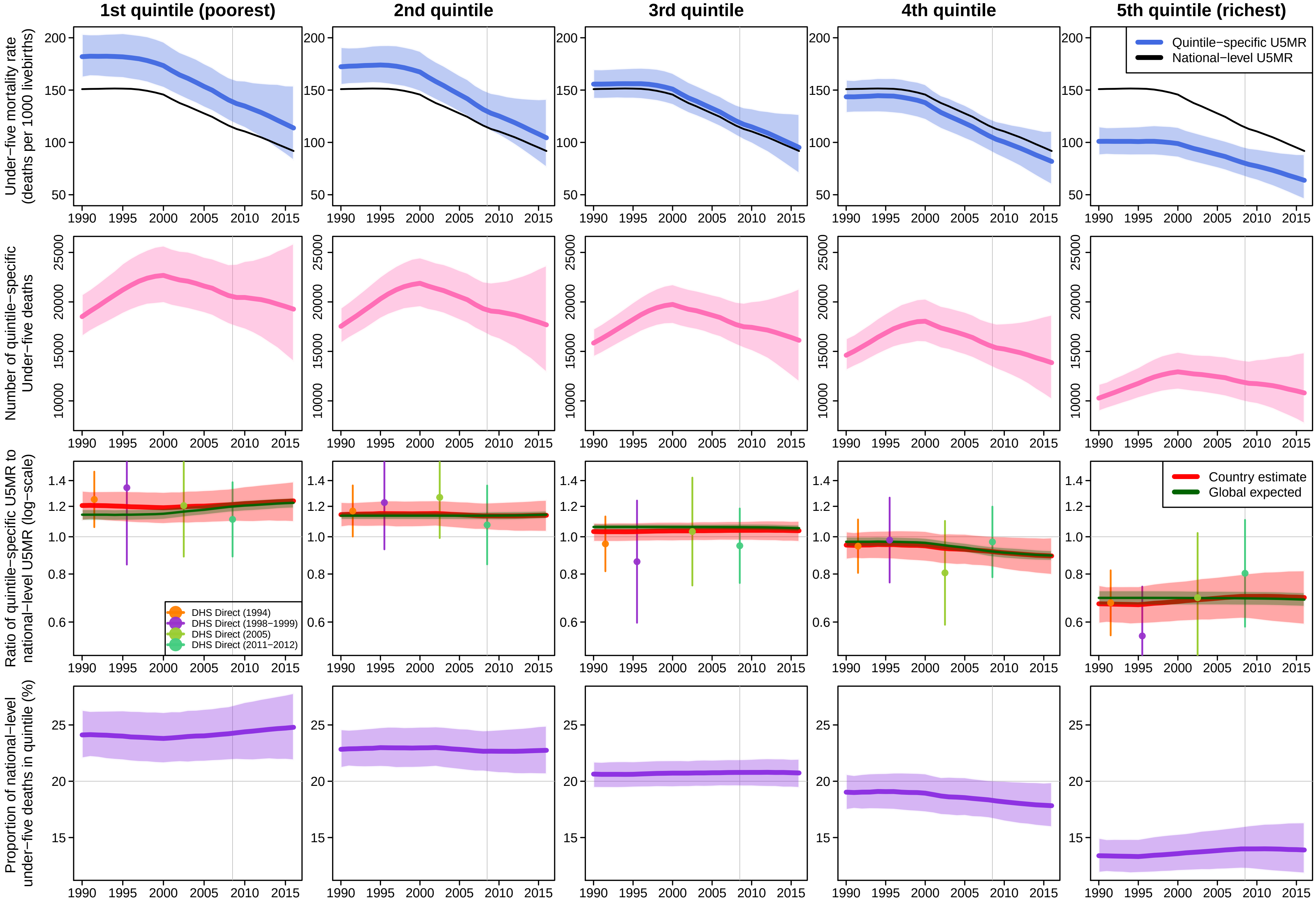

Democratic Republic of the Congo

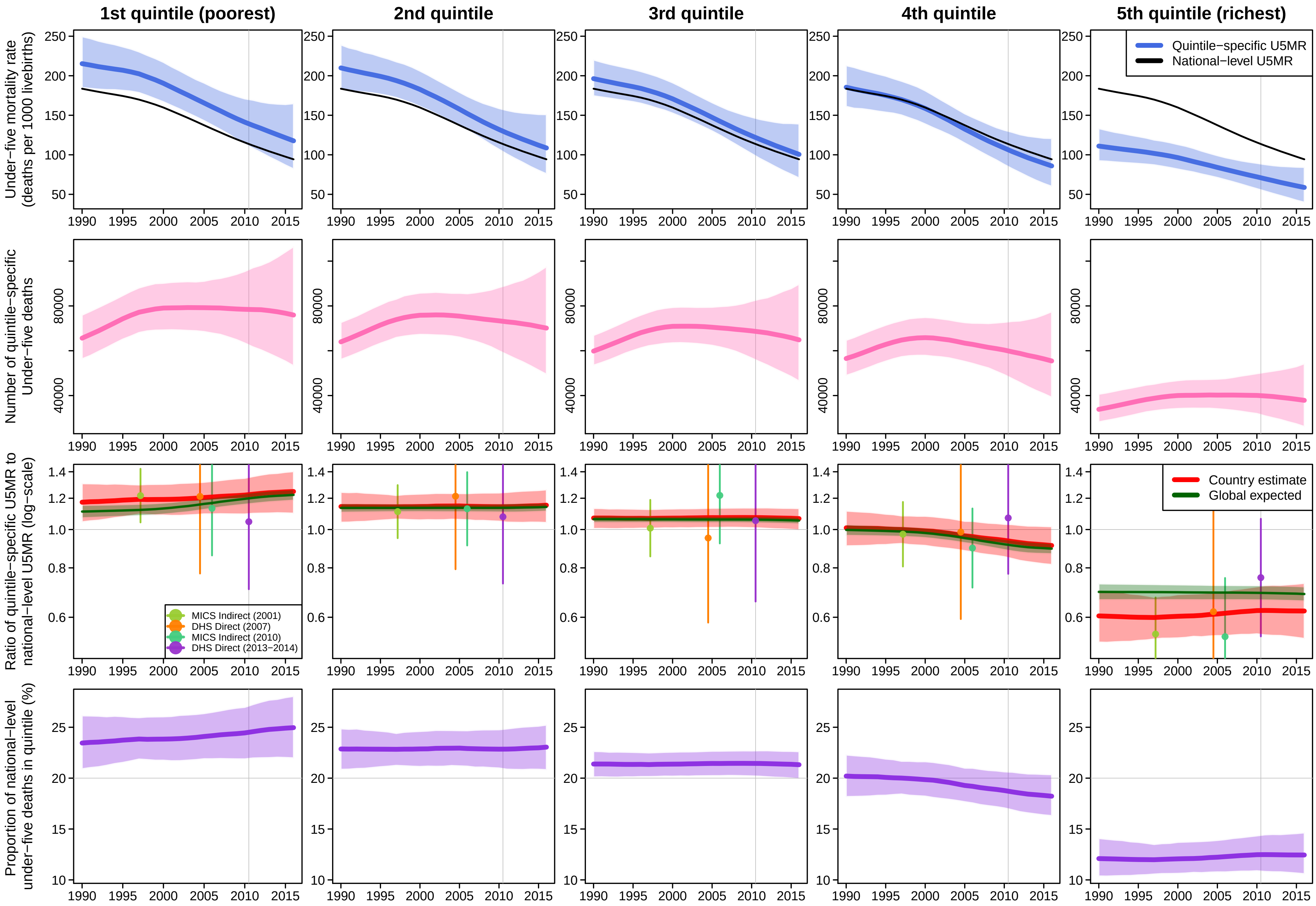

Dominican Republic

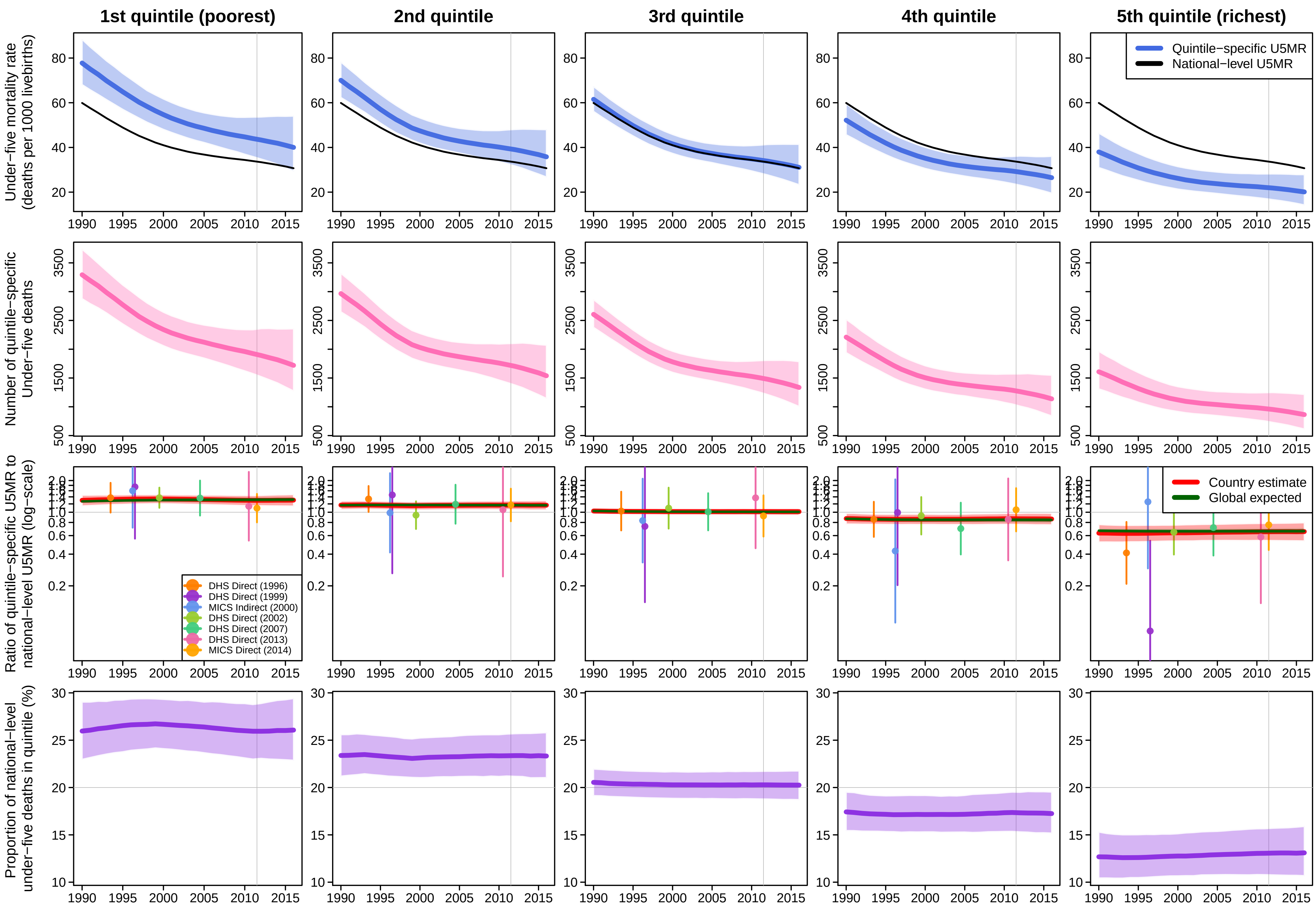

Egypt

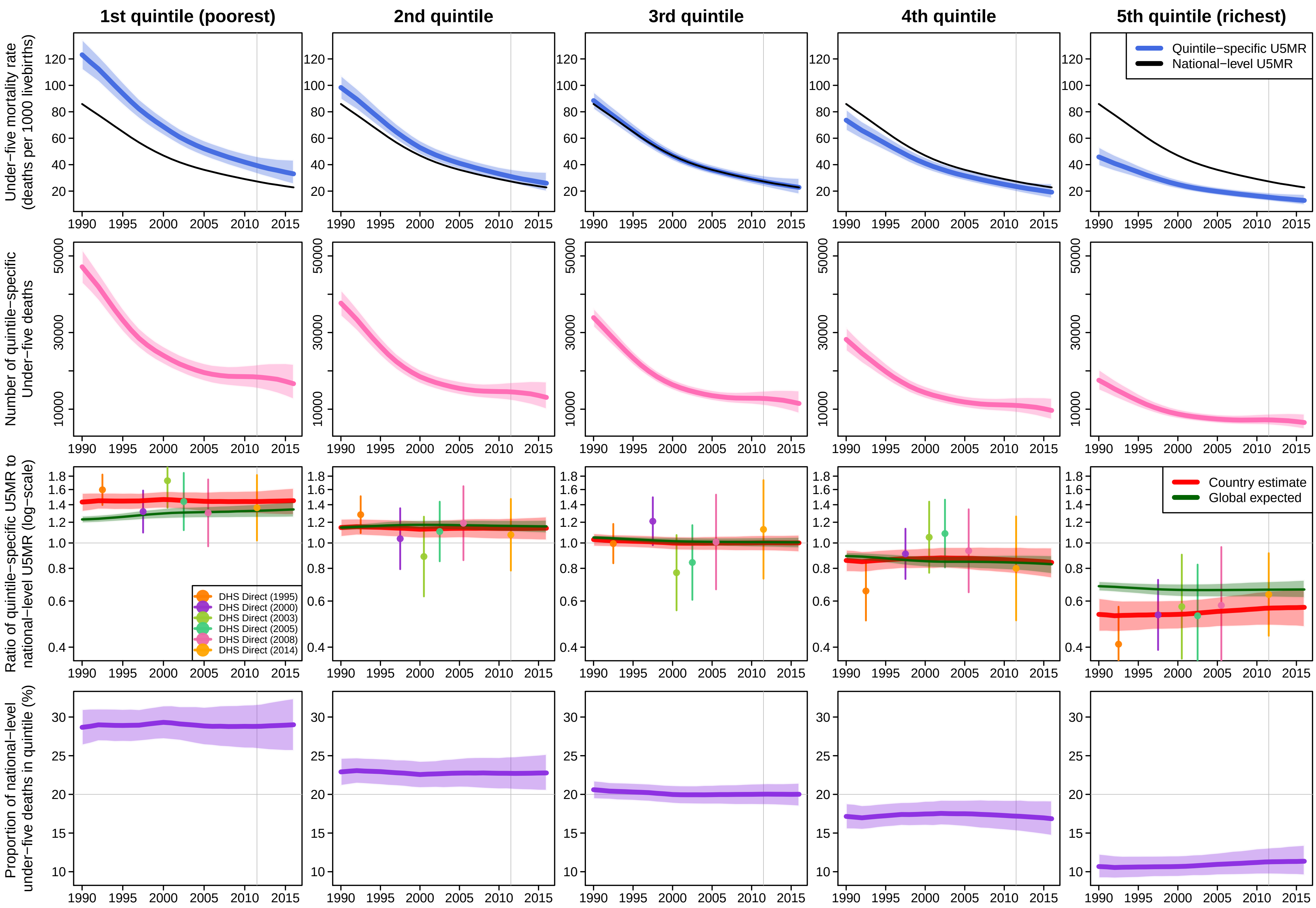

1st quintile (poorest)
2nd quintile
3rd quintile
4th quintile
5th quintile (richest)
Under−five mortality rate (deaths per 1000 livebirths)
Quintile−specific U5MR
National−level U5MR
Number of quintile−specific Under−five deaths
Ratio of quintile−specific U5MR to national−level U5MR (log−scale)
DHS Direct (1995)
DHS Direct (2000)
DHS Direct (2003)
DHS Direct (2005)
DHS Direct (2008)
DHS Direct (2014)
Country estimate
Global expected
Proportion of national−level under−five deaths in quintile (%)

El Salvador

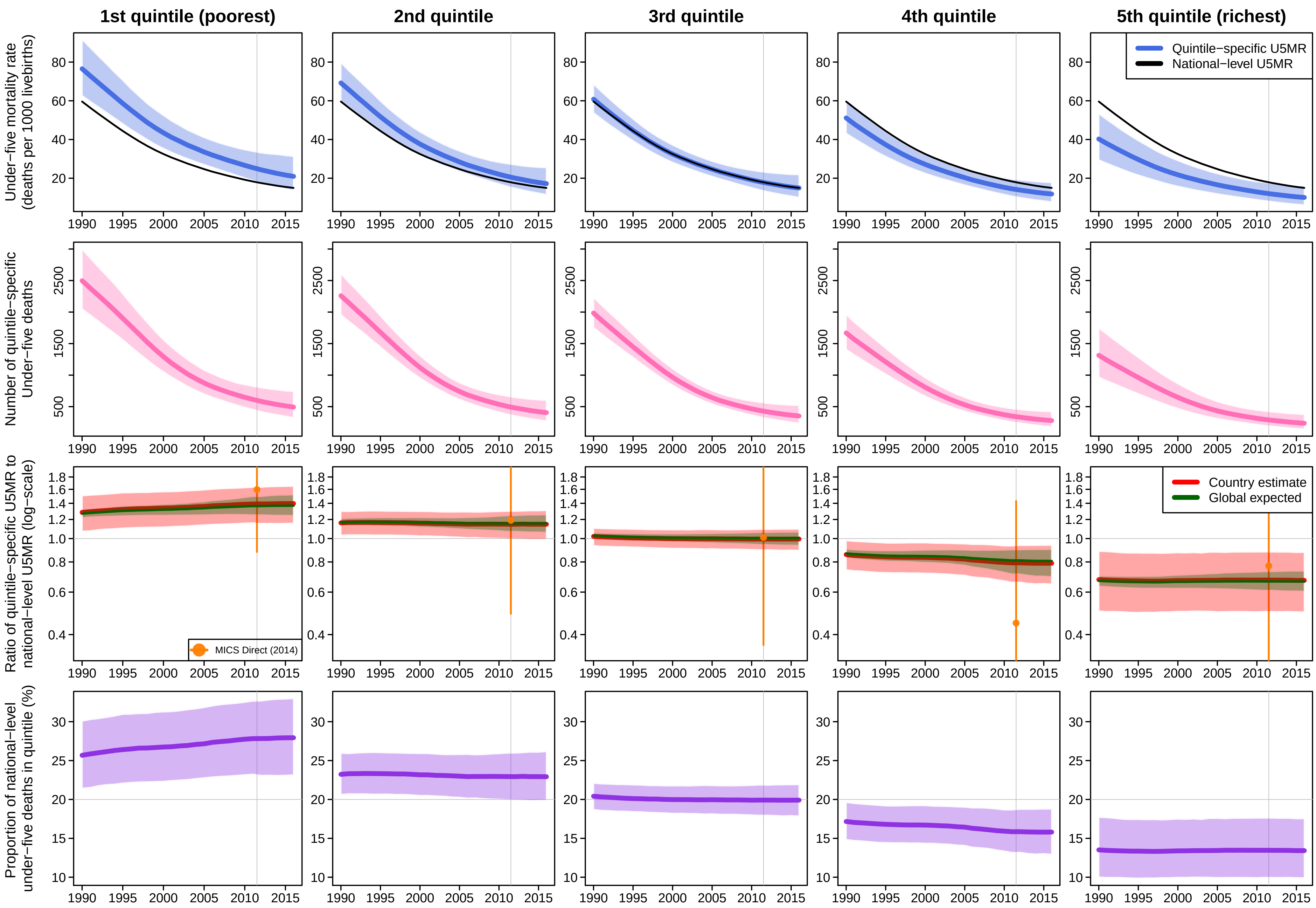

1st quintile (poorest)
2nd quintile
3rd quintile
4th quintile
5th quintile (richest)
Under−five mortality rate (deaths per 1000 livebirths)
Quintile−specific U5MR
National−level U5MR
Number of quintile−specific Under−five deaths
Ratio of quintile−specific U5MR to national−level U5MR (log−scale)
Country estimate
Global expected
MICS Direct (2014)
Proportion of national−level under−five deaths in quintile (%)

Equatorial Guinea

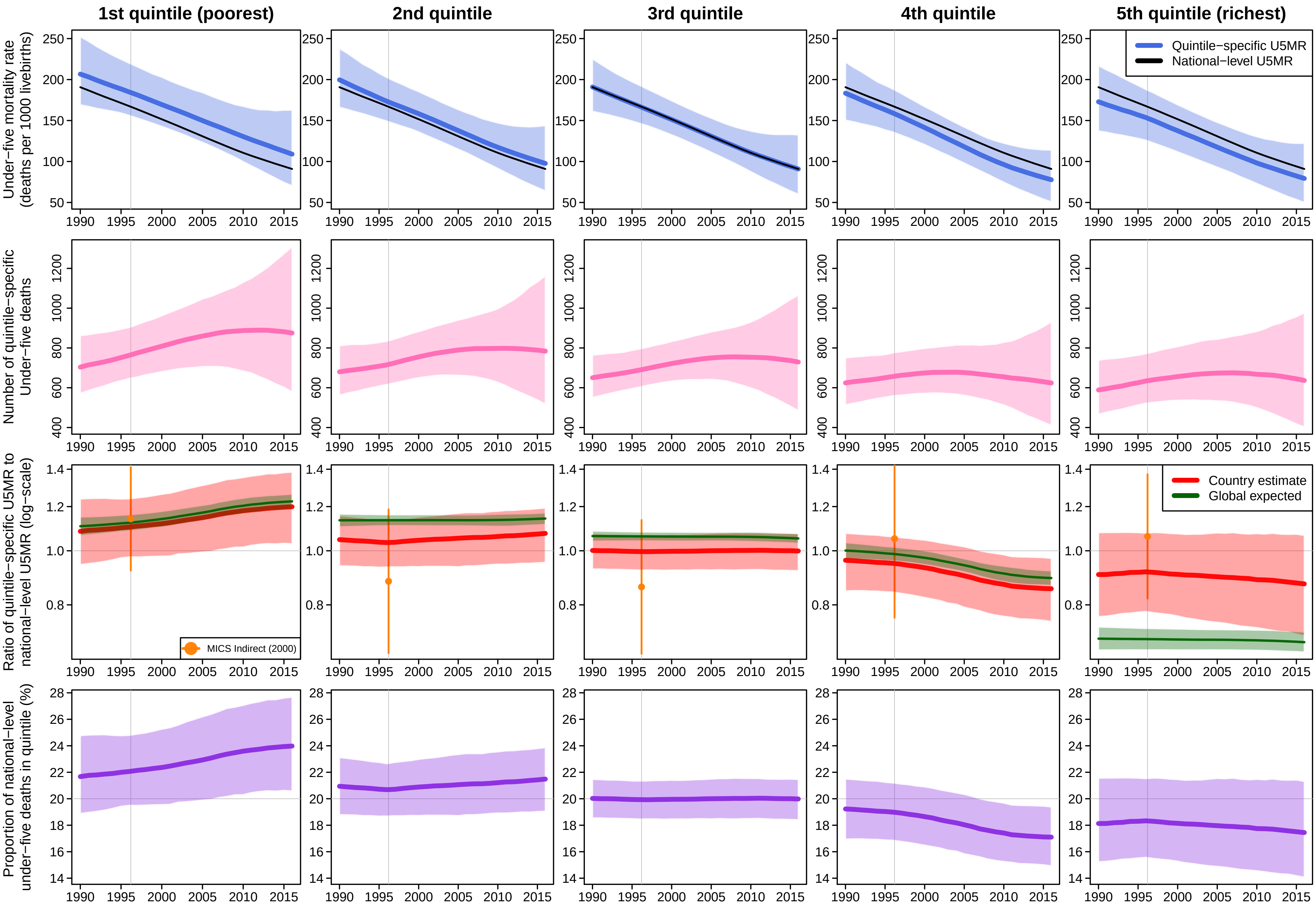

Eritrea

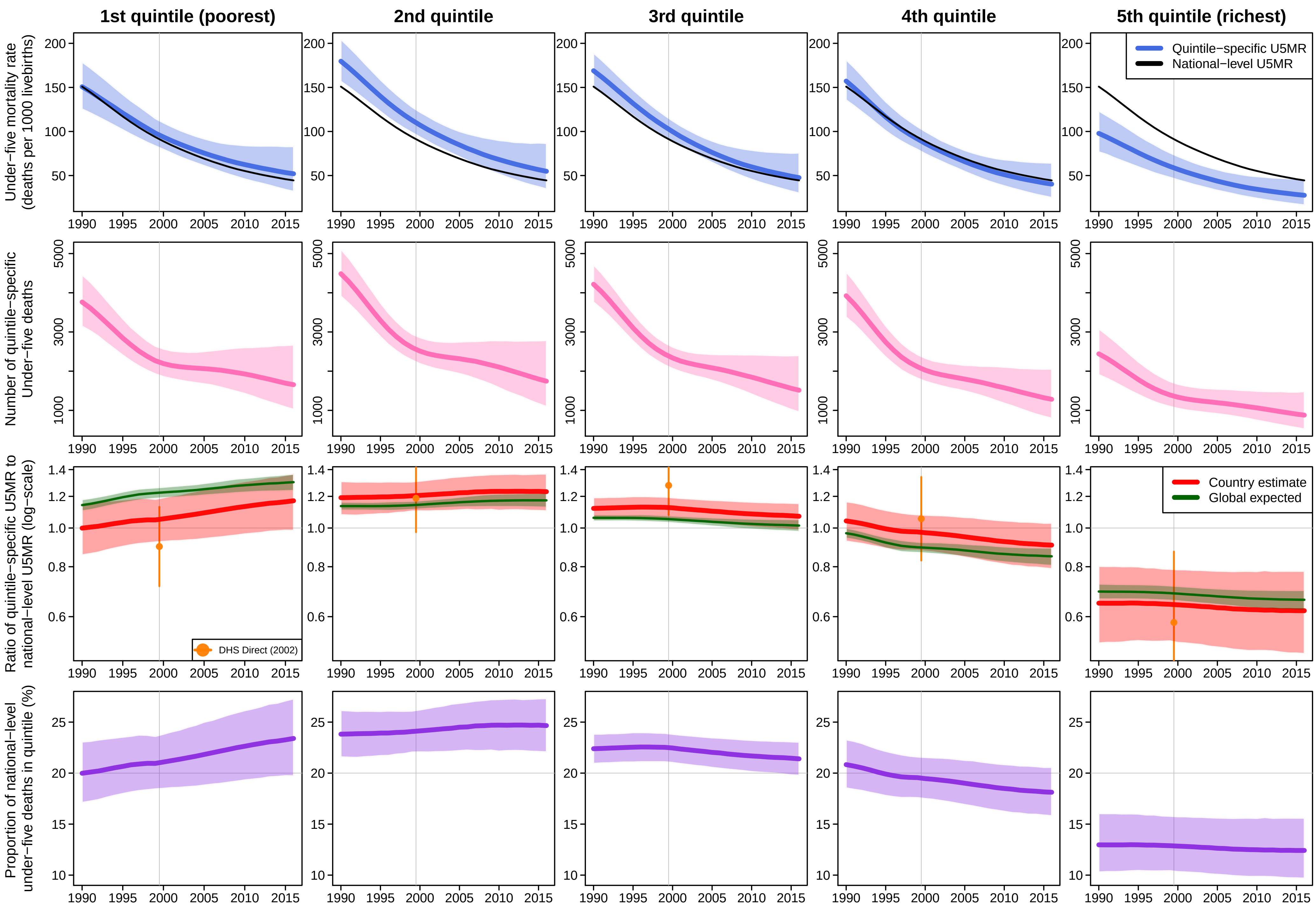
1st quintile (poorest)
2nd quintile
3rd quintile
4th quintile
5th quintile (richest)
Under−five mortality rate (deaths per 1000 livebirths)
Quintile−specific U5MR
National−level U5MR
Number of quintile−specific Under−five deaths
Ratio of quintile−specific U5MR to national−level U5MR (log−scale)
Country estimate
Global expected
DHS Direct (2002)
Proportion of national−level under−five deaths in quintile (%)

Ethiopia

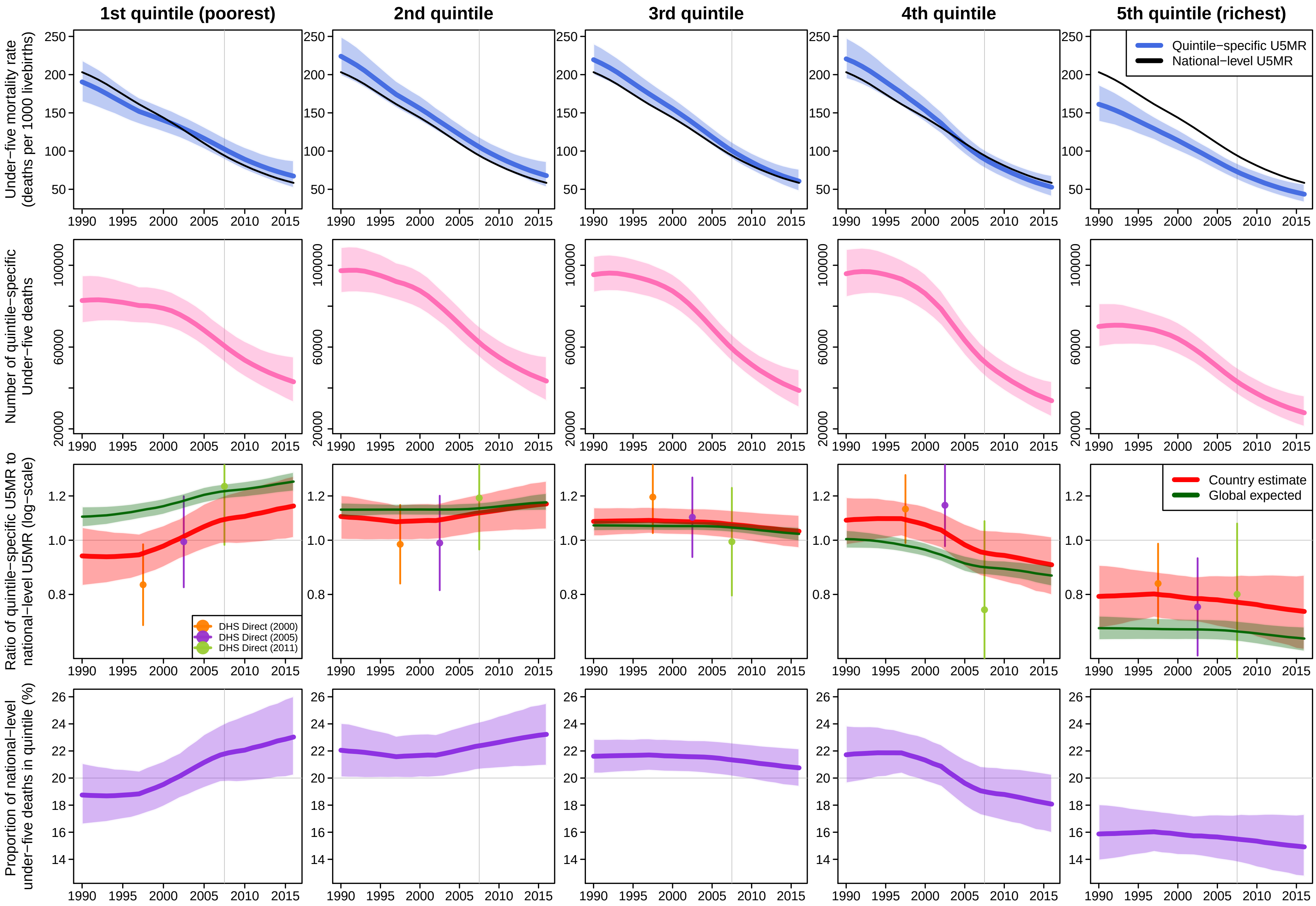

1st quintile (poorest)
2nd quintile
3rd quintile
4th quintile
5th quintile (richest)
Under−five mortality rate (deaths per 1000 livebirths)
Quintile−specific U5MR
National−level U5MR
Number of quintile−specific Under−five deaths
Ratio of quintile−specific U5MR to national−level U5MR (log−scale)
Country estimate
Global expected
DHS Direct (2000)
DHS Direct (2005)
DHS Direct (2011)
Proportion of national−level under−five deaths in quintile (%)

Gabon

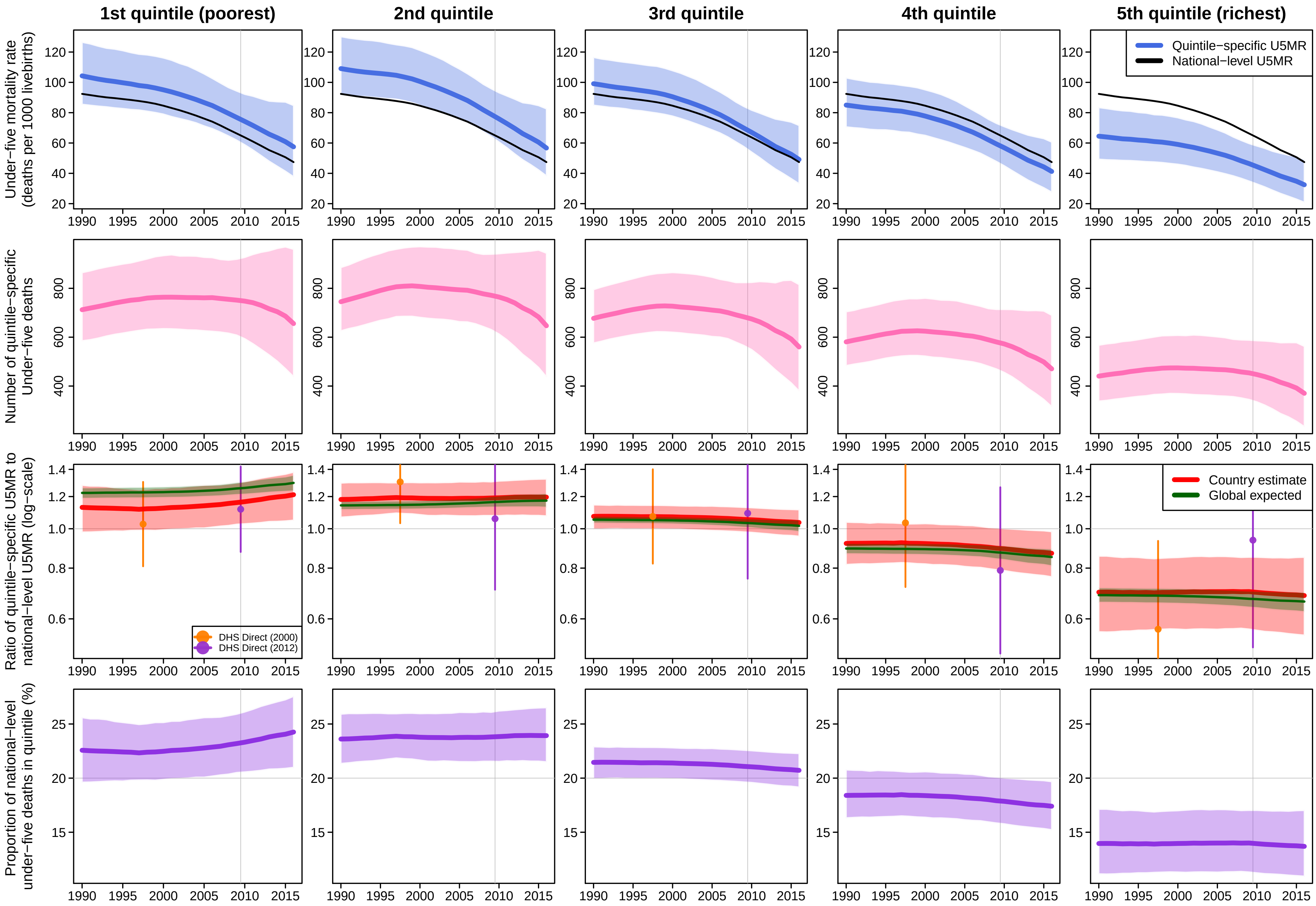
1st quintile (poorest)
2nd quintile
3rd quintile
4th quintile
5th quintile (richest)
Under−five mortality rate (deaths per 1000 livebirths)
Quintile−specific U5MR
National−level U5MR
Number of quintile−specific Under−five deaths
Ratio of quintile−specific U5MR to national−level U5MR (log−scale)
Country estimate
Global expected
DHS Direct (2000)
DHS Direct (2012)
Proportion of national−level under−five deaths in quintile (%)

Georgia

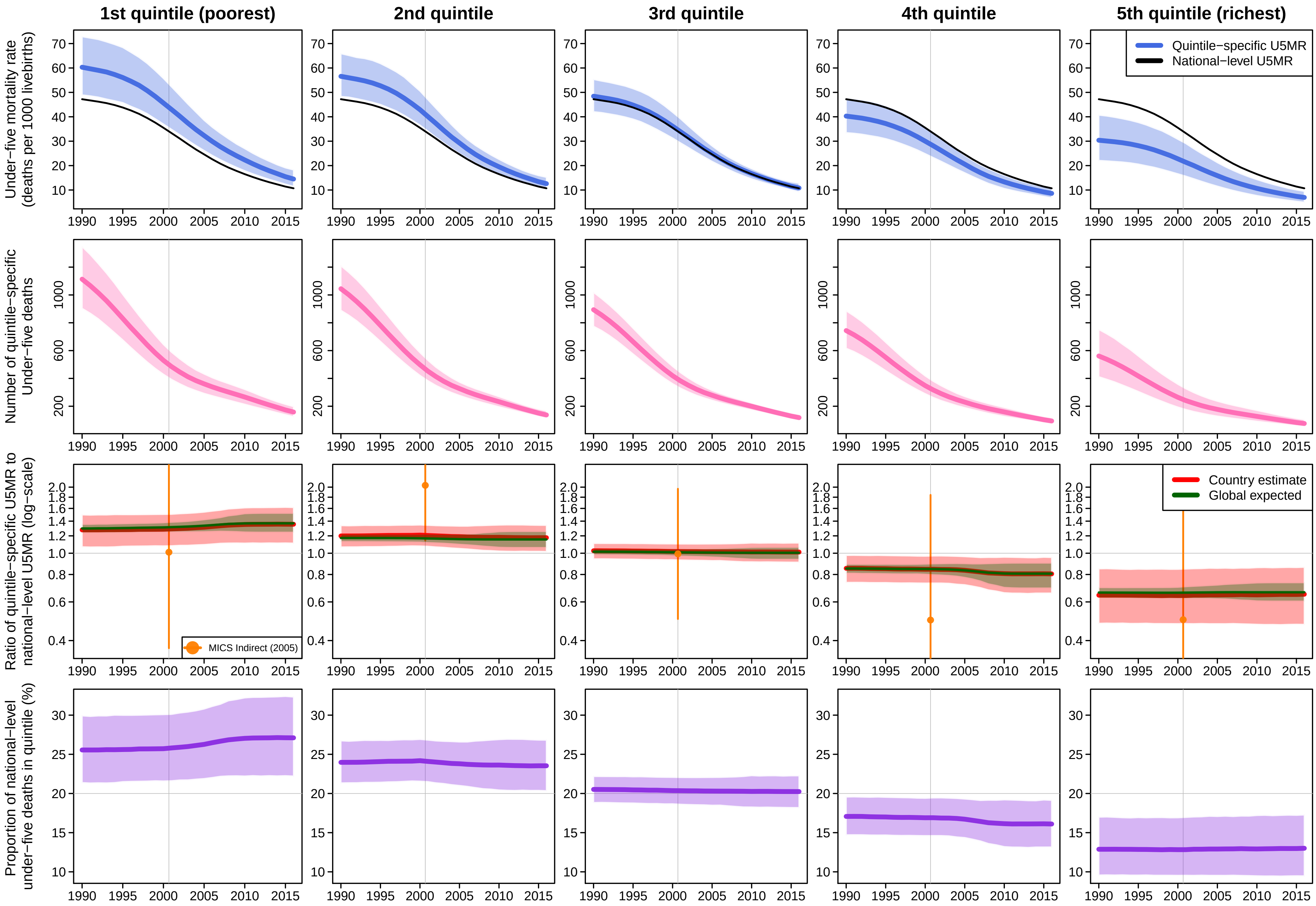
1st quintile (poorest)
2nd quintile
3rd quintile
4th quintile
5th quintile (richest)
Under−five mortality rate (deaths per 1000 livebirths)
Quintile−specific U5MR
National−level U5MR
Number of quintile−specific Under−five deaths
Ratio of quintile−specific U5MR to national−level U5MR (log−scale)
Country estimate
Global expected
MICS Indirect (2005)
Proportion of national−level under−five deaths in quintile (%)

Ghana

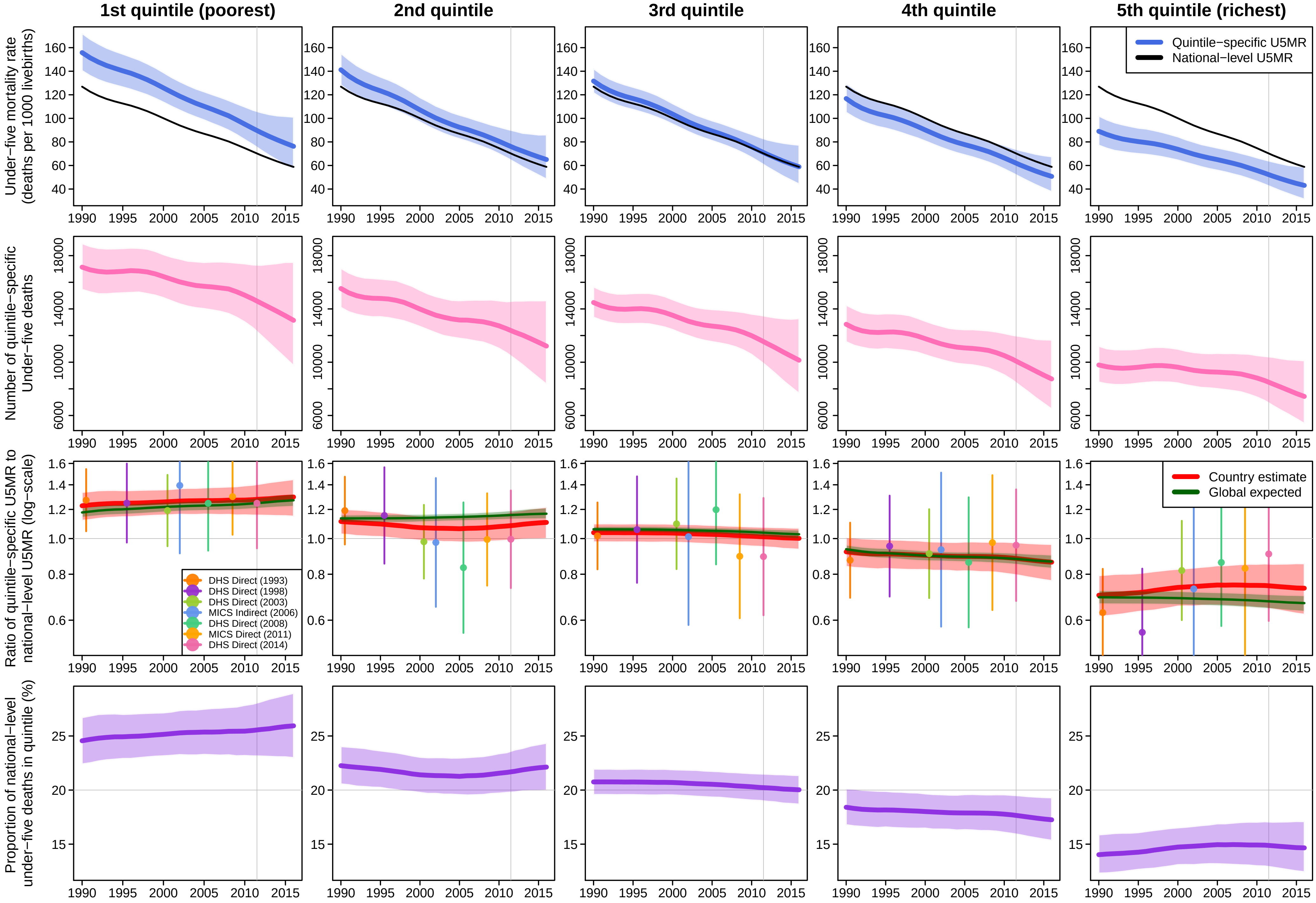
1st quintile (poorest)
2nd quintile
3rd quintile
4th quintile
5th quintile (richest)
Under−five mortality rate (deaths per 1000 livebirths)
Quintile−specific U5MR
National−level U5MR
Number of quintile−specific Under−five deaths
Ratio of quintile−specific U5MR to national−level U5MR (log−scale)
Country estimate
Global expected
DHS Direct (1993)
DHS Direct (1998)
DHS Direct (2003)
MICS Indirect (2006)
DHS Direct (2008)
MICS Direct (2011)
DHS Direct (2014)
Proportion of national−level under−five deaths in quintile (%)

Guatemala

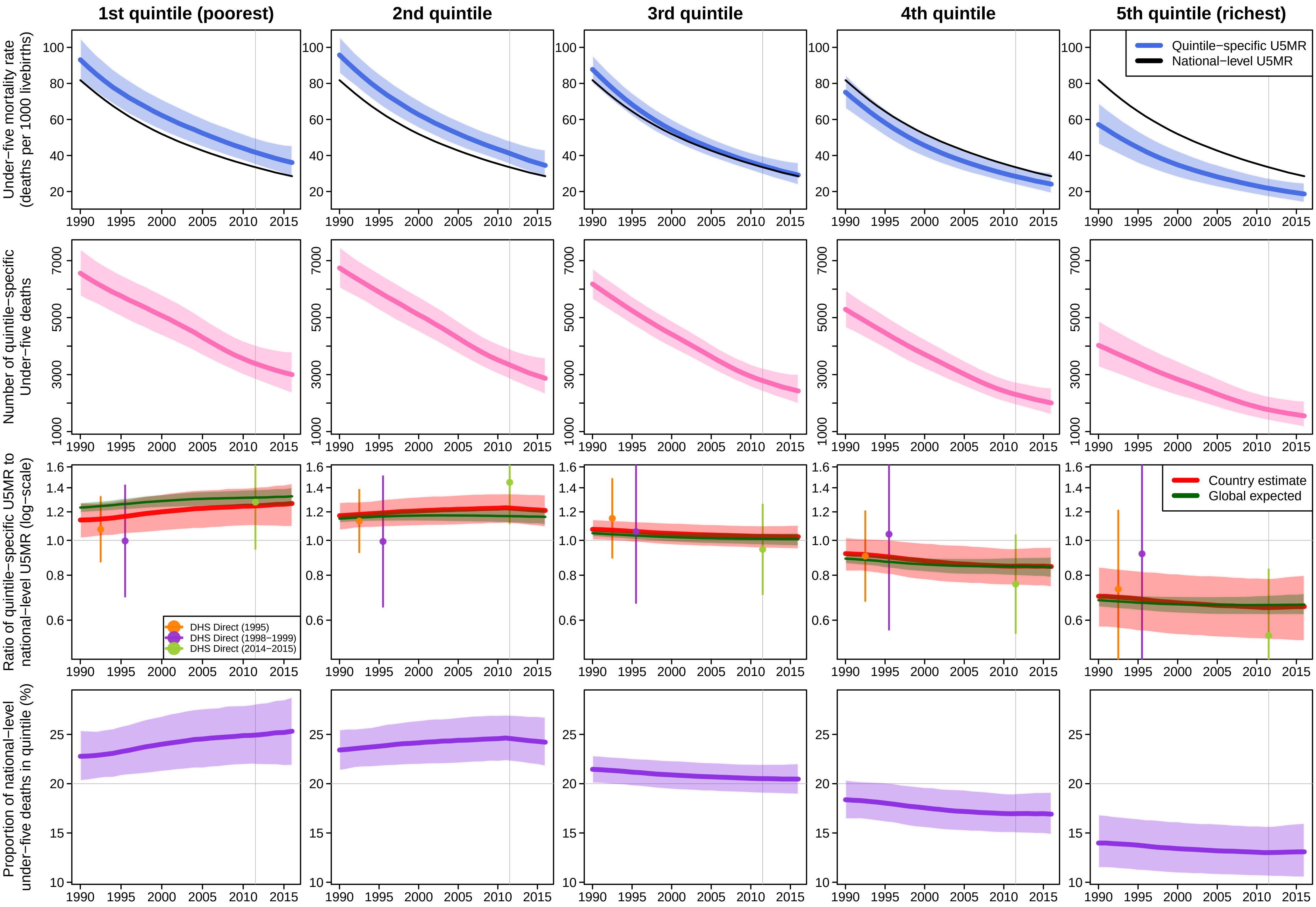
1st quintile (poorest)
2nd quintile
3rd quintile
4th quintile
5th quintile (richest)
Under−five mortality rate (deaths per 1000 livebirths)
Quintile−specific U5MR
National−level U5MR
Number of quintile−specific Under−five deaths
Ratio of quintile−specific U5MR to national−level U5MR (log−scale)
Country estimate
Global expected
DHS Direct (1995)
DHS Direct (1998−1999)
DHS Direct (2014−2015)
Proportion of national−level under−five deaths in quintile (%)

Guinea

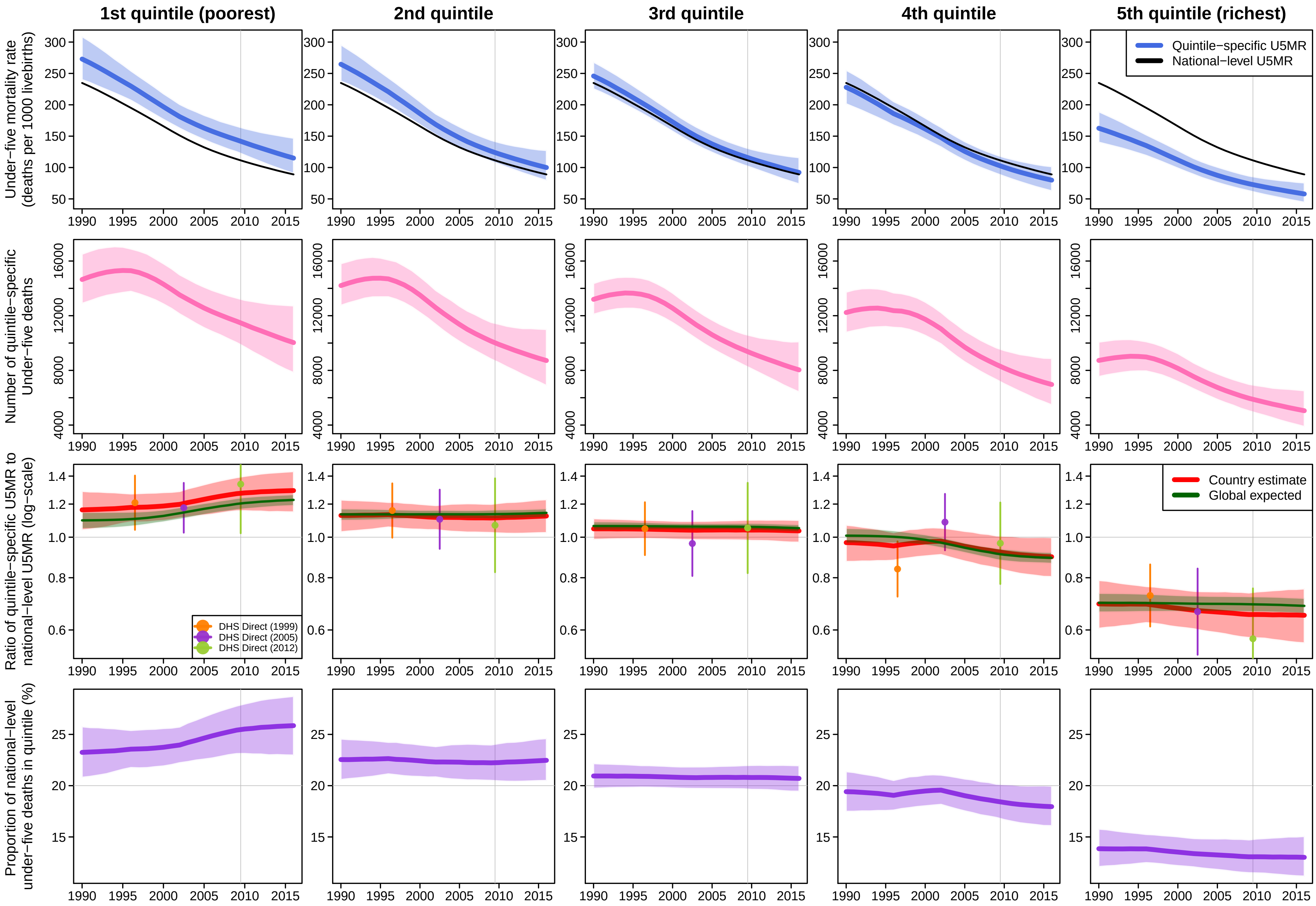
1st quintile (poorest)
2nd quintile
3rd quintile
4th quintile
5th quintile (richest)
Under−five mortality rate (deaths per 1000 livebirths)
Number of quintile−specific Under−five deaths
Ratio of quintile−specific U5MR to national−level U5MR (log−scale)
Proportion of national−level under−five deaths in quintile (%)
Quintile−specific U5MR
National−level U5MR
Country estimate
Global expected
DHS Direct (1999)
DHS Direct (2005)
DHS Direct (2012)

Guinea−Bissau

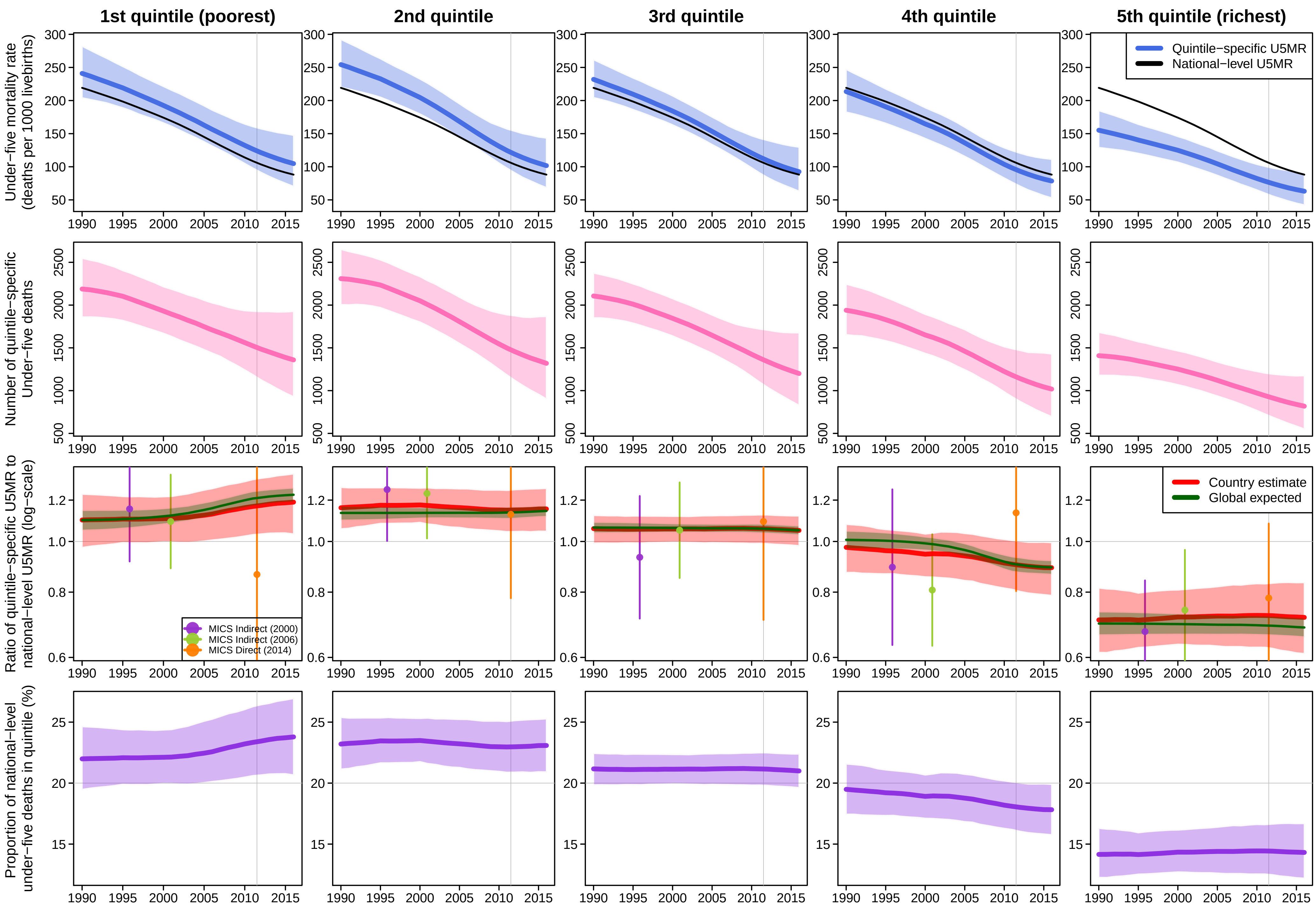

Guyana

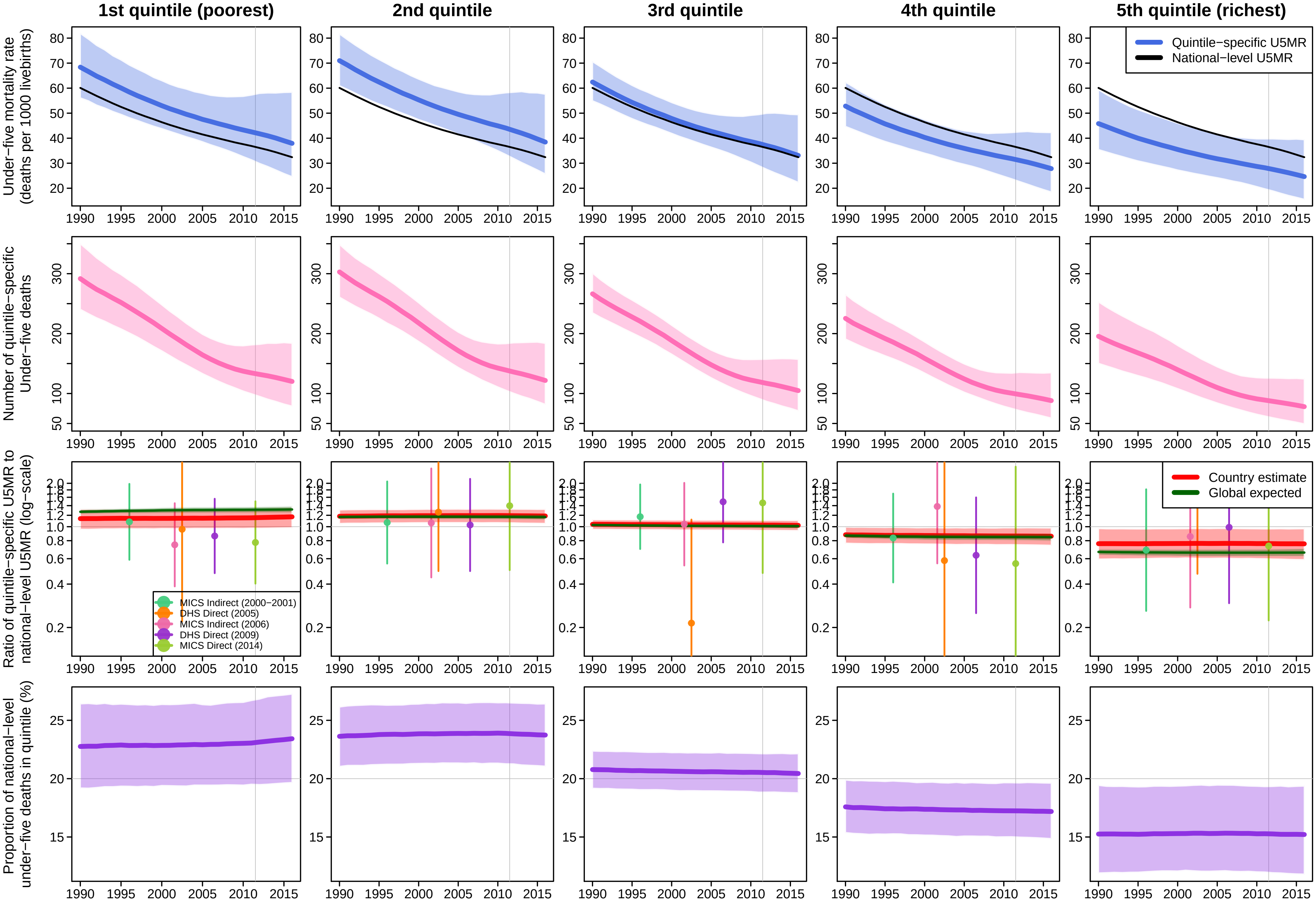
1st quintile (poorest)
2nd quintile
3rd quintile
4th quintile
5th quintile (richest)
Under−five mortality rate (deaths per 1000 livebirths)
Quintile−specific U5MR
National−level U5MR
Number of quintile−specific Under−five deaths
Ratio of quintile−specific U5MR to national−level U5MR (log−scale)
MICS Indirect (2000−2001)
DHS Direct (2005)
MICS Indirect (2006)
DHS Direct (2009)
MICS Direct (2014)
Country estimate
Global expected
Proportion of national−level under−five deaths in quintile (%)

Haiti

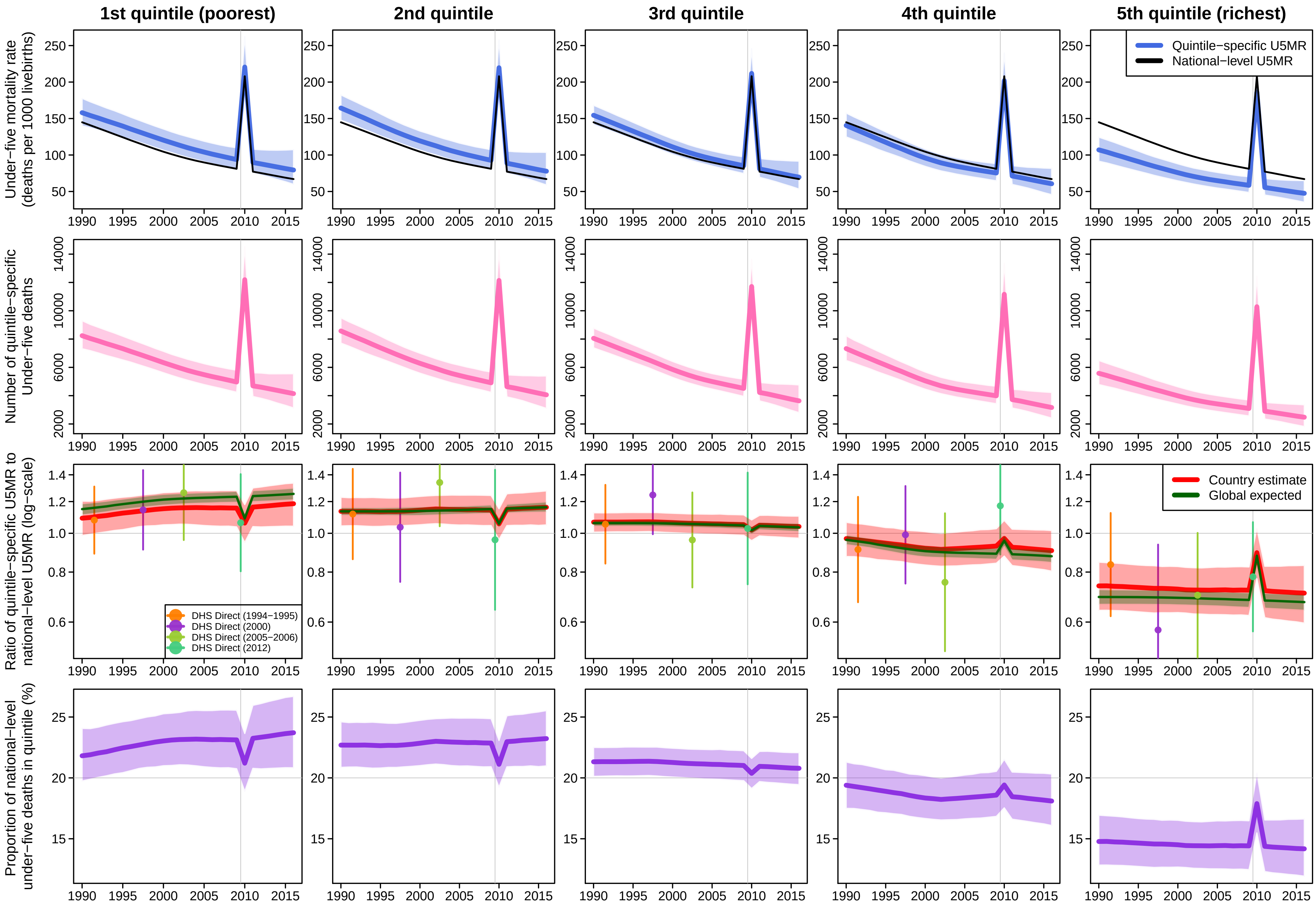
1st quintile (poorest)
2nd quintile
3rd quintile
4th quintile
5th quintile (richest)
Under−five mortality rate (deaths per 1000 livebirths)
Quintile−specific U5MR
National−level U5MR
Number of quintile−specific Under−five deaths
Ratio of quintile−specific U5MR to national−level U5MR (log−scale)
Country estimate
Global expected
DHS Direct (1994−1995)
DHS Direct (2000)
DHS Direct (2005−2006)
DHS Direct (2012)
Proportion of national−level under−five deaths in quintile (%)

Honduras

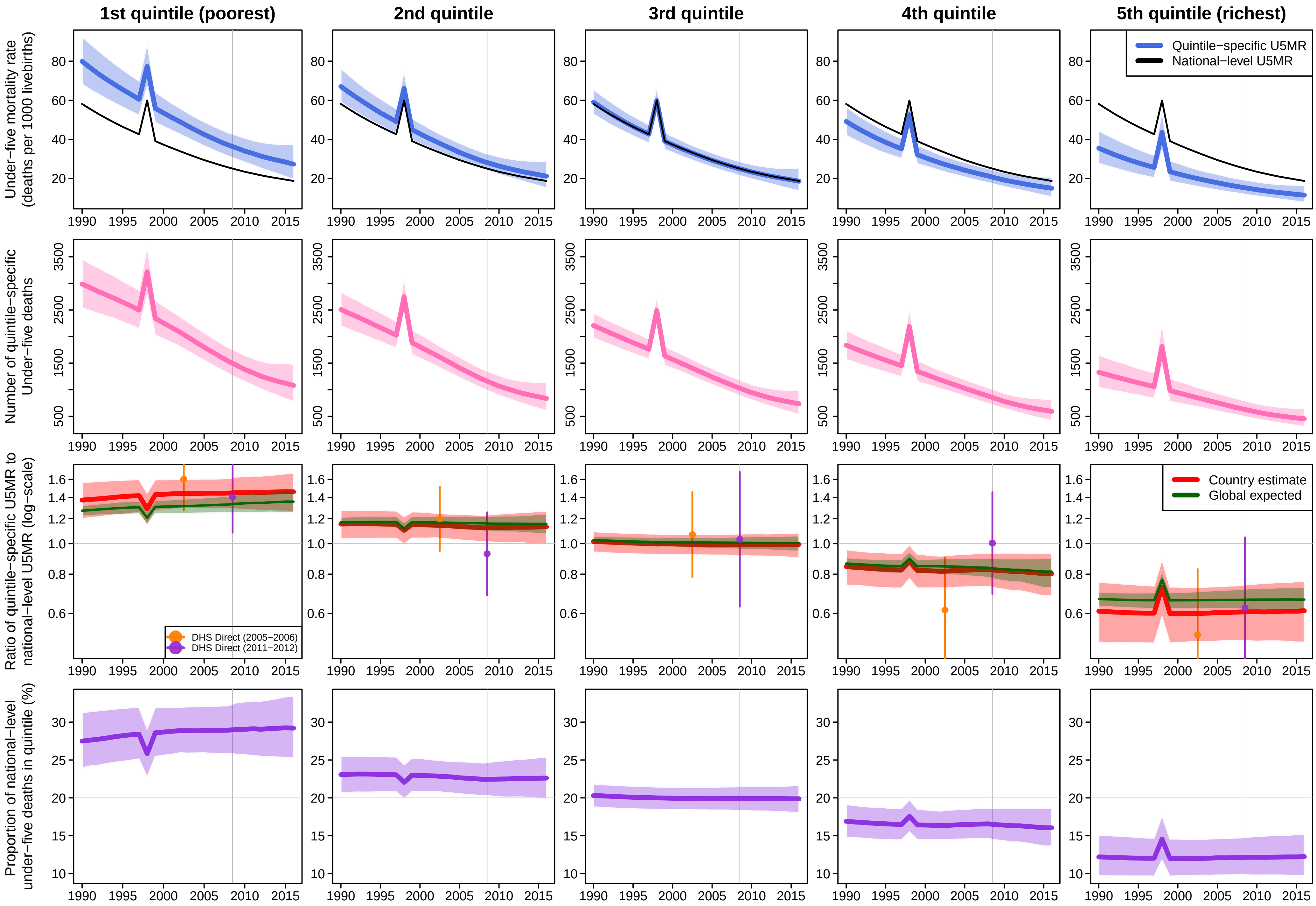
1st quintile (poorest)
2nd quintile
3rd quintile
4th quintile
5th quintile (richest)
Under−five mortality rate (deaths per 1000 livebirths)
Number of quintile−specific Under−five deaths
Ratio of quintile−specific U5MR to national−level U5MR (log−scale)
Proportion of national−level under−five deaths in quintile (%)
Quintile−specific U5MR
National−level U5MR
Country estimate
Global expected
DHS Direct (2005−2006)
DHS Direct (2011−2012)

India

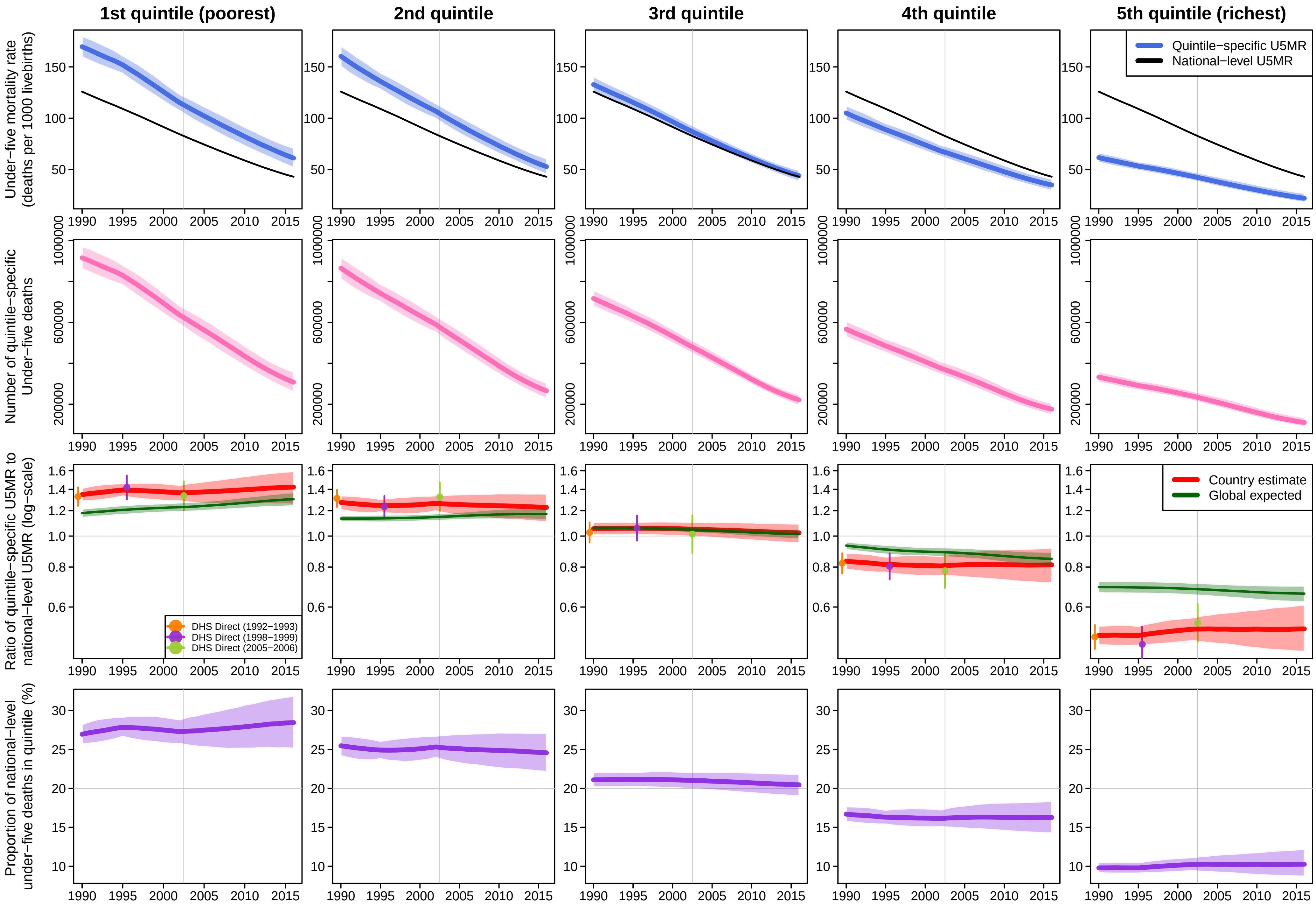
1st quintile (poorest)
2nd quintile
3rd quintile
4th quintile
5th quintile (richest)
Under−five mortality rate (deaths per 1000 livebirths)
Quintile−specific U5MR
National−level U5MR
Number of quintile−specific Under−five deaths
Ratio of quintile−specific U5MR to national−level U5MR (log−scale)
Country estimate
Global expected
DHS Direct (1992−1993)
DHS Direct (1998−1999)
DHS Direct (2005−2006)
Proportion of national−level under−five deaths in quintile (%)

Indonesia

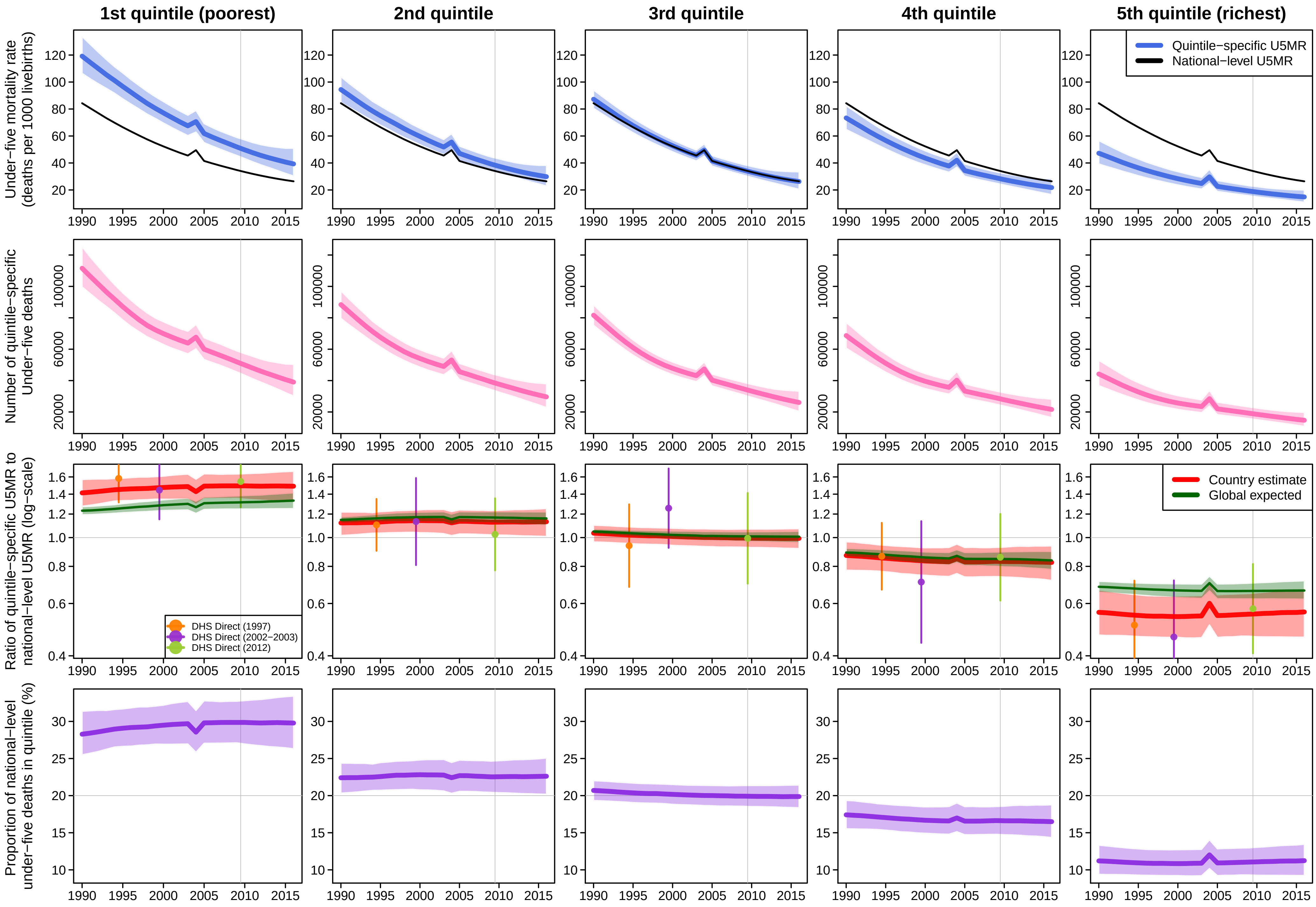

1st quintile (poorest)
2nd quintile
3rd quintile
4th quintile
5th quintile (richest)
Under−five mortality rate (deaths per 1000 livebirths)
Quintile−specific U5MR
National−level U5MR
Number of quintile−specific Under−five deaths
Ratio of quintile−specific U5MR to national−level U5MR (log−scale)
Country estimate
Global expected
DHS Direct (1997)
DHS Direct (2002−2003)
DHS Direct (2012)
Proportion of national−level under−five deaths in quintile (%)

Iraq

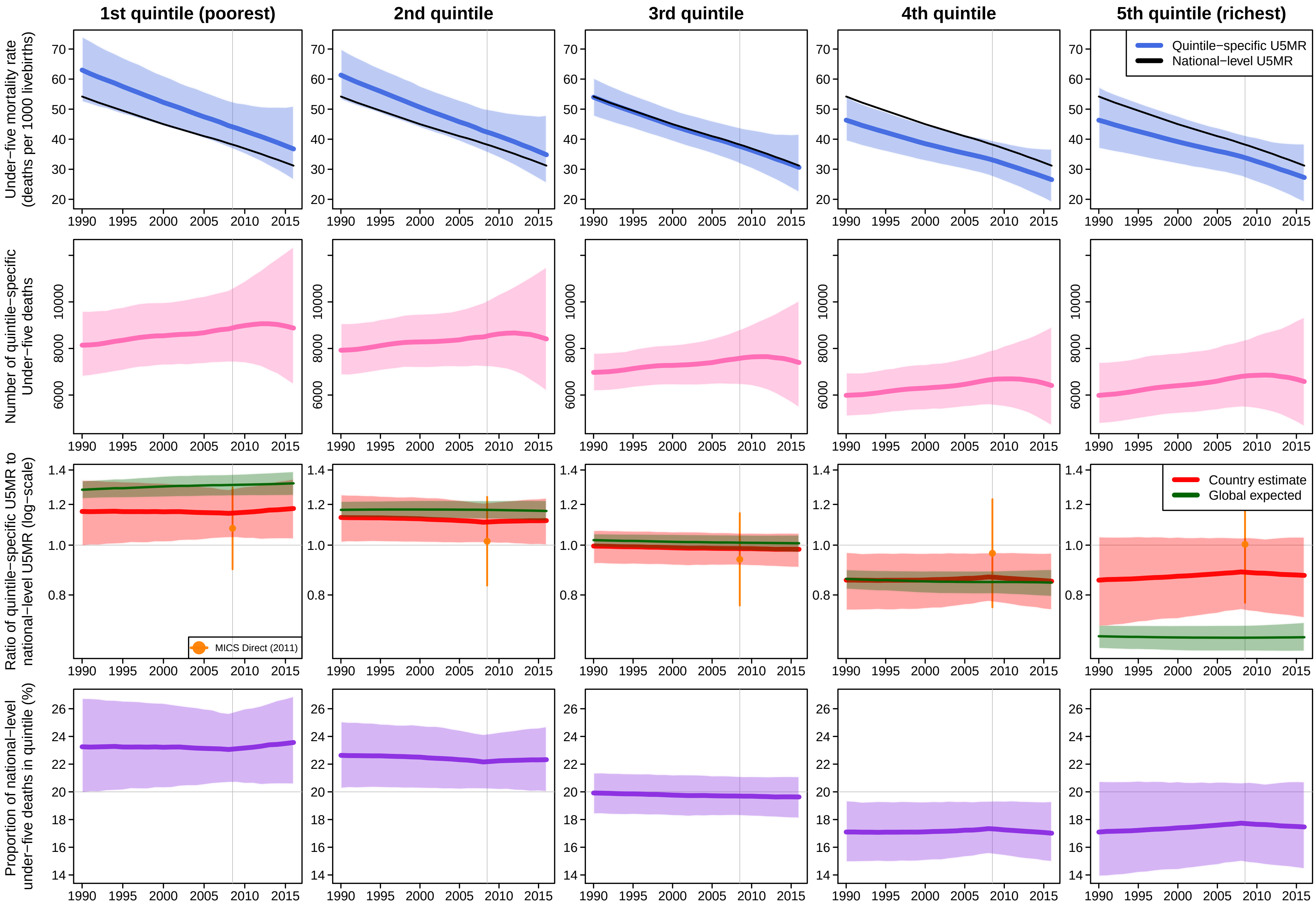
1st quintile (poorest)
2nd quintile
3rd quintile
4th quintile
5th quintile (richest)
Quintile−specific U5MR
National−level U5MR
Under−five mortality rate (deaths per 1000 livebirths)
Number of quintile−specific Under−five deaths
Country estimate
Global expected
MICS Direct (2011)
Ratio of quintile−specific U5MR to national−level U5MR (log−scale)
Proportion of national−level under−five deaths in quintile (%)

Jordan

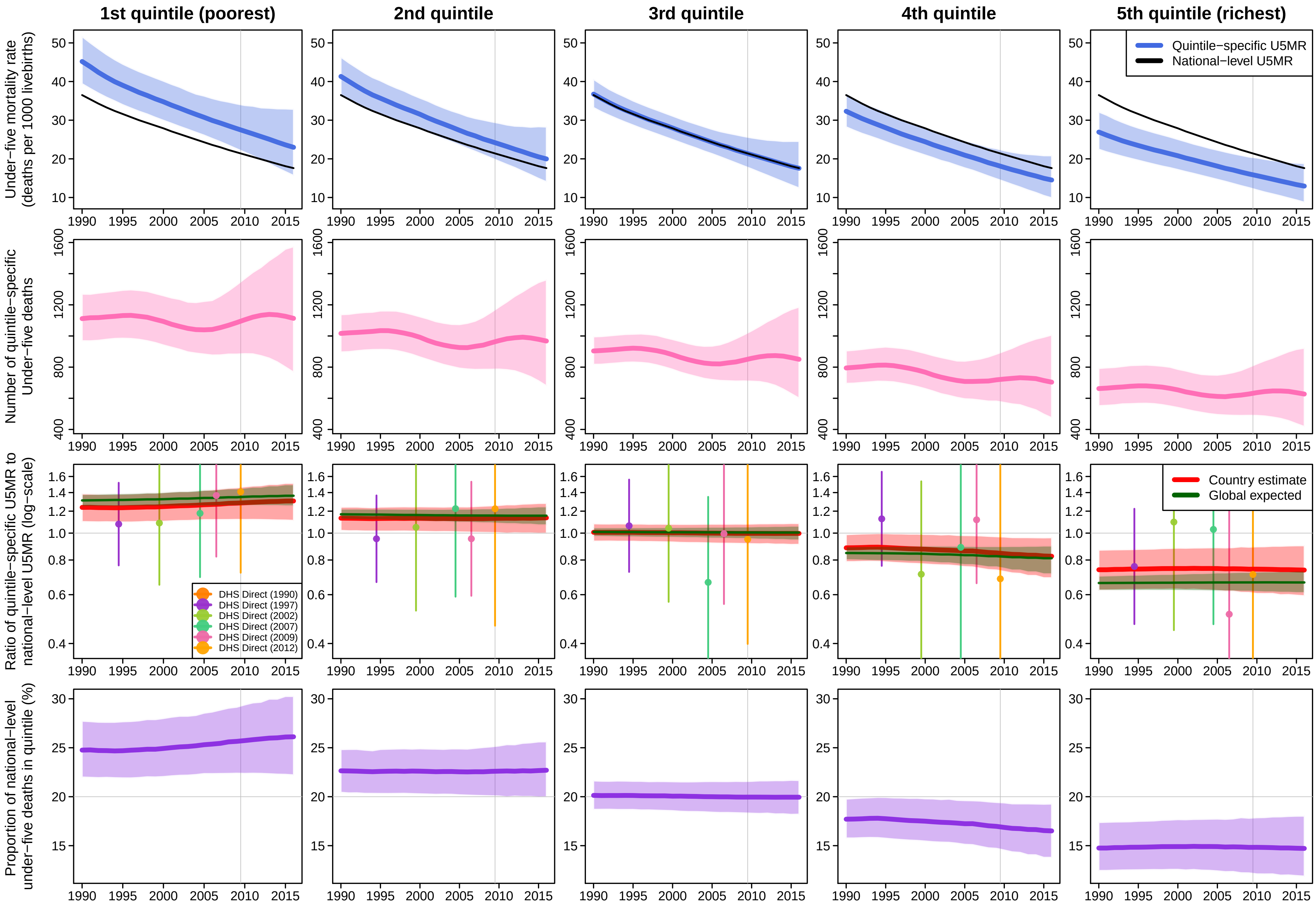
1st quintile (poorest)
2nd quintile
3rd quintile
4th quintile
5th quintile (richest)
Under−five mortality rate (deaths per 1000 livebirths)
Quintile−specific U5MR
National−level U5MR
Number of quintile−specific Under−five deaths
Ratio of quintile−specific U5MR to national−level U5MR (log−scale)
Country estimate
Global expected
DHS Direct (1990)
DHS Direct (1997)
DHS Direct (2002)
DHS Direct (2007)
DHS Direct (2009)
DHS Direct (2012)
Proportion of national−level under−five deaths in quintile (%)

Kazakhstan

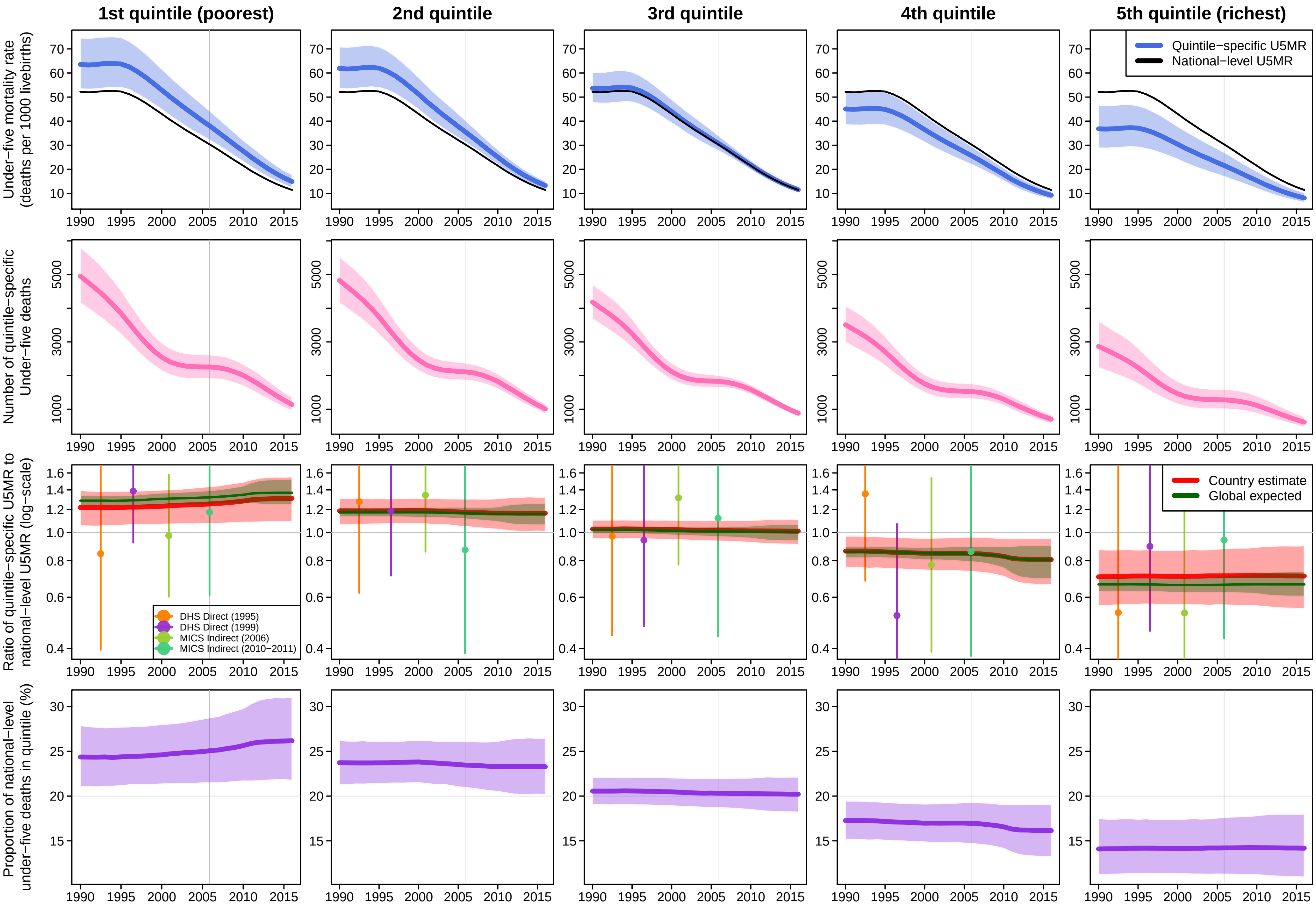

1st quintile (poorest)
2nd quintile
3rd quintile
4th quintile
5th quintile (richest)
Under−five mortality rate (deaths per 1000 livebirths)
Quintile−specific U5MR
National−level U5MR
Number of quintile−specific Under−five deaths
Ratio of quintile−specific U5MR to national−level U5MR (log−scale)
DHS Direct (1995)
DHS Direct (1999)
MICS Indirect (2006)
MICS Indirect (2010−2011)
Country estimate
Global expected
Proportion of national−level under−five deaths in quintile (%)

Kenya

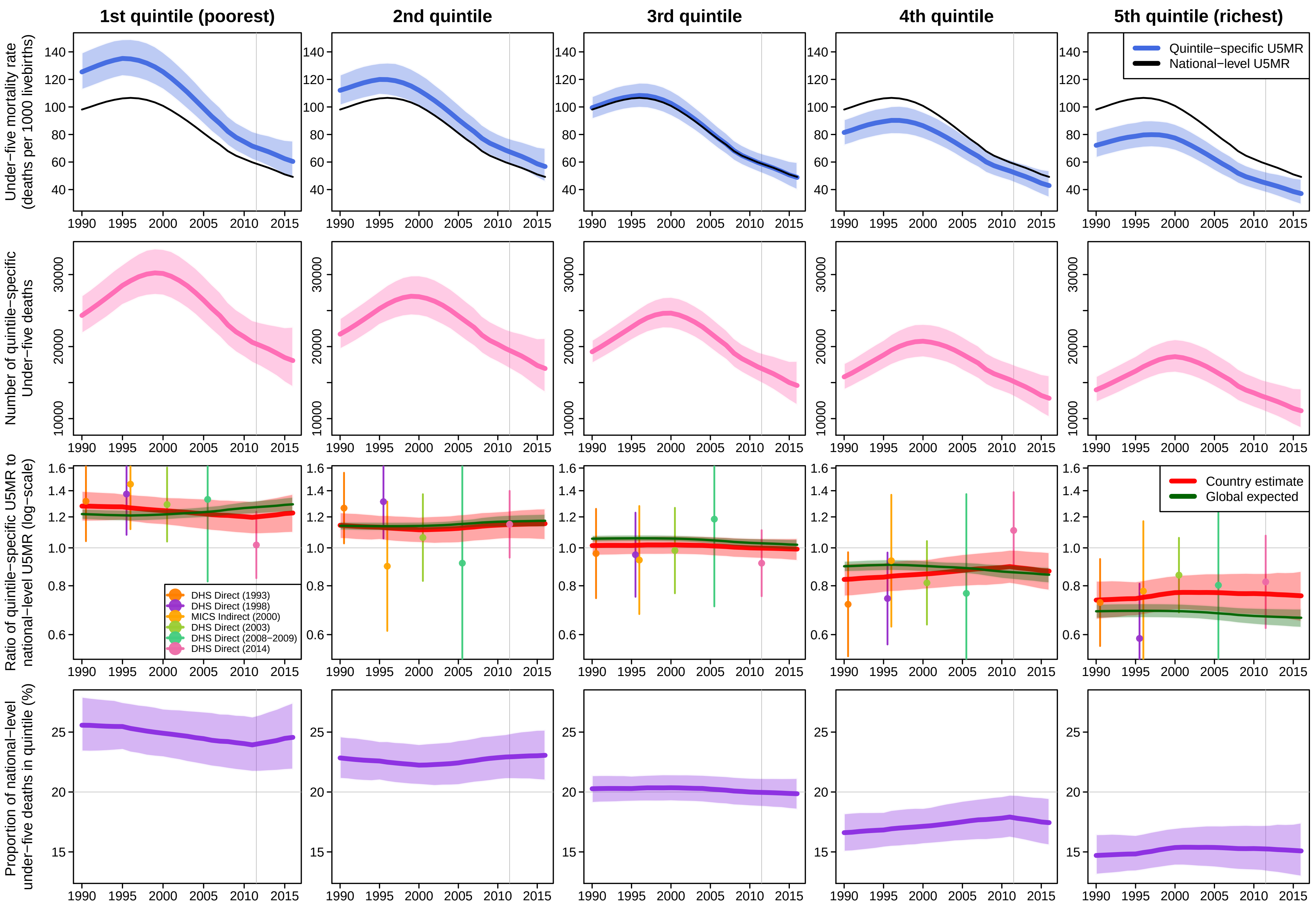

1st quintile (poorest)
2nd quintile
3rd quintile
4th quintile
5th quintile (richest)
Under−five mortality rate (deaths per 1000 livebirths)
Quintile−specific U5MR
National−level U5MR
Number of quintile−specific Under−five deaths
Ratio of quintile−specific U5MR to national−level U5MR (log−scale)
DHS Direct (1993)
DHS Direct (1998)
MICS Indirect (2000)
DHS Direct (2003)
DHS Direct (2008−2009)
DHS Direct (2014)
Country estimate
Global expected
Proportion of national−level under−five deaths in quintile (%)

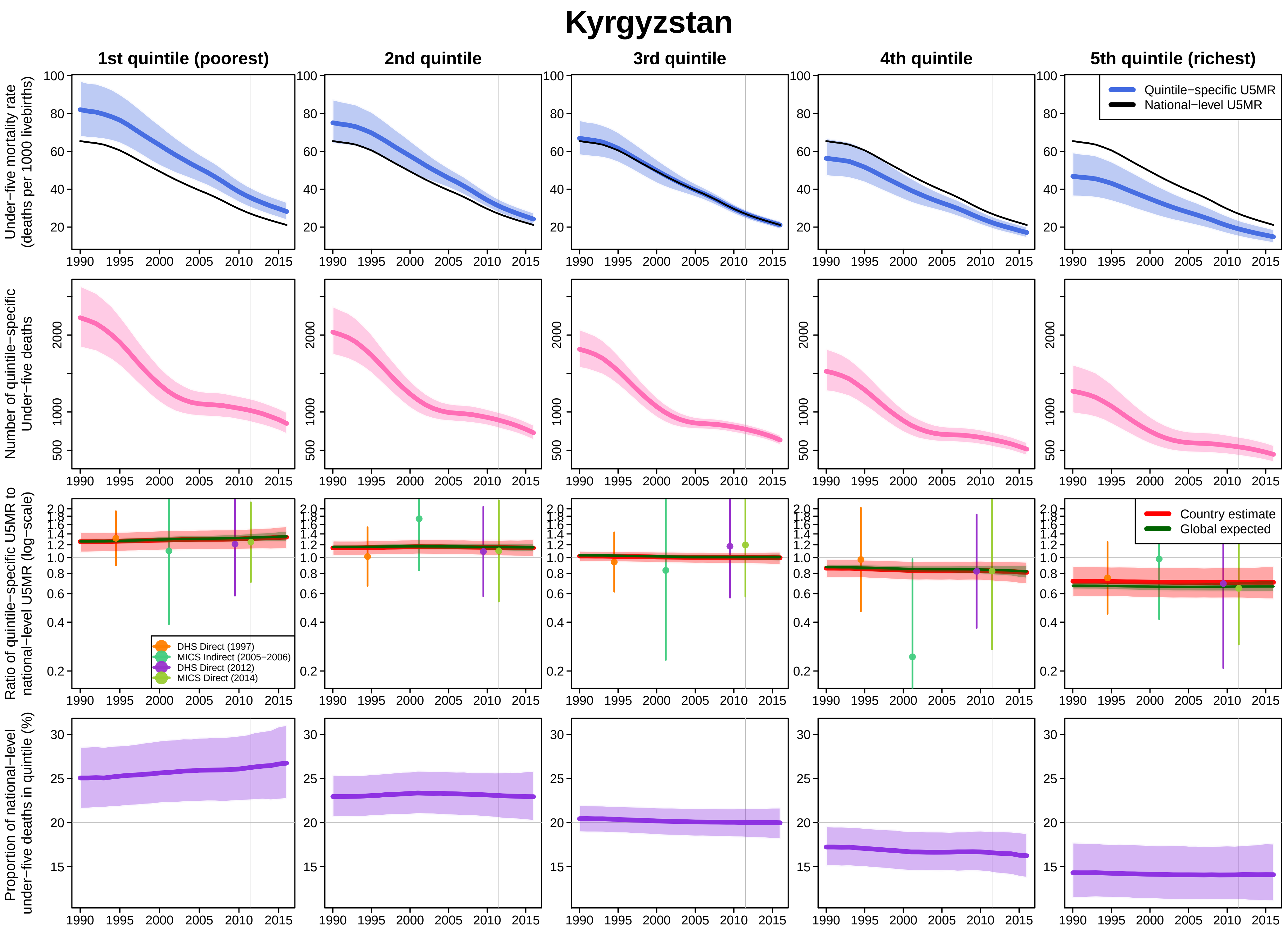
Kyrgyzstan
1st quintile (poorest)
2nd quintile
3rd quintile
4th quintile
5th quintile (richest)
Quintile−specific U5MR
National−level U5MR
Under−five mortality rate (deaths per 1000 livebirths)
Number of quintile−specific Under−five deaths
Ratio of quintile−specific U5MR to national−level U5MR (log−scale)
Country estimate
Global expected
DHS Direct (1997)
MICS Indirect (2005−2006)
DHS Direct (2012)
MICS Direct (2014)
Proportion of national−level under−five deaths in quintile (%)

Lao People's Democratic Republic

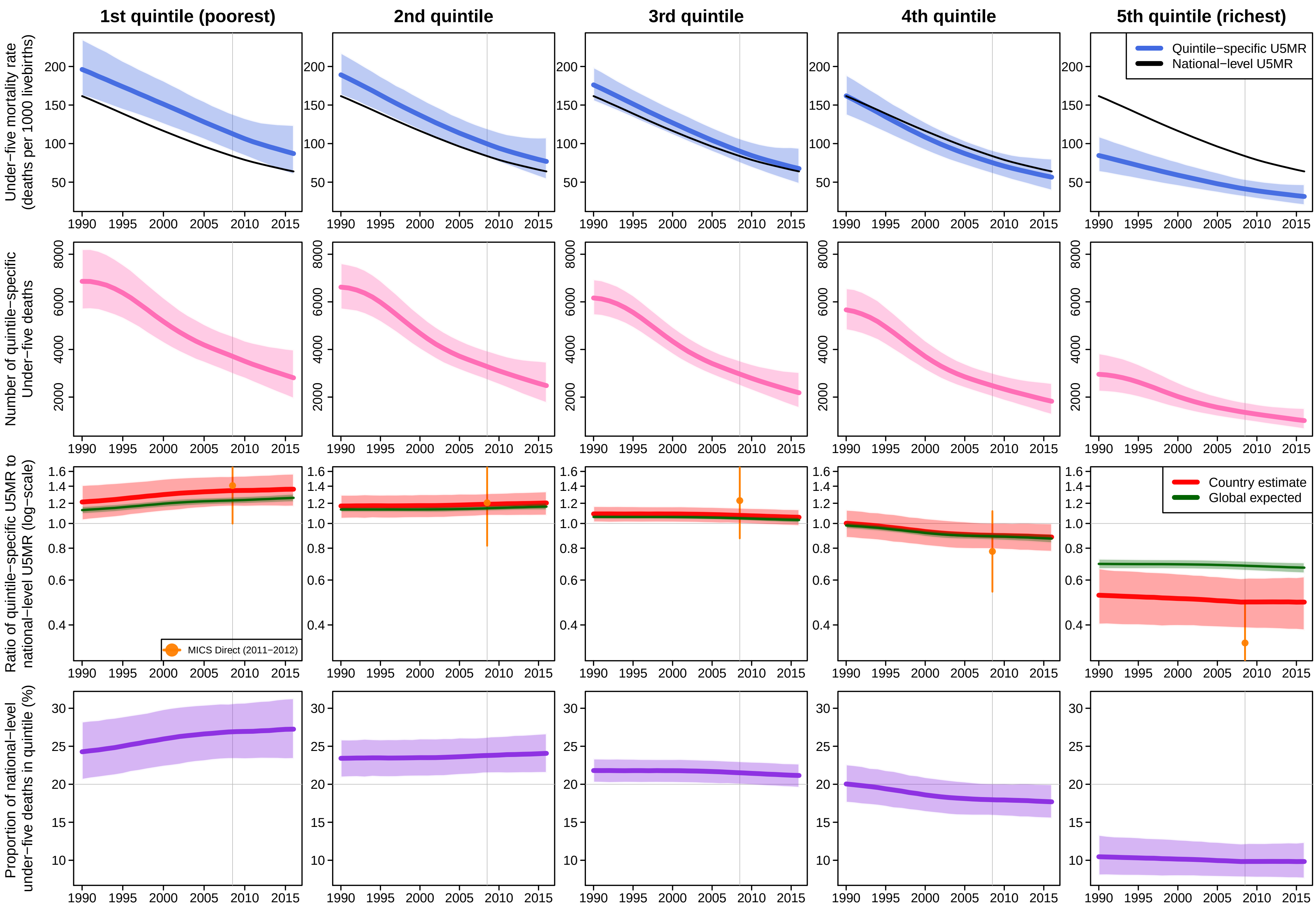

Lesotho

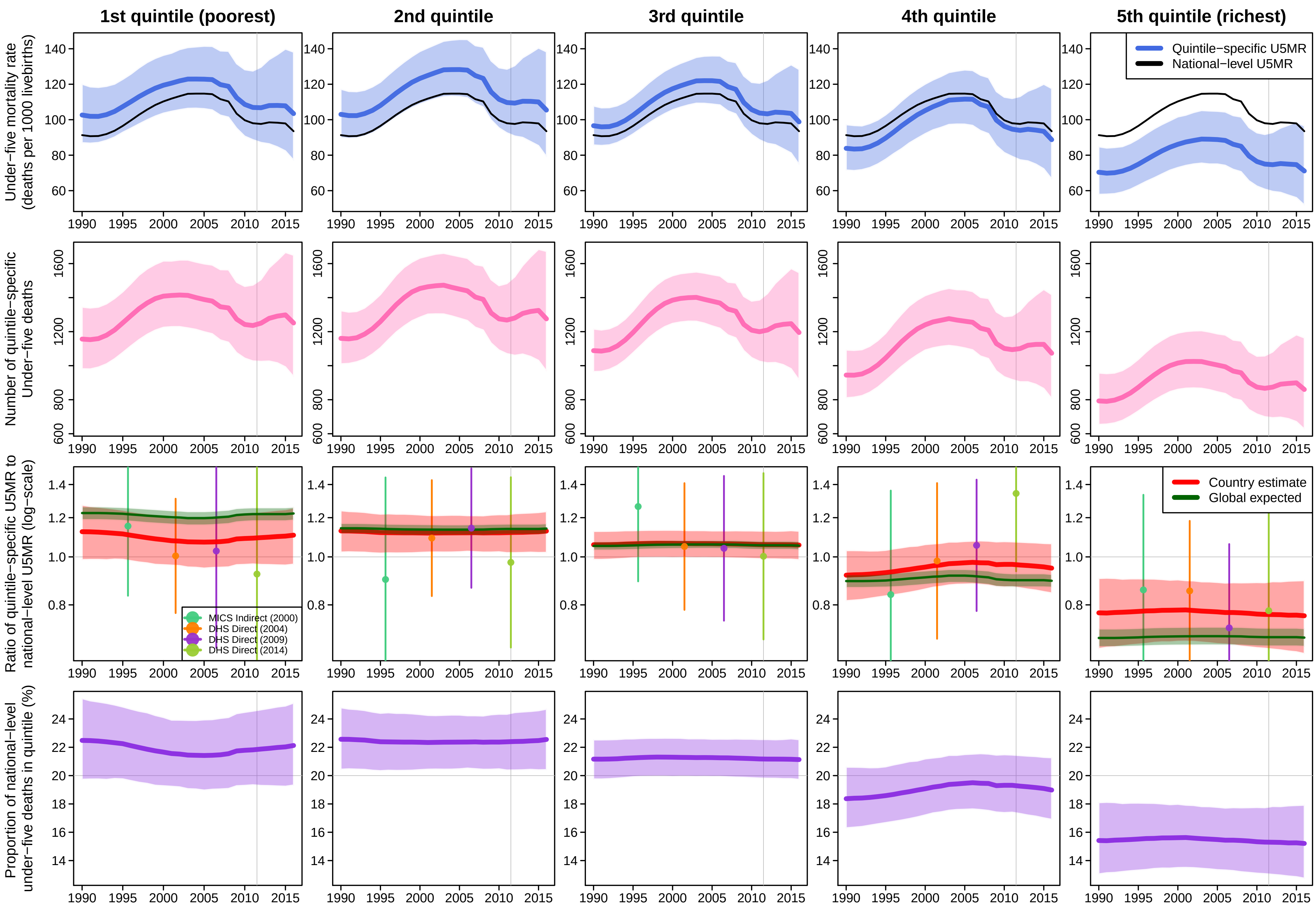

1st quintile (poorest)
2nd quintile
3rd quintile
4th quintile
5th quintile (richest)
Under−five mortality rate (deaths per 1000 livebirths)
Quintile−specific U5MR
National−level U5MR
Number of quintile−specific Under−five deaths
Ratio of quintile−specific U5MR to national−level U5MR (log−scale)
MICS Indirect (2000)
DHS Direct (2004)
DHS Direct (2009)
DHS Direct (2014)
Country estimate
Global expected
Proportion of national−level under−five deaths in quintile (%)

Liberia

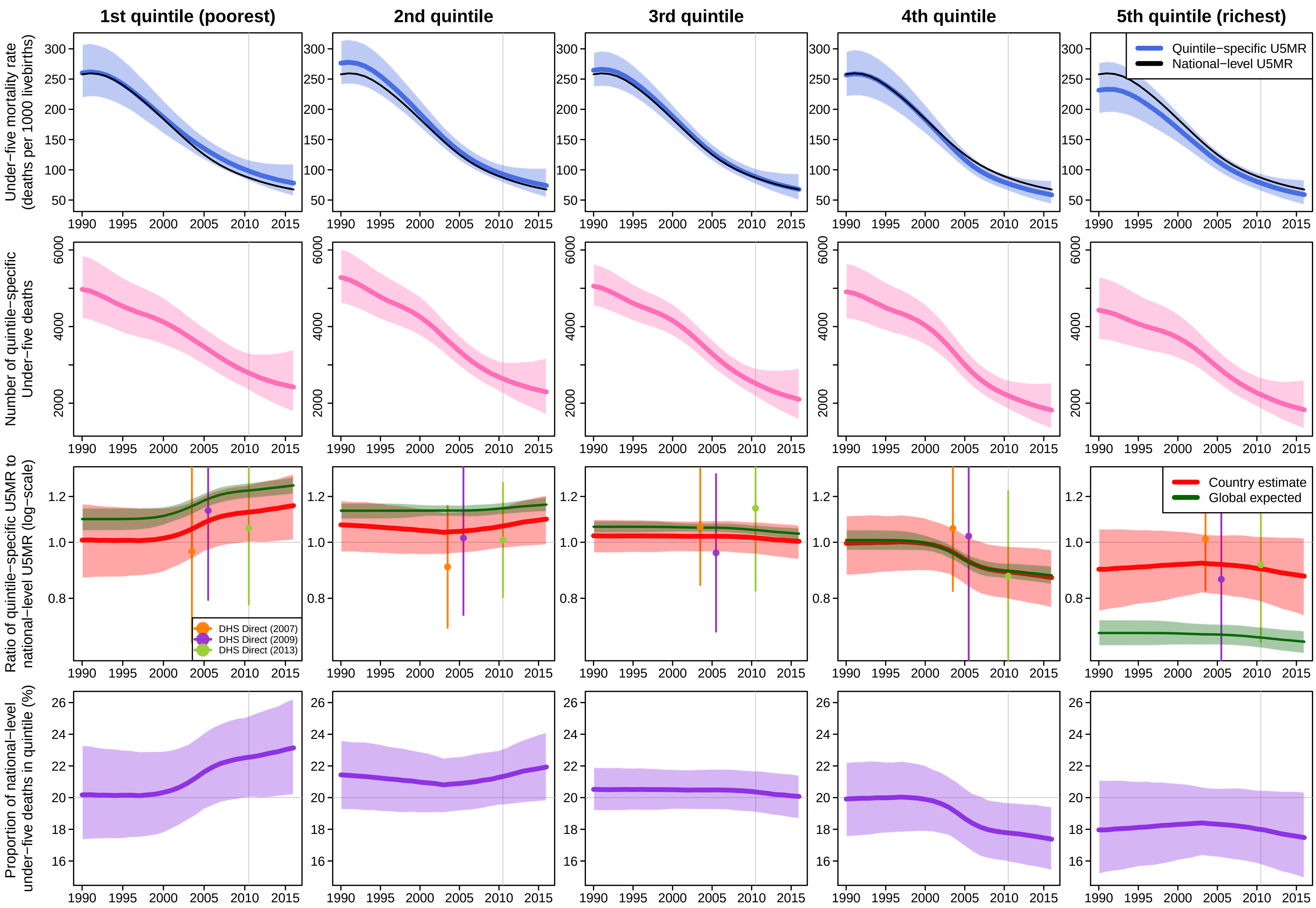
1st quintile (poorest)
2nd quintile
3rd quintile
4th quintile
5th quintile (richest)
Under−five mortality rate (deaths per 1000 livebirths)
Quintile−specific U5MR
National−level U5MR
Number of quintile−specific Under−five deaths
Ratio of quintile−specific U5MR to national−level U5MR (log−scale)
Country estimate
Global expected
DHS Direct (2007)
DHS Direct (2009)
DHS Direct (2013)
Proportion of national−level under−five deaths in quintile (%)

Madagascar

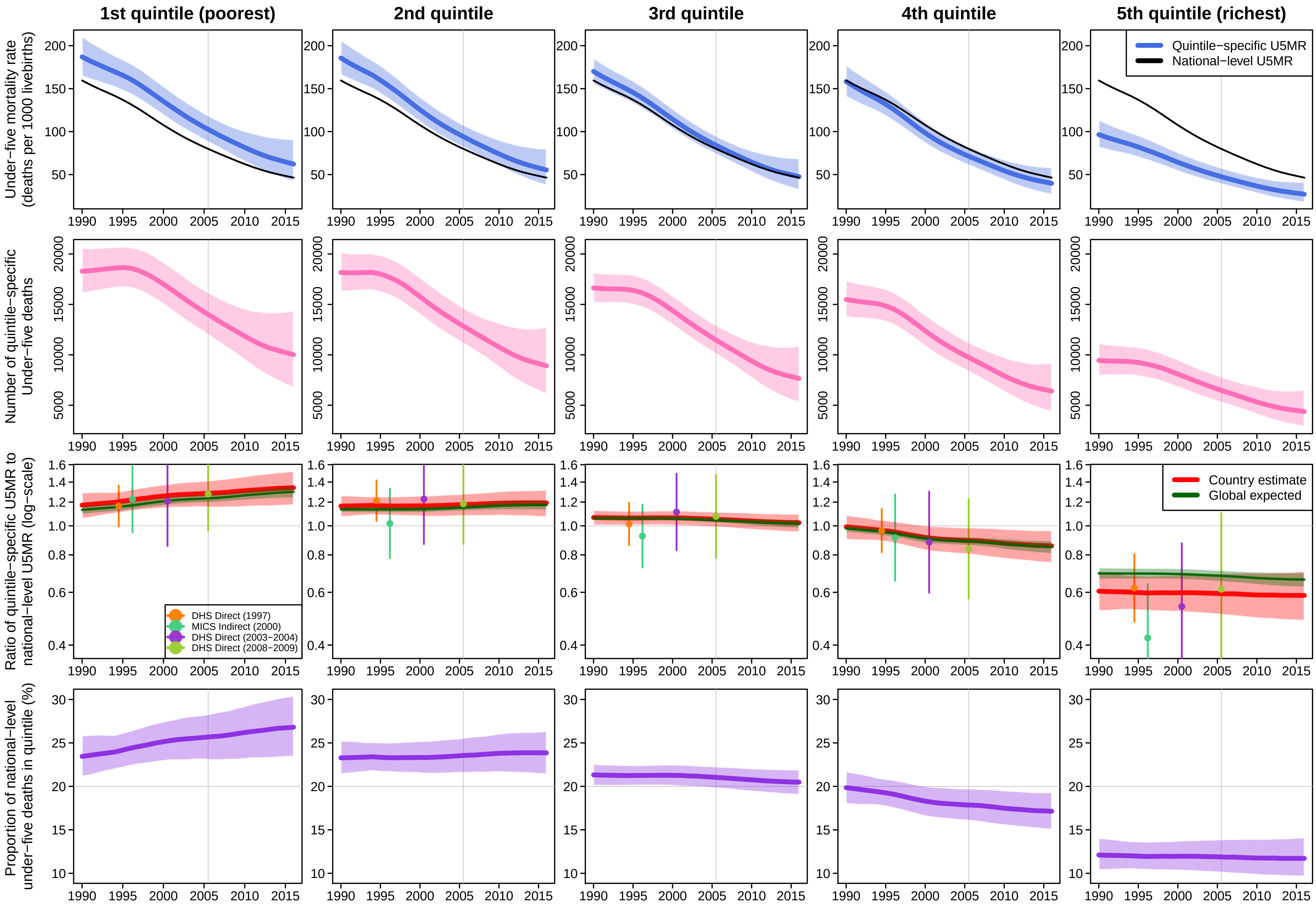
1st quintile (poorest)
2nd quintile
3rd quintile
4th quintile
5th quintile (richest)
Under−five mortality rate (deaths per 1000 livebirths)
Quintile−specific U5MR
National−level U5MR
Number of quintile−specific Under−five deaths
Ratio of quintile−specific U5MR to national−level U5MR (log−scale)
Country estimate
Global expected
DHS Direct (1997)
MICS Indirect (2000)
DHS Direct (2003−2004)
DHS Direct (2008−2009)
Proportion of national−level under−five deaths in quintile (%)
1990
1995
2000
2005
2010
2015

Malawi

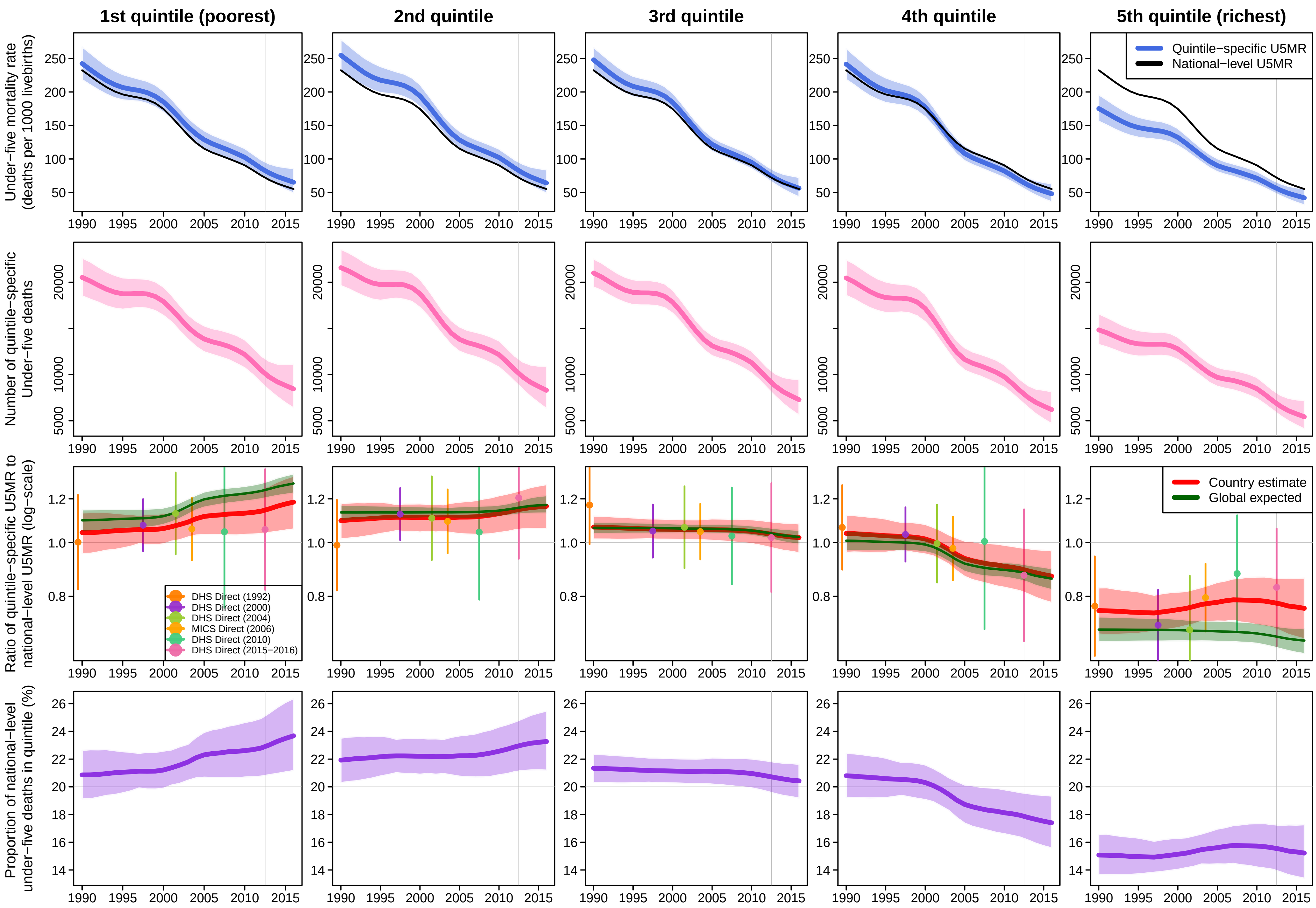
1st quintile (poorest)
2nd quintile
3rd quintile
4th quintile
5th quintile (richest)
Under−five mortality rate (deaths per 1000 livebirths)
Number of quintile−specific Under−five deaths
Ratio of quintile−specific U5MR to national−level U5MR (log−scale)
Proportion of national−level under−five deaths in quintile (%)
Quintile−specific U5MR
National−level U5MR
Country estimate
Global expected
DHS Direct (1992)
DHS Direct (2000)
DHS Direct (2004)
MICS Direct (2006)
DHS Direct (2010)
DHS Direct (2015−2016)

Maldives

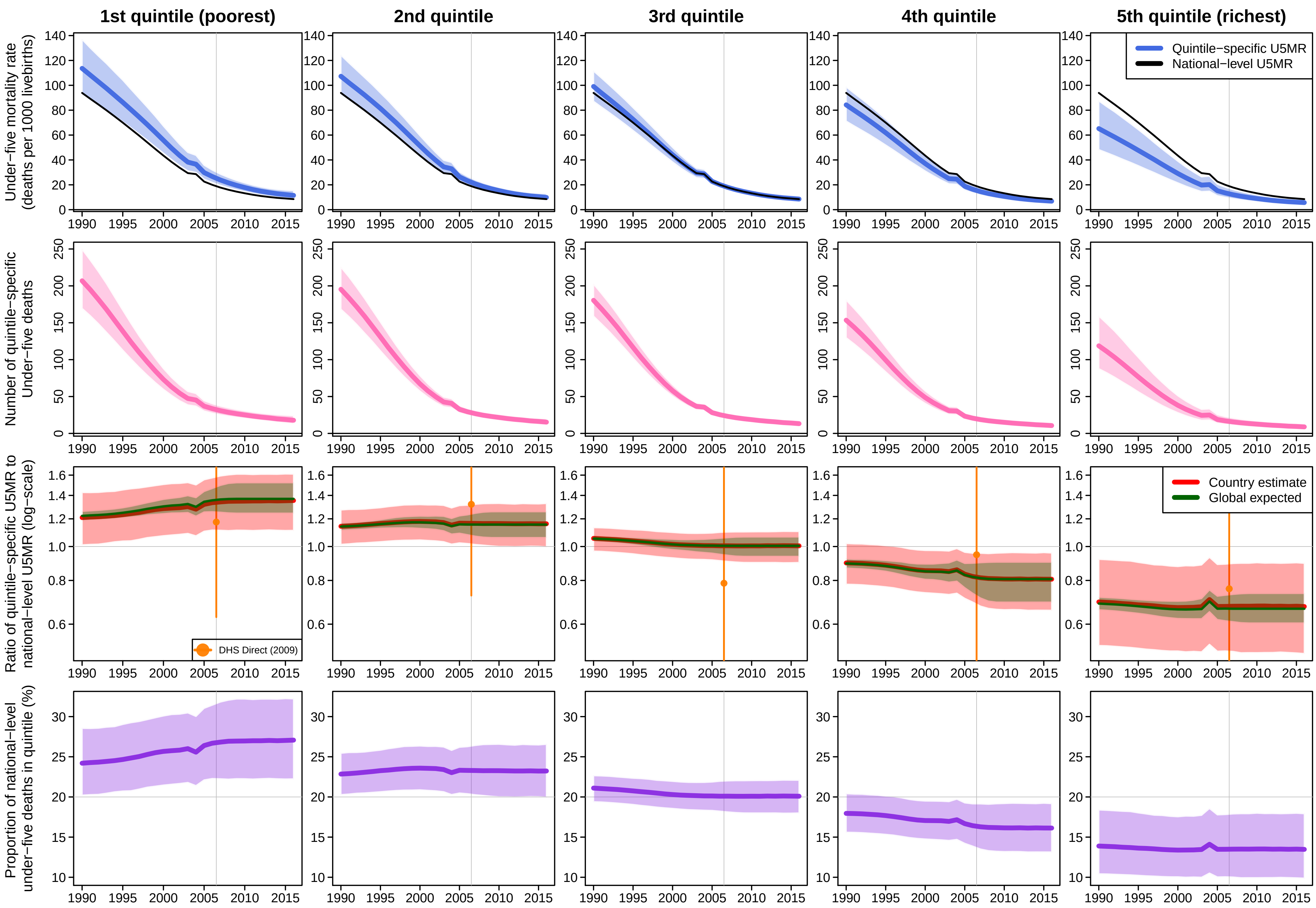

1st quintile (poorest)
2nd quintile
3rd quintile
4th quintile
5th quintile (richest)
Under−five mortality rate (deaths per 1000 livebirths)
Quintile−specific U5MR
National−level U5MR
Number of quintile−specific Under−five deaths
Ratio of quintile−specific U5MR to national−level U5MR (log−scale)
Country estimate
Global expected
DHS Direct (2009)
Proportion of national−level under−five deaths in quintile (%)

Mali

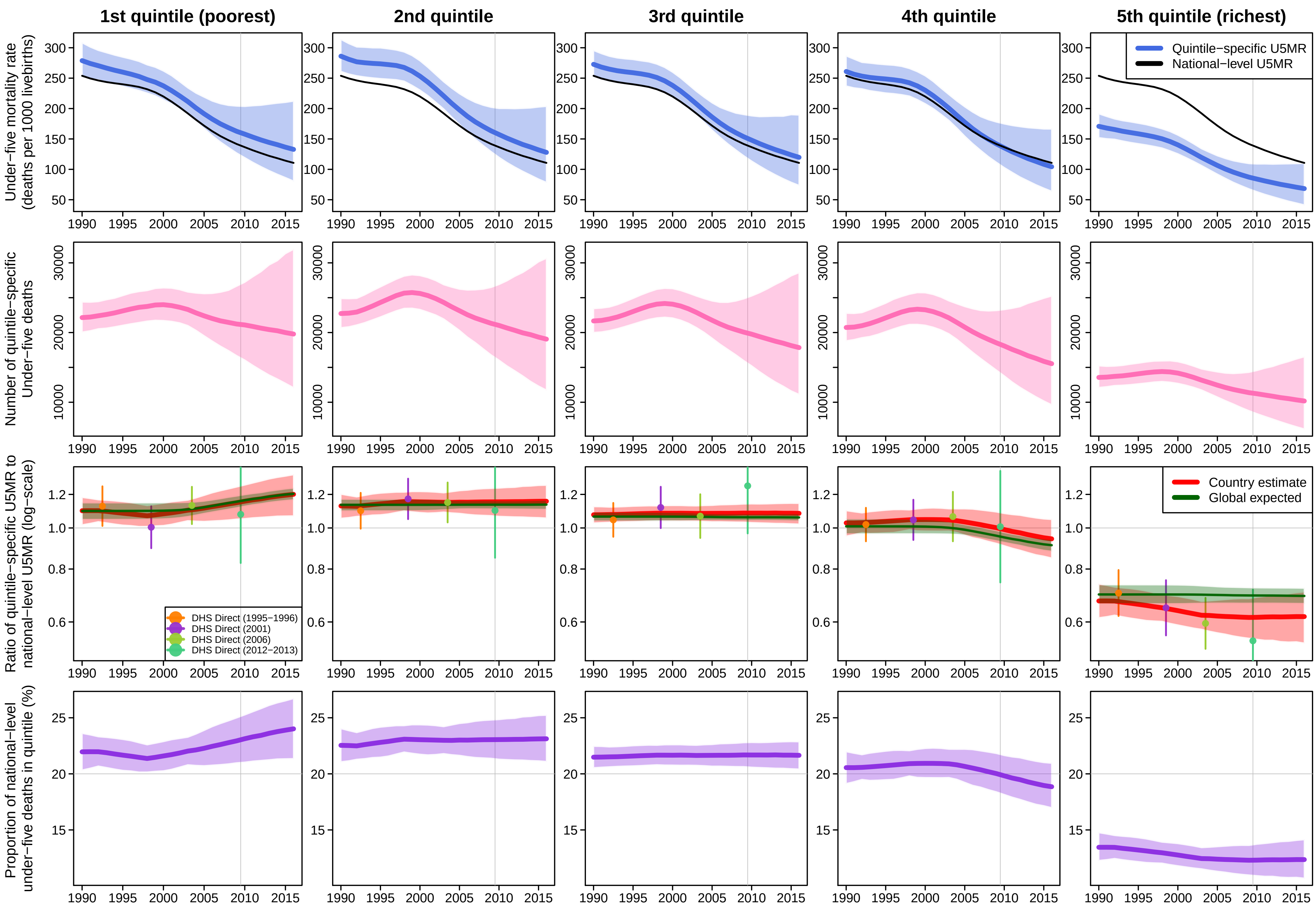
1st quintile (poorest)
2nd quintile
3rd quintile
4th quintile
5th quintile (richest)
Under−five mortality rate (deaths per 1000 livebirths)
Quintile−specific U5MR
National−level U5MR
Number of quintile−specific Under−five deaths
Ratio of quintile−specific U5MR to national−level U5MR (log−scale)
Country estimate
Global expected
DHS Direct (1995−1996)
DHS Direct (2001)
DHS Direct (2006)
DHS Direct (2012−2013)
Proportion of national−level under−five deaths in quintile (%)

Mauritania

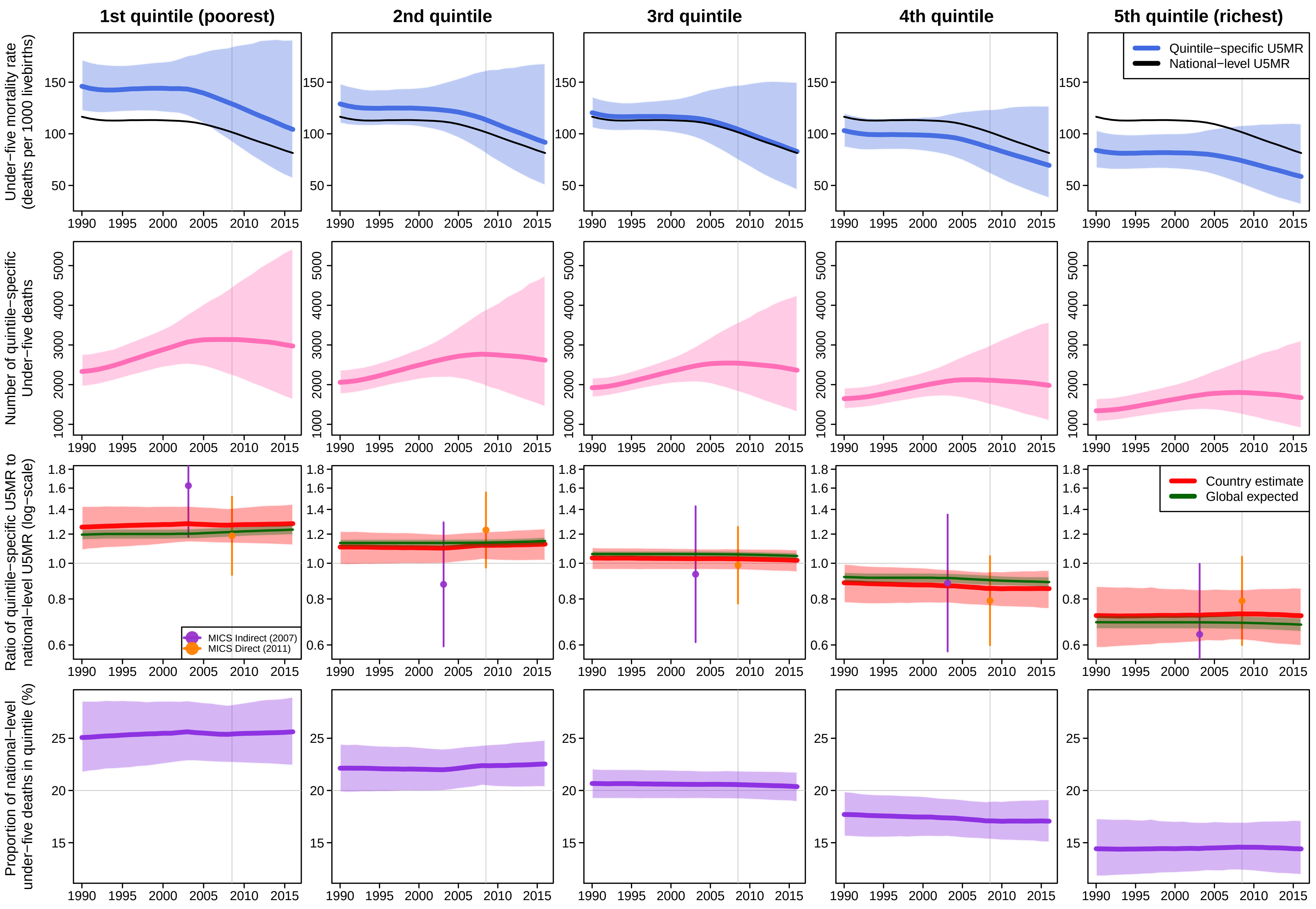

1st quintile (poorest)
2nd quintile
3rd quintile
4th quintile
5th quintile (richest)
Under−five mortality rate (deaths per 1000 livebirths)
Quintile−specific U5MR
National−level U5MR
Number of quintile−specific Under−five deaths
Ratio of quintile−specific U5MR to national−level U5MR (log−scale)
Country estimate
Global expected
MICS Indirect (2007)
MICS Direct (2011)
Proportion of national−level under−five deaths in quintile (%)

Mongolia

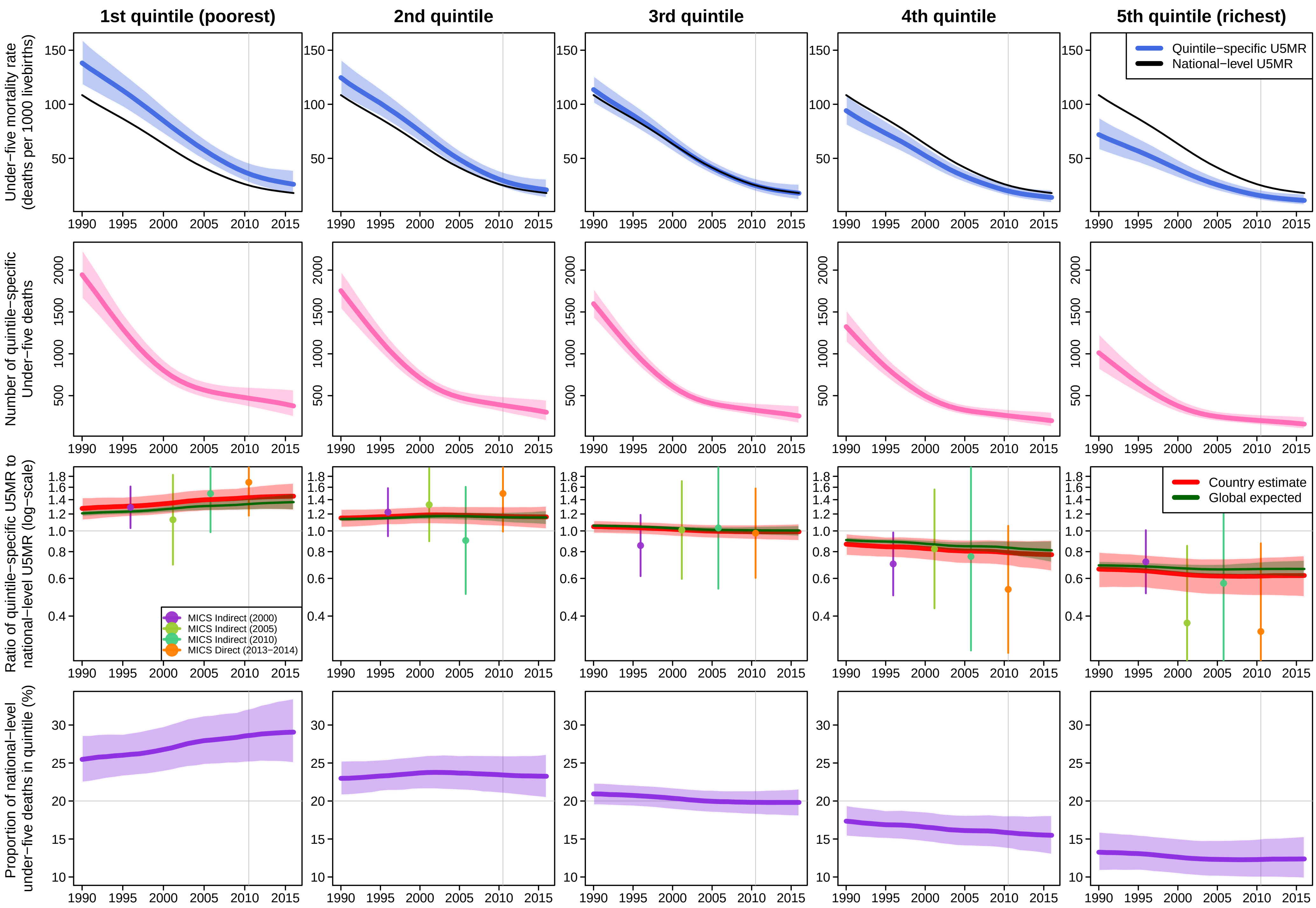
1st quintile (poorest)
2nd quintile
3rd quintile
4th quintile
5th quintile (richest)
Under−five mortality rate (deaths per 1000 livebirths)
Quintile−specific U5MR
National−level U5MR
Number of quintile−specific Under−five deaths
Ratio of quintile−specific U5MR to national−level U5MR (log−scale)
Country estimate
Global expected
MICS Indirect (2000)
MICS Indirect (2005)
MICS Indirect (2010)
MICS Direct (2013−2014)
Proportion of national−level under−five deaths in quintile (%)

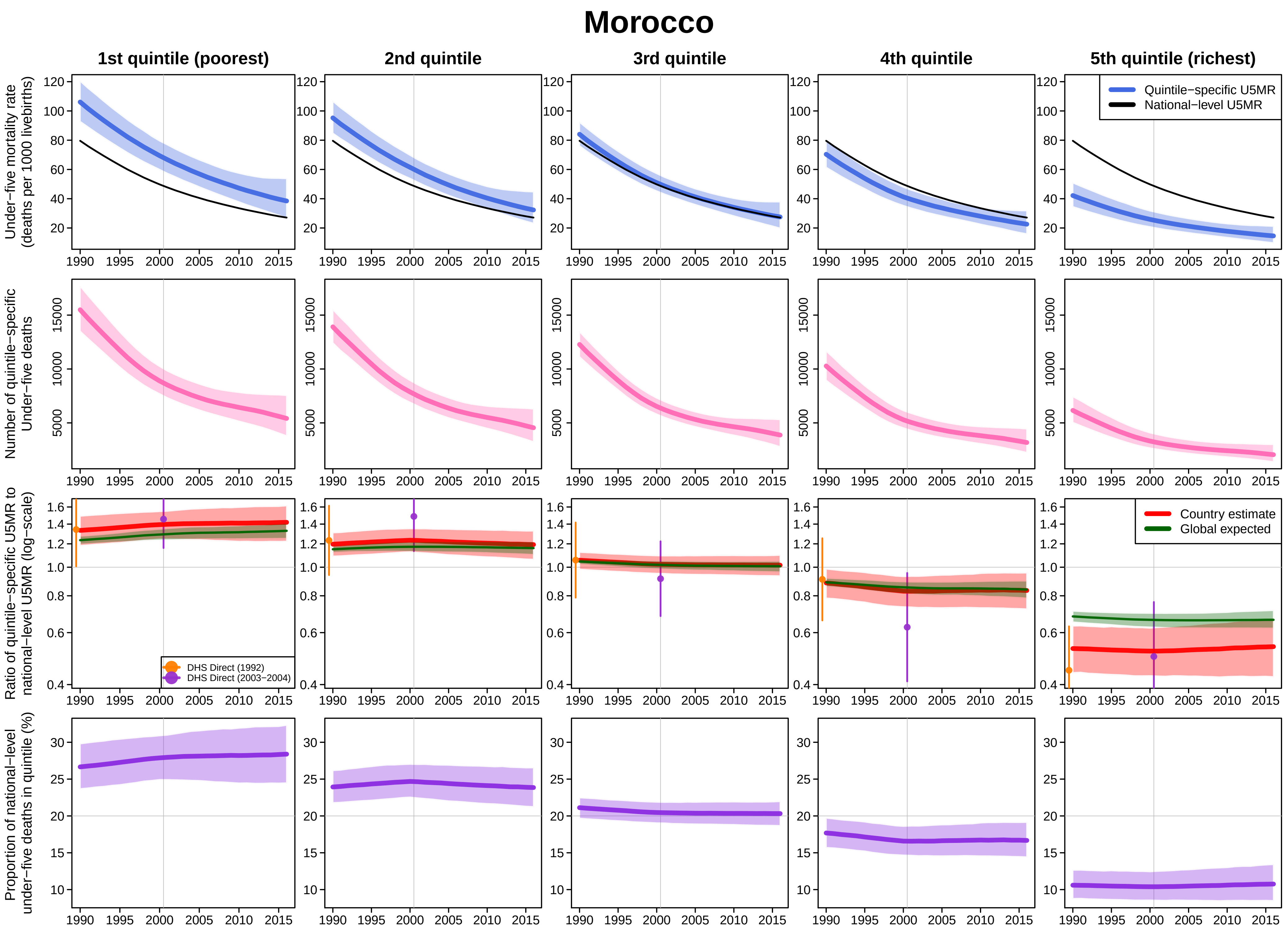

Morocco
1st quintile (poorest)
2nd quintile
3rd quintile
4th quintile
5th quintile (richest)
Under−five mortality rate (deaths per 1000 livebirths)
Quintile−specific U5MR
National−level U5MR
Number of quintile−specific Under−five deaths
Ratio of quintile−specific U5MR to national−level U5MR (log−scale)
Country estimate
Global expected
DHS Direct (1992)
DHS Direct (2003−2004)
Proportion of national−level under−five deaths in quintile (%)

Mozambique

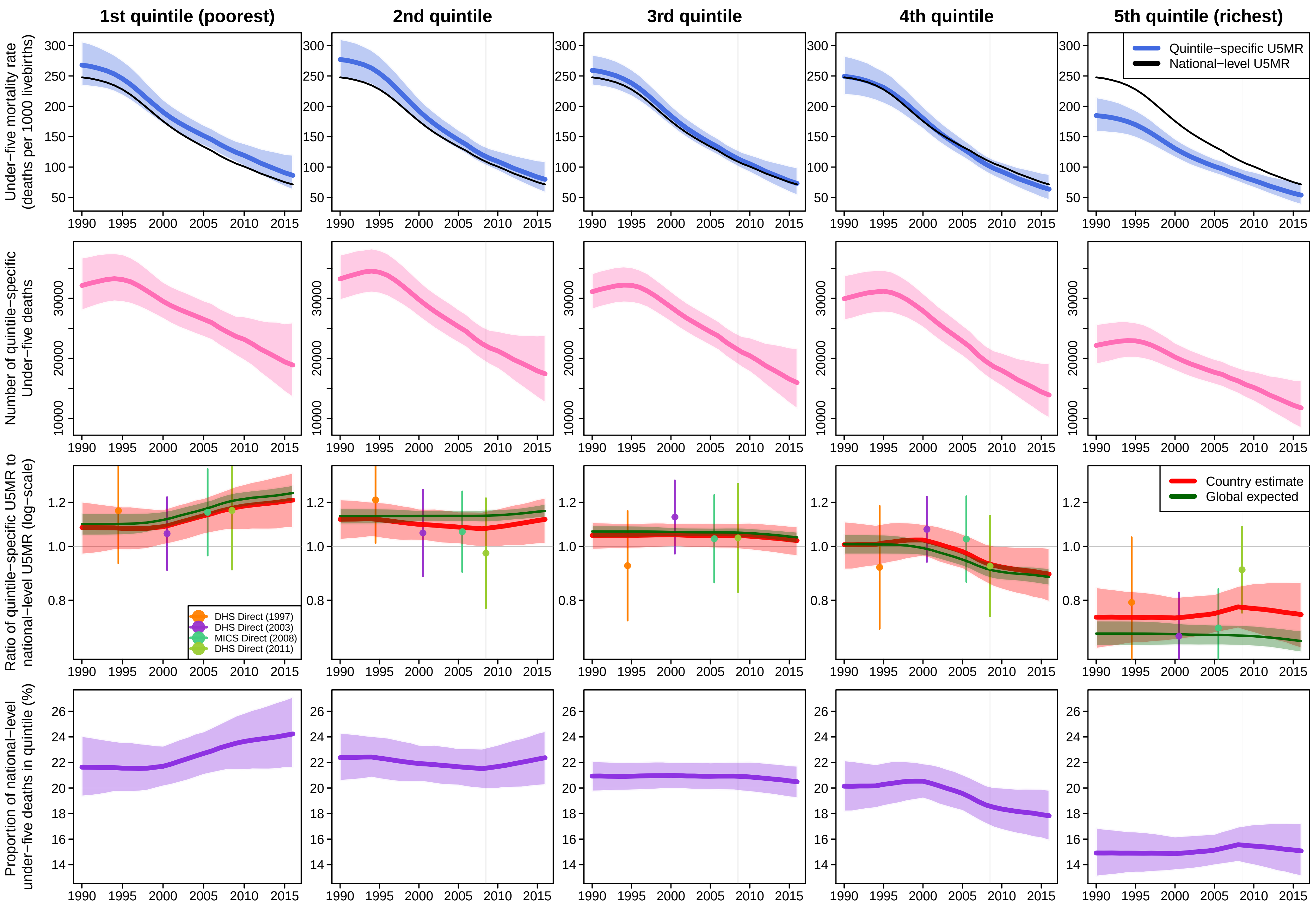

1st quintile (poorest)
2nd quintile
3rd quintile
4th quintile
5th quintile (richest)
Under−five mortality rate (deaths per 1000 livebirths)
Number of quintile−specific Under−five deaths
Ratio of quintile−specific U5MR to national−level U5MR (log−scale)
Proportion of national−level under−five deaths in quintile (%)
Quintile−specific U5MR
National−level U5MR
Country estimate
Global expected
DHS Direct (1997)
DHS Direct (2003)
MICS Direct (2008)
DHS Direct (2011)

Myanmar

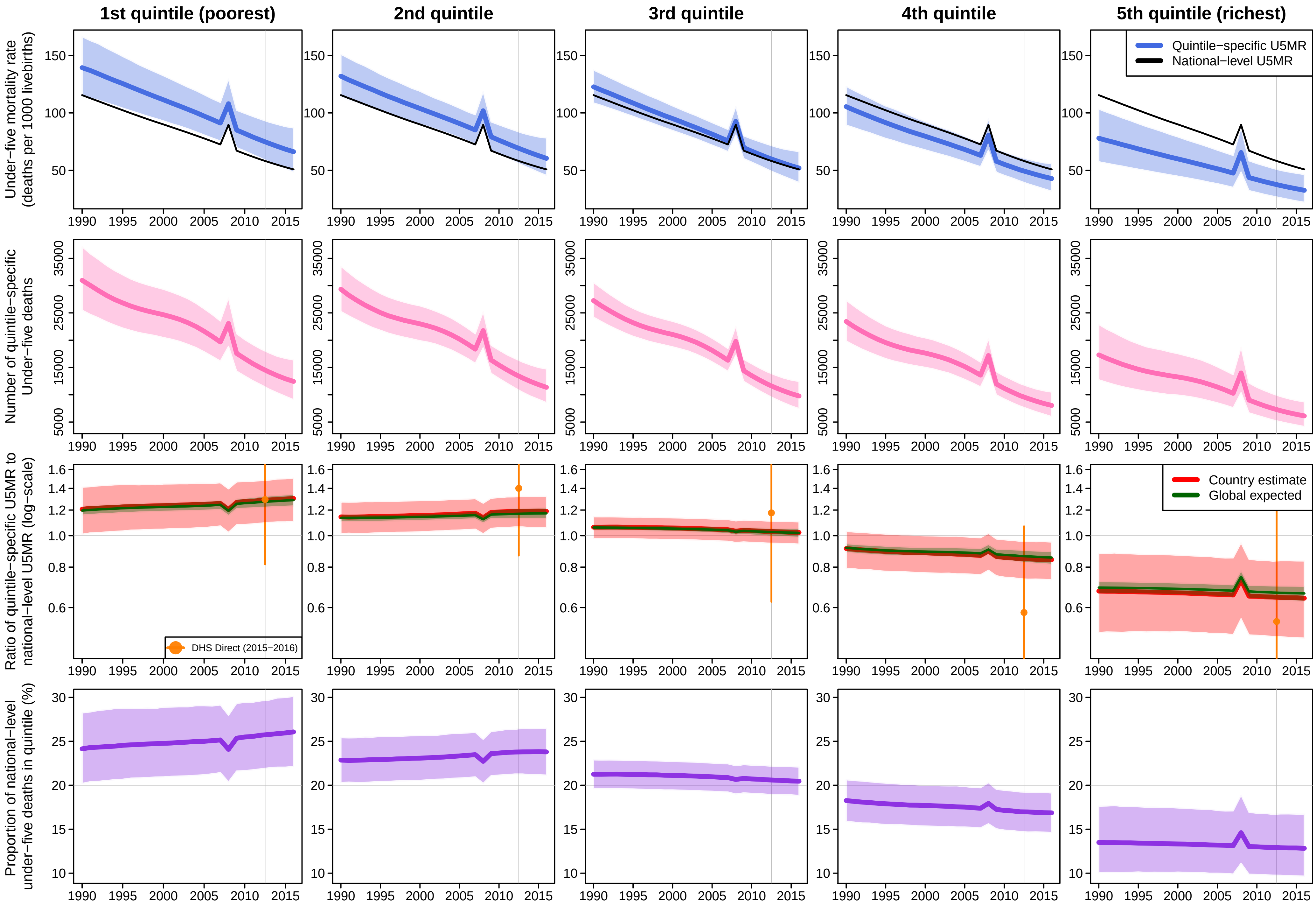

1st quintile (poorest)
2nd quintile
3rd quintile
4th quintile
5th quintile (richest)
Under−five mortality rate (deaths per 1000 livebirths)
Quintile−specific U5MR
National−level U5MR
Number of quintile−specific Under−five deaths
Ratio of quintile−specific U5MR to national−level U5MR (log−scale)
Country estimate
Global expected
DHS Direct (2015−2016)
Proportion of national−level under−five deaths in quintile (%)

Namibia

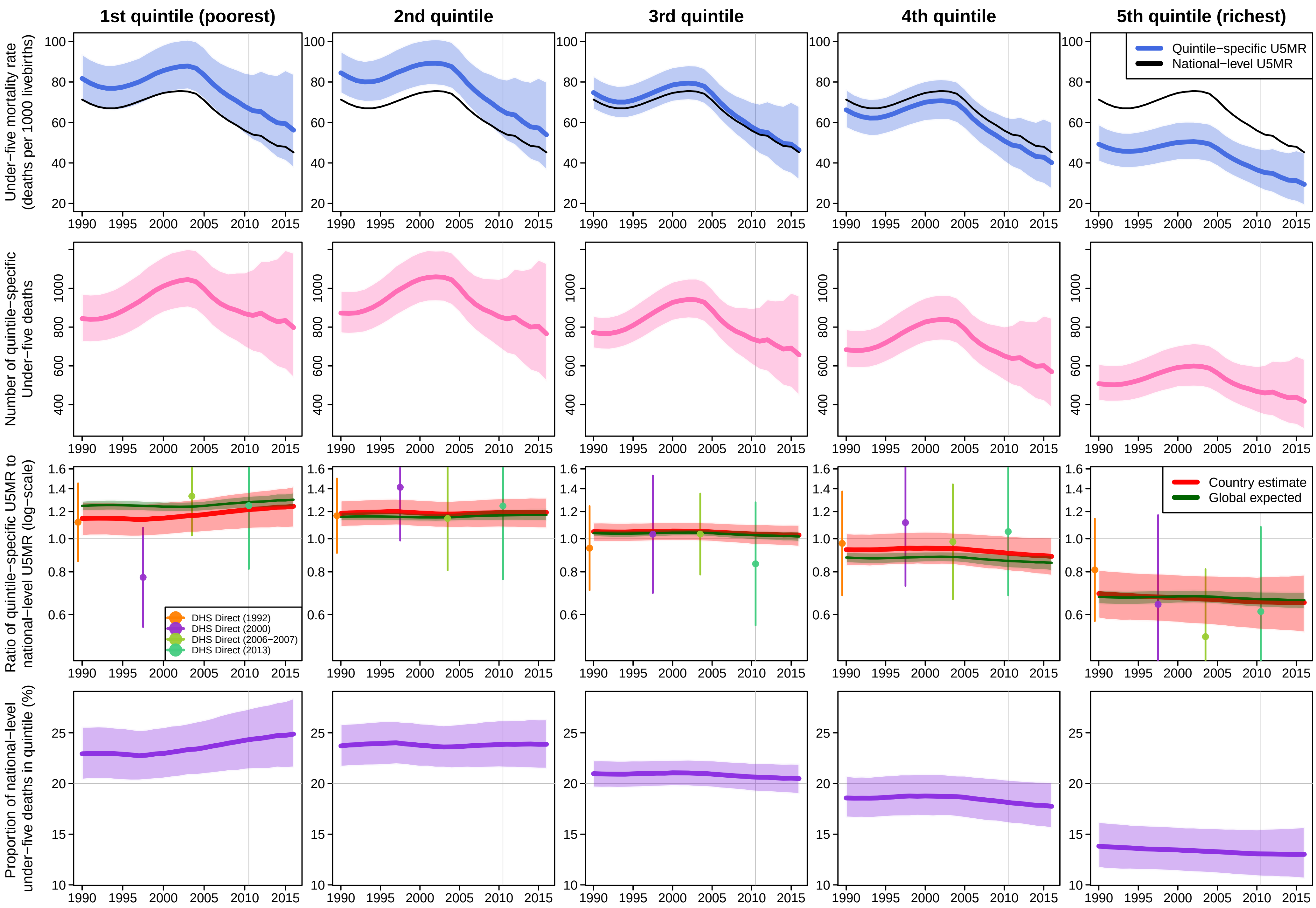

1st quintile (poorest)
2nd quintile
3rd quintile
4th quintile
5th quintile (richest)
Under−five mortality rate (deaths per 1000 livebirths)
Quintile−specific U5MR
National−level U5MR
Number of quintile−specific Under−five deaths
Ratio of quintile−specific U5MR to national−level U5MR (log−scale)
Country estimate
Global expected
DHS Direct (1992)
DHS Direct (2000)
DHS Direct (2006−2007)
DHS Direct (2013)
Proportion of national−level under−five deaths in quintile (%)

Nepal

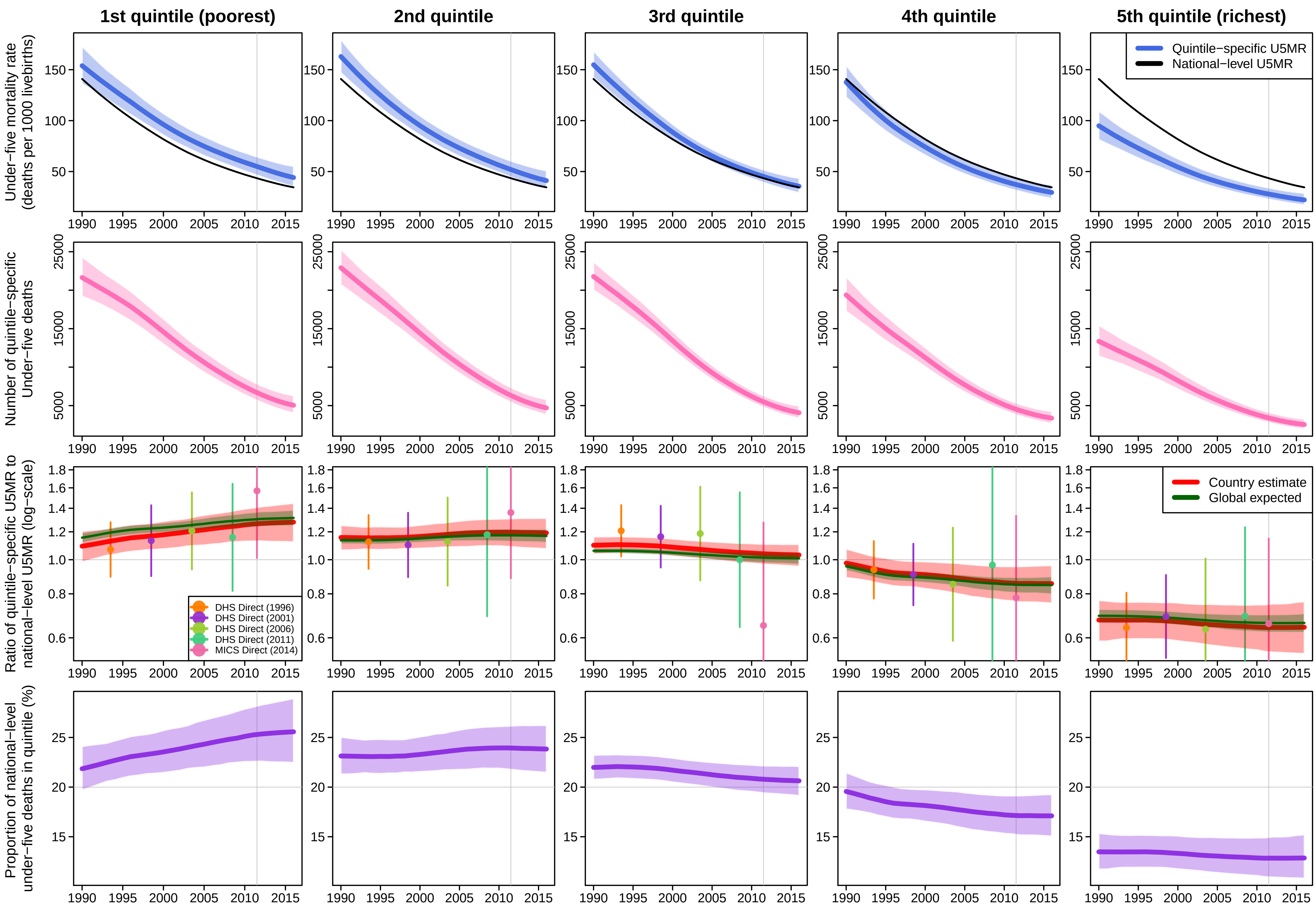

1st quintile (poorest)
2nd quintile
3rd quintile
4th quintile
5th quintile (richest)
Under−five mortality rate (deaths per 1000 livebirths)
Quintile−specific U5MR
National−level U5MR
Number of quintile−specific Under−five deaths
Ratio of quintile−specific U5MR to national−level U5MR (log−scale)
Country estimate
Global expected
DHS Direct (1996)
DHS Direct (2001)
DHS Direct (2006)
DHS Direct (2011)
MICS Direct (2014)
Proportion of national−level under−five deaths in quintile (%)

Nicaragua

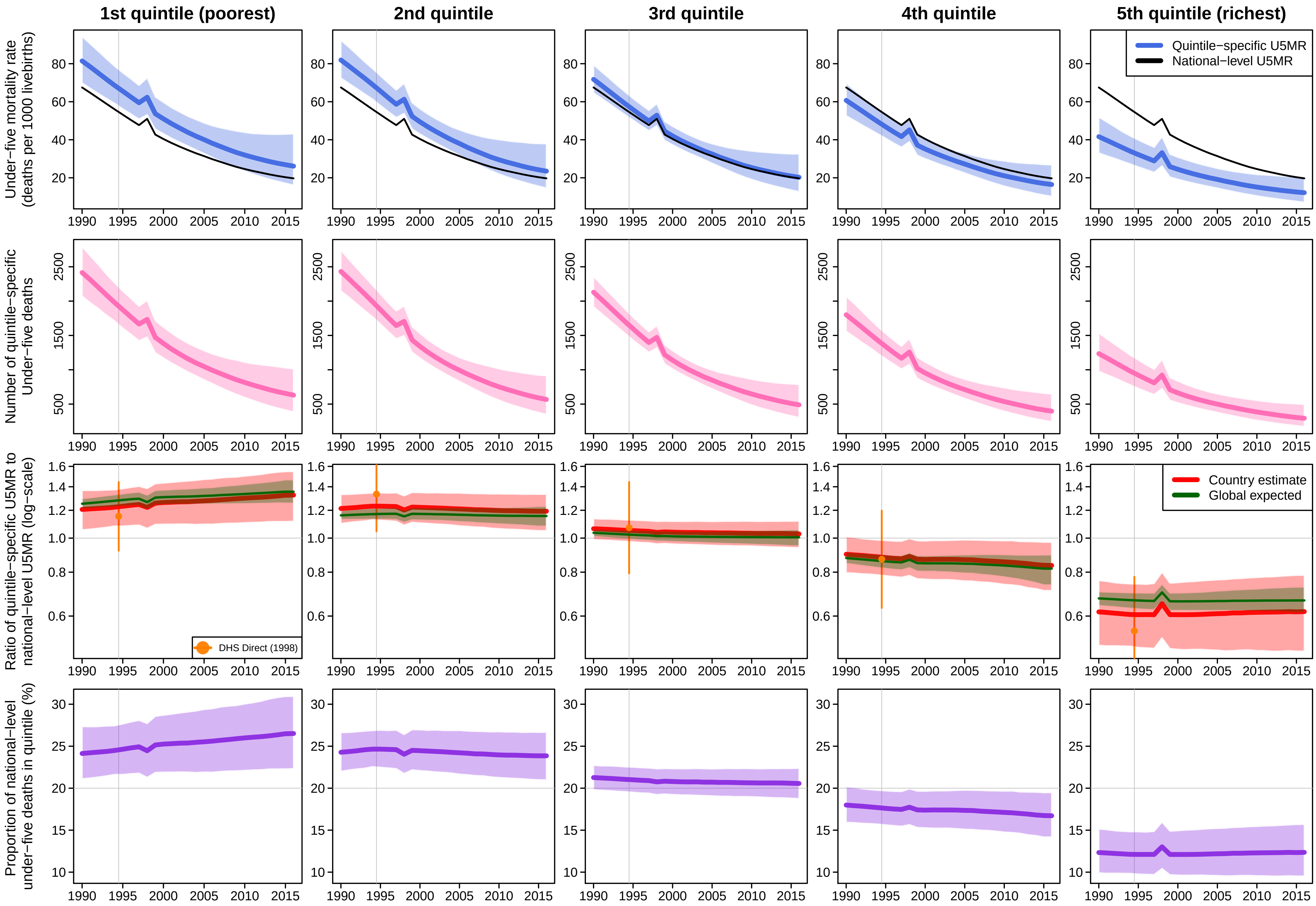

1st quintile (poorest)
2nd quintile
3rd quintile
4th quintile
5th quintile (richest)
Under−five mortality rate (deaths per 1000 livebirths)
Quintile−specific U5MR
National−level U5MR
Number of quintile−specific Under−five deaths
Ratio of quintile−specific U5MR to national−level U5MR (log−scale)
Country estimate
Global expected
DHS Direct (1998)
Proportion of national−level under−five deaths in quintile (%)

Niger

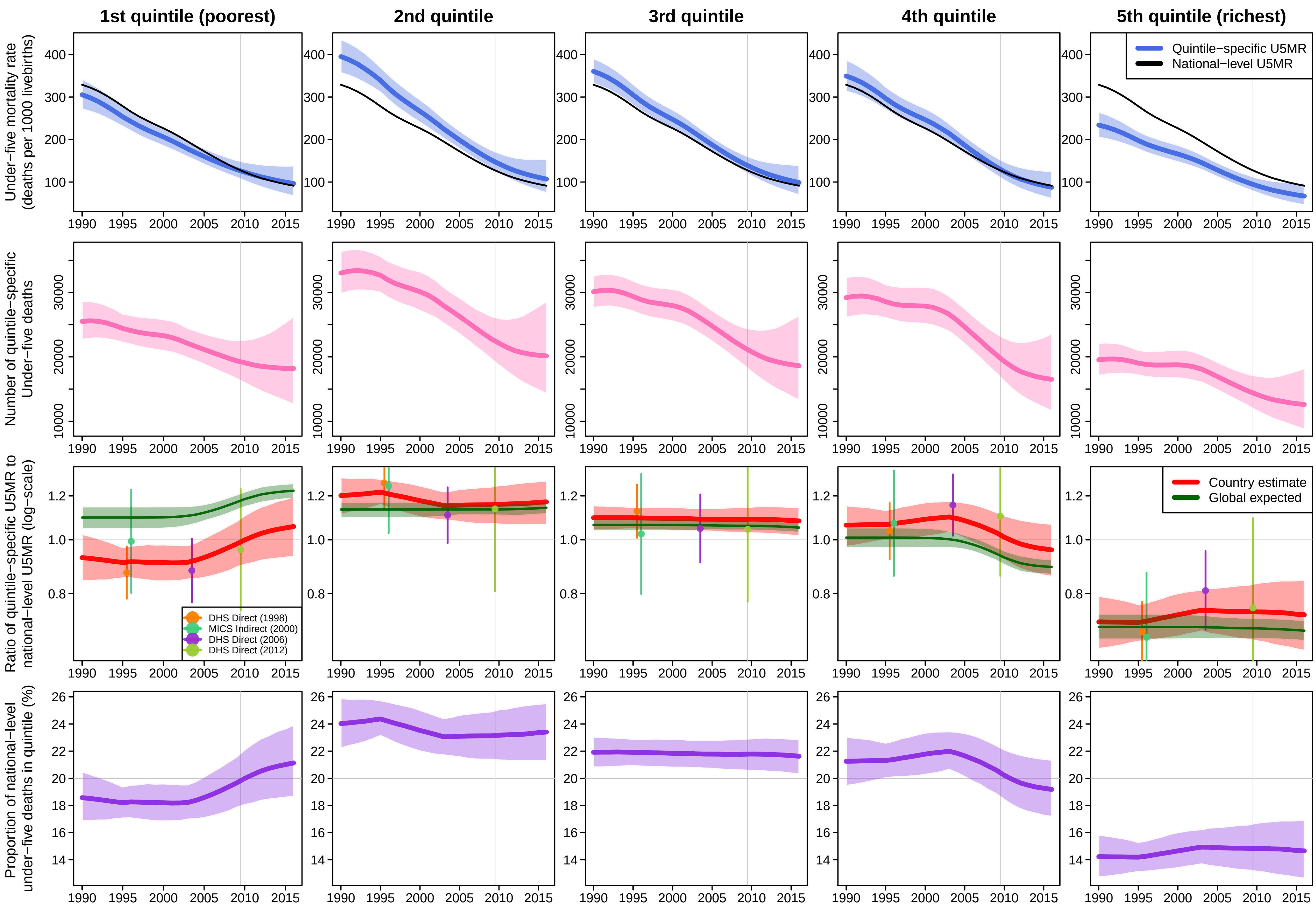

1st quintile (poorest)
2nd quintile
3rd quintile
4th quintile
5th quintile (richest)
Under−five mortality rate (deaths per 1000 livebirths)
Quintile−specific U5MR
National−level U5MR
Number of quintile−specific Under−five deaths
Ratio of quintile−specific U5MR to national−level U5MR (log−scale)
Country estimate
Global expected
DHS Direct (1998)
MICS Indirect (2000)
DHS Direct (2006)
DHS Direct (2012)
Proportion of national−level under−five deaths in quintile (%)

Nigeria

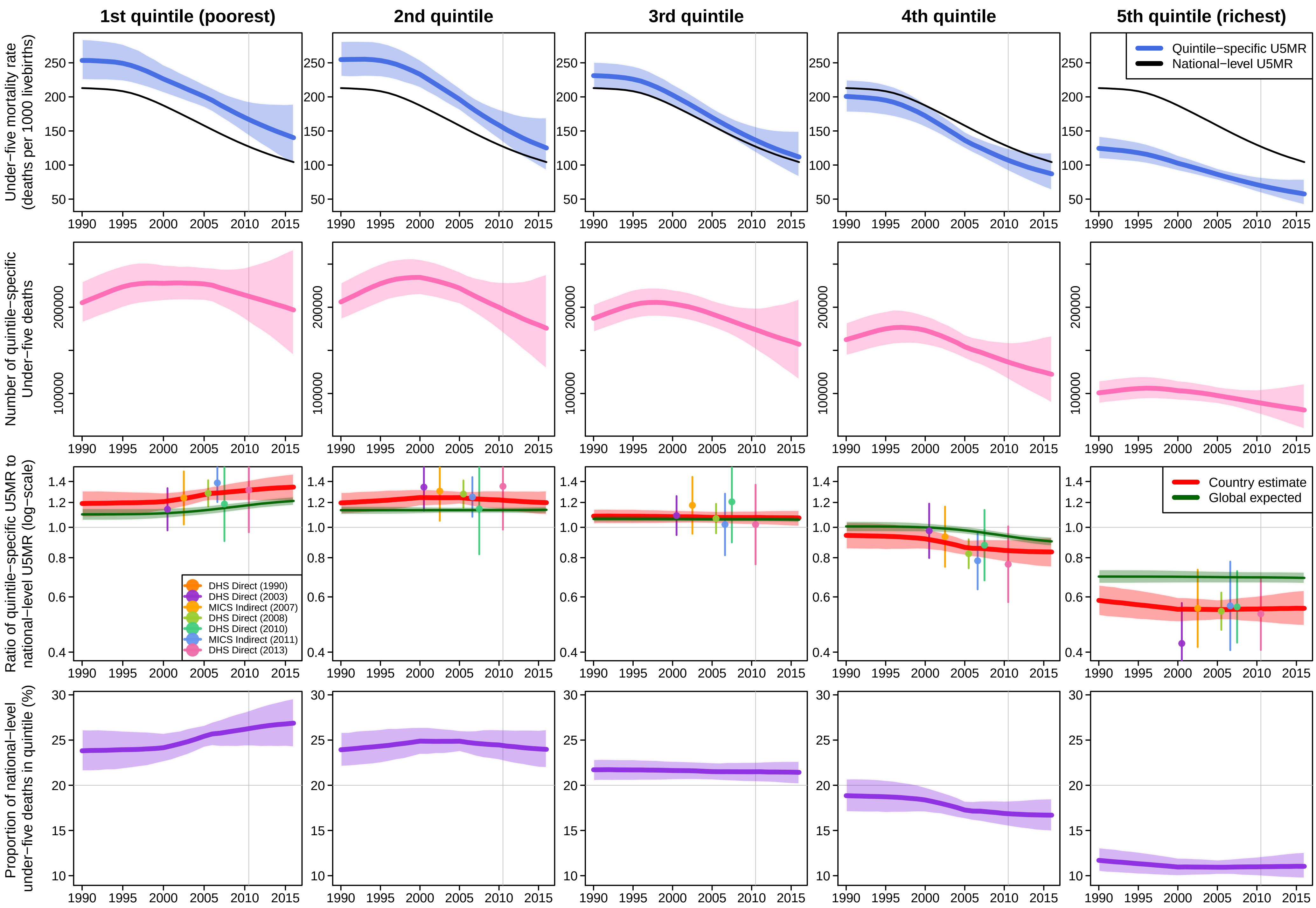
1st quintile (poorest)
2nd quintile
3rd quintile
4th quintile
5th quintile (richest)
Under−five mortality rate (deaths per 1000 livebirths)
Quintile−specific U5MR
National−level U5MR
Number of quintile−specific Under−five deaths
Ratio of quintile−specific U5MR to national−level U5MR (log−scale)
Country estimate
Global expected
DHS Direct (1990)
DHS Direct (2003)
MICS Indirect (2007)
DHS Direct (2008)
DHS Direct (2010)
MICS Indirect (2011)
DHS Direct (2013)
Proportion of national−level under−five deaths in quintile (%)
1990
1995
2000
2005
2010
2015

Pakistan

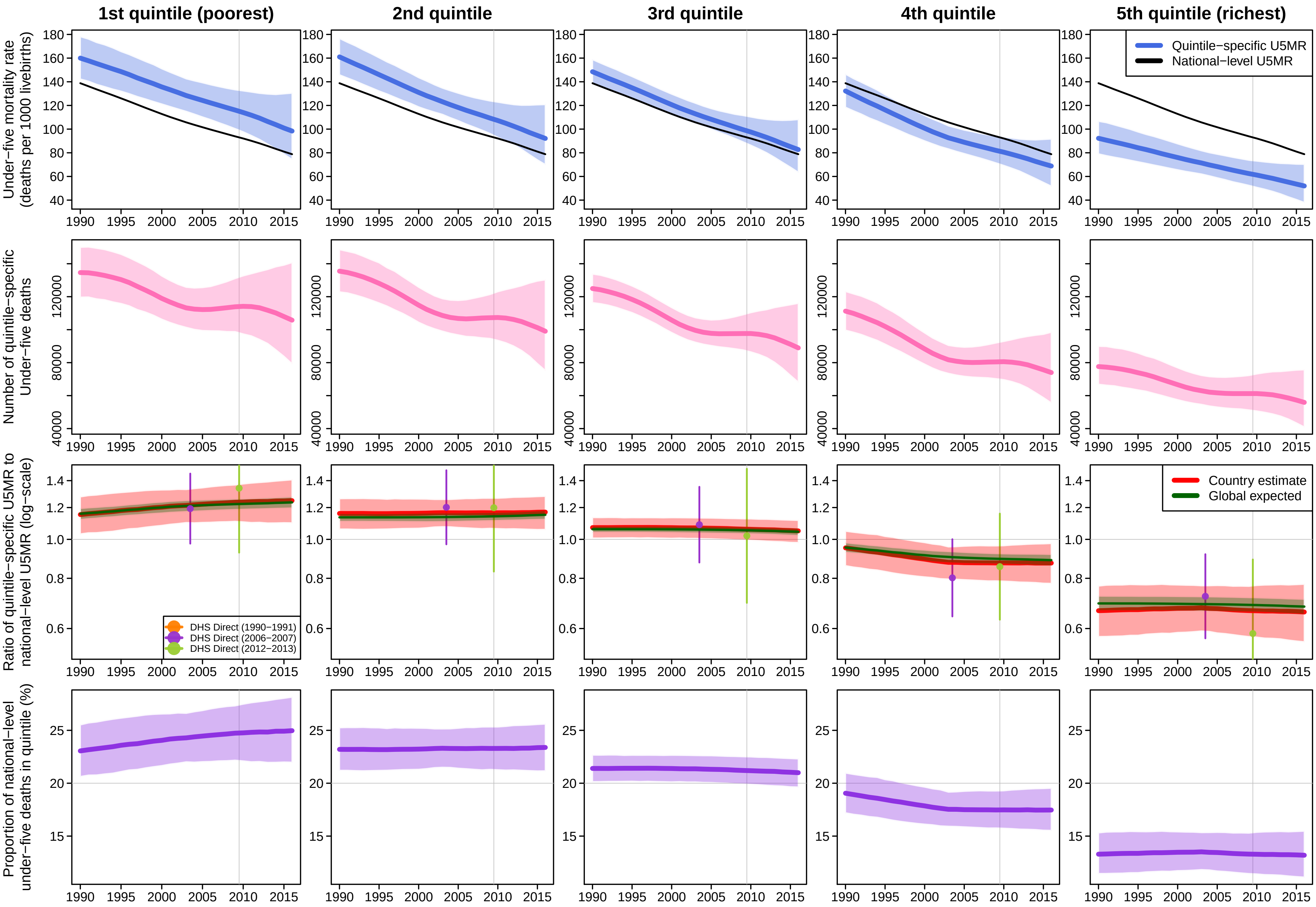
1st quintile (poorest)
2nd quintile
3rd quintile
4th quintile
5th quintile (richest)
Under−five mortality rate (deaths per 1000 livebirths)
Quintile−specific U5MR
National−level U5MR
Number of quintile−specific Under−five deaths
Ratio of quintile−specific U5MR to national−level U5MR (log−scale)
Country estimate
Global expected
DHS Direct (1990−1991)
DHS Direct (2006−2007)
DHS Direct (2012−2013)
Proportion of national−level under−five deaths in quintile (%)

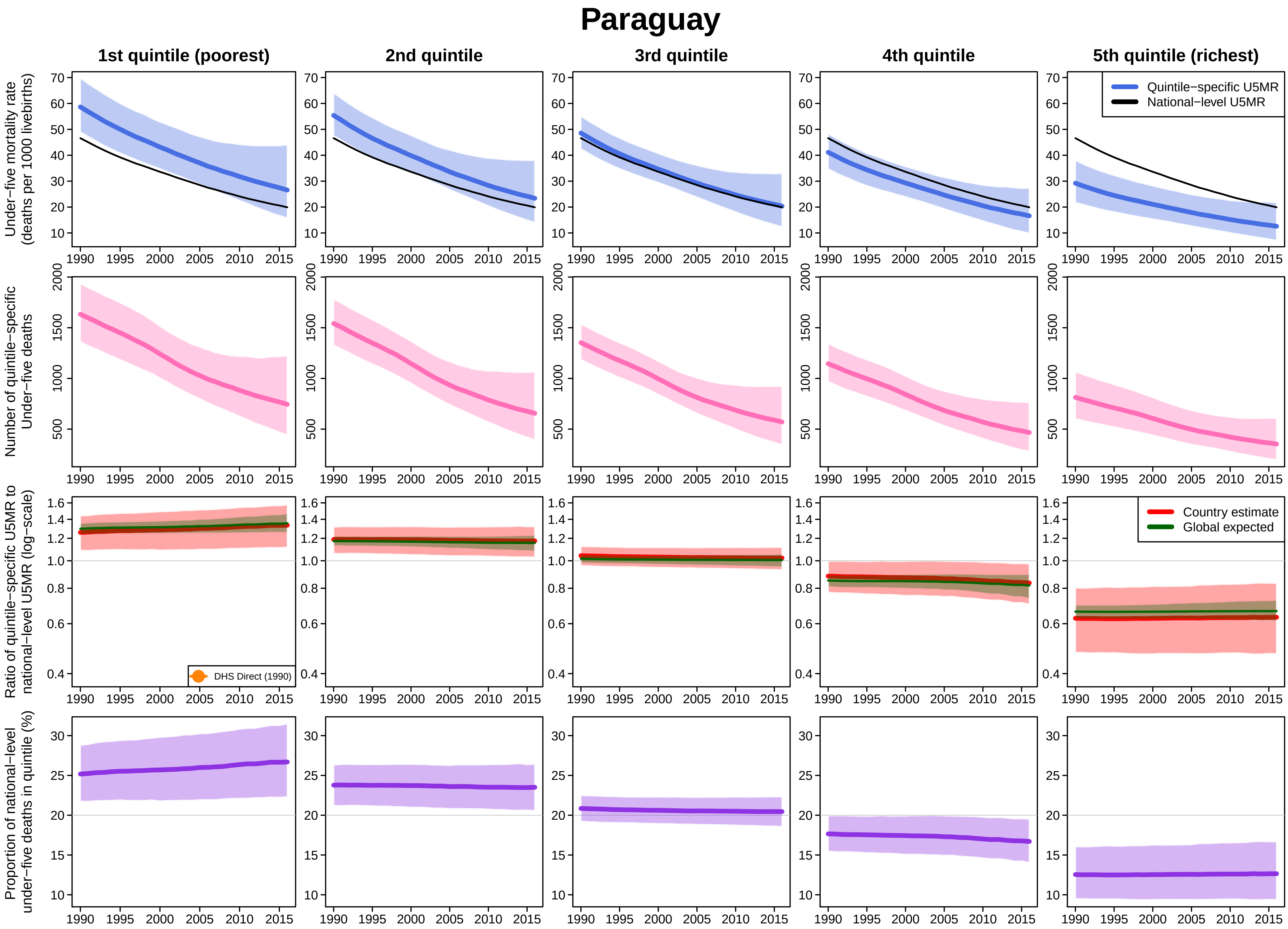

Paraguay
1st quintile (poorest)
2nd quintile
3rd quintile
4th quintile
5th quintile (richest)
Under−five mortality rate (deaths per 1000 livebirths)
Quintile−specific U5MR
National−level U5MR
Number of quintile−specific Under−five deaths
Ratio of quintile−specific U5MR to national−level U5MR (log−scale)
Country estimate
Global expected
DHS Direct (1990)
Proportion of national−level under−five deaths in quintile (%)
1990 1995 2000 2005 2010 2015

Peru

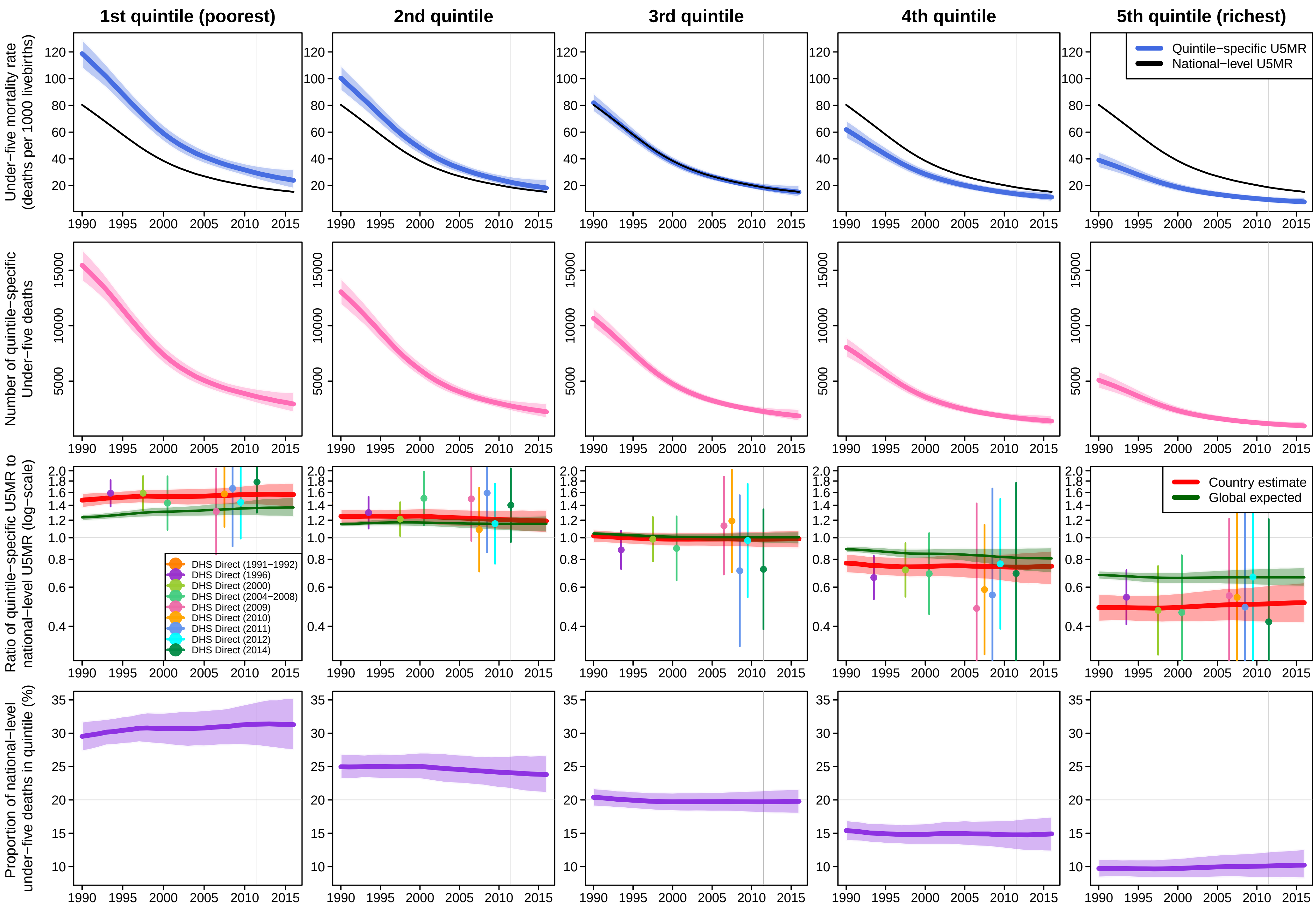

1st quintile (poorest)
2nd quintile
3rd quintile
4th quintile
5th quintile (richest)
Under−five mortality rate (deaths per 1000 livebirths)
Quintile−specific U5MR
National−level U5MR
Number of quintile−specific Under−five deaths
Ratio of quintile−specific U5MR to national−level U5MR (log−scale)
Country estimate
Global expected
DHS Direct (1991−1992)
DHS Direct (1996)
DHS Direct (2000)
DHS Direct (2004−2008)
DHS Direct (2009)
DHS Direct (2010)
DHS Direct (2011)
DHS Direct (2012)
DHS Direct (2014)
Proportion of national−level under−five deaths in quintile (%)

Philippines

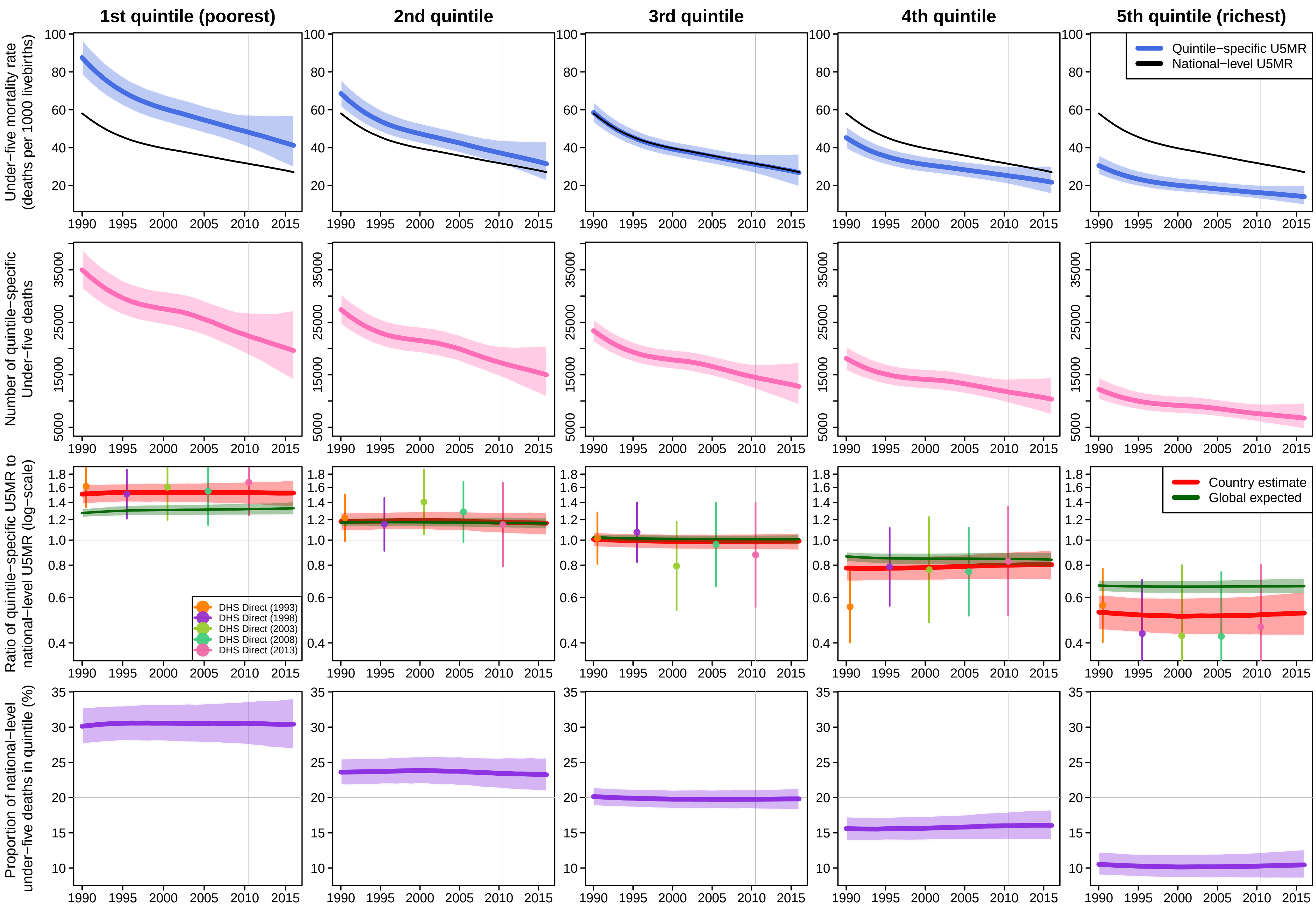

Republic of Moldova

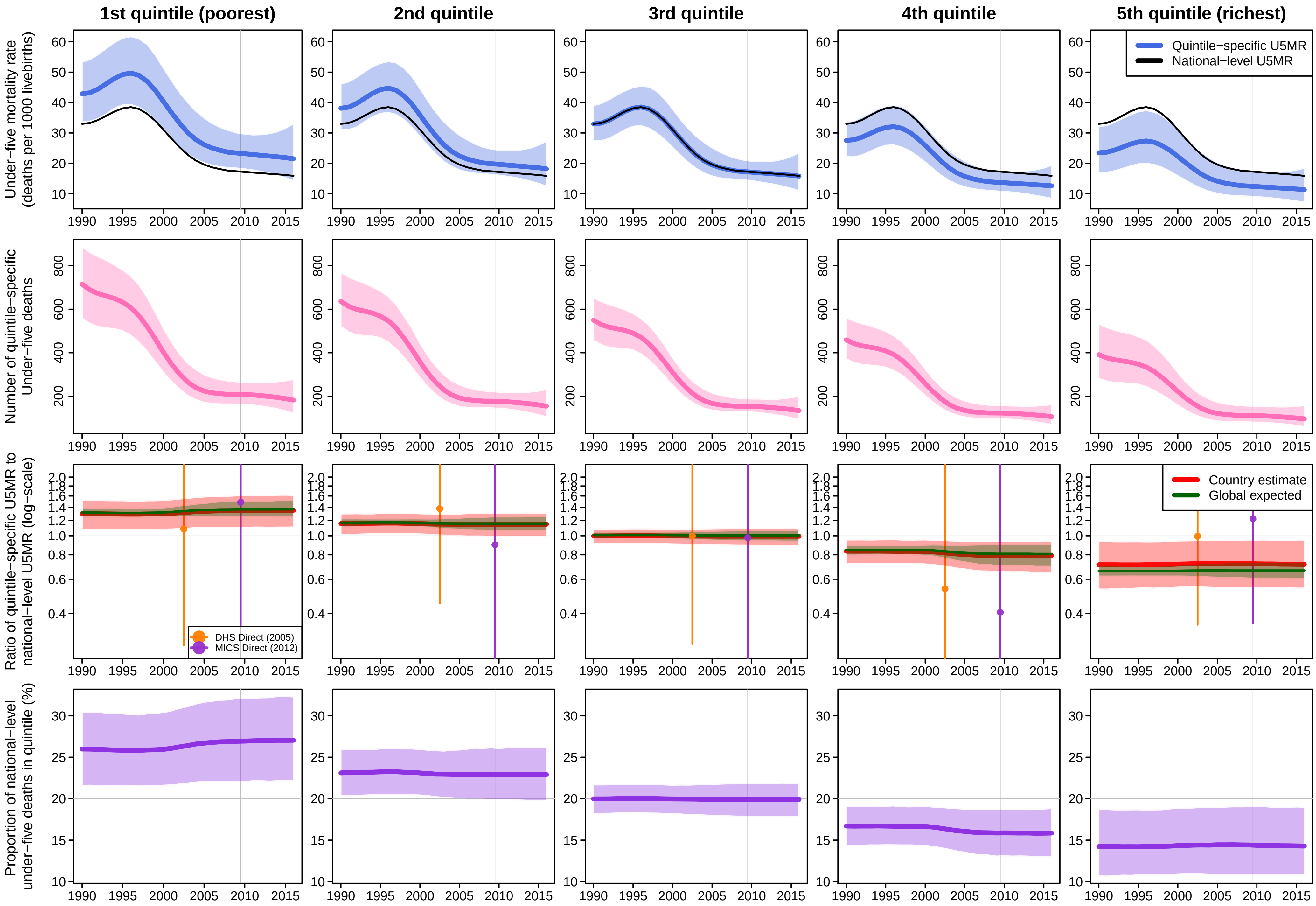

Rwanda

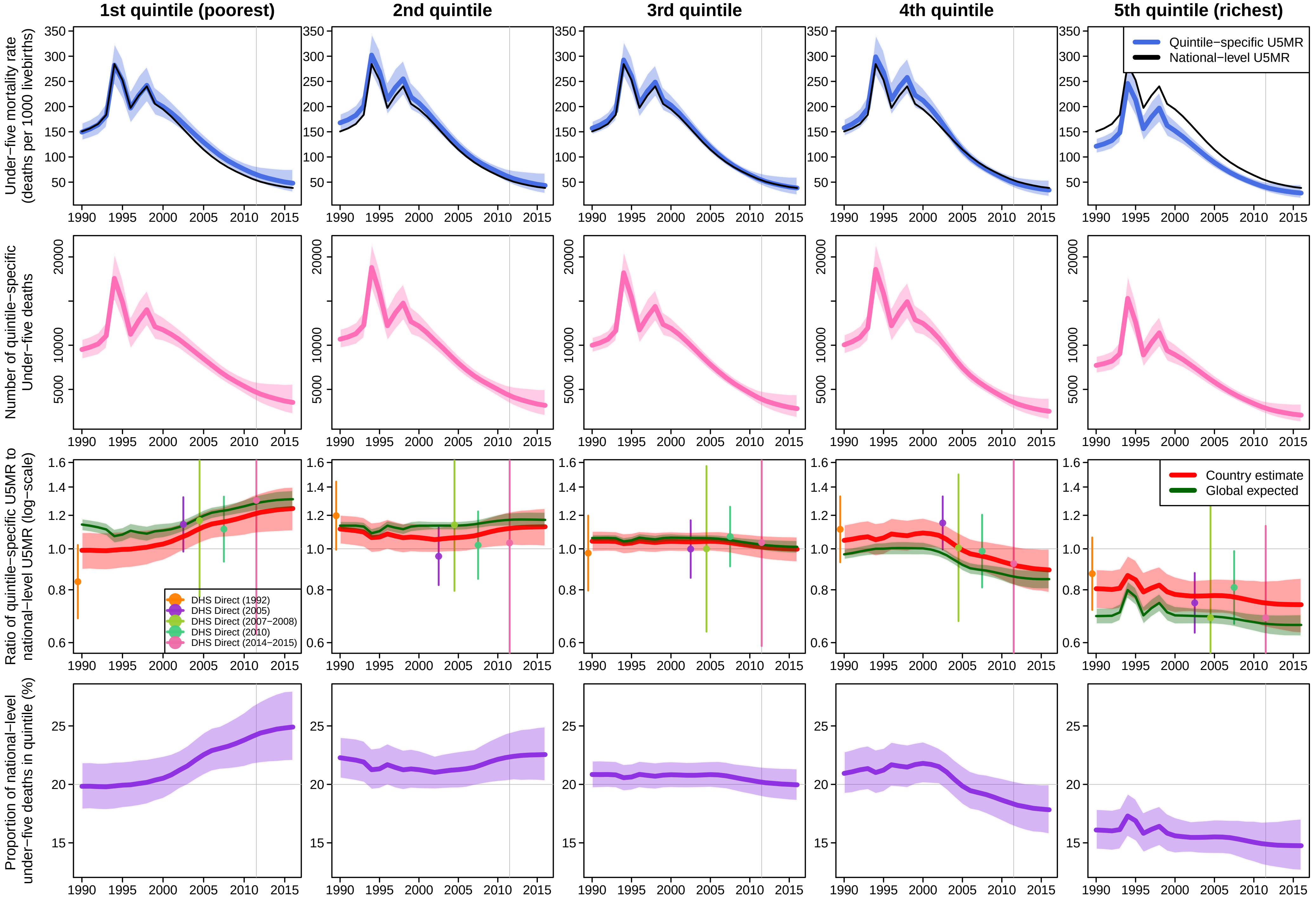

1st quintile (poorest)
2nd quintile
3rd quintile
4th quintile
5th quintile (richest)
Under−five mortality rate (deaths per 1000 livebirths)
Quintile−specific U5MR
National−level U5MR
Number of quintile−specific Under−five deaths
Ratio of quintile−specific U5MR to national−level U5MR (log−scale)
Country estimate
Global expected
DHS Direct (1992)
DHS Direct (2005)
DHS Direct (2007−2008)
DHS Direct (2010)
DHS Direct (2014−2015)
Proportion of national−level under−five deaths in quintile (%)

Sao Tome and Principe

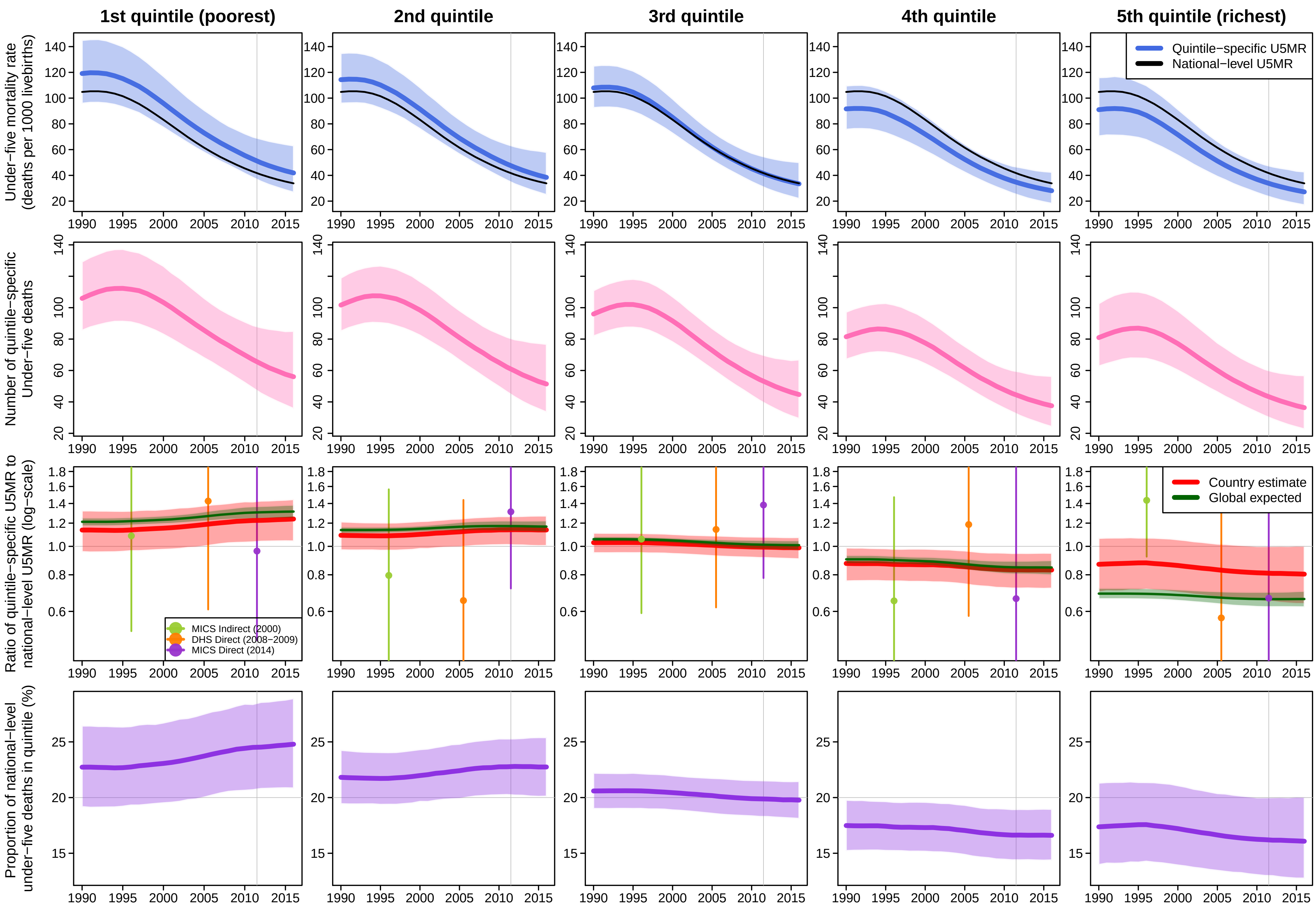

Senegal

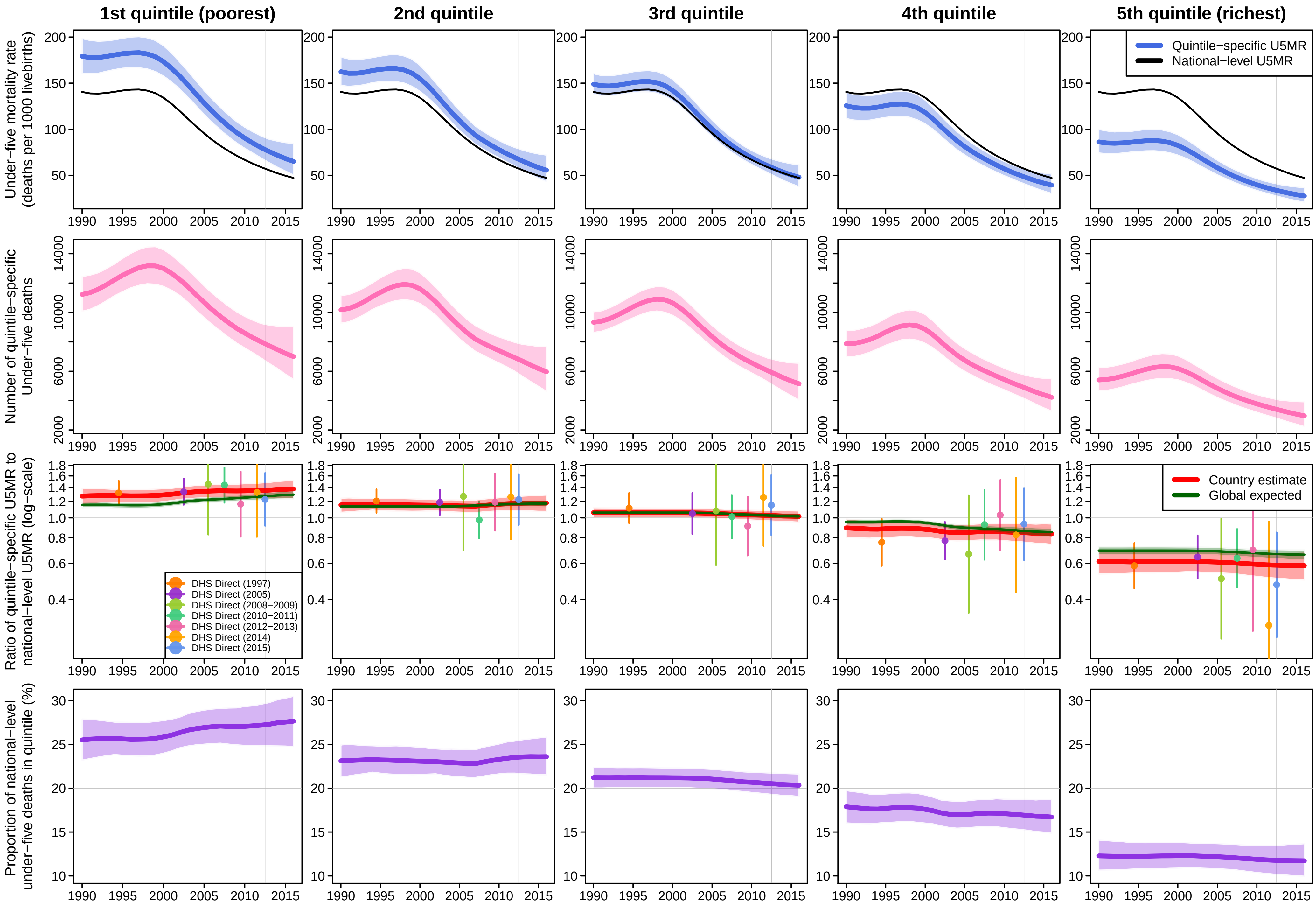

1st quintile (poorest)
2nd quintile
3rd quintile
4th quintile
5th quintile (richest)
Under−five mortality rate (deaths per 1000 livebirths)
Quintile−specific U5MR
National−level U5MR
Number of quintile−specific Under−five deaths
Ratio of quintile−specific U5MR to national−level U5MR (log−scale)
Country estimate
Global expected
DHS Direct (1997)
DHS Direct (2005)
DHS Direct (2008−2009)
DHS Direct (2010−2011)
DHS Direct (2012−2013)
DHS Direct (2014)
DHS Direct (2015)
Proportion of national−level under−five deaths in quintile (%)

Serbia

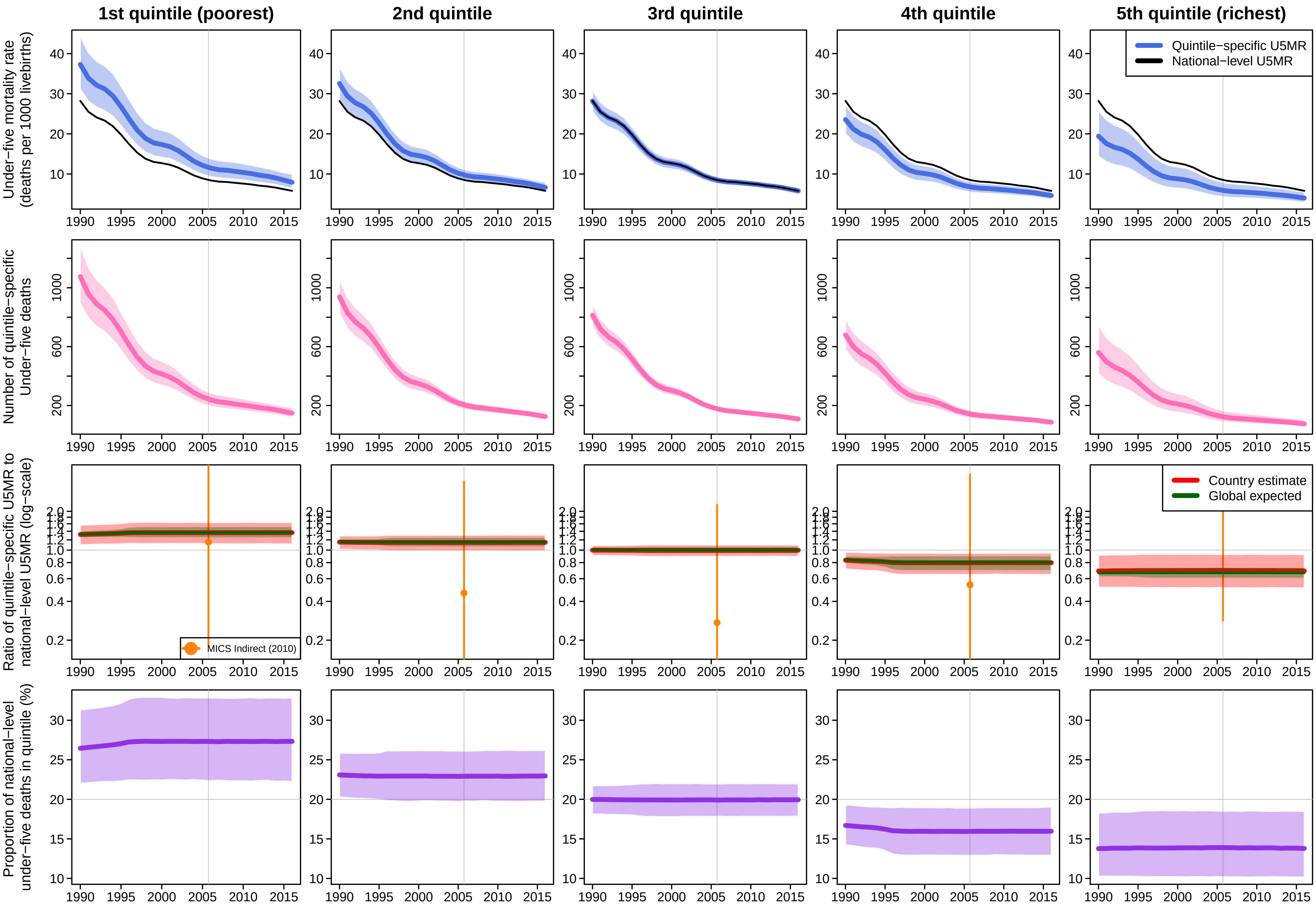
1st quintile (poorest)
2nd quintile
3rd quintile
4th quintile
5th quintile (richest)
Under−five mortality rate (deaths per 1000 livebirths)
Quintile−specific U5MR
National−level U5MR
Number of quintile−specific Under−five deaths
Ratio of quintile−specific U5MR to national−level U5MR (log−scale)
MICS Indirect (2010)
Country estimate
Global expected
Proportion of national−level under−five deaths in quintile (%)
1990
1995
2000
2005
2010
2015

Sierra Leone

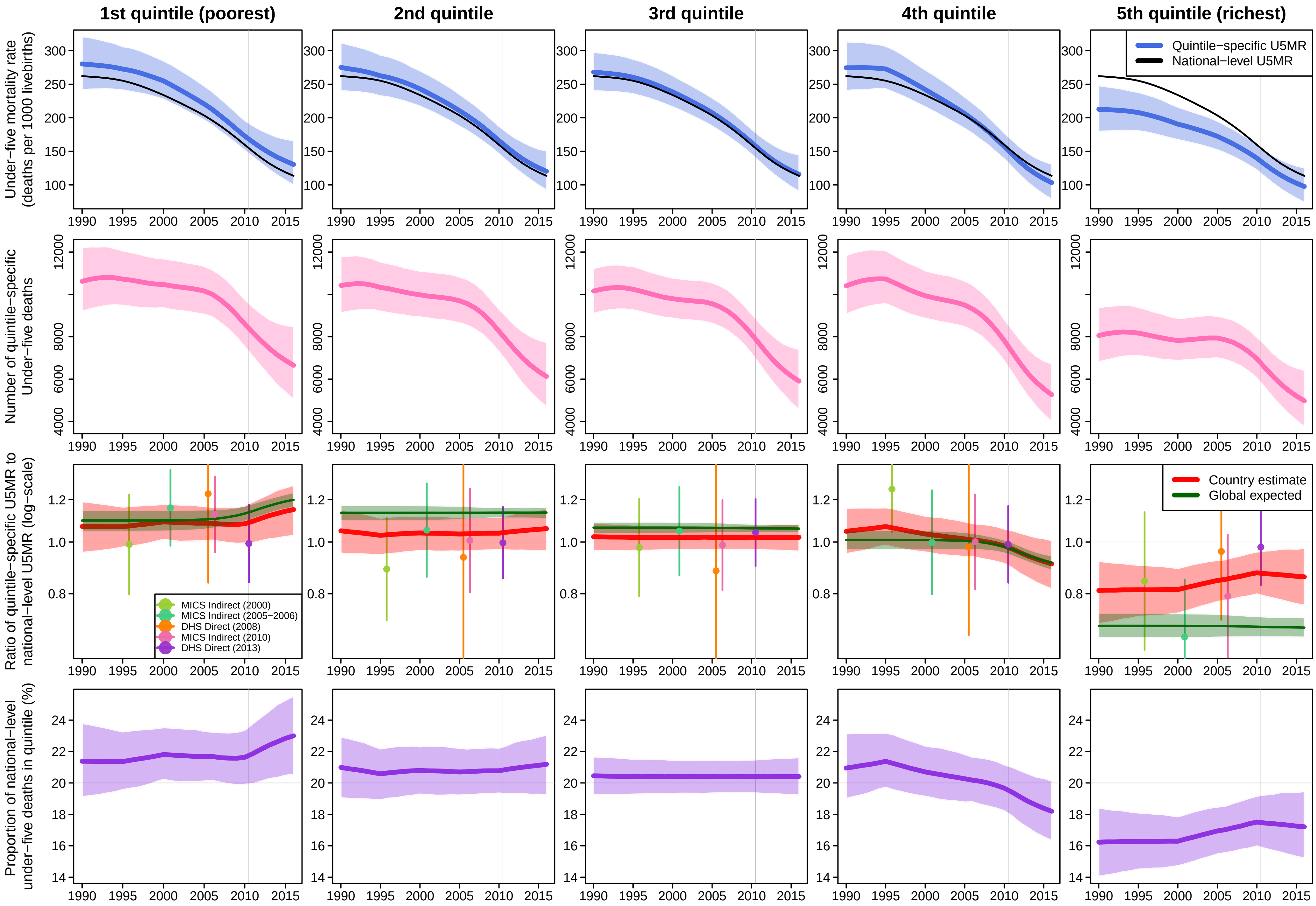

Somalia

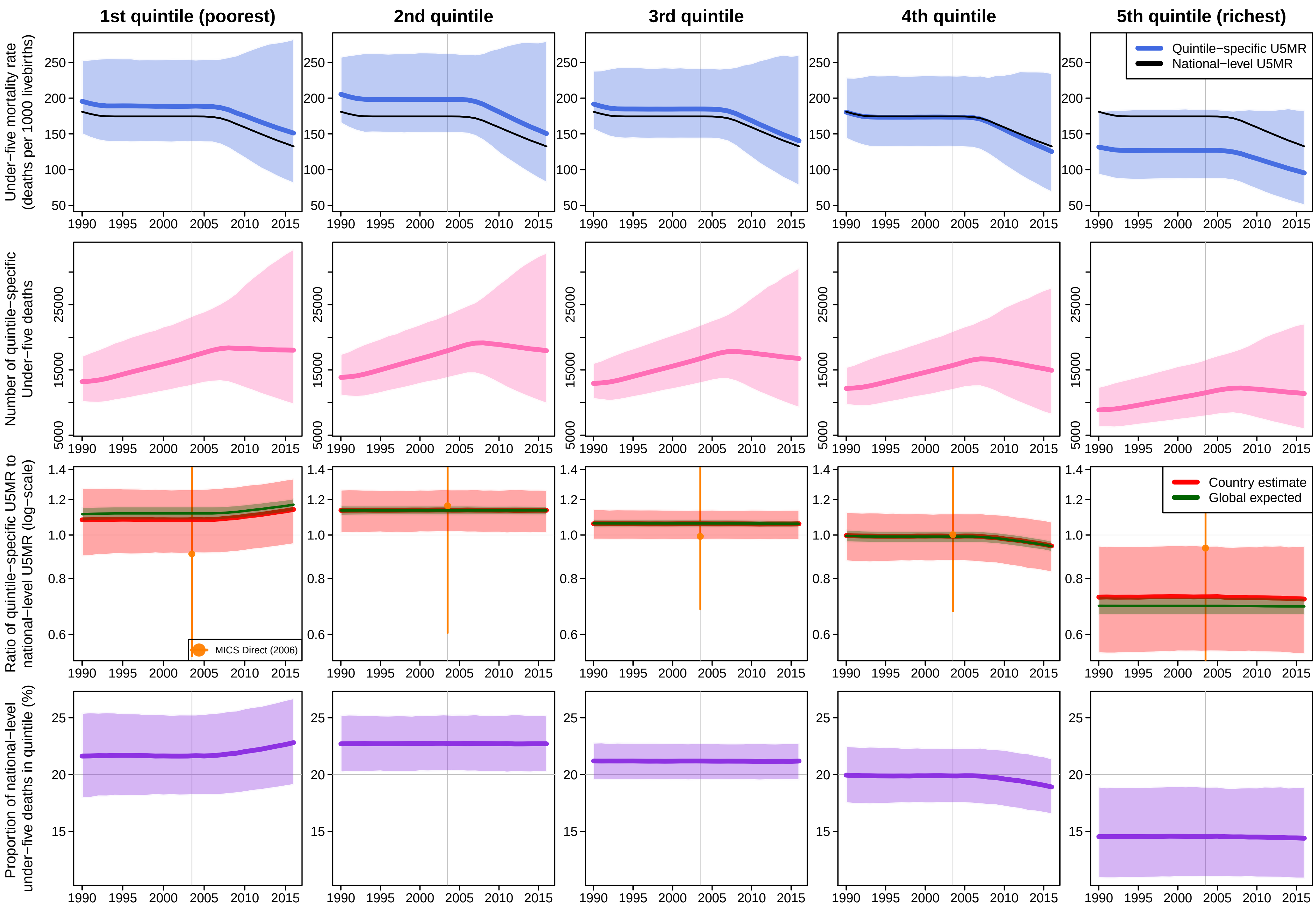

1st quintile (poorest)
2nd quintile
3rd quintile
4th quintile
5th quintile (richest)
Under−five mortality rate (deaths per 1000 livebirths)
Quintile−specific U5MR
National−level U5MR
Number of quintile−specific Under−five deaths
Ratio of quintile−specific U5MR to national−level U5MR (log−scale)
Country estimate
Global expected
MICS Direct (2006)
Proportion of national−level under−five deaths in quintile (%)

South Africa

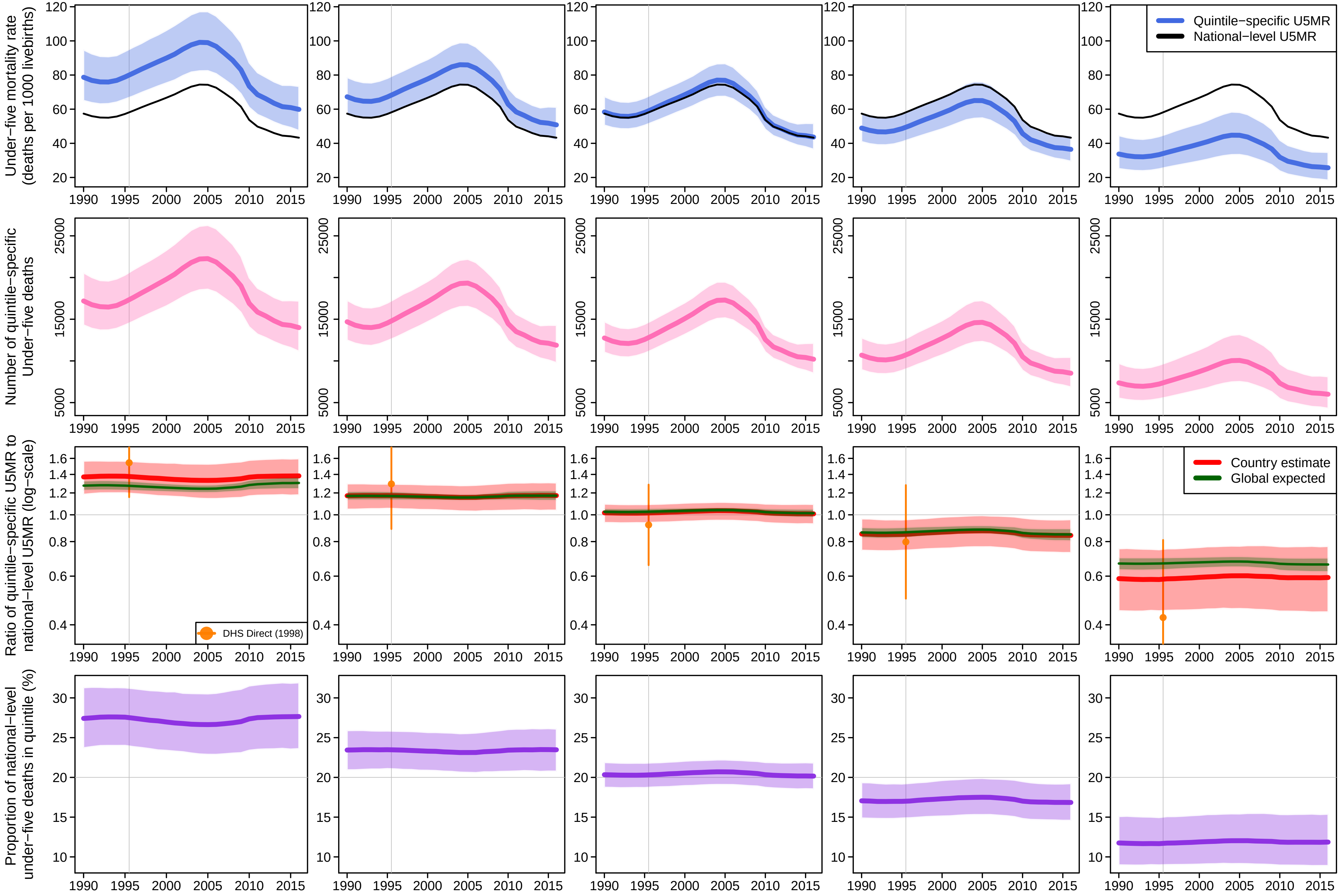

South Sudan

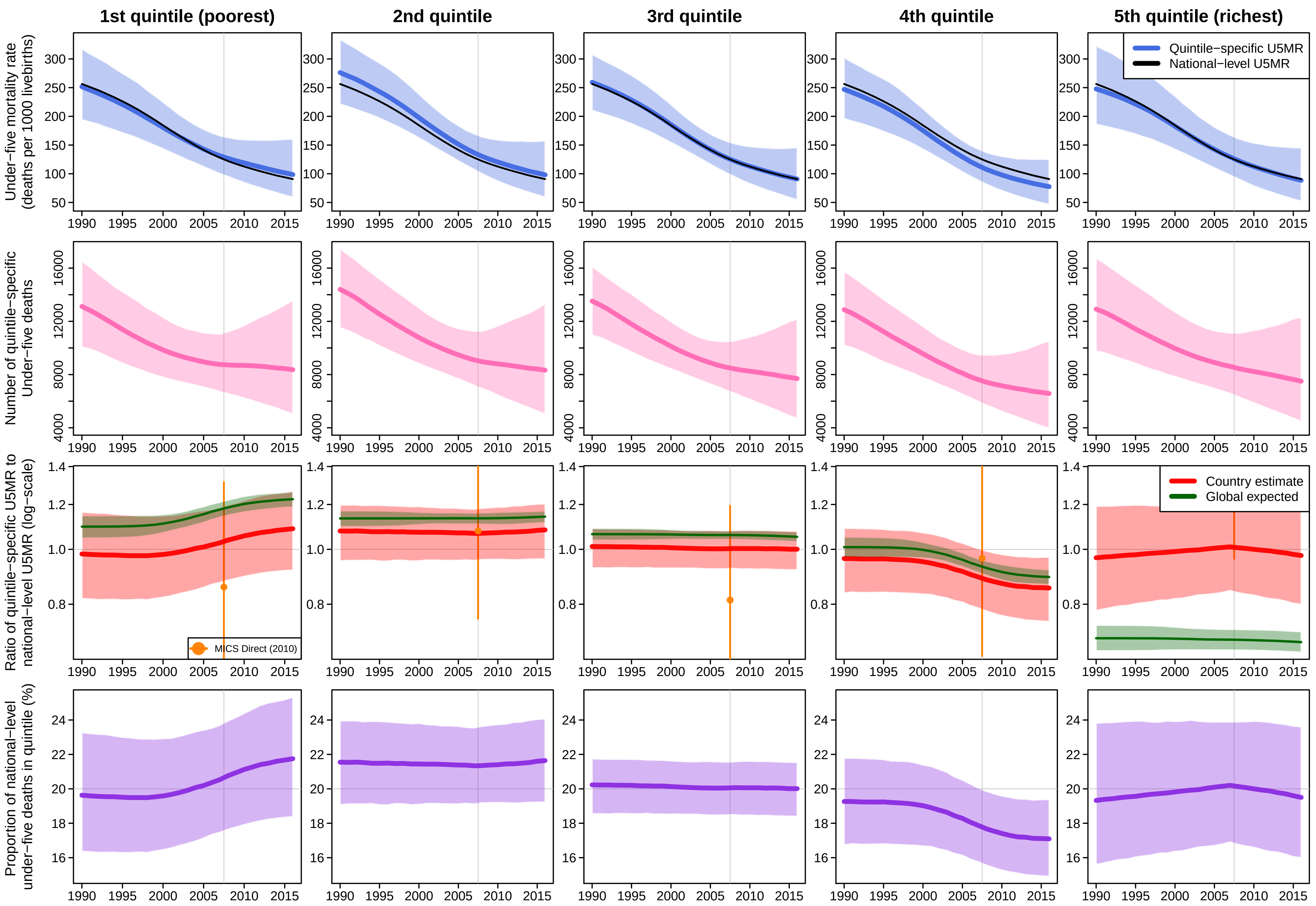
1st quintile (poorest)
2nd quintile
3rd quintile
4th quintile
5th quintile (richest)
Under−five mortality rate (deaths per 1000 livebirths)
Number of quintile−specific Under−five deaths
Ratio of quintile−specific U5MR to national−level U5MR (log−scale)
Proportion of national−level under−five deaths in quintile (%)
Quintile−specific U5MR
National−level U5MR
Country estimate
Global expected
MICS Direct (2010)

State of Palestine

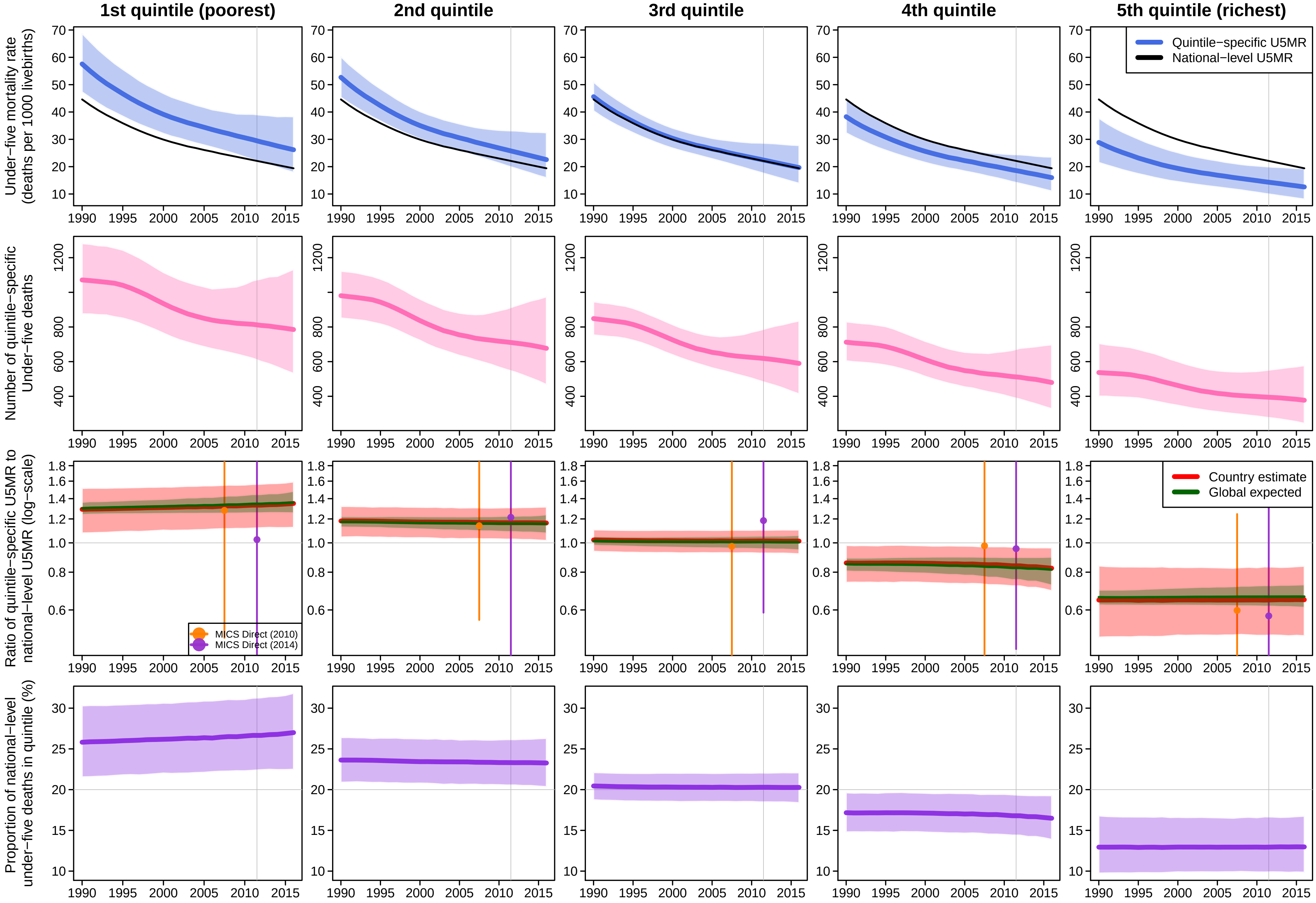

Sudan

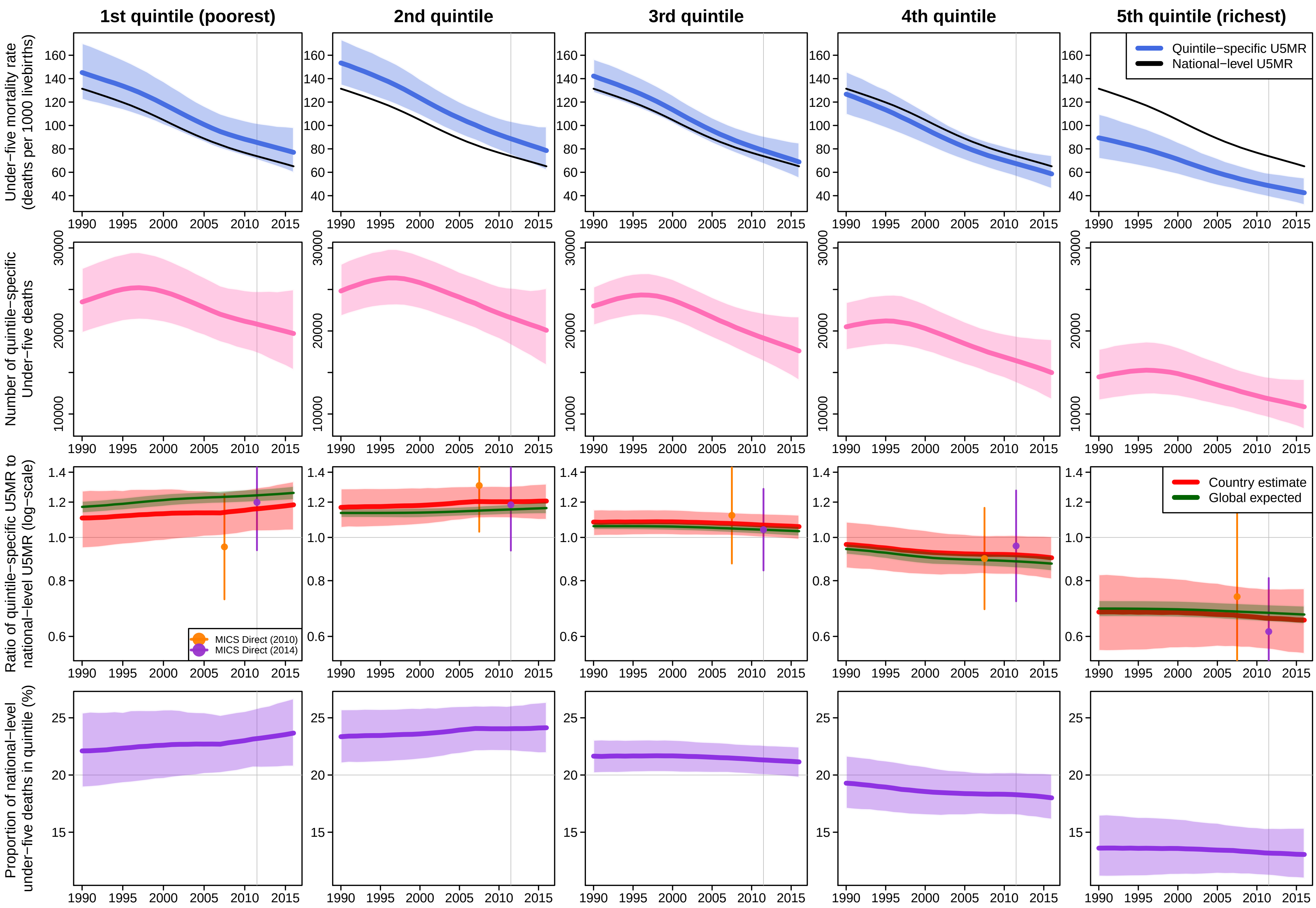
1st quintile (poorest)
2nd quintile
3rd quintile
4th quintile
5th quintile (richest)
Under−five mortality rate (deaths per 1000 livebirths)
Quintile−specific U5MR
National−level U5MR
Number of quintile−specific Under−five deaths
Ratio of quintile−specific U5MR to national−level U5MR (log−scale)
Country estimate
Global expected
MICS Direct (2010)
MICS Direct (2014)
Proportion of national−level under−five deaths in quintile (%)

Suriname

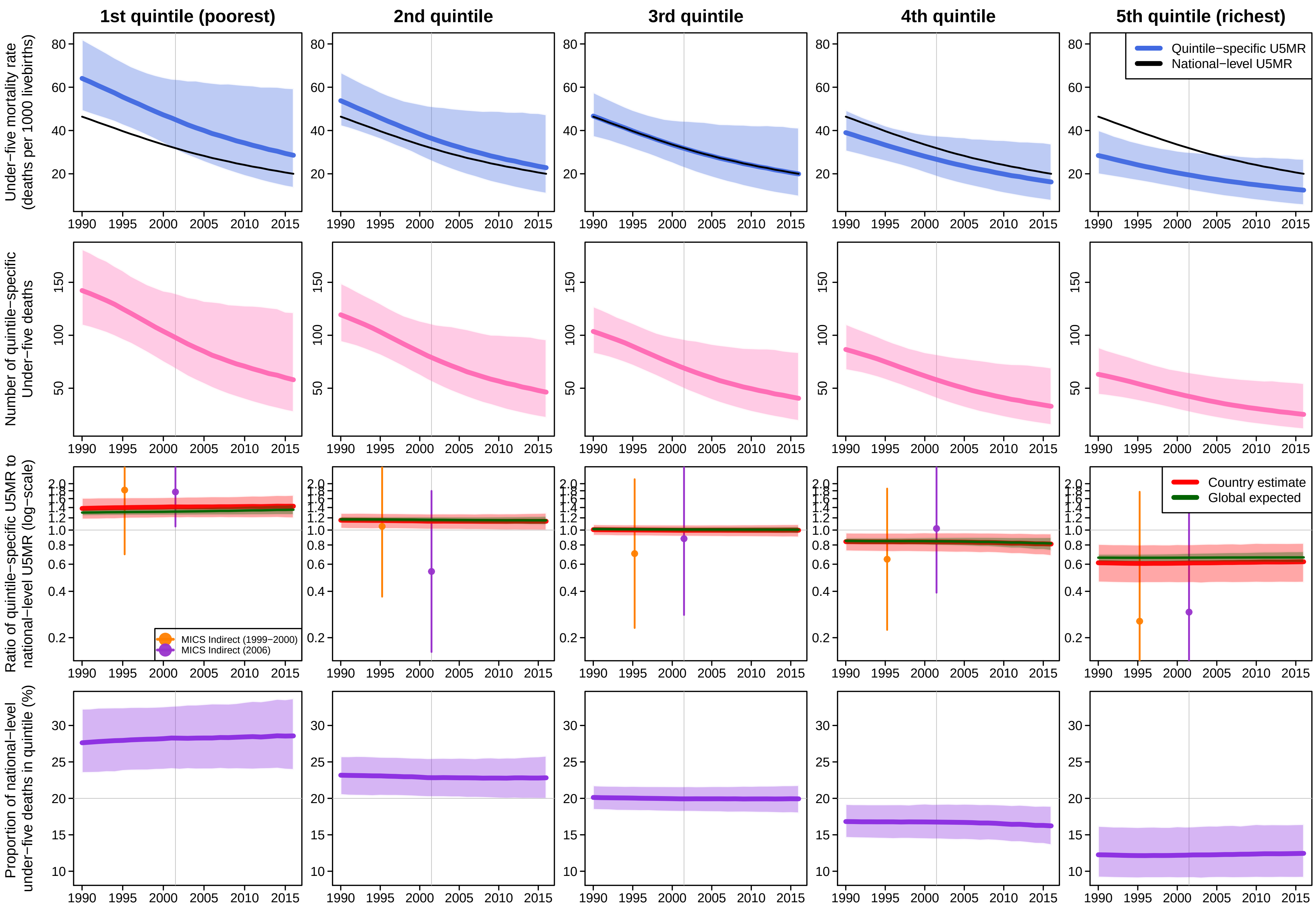

1st quintile (poorest)
2nd quintile
3rd quintile
4th quintile
5th quintile (richest)
Under−five mortality rate (deaths per 1000 livebirths)
Number of quintile−specific Under−five deaths
Ratio of quintile−specific U5MR to national−level U5MR (log−scale)
Proportion of national−level under−five deaths in quintile (%)
Quintile−specific U5MR
National−level U5MR
Country estimate
Global expected
MICS Indirect (1999−2000)
MICS Indirect (2006)

Swaziland

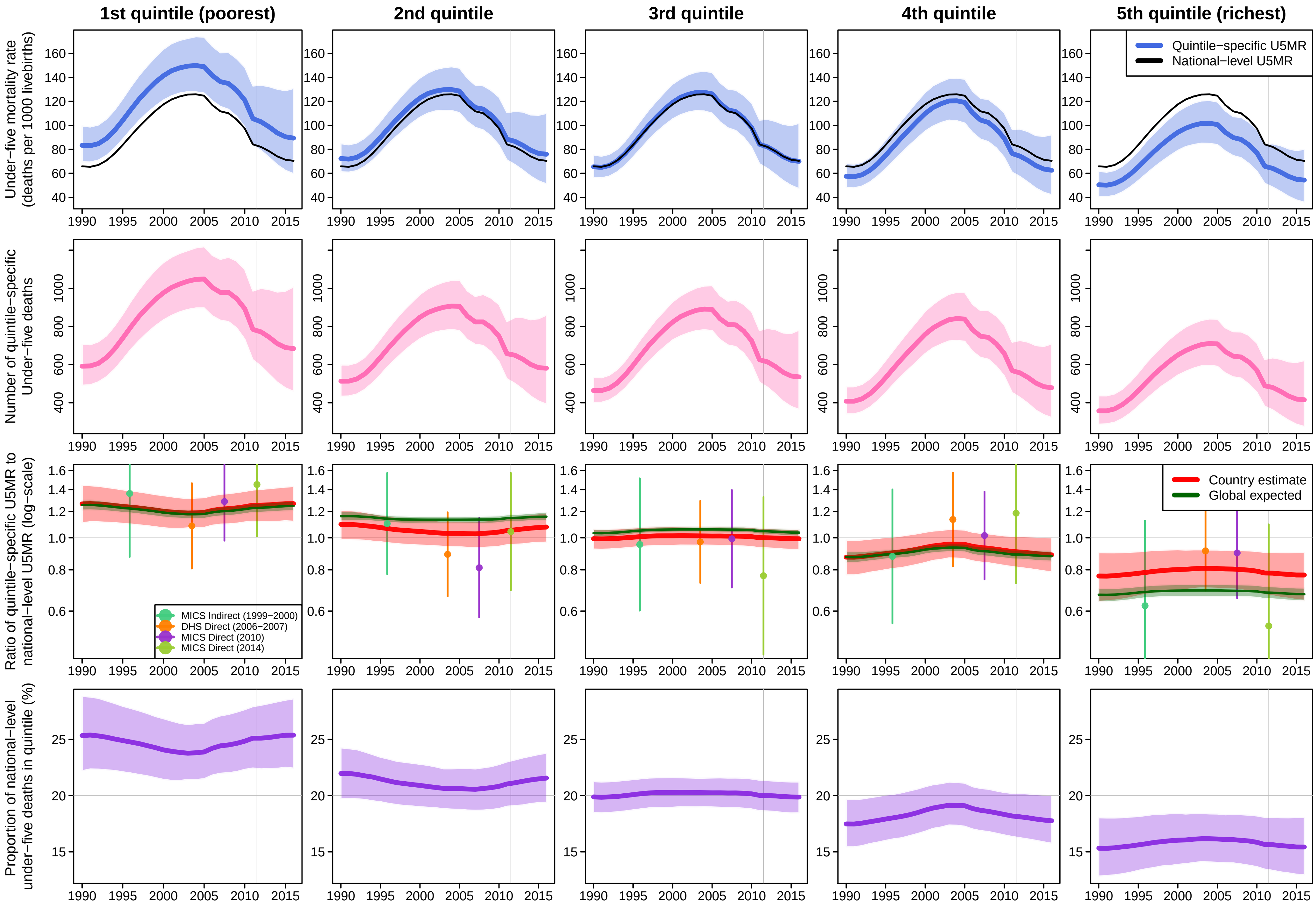

1st quintile (poorest)
2nd quintile
3rd quintile
4th quintile
5th quintile (richest)
Under−five mortality rate (deaths per 1000 livebirths)
Number of quintile−specific Under−five deaths
Ratio of quintile−specific U5MR to national−level U5MR (log−scale)
Proportion of national−level under−five deaths in quintile (%)
Quintile−specific U5MR
National−level U5MR
Country estimate
Global expected
MICS Indirect (1999−2000)
DHS Direct (2006−2007)
MICS Direct (2010)
MICS Direct (2014)

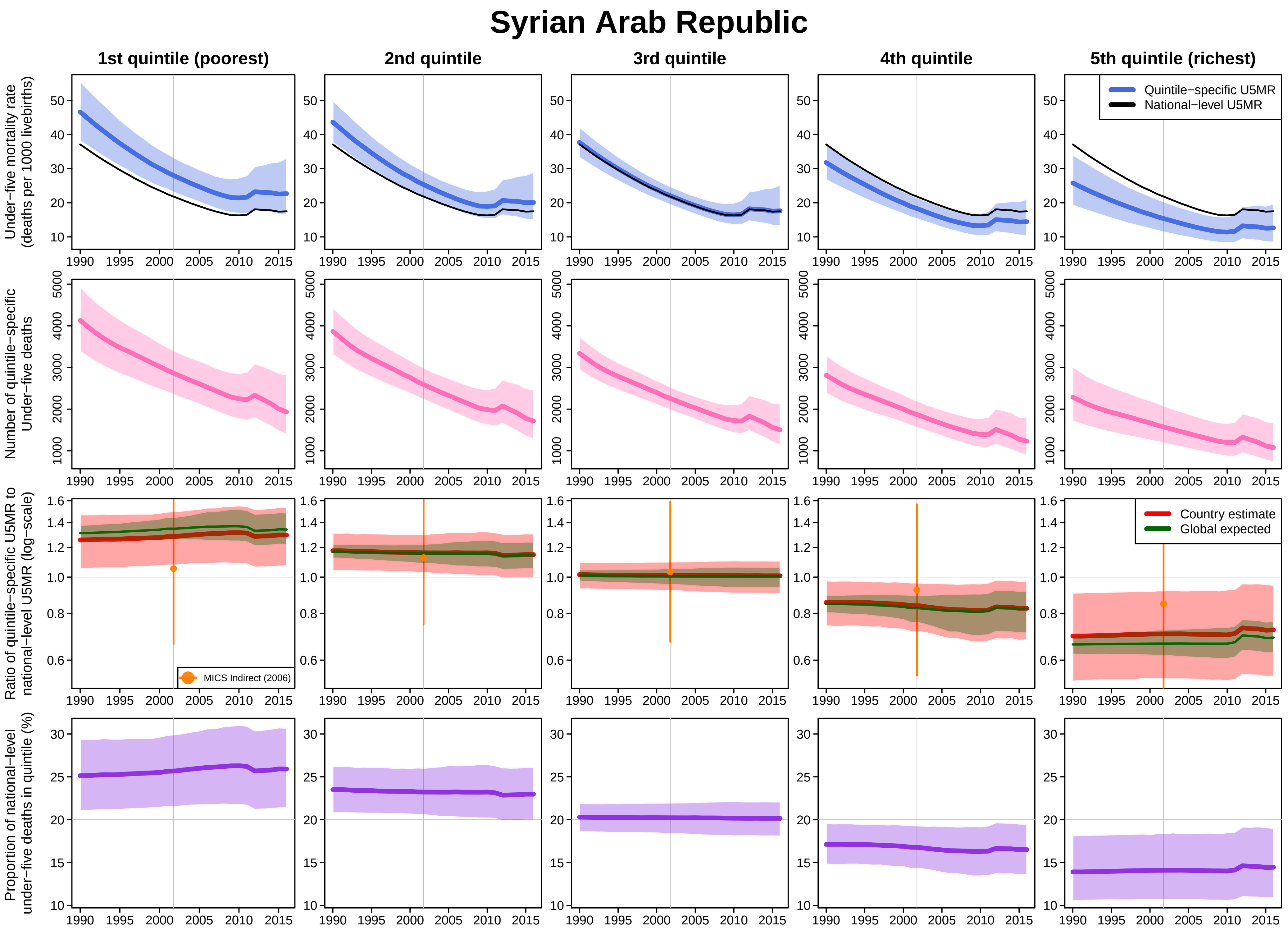

Syrian Arab Republic
1st quintile (poorest)
2nd quintile
3rd quintile
4th quintile
5th quintile (richest)
Under−five mortality rate (deaths per 1000 livebirths)
Quintile−specific U5MR
National−level U5MR
Number of quintile−specific Under−five deaths
Ratio of quintile−specific U5MR to national−level U5MR (log−scale)
MICS Indirect (2006)
Country estimate
Global expected
Proportion of national−level under−five deaths in quintile (%)

Tajikistan

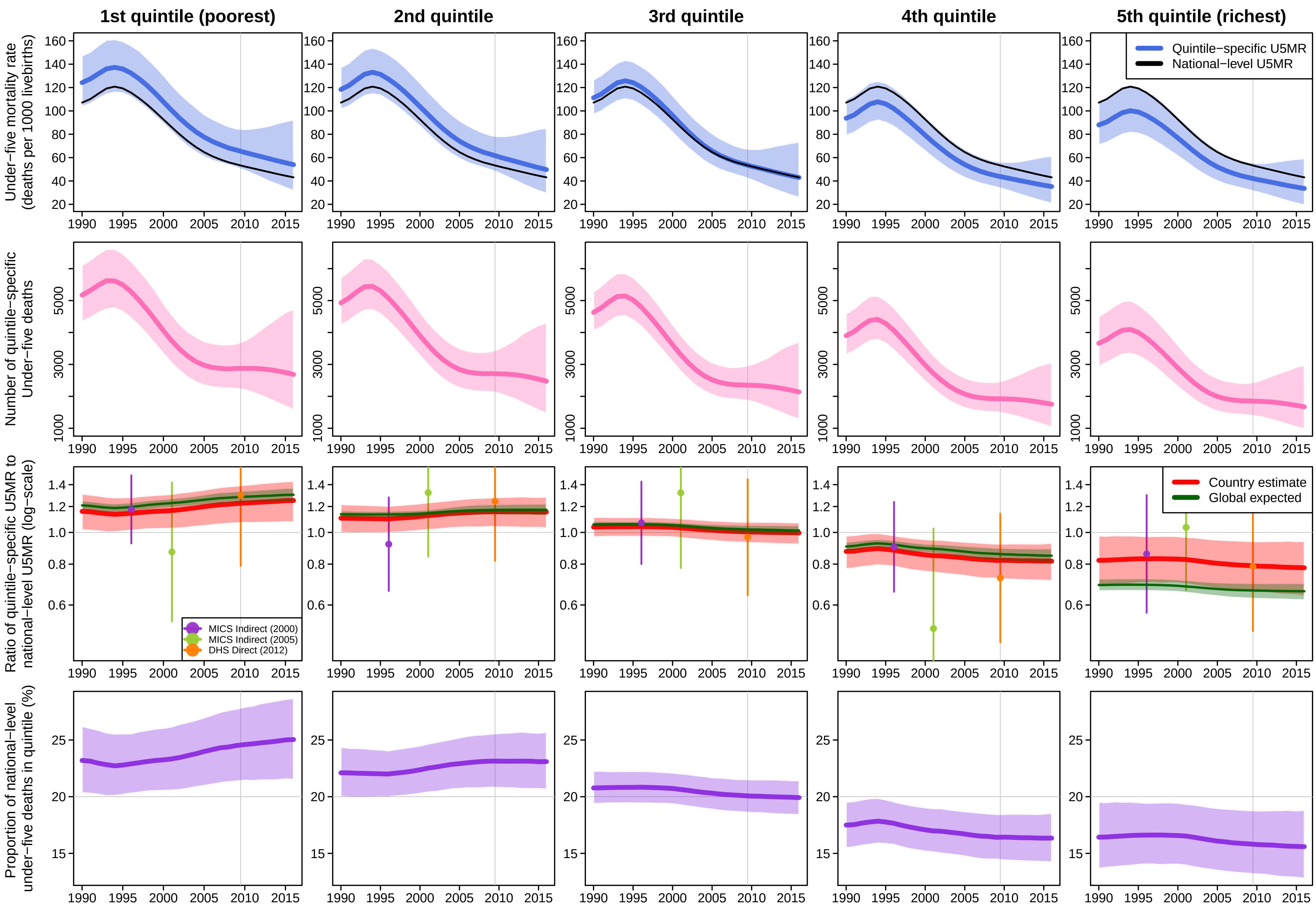
1st quintile (poorest)
2nd quintile
3rd quintile
4th quintile
5th quintile (richest)
Quintile−specific U5MR
National−level U5MR
Under−five mortality rate (deaths per 1000 livebirths)
Number of quintile−specific Under−five deaths
Ratio of quintile−specific U5MR to national−level U5MR (log−scale)
Country estimate
Global expected
MICS Indirect (2000)
MICS Indirect (2005)
DHS Direct (2012)
Proportion of national−level under−five deaths in quintile (%)

Thailand

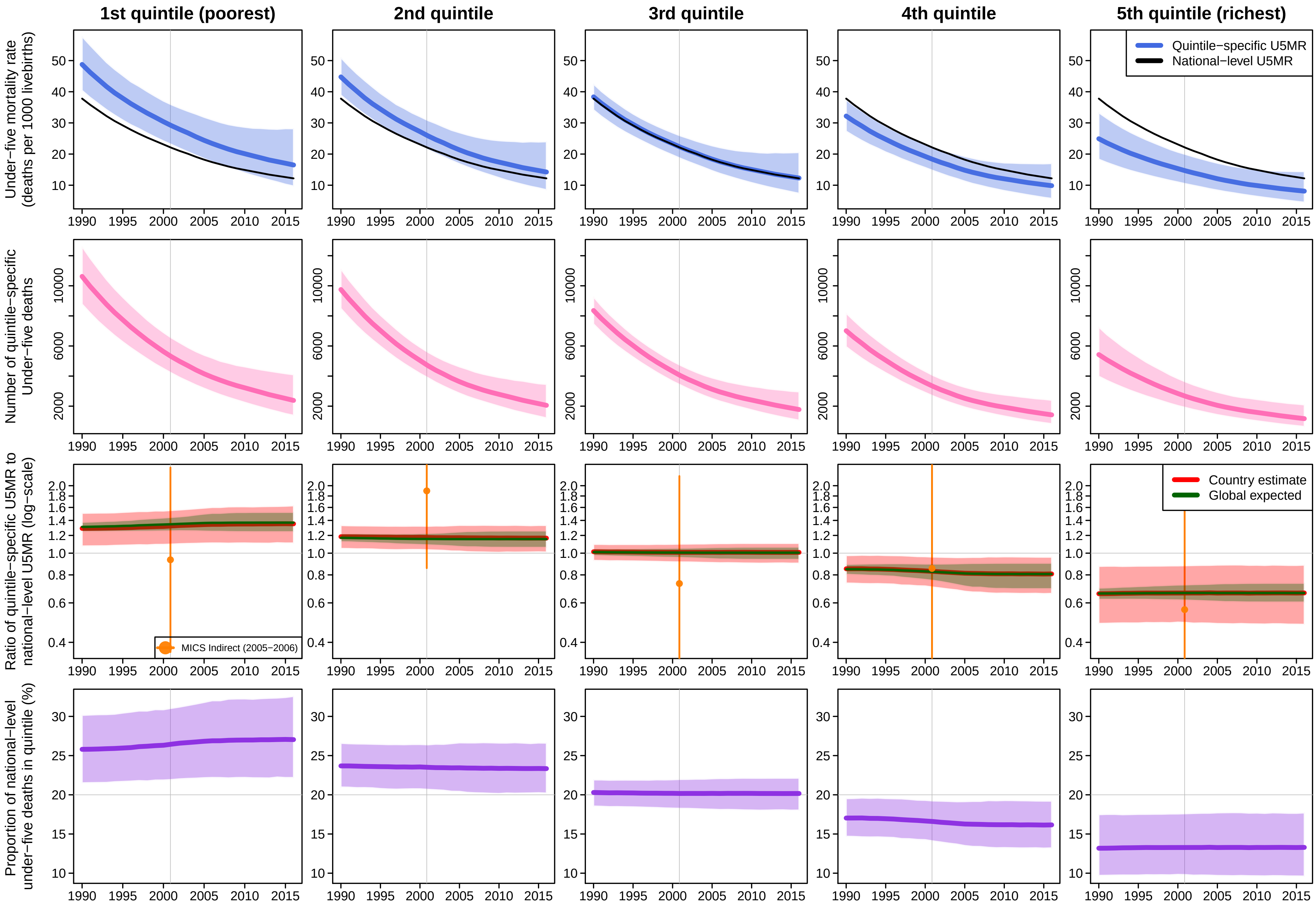

1st quintile (poorest)
2nd quintile
3rd quintile
4th quintile
5th quintile (richest)
Under−five mortality rate (deaths per 1000 livebirths)
Quintile−specific U5MR
National−level U5MR
Number of quintile−specific Under−five deaths
Ratio of quintile−specific U5MR to national−level U5MR (log−scale)
Country estimate
Global expected
MICS Indirect (2005−2006)
Proportion of national−level under−five deaths in quintile (%)

The former Yugoslav Republic of Macedonia

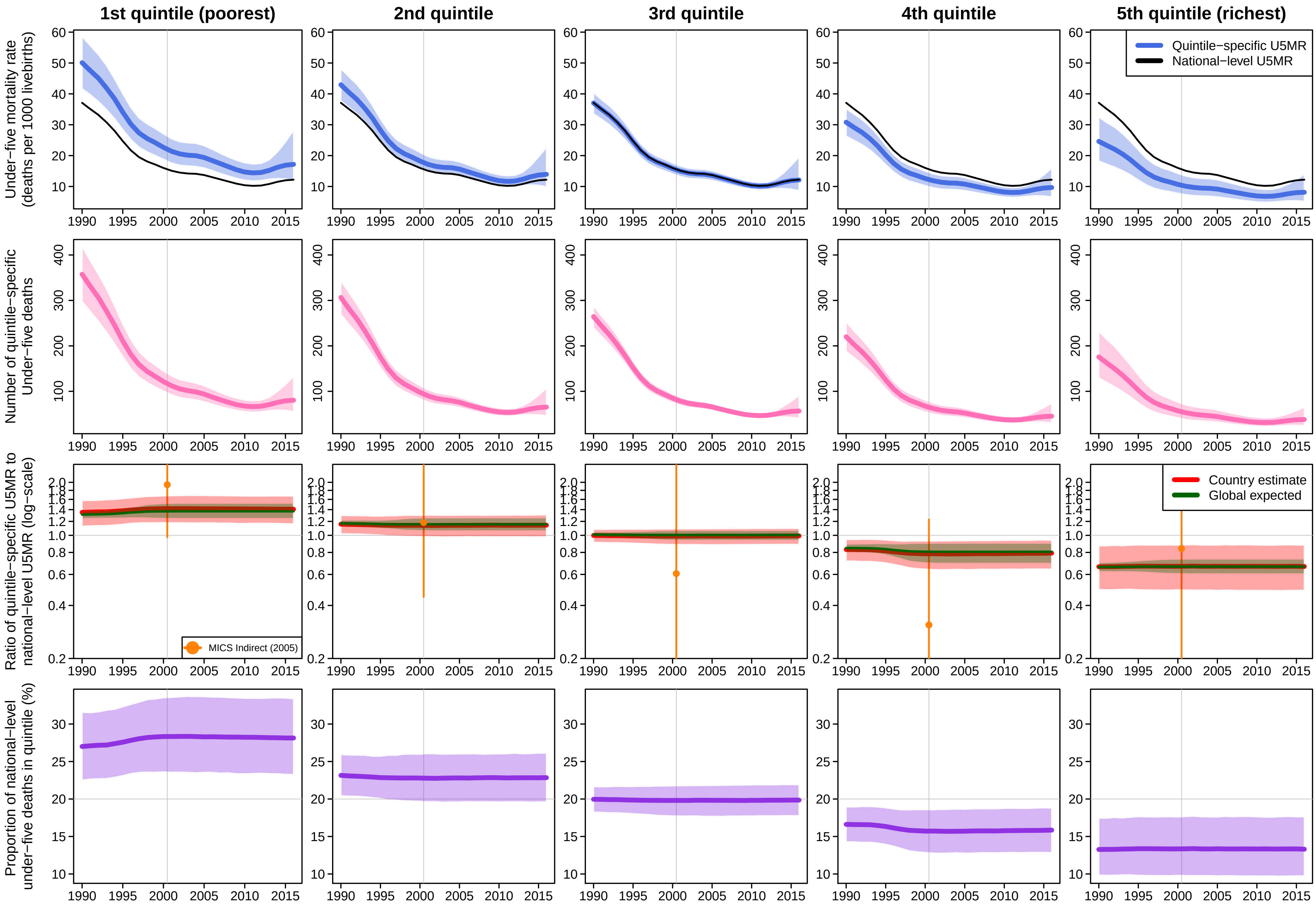

Gambia

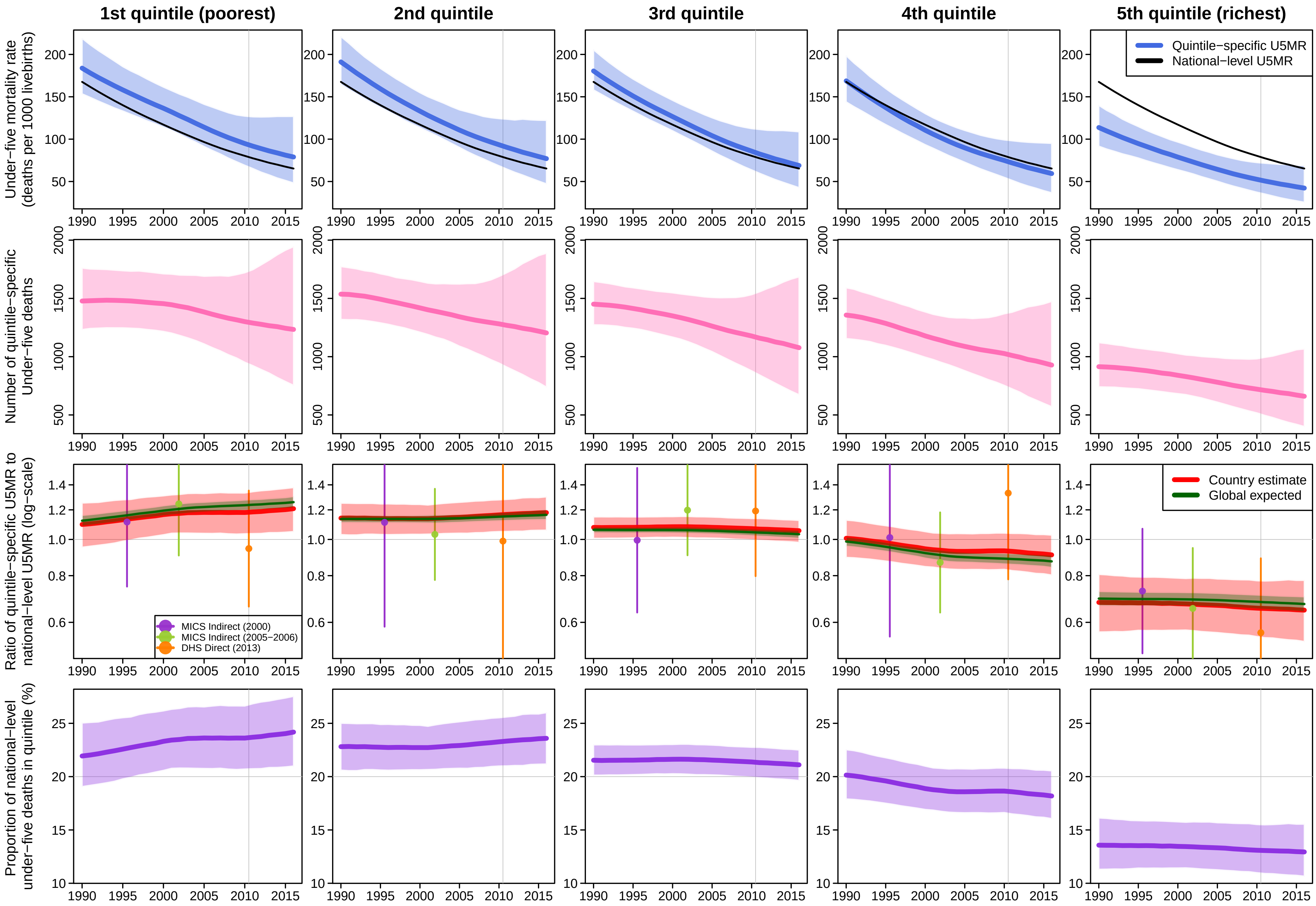

1st quintile (poorest)
2nd quintile
3rd quintile
4th quintile
5th quintile (richest)
Under−five mortality rate (deaths per 1000 livebirths)
Quintile−specific U5MR
National−level U5MR
Number of quintile−specific Under−five deaths
Ratio of quintile−specific U5MR to national−level U5MR (log−scale)
MICS Indirect (2000)
MICS Indirect (2005−2006)
DHS Direct (2013)
Country estimate
Global expected
Proportion of national−level under−five deaths in quintile (%)

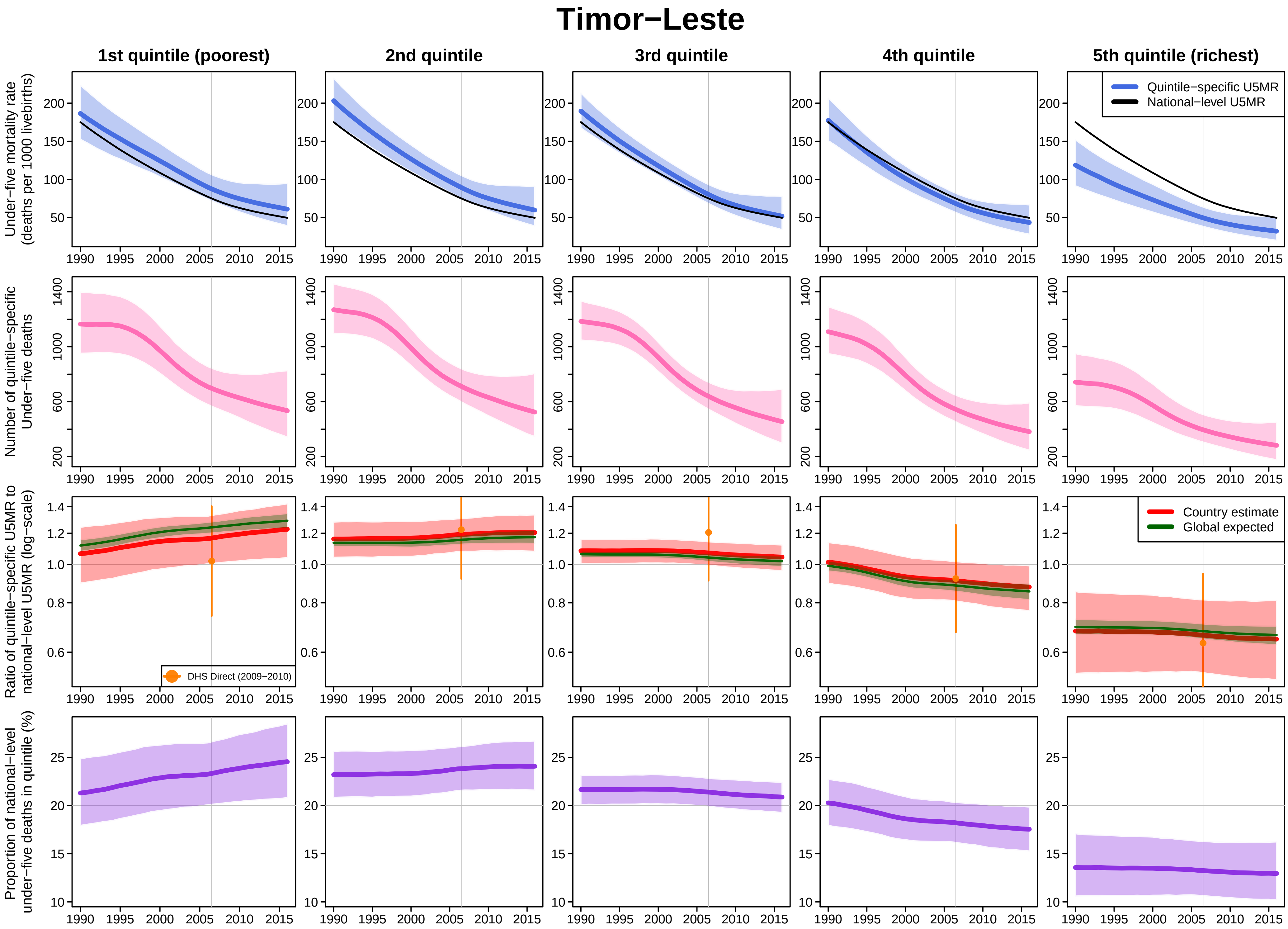
Timor−Leste
1st quintile (poorest)
2nd quintile
3rd quintile
4th quintile
5th quintile (richest)
Under−five mortality rate (deaths per 1000 livebirths)
Quintile−specific U5MR
National−level U5MR
Number of quintile−specific Under−five deaths
Ratio of quintile−specific U5MR to national−level U5MR (log−scale)
Country estimate
Global expected
DHS Direct (2009−2010)
Proportion of national−level under−five deaths in quintile (%)

Togo

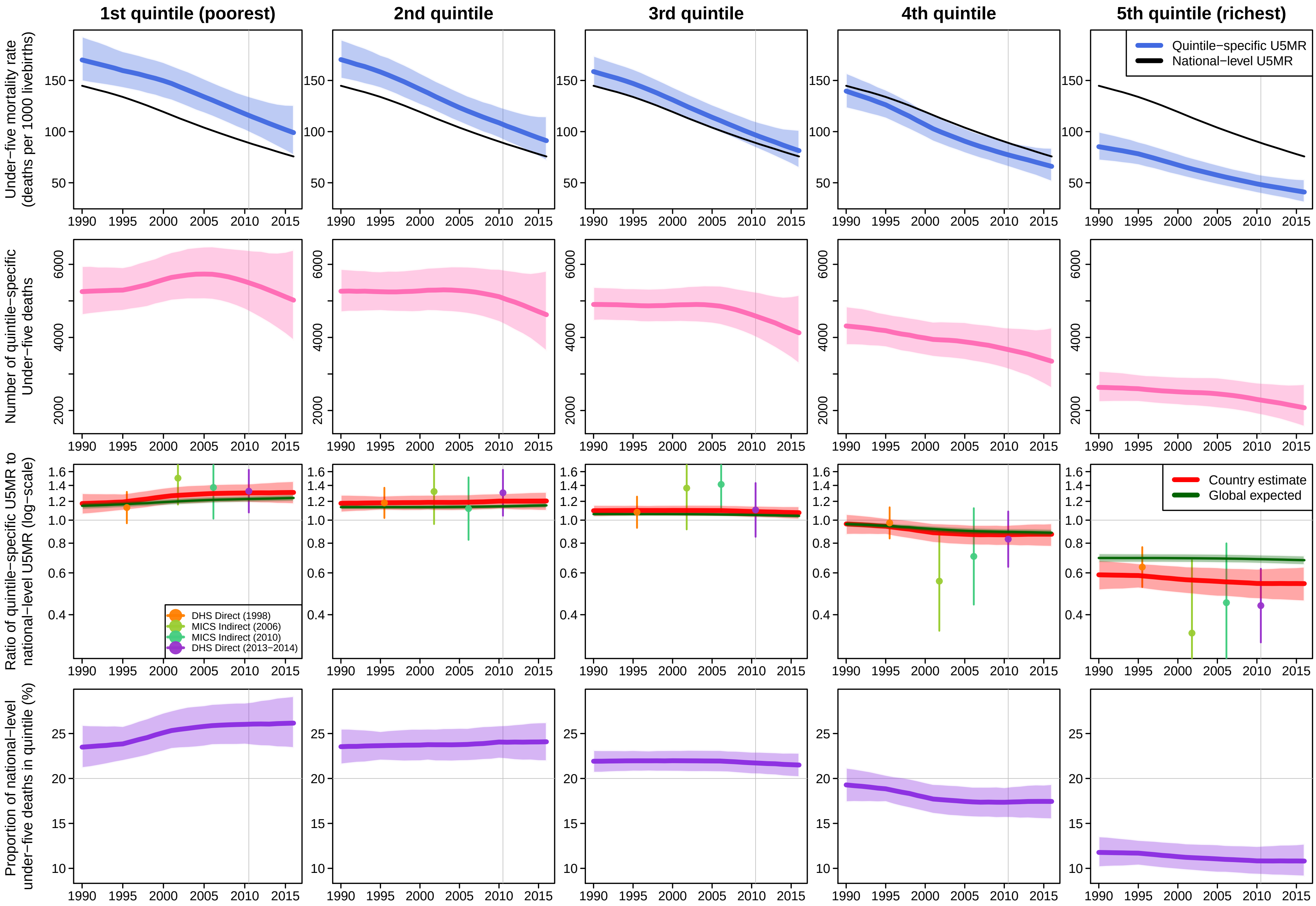
1st quintile (poorest)
2nd quintile
3rd quintile
4th quintile
5th quintile (richest)
Quintile−specific U5MR
National−level U5MR
Under−five mortality rate (deaths per 1000 livebirths)
Number of quintile−specific Under−five deaths
Ratio of quintile−specific U5MR to national−level U5MR (log−scale)
Country estimate
Global expected
DHS Direct (1998)
MICS Indirect (2006)
MICS Indirect (2010)
DHS Direct (2013−2014)
Proportion of national−level under−five deaths in quintile (%)

Tunisia

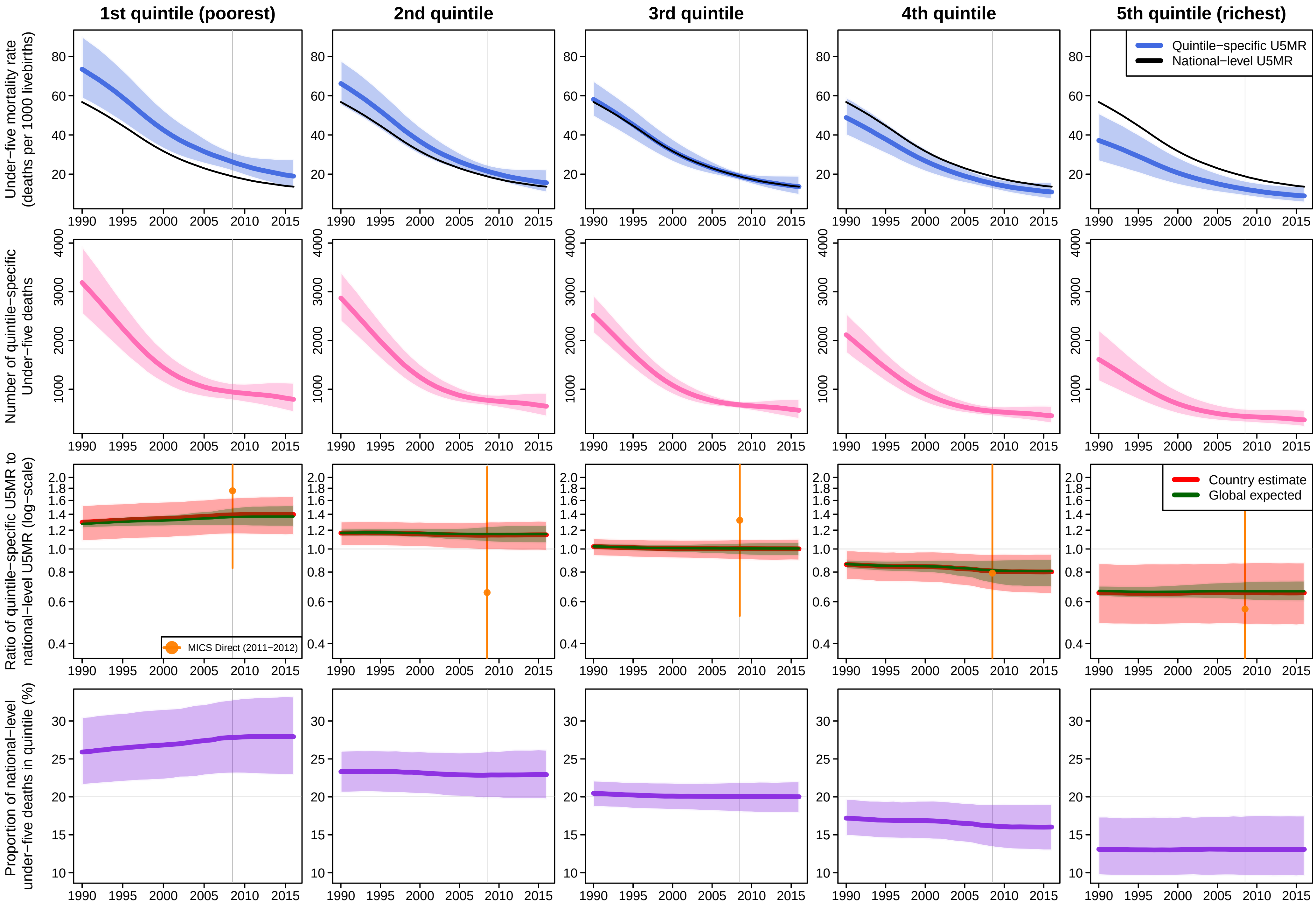
1st quintile (poorest)
2nd quintile
3rd quintile
4th quintile
5th quintile (richest)
Under−five mortality rate (deaths per 1000 livebirths)
Quintile−specific U5MR
National−level U5MR
Number of quintile−specific Under−five deaths
Ratio of quintile−specific U5MR to national−level U5MR (log−scale)
Country estimate
Global expected
MICS Direct (2011−2012)
Proportion of national−level under−five deaths in quintile (%)
1990
1995
2000
2005
2010
2015

Turkey

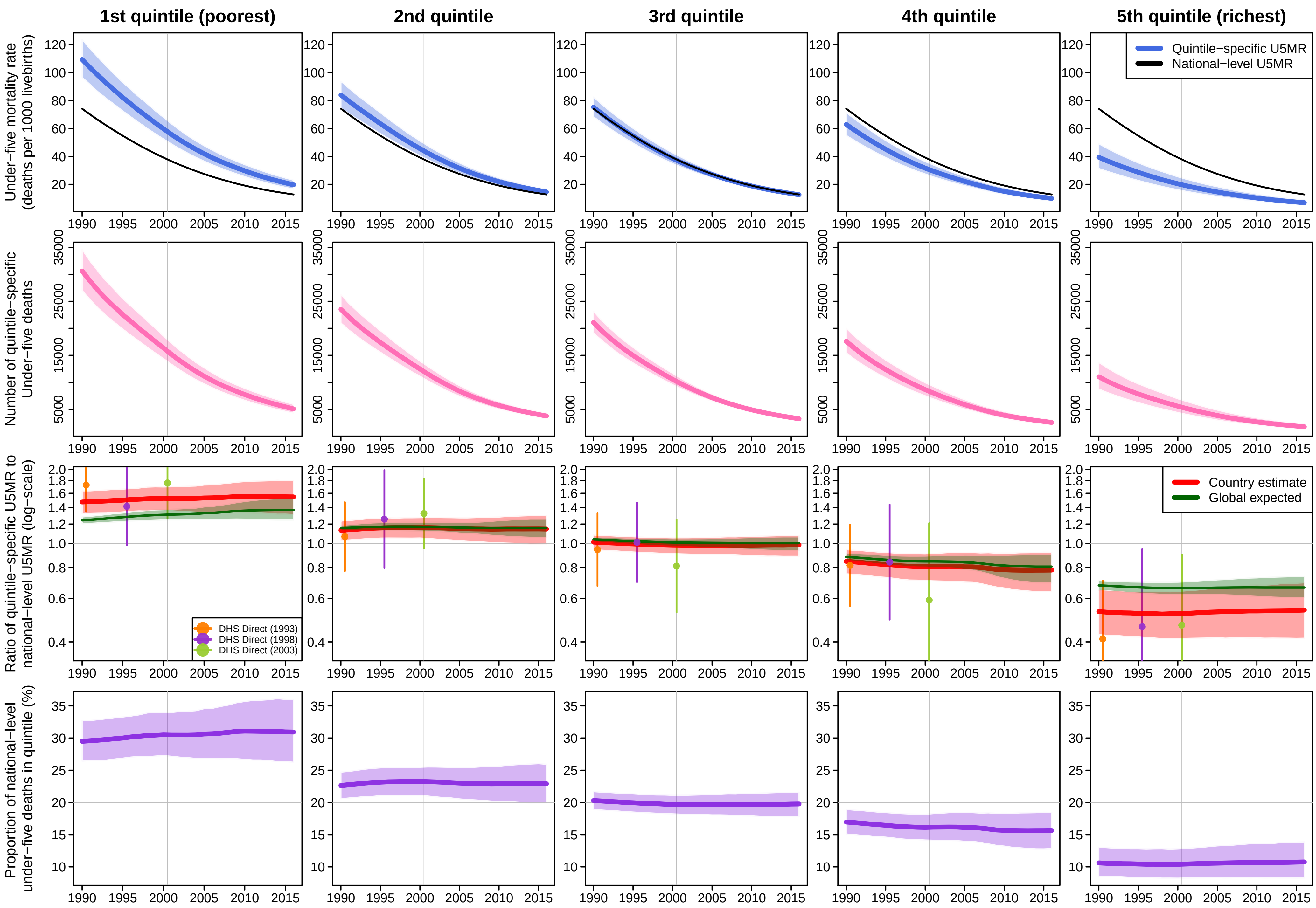
1st quintile (poorest)
2nd quintile
3rd quintile
4th quintile
5th quintile (richest)
Under−five mortality rate (deaths per 1000 livebirths)
Quintile−specific U5MR
National−level U5MR
Number of quintile−specific Under−five deaths
Ratio of quintile−specific U5MR to national−level U5MR (log−scale)
Country estimate
Global expected
DHS Direct (1993)
DHS Direct (1998)
DHS Direct (2003)
Proportion of national−level under−five deaths in quintile (%)

Turkmenistan

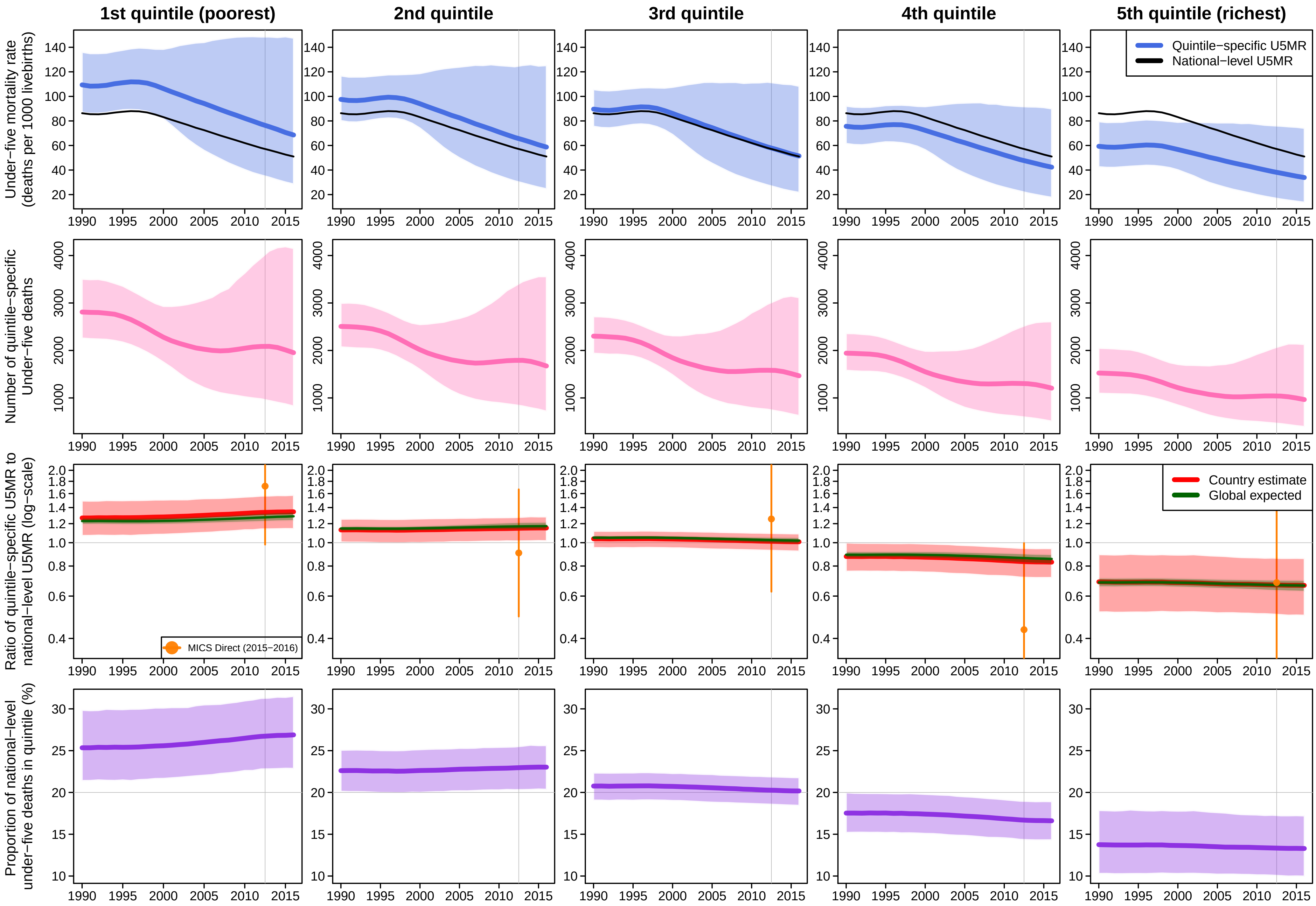

Uganda

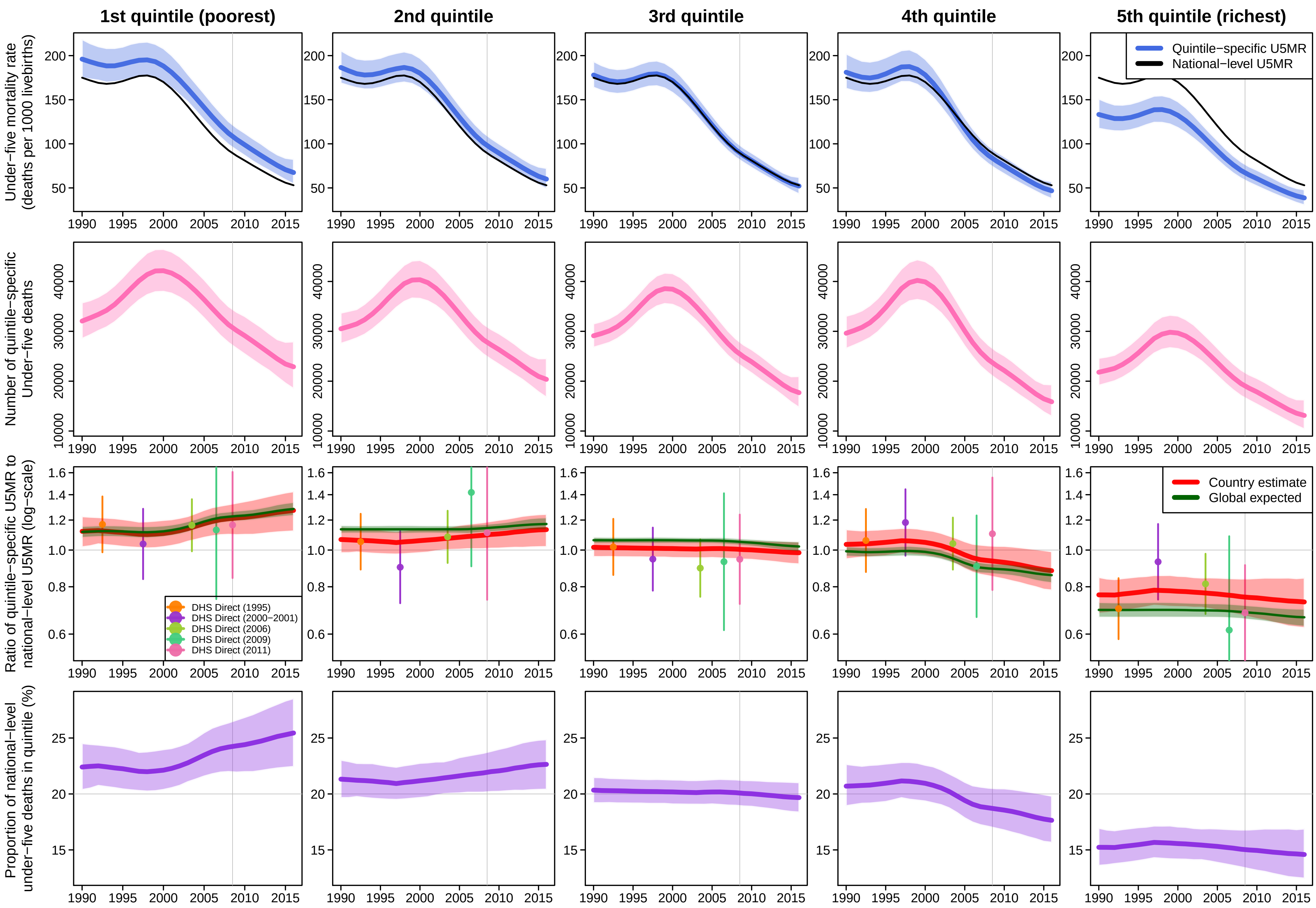

1st quintile (poorest)
2nd quintile
3rd quintile
4th quintile
5th quintile (richest)
Under−five mortality rate (deaths per 1000 livebirths)
Quintile−specific U5MR
National−level U5MR
Number of quintile−specific Under−five deaths
Ratio of quintile−specific U5MR to national−level U5MR (log−scale)
Country estimate
Global expected
DHS Direct (1995)
DHS Direct (2000−2001)
DHS Direct (2006)
DHS Direct (2009)
DHS Direct (2011)
Proportion of national−level under−five deaths in quintile (%)

Ukraine

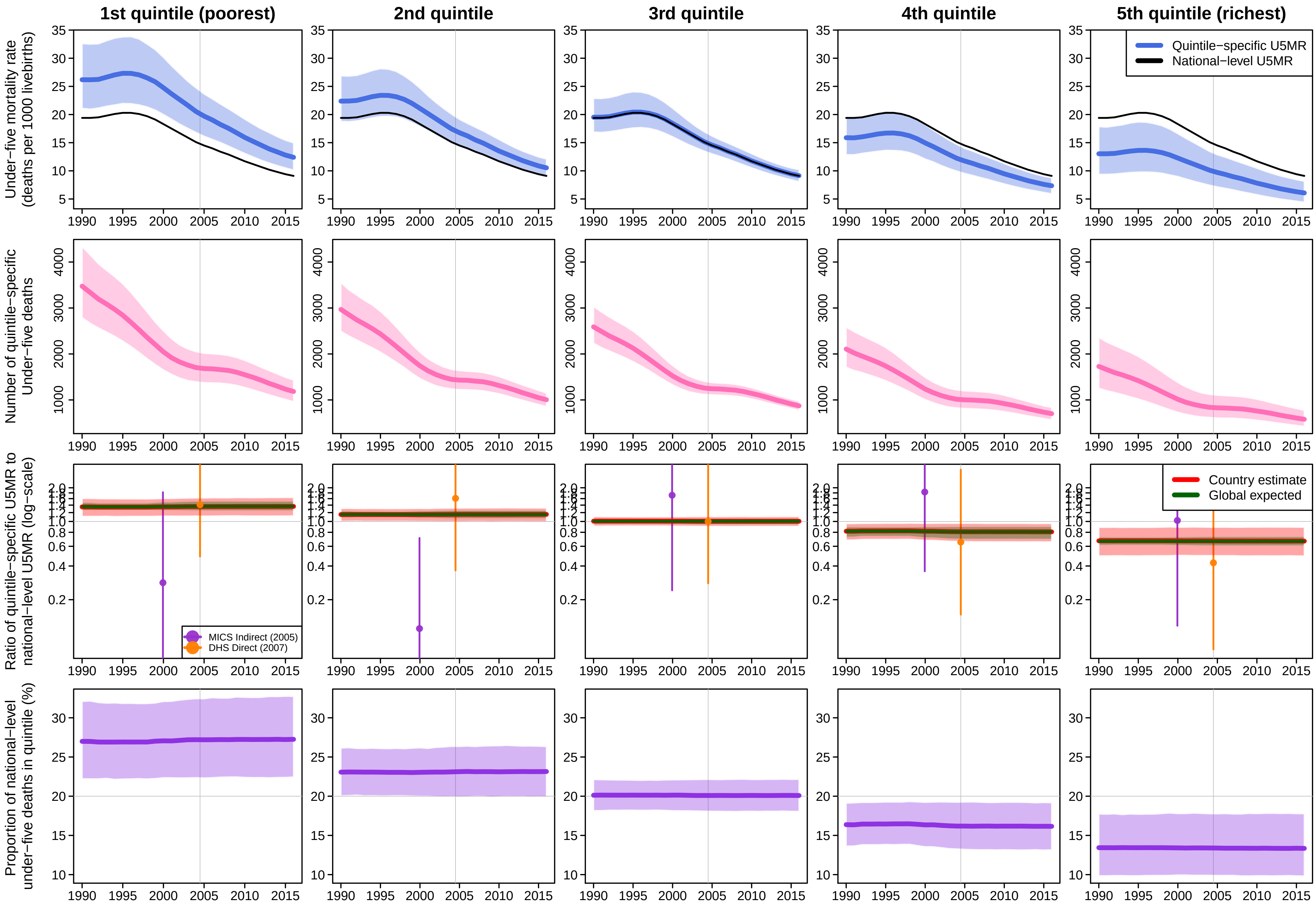
1st quintile (poorest)
2nd quintile
3rd quintile
4th quintile
5th quintile (richest)
Under−five mortality rate (deaths per 1000 livebirths)
Quintile−specific U5MR
National−level U5MR
Number of quintile−specific Under−five deaths
Ratio of quintile−specific U5MR to national−level U5MR (log−scale)
Country estimate
Global expected
MICS Indirect (2005)
DHS Direct (2007)
Proportion of national−level under−five deaths in quintile (%)

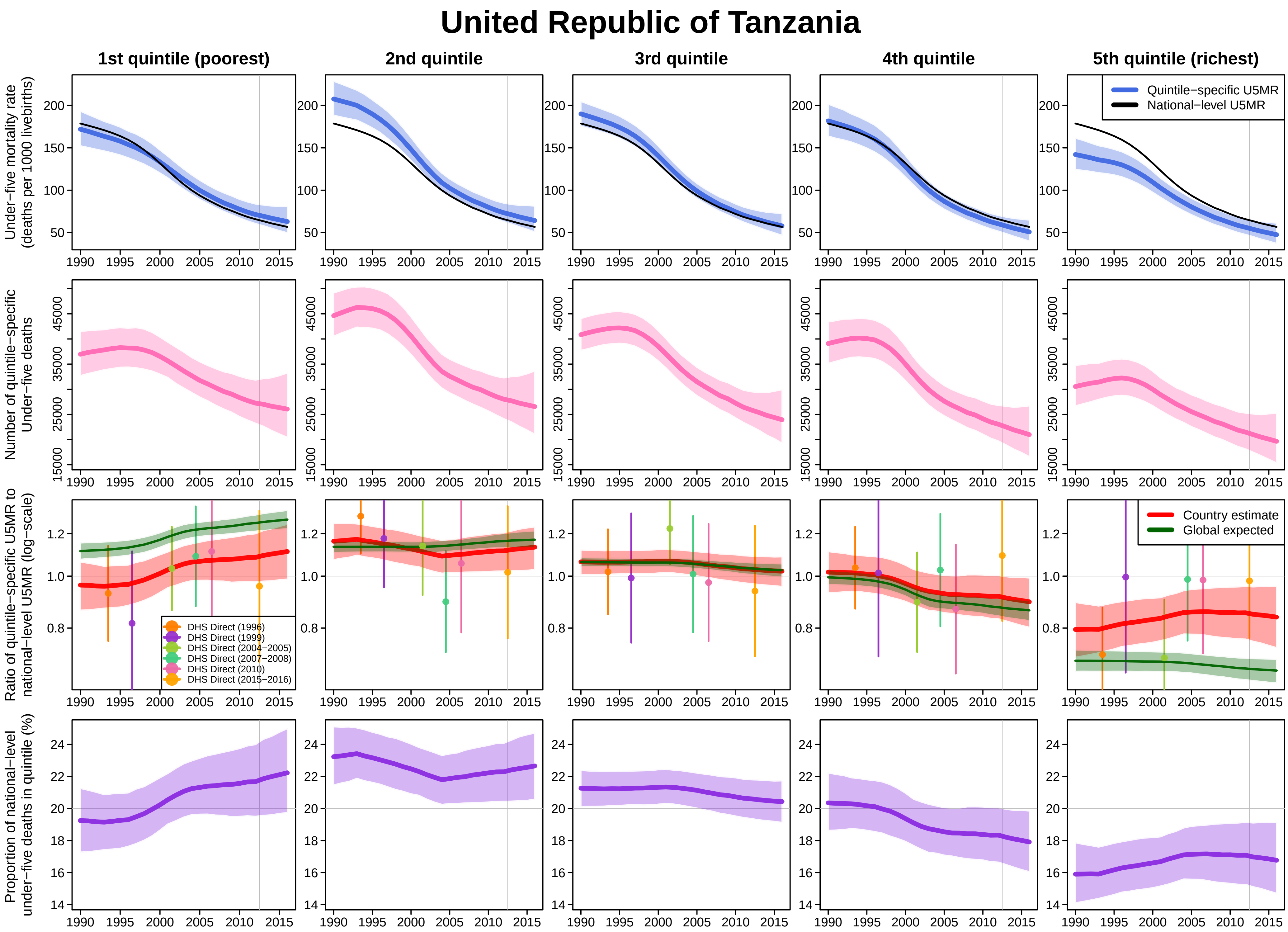
United Republic of Tanzania
1st quintile (poorest)
2nd quintile
3rd quintile
4th quintile
5th quintile (richest)
Under−five mortality rate
(deaths per 1000 livebirths)
Quintile−specific U5MR
National−level U5MR
Number of quintile−specific
Under−five deaths
Ratio of quintile−specific U5MR to
national−level U5MR (log−scale)
Country estimate
Global expected
DHS Direct (1996)
DHS Direct (1999)
DHS Direct (2004−2005)
DHS Direct (2007−2008)
DHS Direct (2010)
DHS Direct (2015−2016)
Proportion of national−level
under−five deaths in quintile (%)

Uzbekistan

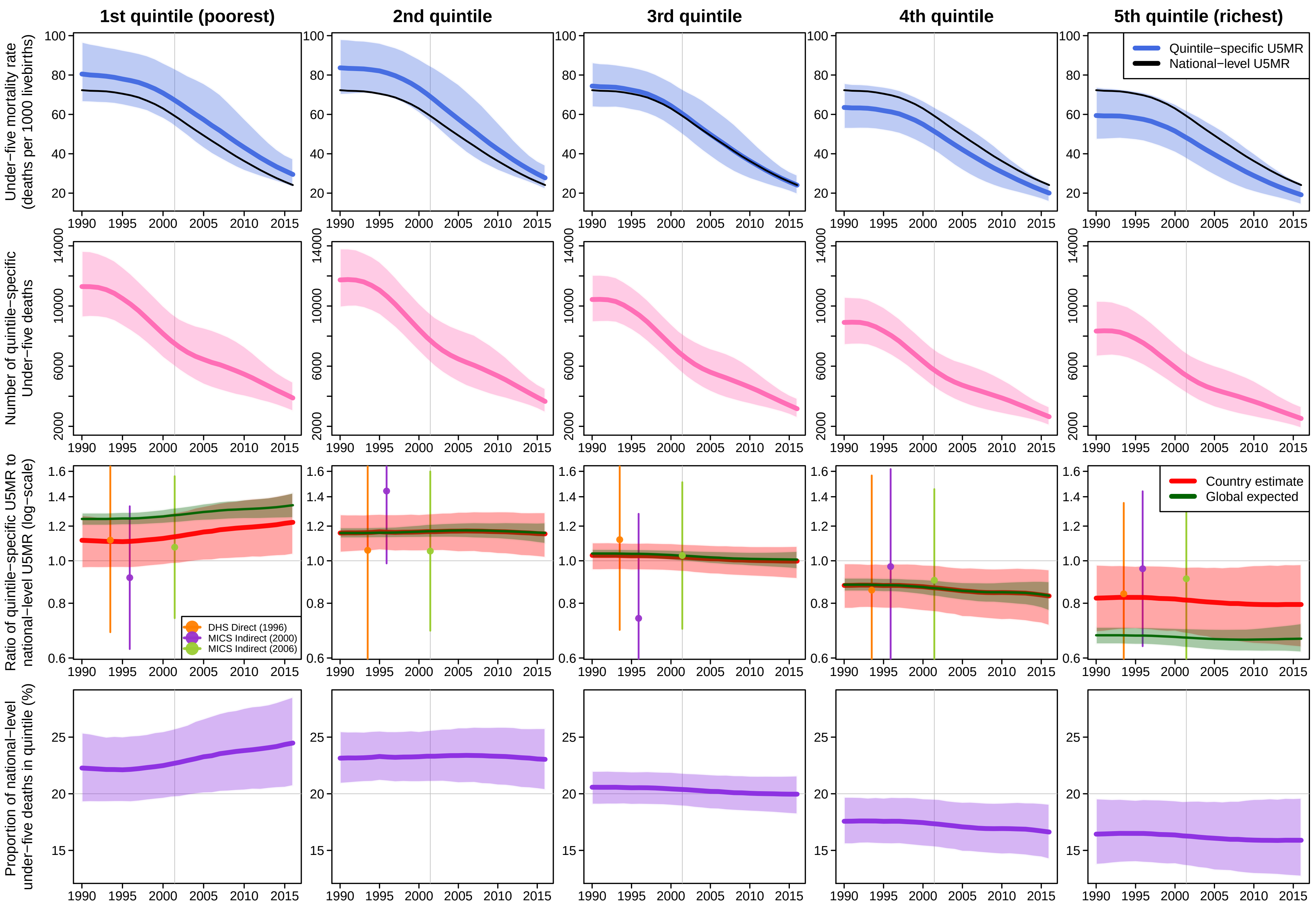

1st quintile (poorest)
2nd quintile
3rd quintile
4th quintile
5th quintile (richest)
Under−five mortality rate (deaths per 1000 livebirths)
Quintile−specific U5MR
National−level U5MR
Number of quintile−specific Under−five deaths
Ratio of quintile−specific U5MR to national−level U5MR (log−scale)
Country estimate
Global expected
DHS Direct (1996)
MICS Indirect (2000)
MICS Indirect (2006)
Proportion of national−level under−five deaths in quintile (%)

Vanuatu

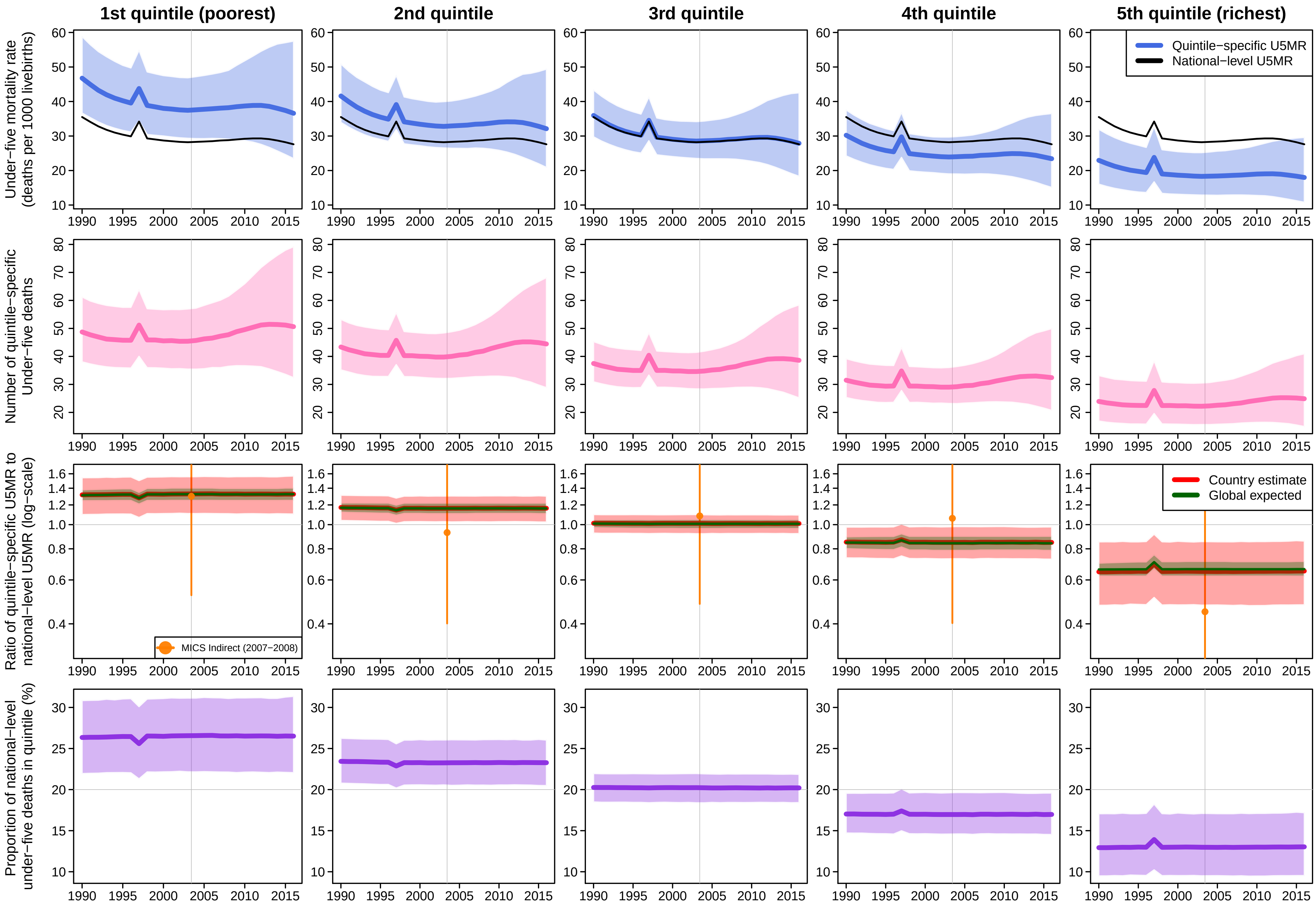
1st quintile (poorest)
2nd quintile
3rd quintile
4th quintile
5th quintile (richest)
Under−five mortality rate (deaths per 1000 livebirths)
Quintile−specific U5MR
National−level U5MR
Number of quintile−specific Under−five deaths
Ratio of quintile−specific U5MR to national−level U5MR (log−scale)
Country estimate
Global expected
MICS Indirect (2007−2008)
Proportion of national−level under−five deaths in quintile (%)
1990
1995
2000
2005
2010
2015

Viet Nam

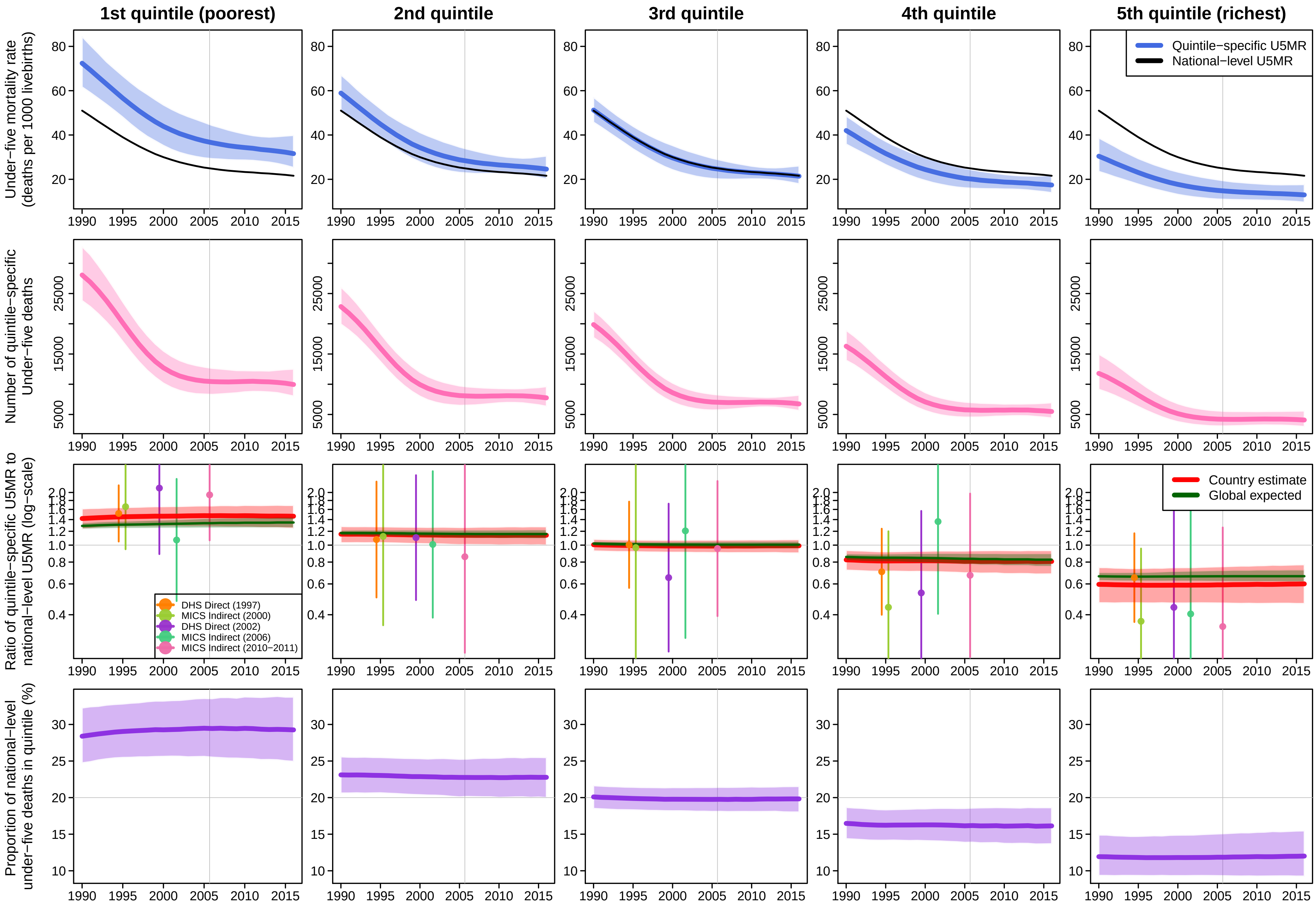

1st quintile (poorest)
2nd quintile
3rd quintile
4th quintile
5th quintile (richest)
Under−five mortality rate (deaths per 1000 livebirths)
Quintile−specific U5MR
National−level U5MR
Number of quintile−specific Under−five deaths
Ratio of quintile−specific U5MR to national−level U5MR (log−scale)
Country estimate
Global expected
DHS Direct (1997)
MICS Indirect (2000)
DHS Direct (2002)
MICS Indirect (2006)
MICS Indirect (2010−2011)
Proportion of national−level under−five deaths in quintile (%)

Yemen

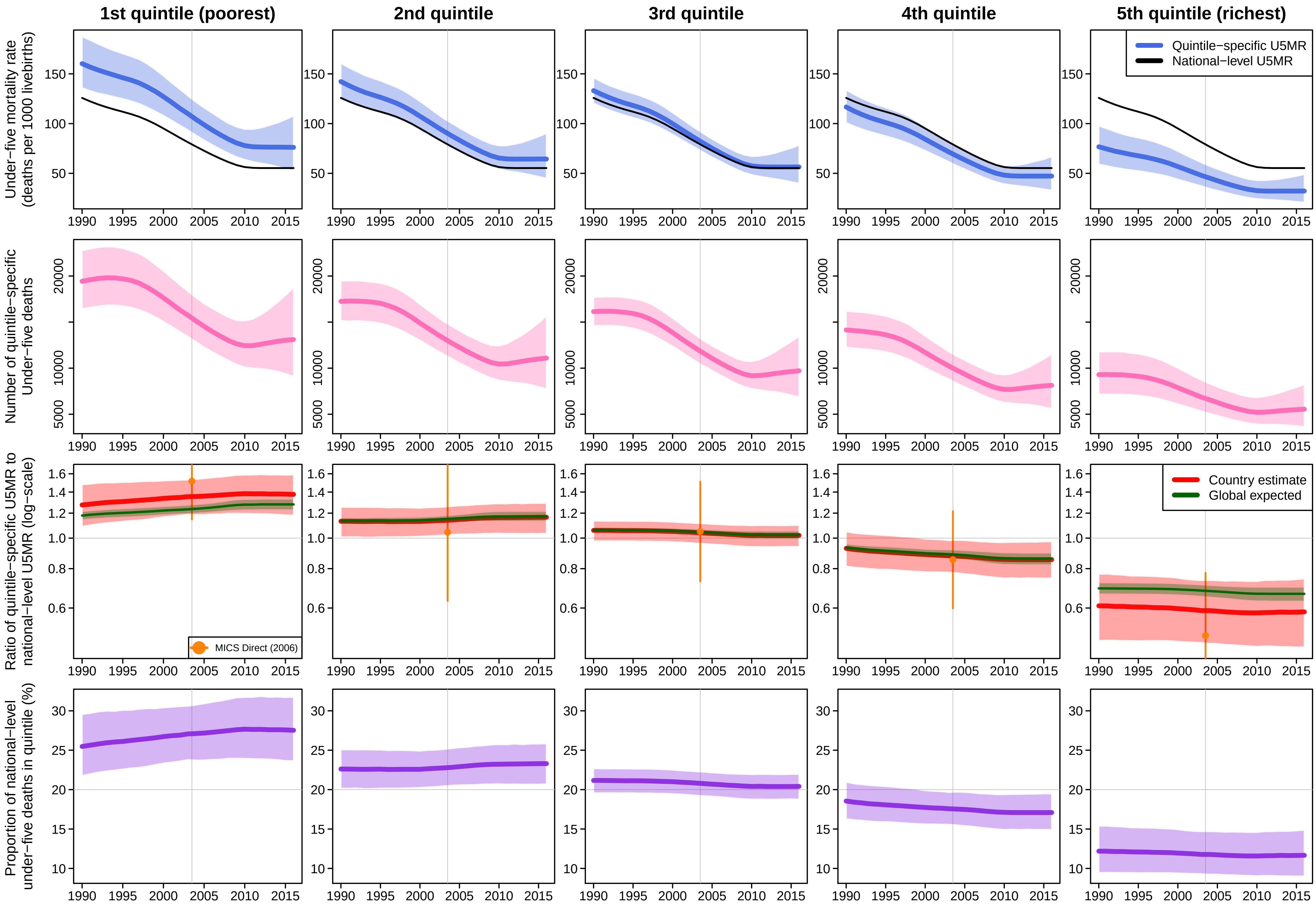

1st quintile (poorest)
2nd quintile
3rd quintile
4th quintile
5th quintile (richest)
Under−five mortality rate (deaths per 1000 livebirths)
Quintile−specific U5MR
National−level U5MR
Number of quintile−specific Under−five deaths
Ratio of quintile−specific U5MR to national−level U5MR (log−scale)
Country estimate
Global expected
MICS Direct (2006)
Proportion of national−level under−five deaths in quintile (%)

Zambia

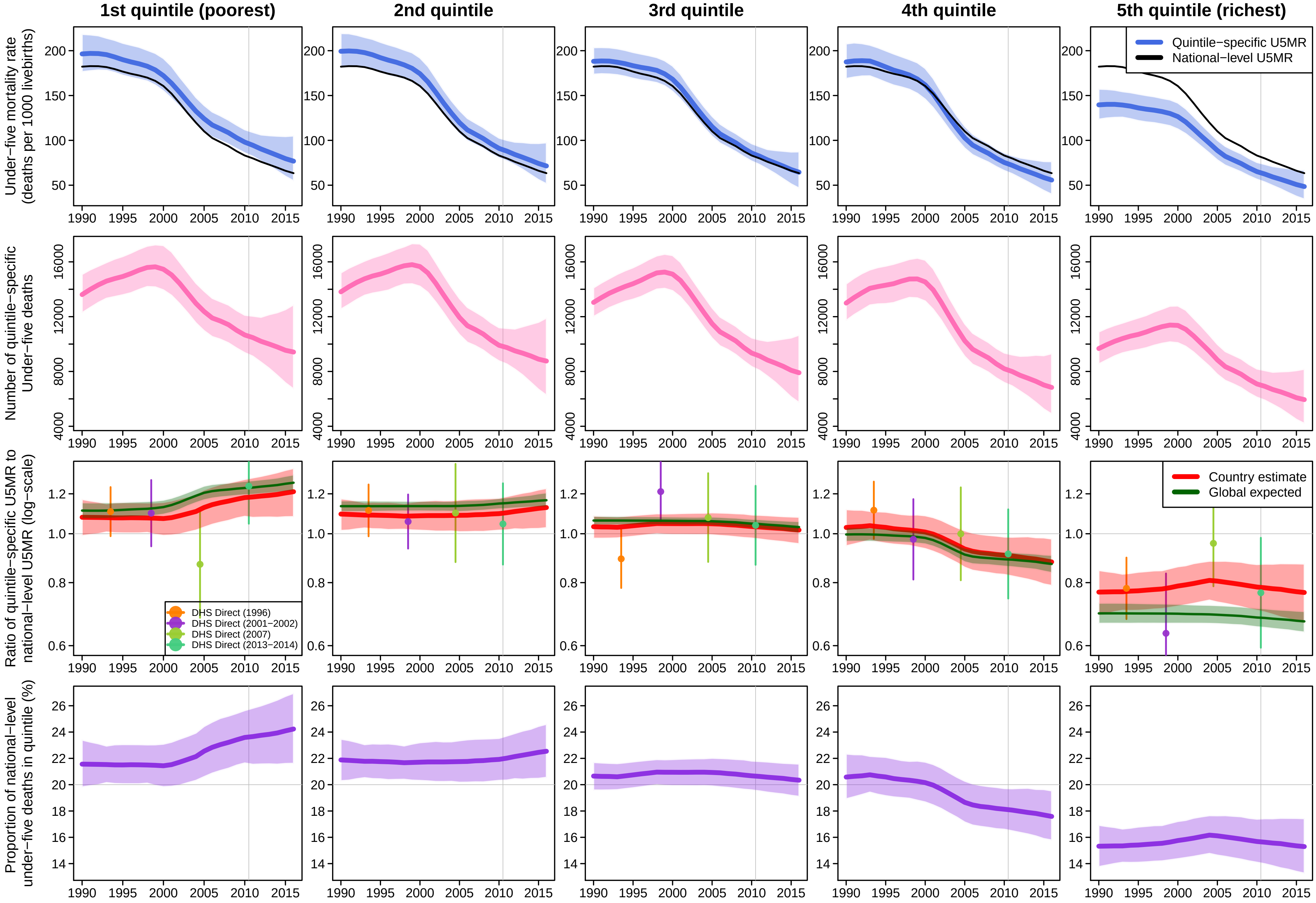

1st quintile (poorest)
2nd quintile
3rd quintile
4th quintile
5th quintile (richest)
Under−five mortality rate (deaths per 1000 livebirths)
Number of quintile−specific Under−five deaths
Ratio of quintile−specific U5MR to national−level U5MR (log−scale)
Proportion of national−level under−five deaths in quintile (%)
Quintile−specific U5MR
National−level U5MR
Country estimate
Global expected
DHS Direct (1996)
DHS Direct (2001−2002)
DHS Direct (2007)
DHS Direct (2013−2014)

Zimbabwe

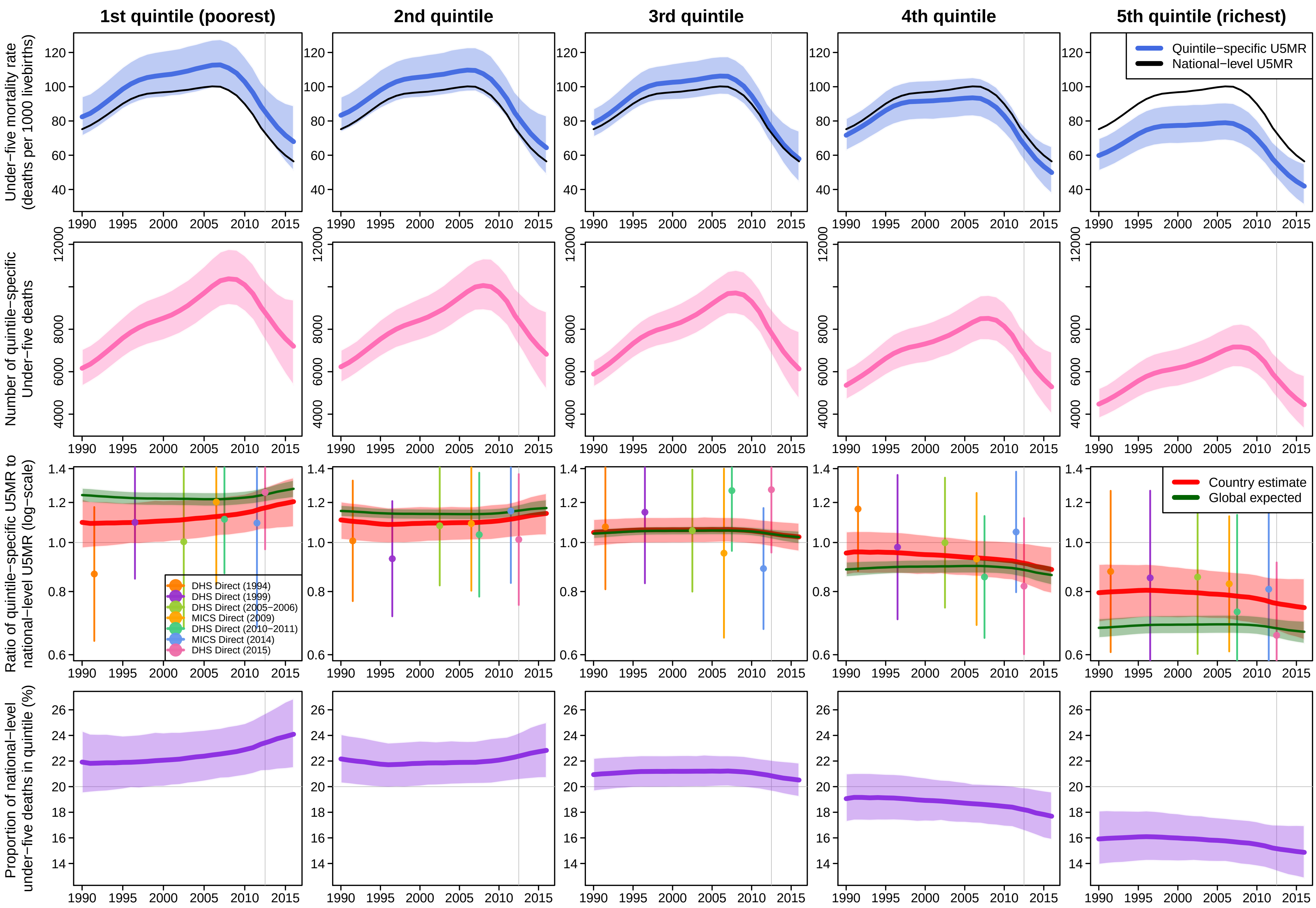

1st quintile (poorest)
2nd quintile
3rd quintile
4th quintile
5th quintile (richest)
Under−five mortality rate (deaths per 1000 livebirths)
Quintile−specific U5MR
National−level U5MR
Number of quintile−specific Under−five deaths
Ratio of quintile−specific U5MR to national−level U5MR (log−scale)
DHS Direct (1994)
DHS Direct (1999)
DHS Direct (2005−2006)
MICS Direct (2009)
DHS Direct (2010−2011)
MICS Direct (2014)
DHS Direct (2015)
Country estimate
Global expected
Proportion of national−level under−five deaths in quintile (%)
